# Supplementary material for: Whole-Blood Transcriptional Profiles Enable Early Prediction of the Presence of Coronary Atherosclerosis and High-Risk Plaque Features at Coronary CT Angiography
Source: Biomedicines. 2022 Jun 2;10(6):1309. doi: 10.3390/biomedicines10061309 (PMC9219643; doi:10.3390/biomedicines10061309)
Supplement: Supplementary file 1 [file biomedicines-10-01309-s001.zip › biomedicines-1685259-figures.pdf]

biomedicines

MDPI

Supplementary Materials: Whole-Blood Transcriptional Profiles Enable Early Prediction of the Presence of Coronary Atherosclerosis and High-Risk Plaque Features at Coronary CT Angiography

Daniele Andreini, Eleonora Melotti, Chiara Vavassori, Mattia Chiesa, Luca Piacentini, Edoardo Conte, Saima Mushtaq, Martina Manzoni, Eleonora Cipriani, Paolo Ravagnani, Antonio L. Bartorelli and Gualtiero I. Colombo

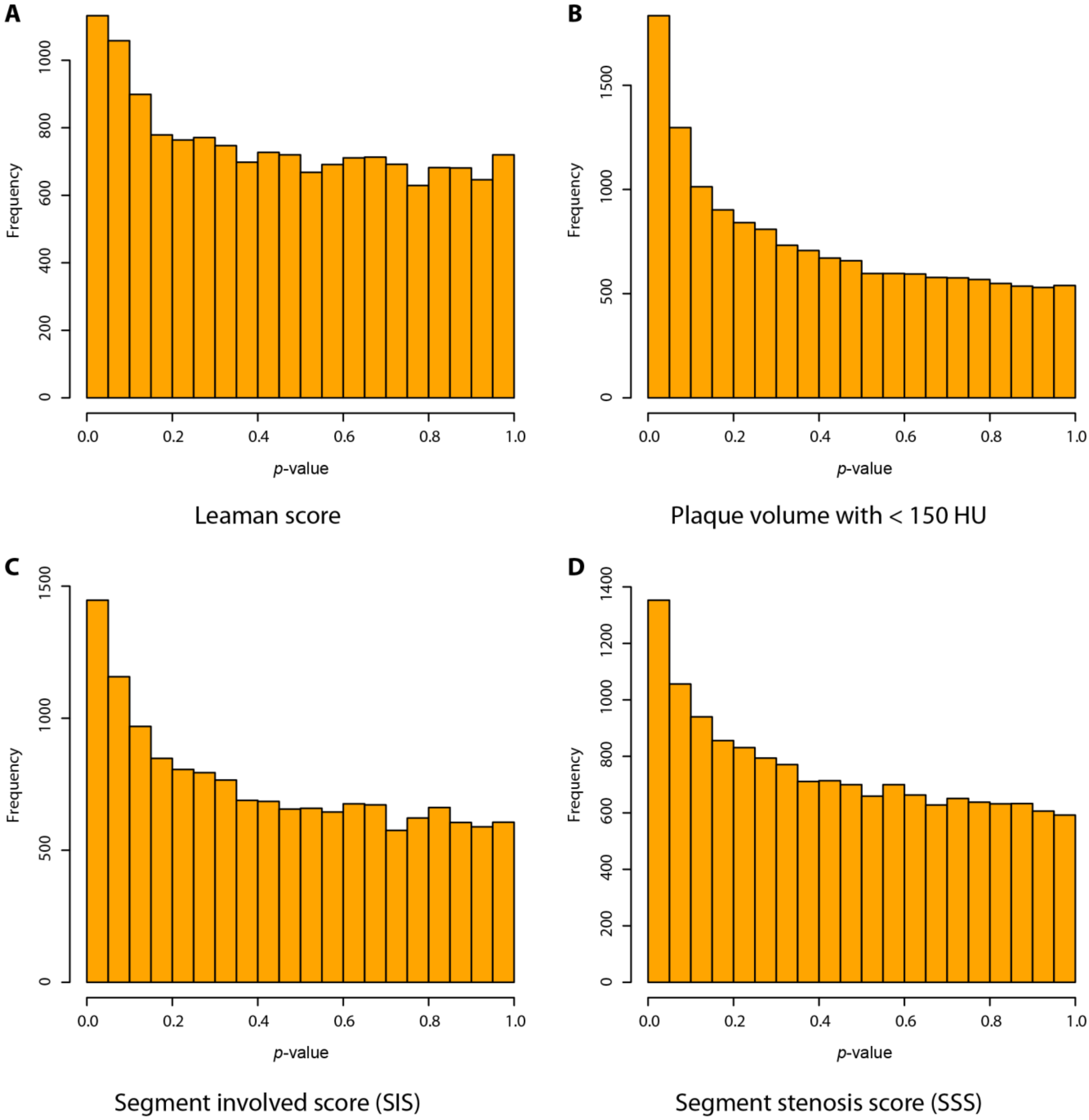

Figure S1. Histograms of the distribution of *p*-values for each regression analysis with reliable results. *p*-Value distribution for (**A**) the Leaman score, (**B**) plaque volumes with density < 150 HU, (**C**) segment involved score, and (**D**) segment stenosis score.

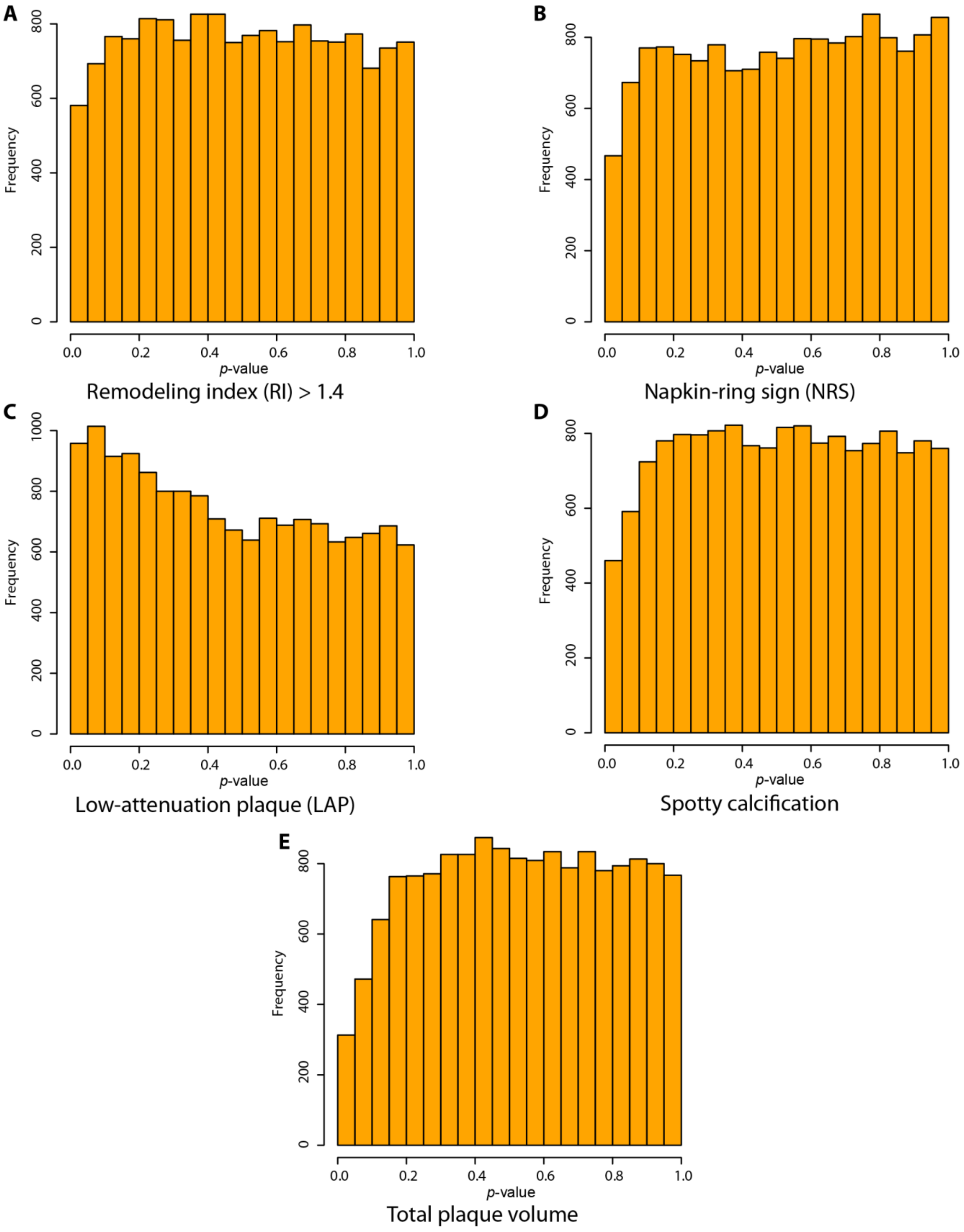

Figure S2. Histograms of the distribution of *p*-values for each regression analysis with inconsistent results; i.e., (**A**) remodeling index >1.4, (**B**) napkin-ring sign, (**C**) low-attenuation plaque, (**D**) spotty calcification, and (**E**) total plaque volume.

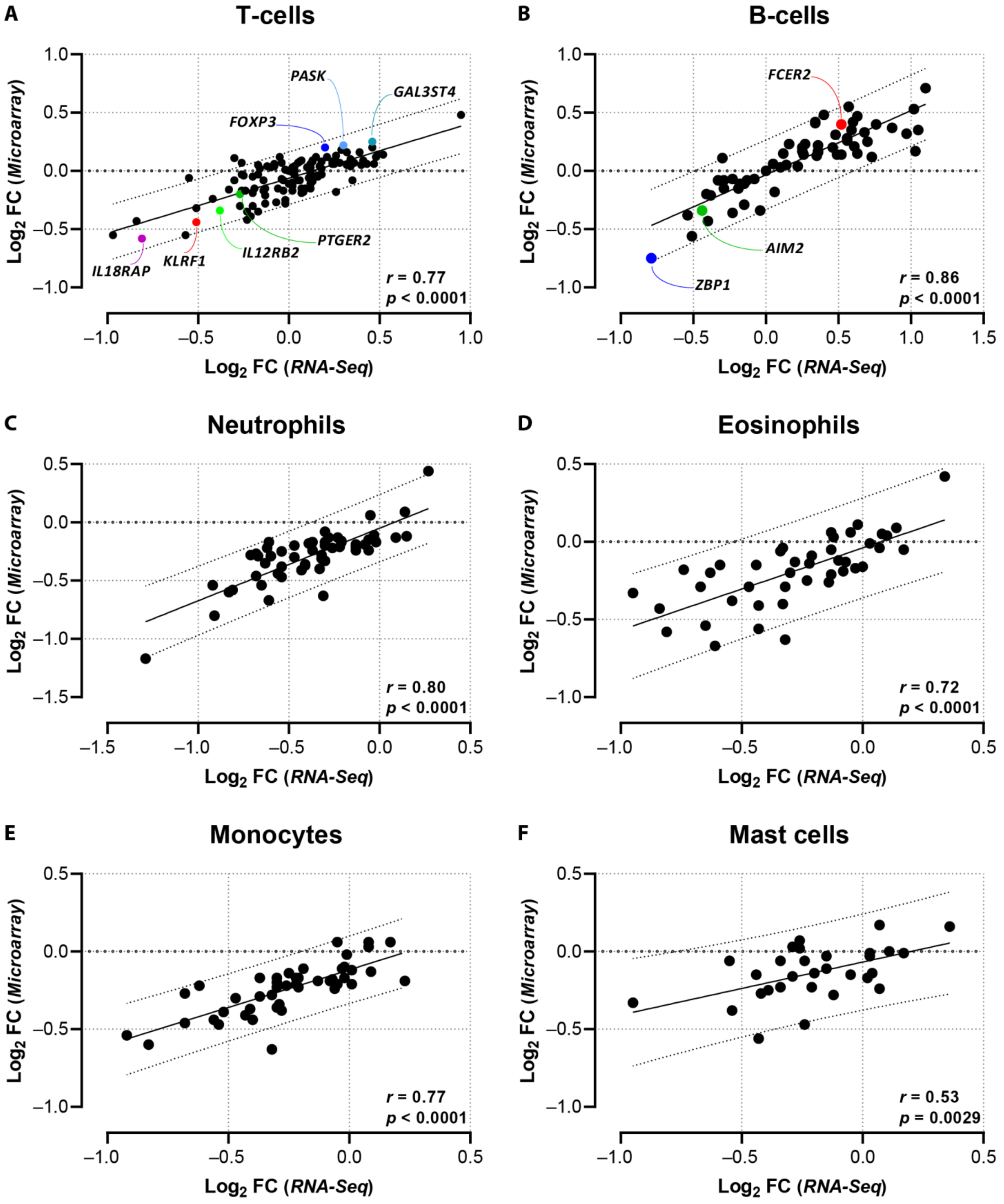

Figure S3. Technical validation of gene sets distinguishing human hematopoietic cell types. The expression levels of genes measured by RNA sequencing (RNA-Seq) were also assessed using high-density microarrays in a subgroup of matched patients (CAD n = 14 *vs.* noCAD n = 13). Mean differences (expressed as $\log_2$ fold-change (Log_2 FC)) in hematopoietic cell genes between the two groups were calculated for both datasets (RNA-Seq and microarray). Pearson's correlation coefficients (r) and significance levels (p) were computed to assess the degree of correlation of the $\log_2$ FC between the two datasets and confirm results through an independent testing technique. Data are

plotted as $\log_2$ FC in CAD *vs.* noCAD assessed by RNA-Seq (x-axis) or microarray (y-axis). The 95% confidence intervals of the trend lines are depicted by dashed lines. (**A**) Dot plot of the $\log_2$ FC of 129 T-cell related genes in CAD *vs.* noCAD assessed by RNA-Seq and microarray. This gene signature includes seven T-cell types and a unique gene for each subtype is highlighted with a color in the plot, namely: *KLRF1*, CD8 T cells; *GAL3ST4*, naive CD4 T cells; *PTGER2*, resting memory CD4 T cells; *IL12RB2*, activated memory CD4 T cells; *PASK*, T follicular helper cells; *FOXP3*, regulatory T cells (Tregs); and *IL18RAP*, gamma-delta ($\gamma\delta$) T cells. (**B**) Dot plot of the $\log_2$ FC of 70 B-cell related genes in CAD *vs.* noCAD assessed by RNA-Seq and microarray. This gene signature includes two B-cell types and plasma cells, and a unique gene for each subtype is highlighted with a color, namely: *FCER2*, naive B cells; *AIM2*, memory B cells; and *ZBP1*, plasma cells. The last four panels are dot plots of the $\log_2$ FC of (**C**) 56 neutrophil-related, (**D**) 43 eosinophil-related, (**E**) 50 monocyte-related, and (**F**) 30 mast-cell-related genes in CAD *vs.* noCAD patients as assessed by RNA-Seq and microarray.
