# Supplementary material for: Whole-Blood Transcriptional Profiles Enable Early Prediction of the Presence of Coronary Atherosclerosis and High-Risk Plaque Features at Coronary CT Angiography
Source: Biomedicines. 2022 Jun 2;10(6):1309. doi: 10.3390/biomedicines10061309 (PMC9219643; doi:10.3390/biomedicines10061309)
Supplement: Supplementary file 1 [file biomedicines-10-01309-s001.zip › Supplementary Table S1.pdf]

Supplementary Table S1. *Results of genome-wide differential expression analysis between CAD ar*
The list shows the statistics for all genes detected by RNA-Seq

CAD.vs.noCAD

Gene Symbol	logFC	AveExpr	t	P.Value
CHEK1	-0.40	5.00	-4.72	0.00002
PRKAR1B	0.49	8.13	4.56	0.00003
PTCD1	0.35	6.66	4.52	0.00004
DNAJC13	-0.29	8.87	-4.51	0.00004
RPL14P3	0.36	5.42	4.49	0.00004
THAP3	0.30	8.32	4.48	0.00004
USP33	-0.34	7.96	-4.46	0.00004
CCDC61	0.32	7.60	4.38	0.00006
ALDOC	0.34	9.00	4.36	0.00006
FAM174C	0.46	8.95	4.35	0.00006
C1GALT1	-0.45	7.56	-4.31	0.00007
TRAPPC11	-0.34	7.86	-4.31	0.00007
SH2B3	-0.32	10.16	-4.30	0.00007
IDH1	-0.34	8.14	-4.27	0.00008
GNB1L	0.35	6.71	4.25	0.00009
OXSR1	-0.28	8.83	-4.25	0.00009
C12orf43	0.35	8.16	4.23	0.00010
NOSIP	0.43	11.37	4.19	0.00011
C16orf74	0.45	7.54	4.18	0.00011
EXOC8	-0.39	7.43	-4.18	0.00011
PLA2G4A	-0.45	5.58	-4.16	0.00012
DMXL2	-0.54	8.60	-4.14	0.00013
GPD2	-0.39	6.84	-4.11	0.00014
MZT2A	0.41	8.43	4.09	0.00015
AL022718.1	0.75	8.71	4.09	0.00015
RITA1	0.39	8.18	4.08	0.00016
FRY	-0.47	9.06	-4.07	0.00016
PCYT2	0.33	8.79	4.05	0.00017
ECSIT	0.37	9.13	4.05	0.00017
MED16	0.33	10.80	4.04	0.00018
TMLHE	-0.35	6.40	-4.04	0.00018
GAMT	0.40	6.94	4.03	0.00018
EEF1D	0.37	13.58	3.99	0.00021
UBOX5	0.28	8.52	3.99	0.00021
ARFRP1	0.30	9.47	3.99	0.00021
MARCHF9	0.34	9.22	3.99	0.00021
RPS9	0.39	14.03	3.96	0.00023
EEF1DP2	0.40	7.68	3.96	0.00023
ARAP2	-0.43	7.75	-3.94	0.00024
ZFAND5	-0.28	10.07	-3.93	0.00026
PMAIP1	-0.36	6.11	-3.92	0.00027
HECTD1	-0.32	8.64	-3.91	0.00027
ETV3	-0.34	8.00	-3.91	0.00027
B3GAT3	0.29	9.30	3.90	0.00028
VPS51	0.34	11.37	3.90	0.00028

PATL1	-0.33	9.02	-3.89	0.00029
MRPL34	0.30	8.72	3.89	0.00029
TMUB1	0.38	9.15	3.88	0.00029
PQBP1	0.31	10.11	3.87	0.00031
SHPK	0.38	5.74	3.86	0.00032
GNL1	0.27	9.63	3.86	0.00032
LENG1	0.33	8.04	3.85	0.00032
FBXO34	-0.34	7.90	-3.85	0.00033
AL096829.1	0.33	5.84	3.84	0.00034
RNASEH2C	0.29	8.97	3.82	0.00036
ZEB2	-0.36	9.38	-3.82	0.00036
C9orf16	0.49	8.86	3.82	0.00036
NENF	0.40	8.70	3.81	0.00037
UTRN	-0.39	9.18	-3.81	0.00037
SETX	-0.26	10.90	-3.81	0.00037
ELOF1	0.41	9.67	3.80	0.00038
AP1G1	-0.32	9.25	-3.80	0.00039
GSE1	-0.29	9.15	-3.79	0.00040
DEAF1	0.24	9.29	3.78	0.00041
USP25	-0.32	8.28	-3.77	0.00042
KIAA1109	-0.46	8.72	-3.77	0.00042
NUDT16L1	0.30	8.86	3.76	0.00043
RAP2C	-0.31	8.32	-3.76	0.00043
UBP1	-0.26	9.06	-3.76	0.00044
HDHD3	0.34	7.53	3.76	0.00044
SIT1	0.42	9.59	3.76	0.00044
TRBJ2-6	0.37	5.01	3.75	0.00045
SETD2	-0.22	10.04	-3.74	0.00046
TLE5	0.34	13.35	3.73	0.00047
ALKBH7	0.44	8.99	3.73	0.00047
ATAD2B	-0.32	6.94	-3.72	0.00050
C12orf57	0.49	9.91	3.71	0.00050
SIKE1	-0.24	6.98	-3.69	0.00053
TSC22D2	-0.30	7.22	-3.69	0.00053
SESTD1	-0.33	6.91	-3.69	0.00054
DUSP6	-0.47	10.68	-3.69	0.00054
BIRC6	-0.30	9.37	-3.68	0.00056
LRRK2	-0.63	10.01	-3.67	0.00057
INF2	0.33	9.54	3.66	0.00059
TNFRSF10B	-0.34	9.11	-3.66	0.00060
GGT1	0.40	8.44	3.66	0.00060
MIS18BP1	-0.36	7.41	-3.65	0.00061
VEGFB	0.35	9.12	3.65	0.00061
ARL2	0.42	8.19	3.65	0.00061
TAF1	-0.37	8.57	-3.65	0.00061
ATAD3C	0.45	5.31	3.64	0.00063
ZNF428	0.37	8.29	3.64	0.00063
OSBPL11	-0.36	8.48	-3.64	0.00063
RPS21	0.51	10.48	3.63	0.00064
ELF2	-0.34	8.83	-3.63	0.00064

POLR1E	0.33	8.42	3.63	0.00065
ASMTL	0.24	9.72	3.63	0.00066
SH3GLB2	0.27	10.41	3.62	0.00067
GRAP	0.48	9.79	3.62	0.00068
L3MBTL3	-0.36	7.14	-3.61	0.00069
ZNF688	0.31	8.23	3.61	0.00069
NOP53	0.45	13.21	3.61	0.00070
ZDHHC20	-0.31	8.21	-3.61	0.00070
DOCK4	-0.53	6.13	-3.60	0.00071
PDE6B	0.28	7.19	3.59	0.00073
ITPRID2	-0.48	8.33	-3.59	0.00073
NDUFS8	0.39	9.48	3.59	0.00073
SPRY2	-0.39	5.08	-3.59	0.00075
THAP7	0.29	8.79	3.58	0.00075
LIPE-AS1	0.32	5.36	3.57	0.00078
STON1	-0.40	5.20	-3.57	0.00079
SPG11	-0.31	9.69	-3.56	0.00080
COX19	0.22	8.82	3.55	0.00082
CEPT1	-0.30	7.68	-3.55	0.00084
BIN1	0.34	11.10	3.55	0.00084
STUB1	0.31	10.74	3.54	0.00085
OXR1	-0.30	6.96	-3.54	0.00085
MEPCE	0.24	10.59	3.54	0.00085
HSPBP1	0.44	8.36	3.54	0.00086
MRPS34	0.37	9.64	3.54	0.00086
ATP10D	-0.38	6.62	-3.54	0.00086
NSUN5	0.21	9.51	3.53	0.00089
AC004241.2	0.41	5.03	3.53	0.00089
RPL13	0.45	15.12	3.52	0.00090
METTL26	0.42	9.16	3.52	0.00091
CD164	-0.28	10.48	-3.52	0.00091
UBR5	-0.30	9.26	-3.52	0.00092
ZSWIM6	-0.44	7.69	-3.51	0.00094
MACF1	-0.30	10.78	-3.51	0.00094
TMEM238	0.45	6.41	3.50	0.00096
SLC22A15	-0.58	6.65	-3.50	0.00096
SERINC1	-0.38	10.03	-3.50	0.00097
KBTBD2	-0.29	9.25	-3.50	0.00097
CHMP6	0.29	8.80	3.50	0.00098
ZCCHC2	-0.51	8.71	-3.50	0.00098
SPIN1	-0.32	7.33	-3.49	0.00098
ERBIN	-0.34	9.11	-3.49	0.00098
RP2	-0.43	8.08	-3.49	0.00099
USP34	-0.24	10.12	-3.49	0.00101
ZNRD2	0.30	8.00	3.48	0.00102
TRIR	0.30	11.81	3.48	0.00103
ZCCHC4	-0.33	5.78	-3.48	0.00103
TNFAIP8L1	0.29	8.53	3.48	0.00104
NMRAL1	0.33	9.24	3.47	0.00105
LMTK3	0.40	6.55	3.47	0.00105

CHMP5	-0.40	8.79	-3.47	0.00105
NDUFAF3	0.27	9.81	3.47	0.00106
SLFN11	-0.30	8.41	-3.46	0.00107
ANKFY1	-0.28	9.73	-3.46	0.00108
HACD4	-0.28	9.19	-3.46	0.00108
PDK4	-0.41	7.38	-3.46	0.00109
RPLP2	0.40	14.80	3.46	0.00109
PYCR2	0.20	9.61	3.46	0.00109
LTB	0.41	12.27	3.46	0.00109
CCNQ	0.24	8.59	3.46	0.00110
DCXR	0.36	9.64	3.46	0.00110
ATP6V1A	-0.32	9.28	-3.45	0.00112
ZNF524	0.39	8.49	3.45	0.00112
AC027097.2	0.29	6.95	3.45	0.00112
TMEM167A	-0.23	8.83	-3.45	0.00114
HIP1	-0.53	9.39	-3.45	0.00114
ATG9B	0.44	7.31	3.44	0.00114
TFPT	0.35	8.40	3.44	0.00114
GIGYF2	-0.24	9.19	-3.44	0.00115
RPL13P12	0.51	11.46	3.44	0.00116
MRC1	-0.43	5.13	-3.44	0.00117
TRBV15	0.48	5.97	3.44	0.00117
MCM4	-0.28	6.96	-3.44	0.00117
PDCD6IP	-0.26	9.79	-3.44	0.00117
ABITRAM	-0.25	6.75	-3.44	0.00117
MRPL43	0.26	9.55	3.43	0.00118
FAM153CP	0.46	6.53	3.43	0.00118
RPLP1	0.39	14.18	3.43	0.00119
CEP76	-0.27	5.51	-3.43	0.00119
DCP1A	0.32	9.58	3.43	0.00119
YJU2	0.29	8.67	3.43	0.00120
RPS25	0.33	13.53	3.43	0.00120
EXOC1	-0.30	8.09	-3.42	0.00123
FAR2	-0.45	7.18	-3.42	0.00124
SUSD3	0.30	9.67	3.42	0.00124
XPR1	-0.31	7.10	-3.41	0.00126
LIPA	-0.34	9.22	-3.41	0.00127
ZBTB20	0.56	7.56	3.41	0.00128
RC3H2	-0.24	8.12	-3.41	0.00128
SNRNP25	0.36	7.75	3.41	0.00129
RCBTB2	-0.30	8.52	-3.40	0.00129
RPRD1A	-0.30	7.59	-3.40	0.00131
LRRC37B	-0.26	7.45	-3.40	0.00132
PRKAB2	-0.37	6.54	-3.39	0.00133
LHPP	0.36	9.12	3.39	0.00133
PXN-AS1	0.30	5.47	3.39	0.00134
RPL29	0.40	13.79	3.39	0.00135
RPS14	0.40	13.57	3.39	0.00135
TMEM134	0.33	9.04	3.39	0.00135
RPL19	0.34	14.90	3.39	0.00135

MFSD14B	-0.43	8.90	-3.38	0.00137
RPS6KC1	-0.26	6.93	-3.38	0.00137
CCR2	-0.36	9.95	-3.38	0.00138
SNX6	-0.23	9.02	-3.38	0.00139
S1PR3	-0.37	6.84	-3.38	0.00139
BACH1	-0.41	9.39	-3.37	0.00141
RPS5	0.38	13.26	3.37	0.00143
ALKBH3	0.25	7.89	3.37	0.00143
CCDC106	0.38	6.76	3.37	0.00143
CCDC124	0.35	8.91	3.37	0.00144
MBD3	0.39	9.29	3.36	0.00146
GIPC1	0.38	8.85	3.36	0.00147
CLEC12A	-0.79	9.37	-3.36	0.00148
C9orf72	-0.44	7.88	-3.35	0.00149
YDJC	0.27	8.16	3.35	0.00150
CEP350	-0.34	8.34	-3.35	0.00150
ZNF777	0.25	8.95	3.35	0.00150
THAP11	0.19	9.82	3.35	0.00151
BICRAL	-0.29	8.89	-3.35	0.00151
NUBP2	0.28	9.16	3.35	0.00152
SNRPN	0.34	7.30	3.35	0.00153
SGF29	0.35	9.06	3.35	0.00153
USP15	-0.45	10.14	-3.35	0.00153
RPL23	0.31	13.92	3.35	0.00153
KCTD12	-0.36	9.57	-3.34	0.00155
SENP6	-0.27	8.69	-3.34	0.00155
TRAPPC6A	0.38	8.63	3.34	0.00155
OCRL	-0.30	5.99	-3.34	0.00156
RIN2	-0.40	7.73	-3.34	0.00156
GADD45GIP1	0.37	8.77	3.34	0.00157
RPL13AP20	0.38	7.07	3.34	0.00158
GTF3C5	0.20	9.87	3.33	0.00159
ZNF526	0.23	8.48	3.33	0.00159
SOX12	0.32	7.67	3.33	0.00160
APOBEC3B	-0.84	6.61	-3.33	0.00161
FGL2	-0.36	11.69	-3.33	0.00162
PDK2	0.26	9.03	3.33	0.00163
TMOD3	-0.23	8.96	-3.32	0.00165
RABAC1	0.40	7.55	3.32	0.00165
FAM120A	-0.26	10.32	-3.32	0.00166
TGFB1I1	0.45	6.16	3.32	0.00166
NSMCE1	0.32	10.02	3.31	0.00168
ZEB2-AS1	-0.30	5.01	-3.31	0.00170
PCF11	-0.34	9.20	-3.31	0.00173
MZT2B	0.46	9.06	3.30	0.00173
SHPRH	-0.33	6.32	-3.30	0.00173
DDX60L	-0.57	9.36	-3.30	0.00174
ZNF444	0.30	7.75	3.30	0.00174
RIC1	-0.35	7.74	-3.30	0.00174
SEPTIN9	0.27	13.62	3.30	0.00175

IVD	0.21	9.41	3.30	0.00175
FBL	0.36	11.62	3.30	0.00175
LYST	-0.42	10.04	-3.30	0.00176
RPL38	0.40	13.53	3.30	0.00177
RPS15AP17	0.39	5.81	3.29	0.00179
DDX5	-0.35	12.19	-3.29	0.00182
SLC52A2	0.28	8.33	3.29	0.00183
DICER1	-0.40	8.83	-3.29	0.00183
CPEB2	-0.37	6.80	-3.28	0.00184
FCGR3A	-0.41	12.06	-3.28	0.00184
RPL37A	0.35	14.23	3.28	0.00185
GGA1	0.21	10.17	3.28	0.00185
ERCC6	-0.23	7.44	-3.28	0.00185
CAVIN1	0.42	5.96	3.28	0.00187
BUD23	0.29	9.20	3.27	0.00189
MARCHF7	-0.36	9.05	-3.27	0.00189
VCPIP1	-0.34	8.49	-3.27	0.00190
JMJD1C	-0.41	9.41	-3.27	0.00190
PPDPF	0.50	11.87	3.27	0.00190
MAGIX	0.27	5.38	3.27	0.00191
TASOR2	-0.43	7.48	-3.27	0.00193
C12orf10	0.29	9.38	3.27	0.00193
RELCH	-0.37	7.54	-3.27	0.00194
NOC2LP2	0.33	5.05	3.27	0.00194
TSPAN2	-0.45	7.59	-3.26	0.00197
ARMT1	-0.28	7.32	-3.26	0.00198
FAM8A1	-0.43	8.83	-3.26	0.00199
P2RY14	-0.43	6.07	-3.26	0.00199
ATP5F1D	0.35	9.38	3.25	0.00200
GDAP2	-0.27	7.26	-3.25	0.00200
SH2D3A	0.32	8.10	3.25	0.00202
FXYD5	0.36	13.21	3.25	0.00203
FLVCR2	-0.29	7.15	-3.25	0.00205
PRDX2	0.36	10.38	3.25	0.00206
FAM198B-AS1	-0.42	5.19	-3.25	0.00206
RPS3AP47	0.57	7.52	3.24	0.00207
SLC25A6	0.33	13.64	3.24	0.00209
PARP8	-0.39	10.20	-3.24	0.00211
ATP8A1	-0.36	7.29	-3.23	0.00213
PRRC1	-0.27	7.56	-3.23	0.00213
CTNNA1	-0.26	10.28	-3.23	0.00216
RPL3	0.35	15.69	3.23	0.00216
HIPK3	-0.41	9.16	-3.23	0.00217
ZNF217	-0.45	9.55	-3.23	0.00217
PRKAR1A	-0.34	10.30	-3.23	0.00218
SYTL1	0.26	11.00	3.22	0.00223
SELENOM	0.44	7.64	3.22	0.00224
PTPN11	-0.36	8.00	-3.22	0.00224
NBPF10	-0.39	7.95	-3.22	0.00224
SPINDOC	0.28	7.74	3.21	0.00225

FNDC10	0.44	7.13	3.21	0.00225
SEPTIN1	0.32	10.72	3.21	0.00226
PRRT3	0.31	6.69	3.21	0.00227
PPTC7	-0.28	8.80	-3.21	0.00228
CPTP	0.41	8.40	3.21	0.00229
EVI5	-0.25	6.77	-3.21	0.00230
KDM1B	-0.43	7.76	-3.21	0.00230
ANAPC16	0.19	10.39	3.21	0.00230
PKN2	-0.44	7.81	-3.21	0.00231
RPL26P6	0.42	6.87	3.21	0.00231
STXBP3	-0.40	8.18	-3.20	0.00232
UCP2	0.32	13.12	3.20	0.00233
HELZ	-0.22	8.75	-3.20	0.00234
PRR7	0.32	6.49	3.20	0.00234
PURA	0.41	8.47	3.20	0.00234
RPS19	0.42	14.32	3.20	0.00235
NRIP1	-0.32	6.56	-3.20	0.00235
GCH1	-0.35	8.06	-3.20	0.00237
ARPC1B	0.43	8.82	3.20	0.00238
C20orf194	-0.25	7.15	-3.19	0.00238
AC107032.1	0.34	7.91	3.19	0.00239
EDF1	0.32	11.54	3.19	0.00239
RPL10P3	0.54	7.48	3.19	0.00240
MIA3	-0.21	9.63	-3.19	0.00240
WASHC4	-0.31	8.29	-3.19	0.00242
EIF3G	0.31	12.07	3.19	0.00242
SNAP47	0.27	8.52	3.19	0.00243
RPL18	0.39	14.32	3.19	0.00244
NLRP3	-0.31	9.05	-3.18	0.00246
TRIO	-0.32	8.20	-3.18	0.00247
RACK1	0.31	14.85	3.18	0.00247
MDH2	0.24	10.97	3.18	0.00248
MEMO1	0.41	5.96	3.18	0.00249
ARHGEF6	-0.21	9.90	-3.18	0.00250
CHCHD6	0.35	6.45	3.18	0.00250
MOSPD2	-0.42	8.00	-3.17	0.00253
ZNF224	-0.25	8.08	-3.17	0.00254
CD7	0.39	11.36	3.17	0.00256
FAM111A	-0.32	8.73	-3.17	0.00257
POLD2	0.30	9.25	3.17	0.00257
SVIP	0.31	8.64	3.17	0.00259
ACSL4	-0.48	8.55	-3.16	0.00261
RPS28P7	0.53	10.00	3.16	0.00261
BEND5	0.33	6.06	3.16	0.00261
ALKBH4	0.27	7.59	3.16	0.00261
AL390728.4	-0.46	7.00	-3.16	0.00261
RPL10A	0.31	13.51	3.16	0.00262
RNF19A	-0.38	8.07	-3.16	0.00262
AMD1	-0.29	9.52	-3.16	0.00262
TCOF1	0.26	10.49	3.16	0.00263

BTN3A3	-0.24	10.15	-3.16	0.00265
DOCK8	-0.37	11.48	-3.16	0.00266
SAMD9	-0.44	8.55	-3.16	0.00267
SNTA1	0.34	7.35	3.16	0.00267
RPS15	0.38	13.85	3.16	0.00267
HNRNPLL	-0.27	7.43	-3.16	0.00267
LMBRD2	-0.27	6.30	-3.15	0.00268
HERC1	-0.23	10.42	-3.15	0.00271
PYM1	0.26	8.92	3.15	0.00271
MRPS18B	0.25	9.70	3.15	0.00272
METTL16	0.25	8.81	3.15	0.00272
TMEM161B	-0.30	6.02	-3.15	0.00273
TSR3	0.27	9.07	3.15	0.00274
LYPLA1	-0.32	7.26	-3.15	0.00274
CD6	0.35	11.89	3.15	0.00274
RNF213	-0.39	12.74	-3.14	0.00275
SNX27	-0.27	9.64	-3.14	0.00276
SLC20A1	-0.23	9.89	-3.14	0.00276
FOPNL	-0.28	7.17	-3.14	0.00277
MAT2B	-0.23	10.27	-3.14	0.00277
RPL23AP3	0.22	5.32	3.14	0.00278
DPP7	0.35	10.70	3.14	0.00279
FBXL15	0.37	7.85	3.14	0.00280
AC027307.2	0.30	7.65	3.14	0.00282
DHX15	-0.25	9.08	-3.14	0.00282
ZNF358	0.40	7.24	3.13	0.00284
TRIQK	-0.37	6.68	-3.13	0.00284
IMPACT	-0.27	6.54	-3.13	0.00285
UBXN1	0.27	11.75	3.13	0.00285
MAN1B1	0.21	10.43	3.13	0.00285
PLAGL1	-0.48	7.51	-3.13	0.00286
NAA10	0.29	9.42	3.13	0.00286
PDGFC	-0.32	5.89	-3.13	0.00288
H1-10	0.29	11.15	3.13	0.00289
SEC24D	-0.37	7.95	-3.13	0.00289
CAPZA2	-0.35	9.13	-3.13	0.00290
CLPP	0.26	9.16	3.13	0.00290
ZNF837	0.31	5.41	3.13	0.00291
FAU	0.35	11.98	3.12	0.00293
CRTC1	0.22	8.95	3.12	0.00293
DIP2B	-0.32	9.06	-3.12	0.00293
ZNF91	0.39	7.69	3.12	0.00294
TIMM50	0.22	8.71	3.12	0.00294
CARD8	-0.33	10.45	-3.12	0.00295
SF3A2	0.28	10.85	3.12	0.00295
CYBB	-0.37	11.42	-3.12	0.00295
LHFPL2	-0.42	7.18	-3.12	0.00296
AKAP11	-0.31	7.39	-3.12	0.00297
RPL13P2	0.31	5.87	3.12	0.00297
GASK1B	-0.37	7.66	-3.12	0.00298

UHRF1BP1L	-0.33	7.50	-3.12	0.00298
PHLPP1	-0.32	7.57	-3.12	0.00298
ANGPTL6	0.31	5.44	3.12	0.00299
AC027644.3	0.27	6.59	3.12	0.00300
RCOR1	-0.35	9.02	-3.12	0.00300
PEX1	-0.29	6.88	-3.11	0.00303
ZNF71	0.31	7.63	3.11	0.00307
CD46	-0.37	10.77	-3.11	0.00307
PAPOLG	-0.31	7.48	-3.11	0.00308
SLC2A4RG	0.33	10.04	3.11	0.00308
XAB2	0.20	10.86	3.10	0.00308
OSGIN2	-0.33	7.79	-3.10	0.00309
NFATC1	0.22	9.74	3.10	0.00309
LYRM4	0.29	8.10	3.10	0.00310
LDLRAD3	-0.26	5.76	-3.10	0.00311
MCRIP1	0.39	9.65	3.10	0.00313
TLR8	-0.48	9.97	-3.10	0.00316
MRPL23	0.35	9.03	3.10	0.00316
RNPEPL1	0.25	12.27	3.10	0.00317
ZNF395	0.27	9.21	3.10	0.00317
N4BP2	-0.31	5.80	-3.09	0.00319
APC	-0.41	7.63	-3.09	0.00322
C8orf82	0.26	7.47	3.09	0.00324
DENND5A	-0.35	10.83	-3.09	0.00324
MFSD6	-0.32	7.73	-3.09	0.00324
GOLGA1	-0.23	8.30	-3.09	0.00324
APAF1	-0.30	9.02	-3.09	0.00326
RPUSD1	0.26	8.83	3.09	0.00326
NDUFA13	0.45	6.50	3.08	0.00327
KCNJ2	-0.56	8.02	-3.08	0.00327
EEF2	0.25	15.49	3.08	0.00328
YIF1B	0.32	9.49	3.08	0.00328
BCLAF1	-0.24	9.09	-3.08	0.00329
RPL14	0.32	13.84	3.08	0.00331
VAMP2	0.22	11.66	3.08	0.00331
RNF126	0.25	9.75	3.08	0.00331
SPCS1	0.20	9.41	3.08	0.00334
SMARCB1	0.25	10.10	3.07	0.00338
ADAM9	-0.40	6.31	-3.07	0.00342
NDUFS7	0.31	9.81	3.07	0.00342
IQGAP2	-0.37	8.95	-3.07	0.00344
DENND1C	0.21	11.40	3.07	0.00345
ZSCAN30	-0.28	5.90	-3.06	0.00345
FAM122B	-0.28	7.96	-3.06	0.00349
CCDC22	0.24	9.63	3.06	0.00351
FBXW5	0.22	11.23	3.06	0.00352
AC005392.2	-0.96	6.22	-3.06	0.00353
USE1	0.27	8.83	3.06	0.00355
SMCHD1	-0.43	10.43	-3.06	0.00355
MAF1	0.30	11.01	3.06	0.00355

GBE1	-0.34	7.08	-3.05	0.00355
TBCK	-0.26	6.83	-3.05	0.00357
MIB1	-0.26	7.09	-3.05	0.00358
AC018755.4	-0.69	6.78	-3.05	0.00360
UXT	0.30	9.78	3.05	0.00362
NEK4	-0.25	6.89	-3.05	0.00362
EXOSC4	0.28	7.09	3.05	0.00364
GALNT7	-0.29	6.92	-3.05	0.00364
ARFGEF1	-0.29	8.72	-3.05	0.00365
CTNNB1	-0.21	10.16	-3.05	0.00365
NUDT18	0.28	8.02	3.05	0.00365
AC142472.1	0.33	5.49	3.04	0.00366
OGFRL1	-0.32	10.13	-3.04	0.00366
LIPN	-0.39	5.47	-3.04	0.00366
HEG1	-0.35	7.54	-3.04	0.00367
KDM8	0.23	7.16	3.04	0.00367
TOR1AIP1	-0.30	9.14	-3.04	0.00367
FMR1	-0.29	7.80	-3.04	0.00368
LPCAT2	-0.45	8.43	-3.04	0.00369
CYP20A1	0.43	8.92	3.04	0.00369
SPAG7	0.23	9.22	3.04	0.00370
SNX10	-0.38	8.56	-3.04	0.00370
NUFIP2	-0.28	9.24	-3.04	0.00371
GALC	-0.27	7.96	-3.04	0.00371
POLR2L	0.36	9.47	3.04	0.00371
IFRD2	0.23	8.42	3.04	0.00372
PANK3	-0.24	7.50	-3.04	0.00374
TRBC2	0.41	13.31	3.04	0.00375
SENP3	0.23	7.35	3.04	0.00375
CCDC71	0.21	8.75	3.04	0.00375
CUTA	0.33	10.11	3.04	0.00376
CTDNEP1	0.24	11.01	3.03	0.00377
RAB3IP	0.28	7.20	3.03	0.00377
RLF	-0.22	7.80	-3.03	0.00379
CHUK	-0.32	7.35	-3.03	0.00379
TMED2	-0.22	9.29	-3.03	0.00380
RABL6	0.30	10.25	3.03	0.00383
RPS3	0.32	14.80	3.03	0.00384
PKN1	0.23	12.24	3.03	0.00385
PTGS2	-0.42	6.76	-3.03	0.00386
COMMD2	-0.21	7.67	-3.02	0.00389
DPYD	-0.39	9.03	-3.02	0.00389
BAD	0.33	8.34	3.02	0.00389
NME4	0.34	8.50	3.02	0.00390
DNAJB2	0.26	10.56	3.02	0.00393
Z97634.1	0.35	5.46	3.02	0.00393
C19orf48	0.31	8.03	3.02	0.00396
AP5S1	0.24	7.62	3.02	0.00397
TCF25	0.19	11.49	3.02	0.00397
BCR	0.19	10.83	3.01	0.00398

NELFE	0.24	9.94	3.01	0.00399
BZW1	-0.28	8.70	-3.01	0.00400
SNHG8	0.42	8.21	3.01	0.00400
COX4I1	0.29	12.78	3.01	0.00401
BCL7C	0.35	8.89	3.01	0.00401
ZCRB1	0.34	8.09	3.01	0.00402
ZNF517	0.28	7.04	3.01	0.00405
GPR55	0.32	7.10	3.01	0.00406
MYO5A	-0.24	9.05	-3.01	0.00406
RPL36	0.34	12.22	3.01	0.00406
RASA1	-0.29	7.39	-3.01	0.00406
SLFN5	-0.36	9.78	-3.01	0.00407
TEP1	-0.29	9.65	-3.01	0.00407
B3GALT6	0.23	8.53	3.01	0.00407
C6orf120	-0.30	6.78	-3.01	0.00407
SERGEF	0.29	7.94	3.00	0.00411
TRBC1	0.36	12.36	3.00	0.00411
HCFC1R1	0.33	8.64	3.00	0.00412
SRM	0.28	9.73	3.00	0.00412
CDK17	-0.37	7.21	-3.00	0.00413
DTX3L	-0.36	9.71	-3.00	0.00414
AC008555.4	0.31	7.91	3.00	0.00415
RPL37P2	0.42	9.42	3.00	0.00416
AC009053.2	-0.31	5.99	-3.00	0.00416
FBXW4	0.23	9.78	3.00	0.00416
NNT	-0.29	8.26	-3.00	0.00417
USF3	-0.30	7.81	-3.00	0.00417
FSD1L	-0.34	4.99	-3.00	0.00419
TP53BP2	-0.23	8.54	-3.00	0.00419
UBR1	-0.27	7.50	-3.00	0.00419
GCHFR	0.34	6.96	2.99	0.00420
DDX3X	-0.40	10.70	-2.99	0.00421
ACBD5	-0.29	7.08	-2.99	0.00422
UROS	0.23	8.68	2.99	0.00425
UBTF	0.16	11.58	2.99	0.00426
TNFSF12	0.28	9.42	2.99	0.00429
SNRPA	0.28	9.57	2.99	0.00429
ZBTB14	-0.24	7.96	-2.99	0.00429
GNB4	-0.29	7.90	-2.99	0.00432
ABHD14B	0.26	9.94	2.99	0.00432
KIDINS220	-0.40	9.11	-2.98	0.00433
CCNT1	-0.22	7.83	-2.98	0.00434
ABCA1	-0.51	7.02	-2.98	0.00436
RPL36AL	0.30	11.43	2.98	0.00437
FN3K	0.35	6.40	2.98	0.00438
ZNF581	0.28	9.08	2.98	0.00439
ARMC6	0.21	8.73	2.98	0.00439
AL078595.1	0.51	5.69	2.98	0.00439
LRRC29	0.25	6.08	2.98	0.00441
ZNF784	0.23	7.50	2.98	0.00441

PGM3	-0.25	6.43	-2.98	0.00443
FAHD1	0.30	7.66	2.97	0.00445
GYS1	0.16	9.53	2.97	0.00446
ZBTB7A	0.31	10.94	2.97	0.00447
MFNG	0.22	11.62	2.97	0.00448
AC136475.5	-0.63	6.27	-2.97	0.00451
DBP	0.27	8.94	2.97	0.00452
TBC1D10C	0.24	11.76	2.97	0.00452
TEX264	0.26	9.77	2.97	0.00453
MTMR4	-0.24	8.67	-2.97	0.00454
TET2	-0.45	8.68	-2.97	0.00457
CCDC120	0.25	6.65	2.96	0.00458
ELF1	-0.39	9.64	-2.96	0.00466
TMOD2	-0.35	7.46	-2.96	0.00466
DMAC2L	0.30	7.32	2.96	0.00466
VCAN	-0.39	11.81	-2.96	0.00467
CORO1C	-0.24	10.20	-2.96	0.00468
POLR2I	0.29	9.00	2.96	0.00468
FBXW4P1	0.27	5.54	2.95	0.00471
TESK2	-0.26	7.25	-2.95	0.00471
SPTLC1	-0.21	8.32	-2.95	0.00471
FAS	-0.40	7.88	-2.95	0.00474
GSS	0.22	9.12	2.95	0.00478
TBC1D8	-0.36	8.43	-2.95	0.00480
PGPEP1	0.23	9.56	2.95	0.00482
CLEC1A	-0.32	5.29	-2.95	0.00483
RAB14	-0.20	9.48	-2.94	0.00483
XRCC1	0.20	9.50	2.94	0.00485
PAPOLA	-0.23	10.20	-2.94	0.00485
SNX30	-0.26	8.04	-2.94	0.00486
MRPL24	0.31	8.62	2.94	0.00486
CIZ1	0.18	10.96	2.94	0.00486
EPC1	-0.16	8.96	-2.94	0.00488
ZNF324	0.19	7.54	2.94	0.00489
POLR2E	0.27	10.94	2.94	0.00491
NUDT21	-0.18	8.80	-2.94	0.00492
CD27	0.41	10.44	2.94	0.00492
ZBTB11	-0.33	7.18	-2.94	0.00492
AL049597.1	0.25	4.97	2.94	0.00493
TOPBP1	-0.24	8.18	-2.94	0.00493
TACO1	0.23	7.92	2.94	0.00494
BORCS6	0.26	9.01	2.93	0.00496
HSPB1	0.38	10.07	2.93	0.00500
SCAF1	0.25	9.92	2.93	0.00500
SLC36A4	-0.24	6.48	-2.93	0.00501
ADSS2	-0.24	8.02	-2.93	0.00503
LINC00654	-0.33	5.17	-2.93	0.00504
RPL21P7	0.25	4.97	2.93	0.00504
AL031727.1	0.37	9.19	2.93	0.00504
ZBTB38	-0.36	7.79	-2.93	0.00510

SF3B1	-0.34	11.32	-2.92	0.00511
NHP2	0.28	9.12	2.92	0.00512
NCSTN	-0.19	10.80	-2.92	0.00512
DMTF1	-0.37	7.92	-2.92	0.00512
LPGAT1	-0.33	9.20	-2.92	0.00513
PICALM	-0.32	11.10	-2.92	0.00514
SLK	-0.27	8.83	-2.92	0.00515
SPOCK2	0.26	13.16	2.92	0.00516
MRPS18A	0.26	8.55	2.92	0.00518
FAM3A	0.23	8.96	2.92	0.00519
WDR44	-0.31	6.97	-2.92	0.00522
ACSF3	0.21	9.51	2.92	0.00523
QKI	-0.34	9.10	-2.92	0.00524
SURF1	0.27	9.52	2.92	0.00524
ANKRD13A	-0.27	10.53	-2.92	0.00524
NTMT1	0.27	8.34	2.92	0.00524
AP000580.1	0.46	5.27	2.91	0.00527
TLR4	-0.49	10.17	-2.91	0.00528
ANP32B	0.24	11.22	2.91	0.00529
PRMT1	0.25	10.31	2.91	0.00530
RPL8	0.33	14.62	2.91	0.00533
HACE1	-0.30	5.44	-2.91	0.00535
THAP9-AS1	-0.29	7.29	-2.91	0.00538
AC008850.2	0.29	5.16	2.90	0.00540
CTSS	-0.26	13.84	-2.90	0.00541
RPL28	0.35	14.73	2.90	0.00541
PEBP1	0.30	10.67	2.90	0.00542
SINHCAF	-0.33	6.99	-2.90	0.00544
CLK4	-0.36	7.70	-2.90	0.00545
OVCH1-AS1	-0.36	5.32	-2.90	0.00546
PRDX3	-0.24	9.02	-2.90	0.00546
LYPLA2	0.25	10.05	2.90	0.00546
TSHZ2	0.54	7.27	2.90	0.00547
MED23	-0.37	7.73	-2.90	0.00547
RPS29	0.48	11.67	2.90	0.00547
MAP2K5	0.21	8.13	2.90	0.00552
C19orf53	0.31	10.44	2.90	0.00553
ZXDC	-0.23	9.26	-2.90	0.00553
PELI1	-0.45	9.19	-2.89	0.00558
Z84492.1	0.28	6.09	2.89	0.00560
ANGPT1	-0.32	5.05	-2.89	0.00560
Z93930.2	0.31	5.12	2.89	0.00560
TXN2	0.29	9.71	2.89	0.00561
IGFBP4	0.35	6.91	2.89	0.00561
CD209	-0.36	5.26	-2.89	0.00561
GTF2F1	0.21	10.77	2.89	0.00562
SMARCA5	-0.21	9.09	-2.89	0.00563
NBDY	0.27	8.08	2.89	0.00564
ZNF714	0.36	6.25	2.89	0.00566
ABHD3	-0.38	8.64	-2.89	0.00566

MCRIP2	0.30	7.21	2.89	0.00568
OTUD4	-0.31	7.40	-2.89	0.00568
SETD7	-0.26	7.56	-2.88	0.00573
EPS15	-0.26	8.63	-2.88	0.00573
MICB	-0.29	8.38	-2.88	0.00574
QTRT2	-0.23	7.57	-2.88	0.00576
C19orf25	0.23	9.00	2.88	0.00580
PSMA7	0.25	10.97	2.88	0.00580
TM7SF2	0.31	7.18	2.88	0.00583
ZNF117	-0.46	7.04	-2.88	0.00584
RPS23	0.30	12.63	2.88	0.00584
CLMN	-0.25	7.56	-2.88	0.00584
AL353705.1	0.27	5.57	2.87	0.00585
DDX58	-0.48	8.87	-2.87	0.00586
ZNF677	-0.30	5.61	-2.87	0.00587
APBB1	0.31	9.68	2.87	0.00587
IMP3	0.24	9.38	2.87	0.00588
AC139495.2	-0.73	7.57	-2.87	0.00588
LIMD2	0.23	13.27	2.87	0.00589
KIF16B	-0.37	6.32	-2.87	0.00590
AL136380.1	0.34	5.24	2.87	0.00591
HPS5	-0.27	7.57	-2.87	0.00591
RPL23A	0.31	13.26	2.87	0.00593
RRP1	0.27	8.15	2.87	0.00594
AC127521.1	0.26	6.53	2.87	0.00594
PUSL1	0.29	6.92	2.87	0.00596
PIK3AP1	-0.44	9.96	-2.87	0.00597
BEX5	0.26	5.55	2.87	0.00597
RPL27	0.34	12.53	2.87	0.00598
CRYBG1	-0.19	9.52	-2.87	0.00598
UBL7	0.28	10.71	2.87	0.00600
USP5	0.21	10.16	2.87	0.00600
AGFG2	0.25	8.93	2.87	0.00601
C5orf22	-0.23	6.61	-2.86	0.00603
HAX1	0.22	9.79	2.86	0.00604
AL355488.1	-0.31	5.49	-2.86	0.00605
ATP11A	-0.44	9.09	-2.86	0.00605
RAPGEF2	-0.44	8.36	-2.86	0.00606
MAP10	-0.35	5.04	-2.86	0.00606
PLCXD2	-0.30	6.09	-2.86	0.00607
MED28	-0.16	9.71	-2.86	0.00609
NACA4P	0.31	6.92	2.86	0.00609
ORMDL3	0.26	10.90	2.86	0.00609
RPS2P35	0.38	6.93	2.86	0.00611
FAM234B	-0.26	6.13	-2.86	0.00612
S1PR4	0.32	11.93	2.86	0.00613
ARAF	0.18	11.04	2.86	0.00614
AL359265.2	0.51	5.70	2.86	0.00614
IGSF8	0.25	9.15	2.86	0.00614
ADAM10	-0.27	9.78	-2.86	0.00614

L3MBTL2	0.19	9.78	2.86	0.00615
UNC45A	0.18	10.29	2.86	0.00615
WDR11	-0.30	8.64	-2.86	0.00615
KDM7A	-0.27	8.95	-2.86	0.00616
WDR74	0.23	8.88	2.86	0.00616
OST4	0.27	10.96	2.85	0.00620
CCDC12	0.29	9.71	2.85	0.00621
COMMD8	-0.25	7.36	-2.85	0.00625
ZNF641	-0.30	9.39	-2.85	0.00625
PRPF31	0.22	10.04	2.85	0.00626
ODC1-DT	-0.28	5.46	-2.85	0.00626
IFIT2	-0.73	10.36	-2.85	0.00628
OXLD1	0.24	8.52	2.85	0.00628
STAG2	-0.30	9.46	-2.85	0.00629
XRN1	-0.35	8.21	-2.85	0.00630
MRPL4	0.29	8.96	2.85	0.00630
ZNF593	0.41	7.14	2.85	0.00632
PAN3	-0.28	8.72	-2.85	0.00634
ZBTB21	-0.23	6.64	-2.84	0.00636
KMT2C	-0.34	9.50	-2.84	0.00638
PLEK	-0.25	11.74	-2.84	0.00640
VPS13C	-0.29	8.79	-2.84	0.00640
EIF2AK3	-0.29	7.05	-2.84	0.00640
SH3BP1	0.35	9.06	2.84	0.00641
ST3GAL6	-0.27	7.16	-2.84	0.00642
CLEC5A	-0.41	6.03	-2.84	0.00643
AP001062.1	0.28	6.38	2.84	0.00643
TTLL12	0.17	9.22	2.84	0.00644
NDUFV3	0.22	8.71	2.84	0.00645
ATP13A3	-0.33	7.32	-2.84	0.00646
ACPP	-0.26	7.23	-2.84	0.00646
RBM42	0.26	10.46	2.84	0.00647
CAMTA1	0.23	7.55	2.84	0.00651
CTBP2	-0.29	9.43	-2.84	0.00651
PTGER4	-0.26	8.81	-2.84	0.00651
COG6	-0.27	6.40	-2.84	0.00652
TMEM223	0.26	7.79	2.83	0.00652
CIAO3	0.22	8.75	2.83	0.00653
AL109809.1	-0.40	5.33	-2.83	0.00654
TRIM5	-0.37	7.33	-2.83	0.00654
SPOPL	-0.31	7.96	-2.83	0.00655
ATR	-0.27	7.58	-2.83	0.00655
PARP9	-0.50	9.74	-2.83	0.00658
SIRT6	0.25	8.55	2.83	0.00659
NEK9	-0.18	9.63	-2.83	0.00659
RPL35	0.35	13.40	2.83	0.00659
NIPSNAP1	0.15	8.72	2.83	0.00659
MCMBP	-0.19	9.49	-2.83	0.00660
LATS2	-0.36	7.07	-2.83	0.00660
RPLP1P6	0.28	5.27	2.83	0.00661

RPS15AP1	0.36	8.60	2.83	0.00661
C18orf25	-0.26	7.80	-2.83	0.00661
PWWP2A	-0.26	7.59	-2.83	0.00661
FNDC3A	-0.34	7.45	-2.83	0.00662
ADCY7	-0.32	10.68	-2.83	0.00662
IFIT5	-0.42	7.98	-2.83	0.00663
AP006621.3	0.37	6.33	2.83	0.00665
ZNF493	-0.30	6.39	-2.83	0.00665
EPHB6	0.24	9.58	2.83	0.00668
HAUS8	0.26	7.66	2.83	0.00668
SH3RF1	-0.29	5.37	-2.82	0.00671
KIF13A	-0.31	9.04	-2.82	0.00672
EIF2AK2	-0.47	8.47	-2.82	0.00672
BICD2	-0.29	9.72	-2.82	0.00675
TRAPPC10	-0.35	8.49	-2.82	0.00676
SHTN1	-0.38	7.29	-2.82	0.00676
GZMM	0.35	9.64	2.82	0.00677
WAC-AS1	0.25	8.92	2.82	0.00678
AC022210.1	0.25	6.30	2.82	0.00680
NCKIPSD	0.21	8.19	2.82	0.00681
FBXO38	-0.27	8.81	-2.82	0.00681
TANK	-0.30	8.61	-2.82	0.00682
CSNK1G1	-0.21	7.32	-2.82	0.00685
RNF114	-0.15	10.11	-2.82	0.00686
PRKCSH	0.21	12.65	2.82	0.00688
TOR1B	-0.31	9.04	-2.82	0.00688
DDA1	0.16	9.32	2.81	0.00689
FNIP1	-0.40	7.77	-2.81	0.00689
UBL4A	0.17	9.75	2.81	0.00689
CSNK2B	0.26	8.03	2.81	0.00690
ATP2A3	0.19	12.87	2.81	0.00690
FAM76B	-0.28	7.06	-2.81	0.00691
DAXX	0.17	10.42	2.81	0.00691
MAZ	0.19	11.35	2.81	0.00692
NIPSNAP2	-0.23	7.62	-2.81	0.00700
ATAD3A	0.25	7.59	2.81	0.00700
SHF	0.31	5.20	2.81	0.00701
PHLDB3	0.32	6.49	2.81	0.00701
LINC00294	-0.24	7.25	-2.81	0.00703
RPL18A	0.35	14.23	2.81	0.00705
NDUFB11	0.34	10.55	2.81	0.00705
FLYWCH1	0.35	9.34	2.81	0.00706
DGCR6	0.28	5.66	2.81	0.00706
RPL31P35	0.31	5.28	2.81	0.00707
METTL4	-0.24	6.79	-2.80	0.00707
CENPJ	-0.29	5.74	-2.80	0.00708
SKIL	-0.28	6.86	-2.80	0.00709
ATG2B	-0.30	8.11	-2.80	0.00712
SUCO	-0.22	7.21	-2.80	0.00714
ARFGEF2	-0.32	7.33	-2.80	0.00715

RPL24	0.29	12.12	2.80	0.00716
PPIP5K2	-0.29	7.54	-2.80	0.00719
MRPL57	0.26	8.36	2.80	0.00720
RC3H1	-0.30	8.28	-2.80	0.00720
BRWD3	-0.30	7.77	-2.80	0.00721
MTHFSD	0.17	8.17	2.80	0.00721
CES2	0.20	9.70	2.80	0.00722
PIGQ	0.25	8.52	2.80	0.00723
RPL37	0.28	14.12	2.79	0.00731
CFDP1	0.21	8.55	2.79	0.00732
DMAC2	0.19	9.00	2.79	0.00733
RPL13A	0.30	15.47	2.79	0.00735
ATP6V0E2	0.31	9.37	2.79	0.00738
RFXANK	0.27	10.36	2.79	0.00740
NDUFV1	0.26	10.81	2.79	0.00741
LTN1	-0.32	6.47	-2.79	0.00741
SIGIRR	0.28	11.10	2.79	0.00741
CX3CR1	-0.32	11.49	-2.79	0.00742
EMSY	-0.28	7.72	-2.79	0.00743
PARP14	-0.50	10.47	-2.79	0.00743
HMGA1	0.27	10.67	2.79	0.00743
SPINT2	0.22	9.39	2.78	0.00748
USP24	-0.17	9.74	-2.78	0.00749
MR1	-0.18	9.14	-2.78	0.00751
CNOT6	-0.20	7.81	-2.78	0.00751
LAMP2	-0.34	10.59	-2.78	0.00753
TOX	-0.31	7.24	-2.78	0.00754
HIPK1	-0.35	9.87	-2.78	0.00755
ATXN1L	-0.21	8.79	-2.78	0.00756
ACP5	0.31	9.24	2.78	0.00760
NCOA3	-0.29	8.77	-2.78	0.00762
AFF1	-0.39	8.89	-2.78	0.00762
TMEM41A	0.23	7.76	2.78	0.00763
AC024568.1	0.39	5.05	2.78	0.00764
ILF3-DT	0.31	8.77	2.78	0.00766
RPS11	0.32	15.34	2.77	0.00767
MGAT4B	0.19	10.80	2.77	0.00769
ARMCX1	-0.34	5.17	-2.77	0.00769
CLEC7A	-0.41	9.78	-2.77	0.00770
DNASE2	0.23	9.77	2.77	0.00770
CBL	-0.32	10.08	-2.77	0.00771
RBMX2	0.27	8.91	2.77	0.00772
KPNA4	-0.20	9.01	-2.77	0.00773
TMEM33	-0.22	8.79	-2.77	0.00774
NRAS	-0.25	7.92	-2.77	0.00774
JAK1	-0.22	11.86	-2.77	0.00775
MEN1	0.16	9.59	2.77	0.00778
THAP4	0.26	9.32	2.77	0.00784
ZBTB2	-0.20	8.15	-2.76	0.00789
CCDC137	0.23	8.96	2.76	0.00790

KLHL12	-0.23	8.28	-2.76	0.00790
TRIM22	-0.51	10.41	-2.76	0.00791
CUEDC2	0.25	9.50	2.76	0.00794
PAFAH1B3	0.32	8.31	2.76	0.00794
GRWD1	0.27	9.10	2.76	0.00795
GNAI3	-0.20	9.86	-2.76	0.00795
SLC31A1	-0.24	8.18	-2.76	0.00796
CSKMT	0.28	6.32	2.76	0.00796
BTBD2	0.19	10.64	2.76	0.00797
AC010680.2	-0.33	5.56	-2.76	0.00797
RPS6KB2	0.19	10.39	2.76	0.00798
EIF4G3	-0.31	8.80	-2.76	0.00798
NUP205	-0.27	8.19	-2.76	0.00800
RNU4-62P	0.53	7.13	2.76	0.00800
AC015819.1	0.25	6.80	2.76	0.00802
RP9	0.21	7.21	2.76	0.00802
AHNAK	-0.25	13.62	-2.76	0.00804
SENP1	-0.22	7.26	-2.76	0.00806
AC114271.1	0.24	5.99	2.76	0.00806
PCSK1N	0.50	5.65	2.76	0.00806
CXorf38	-0.17	9.22	-2.75	0.00811
CBLL1	-0.22	8.27	-2.75	0.00811
EEF1B2	0.28	12.35	2.75	0.00815
H2BC18	-0.40	5.22	-2.75	0.00818
AC009283.1	-0.20	6.67	-2.75	0.00823
PLXDC2	-0.34	9.60	-2.75	0.00826
PIK3CG	-0.25	9.32	-2.75	0.00827
TMEM156	0.30	7.37	2.75	0.00827
TRAPPC6B	-0.19	7.38	-2.75	0.00827
GAB1	-0.32	6.58	-2.75	0.00827
MICOS13	0.33	8.16	2.75	0.00830
TP53INP1	-0.36	8.90	-2.74	0.00831
AL136454.1	0.35	6.64	2.74	0.00831
REEP6	0.28	6.30	2.74	0.00834
SLC25A40	-0.27	6.84	-2.74	0.00835
MAL	0.45	9.67	2.74	0.00835
CCNT2	-0.30	7.74	-2.74	0.00837
U2SURP	-0.17	8.76	-2.74	0.00837
TNKS	-0.21	7.88	-2.74	0.00838
ECI1	0.28	8.16	2.74	0.00839
HMOX2	0.23	10.60	2.74	0.00840
NCAPH2	0.22	9.24	2.74	0.00840
FGD5-AS1	-0.26	9.20	-2.74	0.00841
RLIM	-0.22	8.26	-2.74	0.00841
WEE2-AS1	0.35	5.44	2.74	0.00841
FMNL2	-0.35	5.19	-2.74	0.00841
RPL32	0.29	14.20	2.74	0.00844
AC111170.1	0.42	5.16	2.74	0.00844
GAREM2	0.31	6.16	2.74	0.00845
SNRK	-0.33	9.58	-2.74	0.00850

IFITM9P	-0.40	5.22	-2.74	0.00850
GOLGA5	-0.21	7.72	-2.74	0.00851
FCHO2	-0.39	6.81	-2.73	0.00853
RPS2	0.32	15.01	2.73	0.00853
EIF3K	0.27	11.78	2.73	0.00854
AC090114.1	0.35	6.62	2.73	0.00854
BAZ2A	-0.25	11.33	-2.73	0.00856
SHMT2	0.23	10.11	2.73	0.00857
SELENOF	-0.18	9.57	-2.73	0.00858
SAT1	-0.34	11.88	-2.73	0.00858
MYD88	-0.27	11.54	-2.73	0.00858
FAM160B1	-0.31	7.81	-2.73	0.00858
INSIG1	-0.23	8.67	-2.73	0.00859
CCDC85B	0.43	8.11	2.73	0.00861
CBX6	0.17	10.18	2.73	0.00862
PALLD	-0.30	6.16	-2.73	0.00863
BOLA1	0.26	7.11	2.73	0.00863
KCTD18	-0.18	8.02	-2.73	0.00864
ANKRD39	0.22	6.04	2.73	0.00865
WDFY1	-0.29	7.14	-2.73	0.00867
PHB	0.24	10.16	2.73	0.00867
FAM91A1	-0.26	8.00	-2.73	0.00867
AC133134.1	0.35	6.04	2.73	0.00868
HSD11B1L	0.30	5.43	2.73	0.00871
IPO8	-0.20	8.23	-2.73	0.00872
AAAS	0.20	9.26	2.73	0.00873
ZDHHC6	-0.17	8.29	-2.73	0.00873
CYP2D6	-0.43	5.86	-2.73	0.00873
RBBP4	-0.15	9.67	-2.73	0.00874
LONRF1	-0.26	6.72	-2.73	0.00874
SAP130	-0.16	9.01	-2.73	0.00874
NOTCH2	-0.41	10.85	-2.72	0.00876
EYA3	-0.16	8.31	-2.72	0.00878
M6PR	-0.19	10.10	-2.72	0.00879
MAP3K2	-0.28	9.05	-2.72	0.00880
PHACTR2	-0.24	7.15	-2.72	0.00880
ERGIC3	0.24	11.51	2.72	0.00882
DRAIC	-0.30	5.48	-2.72	0.00885
TRPT1	0.21	8.20	2.72	0.00885
HPS3	-0.22	7.93	-2.72	0.00887
ZNF24	-0.14	9.16	-2.72	0.00889
CGGBP1	-0.17	9.33	-2.72	0.00889
WDR60	0.39	8.53	2.72	0.00892
RUVBL2	0.24	9.58	2.72	0.00893
PAPSS2	-0.29	5.86	-2.72	0.00894
BCAT2	0.25	8.38	2.72	0.00894
PSMD8	0.23	10.67	2.72	0.00895
MRPS26	0.20	8.38	2.72	0.00896
EMC10	0.24	10.39	2.72	0.00897
CD47	-0.19	9.83	-2.72	0.00897

CALM3	0.19	12.95	2.72	0.00898
AC087343.1	0.36	7.18	2.71	0.00902
MAP3K5	-0.30	9.27	-2.71	0.00902
ECPAS	-0.16	9.33	-2.71	0.00903
TMEM106B	-0.27	6.93	-2.71	0.00905
TIPRL	-0.23	7.44	-2.71	0.00906
NBPF3	-0.35	6.22	-2.71	0.00906
ZDHHC17	-0.34	7.39	-2.71	0.00907
SIGLEC14	-0.93	9.39	-2.71	0.00908
CSGALNACT2	-0.42	8.27	-2.71	0.00909
RPS28	0.37	12.96	2.71	0.00911
C1orf56	0.23	5.96	2.71	0.00913
TOMM40	0.24	8.77	2.71	0.00915
ZNF236	-0.31	6.86	-2.71	0.00915
HRAS	0.26	7.74	2.71	0.00918
MAPK1	-0.26	11.01	-2.71	0.00920
CRIP2	0.56	8.21	2.70	0.00923
KRI1	0.24	10.15	2.70	0.00926
PJA2	-0.29	9.27	-2.70	0.00926
CENPB	0.26	10.24	2.70	0.00927
FBXL5	-0.32	10.78	-2.70	0.00928
ERI3	0.21	8.11	2.70	0.00936
ITCH	-0.30	8.61	-2.70	0.00938
TASOR	-0.20	8.84	-2.70	0.00940
SH3GL1	0.23	9.98	2.70	0.00944
RHOF	0.21	10.81	2.70	0.00946
PHB2	0.22	11.52	2.69	0.00948
DCTPP1	0.26	8.20	2.69	0.00949
NUP153	-0.33	8.78	-2.69	0.00950
RPL21P93	0.28	8.38	2.69	0.00952
UBR2	-0.31	10.15	-2.69	0.00952
RPL10	0.31	15.55	2.69	0.00953
SP3	-0.27	9.13	-2.69	0.00955
MCFD2	-0.23	8.00	-2.69	0.00957
NBEAL1	0.32	5.27	2.69	0.00958
CBWD4P	-0.27	5.19	-2.69	0.00963
SPAST	-0.27	7.17	-2.69	0.00968
PRPF39	-0.26	6.62	-2.69	0.00968
PRPF4B	-0.21	8.96	-2.69	0.00971
JUND	0.24	11.24	2.69	0.00972
MORC2	0.18	9.73	2.69	0.00972
STRN3	-0.33	6.42	-2.68	0.00973
BTBD1	-0.21	8.01	-2.68	0.00975
SNHG6	0.25	10.47	2.68	0.00975
PARG	-0.20	7.43	-2.68	0.00976
PARP4	-0.26	9.95	-2.68	0.00976
ADCK2	0.23	8.51	2.68	0.00977
AC109446.3	0.34	4.98	2.68	0.00979
RPS19P3	0.40	9.15	2.68	0.00981
RPS10	0.36	6.78	2.68	0.00982

USP20	0.16	10.71	2.68	0.00982
PLAGL2	-0.22	9.36	-2.68	0.00984
APBA3	0.21	8.58	2.68	0.00988
PIKFYVE	-0.34	7.62	-2.68	0.00990
SLC15A4	-0.29	9.96	-2.68	0.00991
EBLN3P	-0.29	8.17	-2.68	0.00995
RPS6	0.26	14.51	2.68	0.00996
VPS13B	-0.30	8.37	-2.68	0.00996
TMEM123	-0.32	10.27	-2.68	0.00996
FAM98C	0.24	8.19	2.67	0.00999
SMG1	-0.29	9.28	-2.67	0.00999
WDR13	0.21	9.79	2.67	0.00999
GTF2A1	-0.22	7.80	-2.67	0.00999
SLC10A3	0.17	9.15	2.67	0.01000
ARHGEF18	0.18	10.97	2.67	0.01001
SLC25A1	0.30	9.38	2.67	0.01001
OSBPL1A	-0.30	6.17	-2.67	0.01003
HGSNAT	-0.16	9.83	-2.67	0.01003
NARS1	-0.17	9.36	-2.67	0.01004
RPL4	0.26	14.76	2.67	0.01006
AC134407.2	0.42	5.52	2.67	0.01009
CHURC1	-0.34	9.12	-2.67	0.01009
USF2	0.16	11.62	2.67	0.01011
MED13	-0.25	8.20	-2.67	0.01011
NDUFA3	0.33	9.48	2.67	0.01014
SP1	-0.23	10.59	-2.67	0.01015
RPL26	0.36	10.31	2.67	0.01015
NAPSB	0.48	10.58	2.67	0.01017
PNPLA8	-0.24	7.77	-2.67	0.01020
RAB11B	0.22	11.26	2.67	0.01021
DDX54	0.20	10.12	2.67	0.01021
JKAMP	-0.19	7.91	-2.67	0.01021
WDR18	0.33	8.51	2.67	0.01022
ANKRD50	-0.27	5.70	-2.67	0.01023
PDLIM1	0.29	9.43	2.66	0.01025
AC048382.5	0.26	5.68	2.66	0.01025
ZNF518A	-0.30	6.94	-2.66	0.01026
CD79B	0.43	9.97	2.66	0.01028
ELAC1	0.24	5.97	2.66	0.01029
CIB1	0.26	10.66	2.66	0.01030
CHD4	0.28	8.99	2.66	0.01030
DUS1L	0.20	10.30	2.66	0.01032
LINC00852	0.22	6.06	2.66	0.01032
SNRPB	0.23	10.47	2.66	0.01032
AL031985.2	0.24	5.17	2.66	0.01033
VPS28	0.25	11.31	2.66	0.01033
EVA1C	0.28	7.06	2.66	0.01035
RPSAP17	0.22	5.55	2.66	0.01035
ACTR2	-0.25	11.41	-2.66	0.01036
HADHB	-0.16	9.81	-2.66	0.01036

AC046185.3	0.23	8.89	2.66	0.01040
CLASP2	-0.27	7.43	-2.66	0.01041
TBL3	0.23	9.13	2.66	0.01041
SARS1	0.17	11.19	2.66	0.01043
EDEM3	-0.33	7.36	-2.66	0.01043
ZNHIT1	0.25	9.86	2.66	0.01043
AIP	0.22	10.98	2.66	0.01044
ATP2A2	-0.23	9.47	-2.66	0.01044
MPV17L2	0.27	7.47	2.66	0.01047
KPNA6	0.21	9.97	2.66	0.01049
MICU2	-0.21	7.94	-2.66	0.01051
ARRDC4	-0.27	7.00	-2.65	0.01052
PPP3CB-AS1	-0.21	6.61	-2.65	0.01052
DMAC1	0.23	8.17	2.65	0.01054
HGH1	0.25	8.73	2.65	0.01058
PIN1	0.21	9.30	2.65	0.01060
LSM2	0.25	8.44	2.65	0.01060
GMEB2	0.14	9.25	2.65	0.01061
SAMD9L	-0.51	8.84	-2.65	0.01062
APRT	0.30	10.78	2.65	0.01063
GMDS	0.23	7.84	2.65	0.01065
LDLRAP1	0.26	11.33	2.65	0.01065
TMSB4XP4	0.34	6.99	2.65	0.01066
NCAPH	-0.24	5.60	-2.65	0.01066
ERP29	0.24	11.83	2.65	0.01066
FCGR2C	-0.39	8.04	-2.65	0.01068
TRIM28	0.19	12.13	2.65	0.01071
EMD	0.21	10.00	2.65	0.01072
MVD	0.26	8.67	2.65	0.01072
KCTD17	0.22	8.17	2.65	0.01072
RPS8	0.30	14.81	2.65	0.01074
FBXO46	0.16	8.52	2.65	0.01075
ATP8B4	-0.26	6.64	-2.65	0.01076
PTPRC	-0.37	12.52	-2.65	0.01078
ITGA4	-0.22	10.12	-2.65	0.01079
INCENP	-0.28	7.03	-2.64	0.01081
SORBS3	0.32	9.48	2.64	0.01084
HMG20B	0.20	9.98	2.64	0.01084
MEFV	-0.40	10.97	-2.64	0.01085
DNAJC30	0.25	7.05	2.64	0.01086
ZFYVE26	-0.18	8.89	-2.64	0.01086
IL2RG	0.32	11.33	2.64	0.01088
ATF7IP	-0.18	9.43	-2.64	0.01089
AC027031.2	0.27	5.17	2.64	0.01090
PYCR3	0.32	6.53	2.64	0.01090
PRR14L	-0.21	8.93	-2.64	0.01090
AC005899.7	0.34	5.37	2.64	0.01094
AC007406.5	-0.28	6.98	-2.64	0.01096
SLC39A4	0.28	7.19	2.64	0.01097
STAT2	-0.40	10.75	-2.64	0.01097

IMPDH2	0.20	10.25	2.64	0.01098
SNX13	-0.28	7.36	-2.64	0.01100
NME3	0.28	8.31	2.64	0.01101
FOXP1	0.28	9.78	2.64	0.01101
MOB2	0.21	9.80	2.64	0.01103
CERT1	-0.23	8.37	-2.64	0.01103
SMG9	0.20	9.23	2.64	0.01105
TADA1	-0.23	6.80	-2.64	0.01106
TCF7L2	-0.30	7.77	-2.64	0.01107
TOP1MT	0.25	8.73	2.63	0.01108
SLC39A3	0.24	8.14	2.63	0.01110
TCERG1	-0.26	7.90	-2.63	0.01111
RPL27A	0.30	13.98	2.63	0.01113
CD3E	0.33	13.13	2.63	0.01116
AC006538.1	0.29	5.01	2.63	0.01119
INKA1	0.30	6.15	2.63	0.01119
AL096865.1	-0.27	5.27	-2.63	0.01122
DAPK3	0.20	9.14	2.63	0.01122
SYNJ1	-0.34	7.28	-2.63	0.01124
COX6B1	0.29	11.60	2.63	0.01124
CC2D1A	0.21	9.22	2.63	0.01126
ILVBL	0.24	8.47	2.63	0.01126
AMDHD2	0.31	8.56	2.63	0.01126
SNTB1	-0.22	7.57	-2.63	0.01129
ATP5ME	0.30	9.42	2.63	0.01131
R3HCC1	0.25	8.97	2.63	0.01132
DDX27	0.15	10.11	2.63	0.01132
INTS5	0.20	8.96	2.63	0.01133
STK11	0.18	10.15	2.63	0.01136
TENT2	-0.23	8.21	-2.62	0.01139
OLFM2	0.27	7.49	2.62	0.01143
LDHB	0.41	8.14	2.62	0.01144
REV3L	-0.26	7.02	-2.62	0.01144
SHISAL2A	0.30	7.27	2.62	0.01144
TIA1	-0.29	8.25	-2.62	0.01145
DYRK1A	-0.26	9.17	-2.62	0.01147
LINC01948	-0.31	6.23	-2.62	0.01149
BRWD1	-0.19	8.01	-2.62	0.01151
POLD1	0.23	8.30	2.62	0.01152
FAM199X	-0.23	7.39	-2.62	0.01152
NDUFB4	0.18	9.25	2.62	0.01153
MAP3K8	-0.24	8.26	-2.62	0.01155
TET3	-0.35	8.99	-2.62	0.01156
MYL9	0.75	9.38	2.62	0.01156
FBXW11	-0.20	8.29	-2.62	0.01159
BTAF1	-0.38	7.41	-2.62	0.01161
PTAR1	-0.27	7.21	-2.62	0.01163
PI16	0.43	7.68	2.62	0.01164
C2orf81	0.27	5.02	2.62	0.01164
AC064847.1	0.38	5.07	2.61	0.01166

AC005912.1	0.40	10.17	2.61	0.01167
MRPL11	0.23	8.19	2.61	0.01167
ZMIZ1	-0.32	10.17	-2.61	0.01167
ARHGDIA	0.26	12.37	2.61	0.01168
RALGAPB	-0.21	8.82	-2.61	0.01170
PRKCE	-0.36	6.67	-2.61	0.01171
AL355816.2	0.36	6.99	2.61	0.01171
MRPL55	0.21	8.44	2.61	0.01173
LINC02555	-0.33	5.09	-2.61	0.01173
BABAM1	0.26	8.76	2.61	0.01174
XPO1	-0.21	9.35	-2.61	0.01174
ZNF341	0.20	7.96	2.61	0.01174
NHSL1	-0.30	5.15	-2.61	0.01176
RNF187	0.26	11.08	2.61	0.01178
FAM89B	0.29	7.91	2.61	0.01183
AP001024.1	0.36	9.11	2.61	0.01183
TBC1D17	0.20	10.25	2.61	0.01184
PHF2	-0.33	9.12	-2.61	0.01184
CRIM1	-0.32	5.50	-2.61	0.01185
AC131392.2	-0.61	6.47	-2.61	0.01186
ANTXR2	-0.22	9.71	-2.61	0.01186
ZFP36L2	-0.18	12.27	-2.61	0.01187
GTF2H5	0.25	7.56	2.61	0.01189
HECA	-0.25	9.84	-2.61	0.01189
PHACTR4	0.24	8.53	2.61	0.01192
DDX60	-0.45	7.70	-2.61	0.01192
PVR	-0.25	7.20	-2.61	0.01192
TRPM4	0.31	6.18	2.61	0.01193
ZGLP1	0.29	5.85	2.61	0.01193
MBD1	-0.14	10.08	-2.61	0.01194
TAF2	-0.29	6.69	-2.61	0.01196
AC007683.1	0.29	6.61	2.61	0.01196
AQP3	0.35	9.79	2.60	0.01198
SLC30A1	-0.23	7.44	-2.60	0.01198
GRAMD1B	-0.30	7.82	-2.60	0.01199
NUDT1	0.31	8.66	2.60	0.01200
NAT14	0.24	6.12	2.60	0.01200
BTBD3	-0.28	5.59	-2.60	0.01202
RABEP2	0.33	8.50	2.60	0.01203
SRGAP2B	-0.30	5.82	-2.60	0.01205
C5orf51	-0.18	7.26	-2.60	0.01205
LAGE3	0.33	8.07	2.60	0.01206
RPS27	0.33	14.52	2.60	0.01207
PARL	0.27	7.29	2.60	0.01207
RPS16	0.28	14.09	2.60	0.01210
TLR6	-0.29	9.13	-2.60	0.01212
DZIP3	-0.25	6.89	-2.60	0.01212
NBR1	-0.21	10.24	-2.60	0.01214
SPHK2	0.21	8.00	2.60	0.01215
CD5	0.26	11.13	2.60	0.01217

ITGB1	-0.34	9.41	-2.60	0.01218
UBXN2B	-0.34	8.86	-2.60	0.01218
ORAI1	0.20	10.68	2.60	0.01220
B3GALT4	0.26	8.96	2.60	0.01221
CCR7	0.39	11.93	2.60	0.01227
EIF2D	0.16	9.26	2.59	0.01232
AL357033.4	0.27	7.62	2.59	0.01233
KDM3A	-0.20	9.71	-2.59	0.01233
PPP2CB	-0.20	7.99	-2.59	0.01235
TCEA2	0.24	8.08	2.59	0.01235
TRADD	0.20	9.81	2.59	0.01238
TSTD1	0.21	8.57	2.59	0.01239
RPL12	0.29	13.44	2.59	0.01242
RAD23A	0.23	10.96	2.59	0.01243
GIMAP1	0.23	10.47	2.59	0.01248
IRS1	-0.34	5.64	-2.59	0.01251
RPGRIP1L	-0.32	5.00	-2.59	0.01252
PPP1R15B	-0.33	8.40	-2.59	0.01253
RPS20P2	0.34	5.36	2.59	0.01255
NBPF1	-0.31	7.25	-2.59	0.01256
RPL23AP42	0.35	10.05	2.59	0.01257
AC073957.3	-0.34	5.69	-2.59	0.01257
TRAF2	0.23	8.44	2.59	0.01258
RPL3P4	0.37	10.89	2.59	0.01259
SLMAP	-0.30	7.78	-2.58	0.01263
SOAT1	-0.22	8.94	-2.58	0.01265
TBC1D7	0.21	7.18	2.58	0.01268
NCBP3	-0.15	9.32	-2.58	0.01268
MTA1	0.19	9.47	2.58	0.01270
CPNE3	-0.21	8.26	-2.58	0.01271
CEP85	-0.20	7.77	-2.58	0.01271
VPS4B	-0.22	9.48	-2.58	0.01273
ATG101	0.23	8.82	2.58	0.01274
IFI27L2	0.34	8.35	2.58	0.01275
SGMS1	-0.29	7.22	-2.58	0.01277
NAXE	0.28	9.22	2.58	0.01277
PRPF6	0.21	11.70	2.58	0.01278
TXNDC16	-0.24	5.78	-2.58	0.01281
SLAIN2	-0.18	8.36	-2.58	0.01282
C1orf122	0.29	7.64	2.58	0.01282
PSPN	0.19	6.19	2.58	0.01282
COX7C	0.27	11.18	2.58	0.01283
FPGS	0.22	9.41	2.58	0.01283
CHMP4A	0.35	6.18	2.58	0.01284
MUL1	0.19	9.41	2.58	0.01285
RPL4P2	0.23	5.50	2.58	0.01288
STK24P1	-0.28	5.25	-2.58	0.01290
KAT5	0.13	10.03	2.57	0.01292
CD2BP2	0.17	10.93	2.57	0.01295
AL355309.1	0.56	8.42	2.57	0.01296

TMEM115	0.19	9.62	2.57	0.01298
TIPARP	-0.29	7.51	-2.57	0.01301
CLUAP1	0.26	7.04	2.57	0.01302
MAT2A	-0.17	9.77	-2.57	0.01303
ERO1B	-0.24	6.34	-2.57	0.01307
TMEM222	0.20	9.78	2.57	0.01308
FAAP20	0.23	8.50	2.57	0.01308
NBPF8	-0.38	7.71	-2.57	0.01310
TONSL	0.25	6.45	2.57	0.01311
GABPA	-0.26	6.81	-2.57	0.01311
UPF3B	0.27	7.81	2.57	0.01313
RAB40C	0.20	8.55	2.57	0.01314
MAPK8	-0.16	7.43	-2.57	0.01314
MARCKSL1	0.21	9.82	2.57	0.01315
TMEM160	0.31	7.11	2.57	0.01315
NCOA2	-0.41	8.26	-2.57	0.01316
FEM1C	-0.26	7.33	-2.57	0.01317
XPO4	-0.22	7.07	-2.57	0.01318
PPP1R13L	0.23	6.09	2.57	0.01318
DNAJC4	0.23	10.13	2.57	0.01322
B4GALT7	0.24	8.74	2.57	0.01322
ASAP1-IT2	0.45	6.09	2.57	0.01323
TMEM39B	0.18	8.84	2.57	0.01324
ANTKMT	0.28	6.67	2.57	0.01324
SMAD1	-0.27	5.55	-2.56	0.01327
KLF16	0.21	9.04	2.56	0.01327
RBM39	-0.23	10.94	-2.56	0.01332
TSTA3	0.24	10.11	2.56	0.01335
LIG4	-0.31	6.08	-2.56	0.01336
PPP1R3D	-0.35	8.13	-2.56	0.01338
AHCYL2	-0.23	7.03	-2.56	0.01339
PGBD2	-0.22	6.98	-2.56	0.01342
GTF2H2C	-0.27	5.82	-2.56	0.01344
ZBTB9	0.19	7.31	2.56	0.01345
GMFB	-0.25	7.09	-2.56	0.01346
GPR34	-0.35	5.36	-2.56	0.01347
ASLP1	0.31	5.19	2.56	0.01347
ZNF10	0.22	5.78	2.56	0.01348
GCDH	0.21	8.51	2.56	0.01348
ZNF671	0.17	8.29	2.56	0.01350
TUT7	-0.31	9.36	-2.56	0.01351
TMEM102	0.22	8.11	2.56	0.01352
ABHD14A	0.29	7.12	2.56	0.01352
ZNF585A	-0.26	5.91	-2.56	0.01353
ZDHHC12	0.25	9.50	2.56	0.01354
RAB22A	-0.17	8.45	-2.56	0.01358
NR2C2	-0.36	8.40	-2.56	0.01359
KLF3	-0.29	9.79	-2.56	0.01359
ATP6V1B2	-0.25	12.08	-2.55	0.01360
RPL7A	0.25	14.34	2.55	0.01366

RDH11	0.27	8.87	2.55	0.01367
MMD	-0.34	7.87	-2.55	0.01367
SEC31A	-0.16	9.92	-2.55	0.01368
ATP2B4	-0.27	10.15	-2.55	0.01369
CCDC117	-0.23	7.68	-2.55	0.01369
SURF2	0.26	7.92	2.55	0.01372
FBXL19	0.22	8.23	2.55	0.01372
PA2G4	0.22	10.53	2.55	0.01373
FBXL18	0.17	8.41	2.55	0.01373
ENDOG	0.32	7.33	2.55	0.01374
WDR25	0.20	7.43	2.55	0.01374
CLTC	-0.19	10.64	-2.55	0.01376
TDRD7	-0.29	8.09	-2.55	0.01376
BDH1	0.26	7.74	2.55	0.01377
AC011495.1	0.32	7.60	2.55	0.01378
FMO5	-0.21	6.09	-2.55	0.01378
RHOQ	-0.19	8.91	-2.55	0.01379
MEST	-0.31	5.45	-2.55	0.01380
TNKS2	-0.26	8.41	-2.55	0.01380
POLD3	-0.19	7.69	-2.55	0.01382
RASAL3	0.19	11.45	2.55	0.01383
AC241585.2	0.21	5.08	2.55	0.01393
TRANK1	-0.25	11.59	-2.55	0.01394
TAB1	0.16	9.38	2.54	0.01395
STK3	-0.32	6.20	-2.54	0.01395
CCDC85C	0.30	6.95	2.54	0.01395
KPNB1	-0.25	11.55	-2.54	0.01396
TBC1D2B	-0.21	9.94	-2.54	0.01396
FBLN7	0.33	6.27	2.54	0.01397
SMARCAD1	-0.23	6.88	-2.54	0.01401
TOMM7	0.29	11.43	2.54	0.01402
PRXL2B	0.23	8.76	2.54	0.01403
TTF2	-0.23	7.07	-2.54	0.01403
AL669983.1	0.30	7.85	2.54	0.01405
RPL13AP7	0.33	8.13	2.54	0.01406
RESF1	-0.37	10.88	-2.54	0.01407
CDKN2B-AS1	0.46	6.79	2.54	0.01407
AC024293.1	0.36	9.50	2.54	0.01407
PEX14	0.21	8.11	2.54	0.01408
TUBGCP3	-0.18	8.75	-2.54	0.01410
H3C9P	-0.27	5.93	-2.54	0.01411
MEIS1	-0.30	6.36	-2.54	0.01413
TRBD1	0.34	6.31	2.54	0.01415
MSL2	-0.20	9.22	-2.54	0.01416
PRPF19	0.22	10.68	2.54	0.01417
NT5M	0.44	7.37	2.54	0.01420
MTR	-0.26	7.87	-2.54	0.01423
UBE2I	0.14	10.90	2.54	0.01424
IRF2	-0.26	10.50	-2.54	0.01425
BX679664.1	0.20	5.17	2.54	0.01426

MRPL54	0.32	8.85	2.54	0.01427
PAXX	0.26	9.32	2.53	0.01431
MCAT	0.26	7.08	2.53	0.01433
NDUFB7	0.27	9.40	2.53	0.01434
RPL35A	0.26	13.75	2.53	0.01435
SLC6A6	-0.38	11.68	-2.53	0.01435
SPRED1	-0.29	5.46	-2.53	0.01437
UCK1	0.16	8.83	2.53	0.01439
RCC1L	0.22	9.31	2.53	0.01440
PIAS4	0.16	8.65	2.53	0.01441
IQGAP1	-0.31	11.70	-2.53	0.01443
REXO4	0.18	9.08	2.53	0.01444
SOS2	-0.36	8.67	-2.53	0.01446
AC004386.1	0.36	6.62	2.53	0.01446
ZBTB1	-0.28	7.93	-2.53	0.01448
NHSL2	-0.23	10.50	-2.53	0.01453
MAP3K14	0.16	10.19	2.53	0.01454
RPS27A	0.26	13.12	2.53	0.01456
SOCS2	-0.20	6.64	-2.53	0.01457
KATNBL1	-0.29	7.72	-2.53	0.01457
AP3S1	-0.23	7.81	-2.53	0.01458
RAB11FIP1	-0.30	11.46	-2.53	0.01463
DNAH10OS	-0.22	5.53	-2.53	0.01465
TPP2	-0.20	8.09	-2.53	0.01466
PHF3	-0.18	9.13	-2.53	0.01467
PLEKHM3	-0.28	7.17	-2.53	0.01467
PCYT1A	-0.16	9.86	-2.52	0.01470
SAP30L	-0.19	9.04	-2.52	0.01474
AC008026.2	0.40	9.03	2.52	0.01475
ZNF333	-0.31	7.94	-2.52	0.01475
ATP9A	-0.42	6.54	-2.52	0.01476
RPL21P120	0.22	5.58	2.52	0.01476
RPS20	0.28	13.63	2.52	0.01476
LRRCC1	0.25	6.00	2.52	0.01477
CMTM6	-0.29	10.92	-2.52	0.01479
RPL21P11	0.22	6.22	2.52	0.01483
RPL12P47	0.31	5.43	2.52	0.01484
ZNF576	0.23	6.99	2.52	0.01485
AGTPBP1	-0.27	9.32	-2.52	0.01486
AC006978.1	0.19	7.49	2.52	0.01491
SCAND1	0.31	8.85	2.52	0.01492
NABP2	0.25	8.53	2.52	0.01498
CORO7	0.23	9.76	2.52	0.01502
PABPC4	0.17	10.82	2.52	0.01502
AC008280.3	0.23	5.01	2.52	0.01504
SGTA	0.19	9.88	2.52	0.01505
SSX2IP	-0.22	6.38	-2.52	0.01505
TAB2	-0.27	9.72	-2.51	0.01505
TRMT2A	0.20	9.31	2.51	0.01505
OPA1	-0.19	7.91	-2.51	0.01506

RFNG	0.21	9.06	2.51	0.01507
SCARB2	-0.25	8.47	-2.51	0.01511
IRF3	0.20	10.69	2.51	0.01512
Z82206.1	0.30	6.85	2.51	0.01518
AC011979.1	0.23	6.20	2.51	0.01518
GOPC	-0.22	7.32	-2.51	0.01521
BTN2A3P	-0.30	5.50	-2.51	0.01525
HERC2P9	-0.31	6.41	-2.51	0.01526
ZMYM2	-0.23	8.19	-2.51	0.01529
PFKL	0.21	11.54	2.51	0.01529
UFL1	-0.29	6.94	-2.51	0.01529
XPOT	-0.22	8.09	-2.51	0.01530
CCDC186	-0.25	7.34	-2.51	0.01533
EIF3B	0.19	11.46	2.51	0.01536
MRTO4	0.21	8.34	2.51	0.01537
RNFT1	-0.25	6.22	-2.51	0.01539
EDEM1	-0.17	9.75	-2.51	0.01540
DAPK1	-0.32	9.04	-2.51	0.01541
EIF3D	0.18	11.90	2.50	0.01544
PRKRIP1	0.16	9.13	2.50	0.01544
TMED9	0.24	11.24	2.50	0.01547
RYBP	-0.29	9.00	-2.50	0.01552
TAFA2	-0.40	5.79	-2.50	0.01552
CRYBG3	-0.25	6.06	-2.50	0.01556
MYSM1	-0.33	6.99	-2.50	0.01558
FCHSD2	-0.19	9.38	-2.50	0.01561
RNF13	-0.30	9.33	-2.50	0.01563
C21orf91	-0.24	7.35	-2.50	0.01565
RPS23P8	0.32	10.15	2.50	0.01566
PPP2R1B	-0.21	7.35	-2.50	0.01567
CCDC142	0.28	8.05	2.50	0.01569
C2CD5	-0.30	7.76	-2.50	0.01570
KIAA0232	-0.25	9.52	-2.50	0.01573
STARD4	-0.28	5.77	-2.50	0.01575
SPSB3	0.23	10.53	2.50	0.01577
RB1CC1	-0.30	8.38	-2.50	0.01579
AL109628.1	0.42	7.34	2.50	0.01580
AP4E1	-0.26	6.63	-2.50	0.01581
PELI2	-0.28	7.96	-2.50	0.01582
MCTP1	-0.28	7.35	-2.49	0.01583
CDK12	-0.20	9.23	-2.49	0.01584
ST6GALNAC4	0.43	9.58	2.49	0.01585
SMG1P2	-0.26	5.38	-2.49	0.01585
BEX3	0.30	8.12	2.49	0.01586
COPE	0.24	11.01	2.49	0.01587
SWSAP1	0.27	7.24	2.49	0.01590
NUCKS1P1	0.25	5.75	2.49	0.01590
PI4K2B	-0.29	6.38	-2.49	0.01592
MTMR6	-0.28	7.79	-2.49	0.01593
GP9	0.47	7.50	2.49	0.01594

LTB4R2	-0.31	5.09	-2.49	0.01595
NDUFA10	0.15	10.02	2.49	0.01596
PDAP1	0.19	10.53	2.49	0.01598
STK38L	-0.26	7.63	-2.49	0.01599
TCF20	-0.19	9.47	-2.49	0.01602
RNF146	-0.26	8.08	-2.49	0.01602
AL031985.3	0.20	5.16	2.49	0.01604
RWDD1	0.22	8.66	2.49	0.01608
NIPBL-DT	-0.24	6.19	-2.49	0.01609
URM1	0.19	9.78	2.49	0.01610
RPS19P7	0.36	6.41	2.49	0.01611
PCSK7	0.20	11.04	2.49	0.01613
AL592114.1	0.22	5.81	2.49	0.01614
COP1	-0.23	9.27	-2.49	0.01619
ZBTB32	0.35	6.04	2.49	0.01621
DNPH1	0.32	7.60	2.48	0.01625
MED13L	-0.39	9.46	-2.48	0.01628
GPI	0.20	11.34	2.48	0.01632
ASPSCR1	0.22	8.68	2.48	0.01635
IFI16	-0.36	11.11	-2.48	0.01635
CEMIP2	-0.28	9.29	-2.48	0.01637
CALCOCO2	-0.16	10.22	-2.48	0.01639
ADAR	-0.29	13.07	-2.48	0.01640
PLEKHJ1	0.19	8.93	2.48	0.01643
AC074032.1	0.29	5.46	2.48	0.01646
RING1	0.18	9.61	2.48	0.01650
CLEC4D	-0.49	7.11	-2.48	0.01651
DYNC1LI1	-0.18	9.02	-2.48	0.01652
TRAPPC8	-0.30	8.13	-2.48	0.01652
WNT1	0.32	5.93	2.48	0.01656
GPCPD1	-0.24	10.26	-2.48	0.01656
ZNF106	-0.23	9.96	-2.48	0.01657
RPL11	0.26	14.35	2.48	0.01658
MBP	-0.23	11.53	-2.48	0.01660
SDE2	-0.25	7.09	-2.48	0.01661
AC024940.3	0.29	7.51	2.48	0.01662
TECPR1	0.15	10.49	2.48	0.01662
PEMT	0.23	7.41	2.47	0.01666
LINC00869	0.20	7.67	2.47	0.01669
AP5M1	-0.17	7.91	-2.47	0.01671
CCS	0.21	9.60	2.47	0.01671
SLC39A6	-0.21	7.60	-2.47	0.01672
PTPN12	-0.34	9.36	-2.47	0.01674
NFE2L2	-0.26	9.20	-2.47	0.01677
SIRPG	0.31	9.22	2.47	0.01677
KPNA1	-0.21	8.99	-2.47	0.01681
MAP2K2	0.20	11.32	2.47	0.01682
ENPP5	-0.27	5.42	-2.47	0.01682
MIR22HG	-0.31	6.69	-2.47	0.01685
TXNRD2	0.20	8.86	2.47	0.01686

AL138799.3	0.32	5.19	2.47	0.01691
TRAP1	0.19	9.57	2.47	0.01691
EFR3A	-0.18	8.30	-2.47	0.01692
RHOH	0.22	10.06	2.47	0.01694
TAP2	-0.26	9.42	-2.47	0.01702
AC068446.1	-0.34	5.25	-2.47	0.01704
FAM13A-AS1	-0.36	8.84	-2.47	0.01704
SSR4	0.28	11.41	2.47	0.01705
DISC1	-0.30	7.11	-2.46	0.01710
HNRNPA2B1	-0.18	11.65	-2.46	0.01711
AL157392.3	-0.37	7.16	-2.46	0.01711
ABTB2	-0.36	6.09	-2.46	0.01712
AC087633.2	0.38	8.77	2.46	0.01713
SSNA1	0.23	9.99	2.46	0.01714
JAK2	-0.37	7.77	-2.46	0.01718
NELFB	0.19	10.27	2.46	0.01719
RNF168	-0.18	8.03	-2.46	0.01721
TRBJ2-2P	0.29	4.97	2.46	0.01723
CPNE8	-0.26	5.82	-2.46	0.01726
LINC01410	-0.37	7.10	-2.46	0.01726
C6orf62	-0.18	10.78	-2.46	0.01727
NR1H2	0.19	10.59	2.46	0.01729
PLEKHM2	0.14	10.76	2.46	0.01729
MLLT10	-0.19	7.93	-2.46	0.01733
AC087650.1	0.27	5.57	2.46	0.01733
GAPVD1	-0.22	8.63	-2.46	0.01736
TMEM101	0.19	8.68	2.46	0.01738
KLF2	0.19	12.92	2.46	0.01738
DCLRE1A	-0.24	6.08	-2.46	0.01741
CDIP1	0.18	9.62	2.46	0.01742
TMIGD2	0.34	7.90	2.46	0.01747
RNF111	-0.28	7.97	-2.46	0.01748
EPN1	0.26	10.54	2.45	0.01750
UBR4	-0.17	11.27	-2.45	0.01750
RPL26P37	0.23	5.20	2.45	0.01752
DUS3L	0.21	8.47	2.45	0.01753
ERAP1	-0.33	9.23	-2.45	0.01757
AC114728.1	0.32	7.39	2.45	0.01757
NDUFA2	0.24	9.72	2.45	0.01758
RSRC2	-0.16	9.15	-2.45	0.01759
DYM	0.15	8.99	2.45	0.01759
ACTR1B	0.13	10.19	2.45	0.01761
AP002360.2	0.24	5.79	2.45	0.01764
PRNP	-0.19	9.19	-2.45	0.01765
GNS	-0.23	10.74	-2.45	0.01766
MMP24OS	0.26	10.00	2.45	0.01767
POC5	-0.18	6.97	-2.45	0.01767
MTLN	0.25	7.56	2.45	0.01767
AC243960.2	-0.31	6.49	-2.45	0.01771
DNAJC25	-0.24	5.41	-2.45	0.01772

ZNF574	0.15	9.12	2.45	0.01773
MT-ND5	-0.22	13.40	-2.45	0.01775
SVIL	-0.33	10.09	-2.45	0.01779
ITPR2	-0.21	7.83	-2.45	0.01782
CYB5R3	0.21	10.72	2.45	0.01782
HMGCR	-0.24	8.61	-2.45	0.01786
MLST8	0.21	8.76	2.45	0.01787
PRKCQ-AS1	0.27	8.52	2.45	0.01787
EEF1DP1	0.34	8.89	2.45	0.01789
ZNF627	-0.22	6.46	-2.45	0.01789
NOC4L	0.24	8.50	2.44	0.01793
TMC6	0.19	11.66	2.44	0.01797
C1orf35	0.23	8.10	2.44	0.01797
ACAP2	-0.25	9.53	-2.44	0.01798
YWHAG	-0.21	9.38	-2.44	0.01802
LSR	0.28	8.22	2.44	0.01805
FLYWCH2	0.22	7.67	2.44	0.01806
AC025171.1	0.19	6.81	2.44	0.01806
AP000769.1	0.33	6.22	2.44	0.01811
BRF1	0.20	9.00	2.44	0.01812
CD302	-0.23	8.85	-2.44	0.01813
STIM2	-0.15	8.41	-2.44	0.01813
DDX49	0.21	9.27	2.44	0.01814
GTF2E1	-0.18	7.05	-2.44	0.01815
CHM	-0.25	6.21	-2.44	0.01815
VNN1	-0.48	7.50	-2.44	0.01815
SIL1	0.26	8.20	2.44	0.01817
TIMM13	0.26	9.10	2.44	0.01818
TMEM80	0.23	7.92	2.44	0.01822
PLSCR1	-0.51	8.77	-2.44	0.01822
ADRM1	0.21	10.35	2.44	0.01823
GPX4	0.26	11.68	2.44	0.01824
NR3C1	-0.23	9.09	-2.44	0.01824
C11orf68	0.20	10.09	2.44	0.01825
SEPTIN6	0.18	11.73	2.44	0.01827
GPAA1	0.19	10.07	2.44	0.01827
PLEKHB1	0.31	8.65	2.44	0.01828
WDFY3	-0.46	8.92	-2.44	0.01829
SH3BGRL3	0.26	13.90	2.44	0.01829
TWF1	-0.25	6.26	-2.44	0.01831
CDCA4	0.19	7.25	2.44	0.01833
TMEM39A	-0.17	7.31	-2.44	0.01837
DSE	-0.22	7.73	-2.43	0.01842
PIGA	-0.23	5.85	-2.43	0.01845
MRPL2	0.26	5.95	2.43	0.01845
TUG1	-0.15	9.98	-2.43	0.01845
GFER	0.21	8.10	2.43	0.01846
NDUFA11	0.25	8.12	2.43	0.01847
NPEPPS	-0.22	9.69	-2.43	0.01847
PREP	-0.13	8.96	-2.43	0.01853

MRPL41	0.28	8.65	2.43	0.01853
MAP1S	0.23	9.68	2.43	0.01856
MEGF9	-0.39	10.63	-2.43	0.01857
C17orf58	-0.22	5.26	-2.43	0.01857
SEC63	-0.18	8.24	-2.43	0.01858
MMUT	-0.23	6.99	-2.43	0.01858
COPB2	-0.16	9.74	-2.43	0.01860
WDR5B	-0.20	6.57	-2.43	0.01863
MIF	0.34	6.95	2.43	0.01865
PACS1	0.15	12.54	2.43	0.01866
SERF2	0.33	13.58	2.43	0.01866
LDLR	-0.29	7.83	-2.43	0.01868
ST8SIA4	-0.28	8.92	-2.43	0.01869
CRLF3	-0.24	9.15	-2.43	0.01870
FAR1	-0.23	8.56	-2.43	0.01875
SRSF8	0.17	9.51	2.43	0.01879
NAALADL1	0.25	7.77	2.42	0.01884
NDUFB8P2	0.21	5.30	2.42	0.01884
ZBTB37	-0.29	7.71	-2.42	0.01886
TCF7	0.33	12.58	2.42	0.01888
DCBLD1	-0.23	5.82	-2.42	0.01888
CMPK2	-0.66	6.75	-2.42	0.01889
KCTD9	-0.26	5.96	-2.42	0.01891
CASP6	0.20	7.17	2.42	0.01896
STX8	0.21	8.61	2.42	0.01896
LIMS2	0.39	6.95	2.42	0.01906
NFXL1	-0.38	6.52	-2.42	0.01906
ZBTB33	-0.18	7.52	-2.42	0.01907
FHIT	0.46	7.95	2.42	0.01909
PGBD1	-0.26	5.02	-2.42	0.01910
NAA60	0.15	9.99	2.42	0.01911
UBALD1	0.27	9.09	2.42	0.01913
NACC2	-0.18	9.69	-2.42	0.01916
MCM5	0.18	10.47	2.42	0.01918
IFIH1	-0.39	8.42	-2.42	0.01918
PRKAG2	-0.17	8.69	-2.42	0.01922
SLC40A1	-0.35	9.19	-2.42	0.01922
PAPSS1	-0.27	8.55	-2.42	0.01922
MCRS1	0.17	10.32	2.42	0.01923
YIF1A	0.22	8.26	2.42	0.01924
TGFBR2	-0.23	10.90	-2.42	0.01927
SNX18	-0.28	8.73	-2.42	0.01927
TMF1	-0.21	8.10	-2.42	0.01927
BOP1	0.23	8.95	2.42	0.01928
DEXI	0.21	8.72	2.42	0.01928
PPP2R1A	0.20	11.87	2.42	0.01929
CNEP1R1	-0.29	7.28	-2.41	0.01933
DDX39A	0.18	10.26	2.41	0.01934
TSSC4	0.22	8.88	2.41	0.01938
SSR2	0.21	11.96	2.41	0.01938

PES1	0.22	9.72	2.41	0.01940
DST	-0.23	6.35	-2.41	0.01942
NECAP1	-0.18	7.89	-2.41	0.01943
FOXJ3	-0.19	8.64	-2.41	0.01944
AARS1	0.20	9.74	2.41	0.01947
PROSER1	-0.16	8.81	-2.41	0.01947
PLP2	0.36	11.93	2.41	0.01950
RUNDC1	0.20	8.67	2.41	0.01950
MAML3	-0.33	8.02	-2.41	0.01950
POU2F2	0.38	11.86	2.41	0.01951
IPO7	-0.22	8.38	-2.41	0.01952
LNPEP	-0.19	9.47	-2.41	0.01955
AC025164.2	0.22	5.35	2.41	0.01960
RNASEL	-0.25	8.84	-2.41	0.01961
LBR	-0.33	9.87	-2.41	0.01961
GGCX	-0.17	7.82	-2.41	0.01965
KIAA0753	-0.19	7.25	-2.41	0.01965
AP1S3	0.29	5.59	2.41	0.01965
SLC39A10	-0.25	6.56	-2.41	0.01965
UBQLN1	-0.19	9.38	-2.41	0.01966
EPG5	-0.24	8.76	-2.41	0.01968
AC074085.2	0.30	6.88	2.41	0.01968
BROX	-0.21	8.54	-2.41	0.01969
LRCH1	-0.19	7.21	-2.41	0.01969
ABI1	-0.22	9.30	-2.41	0.01971
RCBTB1	-0.26	7.03	-2.41	0.01975
MEF2C-AS1	-0.27	5.09	-2.41	0.01976
ZNF28	-0.16	6.41	-2.41	0.01977
KLHL2	-0.45	8.11	-2.41	0.01977
SLC36A1	-0.33	8.30	-2.40	0.01981
WDR7	-0.18	7.76	-2.40	0.01983
AC007969.1	0.37	10.15	2.40	0.01987
USP38	-0.28	7.73	-2.40	0.01988
IRF2BP1	0.18	8.54	2.40	0.01989
JMJD8	0.23	9.45	2.40	0.01992
ASCC3	-0.31	7.08	-2.40	0.01996
PELP1	0.22	9.67	2.40	0.01996
L3HYPDH	-0.25	5.66	-2.40	0.01999
GPR137	0.20	8.41	2.40	0.01999
HIF1A	-0.35	8.77	-2.40	0.01999
AKT1S1	0.28	8.90	2.40	0.02001
UQCC3	0.25	7.02	2.40	0.02005
AATF	0.18	10.32	2.40	0.02012
TAB3	-0.26	7.28	-2.40	0.02013
KCNE3	-0.28	9.87	-2.40	0.02013
SEC24B	-0.20	8.06	-2.40	0.02013
CEACAM1	-0.57	8.26	-2.40	0.02014
GORAB	-0.24	5.91	-2.40	0.02025
SNAPC2	0.27	8.38	2.40	0.02026
FP565260.3	0.31	5.37	2.40	0.02026

TCF3	0.20	9.76	2.39	0.02030
TBC1D22A	0.17	10.11	2.39	0.02031
DNAJC3	-0.26	9.27	-2.39	0.02036
CHSY1	-0.40	8.56	-2.39	0.02038
HSD17B8	0.25	7.36	2.39	0.02041
ALG13	-0.23	7.78	-2.39	0.02044
TPR	-0.20	10.35	-2.39	0.02045
APOBEC3C	0.27	11.23	2.39	0.02046
TECR	0.23	10.80	2.39	0.02046
RIF1	-0.21	7.66	-2.39	0.02048
CKAP2	-0.22	6.44	-2.39	0.02049
ATP5PO	0.24	8.55	2.39	0.02051
M1AP	0.27	4.99	2.39	0.02053
ODF2L	-0.22	7.05	-2.39	0.02053
NUCKS1	0.22	10.40	2.39	0.02055
FAM86C1	0.26	6.52	2.39	0.02056
PCMTD1	-0.23	8.47	-2.39	0.02056
NFIC	0.26	9.77	2.39	0.02056
CASP8	-0.24	10.35	-2.39	0.02057
FCGR3B	-0.48	14.16	-2.39	0.02059
IDH3G	0.20	10.77	2.39	0.02061
PSMD5	-0.15	7.79	-2.39	0.02061
OAS3	-0.69	9.24	-2.39	0.02061
MFAP3	-0.31	5.72	-2.39	0.02063
HDAC11	0.24	7.29	2.39	0.02063
HEATR5B	-0.22	8.46	-2.39	0.02065
ANKS1A	-0.26	8.49	-2.39	0.02066
NSD1	-0.13	10.27	-2.39	0.02069
SNRPD2	0.27	10.94	2.39	0.02069
CHCHD10	0.31	8.42	2.38	0.02079
CENPN	-0.26	5.34	-2.38	0.02083
HSF1	0.15	10.42	2.38	0.02084
ZNFX1	-0.22	11.43	-2.38	0.02090
GATA3	0.21	9.36	2.38	0.02092
PPIH	0.20	7.98	2.38	0.02094
CERS4	0.24	8.45	2.38	0.02095
ELOVL5	-0.27	9.98	-2.38	0.02097
RMI1	-0.23	6.43	-2.38	0.02097
LYRM9	0.19	7.25	2.38	0.02099
NUS1	-0.24	6.97	-2.38	0.02099
BAG3	0.26	8.05	2.38	0.02102
AP000281.2	0.27	6.85	2.38	0.02103
NR2C2AP	0.19	8.25	2.38	0.02104
AAK1	0.22	10.41	2.38	0.02107
CDK4	0.19	9.13	2.38	0.02109
RANBP6	-0.24	6.84	-2.38	0.02109
ELF4	-0.19	11.25	-2.38	0.02110
SACM1L	-0.18	8.39	-2.38	0.02113
PHGDH	0.37	6.52	2.38	0.02114
CTNS	0.15	8.15	2.38	0.02114

ATF3	-0.31	5.61	-2.38	0.02115
SELENON	0.21	9.43	2.38	0.02115
KIAA1958	-0.29	5.82	-2.38	0.02117
ZC3H14	-0.15	7.89	-2.38	0.02117
NFKBIZ	-0.34	8.57	-2.38	0.02117
RNLS	-0.19	5.32	-2.38	0.02118
CNOT10	0.13	8.59	2.38	0.02119
NUP58	-0.30	7.80	-2.38	0.02121
TMEM170B	-0.22	8.01	-2.38	0.02128
NEK7	-0.34	8.00	-2.37	0.02130
AGFG1	-0.29	9.16	-2.37	0.02133
FGD6	-0.21	6.14	-2.37	0.02135
ZNF468	-0.22	6.69	-2.37	0.02135
SLC16A6	-0.23	7.48	-2.37	0.02143
ACOT7	0.20	7.38	2.37	0.02143
CPEB3	-0.28	5.65	-2.37	0.02145
KLC4	0.13	8.72	2.37	0.02146
C15orf62	0.22	5.38	2.37	0.02153
STX17	-0.18	7.62	-2.37	0.02157
ABHD2	-0.27	11.44	-2.37	0.02160
C3orf38	-0.20	7.23	-2.37	0.02161
MXD4	0.18	10.82	2.37	0.02164
WDR26	-0.24	10.22	-2.37	0.02173
LPAL2	-0.32	5.73	-2.37	0.02175
LINC01137	0.23	6.80	2.37	0.02181
ACVR1B	-0.27	8.42	-2.36	0.02182
ANKRD44	-0.25	9.92	-2.36	0.02186
MAP4K1	0.21	11.03	2.36	0.02189
GTF3C3	-0.17	7.36	-2.36	0.02189
ARRDC3	-0.35	9.29	-2.36	0.02192
MINDY3	-0.19	6.20	-2.36	0.02193
PAAF1	0.20	7.52	2.36	0.02195
FLI1	-0.24	10.95	-2.36	0.02195
RPL30	0.22	14.32	2.36	0.02198
PNPLA7	0.35	7.01	2.36	0.02206
FNDC3B	-0.36	8.18	-2.36	0.02208
YTHDF3	-0.24	8.23	-2.36	0.02209
SCLT1	-0.26	8.18	-2.36	0.02212
FBN2	-0.37	6.88	-2.36	0.02213
MRPL33	0.17	8.05	2.36	0.02213
RPS19P1	0.34	8.75	2.36	0.02214
CCDC146	-0.29	6.77	-2.36	0.02217
DGCR6L	0.26	9.15	2.36	0.02219
SNX19	-0.17	8.98	-2.36	0.02219
SIK3	-0.16	10.42	-2.36	0.02225
AC092868.2	0.29	5.22	2.36	0.02229
ZUP1	-0.24	6.34	-2.36	0.02229
KRT18P5	0.31	4.99	2.36	0.02230
DIPK2A	-0.20	6.90	-2.36	0.02230
MSL1	-0.35	10.99	-2.36	0.02233

CSNK1A1	-0.17	8.91	-2.36	0.02234
SAMD8	-0.24	7.59	-2.35	0.02237
PGM2L1	0.24	6.96	2.35	0.02244
FGD4	-0.30	7.71	-2.35	0.02244
FCMR	0.25	11.89	2.35	0.02245
IGF2R	-0.37	13.02	-2.35	0.02245
STX11	-0.39	10.09	-2.35	0.02247
COQ4	0.24	9.09	2.35	0.02252
AL139174.1	-0.23	5.08	-2.35	0.02252
ATAD2	-0.31	6.39	-2.35	0.02252
FMC1	0.25	5.20	2.35	0.02255
MAGED1	0.18	9.25	2.35	0.02256
TNIP1	0.22	12.99	2.35	0.02256
AC244502.1	-0.25	5.52	-2.35	0.02259
NEMP1	-0.24	6.84	-2.35	0.02263
RBL2	-0.33	10.18	-2.35	0.02263
SPIN4	-0.24	5.20	-2.35	0.02265
F2RL1	-0.43	7.76	-2.35	0.02265
MAP4K3	-0.21	5.45	-2.35	0.02266
TM9SF2	-0.23	10.10	-2.35	0.02272
RPL21P75	0.28	8.50	2.35	0.02272
TMEM203	0.18	9.33	2.35	0.02276
VPS29	-0.16	8.94	-2.35	0.02278
HNRNPH3	-0.17	9.81	-2.35	0.02280
PPP2R5D	0.12	9.52	2.35	0.02282
ATL3	-0.17	8.48	-2.35	0.02283
KXD1	0.15	10.66	2.35	0.02283
TRAM2	-0.18	7.84	-2.35	0.02284
HDAC3	0.12	9.66	2.35	0.02285
AL079342.1	0.34	5.80	2.35	0.02287
C7orf50	0.32	8.83	2.35	0.02287
DNAL1	0.21	6.03	2.35	0.02288
LTBP3	0.30	10.42	2.34	0.02292
PGP	0.17	8.35	2.34	0.02292
DDX59	-0.18	7.60	-2.34	0.02292
SEPTIN7	-0.21	9.61	-2.34	0.02292
FKBP9	-0.34	5.87	-2.34	0.02294
DPH5	0.20	7.94	2.34	0.02296
AMOTL1	-0.24	5.37	-2.34	0.02297
EIF4E3	-0.26	9.17	-2.34	0.02298
SUMF2	0.15	10.16	2.34	0.02303
HDGFL2	0.18	9.44	2.34	0.02304
ALG3	0.21	9.04	2.34	0.02305
FASTK	0.20	9.70	2.34	0.02308
CNTRL	-0.19	9.39	-2.34	0.02314
AC002091.1	-0.28	6.04	-2.34	0.02315
SPNS3	0.35	7.86	2.34	0.02316
RHBDD2	0.20	10.19	2.34	0.02320
TMEM256	0.21	6.62	2.34	0.02320
MDGA1	0.93	6.77	2.34	0.02322

NOD2	-0.34	9.18	-2.34	0.02324
NUP88	0.16	8.58	2.34	0.02326
RAB8B	-0.21	8.60	-2.34	0.02326
PIH1D1	0.20	9.75	2.34	0.02327
MTM1	-0.25	7.32	-2.34	0.02331
FAM50A	0.21	10.15	2.34	0.02333
DOCK5	-0.35	10.36	-2.34	0.02335
VPS50	-0.18	6.98	-2.34	0.02335
SLC11A2	-0.18	7.45	-2.34	0.02343
GPATCH8	-0.23	9.01	-2.34	0.02344
GALK1	0.27	8.68	2.34	0.02345
LATS1	-0.27	6.95	-2.33	0.02348
GDF11	0.20	7.88	2.33	0.02351
ARFGAP1	0.18	10.43	2.33	0.02351
CETP	0.25	5.53	2.33	0.02357
FARSA	0.21	9.73	2.33	0.02359
GPBP1L1	-0.16	10.20	-2.33	0.02360
SLC8A1	-0.43	7.51	-2.33	0.02363
HABP4	0.24	8.02	2.33	0.02370
PAK2	-0.19	10.62	-2.33	0.02379
CTDSP1	0.16	12.39	2.33	0.02395
LINC00847	0.16	7.80	2.32	0.02404
SLC38A9	-0.16	6.35	-2.32	0.02405
LSM14A	-0.15	10.08	-2.32	0.02406
GPX1P1	0.33	7.52	2.32	0.02411
RPTOR	0.20	9.50	2.32	0.02413
TMEM154	-0.30	10.49	-2.32	0.02414
CHERP	0.13	9.73	2.32	0.02418
C11orf49	0.19	7.56	2.32	0.02418
CD37	0.21	14.07	2.32	0.02425
FAM102B	-0.15	7.49	-2.32	0.02426
PSEN1	-0.19	10.46	-2.32	0.02427
ZMYND11	-0.22	7.97	-2.32	0.02427
IVNS1ABP	-0.25	10.38	-2.32	0.02428
TRAV23DV6	0.32	5.88	2.32	0.02430
EMB	-0.18	9.69	-2.32	0.02432
TMCC1	-0.26	8.81	-2.32	0.02433
GPD1L	-0.16	7.39	-2.32	0.02434
ROGDI	0.32	8.31	2.32	0.02435
IL1RAP	-0.49	7.20	-2.32	0.02436
MRPS15	0.19	9.22	2.32	0.02436
AL663070.2	0.23	6.29	2.32	0.02438
RPLP0	0.24	14.36	2.32	0.02443
PHF14	0.25	8.37	2.32	0.02445
MRPL28	0.22	9.74	2.32	0.02447
PANX1	-0.20	6.54	-2.32	0.02453
MRPL45	0.17	7.97	2.32	0.02454
MPP7	-0.25	7.28	-2.32	0.02454
WDR34	0.28	7.08	2.32	0.02455
ST7	0.17	6.88	2.32	0.02455

RASSF3	-0.29	10.63	-2.32	0.02457
ELMO2	-0.18	10.00	-2.32	0.02458
TBK1	-0.25	7.66	-2.32	0.02459
ECH1	0.23	10.57	2.32	0.02460
ZNF48	0.19	7.82	2.32	0.02461
RASSF2	-0.27	12.06	-2.31	0.02462
ARHGAP12	-0.26	6.38	-2.31	0.02464
UBIAD1	0.21	8.70	2.31	0.02466
MOB1A	-0.16	10.61	-2.31	0.02466
CHMP1B	-0.20	10.08	-2.31	0.02467
ANAPC15	0.22	9.05	2.31	0.02469
MYO10	-0.34	5.10	-2.31	0.02474
SDCBP	-0.34	11.56	-2.31	0.02477
AC144530.1	0.32	6.94	2.31	0.02478
CASP10	-0.16	9.15	-2.31	0.02478
STK39	-0.18	7.94	-2.31	0.02479
AC034236.1	0.31	10.11	2.31	0.02486
RAD21	-0.24	10.25	-2.31	0.02487
POU2F1	0.26	9.18	2.31	0.02490
DYNLL2	0.16	10.63	2.31	0.02493
PPM1G	0.17	10.72	2.31	0.02495
PUM2	-0.18	9.87	-2.31	0.02504
CDC37	0.17	11.64	2.31	0.02504
ING3	-0.17	7.36	-2.31	0.02505
STAM	-0.21	7.32	-2.31	0.02506
WDR37	-0.16	9.60	-2.31	0.02512
MORC3	-0.34	8.21	-2.31	0.02518
TSPOAP1-AS1	0.16	9.30	2.31	0.02518
PTPMT1	0.20	6.65	2.31	0.02519
AC008957.1	-0.37	5.38	-2.31	0.02520
ARSB	-0.20	7.67	-2.30	0.02523
PKIG	0.32	7.54	2.30	0.02527
SARNP	0.22	5.09	2.30	0.02530
ACAP1	0.20	12.34	2.30	0.02530
PTPN9	-0.15	8.75	-2.30	0.02532
ATP5MF	0.28	8.91	2.30	0.02536
TWF2	0.21	10.03	2.30	0.02540
TAX1BP3	0.31	6.61	2.30	0.02541
SRPK1	-0.35	9.22	-2.30	0.02547
FZR1	0.14	10.14	2.30	0.02549
UBA3	-0.22	7.98	-2.30	0.02553
SHARPIN	0.32	10.52	2.30	0.02553
TRMT112	0.20	10.58	2.30	0.02557
ANKHD1	0.25	5.67	2.30	0.02560
TNKS1BP1	0.19	7.03	2.30	0.02561
RCC1	0.17	8.17	2.30	0.02568
AC139272.1	-0.53	5.89	-2.30	0.02573
FIS1	0.25	10.57	2.30	0.02575
SURF6	0.18	8.65	2.30	0.02575
S100A11P2	-0.23	5.44	-2.30	0.02576

PRKD3	-0.24	7.65	-2.30	0.02577
HEXIM2	0.29	7.00	2.30	0.02578
TPT1	0.19	15.29	2.30	0.02582
AC116913.1	0.21	5.99	2.29	0.02583
MGLL	0.32	8.93	2.29	0.02584
PCMTD2	-0.25	8.40	-2.29	0.02584
MRPL17	0.23	8.75	2.29	0.02588
DGKG	-0.23	7.08	-2.29	0.02594
SRP54	-0.13	8.38	-2.29	0.02594
ZNF449	-0.21	5.54	-2.29	0.02595
G3BP2	-0.19	9.08	-2.29	0.02599
PARD6A	0.22	6.83	2.29	0.02599
PRDM2	-0.17	9.53	-2.29	0.02601
NT5C2	-0.25	10.51	-2.29	0.02606
RPS7P3	0.25	5.15	2.29	0.02608
IREB2	-0.22	7.84	-2.29	0.02608
PTPRE	-0.24	10.92	-2.29	0.02610
UNC50	-0.13	8.00	-2.29	0.02614
POLR2B	-0.15	9.65	-2.29	0.02614
VPS36	-0.19	8.22	-2.29	0.02615
ARRDC5	0.34	6.89	2.29	0.02615
MED18	0.19	8.27	2.29	0.02616
DDHD1	-0.23	7.54	-2.29	0.02616
POC1B	-0.25	6.93	-2.29	0.02617
C8orf58	0.24	7.02	2.29	0.02618
MT-TG	0.32	5.58	2.29	0.02619
MICOS10	0.15	6.91	2.29	0.02620
BRMS1	0.21	10.22	2.29	0.02621
PTMAP5	0.24	9.18	2.29	0.02623
PCNP	-0.18	8.59	-2.29	0.02625
CSRP1	0.18	10.03	2.29	0.02628
EIF5B	0.33	10.41	2.29	0.02629
TRAF3IP2	0.19	7.93	2.29	0.02629
FBRSL1	0.20	10.00	2.29	0.02631
PITPNC1	0.16	9.84	2.29	0.02632
ATP6V1F	0.24	10.43	2.29	0.02636
SLC66A3	-0.17	8.27	-2.29	0.02643
RPS24	0.22	13.76	2.29	0.02643
SIRT2	0.13	10.17	2.29	0.02644
RALGAPA1	-0.21	6.50	-2.28	0.02646
AC245100.8	0.23	6.73	2.28	0.02646
UBN2	-0.24	7.16	-2.28	0.02648
ZNF708	-0.25	6.27	-2.28	0.02648
NHS	-0.28	5.85	-2.28	0.02653
RPL9	0.28	12.84	2.28	0.02653
STAM2	-0.19	7.59	-2.28	0.02658
RPS19BP1	0.20	9.94	2.28	0.02662
AC010359.3	0.25	5.17	2.28	0.02666
AC105399.1	0.36	7.63	2.28	0.02666
OBI1	-0.17	6.84	-2.28	0.02666

ZNF628	0.24	6.87	2.28	0.02667
DYNLRB1	0.27	8.16	2.28	0.02668
VEZF1	-0.13	8.95	-2.28	0.02670
RPL13AP25	0.35	10.62	2.28	0.02675
AC092620.1	0.44	6.65	2.28	0.02677
ZBTB42	0.17	6.82	2.28	0.02684
TRIM25	-0.38	11.08	-2.28	0.02684
NDUFB8	0.25	7.77	2.28	0.02695
CDC42SE1	-0.21	12.65	-2.28	0.02695
SLC25A23	0.24	8.44	2.28	0.02696
KRAS	-0.18	8.26	-2.28	0.02701
FBXO11	-0.23	8.45	-2.28	0.02702
RPL29P11	0.44	7.09	2.28	0.02702
PAXBP1	-0.25	7.25	-2.28	0.02703
ZNF518B	-0.20	7.28	-2.28	0.02707
AC125807.1	0.23	5.80	2.28	0.02708
GPATCH2L	-0.13	8.32	-2.27	0.02712
DDRGK1	0.20	9.06	2.27	0.02713
VEGFA	-0.28	7.24	-2.27	0.02717
MGMT	0.28	8.13	2.27	0.02721
DGKQ	0.26	9.52	2.27	0.02724
ZNF700	-0.28	7.47	-2.27	0.02725
AC005082.1	-0.28	4.94	-2.27	0.02730
YPEL2	-0.22	7.89	-2.27	0.02736
ADK	0.26	7.59	2.27	0.02739
UBE2D4	0.18	7.50	2.27	0.02739
GLIPR1	-0.17	10.49	-2.27	0.02747
CAMSAP2	-0.25	5.00	-2.27	0.02749
PHC3	-0.22	8.11	-2.27	0.02749
NBN	-0.22	8.20	-2.27	0.02750
ATRIP	0.17	7.82	2.27	0.02750
DHRS9	-0.41	8.09	-2.27	0.02752
VARS1	0.19	10.16	2.27	0.02754
AC103769.1	0.38	5.60	2.27	0.02761
RNF38	-0.18	8.76	-2.27	0.02764
CLCN3	-0.24	7.69	-2.27	0.02766
RPL6P27	0.31	10.64	2.27	0.02766
RPL34	0.27	13.16	2.27	0.02767
NUP107	-0.20	7.67	-2.27	0.02769
CAPRIN1	-0.15	9.85	-2.26	0.02773
PRMT7	0.17	8.77	2.26	0.02775
DGKZ	0.13	12.25	2.26	0.02781
AGO1	-0.11	9.55	-2.26	0.02783
PRXL2A	0.32	6.48	2.26	0.02783
KMT5B	-0.13	9.27	-2.26	0.02784
P2RY13	-0.31	10.83	-2.26	0.02785
LONRF3	0.22	5.36	2.26	0.02785
ATP2C1	-0.16	8.15	-2.26	0.02790
GPAT3	-0.29	8.39	-2.26	0.02791
AP005019.1	0.25	5.95	2.26	0.02792

ZNF707	0.16	8.20	2.26	0.02794
C1GALT1C1	-0.18	7.17	-2.26	0.02794
MCL1	-0.25	13.21	-2.26	0.02795
AL451085.1	0.36	5.47	2.26	0.02800
CCDC130	0.18	9.36	2.26	0.02802
STAG1	-0.16	8.29	-2.26	0.02802
PTPN7	0.17	10.34	2.26	0.02806
PUF60	0.18	10.94	2.26	0.02806
MRPL40	0.20	8.15	2.26	0.02808
SP100	-0.22	10.48	-2.26	0.02814
MED6	0.16	8.00	2.26	0.02817
RPL21	0.26	13.85	2.26	0.02818
MOSPD3	0.22	8.39	2.26	0.02819
RECQL	-0.20	8.25	-2.26	0.02820
BRD9	0.13	9.55	2.26	0.02821
MRPS2	0.23	8.84	2.26	0.02821
TMX1	-0.19	7.73	-2.26	0.02825
SLC12A6	-0.29	10.40	-2.26	0.02829
COA4	0.19	8.37	2.26	0.02830
MT1F	0.27	7.08	2.26	0.02836
KDM2A	-0.21	11.37	-2.26	0.02838
TNFAIP6	-0.50	6.98	-2.25	0.02843
FBLN5	0.36	7.76	2.25	0.02844
TELO2	0.21	8.78	2.25	0.02845
AL590682.1	0.21	6.08	2.25	0.02848
AC123912.4	-0.66	6.73	-2.25	0.02850
AL118508.3	-0.29	5.21	-2.25	0.02852
RPS2P48	0.18	5.22	2.25	0.02852
CPSF4	0.16	8.72	2.25	0.02853
COPA	-0.15	10.86	-2.25	0.02853
RNF141	-0.26	9.14	-2.25	0.02856
SSH2	-0.26	11.60	-2.25	0.02857
AGMAT	0.27	7.01	2.25	0.02860
PIK3R5	-0.18	11.68	-2.25	0.02860
ZFR	-0.26	8.17	-2.25	0.02861
PC	0.26	6.78	2.25	0.02862
SLC25A26	0.20	7.81	2.25	0.02864
ARIH2	-0.11	9.87	-2.25	0.02865
ADM2	0.25	5.76	2.25	0.02866
ERLIN2	-0.16	7.63	-2.25	0.02867
SF3B5	0.23	10.79	2.25	0.02876
LYSMD3	-0.21	6.74	-2.25	0.02878
GCLC	-0.24	7.11	-2.25	0.02881
PWP2	0.38	5.81	2.25	0.02882
FAM189B	0.16	8.22	2.25	0.02886
YWHAZ	-0.14	12.23	-2.25	0.02886
MED12	-0.18	10.41	-2.25	0.02888
AGO4	-0.30	10.19	-2.25	0.02889
NRBF2	-0.34	9.78	-2.25	0.02894
SYP	0.20	5.69	2.25	0.02895

LIG1	0.18	8.89	2.25	0.02897
PAFAH1B1	-0.12	10.53	-2.25	0.02897
RPP14	0.16	7.61	2.25	0.02898
PF4	0.39	10.80	2.25	0.02905
RRN3P3	0.29	7.13	2.25	0.02905
SPAG9	-0.27	8.78	-2.24	0.02908
NCOA6	-0.17	9.59	-2.24	0.02911
MAP3K7	-0.15	8.22	-2.24	0.02915
RAP1B	-0.16	9.61	-2.24	0.02920
RPS10P3	0.27	6.27	2.24	0.02920
TSHZ3	-0.27	7.44	-2.24	0.02922
PFDN4	0.25	5.90	2.24	0.02922
TCF12	-0.20	7.89	-2.24	0.02923
QPCTL	0.20	7.02	2.24	0.02923
MSANTD4	-0.19	6.71	-2.24	0.02923
CIAO1	0.13	10.29	2.24	0.02924
RSRP1	-0.30	10.54	-2.24	0.02925
HMGCL	0.18	8.96	2.24	0.02927
PRDM10	-0.26	6.65	-2.24	0.02927
CCNG2	-0.26	8.57	-2.24	0.02932
OAS2	-0.44	10.27	-2.24	0.02933
RBM10	0.14	10.70	2.24	0.02933
MRPL21	0.19	8.53	2.24	0.02947
RPS6KA3	-0.24	9.50	-2.24	0.02948
KANSL3	-0.15	9.35	-2.24	0.02950
HNRNPA0	0.12	10.88	2.24	0.02959
ADPGK	-0.16	10.30	-2.24	0.02960
ALDH16A1	0.24	9.02	2.24	0.02962
PDPK1	-0.25	8.96	-2.24	0.02967
PPP1CA	0.20	12.69	2.24	0.02967
DHCR7	0.20	7.54	2.24	0.02967
DALRD3	0.16	8.79	2.24	0.02969
IRF6	-0.30	5.22	-2.24	0.02974
SP4	-0.22	6.80	-2.23	0.02977
INTS6L	-0.22	7.84	-2.23	0.02977
NAA25	-0.21	6.71	-2.23	0.02978
FRYL	-0.20	8.58	-2.23	0.02979
AC069218.1	0.30	7.31	2.23	0.02979
DPM2	0.40	10.05	2.23	0.02981
HIVEP1	-0.25	7.84	-2.23	0.02982
CLINT1	-0.28	8.21	-2.23	0.02985
ITGA2B	0.51	10.97	2.23	0.02987
ASXL2	-0.23	8.00	-2.23	0.02988
RAP1AP	-0.30	5.34	-2.23	0.02989
PTGES2	0.19	8.93	2.23	0.02991
DDX17	-0.22	12.27	-2.23	0.02991
SLC31A2	-0.34	9.57	-2.23	0.02997
NEFL	0.36	6.30	2.23	0.02998
SDHAF1	0.26	7.14	2.23	0.03000
AP3M1	-0.16	8.84	-2.23	0.03003

SMARCA2	-0.18	10.23	-2.23	0.03012
SPIB	0.30	8.74	2.23	0.03014
ISY1	0.21	9.08	2.23	0.03014
CLIC5	-0.23	5.83	-2.23	0.03023
RPL7	0.20	13.10	2.23	0.03024
RPL18AP3	0.34	10.10	2.23	0.03026
CCDC93	-0.17	8.72	-2.23	0.03034
MINK1	0.14	11.25	2.23	0.03034
TRBV10-3	0.30	6.45	2.23	0.03036
TXNRD1	-0.19	9.47	-2.23	0.03039
ZNF330	-0.13	8.10	-2.23	0.03044
TMEM141	0.24	7.33	2.22	0.03049
SORL1	-0.37	12.80	-2.22	0.03050
PARVB	0.23	9.34	2.22	0.03052
NEDD1	-0.20	6.63	-2.22	0.03052
DHDDS	0.13	8.36	2.22	0.03058
AC018475.1	0.31	6.23	2.22	0.03060
EFNA4	0.28	7.27	2.22	0.03061
RBFA	0.19	6.75	2.22	0.03066
SERPINB9	-0.22	9.14	-2.22	0.03066
SRGAP2	-0.25	9.44	-2.22	0.03068
DTX1	0.41	8.16	2.22	0.03071
ICAM2	0.20	10.89	2.22	0.03073
BLZF1	-0.22	6.60	-2.22	0.03073
DAP3	0.11	10.23	2.22	0.03078
WDR11-AS1	-0.29	5.74	-2.22	0.03082
CWF19L2	0.27	8.84	2.22	0.03083
TRBV6-1	0.30	7.01	2.22	0.03085
ESF1	0.31	7.67	2.22	0.03086
PLEKHO1	0.19	12.12	2.22	0.03087
NREP	-0.20	6.74	-2.22	0.03087
GCAT	0.45	5.96	2.22	0.03092
PSMC3	0.22	10.13	2.22	0.03096
RAD52	-0.21	6.63	-2.22	0.03100
CACTIN	0.18	8.70	2.22	0.03101
ZFC3H1	-0.22	8.33	-2.22	0.03108
AL162430.2	0.22	5.80	2.22	0.03110
PEAK1	-0.17	7.28	-2.22	0.03111
CCDC167	0.34	7.82	2.22	0.03116
RPIA	0.15	8.74	2.22	0.03118
NBL1	0.35	6.28	2.22	0.03118
SOD1	0.21	10.44	2.21	0.03119
GALNT3	-0.31	6.81	-2.21	0.03124
GPATCH3	0.17	8.47	2.21	0.03125
RPL15	0.20	13.98	2.21	0.03132
STXBP5	-0.28	8.07	-2.21	0.03137
AC079331.1	0.32	5.37	2.21	0.03141
NSUN2	-0.15	9.30	-2.21	0.03145
NDUFB9	0.22	10.24	2.21	0.03148
IPMK	-0.31	7.16	-2.21	0.03151

TMEM201	0.16	6.88	2.21	0.03159
MX2	-0.34	11.88	-2.21	0.03160
TMEM181	-0.20	8.08	-2.21	0.03160
MRPL12	0.26	5.58	2.21	0.03165
RPS4X	0.28	14.28	2.21	0.03173
RN7SL138P	0.32	5.42	2.21	0.03173
TCEAL3	0.18	6.57	2.21	0.03174
CKAP5	-0.16	8.88	-2.21	0.03180
WDR24	0.17	8.44	2.21	0.03182
SF3B2	0.13	12.28	2.21	0.03182
ANKRD28	-0.23	7.30	-2.21	0.03183
ACLY	-0.16	10.40	-2.21	0.03186
RRM2B	-0.25	7.70	-2.21	0.03190
CCDC174	0.12	8.91	2.20	0.03193
ZNF611	0.26	7.43	2.20	0.03194
KDM6A	-0.30	7.80	-2.20	0.03198
TJP3	0.31	6.85	2.20	0.03199
RPL31	0.22	13.88	2.20	0.03204
SUSD6	-0.27	11.20	-2.20	0.03206
KLF10	-0.22	8.28	-2.20	0.03208
TRIM23	-0.23	6.64	-2.20	0.03215
CCR3	-0.48	8.93	-2.20	0.03220
CTSD	0.30	11.87	2.20	0.03226
MRPS7	0.19	9.02	2.20	0.03229
TPGS1	0.27	6.21	2.20	0.03235
AP001505.1	0.26	5.20	2.20	0.03236
QARS1	0.15	11.62	2.20	0.03243
ZFPM1	0.25	7.71	2.20	0.03245
AL161909.1	0.33	8.12	2.20	0.03246
AGBL2	-0.22	5.08	-2.20	0.03252
FH	0.14	8.30	2.20	0.03254
CCDC57	0.18	9.05	2.20	0.03254
NUDT14	0.27	7.04	2.20	0.03255
PIP4P2	-0.30	8.12	-2.20	0.03258
TUBB	0.12	12.02	2.20	0.03263
ANKK1	0.28	5.24	2.20	0.03265
ACTG1	0.17	15.66	2.20	0.03266
CCT3	0.17	11.19	2.19	0.03271
TMEM87B	-0.18	7.61	-2.19	0.03276
MAFB	-0.26	8.88	-2.19	0.03282
RNF25	0.18	8.41	2.19	0.03289
RNF5	0.18	9.04	2.19	0.03290
CCM2	0.16	11.84	2.19	0.03291
BCL7B	0.18	10.25	2.19	0.03293
KPNA3	-0.20	7.54	-2.19	0.03293
ADD1	0.11	12.44	2.19	0.03294
OGT	-0.31	10.70	-2.19	0.03294
FBXL3	-0.25	8.56	-2.19	0.03296
CHST15	-0.31	11.08	-2.19	0.03297
DUS2	0.17	8.68	2.19	0.03298

SLC48A1	0.24	8.56	2.19	0.03301
BLOC1S6	-0.21	8.07	-2.19	0.03303
GIMAP8	-0.21	10.53	-2.19	0.03305
PAXIP1	-0.23	7.02	-2.19	0.03308
BRAT1	0.22	9.61	2.19	0.03321
RNF103	-0.18	7.86	-2.19	0.03322
OGFOD2	0.23	5.09	2.19	0.03322
OGA	-0.21	10.86	-2.19	0.03323
AASDH	-0.17	6.60	-2.19	0.03323
EXOC2	-0.15	8.47	-2.19	0.03323
PPP2R2D	0.12	9.59	2.19	0.03328
ERG28	0.18	8.23	2.19	0.03330
ZNF75D	-0.14	7.53	-2.19	0.03331
NUP98	-0.19	10.59	-2.19	0.03332
SLC35F5	-0.21	7.27	-2.19	0.03335
MFSD1	-0.18	9.52	-2.19	0.03336
AP000437.1	0.40	5.33	2.19	0.03339
AC008038.1	0.30	10.81	2.19	0.03340
FAM86JP	0.24	5.01	2.18	0.03349
GOLPH3	-0.16	9.36	-2.18	0.03350
MAP2K7	0.14	9.86	2.18	0.03350
EHMT2	0.16	10.44	2.18	0.03351
ETHE1	0.23	8.79	2.18	0.03355
ELMSAN1	-0.25	9.06	-2.18	0.03358
AC073210.3	0.35	5.24	2.18	0.03359
FAM162A	0.21	7.81	2.18	0.03360
CD274	-0.36	6.52	-2.18	0.03361
KLF1	0.53	7.70	2.18	0.03364
PLXNC1	-0.35	10.99	-2.18	0.03367
NUMB	-0.30	10.73	-2.18	0.03367
CRIP1	0.21	7.25	2.18	0.03367
TMEM243	0.19	7.99	2.18	0.03370
TNFSF10	-0.33	10.82	-2.18	0.03377
HNRNPF	-0.15	11.37	-2.18	0.03379
ENHO	0.35	6.44	2.18	0.03381
AC040162.3	0.22	5.48	2.18	0.03382
PPM1H	-0.26	5.65	-2.18	0.03393
AC119044.1	0.22	6.92	2.18	0.03393
PPP4R1L	-0.26	7.20	-2.18	0.03397
PAWR	0.24	5.58	2.18	0.03398
DOCK11	-0.20	9.53	-2.18	0.03404
H2AX	0.20	8.10	2.18	0.03406
AC006064.2	-0.25	4.90	-2.18	0.03408
ZNF773	0.22	7.10	2.18	0.03411
Z97989.1	-0.24	5.36	-2.18	0.03413
ECHS1	0.22	9.50	2.18	0.03417
AC078962.2	0.44	6.17	2.18	0.03418
AP006621.5	0.32	5.71	2.18	0.03418
AC004846.2	-0.27	5.58	-2.18	0.03418
MYLIP	-0.22	9.18	-2.18	0.03420

RNF26	0.21	9.17	2.18	0.03421
ICE1	-0.21	8.35	-2.18	0.03421
AC104825.1	-0.20	6.15	-2.18	0.03422
EXOSC5	0.23	8.10	2.18	0.03422
BTN3A1	-0.21	11.38	-2.17	0.03423
AP2A2	0.12	10.24	2.17	0.03432
NEURL2	0.21	5.02	2.17	0.03432
MRPL51	0.20	9.25	2.17	0.03436
C1orf52	-0.12	7.39	-2.17	0.03439
POLR1C	0.21	8.04	2.17	0.03439
PHF1	0.14	10.56	2.17	0.03440
WSB1	-0.24	10.22	-2.17	0.03442
TRAPPC9	0.15	9.90	2.17	0.03443
TUBGCP2	0.13	10.40	2.17	0.03449
MRPL9	0.16	9.16	2.17	0.03449
AC018738.1	0.25	7.33	2.17	0.03452
APPL1	-0.21	7.65	-2.17	0.03455
AC018628.2	0.20	5.18	2.17	0.03462
PLPP1	0.20	6.02	2.17	0.03464
LSM7	0.19	9.59	2.17	0.03464
ZBTB49	-0.15	7.48	-2.17	0.03466
TES	-0.20	9.23	-2.17	0.03467
SAMD3	-0.23	8.09	-2.17	0.03470
ZNF791	0.25	8.81	2.17	0.03472
S100Z	-0.22	6.33	-2.17	0.03472
DONSON	-0.20	5.41	-2.17	0.03473
TIMM17B	0.20	9.33	2.17	0.03480
AC015911.8	0.32	6.05	2.17	0.03489
SUGP1	0.17	9.84	2.17	0.03489
CMTR2	-0.23	7.27	-2.17	0.03490
BTN2A1	-0.21	10.20	-2.17	0.03492
THOP1	0.20	8.16	2.17	0.03492
MYL5	0.22	6.55	2.17	0.03493
SEC23IP	-0.16	8.14	-2.17	0.03493
ZNF579	0.23	6.48	2.17	0.03498
SMARCA4	0.14	10.92	2.17	0.03500
CENPQ	-0.20	5.45	-2.16	0.03506
AC136475.9	-0.52	6.01	-2.16	0.03506
ZNF33A	-0.21	9.13	-2.16	0.03510
ZBTB47	-0.22	7.60	-2.16	0.03512
STARD9	-0.26	6.52	-2.16	0.03516
UQCR11	0.24	8.55	2.16	0.03517
REXO1	0.16	9.38	2.16	0.03520
DTX2	0.20	9.28	2.16	0.03520
RPL15P3	0.27	9.34	2.16	0.03521
FLAD1	0.16	9.04	2.16	0.03521
ARGLU1	-0.22	9.54	-2.16	0.03523
GLI4	0.22	7.81	2.16	0.03524
AP1M1	0.14	11.26	2.16	0.03525
MPI	0.18	9.06	2.16	0.03526

RNPS1	0.14	11.19	2.16	0.03528
TAPT1	-0.15	7.67	-2.16	0.03531
MPHOSPH8	0.26	10.68	2.16	0.03532
ACTG1P9	0.20	5.20	2.16	0.03533
MKLN1-AS	0.20	5.65	2.16	0.03533
SIVA1	0.20	8.90	2.16	0.03536
KMT2D	-0.24	11.15	-2.16	0.03538
BRPF3	-0.24	8.59	-2.16	0.03542
AC069213.3	0.29	5.46	2.16	0.03544
EPC2	-0.22	7.88	-2.16	0.03545
DMXL1	-0.22	6.97	-2.16	0.03547
DENND5B	0.26	6.01	2.16	0.03547
WAC	-0.19	10.59	-2.16	0.03550
OTULINL	-0.17	8.79	-2.16	0.03555
CYB5D2	0.15	7.61	2.16	0.03556
MPG	0.22	8.60	2.16	0.03558
PSMD10	-0.15	7.74	-2.16	0.03561
MBTPS2	-0.21	5.79	-2.16	0.03562
HELZ2	-0.32	11.01	-2.16	0.03562
RPSAP61	0.20	5.51	2.16	0.03562
CTNND1	-0.24	5.34	-2.16	0.03563
ZNF322	-0.23	5.28	-2.16	0.03568
CSNK1E	0.17	8.48	2.16	0.03569
TMEM50A	-0.17	10.52	-2.16	0.03571
LINC01550	0.26	6.88	2.16	0.03572
CARD11	0.16	11.23	2.16	0.03575
GFPT1	-0.18	7.10	-2.16	0.03576
FBXO31	0.17	9.18	2.16	0.03577
NFIA	0.29	7.02	2.16	0.03578
RUNX3	0.26	12.45	2.16	0.03578
FBXO8	-0.17	6.72	-2.16	0.03580
CDK5R1	-0.33	7.65	-2.16	0.03582
TRAV36DV7	0.28	5.41	2.15	0.03584
AC254562.3	0.27	4.95	2.15	0.03588
ELOB	0.26	11.48	2.15	0.03591
LCP2	-0.19	11.69	-2.15	0.03591
AC016739.1	0.32	10.55	2.15	0.03591
SETD1A	0.14	9.75	2.15	0.03592
YIPF2	0.16	7.95	2.15	0.03592
PSTPIP2	-0.39	8.51	-2.15	0.03592
AQR	-0.12	8.86	-2.15	0.03595
IFNAR1	-0.21	10.28	-2.15	0.03599
TESK1	0.15	9.28	2.15	0.03599
STAU1	-0.14	9.84	-2.15	0.03601
COMMD10	-0.17	6.91	-2.15	0.03603
XYLT2	0.13	8.76	2.15	0.03605
SYNE1	-0.19	10.92	-2.15	0.03611
ZNF143	-0.18	7.89	-2.15	0.03612
SNF8	0.18	9.67	2.15	0.03615
B4GAT1	0.22	6.75	2.15	0.03615

PITHD1	0.17	9.46	2.15	0.03618
CHORDC1	-0.21	7.03	-2.15	0.03620
ARMC7	0.17	9.13	2.15	0.03621
RPL36A	0.23	5.67	2.15	0.03621
GCC1	-0.13	9.06	-2.15	0.03622
DDX41	0.14	10.45	2.15	0.03622
INTS1	0.14	10.96	2.15	0.03623
NOB1	0.18	9.73	2.15	0.03624
CD79A	0.42	11.44	2.15	0.03626
NHLRC2	-0.19	6.75	-2.15	0.03627
PHF11	-0.22	8.95	-2.15	0.03634
POLR2J	0.22	9.17	2.15	0.03636
AL139246.5	0.25	6.18	2.15	0.03636
HHLA3	0.25	6.07	2.15	0.03636
STX5	0.17	10.35	2.15	0.03640
HHEX	-0.19	8.76	-2.15	0.03641
POLRMT	0.16	9.84	2.15	0.03642
Z99129.4	-0.22	5.89	-2.15	0.03643
AP2S1	0.23	10.07	2.15	0.03646
SF3B4	0.17	10.39	2.15	0.03649
SENP7	-0.24	7.06	-2.15	0.03650
WDR47	-0.21	6.93	-2.15	0.03654
AURKAIP1	0.21	10.27	2.15	0.03654
ITM2B	-0.27	13.11	-2.15	0.03656
TXNDC11	0.16	9.56	2.15	0.03662
ETFB	0.24	8.76	2.15	0.03662
RPL21P16	0.28	9.76	2.14	0.03667
ARMCX6	0.18	7.12	2.14	0.03669
USO1	-0.17	8.52	-2.14	0.03674
AGPAT5	-0.22	6.93	-2.14	0.03676
EEF1B2P2	0.22	5.47	2.14	0.03677
PPIG	0.28	10.01	2.14	0.03680
NR2C1	-0.24	6.59	-2.14	0.03683
RANBP9	-0.17	8.28	-2.14	0.03684
SMIM7	0.17	8.56	2.14	0.03688
DNAJC17	0.18	8.10	2.14	0.03691
RMDN2	-0.23	5.27	-2.14	0.03695
RSPRY1	-0.15	7.81	-2.14	0.03696
RABGAP1L	-0.15	9.41	-2.14	0.03700
MKRN2	-0.16	8.19	-2.14	0.03700
YTHDC2	-0.19	7.23	-2.14	0.03702
AP2M1	0.16	12.50	2.14	0.03702
POP7	0.18	8.27	2.14	0.03703
AL359962.1	0.35	5.18	2.14	0.03704
AC079250.1	0.31	8.59	2.14	0.03707
TIMM44	0.18	8.55	2.14	0.03713
ZMYM5	-0.22	6.76	-2.14	0.03716
STK4	-0.15	10.70	-2.14	0.03718
NPTN	-0.24	9.54	-2.14	0.03722
REX1BD	0.20	8.61	2.14	0.03723

TM9SF3	-0.14	9.28	-2.14	0.03728
DCLRE1C	0.25	8.98	2.14	0.03729
SCAMP3	0.17	9.77	2.14	0.03742
AC068137.1	0.32	6.27	2.14	0.03745
LINC02245	0.27	6.24	2.14	0.03750
NUP62	0.14	10.43	2.13	0.03755
CDK9	0.11	10.16	2.13	0.03757
CCDC9	0.19	9.01	2.13	0.03758
SNAP23	-0.24	9.44	-2.13	0.03761
CHCHD1	0.20	8.28	2.13	0.03763
IL6R-AS1	0.22	5.05	2.13	0.03767
KCTD21-AS1	0.33	4.91	2.13	0.03774
TAF6	0.15	9.67	2.13	0.03776
GTPBP6	0.17	9.88	2.13	0.03779
RAB5B	-0.16	10.56	-2.13	0.03782
FAM185A	-0.15	5.26	-2.13	0.03783
AC091057.1	-0.24	5.11	-2.13	0.03788
PYCR1	0.28	5.16	2.13	0.03788
IGHV5-10-1	0.65	6.06	2.13	0.03792
CANX	-0.16	10.79	-2.13	0.03792
ZNF326	-0.20	6.55	-2.13	0.03792
FBXL19-AS1	0.22	5.11	2.13	0.03794
DNAJC9	0.15	7.91	2.13	0.03796
LEMD3	-0.21	7.60	-2.13	0.03797
ZNF664	-0.19	7.75	-2.13	0.03798
SMG7	-0.14	10.14	-2.13	0.03802
ZNF212	0.15	8.26	2.13	0.03803
TAGAP	-0.23	11.64	-2.13	0.03805
JAG1	-0.27	5.78	-2.13	0.03808
LTA	0.25	7.61	2.13	0.03813
CLASP1	-0.19	8.72	-2.13	0.03815
EIF2B2	0.15	8.24	2.13	0.03816
THYN1	0.17	8.91	2.13	0.03816
ATP11C	-0.21	6.56	-2.13	0.03818
USP11	0.14	9.89	2.13	0.03818
ZNF624	-0.21	5.33	-2.13	0.03820
MNDA	-0.29	13.55	-2.13	0.03823
WDR33	0.10	9.98	2.13	0.03831
HLA-DQB1	0.41	11.79	2.13	0.03832
EPS8L2	0.24	6.75	2.13	0.03832
TPP1	-0.14	11.63	-2.13	0.03834
SLC5A3	-0.19	6.81	-2.12	0.03839
APEX1	0.16	10.84	2.12	0.03840
TP53TG1	0.21	7.40	2.12	0.03842
NSRP1P1	0.28	5.70	2.12	0.03845
MSN	-0.19	13.64	-2.12	0.03848
AC092944.1	-0.25	5.35	-2.12	0.03852
ACO2	0.13	10.83	2.12	0.03853
NMI	-0.27	9.51	-2.12	0.03862
IDS	-0.24	7.83	-2.12	0.03864

HEIH	0.17	9.70	2.12	0.03867
RPS4XP7	0.28	5.90	2.12	0.03876
ZNF720	-0.16	6.62	-2.12	0.03878
PRKCI	-0.19	6.35	-2.12	0.03881
LYN	-0.28	12.60	-2.12	0.03882
SLC9A6	-0.17	7.39	-2.12	0.03884
MS4A6A	-0.18	10.74	-2.12	0.03885
SDHAF2	0.21	8.31	2.12	0.03885
LCP1	-0.20	14.61	-2.12	0.03885
HDDC2	0.18	8.40	2.12	0.03887
AC099548.2	0.23	5.56	2.12	0.03888
AL160006.1	-0.16	7.25	-2.12	0.03896
UBE2W	-0.22	7.72	-2.12	0.03900
PELI3	0.25	7.69	2.12	0.03901
EEF1B2P3	0.31	8.70	2.12	0.03905
RNF6	-0.22	7.63	-2.12	0.03907
MXD1	-0.38	12.07	-2.12	0.03909
CDPF1	0.18	7.69	2.12	0.03912
AC008764.8	-0.23	5.59	-2.12	0.03915
FAM49B	-0.23	10.53	-2.12	0.03916
PSMD4	0.17	11.17	2.12	0.03922
ACSL3	-0.17	7.99	-2.12	0.03926
AP4S1	0.18	6.81	2.11	0.03935
PGAP3	0.18	9.19	2.11	0.03941
MED19	0.18	7.61	2.11	0.03944
ANKRD54	0.16	8.04	2.11	0.03945
AL109955.1	0.23	5.50	2.11	0.03945
ZC3H7A	-0.17	8.43	-2.11	0.03948
USP9X	-0.18	9.86	-2.11	0.03949
LCORL	-0.16	5.98	-2.11	0.03951
USP32	-0.28	9.60	-2.11	0.03954
PCED1B	0.23	10.31	2.11	0.03954
PRKAR2A	0.18	9.27	2.11	0.03954
THBD	-0.42	8.50	-2.11	0.03955
AC027097.1	0.21	6.72	2.11	0.03957
FRS2	-0.17	6.58	-2.11	0.03960
PTTG1IP	-0.15	11.04	-2.11	0.03969
TECRP1	0.26	7.15	2.11	0.03969
ZFX	-0.24	7.56	-2.11	0.03972
FRS3	0.17	6.83	2.11	0.03974
PLD1	-0.21	6.51	-2.11	0.03974
SCAF8	-0.26	7.90	-2.11	0.03978
VMP1	-0.29	11.66	-2.11	0.03985
AVL9	-0.20	8.45	-2.11	0.03985
EAF1	-0.20	8.00	-2.11	0.03988
CASP2	-0.14	9.26	-2.11	0.03990
FAM133B	0.29	7.54	2.11	0.03991
CPLANE1	-0.20	5.72	-2.11	0.03999
MKI67	-0.28	6.99	-2.11	0.04001
CHD1	-0.22	8.56	-2.11	0.04001

THAP7-AS1	0.23	5.32	2.11	0.04005
SNPH	0.24	7.72	2.11	0.04009
IGLL5	0.44	5.46	2.11	0.04010
SAMM50	0.18	9.47	2.11	0.04010
NDUFS3	0.18	9.26	2.11	0.04012
ASAH1	-0.21	11.17	-2.11	0.04014
MTMR10	-0.20	9.35	-2.11	0.04014
SAC3D1	0.24	6.47	2.11	0.04017
NACA	0.16	12.67	2.10	0.04018
POGZ	-0.16	9.36	-2.10	0.04020
ZNF446	0.16	8.00	2.10	0.04024
ARL15	-0.17	6.92	-2.10	0.04025
NSD3	-0.11	9.89	-2.10	0.04027
ZNF414	0.18	8.00	2.10	0.04028
AL683813.2	0.26	5.63	2.10	0.04030
TSEN15	-0.16	7.42	-2.10	0.04036
UBQLN2	-0.18	9.28	-2.10	0.04042
IL32	0.31	12.26	2.10	0.04051
HBP1	-0.23	9.48	-2.10	0.04053
NOL7	0.15	8.90	2.10	0.04059
LINC02273	0.36	7.89	2.10	0.04063
AC010536.1	0.25	4.96	2.10	0.04067
DNAJB14	-0.20	7.28	-2.10	0.04068
FBXO30	-0.27	6.63	-2.10	0.04074
ZCCHC17	0.16	9.12	2.10	0.04076
LIN7A	-0.30	8.06	-2.10	0.04080
RAB6A	-0.17	8.98	-2.10	0.04080
SCYL2	-0.21	7.83	-2.10	0.04085
AP001324.1	0.29	10.60	2.10	0.04085
CSF2RB	-0.40	11.91	-2.10	0.04087
DOP1A	-0.17	7.45	-2.10	0.04091
C12orf75	-0.26	8.02	-2.10	0.04094
SLC30A5	-0.19	7.27	-2.10	0.04095
AC133435.1	0.22	6.56	2.10	0.04099
FAM102A	0.24	11.35	2.10	0.04108
SYNE2	-0.18	11.27	-2.10	0.04108
TRIM24	-0.17	7.54	-2.09	0.04110
PPP1R7	0.16	10.30	2.09	0.04111
LGALS1	0.33	11.03	2.09	0.04111
USP28	-0.19	8.17	-2.09	0.04116
MTPN	-0.19	10.50	-2.09	0.04119
JMJD4	0.19	8.20	2.09	0.04124
ITSN2	-0.17	9.92	-2.09	0.04124
TXNL4A	0.16	9.52	2.09	0.04126
PPP2R5C	-0.13	10.86	-2.09	0.04129
CAPZA1	-0.20	10.79	-2.09	0.04130
UBE4A	-0.20	8.55	-2.09	0.04132
KRT18	0.24	6.27	2.09	0.04138
SGK3	-0.17	7.90	-2.09	0.04138
EIF4G2	-0.15	12.30	-2.09	0.04139

HSD17B10	0.22	9.36	2.09	0.04141
DTHD1	-0.20	5.25	-2.09	0.04142
FBXO44	0.20	9.05	2.09	0.04144
PMF1	0.18	8.26	2.09	0.04145
CD36	-0.28	9.83	-2.09	0.04150
SEC22B	-0.17	8.13	-2.09	0.04159
RPS13P2	0.27	9.23	2.09	0.04159
KRCC1	-0.20	7.80	-2.09	0.04166
ADCY4	-0.28	7.43	-2.09	0.04168
LAMTOR2	0.24	9.40	2.09	0.04171
AC092119.2	-0.22	5.13	-2.09	0.04174
THUMPD3	-0.17	7.54	-2.09	0.04177
ZNF763	0.18	6.39	2.09	0.04179
PPP3R1	-0.16	9.52	-2.09	0.04180
AGPS	-0.17	7.47	-2.09	0.04181
AL035398.1	0.26	5.94	2.09	0.04182
RPL13AP5	0.30	11.45	2.09	0.04186
SAPCD1-AS1	0.27	4.98	2.09	0.04188
SLC25A42	0.15	8.51	2.09	0.04188
RPL21P119	0.23	7.93	2.09	0.04188
ANXA6	0.19	13.16	2.09	0.04191
VPREB3	0.31	7.35	2.09	0.04194
DAZAP1	0.13	10.59	2.09	0.04194
RARG	0.20	8.64	2.09	0.04196
ZNF567	-0.16	5.71	-2.09	0.04198
RRP9	0.22	8.01	2.09	0.04200
PMS1	-0.18	6.23	-2.09	0.04201
NBPF26	-0.29	7.95	-2.08	0.04203
CCDC34	0.32	6.27	2.08	0.04206
NOP14-AS1	0.14	7.76	2.08	0.04215
PIP5K1C	0.14	9.89	2.08	0.04216
ACTR10	-0.13	8.24	-2.08	0.04216
FBXW2	-0.12	9.45	-2.08	0.04218
ESYT2	-0.15	10.09	-2.08	0.04220
KLHL18	-0.15	8.77	-2.08	0.04229
SLC29A2	0.23	6.58	2.08	0.04233
AL034397.3	-0.38	10.59	-2.08	0.04234
LYRM7	0.21	7.07	2.08	0.04236
KIAA0319	-0.27	6.07	-2.08	0.04239
SIGMAR1	0.14	8.91	2.08	0.04243
TRAFD1	-0.24	10.75	-2.08	0.04249
ZSWIM9	0.20	7.06	2.08	0.04249
CD93	-0.31	11.09	-2.08	0.04251
CCR1	-0.34	10.07	-2.08	0.04254
INTS11	0.15	10.75	2.08	0.04254
LINC02812	0.32	5.69	2.08	0.04256
SPART	-0.23	8.12	-2.08	0.04259
NT5C3AP1	-0.26	5.18	-2.08	0.04262
BCAS4	0.25	8.07	2.08	0.04262
RASA2	-0.30	7.53	-2.08	0.04263

S100A5	0.19	5.01	2.08	0.04264
DAB2	-0.23	7.59	-2.08	0.04268
AC243919.1	0.30	10.43	2.08	0.04268
ETNK1	-0.19	8.05	-2.08	0.04272
SYTL2	-0.24	7.55	-2.08	0.04273
ZNF815P	0.23	6.97	2.08	0.04275
MED14	-0.18	8.18	-2.08	0.04277
ZNF296	0.26	7.71	2.08	0.04277
LMF2	0.17	10.26	2.08	0.04281
CLK1	-0.25	9.93	-2.08	0.04282
WDR20	-0.15	8.09	-2.08	0.04284
MTMR3	-0.25	10.89	-2.08	0.04285
MOB4	-0.19	6.62	-2.08	0.04288
RAB12	-0.18	7.18	-2.08	0.04290
TLR1	-0.28	9.42	-2.08	0.04291
SMIM13	-0.17	6.01	-2.08	0.04292
MDM4	-0.20	8.88	-2.08	0.04296
OSBP	-0.14	9.05	-2.07	0.04298
C9orf78	0.52	12.48	2.07	0.04299
BBS10	-0.24	5.78	-2.07	0.04304
MAPRE1	-0.15	10.21	-2.07	0.04306
RPS12P23	0.20	5.06	2.07	0.04308
SLC2A11	0.20	6.77	2.07	0.04308
ANXA11	0.19	13.06	2.07	0.04312
MAGED2	0.14	10.06	2.07	0.04312
TBCB	0.17	10.18	2.07	0.04315
PAK1	-0.20	11.41	-2.07	0.04316
ALKBH2	0.21	7.13	2.07	0.04320
EFCAB14	-0.15	9.97	-2.07	0.04320
MTA2	0.13	11.37	2.07	0.04320
SUDS3	-0.13	8.90	-2.07	0.04320
ATP11B	-0.26	8.82	-2.07	0.04321
DNAJA3	0.15	8.98	2.07	0.04321
PRKAA1	-0.20	7.91	-2.07	0.04322
AZI2	-0.16	8.08	-2.07	0.04328
TTC13	-0.21	7.71	-2.07	0.04328
EP300	-0.31	10.35	-2.07	0.04331
ARL6IP6	-0.20	7.27	-2.07	0.04332
ASCC2	0.35	12.05	2.07	0.04333
NXT1	0.20	7.80	2.07	0.04337
PTGER2	-0.14	8.09	-2.07	0.04340
UHRF2	-0.18	7.83	-2.07	0.04340
TRAV3	0.30	6.39	2.07	0.04341
RIPK2	-0.18	7.66	-2.07	0.04341
CTSF	0.22	7.55	2.07	0.04343
URGCP	0.17	8.65	2.07	0.04353
ACTR3	-0.20	10.99	-2.07	0.04354
BLOC1S4	0.15	8.39	2.07	0.04359
SDCBPP3	-0.39	6.27	-2.07	0.04360
SERTAD1	0.19	8.01	2.07	0.04362

TRIP12	-0.18	10.59	-2.07	0.04371
TRERF1	-0.17	8.77	-2.07	0.04373
PTCRA	0.31	7.26	2.07	0.04377
TMED7	-0.20	7.33	-2.07	0.04377
PTGES3P1	-0.26	7.05	-2.07	0.04381
NTAN1	0.19	8.76	2.07	0.04382
ZNF667-AS1	-0.26	5.53	-2.07	0.04383
GGT7	0.23	8.26	2.07	0.04385
LEPR	-0.22	5.32	-2.07	0.04386
TFG	-0.14	8.73	-2.06	0.04396
BTN3A2	0.24	11.87	2.06	0.04398
AP003486.1	-0.22	6.68	-2.06	0.04400
PSENEN	0.24	8.36	2.06	0.04401
PTPA	0.18	10.68	2.06	0.04401
WFS1	0.19	6.71	2.06	0.04402
CA2	-0.36	7.90	-2.06	0.04403
MPND	0.20	8.65	2.06	0.04409
TTC37	-0.21	7.98	-2.06	0.04411
LILRA1	-0.22	10.56	-2.06	0.04413
COMMD4	0.20	8.53	2.06	0.04421
ZNF865	0.19	9.47	2.06	0.04425
RGS18	-0.34	9.16	-2.06	0.04440
MOSPD1	-0.23	5.98	-2.06	0.04442
ZNF25	-0.20	6.20	-2.06	0.04444
NFKBIB	0.19	9.41	2.06	0.04446
TRA2A	-0.14	9.37	-2.06	0.04451
MON1A	0.19	7.61	2.06	0.04454
PHF23	0.14	10.19	2.06	0.04456
TRAF6	-0.13	7.94	-2.06	0.04463
SAMD1	0.23	8.57	2.06	0.04466
NAPA-AS1	0.20	5.60	2.06	0.04466
RMND5A	-0.18	9.33	-2.06	0.04467
SERPINH1	0.18	7.38	2.06	0.04470
COG3	-0.18	7.47	-2.06	0.04474
CYSLTR2	-0.28	6.45	-2.06	0.04476
SLC3A2	0.15	10.53	2.06	0.04483
LBH	0.19	12.00	2.06	0.04484
LDOC1	0.25	7.15	2.06	0.04488
SFMBT2	-0.25	7.66	-2.06	0.04493
UROD	0.15	9.34	2.05	0.04496
PLBD1-AS1	-0.28	5.98	-2.05	0.04501
ZSCAN25	-0.14	7.75	-2.05	0.04502
AMY2B	-0.29	6.96	-2.05	0.04503
THRA	0.17	9.30	2.05	0.04512
RCHY1	-0.18	7.07	-2.05	0.04513
HTATSF1P2	-0.47	5.25	-2.05	0.04514
ABCB9	0.25	5.82	2.05	0.04517
MTMR14	0.14	10.93	2.05	0.04520
VRK3	0.15	10.54	2.05	0.04526
NINJ2	0.30	8.82	2.05	0.04530

PMM2	-0.20	6.72	-2.05	0.04531
AL356273.6	0.39	6.88	2.05	0.04532
KLHDC3	0.14	10.11	2.05	0.04534
SIGLEC1	-0.69	7.68	-2.05	0.04535
STOML2	0.17	9.36	2.05	0.04536
PIGU	0.18	7.79	2.05	0.04545
MORF4L2	-0.11	8.65	-2.05	0.04547
EIF4EBP1	0.26	8.59	2.05	0.04550
PRDX5	0.27	11.12	2.05	0.04551
NUDCD3	0.12	9.97	2.05	0.04558
PSMD9	0.17	7.11	2.05	0.04560
LINC01176	-0.26	5.74	-2.05	0.04560
METTL14	-0.18	7.21	-2.05	0.04568
ENPP4	-0.24	6.31	-2.05	0.04569
AC005262.1	0.28	5.56	2.05	0.04569
MCTS1	0.13	8.06	2.05	0.04570
RECK	-0.21	6.51	-2.05	0.04572
TMEM147	0.21	8.77	2.05	0.04577
GOLGA4	-0.15	8.82	-2.05	0.04577
HNRNPH2	-0.22	9.44	-2.05	0.04578
PIANP	0.28	5.08	2.05	0.04580
LRRC37A3	-0.21	5.48	-2.05	0.04585
MYC	0.21	10.26	2.05	0.04586
BLOC1S1	0.24	7.57	2.05	0.04586
TTF1	0.23	8.54	2.05	0.04586
SS18	-0.13	8.43	-2.05	0.04589
CCND1	0.20	5.30	2.04	0.04601
TRPM7	-0.18	7.61	-2.04	0.04601
SMG1P7	-0.22	6.00	-2.04	0.04601
METAP2	0.19	9.14	2.04	0.04603
MAP2K4	-0.20	8.46	-2.04	0.04603
RIT1	-0.19	8.79	-2.04	0.04604
AC022762.2	-0.26	5.26	-2.04	0.04607
ZNF436	-0.16	6.62	-2.04	0.04609
SLC35B3	-0.15	6.79	-2.04	0.04611
MEF2A	-0.25	8.80	-2.04	0.04618
PTBP2	-0.20	6.00	-2.04	0.04619
HERPUD2	-0.14	9.26	-2.04	0.04622
AL355802.1	0.20	6.11	2.04	0.04625
SMDT1	0.32	9.52	2.04	0.04629
ARL4C	0.16	11.39	2.04	0.04634
GNPTAB	-0.18	8.74	-2.04	0.04639
CXCR5	0.27	9.48	2.04	0.04641
TMED3	0.19	9.13	2.04	0.04644
ZNF45	-0.16	6.94	-2.04	0.04645
AC008813.1	0.22	5.62	2.04	0.04645
COQ7	-0.17	6.82	-2.04	0.04648
AC093495.1	-0.40	6.66	-2.04	0.04652
PAF1	0.16	9.91	2.04	0.04655
ARHGAP26	-0.30	10.79	-2.04	0.04655

SLC38A2	-0.21	9.07	-2.04	0.04657
CDK19	-0.19	8.21	-2.04	0.04657
SPTLC2	-0.20	10.07	-2.04	0.04662
DSTYK	0.18	8.89	2.04	0.04662
ATP7A	-0.24	6.60	-2.04	0.04664
RCOR3	-0.18	8.10	-2.04	0.04666
ANO6	-0.23	8.52	-2.04	0.04667
HINT1	0.21	10.32	2.04	0.04668
ZNF697	-0.22	5.25	-2.04	0.04669
AC096733.3	0.23	5.17	2.04	0.04670
MS4A14	-0.30	7.06	-2.04	0.04671
GLE1	-0.11	9.22	-2.04	0.04672
CHPF	0.25	7.15	2.04	0.04672
PAIP2B	0.19	6.10	2.04	0.04674
PSKH1	0.11	9.33	2.04	0.04676
BTBD10	-0.21	8.23	-2.04	0.04677
CDS2	-0.12	10.19	-2.04	0.04683
TRBJ2-3	0.20	7.06	2.04	0.04686
TMEM86B	0.35	7.74	2.04	0.04687
MIR646HG	-0.32	6.80	-2.04	0.04687
GPX1	0.35	12.60	2.04	0.04687
WDR81	0.13	10.06	2.04	0.04690
ZNF124	-0.20	5.56	-2.04	0.04695
TIGD5	0.20	7.38	2.04	0.04697
ZNF532	-0.22	6.53	-2.03	0.04700
STARD3NL	-0.16	7.71	-2.03	0.04703
PDGFD	-0.26	5.91	-2.03	0.04705
PPP1R14B	0.23	8.30	2.03	0.04708
ZNF607	0.17	6.42	2.03	0.04713
DFFA	0.21	8.78	2.03	0.04715
KIF2C	0.25	5.37	2.03	0.04715
FYCO1	-0.18	8.21	-2.03	0.04718
RAB15	-0.30	6.12	-2.03	0.04718
PRAF2	0.19	8.17	2.03	0.04719
DUSP23	0.22	8.57	2.03	0.04719
MSL3	-0.25	10.01	-2.03	0.04720
C1orf109	-0.21	6.54	-2.03	0.04721
NUP43	-0.14	7.84	-2.03	0.04724
NAA80	0.19	7.67	2.03	0.04725
AC004865.2	0.18	6.26	2.03	0.04727
NBPF14	-0.23	8.48	-2.03	0.04734
OSBPL3	-0.18	7.57	-2.03	0.04735
CCT8P1	-0.22	6.51	-2.03	0.04735
SUPT5H	0.12	11.49	2.03	0.04736
AC120053.1	-0.19	5.20	-2.03	0.04736
RNPC3	-0.22	6.76	-2.03	0.04736
GPR174	-0.22	7.57	-2.03	0.04736
CAVIN2	-0.33	9.47	-2.03	0.04738
ZNF653	0.19	7.12	2.03	0.04738
HUWE1	-0.13	11.28	-2.03	0.04739

KRBA1	0.20	7.38	2.03	0.04746
TRGV4	-0.34	5.14	-2.03	0.04748
CD99	0.21	11.83	2.03	0.04751
IL1R1	-0.42	7.24	-2.03	0.04751
INSIG2	-0.15	7.03	-2.03	0.04751
TNFRSF4	0.27	6.19	2.03	0.04751
SBF2	-0.25	7.77	-2.03	0.04753
CASP3	-0.15	8.24	-2.03	0.04758
ZNF606	-0.23	6.43	-2.03	0.04760
SMURF2	-0.14	7.75	-2.03	0.04763
APBA2	0.22	8.91	2.03	0.04763
RPL6	0.20	13.99	2.03	0.04766
UVSSA	-0.32	7.97	-2.03	0.04770
MBOAT1	-0.18	8.61	-2.03	0.04777
C16orf86	0.21	6.23	2.03	0.04777
EML3	0.14	10.77	2.03	0.04779
CAPN2	-0.16	11.12	-2.03	0.04779
EPB41L4A-AS1	0.18	7.96	2.03	0.04781
LILRA4	0.41	7.33	2.03	0.04783
RANGAP1	0.14	10.72	2.03	0.04785
PROC	0.27	5.27	2.03	0.04786
TADA3	0.14	10.94	2.03	0.04787
CSTF1	0.11	8.72	2.03	0.04788
ALDH1A1	-0.26	8.01	-2.03	0.04791
MBLAC1	0.21	5.15	2.03	0.04794
GNA13	-0.23	9.44	-2.03	0.04796
GPR141	-0.38	6.10	-2.03	0.04796
PPP1R37	0.17	7.91	2.03	0.04798
ZNF511	0.18	7.36	2.03	0.04798
UVRAG	-0.14	8.62	-2.03	0.04798
RAB1A	-0.18	9.33	-2.03	0.04801
PDSS1	-0.22	5.68	-2.02	0.04812
ANXA4	-0.15	8.45	-2.02	0.04814
VPS37B	0.18	10.00	2.02	0.04816
RSL1D1	0.16	10.94	2.02	0.04819
EIF1	0.16	13.11	2.02	0.04819
NAGLU	0.16	8.44	2.02	0.04826
SERPINB8	-0.18	7.80	-2.02	0.04828
THRAP3	-0.11	10.69	-2.02	0.04829
ZCCHC10	-0.14	6.88	-2.02	0.04832
DHRS3	0.22	9.17	2.02	0.04835
DMAP1	0.15	9.81	2.02	0.04837
GOLGA7	-0.16	9.53	-2.02	0.04838
MRPL52	0.18	8.70	2.02	0.04839
TRBV7-6	0.24	6.70	2.02	0.04839
PDZD8	-0.27	7.63	-2.02	0.04847
AP001086.1	0.24	5.16	2.02	0.04848
PI4K2A	0.14	8.33	2.02	0.04849
AC004884.1	0.23	5.82	2.02	0.04849
SNAI3	0.19	8.21	2.02	0.04853

ACADS	0.16	8.46	2.02	0.04855
IRF1-AS1	-0.24	8.25	-2.02	0.04856
SMCR8	-0.13	10.16	-2.02	0.04861
ZNF239	0.21	5.52	2.02	0.04861
RPS3AP49	0.22	5.50	2.02	0.04864
CEP162	-0.16	6.33	-2.02	0.04865
ARPP19	-0.14	8.94	-2.02	0.04871
ID3	0.24	8.11	2.02	0.04872
EXOC6	-0.25	7.50	-2.02	0.04872
BACE2	0.25	7.13	2.02	0.04873
C7orf26	0.13	9.64	2.02	0.04876
PPP1R21	-0.15	8.62	-2.02	0.04877
NAPEPLD	-0.19	6.21	-2.02	0.04880
CCDC107	0.24	8.60	2.02	0.04881
KDF1	0.23	5.73	2.02	0.04881
LONP1	0.17	9.90	2.02	0.04890
EVI5L	0.16	8.12	2.02	0.04898
TRBV30	0.36	6.61	2.02	0.04904
IL13RA1	-0.30	9.99	-2.02	0.04908
ERCC1	0.15	9.68	2.02	0.04909
MAP3K7CL	-0.33	8.65	-2.02	0.04909
MRPL20-AS1	0.14	8.73	2.01	0.04913
UBXN2A	0.18	7.00	2.01	0.04914
KIF28P	-0.33	6.05	-2.01	0.04917
TRBV28	0.47	6.94	2.01	0.04922
HDDC3	0.16	8.46	2.01	0.04923
C1orf112	-0.17	5.78	-2.01	0.04925
IGLC3	0.50	10.64	2.01	0.04927
PAK4	0.19	7.50	2.01	0.04931
RPS7	0.20	12.65	2.01	0.04931
AC138466.2	-0.26	5.62	-2.01	0.04931
SLC25A13	-0.17	6.43	-2.01	0.04933
SCYL1	0.14	10.55	2.01	0.04935
CD58	-0.24	7.49	-2.01	0.04939
XAF1	-0.43	9.81	-2.01	0.04941
MCTP2	-0.39	9.09	-2.01	0.04943
IFT140	0.16	6.78	2.01	0.04946
CLDN7	-0.25	5.43	-2.01	0.04952
MYCBP2	-0.20	9.66	-2.01	0.04954
LAMP3	-0.47	5.33	-2.01	0.04955
HP1BP3	-0.20	10.01	-2.01	0.04955
CEP131	0.17	7.36	2.01	0.04959
PDRG1	0.17	8.31	2.01	0.04962
ZNF41	-0.23	6.66	-2.01	0.04970
IFNGR1	-0.25	10.63	-2.01	0.04970
L3MBTL4	-0.22	5.13	-2.01	0.04972
TMEM204	0.27	9.07	2.01	0.04979
BX255925.3	0.23	8.08	2.01	0.04981
B4GALT3	0.15	10.21	2.01	0.04982
RHOU	-0.17	7.50	-2.01	0.04986

FAM86B3P	-0.27	5.29	-2.01	0.04994
AC012368.1	-0.40	9.87	-2.01	0.04997
GAPDH	0.21	14.71	2.01	0.04998
LAMTOR3	-0.21	7.79	-2.01	0.04999
KLF4	-0.22	8.47	-2.01	0.05003
TSPAN33	0.24	9.83	2.01	0.05011
UBE2A	-0.12	8.91	-2.01	0.05011
AC026979.2	0.27	5.97	2.01	0.05012
RAB10	-0.14	9.42	-2.01	0.05013
AP005431.2	0.21	5.84	2.01	0.05019
FIZ1	0.15	7.89	2.01	0.05020
SNHG19	0.25	6.19	2.01	0.05020
NFIL3	-0.31	9.27	-2.00	0.05021
CCDC149	-0.21	7.65	-2.00	0.05021
SENP2	-0.16	8.10	-2.00	0.05022
RGS1	-0.24	5.31	-2.00	0.05027
ELK3	-0.17	8.65	-2.00	0.05031
LINC01934	0.24	6.38	2.00	0.05037
THEM6	0.19	7.50	2.00	0.05041
UTP25	-0.19	7.50	-2.00	0.05051
TAF5L	-0.13	7.99	-2.00	0.05056
SLC25A43	-0.20	6.37	-2.00	0.05057
ATPAF2	0.19	7.81	2.00	0.05062
LRRC6	-0.34	6.41	-2.00	0.05068
RENBP	0.18	9.10	2.00	0.05069
CLEC2B	-0.21	8.98	-2.00	0.05072
CNNM4	-0.16	7.61	-2.00	0.05079
PHRF1	0.13	9.81	2.00	0.05080
CPPED1	-0.25	11.96	-2.00	0.05080
AC008677.2	0.29	8.36	2.00	0.05093
CLEC12B	-0.40	5.61	-2.00	0.05093
AC079140.2	0.36	6.86	2.00	0.05094
MPZL2	-0.27	6.15	-2.00	0.05095
ATXN7L3B	-0.13	9.77	-2.00	0.05095
CPED1	-0.22	5.61	-2.00	0.05105
CDK8	-0.16	6.86	-2.00	0.05105
FAF1	0.15	8.59	2.00	0.05106
RPL21P28	0.29	8.80	2.00	0.05109
TRBV14	0.34	6.02	2.00	0.05117
FUOM	0.24	7.07	2.00	0.05119
RPL24P2	0.24	6.90	2.00	0.05120
MTMR12	-0.16	8.20	-2.00	0.05121
TRAK1	-0.16	9.24	-2.00	0.05123
C14orf132	0.25	6.10	2.00	0.05125
DESI2	-0.12	7.74	-2.00	0.05126
USP1	-0.16	8.00	-2.00	0.05128
KCNK6	0.26	9.64	1.99	0.05136
SMIM4	0.21	6.83	1.99	0.05139
KNSTRN	-0.19	6.64	-1.99	0.05141
AL604028.1	0.23	5.38	1.99	0.05143

AP002381.2	-0.27	6.73	-1.99	0.05148
MED31	-0.18	6.81	-1.99	0.05148
BIRC2	-0.19	8.69	-1.99	0.05150
DUSP12	0.15	7.31	1.99	0.05150
COPB1	-0.16	9.08	-1.99	0.05158
TNFRSF14	0.16	11.97	1.99	0.05169
ABCF3	0.13	9.98	1.99	0.05178
CTDSPL2	-0.17	7.28	-1.99	0.05183
AACS	0.15	7.81	1.99	0.05184
FAM110A	0.23	9.71	1.99	0.05186
ATG3	-0.16	9.89	-1.99	0.05187
BMP6	-0.29	6.66	-1.99	0.05193
RALY	0.15	11.67	1.99	0.05195
ANKEF1	-0.21	5.69	-1.99	0.05196
ARL8B	-0.14	8.57	-1.99	0.05197
MRPS5	0.13	9.14	1.99	0.05199
PPAT	-0.22	5.81	-1.99	0.05207
BAG4	-0.15	6.96	-1.99	0.05207
ZNF621	-0.16	7.34	-1.99	0.05212
HDAC9	-0.27	6.51	-1.99	0.05216
SLBP	-0.14	8.68	-1.99	0.05218
PPIL4	-0.18	7.14	-1.99	0.05219
SLC35A5	-0.18	7.21	-1.99	0.05222
ZBTB26	-0.21	5.80	-1.99	0.05228
KNTC1	-0.22	6.50	-1.99	0.05232
TLE4	-0.19	10.20	-1.99	0.05242
ACSL1	-0.47	12.03	-1.99	0.05243
QRICH1	-0.12	9.84	-1.99	0.05244
SNHG25	0.33	6.47	1.98	0.05249
TNRC6C	0.21	9.44	1.98	0.05250
DARS1	0.24	9.11	1.98	0.05252
MIEN1	0.17	9.26	1.98	0.05255
CCDC7	-0.20	5.36	-1.98	0.05255
NEK8	0.24	7.70	1.98	0.05263
LRMP	-0.18	10.11	-1.98	0.05272
NPC1	-0.15	9.29	-1.98	0.05277
CPOX	-0.20	6.68	-1.98	0.05280
SPRED2	-0.23	5.49	-1.98	0.05280
SERPING1	-0.58	8.50	-1.98	0.05284
PPP4R1	-0.26	10.09	-1.98	0.05284
ARFGAP3	-0.18	8.63	-1.98	0.05287
DLEU1	0.19	5.39	1.98	0.05292
MORF4L1	-0.12	10.75	-1.98	0.05293
ERN1	-0.23	9.72	-1.98	0.05302
APEH	0.13	10.87	1.98	0.05303
WWP1	-0.21	7.91	-1.98	0.05304
TCF4	0.26	7.49	1.98	0.05306
CASD1	-0.23	6.43	-1.98	0.05307
CELF2	-0.19	11.50	-1.98	0.05308
SCAMP2	0.14	11.54	1.98	0.05311

SUCLG2	-0.19	8.22	-1.98	0.05316
STAT1	-0.37	11.26	-1.98	0.05317
SSR1	-0.14	10.01	-1.98	0.05318
ABCB1	-0.18	7.47	-1.98	0.05320
KDM3B	-0.20	10.32	-1.98	0.05321
SMIM15	-0.16	7.05	-1.98	0.05322
GBP1	-0.46	9.15	-1.98	0.05324
ZNF430	0.14	7.48	1.98	0.05327
NLRC4	-0.22	9.10	-1.98	0.05330
BANF1	0.23	10.25	1.98	0.05332
INO80B	0.21	4.99	1.98	0.05333
AC093012.1	-0.18	9.42	-1.98	0.05334
CNN2P4	-0.32	6.14	-1.98	0.05336
AC107956.1	0.25	6.77	1.98	0.05343
AHCTF1	-0.21	8.96	-1.98	0.05343
CREBZF	-0.24	7.90	-1.98	0.05345
WDR76	-0.17	6.23	-1.98	0.05345
RBM22	-0.11	9.96	-1.98	0.05348
NTHL1	0.21	6.99	1.98	0.05350
CLEC4A	-0.20	5.94	-1.98	0.05351
PLEKHF2	-0.20	7.83	-1.98	0.05351
ADD3	-0.24	9.78	-1.98	0.05358
AIDA	-0.20	7.18	-1.98	0.05361
LRIF1	-0.17	7.19	-1.98	0.05361
RPL35P2	0.29	8.03	1.97	0.05366
PUM1	-0.17	9.24	-1.97	0.05369
AC021723.1	0.20	5.90	1.97	0.05371
BRK1	0.16	10.64	1.97	0.05378
C19orf47	0.16	7.94	1.97	0.05379
PPBP	0.43	11.07	1.97	0.05382
LGMN	0.21	6.91	1.97	0.05382
HAL	-0.36	9.44	-1.97	0.05385
PATZ1	0.13	9.45	1.97	0.05385
HDGF	0.19	11.65	1.97	0.05387
LZTS2	0.15	8.09	1.97	0.05388
SPPL2B	0.20	8.75	1.97	0.05390
PGLS	0.20	10.77	1.97	0.05394
AC002558.1	0.26	5.22	1.97	0.05394
PEX16	0.16	9.26	1.97	0.05394
NUCB1	0.18	12.50	1.97	0.05395
ZNF367	-0.18	5.45	-1.97	0.05399
PHB2P1	0.17	5.07	1.97	0.05399
VPS54	-0.18	7.20	-1.97	0.05401
DPYSL2	-0.18	9.68	-1.97	0.05402
RPSA	0.23	14.13	1.97	0.05407
BTF3P9	0.18	5.51	1.97	0.05411
NSMF	0.17	9.14	1.97	0.05415
RANBP2	-0.16	8.95	-1.97	0.05419
DOHH	0.20	6.57	1.97	0.05420
SAMD14	0.44	6.46	1.97	0.05432

XPNPEP1	-0.11	8.93	-1.97	0.05437
TOP2A	0.32	7.48	1.97	0.05439
FER	-0.16	5.90	-1.97	0.05441
RPS2P7	0.25	7.44	1.97	0.05444
NEDD9	-0.25	10.01	-1.97	0.05445
LRRK1	-0.19	8.37	-1.97	0.05451
CD3D	0.26	11.24	1.97	0.05452
TMEM177	0.18	6.15	1.97	0.05452
RPL37P6	0.25	7.36	1.97	0.05452
NUBPL	-0.21	5.45	-1.97	0.05455
CD3EAP	0.19	6.11	1.97	0.05455
AZIN1-AS1	-0.26	6.50	-1.97	0.05455
AC105285.1	-0.18	5.12	-1.97	0.05455
HSCB	0.18	6.66	1.97	0.05456
IGHV3-15	0.44	7.27	1.97	0.05458
NRDE2	0.16	8.79	1.97	0.05459
AL122020.1	0.27	7.39	1.97	0.05459
APOOL	-0.18	6.16	-1.97	0.05467
SOWAHC	-0.23	5.42	-1.97	0.05468
TMEM40	0.27	7.67	1.97	0.05469
AC027575.3	0.21	5.24	1.97	0.05469
TMEM81	0.18	6.31	1.97	0.05471
EIF3F	0.18	11.92	1.97	0.05473
STARD8	-0.21	7.08	-1.97	0.05474
ENOX2	-0.17	6.91	-1.97	0.05477
ZNF181	-0.21	6.15	-1.96	0.05480
SERAC1	-0.17	6.22	-1.96	0.05482
AC093014.1	0.22	5.33	1.96	0.05483
SOCS6	-0.16	5.45	-1.96	0.05484
FBXO10	0.23	6.26	1.96	0.05485
RREB1	-0.17	8.72	-1.96	0.05487
SLC43A1	0.16	7.86	1.96	0.05488
ENDOV	0.18	7.19	1.96	0.05492
ZNF570	0.20	6.44	1.96	0.05493
AL080243.4	0.31	8.33	1.96	0.05494
RPS13	0.19	12.50	1.96	0.05495
IMP4	0.13	9.64	1.96	0.05501
TFCP2	-0.10	9.08	-1.96	0.05503
LEPROT	-0.17	8.73	-1.96	0.05503
PDCD6	-0.32	6.25	-1.96	0.05505
TRAC	0.26	12.95	1.96	0.05508
DHX9	-0.13	10.39	-1.96	0.05510
HSP90AB4P	0.24	5.31	1.96	0.05511
RPL23AP65	0.27	7.70	1.96	0.05515
PPFIA3	0.22	5.79	1.96	0.05515
RAB33B	-0.25	6.95	-1.96	0.05516
EIF5A	0.17	11.66	1.96	0.05543
RPL21P3	0.32	6.86	1.96	0.05546
ZNF500	0.17	7.89	1.96	0.05551
MARF1	-0.22	10.59	-1.96	0.05553

RUBCN	-0.13	9.77	-1.96	0.05557
PTRH1	0.25	6.67	1.96	0.05563
BIK	0.23	5.55	1.96	0.05564
KEAP1	0.14	9.93	1.96	0.05567
MIRLET7A1HG	-0.23	6.12	-1.96	0.05567
AP001496.2	0.16	5.07	1.96	0.05573
RPL23AP74	0.21	7.00	1.96	0.05574
ZBTB34	-0.21	8.55	-1.96	0.05577
ZBTB17	0.13	9.46	1.96	0.05578
PFN1	0.22	14.52	1.96	0.05581
SMC5	-0.16	7.48	-1.96	0.05583
PRCC	0.13	10.05	1.96	0.05588
GK-IT1	0.42	6.05	1.96	0.05591
ABHD13	-0.18	7.16	-1.96	0.05591
ZC3H10	0.12	8.07	1.96	0.05592
TMPO	-0.19	8.49	-1.96	0.05593
BBS2	-0.16	8.42	-1.96	0.05596
POT1	-0.15	6.90	-1.95	0.05600
TRIM14	-0.19	9.48	-1.95	0.05601
MAN2A1	-0.17	8.06	-1.95	0.05604
KLHL22	0.12	9.01	1.95	0.05607
RUNX2	-0.23	8.08	-1.95	0.05607
RABGEF1	0.22	5.39	1.95	0.05610
MGME1	-0.16	7.73	-1.95	0.05613
IDE	-0.16	7.30	-1.95	0.05625
ZNF350	-0.13	7.83	-1.95	0.05626
LAPTM4A	-0.14	10.14	-1.95	0.05627
BTF3	0.16	12.15	1.95	0.05633
IKZF2	-0.21	6.11	-1.95	0.05634
RPS27P14	0.19	5.07	1.95	0.05636
CGRRF1	-0.17	6.42	-1.95	0.05638
C11orf1	0.22	6.69	1.95	0.05640
AC024075.1	0.27	5.76	1.95	0.05646
GATM	-0.28	5.45	-1.95	0.05650
CRTC2	0.13	10.90	1.95	0.05651
AC034207.1	0.26	8.09	1.95	0.05654
TIMP2	-0.22	11.10	-1.95	0.05661
KIR2DS4	0.54	6.70	1.95	0.05662
MRPL20	0.15	9.40	1.95	0.05672
AL450405.1	0.45	9.44	1.95	0.05676
HECW2	-0.33	5.81	-1.95	0.05681
CYP1B1	-0.33	7.81	-1.95	0.05686
LRFN3	0.22	7.83	1.95	0.05690
TRBV5-1	0.30	8.01	1.95	0.05698
MVK	0.19	7.28	1.95	0.05707
MAP4K3-DT	0.19	5.25	1.95	0.05709
RAB31	-0.22	10.62	-1.95	0.05714
CEP128	-0.20	5.72	-1.95	0.05715
AP000936.3	0.27	6.91	1.95	0.05716
PDE12	-0.17	7.13	-1.95	0.05716

VEZT	-0.17	7.11	-1.95	0.05718
TRAPPC2L	0.17	8.74	1.95	0.05719
IGSF6	-0.24	10.70	-1.95	0.05720
MYBBP1A	0.15	9.51	1.95	0.05720
MEX3C	-0.24	7.63	-1.95	0.05720
PLAAT4	0.22	10.97	1.94	0.05726
ZFP41	0.17	6.83	1.94	0.05729
NFATC2IP	0.17	10.01	1.94	0.05731
TIGD7	-0.18	5.84	-1.94	0.05734
BRD3OS	0.15	7.72	1.94	0.05741
AL353662.2	0.18	5.69	1.94	0.05741
ZFAS1	0.20	8.82	1.94	0.05747
IFIT1	-0.68	8.49	-1.94	0.05754
APP	-0.20	9.99	-1.94	0.05765
POLK	-0.17	6.68	-1.94	0.05767
CCT7	0.15	11.10	1.94	0.05770
PBX2P1	-0.30	6.48	-1.94	0.05771
PXK	-0.24	8.45	-1.94	0.05771
H2BC19P	-0.28	6.24	-1.94	0.05783
CISD3	0.23	8.38	1.94	0.05788
ATP6AP2	-0.14	9.80	-1.94	0.05789
ARID2	-0.19	7.65	-1.94	0.05790
P3H3	0.20	5.67	1.94	0.05790
RAP2A	-0.19	7.20	-1.94	0.05793
MDM1	-0.16	7.23	-1.94	0.05796
AL137127.1	0.20	5.23	1.94	0.05812
TLR3	-0.24	5.01	-1.94	0.05818
GOLGA7B	0.22	8.16	1.94	0.05822
TSPYL4	-0.17	7.04	-1.94	0.05823
DCTN3	0.17	9.50	1.94	0.05823
MYO7A	-0.23	6.53	-1.94	0.05826
F2RL3	0.26	5.91	1.94	0.05837
HIGD2A	0.24	10.73	1.94	0.05839
HAUS6	-0.18	6.76	-1.94	0.05839
CDIPT	0.16	10.86	1.94	0.05843
REL	-0.17	6.72	-1.94	0.05844
AL359921.2	0.17	5.62	1.93	0.05850
IWS1	-0.10	9.51	-1.93	0.05860
GNG7	0.21	8.73	1.93	0.05865
ABAT	-0.19	8.46	-1.93	0.05870
DNPEP	0.17	9.63	1.93	0.05871
CCNJ	-0.17	5.95	-1.93	0.05872
EPSTI1	-0.45	8.39	-1.93	0.05873
YES1P1	0.30	5.58	1.93	0.05880
ARL5B	-0.16	6.57	-1.93	0.05881
KEL	0.41	6.63	1.93	0.05886
CNOT8	-0.12	9.61	-1.93	0.05890
NEB	-0.33	4.99	-1.93	0.05890
FAM241B	0.21	5.62	1.93	0.05893
KBTBD4	-0.12	8.38	-1.93	0.05895

ZBTB45	0.13	8.49	1.93	0.05901
AL590762.3	0.19	5.18	1.93	0.05911
FERMT3	0.17	12.62	1.93	0.05911
ZMAT3	0.17	8.03	1.93	0.05914
FAM234A	0.15	9.67	1.93	0.05920
DRAM1	-0.19	7.75	-1.93	0.05924
EMC8	0.15	8.48	1.93	0.05928
SPPL2A	-0.14	8.64	-1.93	0.05934
C21orf59-TCP10L	0.29	5.33	1.93	0.05934
BATF	0.21	9.05	1.93	0.05938
AC092070.2	-0.26	6.52	-1.93	0.05940
CARD6	-0.22	7.67	-1.93	0.05941
RPS2P46	0.31	11.14	1.93	0.05945
SMC1A	-0.17	9.82	-1.93	0.05946
SIRT3	0.12	8.24	1.93	0.05953
ARHGEF3	-0.16	10.13	-1.93	0.05956
RABEP1	-0.13	8.93	-1.93	0.05960
DOCK2	-0.11	12.21	-1.93	0.05967
FRMD4B	-0.24	6.92	-1.93	0.05970
MAP2K1	-0.14	9.42	-1.92	0.05975
BCLAF3	-0.19	7.10	-1.92	0.05976
LYL1	0.32	10.69	1.92	0.05980
UBA52	0.28	14.77	1.92	0.05980
ARMCX3	-0.16	7.53	-1.92	0.05987
NUDT16P1	-0.22	6.61	-1.92	0.05988
LINC00106	-0.26	5.53	-1.92	0.05993
STK25	0.13	10.19	1.92	0.05996
TBC1D23	-0.19	7.41	-1.92	0.05998
SULF2	-0.21	11.51	-1.92	0.06000
RABGGTA	0.14	9.53	1.92	0.06004
IGBP1	0.12	10.51	1.92	0.06004
DLEC1	-0.24	6.57	-1.92	0.06004
GTPBP2	-0.18	9.78	-1.92	0.06008
SEC23A	-0.25	7.32	-1.92	0.06009
ZNF200	-0.17	8.30	-1.92	0.06013
PADI2	-0.40	11.34	-1.92	0.06016
AC004453.1	0.30	10.30	1.92	0.06027
AC116353.6	-0.21	5.89	-1.92	0.06031
TOM1	0.21	10.93	1.92	0.06037
CASP1	-0.19	11.14	-1.92	0.06042
SELENOW	0.19	9.97	1.92	0.06045
AC245297.4	0.22	6.17	1.92	0.06046
PLLP	0.23	5.26	1.92	0.06047
OTX1	-0.37	5.97	-1.92	0.06053
PPP4R3A	-0.12	9.47	-1.92	0.06055
RPL10P4	0.20	6.30	1.92	0.06056
RPL23AP2	0.24	7.96	1.92	0.06058
AC099336.2	0.31	9.46	1.92	0.06063
CLSPN	0.23	5.58	1.92	0.06064
AL365258.3	0.15	5.04	1.92	0.06064

PON2	-0.16	6.27	-1.92	0.06068
CDC42P6	-0.26	6.62	-1.92	0.06074
TRGC1	-0.30	7.97	-1.92	0.06075
EIF5	-0.13	10.38	-1.92	0.06077
INPP4A	-0.16	9.93	-1.92	0.06078
MPEG1	-0.20	12.31	-1.92	0.06082
AL139349.1	-0.25	5.72	-1.92	0.06084
TBC1D25	0.14	9.36	1.92	0.06090
ZNF345	-0.19	5.42	-1.92	0.06091
SMYD5	0.19	8.62	1.92	0.06093
NAB2	0.17	7.72	1.92	0.06096
SLC25A36	-0.17	7.17	-1.91	0.06103
NBPF12	-0.20	7.61	-1.91	0.06105
MACROH2A2	0.16	6.50	1.91	0.06119
TPRA1	0.14	9.07	1.91	0.06119
FGFR2	-0.29	5.08	-1.91	0.06126
IGLV3-21	0.53	8.00	1.91	0.06130
SDR39U1	0.18	9.35	1.91	0.06130
CCZ1B	-0.29	7.39	-1.91	0.06132
CREB5	-0.34	9.45	-1.91	0.06134
IGKV1-17	0.46	6.33	1.91	0.06137
PYGO2	0.10	10.01	1.91	0.06148
TBC1D10B	0.14	10.69	1.91	0.06149
NKAP	0.15	8.42	1.91	0.06150
RHOT1	-0.18	8.15	-1.91	0.06152
FBXO3	-0.15	7.79	-1.91	0.06156
MBIP	-0.16	6.23	-1.91	0.06157
AC008555.7	-0.17	5.38	-1.91	0.06158
PODXL2	0.25	6.01	1.91	0.06159
ZNF749	-0.18	5.70	-1.91	0.06160
DCAF4	-0.19	7.20	-1.91	0.06165
TRBJ2-7	0.26	6.21	1.91	0.06166
RPL12P42	0.18	5.28	1.91	0.06167
NCAPG2	-0.24	6.12	-1.91	0.06172
HSPG2	0.20	6.21	1.91	0.06172
GSK3A	0.17	10.77	1.91	0.06174
RNF169	-0.14	8.65	-1.91	0.06176
SUMO3	0.12	10.00	1.91	0.06180
CRY1	-0.17	6.80	-1.91	0.06181
FZD1	-0.23	6.88	-1.91	0.06183
EIF4A2	-0.14	10.92	-1.91	0.06184
ROMO1	0.23	8.99	1.91	0.06189
SCRN2	0.19	8.20	1.91	0.06189
IL27RA	0.17	11.14	1.91	0.06190
AC009120.1	0.30	5.53	1.91	0.06192
CACFD1	0.20	6.63	1.91	0.06194
SPEN	-0.19	10.74	-1.91	0.06197
UFM1	-0.16	7.56	-1.91	0.06199
LINC02328	0.17	5.79	1.91	0.06199
KIF1B	-0.25	8.49	-1.91	0.06199

CTU2	0.18	7.09	1.91	0.06200
RPL29P12	0.13	5.29	1.91	0.06204
TARBP2	0.19	8.30	1.91	0.06207
POLDIP2	0.11	9.65	1.91	0.06212
ASAP1	-0.25	9.39	-1.91	0.06213
UNC119B	-0.15	8.47	-1.91	0.06214
AL031123.1	-0.19	5.46	-1.91	0.06221
RAD23B	-0.17	9.43	-1.91	0.06221
KLC2	0.18	8.12	1.91	0.06225
SEC23B	-0.12	9.36	-1.91	0.06226
TGFBR1	-0.16	9.23	-1.91	0.06226
AC091153.1	0.21	6.80	1.91	0.06230
HERC5	-0.49	7.83	-1.90	0.06237
SMIM19	0.17	7.17	1.90	0.06241
HPSE	-0.21	8.38	-1.90	0.06244
AP1B1	0.14	11.21	1.90	0.06245
NCAPD3	-0.13	7.72	-1.90	0.06246
ZNF107	-0.30	6.74	-1.90	0.06250
UCKL1	0.18	9.54	1.90	0.06252
KDM4C	-0.13	8.55	-1.90	0.06260
PRORP	0.18	5.90	1.90	0.06261
ZBTB18	-0.23	9.60	-1.90	0.06261
ANXA2R	0.17	8.92	1.90	0.06265
NMT1	0.11	10.89	1.90	0.06267
F5	-0.29	9.07	-1.90	0.06269
MRPL37	0.18	9.07	1.90	0.06274
AL590867.2	0.27	10.50	1.90	0.06276
ACOT8	0.14	9.05	1.90	0.06276
LINC02035	-0.23	8.05	-1.90	0.06280
MAPRE2	-0.10	9.69	-1.90	0.06288
FIBP	0.18	10.09	1.90	0.06291
RPL22L1	0.19	7.41	1.90	0.06292
EBP	0.21	8.00	1.90	0.06294
SLC35C2	0.11	10.49	1.90	0.06298
SDF4	0.13	11.33	1.90	0.06299
AP3B1	-0.13	8.50	-1.90	0.06303
AC131235.2	0.25	9.16	1.90	0.06303
CXCR3	0.27	9.03	1.90	0.06308
LARP4B	-0.11	9.99	-1.90	0.06308
CITED4	0.26	6.26	1.90	0.06309
AGAP1	-0.24	6.95	-1.90	0.06310
RGS10	0.23	10.68	1.90	0.06312
CLTB	0.17	9.80	1.90	0.06313
POLR3H	0.15	8.72	1.90	0.06314
SLCO4C1	-0.29	6.45	-1.90	0.06315
PIK3R4	-0.15	7.90	-1.90	0.06315
NFKBIL1	0.20	7.29	1.90	0.06319
SART1	0.15	11.24	1.90	0.06321
ENO1	0.17	13.24	1.90	0.06324
TEX2	-0.18	7.43	-1.90	0.06327

NGLY1	-0.14	8.26	-1.90	0.06327
COG8	0.17	8.53	1.90	0.06328
PRSS57	0.31	5.51	1.90	0.06330
MAP3K13	0.19	7.16	1.90	0.06331
ATM	-0.22	9.66	-1.90	0.06332
ACD	0.16	8.96	1.90	0.06335
KLF12	-0.17	8.15	-1.90	0.06335
EGLN1	-0.30	7.27	-1.90	0.06338
ZNF426	0.14	7.44	1.90	0.06341
GFOD2	0.16	8.60	1.90	0.06343
FAH	0.18	8.01	1.90	0.06346
MED1	-0.16	8.67	-1.90	0.06351
MIR29B2CHG	-0.34	8.36	-1.90	0.06357
ZNHIT2	0.21	6.90	1.90	0.06358
ITPRIP	-0.22	10.59	-1.90	0.06361
DPYSL4	0.47	5.41	1.90	0.06362
GPSM3	0.19	13.52	1.90	0.06364
CYB561A3	0.20	11.02	1.89	0.06371
FAM49A	-0.16	9.57	-1.89	0.06372
EEFSEC	0.18	9.04	1.89	0.06376
SPP1	-0.38	5.29	-1.89	0.06377
TUBA1C	0.14	9.17	1.89	0.06377
FXYD7	0.25	5.73	1.89	0.06384
PIK3IP1	0.19	11.32	1.89	0.06384
SMIM5	0.35	7.66	1.89	0.06386
ZNF407	-0.13	7.54	-1.89	0.06387
VKORC1	0.23	7.46	1.89	0.06388
ELP5	0.15	8.77	1.89	0.06390
AP000347.1	0.36	6.67	1.89	0.06396
CREB3	0.19	8.54	1.89	0.06397
FAM149B1	-0.17	6.61	-1.89	0.06398
BBC3	0.23	8.69	1.89	0.06401
SLAMF1	0.17	8.57	1.89	0.06403
PLEKHA5	-0.20	5.63	-1.89	0.06406
MAML1	-0.19	9.72	-1.89	0.06406
RASSF5	-0.15	11.91	-1.89	0.06406
PPP1R12C	0.16	10.74	1.89	0.06411
CYC1	0.18	10.07	1.89	0.06414
AL662795.2	0.16	7.06	1.89	0.06428
RN7SL832P	0.24	5.18	1.89	0.06430
FBRS	0.15	11.36	1.89	0.06438
SMARCD2	0.11	11.02	1.89	0.06440
TMEM260	-0.19	7.86	-1.89	0.06446
ESPL1	-0.21	5.21	-1.89	0.06449
MMADHC	-0.14	8.82	-1.89	0.06456
TRAPPC1	0.18	10.96	1.89	0.06458
ZCWPW1	0.18	7.00	1.89	0.06469
MRM1	0.18	7.19	1.89	0.06473
AL672207.1	-0.29	5.25	-1.89	0.06474
RAB39B	-0.16	6.22	-1.89	0.06475

NAGS	0.18	5.99	1.89	0.06475
ZNF394	0.11	9.57	1.89	0.06477
AC055839.1	0.32	5.55	1.89	0.06484
PCM1	-0.14	9.37	-1.89	0.06497
AF001548.2	0.29	7.98	1.89	0.06500
SEC61A1	0.12	11.52	1.89	0.06502
SNX1	-0.12	10.79	-1.88	0.06511
TTC1	0.12	9.41	1.88	0.06512
ATP5MPL	0.17	9.53	1.88	0.06517
AC016772.1	-0.25	5.20	-1.88	0.06517
SLC66A2	0.18	10.02	1.88	0.06519
ARFGAP2	0.11	11.11	1.88	0.06522
CYB5R4	-0.23	9.08	-1.88	0.06522
LINC00482	-0.24	5.38	-1.88	0.06522
SELENOTP1	-0.22	5.29	-1.88	0.06527
GPR65	-0.15	8.91	-1.88	0.06529
SLC9A3R1	0.16	12.56	1.88	0.06530
NDUFAF8	0.22	7.71	1.88	0.06530
CRKL	-0.12	9.96	-1.88	0.06532
ABCF1	0.15	10.74	1.88	0.06532
MT-TH	0.40	6.31	1.88	0.06534
LETMD1	0.10	9.27	1.88	0.06534
AC091429.1	0.18	6.14	1.88	0.06535
RANBP20P	-0.33	6.47	-1.88	0.06538
HRH4	-0.24	5.06	-1.88	0.06539
RASSF7	0.19	7.85	1.88	0.06549
TFAP4	0.17	7.83	1.88	0.06550
UQCC2	0.19	7.98	1.88	0.06551
IFT22	0.21	5.85	1.88	0.06558
IL21R	0.17	9.45	1.88	0.06564
MBTD1	-0.21	7.03	-1.88	0.06564
WDR46	0.14	9.63	1.88	0.06567
AL139100.2	0.24	6.60	1.88	0.06572
SOD2	-0.32	13.25	-1.88	0.06572
NTAN1P2	0.18	5.05	1.88	0.06574
SLC16A10	0.24	5.55	1.88	0.06578
LOXL3	-0.20	7.71	-1.88	0.06579
CXCR2	-0.37	13.10	-1.88	0.06588
DYNC1LI2	-0.15	8.39	-1.88	0.06591
TMEM184C	-0.17	7.37	-1.88	0.06604
PXDC1	-0.21	5.63	-1.88	0.06613
NLRC3	-0.15	9.68	-1.88	0.06616
LGALSL	-0.32	7.21	-1.88	0.06617
ANGEL2	-0.14	7.27	-1.88	0.06618
RTN3P1	-0.32	6.44	-1.88	0.06619
DUSP2	0.27	8.58	1.88	0.06620
NACAP2	0.17	5.75	1.88	0.06632
SCAT8	-0.26	5.67	-1.88	0.06633
C1orf131	-0.12	7.35	-1.88	0.06641
CTNNBL1	0.16	9.73	1.88	0.06643

NID1	-0.26	6.97	-1.87	0.06644
SLF2	-0.14	7.76	-1.87	0.06648
LMNB1	-0.31	9.40	-1.87	0.06649
EVI2B	-0.20	11.71	-1.87	0.06649
WNT7A	0.25	6.41	1.87	0.06653
ASB13	0.16	8.01	1.87	0.06654
TNFRSF18	0.23	6.56	1.87	0.06658
KIF22	0.14	9.75	1.87	0.06665
MBD2	-0.11	10.05	-1.87	0.06666
PLPP6	-0.16	7.03	-1.87	0.06668
IRAK2	-0.16	6.62	-1.87	0.06671
MACROH2A1	-0.16	11.58	-1.87	0.06672
PAQR3	-0.19	5.36	-1.87	0.06672
ZMYM3	-0.14	8.71	-1.87	0.06673
IRAK3	-0.37	8.94	-1.87	0.06676
DCTN4	-0.19	8.07	-1.87	0.06677
CPNE5	0.25	9.15	1.87	0.06681
POLR2A	-0.16	11.96	-1.87	0.06686
SPTY2D1	-0.14	8.19	-1.87	0.06688
ZSCAN32	-0.14	7.89	-1.87	0.06691
TMX3	-0.15	7.53	-1.87	0.06692
LYPD3	0.25	5.47	1.87	0.06695
ZNF821	0.17	7.30	1.87	0.06699
CFAP161	0.21	5.37	1.87	0.06703
SPTBN5	-0.26	6.04	-1.87	0.06704
FHDC1	-0.36	5.67	-1.87	0.06705
PPP6R1	0.11	11.57	1.87	0.06712
CARMIL2	0.22	9.88	1.87	0.06714
RPL37AP1	0.32	9.07	1.87	0.06714
RPL37P23	0.27	8.09	1.87	0.06714
DTNB	0.15	7.53	1.87	0.06722
HTR7P1	-0.15	5.26	-1.87	0.06722
AKT2	0.11	11.45	1.87	0.06725
AC139768.1	0.23	5.37	1.87	0.06728
GPKOW	0.15	9.08	1.87	0.06729
TRBV13	0.25	5.60	1.87	0.06729
CD82	0.20	11.38	1.87	0.06730
DCK	-0.19	7.60	-1.87	0.06731
CASS4	-0.26	8.88	-1.87	0.06732
IPCEF1	-0.12	8.72	-1.87	0.06733
SDC3	-0.29	6.55	-1.87	0.06735
SALL2	0.23	6.18	1.87	0.06737
ABCB10	-0.22	6.92	-1.87	0.06737
CD2	0.21	11.80	1.87	0.06739
NUDC	0.17	9.96	1.87	0.06741
TRIM11	0.11	9.51	1.87	0.06742
CAAP1	-0.19	6.48	-1.87	0.06745
HS3ST1	0.32	5.66	1.87	0.06759
IKZF5	-0.17	7.07	-1.87	0.06760
PTPRJ	-0.21	11.82	-1.87	0.06761

MFSD10	0.17	9.80	1.87	0.06766
LCK	0.17	12.13	1.87	0.06767
PPP1CC	-0.14	9.49	-1.87	0.06771
TTC21B	-0.16	6.83	-1.87	0.06779
POLR3GL	0.19	7.52	1.87	0.06782
WAPL	-0.12	9.16	-1.87	0.06783
TNIP2	0.12	9.68	1.86	0.06789
IL17RA	-0.22	12.46	-1.86	0.06801
PRPF18	0.17	6.30	1.86	0.06802
MANF	0.17	9.23	1.86	0.06811
MZT1	-0.18	6.04	-1.86	0.06813
AC025181.2	0.23	5.59	1.86	0.06821
SLC30A6	-0.14	7.19	-1.86	0.06823
CYLD	-0.13	10.51	-1.86	0.06830
CSNK1G3	-0.14	6.90	-1.86	0.06841
ACOT13	0.14	6.92	1.86	0.06846
DDIT4	0.24	9.39	1.86	0.06861
NABP1	-0.30	9.54	-1.86	0.06866
PPP1R12B	-0.29	9.24	-1.86	0.06866
CASK	-0.14	7.25	-1.86	0.06868
APLP2	-0.16	12.84	-1.86	0.06869
AC133644.2	0.26	5.64	1.86	0.06872
GPA33	0.23	7.32	1.86	0.06875
AC005224.2	0.28	5.17	1.86	0.06875
EMC6	0.16	7.82	1.86	0.06878
AC096664.2	0.23	6.03	1.86	0.06887
NAB1	-0.19	7.23	-1.86	0.06888
P4HA1	-0.15	7.40	-1.86	0.06890
MYLK	-0.27	6.67	-1.86	0.06890
PRKDC	-0.24	10.32	-1.86	0.06891
APPL2	-0.16	8.50	-1.86	0.06897
IRAK1	0.12	10.81	1.86	0.06904
BLK	0.33	9.04	1.86	0.06907
STK35	-0.17	8.89	-1.86	0.06912
MRM3	0.15	7.99	1.86	0.06922
FAM83G	0.21	7.20	1.86	0.06924
STX3	-0.26	10.80	-1.85	0.06931
NDUFA6	0.17	9.08	1.85	0.06931
YWHAZP2	-0.26	5.74	-1.85	0.06937
MIIP	0.18	9.44	1.85	0.06942
CCAR2	0.10	11.03	1.85	0.06944
PRDM4	-0.12	8.44	-1.85	0.06951
AC124016.3	0.18	5.05	1.85	0.06956
GLS	-0.17	8.81	-1.85	0.06957
AC015813.1	-0.29	6.50	-1.85	0.06958
AL671277.2	-0.22	5.19	-1.85	0.06968
CHKB-DT	0.24	5.75	1.85	0.06971
NBPF25P	-0.16	5.15	-1.85	0.06978
HLA-DPB2	-0.30	5.34	-1.85	0.06980
FAAP100	0.15	9.26	1.85	0.06984

ZC3H13	0.20	10.00	1.85	0.06987
PDE4DIP	0.15	8.24	1.85	0.06990
UBL5	0.18	10.66	1.85	0.06998
FDXR	0.20	6.95	1.85	0.07010
USP3	-0.12	10.20	-1.85	0.07010
ITPR1	-0.22	8.05	-1.85	0.07014
AC005000.1	-0.21	5.89	-1.85	0.07015
NDUFB10	0.18	9.96	1.85	0.07020
AC002511.2	-0.27	5.13	-1.85	0.07023
ZNF555	-0.14	6.13	-1.85	0.07024
HLA-F-AS1	-0.19	5.46	-1.85	0.07030
GPATCH4	0.20	8.16	1.85	0.07031
PIWIL4	-0.17	5.95	-1.85	0.07033
AC010099.1	0.21	5.67	1.85	0.07037
SNRNP35	0.15	9.00	1.85	0.07037
MED26	0.11	7.72	1.85	0.07038
BEX2	0.23	7.02	1.85	0.07049
ARNTL	-0.17	8.65	-1.85	0.07051
ZFP91	-0.15	8.27	-1.85	0.07054
WRAP53	0.16	8.24	1.85	0.07056
ZNF234	-0.15	6.36	-1.85	0.07056
SYNGR2	0.17	11.63	1.85	0.07062
PPFIA1	-0.19	8.90	-1.85	0.07063
GFI1B	0.25	9.01	1.85	0.07064
TUFM	0.15	11.10	1.85	0.07064
EML6	-0.27	5.20	-1.85	0.07065
RPL3P12	0.20	5.56	1.85	0.07067
TRBV6-4	-0.24	5.19	-1.85	0.07069
AC135506.1	0.17	6.05	1.85	0.07070
LINC00243	0.32	6.28	1.85	0.07071
AC087284.1	0.33	5.73	1.85	0.07073
TLCD3A	-0.22	5.10	-1.85	0.07076
PBX3	-0.14	7.22	-1.84	0.07078
SCAT1	0.22	4.97	1.84	0.07079
RHOC	0.25	9.47	1.84	0.07080
FAM214A	-0.20	7.71	-1.84	0.07089
FAM114A2	-0.14	7.17	-1.84	0.07090
SAR1A	-0.11	9.71	-1.84	0.07092
NDUFC1	0.17	7.91	1.84	0.07094
CEP170	-0.27	6.64	-1.84	0.07095
RAP1A	-0.15	9.88	-1.84	0.07095
ZNF222	-0.15	5.75	-1.84	0.07096
NDOR1	0.16	8.77	1.84	0.07104
LPCAT4	0.14	9.29	1.84	0.07109
ZNF551	-0.21	6.48	-1.84	0.07110
SLC9A7	-0.24	6.84	-1.84	0.07112
LINC00476	0.26	6.89	1.84	0.07113
AC090498.1	0.25	7.24	1.84	0.07121
ZNF605	-0.17	6.37	-1.84	0.07122
TSPO	0.26	10.96	1.84	0.07126

GPS1	0.15	10.40	1.84	0.07136
AC095052.1	0.29	5.79	1.84	0.07137
DCAF11	0.11	10.46	1.84	0.07140
NCF2	-0.22	13.33	-1.84	0.07142
AC132008.2	-0.23	6.34	-1.84	0.07146
AC012354.2	0.21	5.98	1.84	0.07148
VPS18	0.15	10.05	1.84	0.07149
DCP2	-0.15	9.47	-1.84	0.07154
GBP2	-0.24	11.60	-1.84	0.07161
SDK2	0.30	6.43	1.84	0.07173
RB1	-0.19	7.53	-1.84	0.07173
WDR35	-0.19	6.01	-1.84	0.07177
AL109741.1	0.23	5.12	1.84	0.07180
RPS10P7	0.15	5.93	1.84	0.07181
KATNB1	0.12	9.26	1.84	0.07184
EPM2A	-0.17	5.88	-1.84	0.07185
RUFY4	-0.30	6.09	-1.84	0.07187
RPL21P39	0.24	7.39	1.84	0.07194
AC009495.3	-0.28	5.29	-1.84	0.07196
TUSC2	0.13	9.23	1.84	0.07201
CRISPLD2	-0.30	10.55	-1.84	0.07205
LMO2	-0.19	9.57	-1.84	0.07211
WNK1	-0.20	10.90	-1.84	0.07225
OTUD1	-0.16	7.94	-1.83	0.07229
VRK2	-0.16	6.80	-1.83	0.07231
SYS1	0.11	9.19	1.83	0.07234
H2AJ	0.20	8.96	1.83	0.07240
PNPLA6	0.13	11.21	1.83	0.07243
PLEKHB2	-0.14	10.43	-1.83	0.07246
UQCRH	0.18	10.08	1.83	0.07250
AMOT	-0.18	5.74	-1.83	0.07255
RTN1	-0.26	8.29	-1.83	0.07261
ARHGEF19	0.18	7.47	1.83	0.07264
UBA6	-0.14	7.38	-1.83	0.07267
ABCB8	0.16	8.63	1.83	0.07267
ARV1	0.13	6.46	1.83	0.07268
GIHCG	0.16	5.96	1.83	0.07281
RPP25L	0.17	8.65	1.83	0.07281
CNOT1	-0.09	11.23	-1.83	0.07287
GABPB1	-0.14	7.17	-1.83	0.07288
TSN	-0.11	8.98	-1.83	0.07298
TTL	-0.12	7.93	-1.83	0.07300
DNAJA2	-0.11	9.17	-1.83	0.07300
PDCD1	0.18	6.42	1.83	0.07301
ADAM17	-0.16	8.67	-1.83	0.07307
AC022144.1	0.25	4.97	1.83	0.07319
PPP6C	-0.11	9.45	-1.83	0.07321
MYCBP	-0.12	7.90	-1.83	0.07326
CHD6	-0.14	8.45	-1.83	0.07330
CCDC88C	-0.13	10.77	-1.83	0.07330

TMSB10	0.24	14.81	1.83	0.07334
DYNLT1	-0.20	9.78	-1.83	0.07340
ITGA3	0.18	6.84	1.83	0.07341
THOC2	0.21	9.35	1.83	0.07343
LSM4	0.18	9.57	1.83	0.07343
NAP1L1	0.18	11.57	1.83	0.07345
PACRGL	-0.15	5.78	-1.83	0.07349
ZNF267	-0.23	7.78	-1.83	0.07351
QTRT1	0.18	8.68	1.83	0.07354
FEZ1	0.25	6.29	1.83	0.07359
PNN	-0.21	9.23	-1.83	0.07364
HSP90AB2P	-0.13	5.01	-1.83	0.07372
AC122718.1	0.28	5.99	1.83	0.07374
TRBV19	0.33	7.85	1.82	0.07380
PSMB10	0.17	10.37	1.82	0.07387
OCIAD1	-0.10	9.26	-1.82	0.07390
PHAX	0.16	8.23	1.82	0.07394
ZFP36L1	-0.22	11.91	-1.82	0.07395
PBRM1	-0.16	8.29	-1.82	0.07395
ILRUN	0.11	10.96	1.82	0.07402
PCK2	0.16	8.96	1.82	0.07403
AL133520.1	0.15	5.22	1.82	0.07410
LRRC58	0.22	8.13	1.82	0.07422
GRAMD1C	-0.28	5.79	-1.82	0.07426
EXTL2	-0.17	5.18	-1.82	0.07430
POLR2F	0.18	9.52	1.82	0.07430
MCM9	-0.15	7.51	-1.82	0.07433
SOCS5	-0.15	6.45	-1.82	0.07437
DEDD2	0.16	11.04	1.82	0.07438
ELK4	-0.18	8.23	-1.82	0.07449
ADPRH	-0.18	7.60	-1.82	0.07454
ZMYM4	-0.15	7.78	-1.82	0.07461
CORO1B	0.18	10.11	1.82	0.07462
ACBD4	0.18	7.76	1.82	0.07467
AC138951.1	-0.29	5.22	-1.82	0.07467
BCL2L12	0.14	7.21	1.82	0.07472
YTHDC1	-0.11	9.76	-1.82	0.07472
FADD	0.15	9.66	1.82	0.07473
PUS1	0.17	8.62	1.82	0.07476
AC010894.5	-0.26	6.50	-1.82	0.07478
CNTROB	0.12	8.76	1.82	0.07478
HNRNPH1P3	-0.21	5.58	-1.82	0.07483
Z83843.1	-0.29	5.16	-1.82	0.07486
CCNL1	-0.23	9.88	-1.82	0.07490
ZNF19	0.16	5.56	1.82	0.07490
U40455.1	0.22	5.27	1.82	0.07494
AKR1B1	0.16	10.06	1.82	0.07494
PBXIP1	0.16	12.54	1.82	0.07507
ARHGAP21	-0.18	7.55	-1.82	0.07508
PDE3B	-0.22	8.38	-1.82	0.07509

RPL18P11	0.20	6.05	1.82	0.07512
CMAHP	-0.17	6.57	-1.82	0.07513
SMC3	-0.15	8.52	-1.82	0.07518
OR52K3P	-0.32	7.78	-1.82	0.07518
KCNJ15	-0.37	10.92	-1.82	0.07520
TMEM144	-0.21	5.52	-1.82	0.07522
STMN3	0.20	9.61	1.82	0.07524
SMNDC1	-0.15	7.93	-1.82	0.07528
MRPS11	0.15	8.70	1.82	0.07531
NAMPT	-0.37	12.36	-1.81	0.07533
GRHPR	0.19	9.39	1.81	0.07542
NAMPTP1	-0.42	9.07	-1.81	0.07543
TSR1	0.14	8.42	1.81	0.07547
PRR5	0.22	8.92	1.81	0.07558
SPATC1L	0.35	7.29	1.81	0.07572
FAM135A	-0.20	5.40	-1.81	0.07577
PDE4B	-0.20	9.16	-1.81	0.07579
MRFAP1	0.12	11.35	1.81	0.07579
STEAP4	-0.39	10.37	-1.81	0.07580
CBY1	0.14	7.14	1.81	0.07584
TNFAIP3	-0.24	9.14	-1.81	0.07589
ZMAT5	0.15	8.55	1.81	0.07591
TRMT1L	-0.17	7.34	-1.81	0.07594
ANKRD35	0.24	5.09	1.81	0.07594
GIT2	-0.12	11.01	-1.81	0.07596
CINP	0.13	8.76	1.81	0.07597
MT-TD	0.26	5.84	1.81	0.07598
KHDC4	-0.19	8.37	-1.81	0.07599
PWWP2B	0.19	7.99	1.81	0.07599
H2BC20P	-0.26	6.19	-1.81	0.07607
SNU13	0.17	10.55	1.81	0.07615
ELP6	0.16	8.24	1.81	0.07618
DFFB	-0.14	7.09	-1.81	0.07618
TMEM245	0.20	8.45	1.81	0.07618
AF131215.5	-0.27	5.64	-1.81	0.07622
COMTD1	0.22	6.53	1.81	0.07624
UBASH3A	0.21	9.24	1.81	0.07628
OTUB1	0.14	10.94	1.81	0.07637
MOGS	0.12	10.23	1.81	0.07641
ARHGAP4	0.14	11.88	1.81	0.07642
CSK	0.14	13.03	1.81	0.07643
C1orf115	0.22	6.10	1.81	0.07656
ZSCAN29	-0.15	7.42	-1.81	0.07657
OGFOD3	0.15	8.75	1.81	0.07660
GCLM	0.19	7.40	1.81	0.07661
UBE2D3P1	-0.24	6.63	-1.81	0.07665
EFL1	-0.13	7.92	-1.81	0.07670
SHOC2	-0.19	8.58	-1.81	0.07680
CNOT2	0.15	9.44	1.80	0.07693
TMEM250	0.14	10.45	1.80	0.07698

AC004151.1	0.15	13.57	1.80	0.07701
NEMF	0.22	9.27	1.80	0.07703
SKAP1	0.16	9.45	1.80	0.07714
MT-ND4L	-0.23	12.08	-1.80	0.07719
NT5C3A	-0.24	8.27	-1.80	0.07724
ATP5MC2	0.20	11.78	1.80	0.07732
XRCC6	0.12	11.68	1.80	0.07735
MRPS12	0.19	8.00	1.80	0.07736
BAG5	-0.12	8.42	-1.80	0.07738
PRPS2	-0.15	7.46	-1.80	0.07741
FADS1	-0.19	7.04	-1.80	0.07747
TRAV20	-0.21	5.96	-1.80	0.07748
ARMC5	0.15	7.75	1.80	0.07749
PBX2	-0.19	11.10	-1.80	0.07757
RFXAP	-0.17	5.55	-1.80	0.07766
PKD2	-0.19	6.11	-1.80	0.07769
VAMP3	-0.13	9.59	-1.80	0.07779
IGF2BP2	0.36	8.82	1.80	0.07783
COX6C	0.19	9.04	1.80	0.07789
AKR7A2	0.17	9.00	1.80	0.07801
SULT1B1	-0.36	8.08	-1.80	0.07804
ZNF761	-0.19	6.42	-1.80	0.07808
EVL	0.16	12.77	1.80	0.07817
FEM1B	-0.14	8.35	-1.80	0.07818
SKP2	-0.15	6.55	-1.80	0.07818
RPS15A	0.22	11.30	1.80	0.07819
AC021092.2	-0.16	6.23	-1.80	0.07819
AC090825.1	-0.28	5.51	-1.80	0.07822
LRRC8B	-0.19	6.43	-1.80	0.07825
CLDND1	-0.16	8.00	-1.80	0.07829
BMPR2	-0.18	7.05	-1.80	0.07837
NAGPA	0.17	9.26	1.80	0.07842
FANCF	-0.14	7.35	-1.80	0.07847
HDHD2	0.16	6.72	1.80	0.07849
FBXO45	-0.13	6.66	-1.79	0.07856
ATMIN	-0.13	8.55	-1.79	0.07857
NDE1	-0.17	10.20	-1.79	0.07857
ZFP3	-0.17	6.18	-1.79	0.07866
RPS27L	0.17	8.10	1.79	0.07867
ZNF600	-0.18	7.78	-1.79	0.07868
AC027290.3	0.27	6.46	1.79	0.07877
TMX4	-0.20	9.86	-1.79	0.07882
C11orf21	-0.16	10.47	-1.79	0.07884
OCIAD2	0.19	7.95	1.79	0.07888
SRGAP3	0.17	5.66	1.79	0.07890
CFAP97	-0.21	6.78	-1.79	0.07892
SSH1	-0.16	9.38	-1.79	0.07899
MAP7D1	0.14	12.08	1.79	0.07899
RPRD1B	-0.18	7.97	-1.79	0.07900
DIMT1	-0.19	6.74	-1.79	0.07906

IFI44	-0.45	7.66	-1.79	0.07909
CXXC1	0.13	10.45	1.79	0.07910
AC113404.3	-0.32	6.28	-1.79	0.07911
CUL4B	-0.13	8.89	-1.79	0.07912
NDUFA8	0.17	8.14	1.79	0.07915
CLU	0.33	12.09	1.79	0.07917
RPS6KB1	-0.16	7.40	-1.79	0.07920
CACUL1	-0.16	8.90	-1.79	0.07924
AP006222.1	-0.31	6.13	-1.79	0.07930
BAX	0.16	10.53	1.79	0.07933
MAML2	-0.23	7.72	-1.79	0.07934
ADGRL1	0.17	8.50	1.79	0.07934
AC140479.3	-0.18	5.20	-1.79	0.07935
CRNKL1	-0.13	8.89	-1.79	0.07943
WDR92	0.20	6.67	1.79	0.07946
USPL1	-0.15	7.96	-1.79	0.07949
POLR2J3	0.24	7.09	1.79	0.07956
BATF2	-0.47	6.98	-1.79	0.07961
RNASEK	0.17	6.78	1.79	0.07970
RPS7P1	0.26	9.26	1.79	0.07971
COMMD1	0.19	7.68	1.79	0.07976
TTC14	-0.24	7.82	-1.79	0.07977
RBM47	-0.29	9.64	-1.79	0.07978
MMAB	0.17	7.43	1.79	0.07981
NUP133	-0.13	8.28	-1.79	0.07984
NIPBL	-0.12	9.83	-1.79	0.07985
PAM	-0.24	7.93	-1.79	0.07986
DLSTP1	-0.19	5.29	-1.79	0.07992
RPL39L	0.22	5.45	1.79	0.08004
JAM3	0.24	7.36	1.79	0.08007
LCLAT1	-0.19	5.76	-1.79	0.08008
OCEL1	0.17	8.19	1.78	0.08016
TSC1	-0.15	8.68	-1.78	0.08021
PFDN5	0.20	11.82	1.78	0.08022
ACTL6A	-0.14	7.46	-1.78	0.08027
ZNF408	0.15	8.39	1.78	0.08028
EEF2KMT	0.14	7.32	1.78	0.08032
FADS3	0.19	7.61	1.78	0.08032
AP001273.1	-0.17	5.08	-1.78	0.08035
GXYLT1	-0.15	6.95	-1.78	0.08038
ZNF550	-0.22	6.25	-1.78	0.08039
CBWD1	-0.18	6.20	-1.78	0.08041
TMEM131	-0.16	9.68	-1.78	0.08043
SHQ1	-0.14	6.99	-1.78	0.08046
C4orf48	0.27	6.63	1.78	0.08065
MAFK	0.14	8.90	1.78	0.08067
JMJD6	0.14	9.29	1.78	0.08072
ABRACL	0.16	8.84	1.78	0.08073
EIF2S2	0.13	10.03	1.78	0.08073
MED11	0.13	8.74	1.78	0.08080

ENTPD1	-0.23	9.97	-1.78	0.08088
RNF8	0.15	7.85	1.78	0.08093
CNPY4	0.15	8.26	1.78	0.08097
NPEPPSP1	-0.20	5.03	-1.78	0.08101
SMPD1	0.11	9.77	1.78	0.08106
TRBV7-7	0.20	4.96	1.78	0.08106
CLIP1	-0.22	8.47	-1.78	0.08110
GEMIN6	0.17	7.53	1.78	0.08115
MON2	-0.21	7.53	-1.78	0.08116
PKM	0.15	13.50	1.78	0.08117
TRBV7-4	0.25	5.52	1.78	0.08122
NRGN	0.32	12.76	1.78	0.08126
AL133260.1	0.21	7.17	1.78	0.08126
PARP12	-0.30	10.09	-1.78	0.08131
AC007298.2	-0.31	6.91	-1.78	0.08133
RPL31P2	0.19	6.50	1.78	0.08134
AHSA1	0.11	10.06	1.78	0.08134
SELL	-0.21	13.68	-1.78	0.08135
PCSK5	-0.13	6.79	-1.78	0.08137
ZSCAN12	-0.22	5.51	-1.78	0.08140
ERCC2	0.15	8.44	1.78	0.08147
SLC25A24	-0.21	6.66	-1.78	0.08149
CCDC86	0.14	7.88	1.78	0.08150
AAMP	0.13	10.28	1.78	0.08164
CFAP298	0.18	6.40	1.78	0.08166
CR1	-0.39	10.01	-1.78	0.08167
LINC01145	-0.23	7.52	-1.78	0.08174
SREK1IP1	0.24	8.14	1.77	0.08182
MARCHF2	0.23	10.82	1.77	0.08184
NSMAF	-0.14	8.91	-1.77	0.08191
PPID	-0.11	8.54	-1.77	0.08195
AC112656.1	-0.19	5.16	-1.77	0.08197
AL022311.1	0.22	5.81	1.77	0.08198
BEST1	-0.32	9.36	-1.77	0.08202
AC008026.1	0.22	6.62	1.77	0.08211
RNF112	-0.20	5.21	-1.77	0.08213
RRS1	0.20	7.04	1.77	0.08215
HK2	-0.23	9.25	-1.77	0.08219
RABEPK	0.13	8.27	1.77	0.08225
AC090152.1	-0.16	6.03	-1.77	0.08226
NAA38	0.22	9.41	1.77	0.08228
GOLGA8B	-0.34	7.94	-1.77	0.08229
PHBP19	0.25	5.16	1.77	0.08235
UTP4	0.15	7.94	1.77	0.08237
PPP1R14A	0.29	6.41	1.77	0.08238
FUT7	0.21	8.72	1.77	0.08239
LINC02432	-0.33	6.43	-1.77	0.08243
RALY-AS1	-0.17	5.36	-1.77	0.08248
NDUFS5	0.20	9.57	1.77	0.08261
HSD17B4	-0.12	8.54	-1.77	0.08262

DTWD2	-0.17	5.70	-1.77	0.08265
RALB	-0.25	10.92	-1.77	0.08266
PFDN6	0.15	8.42	1.77	0.08273
TIMM22	0.14	8.35	1.77	0.08280
GAB2	-0.27	10.88	-1.77	0.08281
CCHCR1	0.19	7.88	1.77	0.08284
ENSA	0.12	10.64	1.77	0.08285
TICAM1	0.15	8.46	1.77	0.08290
CTTNBP2NL	-0.24	5.25	-1.77	0.08293
KRT1	0.59	7.98	1.77	0.08297
AC012236.1	0.27	5.17	1.77	0.08300
NOS3	0.20	6.19	1.77	0.08305
NOXA1	0.23	5.85	1.77	0.08314
PNRC2	-0.14	9.48	-1.77	0.08334
ITGB7	0.14	11.81	1.77	0.08339
FBXL4	-0.15	7.13	-1.76	0.08347
ACTR3P2	-0.30	5.71	-1.76	0.08349
BRAF	-0.17	7.98	-1.76	0.08357
MPZL3	-0.25	9.37	-1.76	0.08358
AC026403.1	0.25	8.32	1.76	0.08364
FAM86C2P	0.18	5.62	1.76	0.08374
ARHGEF11	-0.18	10.25	-1.76	0.08379
BTRC	-0.13	7.49	-1.76	0.08380
FAM53C	-0.21	10.55	-1.76	0.08382
DCUN1D5	0.14	6.61	1.76	0.08385
HNRNPK	-0.12	12.28	-1.76	0.08390
MARK4	0.14	9.10	1.76	0.08393
RPGR	-0.21	7.11	-1.76	0.08399
GOSR2	-0.11	8.58	-1.76	0.08400
TMEM30A	-0.16	9.62	-1.76	0.08418
EEF1B2P6	0.23	7.20	1.76	0.08418
CREB1	-0.14	8.73	-1.76	0.08421
PIK3C3	-0.12	8.47	-1.76	0.08423
FAM227B	-0.18	5.23	-1.76	0.08424
CTPS2	-0.18	6.29	-1.76	0.08427
FUT8	-0.19	6.91	-1.76	0.08430
DEF6	0.13	11.79	1.76	0.08441
UBL3	-0.16	8.40	-1.76	0.08444
KNOP1	0.20	7.90	1.76	0.08452
CHMP7	0.14	10.53	1.76	0.08462
MT-TT	0.29	6.05	1.76	0.08481
IGFBP7	0.19	7.03	1.76	0.08482
TMEM71	-0.21	9.88	-1.76	0.08483
PSMC5	0.14	11.01	1.76	0.08489
UBE3C	-0.11	9.41	-1.76	0.08490
SLC47A1	-0.23	5.06	-1.76	0.08491
N4BP2L1	-0.12	7.67	-1.76	0.08492
FPR2	-0.35	10.81	-1.76	0.08503
RPSAP18	0.18	5.82	1.76	0.08507
KRT23	-0.38	9.73	-1.76	0.08509

SLC35A4	0.13	10.81	1.76	0.08510
GRPEL1	0.11	8.66	1.75	0.08518
SEL1L	-0.18	8.61	-1.75	0.08521
ISOC2	0.22	7.63	1.75	0.08525
SRRT	0.12	10.72	1.75	0.08528
SEC13	0.13	10.17	1.75	0.08530
TMEM189	0.13	8.36	1.75	0.08545
AC132938.6	-0.28	5.24	-1.75	0.08546
PMVK	0.19	9.24	1.75	0.08551
YIPF4	0.20	9.76	1.75	0.08557
LINC00877	-0.22	7.75	-1.75	0.08557
ELOVL6	-0.23	6.13	-1.75	0.08563
SELENOH	0.16	6.70	1.75	0.08563
CETN2	0.15	7.49	1.75	0.08563
NPM3	0.17	7.71	1.75	0.08568
SIPA1	0.14	12.10	1.75	0.08574
POLI	-0.18	6.37	-1.75	0.08583
AKAP13	-0.15	11.87	-1.75	0.08589
TRABD	0.14	10.88	1.75	0.08595
YWHAZP3	-0.29	5.89	-1.75	0.08595
FMN1	-0.25	5.51	-1.75	0.08596
AL035587.1	-0.18	6.73	-1.75	0.08598
DRAM2	-0.11	8.66	-1.75	0.08605
FAM210A	-0.16	6.26	-1.75	0.08605
AAR2	0.14	9.30	1.75	0.08616
MFSD12	0.18	9.64	1.75	0.08616
CTU1	0.18	6.64	1.75	0.08617
MKLN1	-0.13	8.51	-1.75	0.08632
AC092155.2	0.25	7.15	1.75	0.08637
AC090559.1	-0.15	10.30	-1.75	0.08640
MRPS21	0.16	9.55	1.75	0.08642
LPIN2	-0.21	10.51	-1.75	0.08649
TTC8	-0.18	5.10	-1.75	0.08650
TTC17	-0.10	9.13	-1.75	0.08652
B3GNT5	-0.28	5.96	-1.75	0.08653
AC004585.1	0.19	6.40	1.75	0.08654
AMN1	-0.22	6.58	-1.75	0.08654
AC107954.1	-0.26	5.55	-1.75	0.08663
CXorf40B	0.15	8.15	1.75	0.08666
SEC62	0.29	11.04	1.75	0.08666
AMMECR1L	-0.11	7.89	-1.75	0.08668
OAT	-0.16	8.07	-1.75	0.08669
AC011497.2	-0.21	6.28	-1.75	0.08674
CHD3	0.13	11.90	1.75	0.08680
EXOSC1	0.12	8.52	1.75	0.08687
RPL22	0.14	10.48	1.75	0.08688
NCOA4	-0.26	12.05	-1.75	0.08690
LINC00909	-0.14	7.28	-1.74	0.08694
HIBADH	-0.14	6.87	-1.74	0.08696
ZFAND6	-0.15	8.56	-1.74	0.08709

AKAP5	-0.20	5.12	-1.74	0.08711
PRPF3	-0.10	9.55	-1.74	0.08711
SIPA1L2	-0.37	7.86	-1.74	0.08717
THAP6	-0.12	6.83	-1.74	0.08733
AKAP10	-0.16	8.65	-1.74	0.08735
TRBV7-9	0.31	7.91	1.74	0.08740
COA3	0.21	9.07	1.74	0.08745
FUBP1	-0.12	9.36	-1.74	0.08746
BECN1	-0.11	10.00	-1.74	0.08748
ZNF775	0.17	6.67	1.74	0.08752
AHCYL1	-0.11	9.43	-1.74	0.08762
PPP2R2A	-0.12	8.94	-1.74	0.08765
CHID1	0.16	8.80	1.74	0.08768
DCUN1D4	-0.16	6.58	-1.74	0.08774
CCDC152	0.19	5.37	1.74	0.08775
RPLP0P6	0.23	9.52	1.74	0.08777
RPL13AP6	0.19	5.49	1.74	0.08777
CREBBP	-0.18	11.33	-1.74	0.08783
IKBKB	-0.12	10.45	-1.74	0.08785
LINC02863	-0.31	5.87	-1.74	0.08795
METTL15	0.13	6.49	1.74	0.08798
TRMT61A	0.18	7.48	1.74	0.08799
ZNF702P	-0.22	5.44	-1.74	0.08810
SSBP3	0.15	10.34	1.74	0.08810
POLR2H	0.14	8.37	1.74	0.08810
PHF8	-0.13	9.16	-1.74	0.08825
ABT1	0.11	9.44	1.74	0.08826
RPL18P13	0.24	7.30	1.74	0.08827
SPRYD3	0.11	10.27	1.74	0.08842
PPM1N	0.19	6.45	1.74	0.08849
BIN2P1	-0.23	6.81	-1.74	0.08855
B4GALT5	-0.29	10.31	-1.74	0.08856
S100PBP	-0.14	8.01	-1.74	0.08866
CLPTM1	0.11	10.76	1.74	0.08867
GLIDR	0.20	5.07	1.73	0.08871
CAPS	0.19	7.88	1.73	0.08872
POU5F2	0.28	5.26	1.73	0.08877
CCR10	0.22	5.61	1.73	0.08880
RNF144B	-0.19	8.57	-1.73	0.08884
NCOR1	0.16	11.19	1.73	0.08888
CSF2RBP1	-0.30	5.10	-1.73	0.08889
TAP1	-0.20	12.48	-1.73	0.08894
MUC20-OT1	0.28	8.06	1.73	0.08895
TRRAP	-0.13	10.22	-1.73	0.08896
USP49	-0.20	5.98	-1.73	0.08897
TFIP11	0.13	10.24	1.73	0.08903
GPATCH2	-0.15	6.17	-1.73	0.08910
P2RX5	0.27	8.84	1.73	0.08915
ZNF594	-0.17	5.56	-1.73	0.08918
LINC01355	-0.28	5.16	-1.73	0.08922

UBE2F	0.18	7.77	1.73	0.08927
CENPW	0.16	5.29	1.73	0.08930
GIPC3	0.19	6.23	1.73	0.08945
TMEM14A	0.15	5.84	1.73	0.08948
KIAA1841	-0.19	5.66	-1.73	0.08949
CTBP1	0.11	11.21	1.73	0.08950
AC092145.1	-0.31	6.62	-1.73	0.08954
GOLM1	-0.21	7.43	-1.73	0.08957
PHF19	0.13	9.57	1.73	0.08959
AGK	-0.17	7.45	-1.73	0.08962
PAGE2B	0.29	5.41	1.73	0.08967
DCTN2	0.12	10.48	1.73	0.08974
CCDC159	0.17	9.68	1.73	0.08984
NDUFAF4	0.20	6.70	1.73	0.08987
AL109811.2	-0.19	5.59	-1.73	0.08992
DTD1	0.14	7.30	1.73	0.08993
RDH10	-0.17	5.76	-1.73	0.08999
DLGAP4	0.10	10.09	1.73	0.09003
CPVL	-0.28	10.66	-1.73	0.09009
TSC22D4	0.15	11.48	1.73	0.09020
CCDC97	0.09	10.04	1.73	0.09021
KLRF1	-0.25	7.92	-1.73	0.09022
SMURF1	-0.12	8.58	-1.73	0.09023
NCR3	0.25	8.61	1.73	0.09028
ANKRD20A11P	0.40	5.38	1.73	0.09033
ADAMTSL4	-0.25	9.42	-1.73	0.09042
TCP11L2	-0.28	7.89	-1.73	0.09047
APOBEC3A	-0.34	11.68	-1.72	0.09055
MATK	0.24	10.05	1.72	0.09057
ZNF133	0.12	7.63	1.72	0.09058
RNF216P1	0.12	8.07	1.72	0.09066
XPNPEP2	0.19	5.91	1.72	0.09074
ZKSCAN3	-0.13	6.08	-1.72	0.09077
PATL2	-0.26	8.27	-1.72	0.09079
NR3C2	-0.21	6.38	-1.72	0.09083
ITFG1	-0.15	7.81	-1.72	0.09086
STX12	-0.12	8.20	-1.72	0.09088
CA15P1	-0.23	5.14	-1.72	0.09094
R3HDM2	-0.10	8.51	-1.72	0.09095
FBXL8	0.18	6.23	1.72	0.09098
FRMD4A	0.37	6.35	1.72	0.09099
TM6SF1	-0.23	7.35	-1.72	0.09101
RPL5	0.17	13.13	1.72	0.09106
FAM222B	0.10	8.15	1.72	0.09106
SUPT20H	-0.16	9.38	-1.72	0.09116
CPSF7	-0.15	10.48	-1.72	0.09117
NOP56	0.12	10.62	1.72	0.09122
LANCL1	-0.16	7.19	-1.72	0.09124
TNFRSF13B	0.29	6.86	1.72	0.09130
TDP2	-0.17	9.44	-1.72	0.09130

VAT1	0.15	9.67	1.72	0.09131
TSR2	0.12	9.29	1.72	0.09132
HERC2P2	-0.31	7.11	-1.72	0.09145
TBC1D13	0.10	9.42	1.72	0.09157
TREM1	-0.30	11.47	-1.72	0.09161
GCA	-0.34	11.28	-1.72	0.09162
RPP21	-0.19	5.30	-1.72	0.09163
ADO	-0.11	7.36	-1.72	0.09173
MACROD1	0.18	6.06	1.72	0.09173
DNAJB4	-0.18	5.27	-1.72	0.09180
CD68	0.33	6.82	1.72	0.09181
ACBD3	-0.15	7.53	-1.72	0.09185
USP46	-0.17	5.92	-1.72	0.09187
FAM13A	-0.22	7.27	-1.72	0.09189
GNA11	0.20	6.65	1.72	0.09190
RPS2P5	0.23	11.22	1.72	0.09205
ENTPD4	-0.15	9.51	-1.72	0.09208
AK5	0.25	7.08	1.72	0.09213
STRN	-0.15	7.78	-1.72	0.09217
TFR2	0.22	6.76	1.72	0.09221
ATOH8	-0.43	6.05	-1.72	0.09221
RPL10AP6	0.26	9.55	1.72	0.09222
NOP14	0.12	9.05	1.72	0.09223
PAXIP1-AS2	-0.16	6.81	-1.72	0.09224
NUB1	-0.17	10.51	-1.72	0.09229
HEATR5A	-0.19	6.97	-1.71	0.09237
DDX19B	0.14	8.54	1.71	0.09238
LYSMD2	-0.18	8.46	-1.71	0.09239
GSAP	-0.19	8.19	-1.71	0.09239
PCBP4	0.17	8.24	1.71	0.09241
AC002075.2	0.18	6.17	1.71	0.09245
COX8A	0.22	9.83	1.71	0.09254
CBWD5	-0.20	5.94	-1.71	0.09254
UBE2E1	-0.12	8.24	-1.71	0.09270
SGPL1	-0.12	9.01	-1.71	0.09277
AP001816.1	0.20	6.57	1.71	0.09277
EPS8	-0.18	5.34	-1.71	0.09284
PDP1	-0.17	7.38	-1.71	0.09285
WDR36	-0.12	7.14	-1.71	0.09287
HMGXB4	0.15	7.77	1.71	0.09291
PCNX1	-0.21	10.11	-1.71	0.09298
TTC33	-0.15	6.52	-1.71	0.09302
RNU6ATAC39P	0.29	5.30	1.71	0.09308
BCL11A	0.19	8.53	1.71	0.09314
BIRC5	0.19	5.65	1.71	0.09322
IGLC7	0.40	6.36	1.71	0.09329
BOLA3-AS1	0.16	5.39	1.71	0.09332
NOMO3	-0.16	7.12	-1.71	0.09333
VPS26C	-0.12	9.37	-1.71	0.09334
ZNF137P	-0.16	5.13	-1.71	0.09335

CD320	0.21	8.85	1.71	0.09338
DYRK4	0.14	7.33	1.71	0.09339
ISCA2	0.15	7.54	1.71	0.09341
CDKN2AIP	-0.16	7.50	-1.71	0.09352
GIMAP2	-0.16	9.49	-1.71	0.09355
RPS18	0.20	13.85	1.71	0.09366
CCDC82	-0.19	7.18	-1.71	0.09381
DRG2	0.12	9.56	1.71	0.09382
TOMM5	0.17	5.58	1.71	0.09397
Z94721.3	0.40	6.43	1.71	0.09400
AC106820.1	0.23	6.62	1.71	0.09402
PNOC	0.24	7.34	1.71	0.09403
TUT4	-0.14	8.73	-1.71	0.09408
NOC2L	0.12	9.89	1.71	0.09408
FKBP15	-0.13	10.75	-1.71	0.09408
RN7SL600P	0.28	6.15	1.71	0.09410
ARPC4	0.18	11.61	1.71	0.09415
CCSAP	-0.19	7.25	-1.71	0.09415
ZNF286B	0.15	5.42	1.71	0.09415
LIPE	0.18	7.44	1.71	0.09416
EIF2B4	0.11	8.87	1.71	0.09417
EPB41L3	-0.26	7.68	-1.70	0.09420
RTRAF	0.14	9.58	1.70	0.09422
SOS1	-0.18	7.89	-1.70	0.09423
GLT8D1	-0.11	8.24	-1.70	0.09434
GNLY	-0.34	13.23	-1.70	0.09435
CDK5	0.16	7.72	1.70	0.09437
SHISA4	0.51	7.66	1.70	0.09439
RRP7A	0.15	9.66	1.70	0.09440
HECTD4	-0.21	9.21	-1.70	0.09447
CHMP2B	-0.15	7.61	-1.70	0.09457
MS4A4E	-0.22	5.53	-1.70	0.09463
TMTC1	-0.37	5.49	-1.70	0.09465
STX6	-0.11	8.71	-1.70	0.09467
STRIP1	-0.09	9.50	-1.70	0.09474
GNB5	0.14	8.85	1.70	0.09479
GNPAT	-0.10	8.61	-1.70	0.09479
LINC00662	0.27	7.54	1.70	0.09479
LARP7	0.22	9.46	1.70	0.09491
MIER1	-0.17	8.26	-1.70	0.09492
CPD	-0.33	9.67	-1.70	0.09493
AL096870.2	0.19	5.07	1.70	0.09511
TEAD2	0.23	5.06	1.70	0.09513
TLR2	-0.28	10.76	-1.70	0.09524
PSMA1	0.14	7.06	1.70	0.09533
TLR7	-0.20	7.45	-1.70	0.09535
DSTNP2	0.14	5.97	1.70	0.09540
BIVM	-0.19	5.90	-1.70	0.09540
GLUD1	-0.09	9.87	-1.70	0.09552
TMEM43	-0.16	10.13	-1.70	0.09552

IFITM3	-0.60	12.11	-1.70	0.09552
RAB1B	0.14	12.00	1.70	0.09554
POLR1D	0.13	10.05	1.70	0.09555
ENOPH1	-0.16	7.34	-1.70	0.09557
ANKS6	0.16	7.28	1.70	0.09559
CREBRF	-0.20	9.02	-1.70	0.09560
ATF2	-0.16	7.63	-1.70	0.09564
ZNRD1	0.16	8.68	1.70	0.09572
MT-TS2	0.35	6.73	1.70	0.09576
PACS2	0.14	9.79	1.70	0.09589
PKP4	-0.15	7.74	-1.70	0.09597
FRG1	0.15	8.15	1.70	0.09599
RPUSD2	0.15	7.56	1.70	0.09603
SAP30BP	0.11	10.43	1.69	0.09611
MAP3K1	-0.19	9.49	-1.69	0.09615
AC016582.2	-0.22	6.16	-1.69	0.09621
MOB1B	-0.16	6.88	-1.69	0.09622
NIN	-0.17	9.98	-1.69	0.09626
DAD1	0.12	10.05	1.69	0.09626
NCDN	0.14	8.47	1.69	0.09630
COMMD7	0.16	9.00	1.69	0.09633
TAPBPL	0.15	10.24	1.69	0.09635
TRIM52-AS1	0.21	7.59	1.69	0.09648
KCTD15	-0.17	7.55	-1.69	0.09651
RFX3	-0.20	6.65	-1.69	0.09653
POMGNT2	0.13	7.39	1.69	0.09653
UBALD2	0.20	11.68	1.69	0.09654
HERC2	-0.16	9.24	-1.69	0.09666
METTL2B	0.13	7.95	1.69	0.09674
MTREX	-0.13	8.16	-1.69	0.09699
MAN1A2	0.21	8.89	1.69	0.09707
AL162151.2	0.24	7.17	1.69	0.09708
NUP155	-0.15	7.12	-1.69	0.09710
TMEM219	0.17	10.18	1.69	0.09711
VMO1	0.38	6.05	1.69	0.09718
LINC00674	0.17	5.41	1.69	0.09718
AQP9	-0.33	12.15	-1.69	0.09727
NTNG2	-0.32	8.75	-1.69	0.09728
MBTPS1	-0.10	9.85	-1.69	0.09730
WIPF1	-0.16	12.15	-1.69	0.09736
AC009244.1	0.19	5.18	1.69	0.09739
GPANK1	0.14	8.70	1.69	0.09741
JRKL	-0.20	5.96	-1.69	0.09743
INTS3	-0.13	10.56	-1.69	0.09745
AC005014.1	-0.23	5.80	-1.69	0.09745
AC080023.2	-0.26	5.16	-1.69	0.09745
MINDY2	0.19	7.39	1.69	0.09748
FDPS	0.10	9.86	1.69	0.09748
FCGR2A	-0.27	12.44	-1.69	0.09752
AC018816.1	0.18	5.03	1.69	0.09758

DOCK11P1	-0.23	5.71	-1.69	0.09760
TRIM4	-0.11	8.83	-1.69	0.09765
FCAR	-0.33	7.55	-1.69	0.09771
PYCARD-AS1	0.15	5.45	1.69	0.09772
E4F1	0.13	9.09	1.69	0.09783
KHNYN	-0.10	10.52	-1.69	0.09793
TSNAX	-0.14	8.02	-1.69	0.09798
ARHGEF5	0.17	6.09	1.69	0.09801
ID2	-0.12	9.80	-1.68	0.09808
AC091825.3	0.19	5.66	1.68	0.09813
PIK3CB	-0.15	8.15	-1.68	0.09815
CEP120	-0.20	7.27	-1.68	0.09816
PMPCB	-0.09	8.94	-1.68	0.09816
AREL1	-0.13	10.05	-1.68	0.09833
TNK1	0.20	6.03	1.68	0.09838
SRGN	-0.27	13.84	-1.68	0.09842
AFAP1L2	-0.18	5.93	-1.68	0.09851
CDC14A	-0.23	7.18	-1.68	0.09853
DNAJB1	0.11	10.89	1.68	0.09855
ZCCHC8	-0.15	8.16	-1.68	0.09856
AFG3L2	0.11	9.04	1.68	0.09858
CD19	0.35	8.57	1.68	0.09868
MORN1	0.17	5.64	1.68	0.09869
EIF3J	0.30	9.00	1.68	0.09876
HEATR3	-0.14	7.30	-1.68	0.09880
RETREG1	0.21	7.27	1.68	0.09880
TNFRSF13C	0.26	8.82	1.68	0.09881
SETD6	-0.14	6.30	-1.68	0.09885
RNF167	0.14	11.80	1.68	0.09899
KCTD21	-0.15	7.94	-1.68	0.09900
LMAN2	0.14	11.71	1.68	0.09900
TMPPE	-0.21	5.61	-1.68	0.09910
OXNAD1	0.19	8.24	1.68	0.09913
B3GALNT2	-0.17	6.82	-1.68	0.09916
UQCRB	0.15	10.67	1.68	0.09920
TENT4A	-0.13	8.44	-1.68	0.09928
VPS8	-0.11	9.31	-1.68	0.09930
ZNF483	-0.20	5.54	-1.68	0.09935
MGAM	-0.41	10.76	-1.68	0.09941
F8A3	0.19	6.18	1.68	0.09941
AL050331.1	0.26	8.26	1.68	0.09942
RAB18	-0.14	8.55	-1.68	0.09950
DRG1	0.14	8.73	1.68	0.09955
YAF2	0.15	7.64	1.68	0.09964
PPIAP31	0.18	5.97	1.68	0.09968
SEC14L1	-0.25	12.46	-1.68	0.09969
RSAD2	-0.64	7.93	-1.68	0.09969
MPLKIP	0.13	7.15	1.68	0.09970
NEIL2	0.15	7.71	1.68	0.09971
TRAV6	0.25	5.39	1.68	0.09973

EML4	-0.14	8.93	-1.68	0.09988
RPL9P32	0.15	5.29	1.68	0.09990
ACYP2	0.13	6.29	1.67	0.10000
RTN3	-0.22	11.55	-1.67	0.10005
KLHL20	-0.16	6.55	-1.67	0.10007
HYAL3	0.19	6.03	1.67	0.10015
AC002350.2	0.22	5.16	1.67	0.10024
TWISTNB	0.22	7.52	1.67	0.10027
ALCAM	-0.17	7.01	-1.67	0.10030
ZNF391	-0.19	4.94	-1.67	0.10034
CTSO	-0.14	7.70	-1.67	0.10035
C1QBP	0.11	9.14	1.67	0.10038
AC012671.1	0.19	5.70	1.67	0.10045
PSMF1	0.26	12.16	1.67	0.10047
AL139095.2	0.26	8.91	1.67	0.10047
ST13P15	0.17	5.56	1.67	0.10048
BCL2L11	-0.15	9.14	-1.67	0.10056
SSBP4	0.19	9.38	1.67	0.10062
MTHFD2	-0.18	7.96	-1.67	0.10067
TRGV10	-0.20	5.84	-1.67	0.10082
SLC66A1	0.13	7.86	1.67	0.10085
AC000089.1	0.22	7.61	1.67	0.10093
AC246787.1	0.27	9.02	1.67	0.10094
CEP250	0.12	10.09	1.67	0.10102
PKMP4	0.19	5.62	1.67	0.10112
OXA1L	0.11	11.49	1.67	0.10113
AC026316.1	0.17	6.25	1.67	0.10117
SEMA6B	0.43	5.60	1.67	0.10117
CDC34	0.30	11.18	1.67	0.10119
MSNP1	-0.34	7.47	-1.67	0.10124
PCGF3	-0.22	9.06	-1.67	0.10125
BCOR	-0.21	8.73	-1.67	0.10127
BUB1	-0.19	5.26	-1.67	0.10128
CAPZB	0.12	12.77	1.67	0.10134
PITPNA-AS1	0.18	5.84	1.67	0.10143
UQCRHL	0.15	6.86	1.67	0.10146
PAN2	-0.17	8.79	-1.67	0.10149
ATP5IF1	0.12	9.46	1.67	0.10153
ZNF3	0.12	8.64	1.67	0.10159
CMTM4	-0.17	5.93	-1.67	0.10161
GNA15	0.17	9.17	1.67	0.10164
CHST10	0.18	6.41	1.67	0.10166
UPF3A	0.14	8.87	1.67	0.10172
TMPRSS9	0.28	5.28	1.67	0.10179
RPSAP58	0.26	9.75	1.67	0.10184
SIRT1	-0.18	7.19	-1.67	0.10185
COX6A1	0.17	9.44	1.66	0.10196
BFAR	0.09	9.02	1.66	0.10215
PDS5B	-0.11	8.29	-1.66	0.10219
CD1D	-0.14	9.32	-1.66	0.10221

RSBN1L	0.20	9.43	1.66	0.10232
RTN2	0.18	7.59	1.66	0.10233
UQCC1	-0.13	8.32	-1.66	0.10235
ADAT2	-0.25	6.29	-1.66	0.10236
SETD1B	-0.11	10.53	-1.66	0.10245
H2BC21	-0.26	8.47	-1.66	0.10255
AC090114.2	0.21	6.81	1.66	0.10255
KLRB1	0.23	9.63	1.66	0.10258
UBE3B	-0.12	9.35	-1.66	0.10258
ZC3HC1	0.13	7.53	1.66	0.10262
CDK2AP2	0.18	10.38	1.66	0.10273
PDXDC1	-0.14	8.85	-1.66	0.10277
ZNF77	0.15	6.05	1.66	0.10277
NUTF2	0.13	10.07	1.66	0.10278
SH2D1B	-0.29	7.98	-1.66	0.10282
INPP5K	0.12	10.77	1.66	0.10289
WIPF2	0.15	10.63	1.66	0.10292
COLQ	0.24	7.40	1.66	0.10306
ALG10B	-0.17	5.42	-1.66	0.10309
CRYZL2P	-0.15	5.48	-1.66	0.10314
AC018638.4	-0.27	7.76	-1.66	0.10315
TAOK1	-0.12	8.81	-1.66	0.10318
TTLL5	-0.13	7.27	-1.66	0.10318
CXCL5	-0.28	6.13	-1.66	0.10321
CLIC4	-0.21	6.85	-1.66	0.10327
ZNF30	-0.16	5.68	-1.66	0.10330
SCAMP4	0.15	9.10	1.66	0.10340
NOL4L	-0.16	9.12	-1.66	0.10341
PPM1D	-0.15	7.50	-1.66	0.10346
OAZ1	0.24	14.67	1.66	0.10352
AC147067.1	0.19	6.93	1.66	0.10355
RELL1	-0.21	7.86	-1.66	0.10355
SIPA1L1	-0.15	10.54	-1.66	0.10357
BIN2P2	-0.22	5.69	-1.66	0.10358
RPL3P7	0.30	8.83	1.66	0.10376
UAP1L1	-0.19	7.96	-1.66	0.10391
IL10RB-DT	0.20	5.90	1.65	0.10401
WDSUB1	-0.19	6.28	-1.65	0.10404
NHLRC4	0.17	5.20	1.65	0.10405
NAIPP2	-0.30	5.62	-1.65	0.10405
MIB2	0.18	8.45	1.65	0.10405
NDUFS4	0.17	7.76	1.65	0.10411
SRRD	0.19	8.58	1.65	0.10413
JARID2	-0.13	9.99	-1.65	0.10413
RPLP0P2	0.23	6.16	1.65	0.10420
HIRA	0.12	7.78	1.65	0.10426
CHCHD2	0.15	10.81	1.65	0.10429
RNF139	-0.12	8.21	-1.65	0.10447
BHLHE40-AS1	0.16	4.99	1.65	0.10452
H3C6	-0.24	5.45	-1.65	0.10455

H2AC6	-0.27	10.66	-1.65	0.10460
AC005154.2	-0.19	5.06	-1.65	0.10464
GNG2	-0.15	10.24	-1.65	0.10469
GBP5	-0.34	10.70	-1.65	0.10470
HDAC5	0.12	11.23	1.65	0.10487
HAGHL	0.20	6.17	1.65	0.10490
ARL4D	0.20	5.73	1.65	0.10494
TMED1	0.15	8.43	1.65	0.10502
NACC1	0.12	9.74	1.65	0.10507
IGHD	0.36	11.28	1.65	0.10509
TMEM87A	-0.10	8.58	-1.65	0.10509
AP5Z1	0.13	10.32	1.65	0.10509
CPNE1	0.16	11.39	1.65	0.10517
PARTICL	-0.12	5.27	-1.65	0.10518
AC104563.1	0.23	7.77	1.65	0.10521
ZDHHC13	-0.16	6.70	-1.65	0.10525
NBPF20	-0.22	7.32	-1.65	0.10536
CD81	0.16	11.08	1.65	0.10539
PRAG1	0.19	9.11	1.65	0.10539
TRUB1	-0.18	5.64	-1.65	0.10551
KLHDC10	-0.11	7.88	-1.65	0.10551
AL034370.1	-0.24	6.85	-1.65	0.10555
ALOX15P1	0.16	5.30	1.65	0.10557
RPS2P55	0.21	8.02	1.65	0.10558
DUSP18	0.22	7.68	1.65	0.10563
RTF1	0.19	10.39	1.65	0.10565
CTSC	-0.16	11.13	-1.65	0.10568
FAM114A1	-0.15	5.61	-1.65	0.10575
NDUFS6	0.22	9.47	1.65	0.10580
NPIPB15	0.38	5.70	1.65	0.10580
KLHL9	-0.15	7.79	-1.65	0.10594
AC245884.11	-0.21	5.19	-1.65	0.10600
TOE1	0.09	7.74	1.64	0.10605
AL033519.3	0.18	7.15	1.64	0.10606
PRR14	0.14	10.72	1.64	0.10611
FAM53B	0.13	10.92	1.64	0.10612
NIT2	0.13	7.50	1.64	0.10613
MRPS10	0.15	8.52	1.64	0.10613
CNPY2	0.18	6.95	1.64	0.10617
PHPT1	0.19	9.21	1.64	0.10617
ZNF776	-0.18	7.13	-1.64	0.10618
CASP5	-0.34	6.63	-1.64	0.10627
PRELID3B	-0.11	7.66	-1.64	0.10630
RPL35P5	0.23	7.47	1.64	0.10639
ESS2	0.11	9.16	1.64	0.10641
PPP1R14BP3	0.17	5.99	1.64	0.10647
CD2AP	-0.18	6.77	-1.64	0.10647
HIRIP3	0.15	8.36	1.64	0.10649
MLKL	-0.17	9.65	-1.64	0.10650
CD55	-0.31	10.55	-1.64	0.10657

ZNF575	0.16	6.43	1.64	0.10668
CACNB3	0.17	6.43	1.64	0.10674
SMARCAL1	0.12	8.77	1.64	0.10678
MAPK1IP1L	-0.08	9.28	-1.64	0.10681
MTCO2P12	0.21	8.73	1.64	0.10684
CD300E	-0.27	10.74	-1.64	0.10684
CDC6	-0.19	5.05	-1.64	0.10685
ATF1	-0.13	7.20	-1.64	0.10695
FBXO2	0.22	6.40	1.64	0.10700
CSRNP2	-0.14	7.50	-1.64	0.10700
EZH2	-0.14	6.83	-1.64	0.10702
TSKS	0.28	5.35	1.64	0.10705
AKIRIN2	-0.16	9.62	-1.64	0.10711
SCRN3	-0.13	6.14	-1.64	0.10713
AEBP1	0.30	7.83	1.64	0.10723
DHRS4L2	0.18	7.52	1.64	0.10734
TRBJ2-4	0.14	5.30	1.64	0.10737
ANKRD26	0.21	6.58	1.64	0.10744
CYHR1	0.13	10.08	1.64	0.10751
DOK2	0.18	11.60	1.64	0.10759
PGM5	-0.33	5.81	-1.64	0.10763
OSTM1	-0.14	8.44	-1.64	0.10778
ZNF22	-0.13	7.95	-1.64	0.10792
EGR1	-0.21	6.06	-1.64	0.10793
LACTB	-0.16	8.36	-1.64	0.10794
IGLV3-25	0.38	6.95	1.64	0.10794
THOC6	0.16	8.96	1.64	0.10794
TRMT1	0.16	9.40	1.64	0.10800
SEC24A	-0.17	6.77	-1.64	0.10808
ST6GALNAC6	0.23	5.36	1.64	0.10811
PPP1R3B	-0.28	9.75	-1.63	0.10818
ARNT	-0.13	8.95	-1.63	0.10821
CTNNA1P1	-0.19	5.30	-1.63	0.10822
ATIC	0.12	9.13	1.63	0.10831
AC097376.1	0.24	5.23	1.63	0.10834
ETV7	-0.45	6.88	-1.63	0.10836
IFIT3	-0.48	10.59	-1.63	0.10836
UGCG	-0.23	7.58	-1.63	0.10848
AC106872.2	0.16	6.29	1.63	0.10861
NIBAN1	-0.29	11.44	-1.63	0.10863
ZNF146	-0.17	7.95	-1.63	0.10864
SLFN13	-0.18	8.00	-1.63	0.10865
C6orf47	0.11	9.36	1.63	0.10868
TADA2B	-0.10	10.01	-1.63	0.10872
HMGN3P1	0.16	5.19	1.63	0.10874
AC012618.1	0.25	8.08	1.63	0.10874
NOP58	0.14	9.11	1.63	0.10876
PIAS1	-0.14	9.74	-1.63	0.10886
GK	-0.28	8.24	-1.63	0.10891
MTO1	-0.10	8.24	-1.63	0.10891

AC135068.6	0.15	5.34	1.63	0.10893
TASP1	-0.15	6.14	-1.63	0.10897
BCL6	-0.35	12.12	-1.63	0.10901
ZBTB43	-0.11	7.33	-1.63	0.10906
AL512844.1	-0.24	5.09	-1.63	0.10907
SLC25A3	0.10	11.30	1.63	0.10922
PLCB1	-0.16	5.95	-1.63	0.10935
PNPLA1	-0.23	6.40	-1.63	0.10957
ATXN7L2	0.16	7.31	1.63	0.10968
HLA-L	-0.55	9.79	-1.63	0.10973
MAP4K2	0.09	10.67	1.63	0.10974
RPL23AP57	0.13	5.50	1.63	0.10977
RNH1	0.18	11.46	1.63	0.10983
B2M	-0.16	16.09	-1.63	0.10984
INAFM2	-0.14	8.47	-1.63	0.10990
PLEKHG3	-0.15	11.36	-1.63	0.10992
UBE2L3	0.12	10.76	1.63	0.10994
AC020893.2	-0.14	5.29	-1.63	0.10995
CDKL5	-0.20	6.77	-1.63	0.10997
ZBTB8OS	0.15	7.71	1.63	0.11002
SLA2	0.13	9.59	1.63	0.11007
PRDM8	-0.19	7.43	-1.63	0.11007
CEP192	-0.15	7.77	-1.63	0.11017
NUDT22	0.14	8.93	1.63	0.11018
TRIM21	-0.17	10.44	-1.62	0.11027
ZSWIM1	-0.12	7.55	-1.62	0.11027
MYO1C	0.16	8.65	1.62	0.11034
EIF3I	0.14	10.93	1.62	0.11041
TRAV8-4	0.23	6.41	1.62	0.11046
PCCA-DT	0.18	5.05	1.62	0.11047
RAP1GAP	0.57	5.76	1.62	0.11047
ZNF282	0.10	9.02	1.62	0.11056
RPL12P4	0.25	8.08	1.62	0.11058
PPIE	0.12	9.48	1.62	0.11065
NOL8	-0.12	8.10	-1.62	0.11065
PREPL	-0.16	7.39	-1.62	0.11070
CAB39	-0.18	10.11	-1.62	0.11078
FTX	-0.24	7.87	-1.62	0.11082
CAPN5	0.21	7.54	1.62	0.11087
GTF3A	0.13	10.64	1.62	0.11091
CERNA1	0.21	5.07	1.62	0.11091
NUP50	-0.14	10.09	-1.62	0.11094
COPS6	0.12	9.80	1.62	0.11097
U2AF1L4	0.17	7.49	1.62	0.11100
RCE1	0.13	8.32	1.62	0.11104
NSF	-0.11	8.29	-1.62	0.11109
AC073111.4	0.18	7.93	1.62	0.11110
MAPK14	-0.22	10.71	-1.62	0.11114
TIGAR	0.19	6.63	1.62	0.11117
CCDC115	0.09	9.27	1.62	0.11125

ARHGAP18	-0.16	6.84	-1.62	0.11131
H19	0.32	6.00	1.62	0.11132
ZNF469	0.15	6.17	1.62	0.11133
SPTAN1	0.13	12.48	1.62	0.11143
AC108488.3	0.17	5.11	1.62	0.11144
PRKCZ	0.14	8.86	1.62	0.11149
TPI1	0.15	12.11	1.62	0.11152
ANKRD12	0.25	10.88	1.62	0.11156
VTA1	-0.13	7.91	-1.62	0.11161
SSBP1	0.14	8.70	1.62	0.11164
BAG1	0.30	11.90	1.62	0.11167
AL009179.1	0.18	5.00	1.62	0.11169
KLRC1	-0.28	6.03	-1.62	0.11176
ATF6B	0.12	11.26	1.62	0.11191
PSMB4	0.12	11.33	1.62	0.11202
FEZ2	-0.10	7.45	-1.62	0.11203
AC005674.2	-0.25	6.98	-1.62	0.11205
MRPL36	0.18	7.79	1.62	0.11208
C5orf63	0.15	5.62	1.62	0.11213
ATG12	-0.10	8.30	-1.62	0.11216
AC009061.2	0.27	7.27	1.62	0.11222
RELB	0.16	9.31	1.62	0.11223
METTL8	0.17	6.73	1.62	0.11233
APOBEC3G	-0.16	10.13	-1.62	0.11238
TMEM255B	0.19	5.48	1.61	0.11240
RAB11FIP3	0.11	8.73	1.61	0.11246
LRRC40	-0.15	6.04	-1.61	0.11246
AL158212.3	-0.19	6.57	-1.61	0.11252
FXN	0.15	6.33	1.61	0.11263
IL1RN	-0.27	10.10	-1.61	0.11270
CACNA1E	-0.31	6.43	-1.61	0.11270
CDK11B	0.12	10.12	1.61	0.11277
AP003307.2	0.14	5.10	1.61	0.11278
PPM1K	0.20	9.32	1.61	0.11283
RALGAPA2	-0.24	9.52	-1.61	0.11283
GUK1	0.31	12.57	1.61	0.11297
FNIP2	-0.18	6.65	-1.61	0.11298
SLC39A13	0.12	9.35	1.61	0.11298
PCNX2	0.16	8.43	1.61	0.11300
RPS7P14	0.17	5.65	1.61	0.11320
KCNH2	0.21	6.70	1.61	0.11341
PLEK2	0.36	6.81	1.61	0.11349
HEATR9	-0.25	5.31	-1.61	0.11358
GOLGA8N	-0.19	5.08	-1.61	0.11361
PINK1-AS	0.14	6.55	1.61	0.11362
RPS11P5	0.27	10.37	1.61	0.11368
LETM2	-0.16	6.60	-1.61	0.11370
RPL12P12	0.15	5.91	1.61	0.11370
SYCP2	-0.20	5.05	-1.61	0.11376
KANSL1	-0.11	9.91	-1.61	0.11376

HOXB3	-0.19	5.30	-1.61	0.11379
EEF1A1P7	0.12	5.57	1.61	0.11385
RPL7P9	0.22	9.60	1.61	0.11388
FBXO28	-0.14	7.45	-1.61	0.11396
ACSF2	0.15	8.23	1.61	0.11398
SERINC3	-0.14	10.38	-1.61	0.11408
ARFIP2	0.11	9.14	1.61	0.11409
STK26	-0.18	7.83	-1.61	0.11410
LINC02361	0.15	6.75	1.61	0.11413
MME	-0.29	10.44	-1.61	0.11418
AC026436.1	0.16	5.12	1.61	0.11427
PTRHD1	0.17	7.87	1.61	0.11434
ATP6V1E2	0.13	7.15	1.61	0.11435
ARRDC2	0.10	10.88	1.61	0.11440
CAPNS1	0.15	12.97	1.61	0.11441
PEX2	-0.13	7.79	-1.61	0.11447
BHLHE40	-0.16	10.10	-1.61	0.11447
SMARCD1	0.08	10.12	1.61	0.11448
FTSJ1	0.11	8.66	1.61	0.11457
AL096816.1	-0.16	6.56	-1.60	0.11467
ATF7	-0.11	8.16	-1.60	0.11471
CHMP1A	0.13	10.75	1.60	0.11485
RTL8C	0.14	8.99	1.60	0.11485
UBE2S	0.17	7.28	1.60	0.11503
CREG1	-0.19	9.02	-1.60	0.11504
AC097376.3	0.25	7.02	1.60	0.11504
HCST	0.19	11.15	1.60	0.11507
FSTL1	0.27	5.98	1.60	0.11509
RPL14P1	0.21	10.11	1.60	0.11514
TUBB4B	0.13	10.80	1.60	0.11527
RPS2P4	0.13	5.69	1.60	0.11530
HSPA9	-0.11	9.84	-1.60	0.11537
CLYBL	0.16	6.45	1.60	0.11548
AC025458.1	0.23	6.87	1.60	0.11554
KRBOX4	-0.14	6.63	-1.60	0.11556
FOXP3	0.20	7.91	1.60	0.11562
LCMT1	0.14	8.65	1.60	0.11568
RCAN3AS	0.15	5.31	1.60	0.11569
THG1L	0.13	7.54	1.60	0.11569
VAPB	-0.07	8.50	-1.60	0.11572
AC026401.3	0.21	7.50	1.60	0.11578
DHRS11	0.14	6.11	1.60	0.11582
ZNF772	-0.19	5.56	-1.60	0.11592
TRIB1	-0.25	8.46	-1.60	0.11593
ENTPD7	-0.17	6.03	-1.60	0.11599
DPY30	0.11	8.23	1.60	0.11600
DENND3	-0.23	11.97	-1.60	0.11605
PKD1P6	-0.21	5.22	-1.60	0.11609
KDELR1	0.13	10.19	1.60	0.11628
TCEAL1	0.18	5.64	1.60	0.11628

ANAPC2	0.11	9.65	1.60	0.11633
AARSD1	0.17	5.28	1.60	0.11637
PHKB	-0.10	8.95	-1.60	0.11637
CTDSP2	-0.13	12.01	-1.60	0.11637
RAB5A	-0.15	8.19	-1.60	0.11644
CIAO2B	0.14	9.53	1.60	0.11644
PNKP	0.14	9.74	1.60	0.11647
ATP6V1C1	-0.21	8.48	-1.60	0.11650
ARCN1	-0.08	10.02	-1.60	0.11658
SNRPB2	0.13	8.85	1.60	0.11662
FOSL2	-0.22	11.05	-1.60	0.11666
KCTD13	0.14	8.79	1.60	0.11669
RPSAP45	0.15	5.40	1.60	0.11671
CYFIP1	-0.15	9.10	-1.60	0.11674
BMERB1	0.21	5.94	1.60	0.11676
TAF1D	-0.09	8.60	-1.59	0.11682
TSEN54	0.15	9.64	1.59	0.11696
AGAP2	0.11	10.45	1.59	0.11701
SLC27A4	0.14	8.50	1.59	0.11705
TRABD2A	0.25	9.82	1.59	0.11708
AC107983.1	0.20	7.39	1.59	0.11709
SNHG29	0.15	10.63	1.59	0.11710
FCRL1	-0.33	7.33	-1.59	0.11716
PSMD1	0.11	9.27	1.59	0.11718
DENND4B	-0.14	11.28	-1.59	0.11726
DNAJB5	-0.23	6.37	-1.59	0.11731
ZBED5	-0.12	8.64	-1.59	0.11732
JADE3	-0.22	4.97	-1.59	0.11737
EIF2AK4	-0.15	7.92	-1.59	0.11739
FGFR1OP	-0.17	6.93	-1.59	0.11747
LSS	0.14	9.17	1.59	0.11748
KLRK1	-0.23	5.19	-1.59	0.11749
MITF	-0.16	5.38	-1.59	0.11759
RPS3P6	0.14	5.91	1.59	0.11764
EIF6	0.16	9.89	1.59	0.11765
SH3GLB1	-0.19	9.85	-1.59	0.11767
BNIP3	0.14	7.24	1.59	0.11780
IFNAR2	-0.13	9.69	-1.59	0.11780
MYPOP	0.13	7.47	1.59	0.11791
ZNF793	0.21	5.73	1.59	0.11793
AC243829.1	-0.32	5.32	-1.59	0.11794
RPL37AP8	0.16	5.82	1.59	0.11798
PSMA3-AS1	-0.17	8.86	-1.59	0.11802
HBS1L	-0.12	7.47	-1.59	0.11805
TOMM22	0.13	9.45	1.59	0.11806
SNRPC	0.16	8.84	1.59	0.11818
NCLN	0.13	10.32	1.59	0.11828
AL022344.1	0.20	5.21	1.59	0.11834
CNBP	-0.10	11.91	-1.59	0.11837
LSM5	0.17	6.96	1.59	0.11845

BTNL3	-0.73	6.53	-1.59	0.11846
TOLLIP	0.14	10.39	1.59	0.11847
PRADC1	0.17	7.27	1.59	0.11847
AL441992.2	0.17	5.48	1.59	0.11848
AC025627.3	0.20	5.12	1.59	0.11856
RAB21	-0.12	8.96	-1.59	0.11868
MAD2L2	0.17	9.24	1.59	0.11871
KLF11	-0.16	8.14	-1.59	0.11873
HMGN5	0.17	5.64	1.59	0.11877
CXXC5	0.16	9.21	1.59	0.11882
CLIC2	-0.23	5.58	-1.59	0.11884
FAM13B	-0.14	8.10	-1.59	0.11886
GPX7	0.15	7.52	1.59	0.11894
LAS1L	0.13	9.32	1.59	0.11901
MED7	0.12	6.78	1.58	0.11910
SAE1	0.11	9.50	1.58	0.11911
HBA1	0.44	12.89	1.58	0.11914
CRAT	0.15	9.08	1.58	0.11917
USP48	-0.12	8.99	-1.58	0.11917
LRRFIP2	-0.14	8.29	-1.58	0.11917
RPS14P8	0.32	6.10	1.58	0.11926
R3HDM1	-0.13	7.83	-1.58	0.11934
FAM126A	-0.13	7.03	-1.58	0.11934
DPCD	0.27	6.48	1.58	0.11937
SNX14	-0.12	8.00	-1.58	0.11948
CSNK1G2	0.11	11.20	1.58	0.11950
PPP3CC	0.12	8.43	1.58	0.11960
DAZAP2P1	-0.20	6.50	-1.58	0.11972
MT-CO1	-0.13	15.59	-1.58	0.11973
CFL1	0.15	14.23	1.58	0.11974
EHD1	0.13	12.71	1.58	0.11983
RBMXL1	-0.12	7.39	-1.58	0.11983
IMPDH1P3	-0.16	5.09	-1.58	0.11988
SECISBP2L	-0.17	7.45	-1.58	0.12003
BCL7A	0.22	7.20	1.58	0.12012
TPK1	-0.17	7.47	-1.58	0.12019
ATXN7	-0.18	8.73	-1.58	0.12024
EIF3H	0.11	11.62	1.58	0.12026
JADE1	0.14	9.33	1.58	0.12031
DNAJA1	-0.12	10.23	-1.58	0.12036
PDHX	-0.15	6.62	-1.58	0.12042
TRIM56	0.17	11.38	1.58	0.12044
RBM48	-0.11	7.35	-1.58	0.12044
BAK1	0.16	9.55	1.58	0.12046
NHLRC3	-0.15	7.09	-1.58	0.12051
RPS27AP16	0.23	9.91	1.58	0.12054
ARMH1	0.19	8.87	1.58	0.12061
AC017116.2	0.25	5.70	1.58	0.12064
PLVAP	0.36	7.80	1.58	0.12075
KCNAB3	-0.20	6.01	-1.58	0.12075

LINC00638	0.19	5.78	1.58	0.12076
ZNF674-AS1	-0.17	5.32	-1.58	0.12079
BZW1P2	-0.24	5.54	-1.58	0.12092
AC005072.1	0.23	5.10	1.58	0.12094
PDS5A	-0.14	9.08	-1.58	0.12095
AC078899.1	-0.29	5.46	-1.58	0.12105
AC026471.1	0.17	6.04	1.58	0.12108
SPRTN	-0.14	6.30	-1.58	0.12109
ZNF678	-0.14	5.84	-1.58	0.12118
RAD51D	0.15	7.64	1.58	0.12123
TGS1	-0.12	7.94	-1.58	0.12124
ICAM3	0.19	13.94	1.58	0.12126
RASGRP1	-0.18	8.66	-1.58	0.12127
COA8	0.13	7.78	1.58	0.12127
MYL6B	0.20	6.55	1.57	0.12142
ERAP2	0.49	9.58	1.57	0.12145
MAVS	0.10	10.50	1.57	0.12145
EHD3	0.19	8.36	1.57	0.12149
MCCC2	-0.11	7.95	-1.57	0.12155
TAF3	0.19	8.53	1.57	0.12157
QSER1	-0.15	6.42	-1.57	0.12174
CLIP4	-0.17	7.12	-1.57	0.12174
KLC3	0.35	6.78	1.57	0.12176
RFC5	-0.12	6.76	-1.57	0.12177
AL138756.1	-0.15	7.61	-1.57	0.12182
SCART1	-0.24	5.74	-1.57	0.12191
ZNF232	0.12	6.60	1.57	0.12193
AL353625.1	-0.24	6.23	-1.57	0.12195
AZIN1	-0.16	8.90	-1.57	0.12195
CIDECP1	0.14	8.10	1.57	0.12199
PARP11	-0.14	6.68	-1.57	0.12210
AP001107.9	0.20	5.13	1.57	0.12210
MEA1	0.14	9.56	1.57	0.12216
TARS2	0.09	8.60	1.57	0.12218
MVB12A	0.13	8.84	1.57	0.12219
FBXO22	-0.13	6.94	-1.57	0.12227
VPS25	0.12	9.13	1.57	0.12229
KIF13B	-0.10	8.59	-1.57	0.12231
ST6GALNAC2	-0.28	7.11	-1.57	0.12241
TJAP1	0.11	9.11	1.57	0.12241
SH3TC2	-0.22	5.52	-1.57	0.12243
CD44	-0.10	12.15	-1.57	0.12246
CEP135	-0.15	6.82	-1.57	0.12249
UBTD2	-0.15	6.37	-1.57	0.12256
C5orf15	-0.12	8.26	-1.57	0.12264
NFYA	-0.14	8.34	-1.57	0.12270
AL590617.1	-0.17	5.45	-1.57	0.12274
RPL30P4	0.22	9.20	1.57	0.12282
AC108134.3	-0.23	8.26	-1.57	0.12289
FIGNL1	-0.18	5.68	-1.57	0.12290

HNRNPH1	-0.14	11.10	-1.57	0.12292
POLB	-0.15	8.56	-1.57	0.12301
RERE-AS1	-0.24	6.70	-1.57	0.12303
AL450998.1	0.25	7.82	1.57	0.12311
EMC4	0.10	8.83	1.57	0.12312
ZNF787	0.17	8.91	1.57	0.12314
UBE2D1	-0.26	8.66	-1.57	0.12318
RUBCNL	-0.18	9.68	-1.57	0.12322
SLC22A16	-0.21	5.29	-1.57	0.12322
CD40LG	0.19	8.30	1.57	0.12325
WDPCP	0.16	6.29	1.57	0.12329
TMEM161A	0.14	7.82	1.57	0.12331
TRPS1	-0.15	6.86	-1.57	0.12337
CLIC3	0.25	8.50	1.57	0.12339
CLCN6	-0.15	8.10	-1.57	0.12340
BX322784.1	-0.16	5.48	-1.57	0.12342
CDCA7	-0.16	5.26	-1.57	0.12343
TNPO1	-0.13	8.32	-1.57	0.12352
DANCR	0.14	8.11	1.57	0.12354
TLE2	0.21	7.26	1.57	0.12360
AC016957.2	0.18	7.42	1.56	0.12368
BCAP31	0.14	11.61	1.56	0.12369
NIPSNAP3A	-0.14	6.88	-1.56	0.12380
AC099789.1	0.22	5.88	1.56	0.12381
CCDC126	-0.20	6.16	-1.56	0.12387
BET1L	0.09	10.50	1.56	0.12394
KIF1C-AS1	0.20	5.20	1.56	0.12404
INTS8	-0.12	8.20	-1.56	0.12408
CCNYL1	-0.16	6.77	-1.56	0.12411
AC019069.1	0.17	5.56	1.56	0.12414
AC093525.8	-0.18	5.63	-1.56	0.12428
HELQ	-0.13	7.16	-1.56	0.12428
AHSA2P	-0.24	9.25	-1.56	0.12436
ATP13A2	0.15	9.85	1.56	0.12437
SLC26A8	-0.28	6.74	-1.56	0.12442
FAM169A	-0.18	5.68	-1.56	0.12444
PANK4	0.08	9.54	1.56	0.12451
VCPKMT	-0.19	7.53	-1.56	0.12457
NDUFB5	0.11	8.13	1.56	0.12460
KDSR	0.13	7.85	1.56	0.12467
RFX5	-0.11	9.34	-1.56	0.12470
C2CD3	-0.10	8.43	-1.56	0.12481
MALAT1	0.32	14.05	1.56	0.12482
CLTA	0.16	10.06	1.56	0.12491
PPP1R9B	0.11	12.13	1.56	0.12502
CMKLR1	-0.23	8.84	-1.56	0.12503
CORO1A	0.12	14.65	1.56	0.12505
NLE1	0.15	7.89	1.56	0.12516
AP002387.2	0.22	7.17	1.56	0.12525
DAP3P2	0.19	5.17	1.56	0.12537

MAPK11	0.16	6.46	1.56	0.12540
CHKB-CPT1B	-0.16	5.01	-1.56	0.12544
RANBP1	0.13	8.88	1.56	0.12554
MTRR	-0.16	7.35	-1.56	0.12562
ZBTB44	-0.12	8.96	-1.56	0.12563
SLC22A17	0.24	6.39	1.56	0.12569
ZKSCAN2-DT	-0.18	5.48	-1.56	0.12570
NCOA4P3	-0.23	5.14	-1.56	0.12573
ADIPOR2	-0.09	9.66	-1.56	0.12573
CARHSP1	0.12	9.74	1.56	0.12576
AC002316.1	-0.15	10.74	-1.56	0.12589
CERCAM	0.26	6.30	1.56	0.12591
TMC8	0.10	12.28	1.56	0.12592
MYBL2	0.29	7.11	1.56	0.12599
HOOK2	0.17	7.54	1.55	0.12617
MKNK1	-0.25	10.12	-1.55	0.12617
WWC3	-0.17	9.98	-1.55	0.12620
PHTF1	-0.22	7.25	-1.55	0.12626
FXR2	0.10	9.38	1.55	0.12631
CRTAM	-0.16	7.26	-1.55	0.12641
ZNF268	0.16	7.09	1.55	0.12648
PPFIA4	-0.24	5.47	-1.55	0.12649
GLT1D1	-0.24	11.02	-1.55	0.12662
RPSAP19	0.21	7.91	1.55	0.12664
MRPS16	0.12	8.62	1.55	0.12666
AP3S2	0.12	8.29	1.55	0.12666
CNFN	0.18	5.02	1.55	0.12676
VPS37A	-0.13	7.05	-1.55	0.12697
ATXN1	-0.15	8.98	-1.55	0.12697
ASH1L	-0.19	8.74	-1.55	0.12703
VAC14	0.10	9.30	1.55	0.12703
ARHGAP19	-0.15	8.45	-1.55	0.12705
SPAG1	-0.17	5.59	-1.55	0.12709
BSG	0.21	13.04	1.55	0.12716
UQCR10	0.15	9.60	1.55	0.12716
PIDD1	0.19	8.01	1.55	0.12718
POLR2G	0.11	9.71	1.55	0.12735
AC006504.5	0.11	6.70	1.55	0.12740
YARS1	0.10	10.28	1.55	0.12742
DNAJB6	-0.11	9.82	-1.55	0.12766
DNAJC10	-0.12	8.48	-1.55	0.12767
ANKRD40	-0.13	8.15	-1.55	0.12767
EFNA3	0.26	5.71	1.55	0.12768
SLFN12	-0.17	6.18	-1.55	0.12770
SIRPB2	-0.23	10.35	-1.55	0.12774
RACGAP1	-0.15	6.72	-1.55	0.12774
AC010247.2	0.18	7.12	1.55	0.12775
B3GNT2	-0.22	7.36	-1.55	0.12779
NSMCE3	0.11	8.64	1.55	0.12786
RNF217	-0.17	5.23	-1.55	0.12792

PLBD1	-0.22	10.90	-1.55	0.12793
GRASP	0.17	6.63	1.55	0.12794
TYW1	-0.11	7.51	-1.55	0.12794
NORAD	-0.11	10.45	-1.55	0.12799
ZNF17	-0.14	5.87	-1.55	0.12808
AL158823.1	0.26	7.04	1.55	0.12810
SEPTIN11	-0.13	7.81	-1.55	0.12813
WASL	-0.15	6.02	-1.55	0.12816
AL034379.1	0.14	5.96	1.55	0.12818
STX18	0.09	8.36	1.55	0.12819
ZBTB40	-0.17	9.06	-1.55	0.12820
GPRIN3	-0.15	8.00	-1.55	0.12825
CDV3	-0.09	10.74	-1.55	0.12829
IFI27L1	0.14	6.10	1.55	0.12829
AL355388.1	-0.21	5.70	-1.55	0.12836
BMX	-0.34	6.28	-1.55	0.12839
SLC16A1-AS1	0.25	6.58	1.55	0.12844
PLEKHA6	0.17	5.07	1.54	0.12845
LRRC56	0.17	5.66	1.54	0.12848
DDI2	-0.10	8.19	-1.54	0.12850
MED30	-0.12	7.33	-1.54	0.12853
CD72	0.31	8.44	1.54	0.12861
HSPB1P1	0.17	5.58	1.54	0.12862
SIRPAP1	-0.33	6.11	-1.54	0.12866
ATP8B3	-0.17	6.30	-1.54	0.12867
TTN-AS1	-0.16	7.30	-1.54	0.12876
HES1	0.20	5.86	1.54	0.12879
COG2	0.11	8.69	1.54	0.12880
AC009118.3	0.15	5.19	1.54	0.12882
MLYCD	0.09	8.34	1.54	0.12883
MYO1E	-0.19	7.13	-1.54	0.12888
AC027279.1	0.22	5.08	1.54	0.12893
STIMATE-MUSTN1	-0.22	5.10	-1.54	0.12900
HBQ1	0.36	7.74	1.54	0.12905
AL645933.4	-0.21	5.59	-1.54	0.12910
RMND5B	0.09	8.98	1.54	0.12921
PPARA	-0.12	7.32	-1.54	0.12921
AC104530.1	0.15	9.45	1.54	0.12921
ZMYND19	0.12	7.54	1.54	0.12922
PFKFB2	-0.38	7.15	-1.54	0.12924
TSPYL2	0.16	9.12	1.54	0.12929
DCUN1D1	-0.17	7.78	-1.54	0.12939
ZNF701	-0.12	7.26	-1.54	0.12943
IGHV2-70D	0.29	5.18	1.54	0.12954
EXT1	-0.14	7.90	-1.54	0.12956
AL445426.1	-0.20	5.25	-1.54	0.12980
LINC01521	0.19	5.18	1.54	0.12982
CMTM1	-0.25	5.70	-1.54	0.12983
SWI5	0.15	7.80	1.54	0.12985
UBR3	-0.13	7.19	-1.54	0.12986

ANO9	0.24	9.22	1.54	0.12989
PEPD	0.13	9.56	1.54	0.12990
IL23A	0.19	6.56	1.54	0.12996
RPE	-0.11	6.62	-1.54	0.12997
ADNP	-0.11	9.80	-1.54	0.12998
MARCHF6	-0.12	9.73	-1.54	0.12998
CIC	0.12	11.06	1.54	0.13004
ZMIZ2	0.14	10.96	1.54	0.13006
STXBP4	-0.15	5.20	-1.54	0.13007
CHAC2	-0.16	5.08	-1.54	0.13013
RBM12	-0.10	9.08	-1.54	0.13025
ATP5MG	0.14	8.08	1.54	0.13047
ZNF638	-0.11	9.21	-1.54	0.13048
ISOC1	-0.15	6.76	-1.54	0.13057
ANXA5	-0.14	10.89	-1.54	0.13062
GALT	0.15	8.77	1.54	0.13070
FCRLB	0.16	5.40	1.54	0.13072
CNPY3	0.17	12.48	1.54	0.13075
TBRG1	-0.12	9.37	-1.54	0.13075
TRGV7	-0.20	5.24	-1.54	0.13079
FAUP1	0.23	8.27	1.54	0.13085
GALNT12	0.16	6.50	1.54	0.13087
KIF11	-0.18	5.35	-1.53	0.13099
CD96	0.14	9.97	1.53	0.13106
MAN2A2	-0.24	10.93	-1.53	0.13118
ZDHHC7	-0.10	10.72	-1.53	0.13122
TMEM62	-0.14	7.39	-1.53	0.13130
CMTM7	0.12	9.86	1.53	0.13140
SGO1	0.21	5.19	1.53	0.13151
N4BP1	-0.14	10.74	-1.53	0.13153
SLC25A22	0.15	8.18	1.53	0.13169
SUSD1	-0.14	8.96	-1.53	0.13171
KRT10	0.15	7.30	1.53	0.13180
KTI12	0.15	6.54	1.53	0.13183
HNRNPCP4	-0.13	5.37	-1.53	0.13200
ROM1	0.16	5.19	1.53	0.13201
UTP6	-0.10	8.98	-1.53	0.13203
TMEM165	-0.16	8.45	-1.53	0.13212
NPLOC4	0.08	10.84	1.53	0.13219
AC139530.3	0.15	5.04	1.53	0.13221
CFL2	-0.17	5.77	-1.53	0.13226
AC005632.3	-0.18	6.18	-1.53	0.13228
COMT	0.14	9.80	1.53	0.13235
PTDSS2	0.15	7.98	1.53	0.13237
THAP12	-0.13	8.42	-1.53	0.13240
RPSAP4	0.12	5.07	1.53	0.13249
GSTP1	0.21	11.34	1.53	0.13250
BBS7	-0.13	5.73	-1.53	0.13256
BBS12	-0.15	5.61	-1.53	0.13258
FKBP14	-0.16	5.39	-1.53	0.13266

NLK	-0.11	7.12	-1.53	0.13271
NF2	0.11	8.97	1.53	0.13271
PLRG1	-0.09	8.37	-1.53	0.13273
NAIP	-0.28	7.54	-1.53	0.13274
SMAD2	-0.10	9.06	-1.53	0.13278
NCOA7	-0.16	7.57	-1.53	0.13278
S100A11P1	-0.19	5.87	-1.53	0.13283
LINC01597	0.19	4.89	1.53	0.13290
AC114778.2	-0.23	5.44	-1.53	0.13291
NCKAP1	-0.18	5.44	-1.53	0.13293
LGALS3	0.25	9.90	1.53	0.13296
NHP2P1	0.15	5.47	1.53	0.13297
AC100835.2	0.38	6.95	1.53	0.13300
PGK1	-0.12	12.18	-1.53	0.13301
FO538757.1	-0.25	4.98	-1.53	0.13317
MYL6	0.17	12.95	1.53	0.13320
MAP4K5	-0.17	6.63	-1.53	0.13324
E2F3	-0.21	8.28	-1.53	0.13325
MRVI1	-0.32	9.62	-1.53	0.13326
NLRC5	-0.17	11.78	-1.52	0.13354
TMEM51	-0.27	5.63	-1.52	0.13354
SLC46A3	-0.12	8.15	-1.52	0.13356
TIMELESS	-0.14	7.54	-1.52	0.13357
NRM	0.19	8.89	1.52	0.13359
DENND6A	-0.14	7.31	-1.52	0.13365
STX4	0.11	10.26	1.52	0.13366
VPS4A	0.13	8.92	1.52	0.13366
LSP1P3	0.19	5.50	1.52	0.13378
PPT1	-0.20	11.00	-1.52	0.13380
GABARAPL1	-0.19	9.75	-1.52	0.13383
TRAF3IP2-AS1	-0.15	5.46	-1.52	0.13385
SNAPC4	0.12	8.21	1.52	0.13388
ITPRIPL2	-0.15	7.43	-1.52	0.13392
PTOV1	0.14	10.85	1.52	0.13396
NEGR1	0.20	5.23	1.52	0.13400
TRAV41	-0.21	5.46	-1.52	0.13403
EIPR1	0.13	8.58	1.52	0.13412
CD163	-0.25	9.99	-1.52	0.13413
KCND1	-0.19	6.68	-1.52	0.13415
CCDC25	0.13	8.05	1.52	0.13418
ITGB4	0.28	6.32	1.52	0.13419
PIP4K2C	-0.09	8.88	-1.52	0.13423
HMBS	0.24	8.40	1.52	0.13431
ROCK2	-0.11	7.72	-1.52	0.13431
UQCRFS1	0.13	8.90	1.52	0.13433
PTPN22	-0.18	7.61	-1.52	0.13447
RPS4XP1	0.18	6.21	1.52	0.13453
EEA1	-0.14	6.16	-1.52	0.13454
NSD2	-0.11	8.83	-1.52	0.13455
AL159141.1	0.12	12.41	1.52	0.13462

RIC3	0.22	6.44	1.52	0.13480
LDB1	0.08	11.49	1.52	0.13481
CTDSPL	0.25	7.98	1.52	0.13481
TGIF2	0.10	8.83	1.52	0.13486
NAP1L4	0.10	11.15	1.52	0.13494
MRC2	0.32	7.71	1.52	0.13503
ECE1-AS1	-0.16	5.46	-1.52	0.13507
AC096667.1	-0.16	6.47	-1.52	0.13524
KAT7	-0.07	9.44	-1.52	0.13529
ZNF175	-0.16	6.44	-1.52	0.13529
IL18BP	0.14	8.37	1.52	0.13530
TLK2	-0.11	8.52	-1.52	0.13535
ANKRD27	-0.08	8.65	-1.52	0.13535
GART	-0.11	8.63	-1.52	0.13541
AC074019.1	-0.15	4.96	-1.52	0.13545
ASB6	0.08	9.08	1.52	0.13562
GRK6	0.11	12.51	1.52	0.13564
SBDSP1	-0.17	6.59	-1.52	0.13566
TRIM66	-0.21	7.72	-1.52	0.13566
LRP8	-0.17	6.45	-1.52	0.13573
RPL10P2	0.15	5.32	1.51	0.13588
SPATA13	-0.18	9.28	-1.51	0.13591
GPN2	0.10	8.81	1.51	0.13619
SIGLEC16	-0.19	5.86	-1.51	0.13620
UCHL5	-0.12	7.22	-1.51	0.13621
UFD1	0.09	9.42	1.51	0.13624
RPL22P1	0.23	8.84	1.51	0.13627
ZNF783	0.11	8.42	1.51	0.13631
XRCC5	-0.09	11.24	-1.51	0.13633
OSBPL9	-0.17	8.03	-1.51	0.13636
RBM38	0.31	12.31	1.51	0.13636
STXBP5-AS1	-0.18	5.40	-1.51	0.13637
MTMR1	-0.14	9.01	-1.51	0.13640
RPL27AP5	0.14	5.11	1.51	0.13640
RPS4XP22	-0.40	5.50	-1.51	0.13646
CNOT7	-0.11	8.41	-1.51	0.13649
C2	-0.25	5.44	-1.51	0.13651
TAF4	-0.13	8.16	-1.51	0.13653
SLC38A1	-0.19	9.14	-1.51	0.13663
ZNF136	-0.14	6.86	-1.51	0.13666
RUVBL1	0.13	9.05	1.51	0.13667
IGHV4-4	0.36	6.66	1.51	0.13667
TRBV18	0.19	6.42	1.51	0.13668
DNAJC2	0.24	8.95	1.51	0.13671
LRRC8D	-0.12	8.46	-1.51	0.13672
XIAP	0.14	9.44	1.51	0.13675
MERTK	-0.23	6.06	-1.51	0.13676
ALOX5	-0.22	12.56	-1.51	0.13694
NEU3	-0.11	7.47	-1.51	0.13699
BHLHE41	-0.19	5.19	-1.51	0.13701

TRAV1-2	0.16	6.03	1.51	0.13704
TM2D3	-0.08	8.93	-1.51	0.13708
CASP8AP2	-0.19	6.84	-1.51	0.13714
ZNF480	0.11	7.50	1.51	0.13717
GPT2	0.18	6.20	1.51	0.13735
PGM2	-0.16	7.96	-1.51	0.13746
AL355472.1	0.18	6.73	1.51	0.13746
AL391988.1	0.18	5.16	1.51	0.13747
RPL7AP30	0.21	7.58	1.51	0.13748
AC020915.3	0.17	5.79	1.51	0.13754
CAT	-0.18	11.02	-1.51	0.13755
SLC35F6	0.12	9.15	1.51	0.13757
ALS2	-0.13	6.90	-1.51	0.13758
LINC01002	-0.33	7.96	-1.51	0.13762
RPS27P29	0.14	5.45	1.51	0.13769
DOCK10	-0.16	9.77	-1.51	0.13771
RPL31P49	0.24	7.07	1.51	0.13771
LINC02611	0.14	6.13	1.51	0.13772
ITPA	0.14	8.65	1.51	0.13775
CCDC77	-0.12	6.94	-1.51	0.13776
DEDD	-0.09	9.50	-1.51	0.13779
DPP3	0.10	8.81	1.51	0.13810
AOAH	-0.15	11.63	-1.51	0.13810
GAS5	0.16	9.74	1.51	0.13811
PUS7	-0.15	5.23	-1.51	0.13813
STK19	0.12	7.60	1.51	0.13815
VPS53	0.11	9.62	1.51	0.13815
MFSD14C	-0.13	7.89	-1.51	0.13819
TSSK6	0.15	6.27	1.51	0.13827
VIPR2	-0.18	5.11	-1.51	0.13832
C1orf50	0.15	5.50	1.50	0.13847
F8	-0.16	5.83	-1.50	0.13849
GNL3L	0.18	9.33	1.50	0.13852
TNFAIP8L2	0.12	10.37	1.50	0.13865
ATP2B1	-0.11	9.29	-1.50	0.13866
EMP3	0.18	12.55	1.50	0.13866
ARF4	-0.11	8.99	-1.50	0.13870
TGFBRAP1	-0.12	8.34	-1.50	0.13871
FAM210B	0.34	12.10	1.50	0.13872
CRBN	-0.12	8.30	-1.50	0.13886
SLC27A5	0.16	6.24	1.50	0.13887
POLR2J4	0.12	7.25	1.50	0.13888
TOR2A	0.13	8.99	1.50	0.13890
SRPK2	-0.11	9.29	-1.50	0.13904
SNHG11	0.15	6.56	1.50	0.13905
DMWD	0.12	7.91	1.50	0.13915
EDC3	0.10	8.91	1.50	0.13926
PSMC4	0.10	10.11	1.50	0.13935
STX7	-0.15	9.43	-1.50	0.13945
PUDP	0.16	7.78	1.50	0.13954

RPL12P1	0.13	5.27	1.50	0.13957
RPL19P16	0.14	5.52	1.50	0.13963
AC093151.5	-0.24	5.69	-1.50	0.13966
SNHG9	0.19	6.64	1.50	0.13968
RPL10P12	0.19	6.97	1.50	0.13969
ZNF844	0.16	6.79	1.50	0.13973
POLH	0.17	8.08	1.50	0.13977
LY75	-0.14	7.79	-1.50	0.13980
RPL34P27	0.19	6.54	1.50	0.13983
RPS3A	0.20	12.23	1.50	0.13984
ACOX1	-0.17	10.13	-1.50	0.13988
RPL10P16	0.23	9.89	1.50	0.14008
AC117382.1	-0.16	5.47	-1.50	0.14011
ZEB1	-0.16	6.98	-1.50	0.14015
NUBP1	0.14	8.94	1.50	0.14019
SLC25A44	-0.18	9.87	-1.50	0.14020
MICALL1	0.11	9.04	1.50	0.14030
KIF27	-0.26	6.04	-1.50	0.14033
SLC4A10	-0.25	5.16	-1.50	0.14034
STXBP2	0.17	11.51	1.50	0.14041
AC079331.2	0.21	5.71	1.50	0.14044
ME2	-0.10	9.08	-1.50	0.14049
AC044787.1	0.20	7.31	1.50	0.14055
PPP1R35	0.14	9.21	1.50	0.14056
AC010326.5	0.20	7.29	1.50	0.14064
XYLT1	-0.17	8.34	-1.50	0.14070
NOTCH1	-0.20	11.91	-1.50	0.14073
MYO6	-0.20	5.99	-1.50	0.14073
PTEN	-0.19	10.71	-1.50	0.14074
GSTZ1	0.15	7.30	1.50	0.14074
GOLGB1	-0.09	9.75	-1.50	0.14077
SESN2	0.14	8.64	1.50	0.14081
CIAPIN1	0.11	9.24	1.50	0.14085
PGAP6	0.14	11.11	1.50	0.14085
HID1	0.20	6.26	1.50	0.14086
SMIM29	0.14	8.92	1.50	0.14086
PCBP1-AS1	-0.17	9.08	-1.50	0.14087
EIF4A1	-0.21	5.65	-1.50	0.14094
SNRPE	0.16	7.55	1.50	0.14097
SMAD4	-0.09	8.97	-1.49	0.14101
DACH1	-0.21	5.30	-1.49	0.14102
AKAP8	-0.10	8.72	-1.49	0.14114
NAPRT	0.17	9.00	1.49	0.14114
SFXN2	0.15	6.43	1.49	0.14115
AC116049.1	-0.26	6.59	-1.49	0.14117
RHBDD3	0.12	6.65	1.49	0.14130
FAM86DP	0.16	6.56	1.49	0.14140
C12orf45	0.16	7.35	1.49	0.14141
ZDHHC4	0.14	8.36	1.49	0.14149
EOGT	-0.15	5.75	-1.49	0.14153

MKS1	0.13	7.36	1.49	0.14158
VPS72	0.10	9.26	1.49	0.14161
EPAS1	-0.17	5.87	-1.49	0.14170
AASDHPPT	-0.13	7.22	-1.49	0.14178
AC104695.3	-0.18	5.63	-1.49	0.14188
BRCA2	-0.17	5.12	-1.49	0.14189
RPL11P5	0.23	7.14	1.49	0.14190
C22orf46	0.13	7.59	1.49	0.14195
YY1	0.08	10.79	1.49	0.14197
MITD1	-0.12	7.80	-1.49	0.14203
MFGE8	0.16	7.71	1.49	0.14217
PRR12	0.12	9.70	1.49	0.14226
ZNF83	-0.18	7.56	-1.49	0.14227
ITM2C	0.23	9.80	1.49	0.14229
AP003733.3	0.23	5.48	1.49	0.14229
SLX4IP	-0.15	6.17	-1.49	0.14244
RANGRF	0.14	8.64	1.49	0.14253
THAP5	-0.14	7.13	-1.49	0.14253
PLEKHA8	-0.15	6.29	-1.49	0.14264
MDN1	-0.15	8.62	-1.49	0.14280
TMTC2	-0.14	6.16	-1.49	0.14281
ZNF654	-0.16	6.26	-1.49	0.14302
AC002456.1	-0.16	4.96	-1.49	0.14307
PI4KAP2	-0.21	7.23	-1.49	0.14311
BBIP1	-0.15	7.63	-1.49	0.14311
MAPKAPK5	-0.08	8.15	-1.49	0.14324
GAB3	-0.13	9.41	-1.49	0.14324
ARHGEF7	-0.09	9.15	-1.49	0.14326
FEM1A	0.08	9.52	1.49	0.14328
RPL7AP6	0.22	9.23	1.49	0.14337
ACVR2A	-0.13	6.38	-1.49	0.14342
RHOT2	0.15	10.63	1.49	0.14345
TRBV5-6	0.19	6.63	1.49	0.14351
RPSAP9	0.14	5.84	1.48	0.14362
LINC01011	0.13	6.71	1.48	0.14363
ATRX	0.19	9.44	1.48	0.14368
ZNF236-DT	0.20	5.35	1.48	0.14373
AGO2	-0.16	9.63	-1.48	0.14378
LAIR2	0.38	6.14	1.48	0.14382
AC092053.6	-0.21	5.52	-1.48	0.14382
HBG2	0.76	7.34	1.48	0.14383
AC009245.1	0.19	7.97	1.48	0.14383
RPS23P1	0.19	5.72	1.48	0.14389
CDC23	-0.12	7.49	-1.48	0.14389
EXOSC7	0.14	7.98	1.48	0.14391
CCDC88A	0.19	8.71	1.48	0.14393
LINC01006	-0.17	5.42	-1.48	0.14405
PRRC2C	0.20	11.50	1.48	0.14411
PDLIM2	0.09	10.01	1.48	0.14421
TYSND1	0.12	8.67	1.48	0.14425

ALOX12	0.24	7.71	1.48	0.14431
BCLAF1P2	-0.19	5.30	-1.48	0.14432
NMD3	-0.13	7.16	-1.48	0.14436
AL359762.1	-0.24	6.54	-1.48	0.14444
ZADH2	-0.10	8.30	-1.48	0.14447
AC084871.1	0.27	5.37	1.48	0.14448
GBA2	-0.11	10.18	-1.48	0.14449
DEPTOR	0.16	4.99	1.48	0.14466
BORCS5	0.10	7.25	1.48	0.14476
MTF1	-0.12	8.76	-1.48	0.14477
PTMA	0.12	12.89	1.48	0.14482
APTX	0.09	7.77	1.48	0.14501
MRPS36	0.14	7.16	1.48	0.14502
SLC38A10	0.11	10.90	1.48	0.14505
SIRT5	-0.13	7.57	-1.48	0.14518
TRBV7-2	0.26	8.34	1.48	0.14537
ZNF577	-0.17	5.98	-1.48	0.14542
SMPDL3A	-0.13	5.89	-1.48	0.14546
SNRK-AS1	-0.18	5.09	-1.48	0.14553
RPL11P3	0.21	8.52	1.48	0.14559
MAST2	0.12	8.15	1.48	0.14571
AKAP12	-0.22	5.44	-1.48	0.14572
LINC-PINT	0.24	6.87	1.48	0.14574
AC107375.1	0.18	5.85	1.48	0.14580
TXNIP	-0.13	15.08	-1.48	0.14580
AC092597.1	0.14	6.38	1.48	0.14584
GRK4	-0.18	5.19	-1.48	0.14592
MASTL	-0.16	5.90	-1.48	0.14606
NKTR	-0.18	9.34	-1.48	0.14607
NOL9	0.15	8.32	1.48	0.14610
HYI	0.15	7.19	1.48	0.14612
GTF2H3	0.12	7.43	1.48	0.14615
OSBPL8	-0.21	8.66	-1.48	0.14617
RPL23AP18	0.12	5.25	1.47	0.14641
ADA2	-0.14	12.02	-1.47	0.14644
RBM3	0.10	10.93	1.47	0.14645
ZZEF1	-0.10	10.98	-1.47	0.14646
C11orf24	0.14	8.33	1.47	0.14650
MEG3	-0.40	5.95	-1.47	0.14651
SLC25A11	0.11	10.13	1.47	0.14662
KLF5	-0.22	5.48	-1.47	0.14666
ZNF37BP	-0.18	7.16	-1.47	0.14666
FAM78A	0.09	11.62	1.47	0.14666
COX7CP1	0.20	6.32	1.47	0.14668
SRSF10	-0.10	8.52	-1.47	0.14689
WDR54	0.15	8.13	1.47	0.14692
MRPS18C	0.12	7.09	1.47	0.14697
VWA8	-0.18	6.69	-1.47	0.14700
COQ8A	0.11	10.47	1.47	0.14705
EVI2A	-0.13	8.95	-1.47	0.14717

GOLIM4	-0.18	6.13	-1.47	0.14720
AC097382.3	0.14	6.70	1.47	0.14721
ZNHIT3	0.10	8.47	1.47	0.14734
AGPAT2	0.16	9.19	1.47	0.14736
LIMD1	-0.13	8.63	-1.47	0.14742
CD99L2	0.09	9.40	1.47	0.14752
SLC35C1	0.11	8.91	1.47	0.14753
CCDC15	0.15	5.48	1.47	0.14756
PLD4	0.24	9.03	1.47	0.14768
NAPSA	0.17	5.61	1.47	0.14769
TOP2B	-0.12	9.96	-1.47	0.14777
ZNF672	0.10	9.74	1.47	0.14780
PIK3CDP1	-0.21	5.90	-1.47	0.14781
FOXRED1	0.12	8.25	1.47	0.14792
IRF9	-0.23	8.08	-1.47	0.14793
GCNT2	-0.21	6.49	-1.47	0.14800
TCAF2	-0.16	6.73	-1.47	0.14804
RNF19B	-0.15	10.67	-1.47	0.14811
NVL	0.10	7.95	1.47	0.14822
C8orf76	0.13	6.29	1.47	0.14825
HCAR2	-0.32	10.00	-1.47	0.14837
AC005842.1	0.21	6.74	1.47	0.14847
PRICKLE1	-0.24	5.88	-1.47	0.14849
AC009005.1	-0.20	5.64	-1.47	0.14849
SGMS2	-0.17	6.05	-1.47	0.14850
MFSD3	0.17	6.39	1.47	0.14852
AC010969.2	0.13	6.48	1.47	0.14863
H2AC20	0.15	5.19	1.47	0.14865
CMTM5	0.30	7.64	1.47	0.14866
AL359263.1	-0.19	5.23	-1.47	0.14866
ZFP62	-0.13	7.25	-1.47	0.14870
HMGB3	-0.18	5.34	-1.47	0.14870
LZTFL1	-0.15	6.19	-1.47	0.14875
NDUFB2	0.15	9.21	1.47	0.14877
GRB10	-0.32	7.21	-1.47	0.14879
ECM1	0.18	5.36	1.47	0.14884
COX10	0.12	7.36	1.47	0.14897
SEM1	0.15	8.43	1.46	0.14901
PHC1	-0.16	6.87	-1.46	0.14909
CCDC14	-0.17	6.64	-1.46	0.14911
CYB5RL	-0.10	6.31	-1.46	0.14915
SRSF1	-0.12	7.84	-1.46	0.14915
CBX7	0.10	10.80	1.46	0.14927
ELMOD2	-0.14	6.88	-1.46	0.14936
PMM1	0.13	8.64	1.46	0.14938
LINC02723	-0.19	6.77	-1.46	0.14941
PDPR	-0.16	9.32	-1.46	0.14956
DBR1	-0.13	7.04	-1.46	0.14957
GHDC	0.12	9.02	1.46	0.14964
ZNF287	0.17	5.98	1.46	0.14972

TNFAIP2	-0.19	12.95	-1.46	0.14978
UBE2J1	-0.18	9.53	-1.46	0.14982
AL512599.1	0.23	7.12	1.46	0.14986
SUPT16HP1	-0.18	5.88	-1.46	0.14989
MRPL39	0.12	7.01	1.46	0.14990
ERLEC1	-0.12	7.95	-1.46	0.15009
COQ8B	0.12	9.06	1.46	0.15021
AC117409.1	0.16	5.36	1.46	0.15023
C16orf58	0.10	9.45	1.46	0.15026
PDE9A	0.21	6.38	1.46	0.15028
KCTD6	-0.09	6.69	-1.46	0.15033
CTBS	-0.21	8.07	-1.46	0.15035
ZNF587	-0.23	6.84	-1.46	0.15038
TAPBP	0.12	13.06	1.46	0.15039
EFCAB7	-0.16	5.22	-1.46	0.15044
TUBG2	0.16	6.89	1.46	0.15058
SRSF2	-0.09	10.05	-1.46	0.15070
TPX2	-0.15	5.74	-1.46	0.15072
PLD3	0.14	11.10	1.46	0.15076
AC138969.3	0.25	5.29	1.46	0.15084
CD38	-0.21	7.32	-1.46	0.15097
COPZ1	0.11	10.58	1.46	0.15099
SLC22A5	-0.17	6.10	-1.46	0.15112
TRAF7	0.09	10.35	1.46	0.15116
GPBP1	-0.09	10.00	-1.46	0.15130
MTRES1	0.13	6.49	1.46	0.15131
ADAM28	-0.18	7.44	-1.46	0.15135
AC115223.1	0.21	9.10	1.46	0.15139
RPP40	0.14	5.43	1.46	0.15151
RNF40	0.09	11.20	1.46	0.15158
NDUFAF2	0.16	6.61	1.46	0.15166
UTS2	-0.29	5.21	-1.45	0.15172
SIDT1	-0.13	8.53	-1.45	0.15173
STX2	-0.16	6.93	-1.45	0.15185
FNBP4	-0.14	9.05	-1.45	0.15192
NDUFA1	0.15	9.58	1.45	0.15197
RNF122	-0.11	8.62	-1.45	0.15199
IGKJ1	0.25	6.11	1.45	0.15201
AC131206.1	0.16	5.11	1.45	0.15203
PMPCA	0.09	9.43	1.45	0.15204
GLG1	0.07	11.90	1.45	0.15204
RTP4	-0.23	7.50	-1.45	0.15205
B4GALNT4	-0.22	5.13	-1.45	0.15209
ENTPD2	-0.28	5.75	-1.45	0.15212
CHFR	-0.10	9.62	-1.45	0.15214
ZGRF1	-0.15	5.57	-1.45	0.15216
AMZ2P1	-0.14	6.93	-1.45	0.15216
HNRNPA1P50	0.12	5.05	1.45	0.15218
RPL12P38	0.13	6.26	1.45	0.15221
SFT2D2	-0.11	9.43	-1.45	0.15221

POMT1	0.12	8.63	1.45	0.15226
SNX17	0.12	11.62	1.45	0.15240
CRACR2B	0.20	6.35	1.45	0.15247
CAND1	-0.11	8.73	-1.45	0.15261
ELK1	0.09	8.22	1.45	0.15265
NEAT1	0.27	13.62	1.45	0.15268
PHF12	-0.14	10.84	-1.45	0.15275
KLHL15	-0.15	5.94	-1.45	0.15276
UFC1	0.13	10.03	1.45	0.15288
PLAA	-0.10	7.60	-1.45	0.15296
TIMM29	0.10	7.73	1.45	0.15297
LINC01963	0.11	7.19	1.45	0.15307
ABHD17A	0.15	11.16	1.45	0.15309
RFC3	-0.17	5.70	-1.45	0.15314
P4HB	0.12	12.50	1.45	0.15317
AC009404.1	0.16	5.51	1.45	0.15319
AC004941.2	-0.18	5.31	-1.45	0.15367
FOXJ2	-0.10	8.88	-1.45	0.15368
CREM	0.15	6.79	1.45	0.15384
UBR5-AS1	-0.19	7.11	-1.45	0.15388
AC131009.4	0.28	6.04	1.45	0.15391
SRP19	0.11	6.80	1.45	0.15397
CATSPER1	0.18	7.41	1.45	0.15398
SLC35D1	-0.13	7.09	-1.45	0.15400
SAMSN1	-0.22	8.21	-1.45	0.15405
TYW1B	-0.20	5.83	-1.45	0.15416
MCM7	0.09	10.44	1.45	0.15421
IKBKE	0.13	9.91	1.45	0.15424
IGHM	0.22	14.01	1.45	0.15428
C15orf40	0.09	7.93	1.45	0.15431
RPS21P4	0.21	6.62	1.45	0.15433
ERHP1	-0.14	5.15	-1.45	0.15438
SPSB2	0.17	7.62	1.45	0.15438
MRPS17	0.15	6.09	1.45	0.15446
RPS20P14	0.22	8.37	1.44	0.15458
TLR5	-0.27	7.85	-1.44	0.15473
ZC3H6	0.25	9.20	1.44	0.15485
FAHD2B	0.13	6.41	1.44	0.15485
RAD23BP1	-0.17	5.16	-1.44	0.15488
SEMA3C	-0.18	5.28	-1.44	0.15492
HIVEP2	-0.16	9.06	-1.44	0.15493
CENPP	0.16	5.65	1.44	0.15493
CDC73	-0.13	7.71	-1.44	0.15496
EBF1	0.21	5.92	1.44	0.15513
ANKIB1	-0.16	7.18	-1.44	0.15513
RPL7P23	0.16	6.59	1.44	0.15513
AL596275.1	0.11	5.44	1.44	0.15516
LINC01184	0.15	7.32	1.44	0.15522
CMSS1	0.16	6.79	1.44	0.15534
ANKRA2	-0.11	6.93	-1.44	0.15555

TRUB2	0.12	8.25	1.44	0.15556
ILK	0.11	11.37	1.44	0.15557
BST1	-0.19	10.14	-1.44	0.15558
TSEN2	0.15	6.34	1.44	0.15573
ACTN1	-0.19	12.61	-1.44	0.15579
TUBB1	-0.25	10.49	-1.44	0.15587
BRD4	0.11	11.77	1.44	0.15588
MFSD2A	-0.18	6.60	-1.44	0.15598
GLYCTK	0.12	8.12	1.44	0.15599
AMPD3	-0.16	8.82	-1.44	0.15600
HMGCS1	-0.11	7.41	-1.44	0.15601
AC092279.2	-0.27	6.41	-1.44	0.15611
RPS4XP8	0.15	5.27	1.44	0.15611
HCN2	0.15	5.88	1.44	0.15614
TRAIP	0.13	5.63	1.44	0.15616
CYP4F12	0.22	6.50	1.44	0.15623
CDC25B	0.13	11.56	1.44	0.15629
SPATS2L	-0.30	7.02	-1.44	0.15650
H3-3A	-0.23	10.25	-1.44	0.15650
CREB3L2	-0.09	8.50	-1.44	0.15655
VANGL1	-0.14	6.17	-1.44	0.15656
DOC2B	-0.24	5.05	-1.44	0.15676
AL353678.1	0.24	7.18	1.44	0.15691
ZNF580	0.13	8.51	1.44	0.15692
MRPS27	0.09	8.86	1.44	0.15698
ATL1	-0.15	5.12	-1.44	0.15698
AC021016.1	0.17	5.81	1.44	0.15704
RN7SL172P	0.23	5.36	1.44	0.15708
AL358176.3	0.15	5.11	1.44	0.15715
MAP7D3	0.12	7.19	1.44	0.15720
POLR2C	0.09	10.08	1.44	0.15721
PBX4	0.15	7.02	1.44	0.15722
SNHG32	0.15	7.65	1.43	0.15731
ITGAD	-0.21	6.01	-1.43	0.15737
TECRP2	0.15	5.37	1.43	0.15742
GSTK1	0.11	11.98	1.43	0.15742
AGBL5	0.10	7.77	1.43	0.15744
IGHV1-69	0.32	6.49	1.43	0.15744
ARHGAP31	-0.13	8.02	-1.43	0.15745
YWHAQ	-0.10	10.09	-1.43	0.15746
SLC37A3	-0.26	7.51	-1.43	0.15747
AC009487.2	-0.18	5.80	-1.43	0.15749
SNX2	-0.11	9.64	-1.43	0.15752
AC147651.4	0.13	6.22	1.43	0.15760
ABCA5	-0.16	6.47	-1.43	0.15761
POGK	-0.09	8.85	-1.43	0.15769
AP003774.2	0.17	5.61	1.43	0.15774
FRMPD3	-0.19	6.33	-1.43	0.15776
FAM172A	-0.13	6.89	-1.43	0.15780
GET3	0.10	9.88	1.43	0.15781

RUFY2	-0.15	5.79	-1.43	0.15785
ZBTB6	-0.14	6.59	-1.43	0.15798
CA11	0.17	7.37	1.43	0.15801
NCEH1	-0.17	6.30	-1.43	0.15809
AL035530.2	-0.15	5.21	-1.43	0.15810
MBNL1	-0.12	10.97	-1.43	0.15810
ADGRE3	-0.21	10.23	-1.43	0.15816
CENPM	0.16	5.88	1.43	0.15823
AFF4	-0.14	8.09	-1.43	0.15827
USP19	-0.09	10.44	-1.43	0.15829
ZNF598	0.10	9.23	1.43	0.15831
AL138785.2	0.16	6.54	1.43	0.15833
LUNAR1	-0.21	5.27	-1.43	0.15834
TRBJ2-2	0.14	5.42	1.43	0.15838
CARMIL3	-0.16	6.12	-1.43	0.15838
AC008966.1	0.14	5.24	1.43	0.15857
AC019181.1	0.15	5.40	1.43	0.15858
ASL	0.14	8.13	1.43	0.15859
C16orf72	-0.16	9.34	-1.43	0.15861
SEC61A2	-0.18	6.57	-1.43	0.15861
AHR	-0.17	7.32	-1.43	0.15864
LOXL2	-0.14	5.85	-1.43	0.15878
HNRNPU	-0.09	11.51	-1.43	0.15879
RFFL	-0.12	9.03	-1.43	0.15882
AC104619.1	0.15	6.03	1.43	0.15891
STIM1	0.07	10.75	1.43	0.15894
TMEM11	0.11	8.55	1.43	0.15895
FKBP1B	0.17	5.30	1.43	0.15896
MAPK6	-0.16	6.43	-1.43	0.15900
KDM6B	-0.21	11.72	-1.43	0.15906
LSM10	0.13	9.83	1.43	0.15908
AVEN	0.13	6.68	1.43	0.15911
GLB1	-0.10	10.02	-1.43	0.15915
SAT2	0.13	9.22	1.43	0.15916
SOCS4	-0.14	6.62	-1.43	0.15917
HSPA9P1	-0.21	5.71	-1.43	0.15917
ANKRD36C	0.23	5.99	1.43	0.15920
AL121933.2	0.19	7.41	1.43	0.15921
CABLES2	-0.12	7.76	-1.43	0.15922
TFEB	0.16	10.59	1.43	0.15926
UQCRC1	0.14	11.03	1.43	0.15929
PABPN1	0.11	9.88	1.43	0.15932
ATE1	-0.14	6.94	-1.43	0.15935
TOR1AIP2	-0.11	8.55	-1.43	0.15936
GLCCI1	-0.17	7.35	-1.43	0.15944
PLAG1	0.16	5.59	1.43	0.15948
NDUFAB1	0.14	8.16	1.43	0.15962
LTBP4	0.15	9.40	1.43	0.15963
XPNPEP3	0.11	7.14	1.43	0.15969
SRBD1	-0.14	7.36	-1.43	0.15971

MAP2K3	0.18	12.19	1.43	0.15971
RPL7AP60	0.17	4.99	1.43	0.15974
AK1	0.21	6.67	1.43	0.15975
BEST4	-0.15	5.15	-1.43	0.15985
AC011899.2	-0.20	5.98	-1.43	0.15989
SLC26A2	-0.13	6.53	-1.43	0.15990
MED27	0.13	7.96	1.43	0.15994
MYDGF	0.18	9.65	1.43	0.15996
PPARD	0.11	9.39	1.43	0.16014
RTL10	0.10	8.02	1.42	0.16020
RPS20P10	0.19	5.13	1.42	0.16021
ATP5PD	0.12	9.59	1.42	0.16026
ANKH	0.17	9.96	1.42	0.16042
DHRS4-AS1	-0.12	7.29	-1.42	0.16047
NRF1	0.07	8.91	1.42	0.16052
COASY	0.10	10.13	1.42	0.16053
CPEB4	-0.27	7.81	-1.42	0.16054
GLRX	-0.13	10.56	-1.42	0.16062
ETFA	-0.08	8.77	-1.42	0.16071
NADK	-0.20	12.87	-1.42	0.16072
MRGBP	0.11	8.38	1.42	0.16081
FAM157C	-0.34	7.87	-1.42	0.16084
INTS6	-0.13	8.12	-1.42	0.16086
FGD2	-0.17	9.99	-1.42	0.16090
RPSAP15	0.20	7.27	1.42	0.16091
CBR4	-0.11	6.99	-1.42	0.16094
LUZP1	-0.10	7.65	-1.42	0.16127
BACH2	0.22	8.73	1.42	0.16131
RPUSD3	0.09	8.57	1.42	0.16135
ARL6IP1	-0.12	8.60	-1.42	0.16143
NCOR2	0.11	10.76	1.42	0.16143
RPL23P8	0.23	8.13	1.42	0.16150
IGHA2	0.50	10.93	1.42	0.16158
PWWP3A	0.12	8.49	1.42	0.16170
EHBP1	-0.13	6.71	-1.42	0.16184
SMAD7	-0.13	8.21	-1.42	0.16185
ZNF354A	-0.19	7.12	-1.42	0.16194
SARM1	0.09	8.52	1.42	0.16201
KRTCAP2	-0.18	5.96	-1.42	0.16219
SZRD1	0.09	11.24	1.42	0.16219
DPM3	0.20	7.92	1.42	0.16236
ALPK1	-0.27	9.68	-1.42	0.16239
ZNF597	-0.09	6.19	-1.42	0.16247
TMEM129	0.10	9.06	1.42	0.16262
AC130472.1	0.16	5.04	1.42	0.16283
SIRPA	-0.19	12.04	-1.42	0.16286
PSMD7	0.08	9.52	1.42	0.16287
ATP6V0E2-AS1	0.17	6.13	1.42	0.16287
ATF6	-0.15	9.41	-1.42	0.16292
PHF20	0.10	9.88	1.42	0.16302

GRAP2	0.11	10.75	1.41	0.16311
RAB4A	0.10	8.32	1.41	0.16320
MED9	0.12	7.87	1.41	0.16330
DGUOK	0.12	9.27	1.41	0.16340
SLC9B2	-0.13	6.13	-1.41	0.16343
ATF4	0.09	11.81	1.41	0.16346
ZSCAN5A	0.14	6.18	1.41	0.16352
NT5C	0.12	8.52	1.41	0.16352
RNF170	0.11	7.14	1.41	0.16352
CGAS	-0.16	6.80	-1.41	0.16365
GFM2	-0.10	7.12	-1.41	0.16367
TBPL1	-0.12	7.30	-1.41	0.16369
RPL21P134	0.23	6.74	1.41	0.16376
LMF1	0.15	7.59	1.41	0.16384
SNRNP200	-0.08	11.76	-1.41	0.16386
CDC42BPA	-0.13	5.15	-1.41	0.16394
KIAA0556	-0.14	8.80	-1.41	0.16395
ZNF417	-0.14	6.66	-1.41	0.16402
LMTK2	-0.19	8.86	-1.41	0.16410
MIGA2	0.12	9.10	1.41	0.16426
STARD3	0.09	10.51	1.41	0.16426
ZNF487	-0.16	6.54	-1.41	0.16427
GTF3C6	0.11	7.86	1.41	0.16427
DHFR2	0.12	6.68	1.41	0.16434
HIP1R	0.14	9.04	1.41	0.16444
NDUFAF1	0.14	7.58	1.41	0.16445
HDAC4	-0.17	9.36	-1.41	0.16448
PTPN18	0.09	12.08	1.41	0.16454
SLC35E3	0.19	8.02	1.41	0.16477
MIR3142HG	-0.13	5.26	-1.41	0.16481
CAHM	0.15	5.01	1.41	0.16482
TRIM37	-0.12	7.16	-1.41	0.16491
GOLGA8A	-0.28	7.58	-1.41	0.16500
SLC16A1	-0.15	6.37	-1.41	0.16503
PIGL	-0.14	6.45	-1.41	0.16505
PRKCD	-0.14	12.04	-1.41	0.16506
ST6GALNAC1	0.24	5.77	1.41	0.16506
UBAC2	0.08	10.30	1.41	0.16513
ZFYVE16	-0.17	7.66	-1.41	0.16513
EP400	-0.10	9.74	-1.41	0.16518
TMEM267	-0.14	5.98	-1.41	0.16533
UBE2D3P2	-0.16	5.19	-1.41	0.16536
USP16	-0.11	8.32	-1.41	0.16536
HLA-K	0.25	6.78	1.41	0.16539
CREB3L4	0.14	6.72	1.41	0.16541
CD53	-0.13	12.93	-1.41	0.16546
SLC25A15	0.15	6.53	1.41	0.16555
IFI44L	-0.47	7.48	-1.41	0.16564
MT-ND3	0.12	13.03	1.41	0.16566
DOP1B	-0.12	8.76	-1.41	0.16578

AC078817.1	0.16	6.21	1.41	0.16583
SERPINF2	0.19	5.94	1.41	0.16588
LINC00892	0.18	5.91	1.41	0.16592
C4orf46	-0.11	6.00	-1.41	0.16593
METTL7A	0.13	9.66	1.41	0.16593
BMT2	-0.14	6.74	-1.40	0.16601
CA5BP1	0.14	7.46	1.40	0.16611
MTERF2	-0.14	6.18	-1.40	0.16613
ACOX3	0.13	7.42	1.40	0.16646
ZNF789	-0.14	6.25	-1.40	0.16646
NECAP2	0.10	10.85	1.40	0.16650
AL353743.1	-0.13	5.48	-1.40	0.16650
GSDMD	0.12	10.84	1.40	0.16656
CBX1	-0.10	8.61	-1.40	0.16660
LINC01534	0.16	5.55	1.40	0.16662
STAT5B	-0.16	12.12	-1.40	0.16666
NFKBID	0.14	8.92	1.40	0.16676
C12orf4	-0.13	6.63	-1.40	0.16677
ATL2	-0.14	6.99	-1.40	0.16683
TOMM34	0.12	8.42	1.40	0.16685
ADI1	0.10	9.46	1.40	0.16686
RPL7P15	0.14	5.02	1.40	0.16715
AC023906.1	0.13	5.42	1.40	0.16715
DIP2A	-0.18	9.31	-1.40	0.16727
TRBV9	0.22	6.71	1.40	0.16728
PINLYP	0.18	5.22	1.40	0.16732
IKZF3	0.13	11.24	1.40	0.16732
FAM168A	-0.10	8.72	-1.40	0.16735
AC067852.2	0.11	7.92	1.40	0.16736
LLGL2	0.16	9.24	1.40	0.16736
SNRPG	0.12	7.91	1.40	0.16739
MAGEH1	0.13	7.63	1.40	0.16744
AC093591.1	0.14	6.64	1.40	0.16762
PSMB8-AS1	-0.09	10.24	-1.40	0.16766
TRBJ2-5	0.16	5.85	1.40	0.16772
RAF1	-0.14	11.77	-1.40	0.16780
MROH1	0.12	9.18	1.40	0.16786
RYK	-0.12	7.58	-1.40	0.16788
MRPL37P1	0.21	5.07	1.40	0.16789
TMEM167B	-0.11	9.23	-1.40	0.16793
TMEM187	0.13	7.26	1.40	0.16796
RHOBTB2	0.18	8.27	1.40	0.16804
PPRC1	0.09	9.38	1.40	0.16810
TAOK3	0.13	10.99	1.40	0.16827
RPL7P19	0.11	5.45	1.40	0.16844
IL6R	-0.16	12.25	-1.40	0.16845
NPAT	-0.15	7.40	-1.40	0.16846
JADE2	0.10	10.84	1.40	0.16852
AL354707.2	0.16	5.34	1.40	0.16853
VAMP8	0.18	10.77	1.40	0.16859

PPARGC1B	-0.15	6.17	-1.40	0.16864
RAB5IF	0.19	7.13	1.40	0.16867
TRAV26-2	0.19	5.51	1.40	0.16867
FCGR1B	-0.33	7.78	-1.40	0.16871
TGFA	-0.27	7.38	-1.40	0.16892
RPS4XP16	0.24	8.00	1.40	0.16894
DVL1	0.11	8.31	1.39	0.16911
RPL4P1	0.16	5.25	1.39	0.16921
PROK2	-0.32	9.91	-1.39	0.16925
HLA-DOB	0.20	7.58	1.39	0.16933
AC127070.4	0.19	5.40	1.39	0.16958
SPEG	0.19	5.46	1.39	0.16959
ZNF253	-0.16	5.85	-1.39	0.16973
GPR132	0.11	9.50	1.39	0.16977
LSM14B	-0.09	9.28	-1.39	0.16979
ACP6	0.11	7.21	1.39	0.16986
SLC4A8	-0.15	6.13	-1.39	0.16987
STARD10	0.14	8.54	1.39	0.16992
TMCO4	0.10	8.33	1.39	0.16992
CAPN3	-0.25	6.56	-1.39	0.16999
PCP2	0.19	5.66	1.39	0.17006
ANAPC11	0.16	9.37	1.39	0.17010
RWDD2A	-0.14	5.58	-1.39	0.17011
RPL4P4	0.21	10.41	1.39	0.17014
ST8SIA6	-0.15	5.20	-1.39	0.17016
DNM1L	-0.09	7.85	-1.39	0.17017
TRAPPC3	0.09	9.55	1.39	0.17026
PHKG2	0.11	9.61	1.39	0.17030
RBPMS2	0.33	5.80	1.39	0.17039
MDS2	0.18	6.24	1.39	0.17041
HTATIP2	-0.11	8.73	-1.39	0.17056
ZNF93	0.15	6.12	1.39	0.17066
AC010343.1	0.23	9.37	1.39	0.17072
RNF138	-0.13	7.32	-1.39	0.17073
AP000919.4	-0.16	5.18	-1.39	0.17073
AL513550.1	0.15	5.91	1.39	0.17073
ARMC1	-0.13	7.14	-1.39	0.17073
AC005822.1	-0.13	5.23	-1.39	0.17081
FAM200A	-0.11	6.53	-1.39	0.17082
TMEM258	0.15	9.16	1.39	0.17090
PIGF	-0.11	6.57	-1.39	0.17093
MARC1	-0.25	7.70	-1.39	0.17104
ATG4D	0.11	9.03	1.39	0.17108
PALD1	0.16	5.49	1.39	0.17110
TSPAN18	0.14	8.94	1.39	0.17117
MATR3	-0.11	10.14	-1.39	0.17130
AL353593.1	-0.14	5.59	-1.39	0.17134
MRPL22	0.11	7.38	1.39	0.17138
PIK3CD-AS1	-0.18	5.86	-1.39	0.17141
GTF2IP4	-0.18	7.70	-1.39	0.17147

ECHDC1	-0.13	8.07	-1.39	0.17149
ZNF496	-0.13	7.84	-1.39	0.17161
AL445433.1	0.19	6.79	1.39	0.17166
OSBPL5	-0.15	9.51	-1.39	0.17176
ZNF230	-0.16	7.10	-1.39	0.17182
AC135068.8	0.17	4.98	1.39	0.17190
ZNF764	0.10	7.37	1.39	0.17192
ZNF792	0.14	7.10	1.38	0.17202
PTP4A3	0.16	8.58	1.38	0.17213
CRIPT	-0.10	7.50	-1.38	0.17216
PDIK1L	-0.13	6.28	-1.38	0.17221
REV1	-0.10	7.87	-1.38	0.17222
UBE2M	0.13	9.31	1.38	0.17232
ZNF680	-0.15	5.42	-1.38	0.17257
CNOT2-DT	0.15	6.22	1.38	0.17258
PPIB	0.17	12.24	1.38	0.17265
SMC2	-0.14	6.45	-1.38	0.17269
TPI1P1	0.17	7.85	1.38	0.17272
AC103810.3	0.17	5.12	1.38	0.17274
MYORG	-0.14	5.05	-1.38	0.17275
SELENOT	-0.12	8.72	-1.38	0.17275
ZEB1-AS1	-0.14	5.70	-1.38	0.17300
G6PC3	0.15	8.61	1.38	0.17303
AC245060.5	0.30	6.75	1.38	0.17307
LIME1	0.21	5.81	1.38	0.17308
FCSK	0.10	8.39	1.38	0.17308
EEF1AKMT4	0.16	5.71	1.38	0.17318
DNAJC1	0.13	8.84	1.38	0.17323
UBE4B	-0.11	9.77	-1.38	0.17329
NIPA2	-0.08	9.07	-1.38	0.17330
GAL3ST4	0.22	7.10	1.38	0.17338
MPST	0.15	8.46	1.38	0.17340
H2BC9	-0.18	6.00	-1.38	0.17340
STK36	-0.16	7.01	-1.38	0.17342
RPL38P3	0.16	6.05	1.38	0.17346
LRRC45	0.12	7.90	1.38	0.17346
SLC14A1	0.28	7.98	1.38	0.17349
ZNF746	-0.16	10.09	-1.38	0.17349
RNGTT	-0.13	7.33	-1.38	0.17352
AF117829.1	-0.13	5.27	-1.38	0.17364
DPP8	-0.10	8.34	-1.38	0.17364
LZIC	0.11	8.44	1.38	0.17368
CDCA8	-0.11	6.00	-1.38	0.17368
DYRK1B	0.13	8.72	1.38	0.17371
MS4A4A	-0.21	6.28	-1.38	0.17374
GPR180	-0.14	5.80	-1.38	0.17375
CLP1	-0.09	8.26	-1.38	0.17376
TMEM205	0.15	8.69	1.38	0.17377
TTC32	-0.13	6.22	-1.38	0.17393
SLC41A3	0.10	8.76	1.38	0.17396

TRAV13-1	0.16	7.22	1.38	0.17396
HSPBAP1	-0.14	8.21	-1.38	0.17399
LINC02285	-0.16	4.86	-1.38	0.17400
LYZ	-0.23	14.34	-1.38	0.17411
CNN2	0.14	13.63	1.38	0.17415
UIMC1	-0.12	9.18	-1.38	0.17417
AC092045.1	-0.18	6.07	-1.38	0.17418
CSAD	-0.25	9.21	-1.38	0.17425
FAM207A	0.14	6.85	1.38	0.17427
GUSB	0.10	10.49	1.38	0.17444
NELFA	0.10	8.64	1.38	0.17445
TRIM33	0.15	9.23	1.38	0.17448
GMPPA	0.13	9.33	1.38	0.17460
TMED5	-0.13	8.40	-1.38	0.17460
TST	0.17	8.92	1.38	0.17461
FO393411.1	0.21	9.17	1.38	0.17462
CEP57	-0.10	7.90	-1.38	0.17462
MIS12	-0.12	7.29	-1.38	0.17466
LINC00920	0.14	5.49	1.38	0.17471
RINL	0.08	9.20	1.38	0.17471
TGIF1	0.11	8.07	1.38	0.17473
ACTG1P20	0.18	6.58	1.38	0.17473
NOG	0.26	6.35	1.38	0.17474
REM2	-0.20	7.61	-1.38	0.17483
GATD1	0.11	9.03	1.38	0.17485
HLA-W	-0.48	7.42	-1.38	0.17485
MT-TQ	-0.12	4.98	-1.38	0.17486
ULK2	-0.12	7.87	-1.38	0.17504
TXLNG	-0.13	7.09	-1.37	0.17509
RGCC	0.14	8.71	1.37	0.17510
AC090198.1	-0.16	5.06	-1.37	0.17511
TRIP4	-0.08	8.21	-1.37	0.17515
ARPC5L	0.11	8.97	1.37	0.17523
NCLP1	-0.14	5.14	-1.37	0.17524
IER3	0.18	8.35	1.37	0.17525
SNX5	-0.09	9.06	-1.37	0.17531
SDF2L1	0.18	8.22	1.37	0.17537
MAP2K6	-0.16	7.57	-1.37	0.17556
TBCC	-0.07	9.55	-1.37	0.17557
PSMC6	-0.07	8.90	-1.37	0.17562
MCM8	0.14	5.99	1.37	0.17563
NOL10	0.11	7.76	1.37	0.17563
EFNA5	-0.20	5.00	-1.37	0.17565
H2BC4	-0.25	7.63	-1.37	0.17570
SLC35E4	0.14	5.63	1.37	0.17572
BRCA1	-0.14	6.75	-1.37	0.17574
C4orf33	-0.15	6.33	-1.37	0.17576
AC008124.1	-0.10	7.14	-1.37	0.17579
SMIM11B	-0.12	4.98	-1.37	0.17580
NDUFA6-DT	-0.11	5.66	-1.37	0.17581

AL645949.2	0.13	5.31	1.37	0.17597
P2RX7	-0.15	8.22	-1.37	0.17604
FCER2	0.30	9.27	1.37	0.17606
PLEKHA8P1	0.11	6.61	1.37	0.17608
AC005224.3	0.15	5.47	1.37	0.17609
ORMDL1	-0.11	8.33	-1.37	0.17609
ICAM1	-0.15	9.62	-1.37	0.17614
SLC16A7	-0.14	6.48	-1.37	0.17615
SCN1B	0.25	7.08	1.37	0.17616
HSPA8P7	-0.14	5.20	-1.37	0.17617
KTN1	0.14	9.65	1.37	0.17624
LIMK1	0.10	9.95	1.37	0.17624
SERP1	0.12	10.61	1.37	0.17625
TBCD	0.10	10.37	1.37	0.17627
PIGX	-0.14	8.61	-1.37	0.17641
ASTN2	0.14	5.72	1.37	0.17643
SPCS2P4	-0.15	6.05	-1.37	0.17651
SLC37A4	0.09	7.70	1.37	0.17656
TAF6L	0.13	7.25	1.37	0.17656
PIK3C2A	-0.15	6.44	-1.37	0.17657
CLSTN1	0.09	11.29	1.37	0.17660
ARMCX5	-0.12	6.09	-1.37	0.17661
WBP4	0.16	8.02	1.37	0.17674
RBM18	-0.09	7.85	-1.37	0.17674
SIMC1	-0.12	7.05	-1.37	0.17678
KIR2DL4	-0.19	5.50	-1.37	0.17688
RERE	-0.13	11.34	-1.37	0.17689
AL645933.3	-0.34	6.40	-1.37	0.17692
OSTF1	-0.15	10.34	-1.37	0.17695
RPL3P2	0.18	8.60	1.37	0.17696
MT-TL1	0.20	7.30	1.37	0.17707
PARP15	-0.20	7.65	-1.37	0.17709
POLD2P1	0.13	4.98	1.37	0.17710
DNAJB11	-0.11	7.73	-1.37	0.17722
HPS1	0.16	11.20	1.37	0.17723
COPS9	0.17	8.37	1.37	0.17724
AC097376.2	0.21	5.04	1.37	0.17750
RNF215	0.11	6.53	1.37	0.17751
PASK	0.18	9.13	1.37	0.17755
KIAA0355	-0.13	8.32	-1.37	0.17771
KLHL5	-0.12	7.13	-1.37	0.17776
COX17	0.14	8.05	1.37	0.17778
RPL35AP21	0.21	6.53	1.37	0.17779
ZNF207	-0.07	10.12	-1.37	0.17779
RAD54L2	0.08	8.75	1.37	0.17784
PLCH2	-0.21	7.58	-1.37	0.17794
SMG6	0.08	9.43	1.37	0.17803
API5	-0.11	9.02	-1.37	0.17807
SPINT1	0.15	9.18	1.37	0.17807
ENKUR	-0.17	6.20	-1.36	0.17817

POU5F1B	0.19	5.00	1.36	0.17819
LRRC37BP1	0.10	6.23	1.36	0.17821
CASC3	-0.14	11.19	-1.36	0.17823
SPATA6	-0.16	5.89	-1.36	0.17826
KLF13	-0.09	12.28	-1.36	0.17827
ERF	0.12	9.10	1.36	0.17832
GPR107	-0.09	9.69	-1.36	0.17833
TLK2P1	-0.14	5.43	-1.36	0.17840
ACOT9	-0.12	8.88	-1.36	0.17841
DOCK7	-0.13	5.94	-1.36	0.17855
AC011484.1	0.26	5.09	1.36	0.17865
ABHD17AP1	0.14	5.80	1.36	0.17874
SH3BP5-AS1	-0.22	7.50	-1.36	0.17881
TBC1D10A	0.08	8.50	1.36	0.17887
WDR91	-0.11	8.62	-1.36	0.17890
BNIP1	0.13	6.87	1.36	0.17908
ZNF92	-0.15	6.52	-1.36	0.17913
EFHC2	-0.18	5.38	-1.36	0.17921
WARS1	-0.22	11.73	-1.36	0.17936
HARS1	0.08	9.63	1.36	0.17940
TMEM109	0.11	10.38	1.36	0.17943
TMEM120B	0.14	6.64	1.36	0.17945
HMBOX1	0.11	7.99	1.36	0.17946
ZBTB39	-0.12	6.83	-1.36	0.17950
APOL2	-0.16	10.87	-1.36	0.17960
BBS4	-0.11	6.79	-1.36	0.17969
ARFIP1	-0.15	7.77	-1.36	0.17985
TUBD1	0.11	6.90	1.36	0.17987
INAFM1	0.16	7.53	1.36	0.17987
HINT3	-0.10	7.56	-1.36	0.17998
NPL	-0.18	9.94	-1.36	0.18009
BNC2	-0.19	5.03	-1.36	0.18010
CAST	-0.11	10.85	-1.36	0.18011
SUZ12	-0.12	7.71	-1.36	0.18012
INIP	-0.08	8.11	-1.36	0.18017
SLC4A7	-0.15	6.46	-1.36	0.18022
KIN	-0.11	7.14	-1.36	0.18024
AC093249.6	0.19	5.05	1.36	0.18034
EIF4HP1	-0.18	5.99	-1.36	0.18042
LSM3	0.11	8.43	1.36	0.18063
IGHA1	0.46	12.84	1.36	0.18064
AC215522.2	-0.24	5.80	-1.36	0.18069
LINC00641	-0.19	5.58	-1.36	0.18071
AC020899.1	0.10	5.30	1.36	0.18072
TRBV11-2	0.20	6.58	1.36	0.18074
DLGAP1-AS1	0.15	7.06	1.36	0.18079
GABPB2	0.12	8.43	1.36	0.18080
WDR59	0.09	9.21	1.36	0.18083
PFDN2	0.11	8.25	1.36	0.18084
UBE2J2	0.08	9.24	1.36	0.18097

TPT1P6	0.14	6.28	1.36	0.18104
SUN1	-0.18	8.62	-1.36	0.18107
LIAS	0.09	7.21	1.36	0.18108
KCNJ2-AS1	-0.18	5.09	-1.36	0.18110
UBXN6	0.27	12.45	1.36	0.18113
JOSD2	0.18	7.21	1.36	0.18121
AC079922.1	0.15	6.72	1.36	0.18126
NCBP2AS2	0.11	7.92	1.35	0.18137
PRCP	-0.09	10.08	-1.35	0.18137
HCG18	0.12	8.50	1.35	0.18139
SLC8B1	0.10	10.20	1.35	0.18150
AC016747.1	0.16	6.31	1.35	0.18165
DTX3	0.15	8.17	1.35	0.18167
SBF1	0.09	11.41	1.35	0.18181
ABHD8	0.16	7.93	1.35	0.18191
AOC2	-0.23	7.29	-1.35	0.18196
PRPF40A	-0.10	9.15	-1.35	0.18203
WASHC5	-0.10	8.41	-1.35	0.18208
ANO10	-0.11	7.87	-1.35	0.18215
FCRL6	-0.21	8.89	-1.35	0.18223
MT-TI	0.20	6.72	1.35	0.18226
PAX5	0.29	9.27	1.35	0.18232
ZNF90	-0.13	5.06	-1.35	0.18236
CCL5	0.24	13.71	1.35	0.18241
METAP1	-0.11	8.06	-1.35	0.18243
ARMC8	-0.07	8.65	-1.35	0.18264
RNF212	0.20	5.17	1.35	0.18270
PRMT3	-0.12	6.06	-1.35	0.18280
GABPB1-AS1	0.30	8.25	1.35	0.18281
ZNF571	-0.12	5.55	-1.35	0.18282
AC099524.1	0.20	6.47	1.35	0.18283
DVL2	0.09	8.68	1.35	0.18297
IRF8	-0.13	9.97	-1.35	0.18306
FGR	-0.15	13.45	-1.35	0.18316
ZNF337-AS1	0.15	5.06	1.35	0.18322
AC005831.1	-0.16	5.55	-1.35	0.18331
AC114752.2	-0.18	4.97	-1.35	0.18336
COLGALT2	-0.22	6.10	-1.35	0.18350
CCDC92	0.10	9.92	1.35	0.18352
BTBD9	0.08	8.30	1.35	0.18354
WIPI1	-0.16	8.27	-1.35	0.18355
WDFY2	-0.09	9.36	-1.35	0.18359
TAF7	-0.08	10.38	-1.35	0.18371
CCDC47	-0.11	8.30	-1.35	0.18373
TRBV5-5	0.15	5.54	1.35	0.18382
TEKT4P2	0.30	7.79	1.35	0.18397
NEO1	0.14	7.10	1.35	0.18398
GEMIN8	0.12	7.29	1.35	0.18411
AC005005.1	0.12	5.02	1.35	0.18412
VAV3	-0.15	8.23	-1.35	0.18415

MTIF3	0.08	8.69	1.35	0.18417
AC116533.1	0.19	10.52	1.35	0.18420
AP1S2	-0.12	9.78	-1.35	0.18421
RRNAD1	0.10	8.17	1.35	0.18425
LILRA6	-0.24	10.77	-1.35	0.18426
EMID1	0.21	5.30	1.35	0.18428
HMGB1P6	-0.18	7.34	-1.35	0.18429
LINC01781	0.22	5.34	1.35	0.18431
AC104837.1	-0.17	5.48	-1.35	0.18433
AC138150.1	-0.13	5.02	-1.35	0.18435
TNNC2	0.20	5.71	1.35	0.18436
CD40	0.13	8.30	1.35	0.18445
BRI3BP	0.12	8.39	1.34	0.18450
LRRC4	-0.25	8.89	-1.34	0.18469
MRPS23	0.11	7.93	1.34	0.18471
NEDD4	-0.18	5.50	-1.34	0.18475
ACSL6	0.13	6.07	1.34	0.18486
BCL2L2	-0.11	6.83	-1.34	0.18488
SMG8	-0.11	7.37	-1.34	0.18491
RNF220	0.07	10.62	1.34	0.18493
ENO2	0.13	9.07	1.34	0.18495
AL078621.3	0.14	5.21	1.34	0.18496
CLPX	-0.11	8.12	-1.34	0.18509
EMG1	0.11	9.03	1.34	0.18521
CLN5	0.09	7.24	1.34	0.18525
RPS7P11	0.15	6.56	1.34	0.18528
TNS3	-0.16	8.32	-1.34	0.18532
SNN	-0.16	11.37	-1.34	0.18536
NSRP1	0.15	8.68	1.34	0.18538
PTPN13	-0.18	5.42	-1.34	0.18542
STX10	0.13	11.16	1.34	0.18544
GTF2IP1	-0.19	7.60	-1.34	0.18546
TKFC	0.10	8.57	1.34	0.18546
DPH1	0.12	7.46	1.34	0.18547
ZNF736	-0.16	6.77	-1.34	0.18551
HMCES	0.09	9.60	1.34	0.18567
ZNF211	-0.10	7.96	-1.34	0.18579
AC015911.3	-0.16	5.74	-1.34	0.18582
TGM1	-0.15	5.69	-1.34	0.18589
RNF113A	0.11	9.40	1.34	0.18593
CORO2A	0.09	8.07	1.34	0.18594
CAPRIN2	-0.13	7.38	-1.34	0.18596
HSPA14	-0.07	7.82	-1.34	0.18602
TMSB10P1	0.20	7.55	1.34	0.18618
LINC00963	0.17	9.94	1.34	0.18623
COPDA1	0.22	5.07	1.34	0.18634
ATP5MGP2	0.17	6.13	1.34	0.18636
C15orf41	-0.15	5.28	-1.34	0.18645
WASHC2A	-0.09	9.00	-1.34	0.18655
RORA	-0.13	8.02	-1.34	0.18655

RPSAP12	0.19	7.62	1.34	0.18658
FAAP24	0.13	6.67	1.34	0.18672
SNIP1	-0.09	7.62	-1.34	0.18675
LINC00265	-0.14	6.78	-1.34	0.18678
TSPAN17	0.13	9.27	1.34	0.18680
SAMD10	0.18	7.45	1.34	0.18681
FTSJ3	0.10	9.62	1.34	0.18682
KSR1	-0.14	9.39	-1.34	0.18713
HADHA	0.09	11.92	1.34	0.18717
HEATR6	-0.10	7.65	-1.34	0.18719
CTR9	-0.09	9.49	-1.34	0.18722
FOXP4	0.15	8.25	1.34	0.18727
PHF21A	-0.17	10.84	-1.34	0.18730
RWDD2B	0.12	6.68	1.34	0.18731
ICA1L	0.16	5.62	1.34	0.18737
SNRNP48	-0.10	6.98	-1.34	0.18738
MBOAT2	-0.22	7.87	-1.34	0.18750
ZNF274	-0.10	8.82	-1.34	0.18753
LINC00402	0.21	6.45	1.34	0.18764
ZFAND2A	0.16	7.92	1.34	0.18766
NAT9	0.11	8.64	1.33	0.18774
AL136968.2	0.13	5.40	1.33	0.18795
HSPA13	-0.14	6.39	-1.33	0.18806
PARK7	0.12	10.85	1.33	0.18807
SFI1	0.13	10.05	1.33	0.18822
IGSF22	0.14	5.22	1.33	0.18825
RPL10P19	0.15	5.83	1.33	0.18840
SLC12A2	-0.14	5.71	-1.33	0.18844
AL132709.8	0.17	5.80	1.33	0.18848
MGAT1	0.10	12.27	1.33	0.18850
CA13	-0.15	5.23	-1.33	0.18853
ACO1	-0.12	7.33	-1.33	0.18854
ADH5	0.09	9.14	1.33	0.18861
TMEM9	0.14	8.75	1.33	0.18877
SAP18	0.08	10.48	1.33	0.18880
EXOC3-AS1	0.16	5.39	1.33	0.18888
P2RY8	0.09	11.44	1.33	0.18888
RTN4	-0.11	9.90	-1.33	0.18889
PNPLA4	0.12	6.32	1.33	0.18904
KIAA1671	-0.22	6.82	-1.33	0.18916
MLF2	0.12	11.34	1.33	0.18918
EAPP	-0.08	9.49	-1.33	0.18924
MRPL1	0.13	6.86	1.33	0.18927
RUFY3	-0.10	7.42	-1.33	0.18932
HSD17B11	-0.15	10.15	-1.33	0.18935
ANKLE1	-0.14	6.16	-1.33	0.18937
ALOX15B	-0.22	5.54	-1.33	0.18944
ABCC5	-0.17	8.90	-1.33	0.18944
MMS22L	-0.11	5.83	-1.33	0.18950
MFN1	-0.12	7.73	-1.33	0.18950

EFTUD2	0.10	10.76	1.33	0.18951
TP53RK	0.10	7.97	1.33	0.18957
CRCP	0.08	8.10	1.33	0.18971
AC141586.1	0.12	4.99	1.33	0.18973
NANS	0.14	8.98	1.33	0.18978
THUMPD1	-0.10	8.20	-1.33	0.18980
IKBKG	0.11	7.94	1.33	0.18981
TRNAU1AP	0.09	8.54	1.33	0.18985
LPAR6	-0.14	8.56	-1.33	0.18993
ANKRD36	0.17	5.89	1.33	0.19004
ABCA3	0.11	7.54	1.33	0.19020
ZNF70	-0.16	5.93	-1.33	0.19040
GBGT1	0.15	8.21	1.33	0.19057
RPL7P18	0.19	5.56	1.33	0.19070
AC004466.1	0.14	5.12	1.33	0.19083
SUN2	0.07	13.59	1.33	0.19085
ZNF879	0.14	5.90	1.33	0.19090
RBM15B	0.08	10.15	1.33	0.19094
HAAO	0.15	6.98	1.33	0.19096
SLC35A1	-0.11	7.45	-1.32	0.19100
SDHD	-0.12	7.81	-1.32	0.19106
PITPNB	-0.07	8.48	-1.32	0.19107
ZFY	-0.32	5.41	-1.32	0.19115
CLCC1	-0.10	7.61	-1.32	0.19115
CSGALNACT1	-0.25	8.18	-1.32	0.19123
POP4	0.09	8.84	1.32	0.19126
C17orf113	0.12	5.09	1.32	0.19134
DDX24	0.09	11.23	1.32	0.19158
AC009041.1	0.19	5.97	1.32	0.19165
ZNF853	0.13	6.84	1.32	0.19165
FBH1	0.06	10.11	1.32	0.19170
SCAMP5	0.19	6.60	1.32	0.19172
RNU7-45P	0.21	5.00	1.32	0.19177
AC040162.1	-0.19	7.02	-1.32	0.19189
ANKRD13C	-0.11	6.61	-1.32	0.19191
N4BP2L2	-0.13	9.83	-1.32	0.19193
GNG8	0.14	5.09	1.32	0.19197
NAA40	-0.11	8.38	-1.32	0.19197
P4HTM	0.12	8.35	1.32	0.19205
ADAP1	0.18	8.09	1.32	0.19218
GMPSP1	0.16	5.09	1.32	0.19220
KCTD20	-0.10	9.78	-1.32	0.19222
PITPNM1	0.09	10.81	1.32	0.19225
TESPA1	0.16	10.05	1.32	0.19236
GARS1	0.08	9.47	1.32	0.19238
CLCN4	-0.21	6.78	-1.32	0.19257
SPIDR	-0.10	8.85	-1.32	0.19271
RECQL5	0.09	8.74	1.32	0.19276
AC074117.1	-0.13	5.74	-1.32	0.19276
SQSTM1	0.08	12.24	1.32	0.19279

WASHC1	0.22	8.38	1.32	0.19283
AC118549.1	-0.13	7.24	-1.32	0.19295
PAGR1	0.11	7.26	1.32	0.19295
ZFPL1	0.09	8.42	1.32	0.19295
AC113367.1	0.23	8.18	1.32	0.19296
TRGV9	-0.19	6.16	-1.32	0.19297
SASH1	-0.22	5.58	-1.32	0.19302
HAGH	0.23	10.98	1.32	0.19303
ESAM	0.19	8.09	1.32	0.19304
AL162424.1	0.17	6.13	1.32	0.19305
CHAF1B	0.14	5.97	1.32	0.19306
CSTF2T	-0.08	8.91	-1.32	0.19306
CLASRP	0.12	10.31	1.32	0.19307
PDE7A	-0.12	9.24	-1.32	0.19314
ZNF622	0.11	9.37	1.32	0.19320
DDX46	0.13	9.52	1.32	0.19334
AL133367.1	-0.18	5.17	-1.32	0.19336
NSUN4	0.10	7.92	1.32	0.19337
CMTM3	0.13	11.01	1.32	0.19343
POLL	0.20	9.75	1.32	0.19349
RBBP5	-0.09	8.30	-1.32	0.19360
PORCN	0.11	7.52	1.32	0.19360
NLRP1	-0.16	12.08	-1.32	0.19368
AC016727.1	-0.12	5.59	-1.32	0.19374
CD86	-0.14	9.30	-1.32	0.19384
SPTSSA	-0.12	7.24	-1.32	0.19388
ZNF644	-0.13	7.30	-1.32	0.19391
EMC3	0.17	10.72	1.32	0.19392
FCHO1	0.09	10.81	1.32	0.19396
PDE6G	0.13	6.30	1.32	0.19400
GMPR2	-0.11	10.09	-1.32	0.19402
FAM171A1	0.19	6.73	1.32	0.19404
SNRPD1	0.13	8.19	1.32	0.19407
NAIPP1	-0.16	5.72	-1.32	0.19427
PTBP3	-0.12	9.85	-1.32	0.19427
CWC22	-0.09	7.97	-1.31	0.19434
VIM-AS1	-0.13	8.03	-1.31	0.19458
LPAR5	0.12	8.52	1.31	0.19465
BTBD6	0.10	9.18	1.31	0.19467
MIRLET7BHG	0.19	6.84	1.31	0.19468
MTPAP	-0.12	7.06	-1.31	0.19470
RPL10P15	0.22	7.54	1.31	0.19475
MBD4	-0.08	9.13	-1.31	0.19479
PTMS	0.21	8.63	1.31	0.19480
STMN1	0.14	8.29	1.31	0.19483
AC103706.1	0.14	5.49	1.31	0.19488
USP45	-0.14	5.56	-1.31	0.19489
BARD1	-0.13	5.32	-1.31	0.19489
PYGB	0.08	10.52	1.31	0.19491
ZNF689	0.10	7.87	1.31	0.19502

MANBA	-0.09	9.89	-1.31	0.19516
RIPK1	-0.08	9.98	-1.31	0.19519
SAMD4A	-0.20	5.99	-1.31	0.19521
EBLN2	0.18	5.37	1.31	0.19522
PRX	0.15	6.65	1.31	0.19524
GKAP1	0.12	5.86	1.31	0.19530
SH3PXD2B	-0.17	5.32	-1.31	0.19534
ZNF827	0.16	6.90	1.31	0.19538
MPL	0.19	5.41	1.31	0.19540
CFLAR	-0.18	11.96	-1.31	0.19547
SNX6P1	-0.18	5.25	-1.31	0.19548
TTC31	-0.10	8.60	-1.31	0.19554
MX1	-0.37	10.80	-1.31	0.19555
LINC01278	-0.10	7.83	-1.31	0.19559
AC099778.1	0.16	5.52	1.31	0.19566
FANCA	-0.16	7.99	-1.31	0.19580
BMP2K	-0.16	8.19	-1.31	0.19582
AC019205.1	0.14	5.31	1.31	0.19583
TRMU	0.15	8.59	1.31	0.19588
RGPD8	-0.13	5.26	-1.31	0.19593
NCK1	-0.12	7.30	-1.31	0.19601
ZNF583	-0.15	5.60	-1.31	0.19617
AL356653.1	0.14	5.13	1.31	0.19623
GFM1	-0.13	7.36	-1.31	0.19633
ELOVL7	-0.19	5.83	-1.31	0.19638
PIGW	-0.15	5.30	-1.31	0.19639
AL023653.1	-0.16	5.19	-1.31	0.19663
GLS2	0.16	5.63	1.31	0.19663
HBB	0.39	13.11	1.31	0.19668
AC015883.1	0.21	5.17	1.31	0.19671
AC016712.1	0.14	5.18	1.31	0.19673
TRMT13	-0.14	6.27	-1.31	0.19675
THAP1	-0.12	7.00	-1.31	0.19675
SEMA4F	0.12	6.85	1.31	0.19678
AC138393.1	-0.14	5.52	-1.31	0.19693
RNF181	0.13	9.95	1.31	0.19694
AP5B1	-0.19	11.01	-1.31	0.19700
CHCHD5	0.14	7.93	1.31	0.19704
PAIP1	-0.10	7.24	-1.31	0.19718
LINC01134	-0.12	5.09	-1.31	0.19719
CCDC127	0.12	8.17	1.31	0.19725
TMCC3	-0.20	9.25	-1.31	0.19727
PF4V1	0.25	6.73	1.31	0.19728
PLA2G4C	0.27	5.93	1.31	0.19732
URB1	0.11	8.25	1.31	0.19740
RICTOR	-0.17	8.53	-1.31	0.19745
OPTN	0.18	11.59	1.31	0.19752
ATG9A	-0.15	8.62	-1.31	0.19754
BNIP2	-0.11	9.47	-1.31	0.19754
COL6A1	0.20	6.14	1.31	0.19758

PIGN	-0.11	6.89	-1.31	0.19763
SNAPC1	0.13	5.67	1.31	0.19764
PAN3-AS1	-0.12	5.45	-1.30	0.19775
INKA2	-0.21	10.64	-1.30	0.19775
ATP5F1E	0.14	11.95	1.30	0.19787
LSP1	0.15	14.36	1.30	0.19790
UTP15	-0.12	6.35	-1.30	0.19794
ERFL	0.16	5.66	1.30	0.19821
ANKHD1-EIF4EBP3	0.16	5.66	1.30	0.19827
RPL38P4	0.17	6.82	1.30	0.19827
GRK5	0.08	9.19	1.30	0.19831
NFKB2	0.09	11.24	1.30	0.19833
CCT8	-0.09	9.94	-1.30	0.19836
ABHD17B	-0.12	6.62	-1.30	0.19846
MINPP1	-0.10	6.41	-1.30	0.19850
SRP68	0.09	10.12	1.30	0.19855
RABL2A	0.13	6.95	1.30	0.19874
CARM1	0.19	10.21	1.30	0.19877
DDIAS	-0.12	5.72	-1.30	0.19878
BYSL	0.17	7.14	1.30	0.19902
GAPT	-0.12	9.26	-1.30	0.19911
ZC3H3	0.12	9.36	1.30	0.19912
TSTD2	-0.12	7.06	-1.30	0.19913
HTR6	0.27	5.49	1.30	0.19915
AC023157.1	-0.28	8.41	-1.30	0.19926
P2RX4	0.13	9.07	1.30	0.19926
DEGS1	-0.12	9.19	-1.30	0.19930
KCTD2	0.08	9.98	1.30	0.19941
ZKSCAN7	-0.16	5.44	-1.30	0.19946
GOT2	0.09	9.34	1.30	0.19975
CTSA	0.12	12.38	1.30	0.19979
PFKFB4	-0.19	10.04	-1.30	0.19990
BX679664.3	0.21	8.56	1.30	0.19991
CDH1	0.19	5.87	1.30	0.19995
HOOK3	0.14	8.65	1.30	0.19997
AGAP4	-0.21	6.60	-1.30	0.20000
TMEM126B	-0.10	7.32	-1.30	0.20004
RETREG3	0.07	10.09	1.30	0.20009
DLD	-0.09	8.52	-1.30	0.20019
MRPL38	0.18	5.83	1.30	0.20021
PHKG1	0.12	5.29	1.30	0.20022
GTF2H1	-0.09	8.21	-1.30	0.20036
SNX4	-0.09	7.15	-1.30	0.20036
CHST11	-0.12	10.72	-1.30	0.20038
ATN1	0.12	9.12	1.30	0.20041
MROH8	0.17	5.07	1.30	0.20046
PER1	0.15	9.18	1.30	0.20056
PRIM1	-0.13	5.75	-1.30	0.20062
C12orf65	0.11	8.43	1.30	0.20073
CMTR1	-0.15	10.13	-1.30	0.20078

SMG1P5	-0.12	5.05	-1.30	0.20101
SLAIN1	-0.13	6.67	-1.30	0.20105
AL139246.3	0.17	5.47	1.30	0.20106
PIK3CD	-0.11	13.07	-1.29	0.20115
INTS2	-0.15	6.22	-1.29	0.20118
AC087501.4	-0.13	5.04	-1.29	0.20119
BIN2	-0.11	12.32	-1.29	0.20129
MRPL14	0.15	8.68	1.29	0.20134
ATP5F1A	0.09	12.08	1.29	0.20135
ZFHX3	0.17	7.58	1.29	0.20160
MCF2L	0.15	6.67	1.29	0.20169
LINC01857	0.19	5.38	1.29	0.20172
DRAP1	0.16	10.91	1.29	0.20180
DAPP1	-0.13	10.07	-1.29	0.20188
HPRT1	-0.10	7.10	-1.29	0.20192
COG5	-0.12	7.56	-1.29	0.20194
CXCL16	-0.18	9.87	-1.29	0.20207
GATD3A	0.16	6.14	1.29	0.20221
OSBPL7	0.12	8.98	1.29	0.20228
DSP	-0.36	5.63	-1.29	0.20232
AC108673.3	-0.15	5.04	-1.29	0.20238
ZNF382	-0.14	5.37	-1.29	0.20239
COMMD6	0.15	9.38	1.29	0.20241
CHCHD7	0.08	7.84	1.29	0.20244
PCBP2	0.08	11.93	1.29	0.20255
IFRD1	-0.18	8.98	-1.29	0.20259
EPHB1	-0.17	7.42	-1.29	0.20263
SLC35D2	0.12	6.48	1.29	0.20271
FYTTD1	-0.10	8.33	-1.29	0.20276
MFSD4B	0.16	7.09	1.29	0.20291
ADAM19	-0.17	10.41	-1.29	0.20292
LINC01506	-0.24	7.23	-1.29	0.20301
DPY19L3	-0.17	7.26	-1.29	0.20301
GZMK	0.20	9.45	1.29	0.20325
AC046134.2	0.15	5.15	1.29	0.20328
STT3B	-0.10	9.33	-1.29	0.20365
AC020904.2	-0.16	5.34	-1.29	0.20365
BIRC3	0.14	9.54	1.29	0.20372
IL15	-0.15	6.54	-1.29	0.20379
BICD1	-0.14	5.96	-1.29	0.20382
CCT2	0.11	9.01	1.29	0.20395
SCML1	-0.18	5.35	-1.29	0.20404
TMEM120A	0.16	10.08	1.29	0.20407
U91328.1	-0.13	5.77	-1.29	0.20408
NFE2L3	-0.18	6.18	-1.29	0.20413
LINC01128	0.12	7.37	1.29	0.20419
COPG2	-0.12	7.39	-1.29	0.20426
GTPBP4	0.07	8.62	1.29	0.20432
NF1	-0.11	7.96	-1.29	0.20433
AC073072.1	0.22	5.20	1.29	0.20440

ARHGEF17	0.22	5.23	1.29	0.20444
C1D	-0.12	7.11	-1.29	0.20444
TRPV2	0.09	10.73	1.29	0.20448
ZNF573	0.15	5.23	1.29	0.20450
AC127496.7	0.14	5.27	1.29	0.20451
ENTPD1-AS1	0.09	6.39	1.28	0.20452
OAS1	-0.30	10.54	-1.28	0.20461
PFKP	0.10	9.43	1.28	0.20461
UBN1	-0.14	11.28	-1.28	0.20463
RPL5P1	0.20	6.91	1.28	0.20467
CCL28	-0.13	6.30	-1.28	0.20470
AL136295.2	-0.19	6.25	-1.28	0.20480
BAP1	0.06	10.54	1.28	0.20486
ZXDA	-0.13	5.32	-1.28	0.20487
MAPKAP1	0.07	9.72	1.28	0.20488
AUP1	0.08	11.15	1.28	0.20494
PLCG2	-0.12	11.23	-1.28	0.20495
CENPBD1	0.12	7.63	1.28	0.20496
ZC3H11B	-0.17	5.67	-1.28	0.20507
ZNF655	-0.08	9.29	-1.28	0.20511
C1orf61	0.16	5.16	1.28	0.20518
NSDHL	0.12	7.93	1.28	0.20535
TRIB3	0.13	7.63	1.28	0.20536
ASAP2	-0.16	5.59	-1.28	0.20549
GLMN	-0.12	5.69	-1.28	0.20550
KYNU	-0.17	7.93	-1.28	0.20571
SC5D	-0.12	6.62	-1.28	0.20575
SFT2D1	-0.09	8.36	-1.28	0.20585
GSTM3	0.30	6.31	1.28	0.20591
XRCC3	0.12	6.89	1.28	0.20591
IFNGR2	-0.13	11.54	-1.28	0.20592
IGLV3-9	0.28	5.86	1.28	0.20594
CASTOR3	-0.13	7.35	-1.28	0.20597
ACAP3	0.12	9.66	1.28	0.20601
PTPN1	-0.08	10.29	-1.28	0.20606
AL591846.2	0.29	6.96	1.28	0.20607
SERINC2	0.28	6.85	1.28	0.20611
TGFB1	0.11	13.17	1.28	0.20646
MON1B	0.10	10.24	1.28	0.20656
SHKBP1	0.16	12.70	1.28	0.20661
WDR45BP1	-0.15	5.07	-1.28	0.20664
HMGA1P4	0.17	5.49	1.28	0.20675
KIAA2013	0.11	10.76	1.28	0.20677
TREML1	0.28	9.47	1.28	0.20680
TOX4	-0.08	9.98	-1.28	0.20688
KIAA0895L	-0.20	6.53	-1.28	0.20698
C8orf33	0.09	8.59	1.28	0.20701
GAS2L1	0.20	8.29	1.28	0.20708
DPP4	0.17	7.92	1.28	0.20715
TBC1D14	-0.15	10.97	-1.28	0.20721

LRATD2	-0.13	6.83	-1.28	0.20724
RWDD4	-0.10	6.09	-1.28	0.20733
HNRNPM	0.08	11.03	1.28	0.20734
PRR11	0.14	6.72	1.28	0.20735
KLHL6	-0.10	8.86	-1.28	0.20743
LARGE1	-0.14	6.30	-1.28	0.20752
SERINC5	-0.13	8.57	-1.28	0.20755
SRP14P1	0.14	5.42	1.28	0.20764
SLC25A6P2	0.14	5.40	1.28	0.20777
SDCBPP2	-0.18	5.50	-1.28	0.20779
ANXA3	-0.34	8.08	-1.28	0.20779
G6PD	0.14	12.08	1.28	0.20783
KLLN	-0.14	4.95	-1.28	0.20783
AC073508.3	0.12	6.02	1.28	0.20788
SH3RF3	0.19	6.72	1.28	0.20795
KCNAB2	0.08	11.92	1.28	0.20798
HLF	0.14	5.02	1.28	0.20799
TRMT12	0.09	7.08	1.27	0.20802
SGSH	0.10	9.65	1.27	0.20802
MAPK3	0.12	10.59	1.27	0.20808
ING5	0.09	9.05	1.27	0.20822
AL109627.1	0.11	6.67	1.27	0.20831
SRA1	0.11	9.48	1.27	0.20841
ITK	-0.17	9.75	-1.27	0.20843
DNAL4	0.10	7.88	1.27	0.20848
CLDN5	0.22	7.02	1.27	0.20859
HCN3	-0.14	5.41	-1.27	0.20859
DDX55	0.13	7.81	1.27	0.20865
CYP4F3	-0.22	10.20	-1.27	0.20865
PINX1	0.11	7.58	1.27	0.20867
Z74021.1	0.21	8.68	1.27	0.20880
SATB1-AS1	0.14	6.74	1.27	0.20884
DENND11	-0.14	8.09	-1.27	0.20888
PCID2	0.08	8.60	1.27	0.20907
SLC2A14	-0.29	5.91	-1.27	0.20912
MSANTD3	0.14	6.67	1.27	0.20912
C1orf54	0.14	5.69	1.27	0.20918
PGAP2	0.08	7.83	1.27	0.20919
ZFP82	0.11	6.44	1.27	0.20928
METTL25	-0.11	5.78	-1.27	0.20929
BCL10	-0.12	7.57	-1.27	0.20934
SMARCC2	-0.07	10.68	-1.27	0.20935
CSNK2A2	0.08	9.26	1.27	0.20942
NOP16	0.12	7.73	1.27	0.20950
HIC2	0.10	7.22	1.27	0.20951
ERMN	-0.15	5.94	-1.27	0.20956
MMP28	0.17	5.38	1.27	0.20977
MAK	-0.23	6.76	-1.27	0.20980
AC099560.2	0.22	9.22	1.27	0.20981
BLOC1S3	0.08	9.09	1.27	0.20986

CCDC112	0.13	6.61	1.27	0.20987
ZNF674	-0.12	5.59	-1.27	0.21008
ECT2	-0.15	5.11	-1.27	0.21012
MOCS2	-0.11	7.07	-1.27	0.21017
SEPTIN2	-0.08	10.63	-1.27	0.21030
CD99P1	-0.12	6.15	-1.27	0.21040
HNRNPL	0.06	10.62	1.27	0.21042
SHLD1	0.12	7.24	1.27	0.21072
TUBA4A	0.13	11.76	1.27	0.21076
ZSCAN20	-0.12	5.21	-1.27	0.21085
WASF4P	-0.21	5.35	-1.27	0.21091
ITGB5	0.20	8.47	1.27	0.21094
TMEM68	-0.11	6.24	-1.27	0.21099
MEF2C	-0.13	8.52	-1.27	0.21103
PSMD13	0.08	10.93	1.27	0.21105
NSG1	0.27	7.99	1.27	0.21114
EMC9	0.13	6.90	1.27	0.21118
SRSF5	-0.11	11.42	-1.27	0.21119
DAZAP2	-0.10	13.06	-1.27	0.21131
CASC4	-0.11	8.21	-1.27	0.21133
CAD	0.09	9.62	1.26	0.21163
TRAF4	0.14	7.68	1.26	0.21165
FAM104A	0.15	9.45	1.26	0.21167
TIMM9	0.14	7.37	1.26	0.21169
DHRS4	0.13	7.87	1.26	0.21170
LUC7L3	0.18	10.42	1.26	0.21198
RRP15	-0.14	6.56	-1.26	0.21214
TTC24	-0.18	5.16	-1.26	0.21221
AL645568.1	0.16	6.55	1.26	0.21230
AL031777.1	-0.15	5.37	-1.26	0.21242
TTC19	0.10	7.73	1.26	0.21248
GNPTG	0.10	10.11	1.26	0.21256
PCIF1	0.07	10.76	1.26	0.21285
SNHG7	0.17	8.38	1.26	0.21296
NMNAT1	0.10	7.01	1.26	0.21302
TYW3	-0.11	7.08	-1.26	0.21302
ORC2	-0.11	7.47	-1.26	0.21307
UBE2D3	-0.11	11.55	-1.26	0.21310
TATDN3	-0.09	7.06	-1.26	0.21312
PLEKHF1	0.16	9.02	1.26	0.21313
NAT8B	0.17	5.94	1.26	0.21318
SLC24A4	-0.15	9.29	-1.26	0.21322
MAU2	-0.11	10.72	-1.26	0.21325
ENTR1	0.09	8.80	1.26	0.21325
PIGM	0.15	7.65	1.26	0.21326
MYCL	-0.16	8.77	-1.26	0.21327
ZNF12	-0.09	7.89	-1.26	0.21329
VNN3	-0.21	9.01	-1.26	0.21334
BLVRB	0.25	11.23	1.26	0.21345
CHST8	-0.17	4.96	-1.26	0.21349

RGPD5	-0.15	5.19	-1.26	0.21365
RAVER1	0.12	8.51	1.26	0.21366
BCL9L	0.11	11.11	1.26	0.21366
AGRN	-0.26	6.18	-1.26	0.21371
FAM120C	-0.11	6.09	-1.26	0.21375
RBM33	-0.12	10.17	-1.26	0.21390
MAN1A1	-0.18	8.08	-1.26	0.21399
CACNA1I	0.19	8.21	1.26	0.21407
AGPAT1	0.09	10.74	1.26	0.21428
AC004890.2	0.16	5.29	1.26	0.21428
ZNF782	-0.14	5.55	-1.26	0.21438
ATP13A1	0.10	10.53	1.26	0.21444
AC018868.1	0.14	6.07	1.26	0.21446
KHSRP	0.10	10.00	1.26	0.21453
HK1	0.08	11.74	1.26	0.21466
KLHL3	-0.17	6.18	-1.26	0.21470
AC004224.1	0.17	6.35	1.26	0.21484
RNF24	-0.20	11.21	-1.26	0.21498
ATF5	0.17	7.83	1.26	0.21503
NEDD4L	0.20	6.83	1.26	0.21511
TCAIM	-0.10	6.74	-1.25	0.21517
PYCARD	0.15	11.03	1.25	0.21528
PCED1A	0.14	9.23	1.25	0.21531
IGKV1-9	0.27	6.88	1.25	0.21544
FAM3C	-0.11	6.38	-1.25	0.21555
POM121B	-0.14	5.40	-1.25	0.21561
SGK1	-0.14	9.63	-1.25	0.21586
DENND2C	-0.13	5.14	-1.25	0.21587
TBC1D30	-0.17	6.10	-1.25	0.21598
SRPRA	-0.06	11.32	-1.25	0.21603
PRDM5	-0.17	5.29	-1.25	0.21612
RPL7P1	0.18	8.57	1.25	0.21625
TNXB	0.16	5.84	1.25	0.21633
MST1P2	0.22	5.61	1.25	0.21648
CYBA	0.14	13.26	1.25	0.21664
RPL7P32	0.09	5.56	1.25	0.21667
CUL3	-0.09	9.70	-1.25	0.21672
SNX16	-0.15	5.44	-1.25	0.21676
AC040160.3	0.22	5.35	1.25	0.21678
EPOP	-0.15	5.08	-1.25	0.21686
DDX50	0.08	8.88	1.25	0.21687
TCEANC2	0.07	7.92	1.25	0.21692
MAP3K4	-0.10	8.57	-1.25	0.21700
AIFM3	0.19	6.66	1.25	0.21703
RASGEF1B	-0.12	6.50	-1.25	0.21707
POMGNT1	0.09	8.85	1.25	0.21708
RBIS	0.12	7.80	1.25	0.21715
ZBED4	-0.11	8.23	-1.25	0.21727
RUFY1	0.10	10.50	1.25	0.21729
ORAI3	0.12	7.56	1.25	0.21733

SLC1A4	-0.16	7.72	-1.25	0.21736
RAP2B	-0.07	10.10	-1.25	0.21740
U2AF2	0.07	11.85	1.25	0.21742
IKBIP	-0.16	8.09	-1.25	0.21746
FAM110B	-0.16	5.70	-1.25	0.21750
RHPN1	0.21	6.99	1.25	0.21751
SLC22A4	-0.23	7.96	-1.25	0.21755
MAP3K11	0.12	11.62	1.25	0.21760
LY9	0.11	9.77	1.25	0.21764
FUT11	0.14	8.99	1.25	0.21774
HUS1	-0.08	7.19	-1.25	0.21782
RPS4XP11	0.19	6.78	1.25	0.21783
KLHL26	0.08	7.42	1.25	0.21785
AXIN1	0.07	10.20	1.25	0.21794
ZNF432	0.14	6.36	1.25	0.21801
NRADDP	-0.18	5.68	-1.25	0.21805
LINC02132	0.17	5.31	1.25	0.21813
ERP44	-0.09	9.46	-1.25	0.21815
MRPL27	0.14	8.31	1.25	0.21816
CLEC4E	-0.27	10.20	-1.25	0.21822
DYNC1H1	-0.07	11.90	-1.25	0.21825
DERL1	-0.06	9.59	-1.25	0.21825
EEF1A1P8	-0.10	5.36	-1.25	0.21830
BCL2L13	-0.10	9.12	-1.25	0.21831
ZNF365	-0.15	5.01	-1.25	0.21833
RNFT2	0.16	5.92	1.25	0.21835
DCPS	0.10	8.93	1.25	0.21840
RFWD3	-0.11	7.90	-1.25	0.21841
UBE2D2	0.09	9.91	1.25	0.21842
GVQW3	-0.15	5.52	-1.25	0.21848
AC012645.3	0.14	6.16	1.25	0.21850
CDC42EP3	-0.14	10.47	-1.25	0.21863
SDHA	0.10	7.63	1.25	0.21870
KIFBP	-0.10	6.58	-1.25	0.21874
CLN6	0.13	8.00	1.24	0.21882
NSL1	-0.07	8.24	-1.24	0.21889
AL021707.1	-0.20	5.40	-1.24	0.21905
Z69706.1	0.15	5.32	1.24	0.21914
DHODH	0.10	6.98	1.24	0.21915
COQ2	-0.12	6.78	-1.24	0.21918
USP21	0.06	9.10	1.24	0.21942
RHD	-0.22	5.05	-1.24	0.21943
RBM7	-0.11	7.24	-1.24	0.21943
DCTN5	0.09	9.61	1.24	0.21951
MRTFB	-0.12	5.91	-1.24	0.21952
MPIG6B	0.21	9.04	1.24	0.21958
SFXN1	0.11	8.15	1.24	0.21964
GALNT1	-0.11	8.35	-1.24	0.21968
PAK6	-0.16	5.60	-1.24	0.21973
ZNF675	-0.13	5.96	-1.24	0.21974

ICK	-0.13	6.52	-1.24	0.21976
AL512633.1	-0.20	5.40	-1.24	0.21978
SREK1	0.13	8.44	1.24	0.21980
SYNPO	0.13	6.83	1.24	0.21981
EBNA1BP2	0.10	8.69	1.24	0.21981
NAT1	-0.12	6.02	-1.24	0.21989
TIFA	-0.11	7.54	-1.24	0.21994
PRSS33	0.33	7.52	1.24	0.21997
SHISA7	0.23	5.25	1.24	0.22005
IGHV3-11	0.26	6.90	1.24	0.22007
CRB3	0.13	4.96	1.24	0.22011
POFUT2	-0.11	8.54	-1.24	0.22013
ATP1B3	-0.10	9.21	-1.24	0.22014
PTGIR	0.14	7.99	1.24	0.22015
AHCY	0.10	9.07	1.24	0.22032
MECR	0.11	7.20	1.24	0.22033
ST3GAL3	0.14	5.70	1.24	0.22062
PREX1	-0.17	13.36	-1.24	0.22073
TMEM168	-0.12	7.17	-1.24	0.22083
MFSD2B	0.26	7.02	1.24	0.22084
ARL2BP	-0.08	9.06	-1.24	0.22103
LACC1	-0.11	5.22	-1.24	0.22105
PSMB6	0.12	9.80	1.24	0.22108
HNRNPDL	-0.08	10.62	-1.24	0.22110
RIPOR2	-0.14	12.78	-1.24	0.22110
ATG16L2	-0.21	13.33	-1.24	0.22113
TNPO2	0.09	9.66	1.24	0.22118
ERP29P1	0.15	5.80	1.24	0.22127
RBM5	-0.10	11.61	-1.24	0.22131
NISCH	0.09	10.92	1.24	0.22143
TTC30B	-0.12	5.33	-1.24	0.22154
ZNF609	-0.10	9.22	-1.24	0.22167
HARS2	-0.07	8.87	-1.24	0.22169
RPS12	0.14	14.04	1.24	0.22175
LRRC28	0.13	7.57	1.24	0.22181
FBXO5	-0.12	5.91	-1.24	0.22190
AP4M1	0.11	7.45	1.24	0.22191
AC007688.2	0.18	7.14	1.24	0.22207
PIGK	-0.10	7.08	-1.24	0.22209
ATP6V0CP3	0.15	5.20	1.24	0.22211
ABHD4	-0.12	9.62	-1.24	0.22220
AL160272.1	-0.17	5.27	-1.24	0.22221
HCAR3	-0.22	9.94	-1.24	0.22226
PNPLA2	0.14	11.88	1.24	0.22232
RAE1	0.08	9.15	1.24	0.22234
DNAJB9	-0.10	6.90	-1.23	0.22245
COPS7A	0.08	9.54	1.23	0.22247
KIAA0513	-0.11	11.20	-1.23	0.22249
SMIM27	0.13	7.75	1.23	0.22251
IARS2	-0.10	8.46	-1.23	0.22268

LINC01127	-0.28	8.07	-1.23	0.22268
ICOS	0.14	6.98	1.23	0.22269
ANKRD49	-0.09	7.50	-1.23	0.22282
AL691432.4	0.13	5.56	1.23	0.22299
B4GALNT3	0.36	6.43	1.23	0.22301
RGL1	-0.19	5.80	-1.23	0.22301
AF241726.1	0.18	5.54	1.23	0.22318
NUDT19	-0.09	6.56	-1.23	0.22323
AC092683.1	0.15	5.38	1.23	0.22324
AC244157.2	-0.17	5.10	-1.23	0.22335
GALNT11	-0.08	8.36	-1.23	0.22337
ADGRE2	-0.19	10.92	-1.23	0.22345
PPIA	0.12	12.61	1.23	0.22356
DHFR	0.14	6.64	1.23	0.22361
UBL7-AS1	0.12	6.23	1.23	0.22363
RNF216	0.07	10.11	1.23	0.22378
UBASH3B	-0.10	8.13	-1.23	0.22383
ABCB7	-0.08	7.65	-1.23	0.22386
ZNF561-AS1	0.12	5.43	1.23	0.22388
SLC19A2	-0.13	5.09	-1.23	0.22406
KIF2A	-0.12	7.67	-1.23	0.22411
PHLDB2	-0.14	5.61	-1.23	0.22421
TKTL1	0.20	7.26	1.23	0.22432
C6orf136	0.08	7.67	1.23	0.22453
GSK3B	-0.11	9.06	-1.23	0.22454
TMEM44	0.14	5.37	1.23	0.22465
DNASE1L3	0.14	5.62	1.23	0.22473
CCDC189	-0.14	6.54	-1.23	0.22476
SLC38A5	0.23	9.84	1.23	0.22491
ARRDC1	0.11	9.98	1.23	0.22495
EZH1	-0.10	10.54	-1.23	0.22496
BACE1	-0.12	6.31	-1.23	0.22508
CKS2	0.15	5.58	1.23	0.22510
HNRNPKP4	-0.18	7.74	-1.23	0.22522
AUTS2	-0.13	8.06	-1.23	0.22531
CAPN1	0.09	11.77	1.23	0.22533
PEG10	-0.13	5.19	-1.23	0.22540
ERMP1	-0.11	7.51	-1.23	0.22543
MT-ATP8	-0.14	9.87	-1.23	0.22544
TIMMDC1	-0.06	8.64	-1.23	0.22546
STK38	-0.07	11.62	-1.23	0.22550
GPR155	0.16	9.15	1.23	0.22555
TGOLN2	-0.08	12.34	-1.23	0.22558
ABL2	0.10	7.97	1.23	0.22558
DNAJC8	0.09	10.01	1.23	0.22559
LINC02218	-0.20	5.96	-1.23	0.22561
AL136126.1	0.17	7.60	1.23	0.22562
CPSF1	0.12	10.86	1.23	0.22563
CCDC17	-0.20	6.77	-1.23	0.22567
EIF3A	-0.10	10.71	-1.23	0.22580

TMEM192	0.12	7.61	1.23	0.22597
NMT2	0.14	8.45	1.23	0.22605
PLCD3	-0.13	6.03	-1.22	0.22619
GFI1	-0.13	7.91	-1.22	0.22636
SCAF11	-0.11	9.80	-1.22	0.22656
VPS26A	-0.09	8.17	-1.22	0.22660
AC073861.1	0.19	9.57	1.22	0.22661
UBA2	-0.10	8.72	-1.22	0.22662
BX248409.1	0.13	5.22	1.22	0.22678
AL590560.3	0.23	5.22	1.22	0.22683
REEP3	-0.10	7.01	-1.22	0.22683
UHRF1BP1	-0.12	6.84	-1.22	0.22690
HAVCR2	-0.12	8.74	-1.22	0.22701
THAP8	0.13	6.69	1.22	0.22705
RPS27P21	0.11	5.49	1.22	0.22712
ABCG1	-0.16	9.12	-1.22	0.22738
LINC01146	-0.14	5.01	-1.22	0.22738
MRPS9	0.11	7.91	1.22	0.22753
KLKB1	0.14	4.98	1.22	0.22757
CCAR1	0.16	9.50	1.22	0.22764
PLEC	0.12	12.60	1.22	0.22765
CCDC88B	0.13	12.03	1.22	0.22775
GAPDHP60	0.15	6.62	1.22	0.22776
GLULP3	-0.18	5.46	-1.22	0.22783
TSPAN31	-0.08	8.09	-1.22	0.22791
CENPU	-0.14	5.29	-1.22	0.22797
RAB34	0.18	8.13	1.22	0.22799
TRBV20-1	0.19	8.41	1.22	0.22799
AL109741.2	0.17	6.02	1.22	0.22804
FLNA	0.09	15.09	1.22	0.22809
EIF3M	0.10	10.22	1.22	0.22810
AC021074.1	0.21	7.83	1.22	0.22839
EGLN2	-0.19	7.78	-1.22	0.22841
C1orf198	0.10	6.84	1.22	0.22844
BBOF1	0.24	6.97	1.22	0.22845
RTN4R	0.14	5.67	1.22	0.22852
SP140L	-0.11	8.62	-1.22	0.22858
APLF	0.12	5.22	1.22	0.22865
IGLV3-19	0.27	7.11	1.22	0.22866
AFDN	-0.19	6.43	-1.22	0.22866
PPP4R3B	-0.10	8.88	-1.22	0.22867
FKRP	0.09	7.47	1.22	0.22870
ASB8	0.10	9.82	1.22	0.22871
NDUFB1	0.12	8.28	1.22	0.22872
SLC4A2	0.09	9.66	1.22	0.22875
MTMR11	-0.13	8.09	-1.22	0.22885
LINC00570	0.27	6.26	1.22	0.22897
TTC26	-0.17	5.35	-1.22	0.22899
MAMLD1	-0.15	5.14	-1.22	0.22905
NXF1	-0.10	10.76	-1.22	0.22913

EXOC3	0.07	11.03	1.22	0.22913
IGHV5-51	0.26	7.97	1.22	0.22914
RPL8P2	0.15	5.76	1.22	0.22915
ZNF737	-0.13	6.62	-1.22	0.22917
ATP5F1B	0.07	12.39	1.22	0.22927
PDCD11	0.07	9.67	1.22	0.22927
SLC37A1	0.09	8.60	1.22	0.22939
ZNF362	0.07	8.85	1.22	0.22944
ZBTB24	0.12	7.54	1.22	0.22945
TMTC3	-0.12	5.36	-1.22	0.22949
HKDC1	0.19	6.72	1.22	0.22953
MRPS31	-0.09	7.40	-1.22	0.22976
DIS3L2	0.08	8.15	1.22	0.22977
DUSP4	-0.13	5.41	-1.21	0.22992
LRP1	-0.16	12.69	-1.21	0.22997
EFCAB12	-0.16	5.72	-1.21	0.23001
UHMK1	-0.11	8.82	-1.21	0.23002
CTCF	-0.08	9.73	-1.21	0.23004
KCNA3	-0.11	7.05	-1.21	0.23004
ITFG2	0.08	8.91	1.21	0.23020
CHST7	0.13	7.82	1.21	0.23025
SMIM1	0.29	6.49	1.21	0.23038
ADAT1	0.10	8.02	1.21	0.23049
PTMAP12	0.08	4.94	1.21	0.23054
TCL1A	0.30	9.29	1.21	0.23054
PPP4C	0.11	11.84	1.21	0.23061
INPP5A	-0.13	8.44	-1.21	0.23062
KIF3A	-0.12	6.03	-1.21	0.23075
EDARADD	0.16	5.91	1.21	0.23077
ZRSR2	0.12	8.32	1.21	0.23078
GAPDHP35	0.17	5.21	1.21	0.23081
IKZF1	-0.09	11.49	-1.21	0.23082
ZBTB3	0.13	7.07	1.21	0.23104
BCDIN3D	0.09	6.99	1.21	0.23108
ANKRD16	0.12	5.94	1.21	0.23115
C12orf76	-0.10	7.43	-1.21	0.23117
EIF2B5	0.07	9.72	1.21	0.23141
DDX1	-0.08	8.44	-1.21	0.23146
SNRPFP1	0.14	5.32	1.21	0.23150
CALCOCO1	-0.11	11.17	-1.21	0.23159
CDADC1	-0.11	6.37	-1.21	0.23190
CYFIP2	0.06	12.29	1.21	0.23204
AC093323.1	0.11	8.41	1.21	0.23209
CNOT6L	-0.09	9.04	-1.21	0.23209
KCTD3	-0.13	5.43	-1.21	0.23261
RIMBP3B	0.12	5.11	1.21	0.23265
ACSS2	-0.10	9.40	-1.21	0.23268
MT-TM	0.23	7.13	1.21	0.23277
LRR1	-0.09	6.65	-1.21	0.23283
TOPORS	-0.13	7.81	-1.21	0.23284

SH2D2A	0.17	9.66	1.21	0.23298
COX4I1P1	0.14	5.98	1.21	0.23303
FASTKD3	-0.10	6.05	-1.21	0.23305
TWSG1	-0.13	5.65	-1.21	0.23307
HINFP	0.07	8.67	1.21	0.23322
MTOR	-0.09	9.32	-1.21	0.23325
C2orf49	-0.10	7.13	-1.21	0.23348
AC114980.1	0.13	5.35	1.21	0.23351
WEE1	-0.13	6.27	-1.21	0.23356
MTSS1	-0.12	9.05	-1.20	0.23370
EPHB2	-0.17	6.41	-1.20	0.23380
AC063952.2	0.12	5.77	1.20	0.23390
CRYZ	-0.13	6.72	-1.20	0.23394
AL009179.2	-0.17	5.09	-1.20	0.23395
PRSS36	0.14	6.00	1.20	0.23395
ELAC2	0.07	9.93	1.20	0.23419
SCIMP	-0.13	9.48	-1.20	0.23427
AC010210.1	-0.16	5.28	-1.20	0.23437
NUDT8	-0.15	5.09	-1.20	0.23441
AC012146.1	0.12	5.79	1.20	0.23450
TMOD1	0.30	8.48	1.20	0.23454
UBE2SP1	0.11	5.01	1.20	0.23456
VPS37C	-0.12	8.96	-1.20	0.23457
TRIB2	-0.13	9.02	-1.20	0.23462
ATOX1	0.12	7.92	1.20	0.23468
DDX39B	-0.19	10.41	-1.20	0.23484
ZNF767P	-0.13	8.15	-1.20	0.23488
ADM	-0.24	9.10	-1.20	0.23516
SMC6	-0.12	7.34	-1.20	0.23525
PRIMPOL	-0.11	6.42	-1.20	0.23530
CD52	0.18	12.65	1.20	0.23546
RIPOR1	-0.09	10.44	-1.20	0.23547
TSC2	0.08	10.77	1.20	0.23548
FOLR2	-0.15	6.82	-1.20	0.23569
PLXNA2	-0.16	7.15	-1.20	0.23569
ZNF608	-0.24	5.24	-1.20	0.23573
ATAD3B	0.14	8.03	1.20	0.23573
ETNK2	0.11	5.56	1.20	0.23577
PROSER2	0.12	5.93	1.20	0.23578
HNRNPUL2	0.09	9.74	1.20	0.23582
RNF44	-0.10	11.24	-1.20	0.23582
TNNT1	0.30	6.16	1.20	0.23582
AC079781.5	0.11	5.44	1.20	0.23587
HMG20A	-0.08	8.22	-1.20	0.23588
AC080112.2	0.12	5.13	1.20	0.23598
PTBP1P	-0.13	5.36	-1.20	0.23603
PSMD3	0.09	9.85	1.20	0.23609
RPL5P17	0.18	7.23	1.20	0.23613
CEBPZOS	0.12	7.68	1.20	0.23644
RPL10AP2	0.15	6.53	1.20	0.23648

XXYLT1	0.09	7.50	1.20	0.23649
MYOF	-0.22	8.00	-1.20	0.23649
CYBRD1	-0.21	8.03	-1.20	0.23658
MTFMT	-0.10	6.97	-1.20	0.23662
DNAH10	-0.18	5.09	-1.20	0.23669
SEPSECS	-0.13	6.41	-1.20	0.23685
RPS29P5	0.14	5.82	1.20	0.23692
KIAA0040	-0.14	10.60	-1.20	0.23702
DOK1	0.11	10.20	1.20	0.23704
CRIP1P4	0.17	6.65	1.20	0.23706
FAM30A	0.16	8.02	1.20	0.23707
INTS9	0.09	8.69	1.20	0.23721
LINC01347	-0.17	5.66	-1.20	0.23722
RPL10P7	0.13	5.45	1.20	0.23726
ECRG4	0.15	4.96	1.20	0.23739
CENPX	0.12	7.93	1.20	0.23743
CCNA2	-0.14	4.94	-1.20	0.23745
MTHFR	0.08	9.74	1.20	0.23749
LGALS12	0.18	8.14	1.20	0.23752
TIMM17A	-0.09	8.16	-1.19	0.23755
TMIE	0.15	5.19	1.19	0.23762
NOP10	0.15	10.54	1.19	0.23763
PLK4	-0.09	4.98	-1.19	0.23765
PDZD4	0.16	9.92	1.19	0.23767
EPHA1	0.18	7.82	1.19	0.23778
STAMBP	-0.08	8.50	-1.19	0.23785
YPEL5	-0.12	10.48	-1.19	0.23786
TRBV7-3	0.22	6.23	1.19	0.23790
VPS39	-0.07	10.66	-1.19	0.23803
CD74	0.16	16.13	1.19	0.23810
MGAT2	-0.09	8.40	-1.19	0.23824
PPP3CA	-0.12	9.11	-1.19	0.23829
MVP	0.11	12.54	1.19	0.23829
RELA-DT	0.12	6.23	1.19	0.23836
LINC00663	-0.12	5.39	-1.19	0.23856
RPL3P3	0.16	6.25	1.19	0.23867
IGLC6	0.21	5.27	1.19	0.23896
NUCB2	0.12	8.75	1.19	0.23897
PPWD1	-0.11	8.07	-1.19	0.23898
POLE4	0.13	9.26	1.19	0.23921
RAB3GAP2	0.11	8.62	1.19	0.23922
BTG3	-0.10	6.26	-1.19	0.23930
C2orf88	-0.17	7.64	-1.19	0.23934
AMFR	-0.07	10.12	-1.19	0.23936
STX18-AS1	0.12	6.12	1.19	0.23937
NR1D1	0.14	8.44	1.19	0.23940
AC004233.2	-0.18	5.89	-1.19	0.23946
ZFAND2B	0.08	10.07	1.19	0.23946
MSH6	-0.08	8.27	-1.19	0.23953
POLM	0.10	8.92	1.19	0.23964

OTUD6B	-0.15	5.40	-1.19	0.23967
HSDL2	-0.14	8.93	-1.19	0.23968
TMA7	0.13	10.82	1.19	0.23971
ABCD3	-0.10	7.14	-1.19	0.23972
GARS-DT	-0.18	8.84	-1.19	0.23972
FOXK2	0.06	9.60	1.19	0.23979
CENPK	0.20	5.37	1.19	0.23981
S100A4	0.16	13.56	1.19	0.23988
CYP4F29P	0.47	6.48	1.19	0.23990
ARMCX7P	0.10	5.19	1.19	0.24002
CATSPERG	-0.12	6.17	-1.19	0.24010
TTYH3	-0.14	10.09	-1.19	0.24012
MYBL1	-0.14	7.50	-1.19	0.24014
MLXIP	0.09	11.09	1.19	0.24030
ELL2	-0.18	6.85	-1.19	0.24038
ADPRHL2	0.10	9.43	1.19	0.24052
ESCO1	-0.09	7.24	-1.19	0.24057
SETD3	-0.07	9.23	-1.19	0.24066
SERPINA1	-0.17	14.80	-1.19	0.24073
SOX4	0.12	7.13	1.19	0.24097
SELENOO	0.09	9.45	1.19	0.24117
HERPUD1	-0.08	10.18	-1.19	0.24122
LRRFIP1	0.11	12.10	1.19	0.24125
DHX9P1	-0.17	5.68	-1.19	0.24125
SLC6A9	0.25	5.99	1.19	0.24126
AC009220.1	0.13	6.25	1.19	0.24129
CDKN1A	0.16	9.27	1.19	0.24130
ALDOA	0.14	8.49	1.19	0.24134
SLC4A1AP	-0.08	8.35	-1.19	0.24140
MANBAL	0.09	8.16	1.18	0.24144
PDK3	-0.09	9.24	-1.18	0.24145
RPL15P2	0.12	6.30	1.18	0.24149
PTPRO	-0.14	6.63	-1.18	0.24154
NFS1	0.08	7.50	1.18	0.24164
FAM160B2	0.10	9.39	1.18	0.24165
YME1L1	-0.07	10.06	-1.18	0.24182
CEL	0.13	5.05	1.18	0.24182
FPGT	-0.13	6.34	-1.18	0.24186
APH1A	0.08	10.83	1.18	0.24194
UBE2MP1	0.13	5.69	1.18	0.24194
AC067945.1	-0.13	5.08	-1.18	0.24196
SORD2P	-0.13	5.49	-1.18	0.24204
FBXL17	0.10	7.65	1.18	0.24218
MSH2	-0.12	6.82	-1.18	0.24229
ACACA	-0.10	7.66	-1.18	0.24234
CNPPD1	0.12	11.41	1.18	0.24252
RPL7P26	0.12	5.60	1.18	0.24259
FARSB	0.10	8.11	1.18	0.24267
SFSWAP	0.07	9.64	1.18	0.24272
WNT10A	0.15	6.36	1.18	0.24273

WDR61	0.09	8.19	1.18	0.24279
MRPS33	0.11	7.32	1.18	0.24287
PPM1J	0.13	5.09	1.18	0.24299
ARHGEF2	-0.07	11.54	-1.18	0.24299
HSPA6	-0.18	10.95	-1.18	0.24308
AL161787.1	0.18	6.59	1.18	0.24325
AC064807.1	0.11	5.64	1.18	0.24326
NUP42	-0.08	7.26	-1.18	0.24340
CEBPG	-0.07	8.79	-1.18	0.24352
ZNF530	0.11	6.02	1.18	0.24359
PACSIN1	0.16	6.95	1.18	0.24365
BPTF	-0.11	9.63	-1.18	0.24373
ZNF300	-0.18	5.01	-1.18	0.24374
TAF8	-0.09	8.60	-1.18	0.24383
SCO1	0.07	8.22	1.18	0.24388
PSMB1	0.09	10.62	1.18	0.24396
AC078846.1	-0.11	5.58	-1.18	0.24398
CUL7	0.10	8.38	1.18	0.24401
ATP6V1D	-0.09	8.39	-1.18	0.24408
PTER	-0.12	6.12	-1.18	0.24408
AL139113.1	0.21	5.25	1.18	0.24409
EEF1GP1	0.13	5.48	1.18	0.24423
CBLB	-0.09	8.53	-1.18	0.24429
NUP54	-0.11	7.12	-1.18	0.24436
SNAPIN	0.08	8.08	1.18	0.24441
SNHG16	0.12	9.17	1.18	0.24443
AL355032.1	0.12	6.07	1.18	0.24452
DDX51	0.09	8.85	1.18	0.24460
ZMAT2	0.09	10.58	1.18	0.24464
ANP32E	-0.09	8.18	-1.18	0.24471
BLNK	0.17	7.47	1.18	0.24506
ANKS3	0.12	7.38	1.18	0.24513
FKBP8	0.27	14.77	1.18	0.24521
UPF1	-0.09	11.51	-1.18	0.24521
HMGN2	0.09	11.00	1.18	0.24523
AC011472.2	-0.28	8.78	-1.18	0.24527
IPO13	0.07	8.89	1.18	0.24529
AC006511.4	0.11	5.15	1.18	0.24537
SRD5A1	-0.10	7.06	-1.17	0.24547
PTMAP9	0.10	5.77	1.17	0.24559
TK1	0.15	6.33	1.17	0.24566
TREML2	-0.19	10.40	-1.17	0.24570
PNMA3	0.25	6.82	1.17	0.24571
PFN1P1	0.20	8.83	1.17	0.24577
FNTA	-0.07	8.83	-1.17	0.24577
NXPH4	0.14	5.24	1.17	0.24577
ZFAND4	-0.12	6.57	-1.17	0.24600
NAA15	-0.10	7.60	-1.17	0.24606
VAMP7	-0.10	7.88	-1.17	0.24615
KLF9	-0.14	7.34	-1.17	0.24617

PCNX4	-0.10	7.68	-1.17	0.24620
AC104339.1	0.19	4.97	1.17	0.24632
MDFIC	-0.14	8.04	-1.17	0.24639
ERMAP	-0.15	7.65	-1.17	0.24640
RPAP1	0.08	8.69	1.17	0.24654
BCAS2P2	-0.11	5.22	-1.17	0.24657
CEP85L	-0.11	7.21	-1.17	0.24679
TIGD1	-0.11	5.80	-1.17	0.24682
NLGN3	-0.16	6.96	-1.17	0.24697
SATB1	-0.15	9.39	-1.17	0.24708
SLC35A3	-0.10	6.45	-1.17	0.24719
KIAA1211L	-0.13	6.09	-1.17	0.24724
SLC25A38	0.08	9.27	1.17	0.24724
ANKRD6	-0.11	6.18	-1.17	0.24724
TM9SF1	0.13	5.38	1.17	0.24734
GTSF1	0.16	6.08	1.17	0.24738
U62317.4	-0.19	6.64	-1.17	0.24747
RNF126P1	0.12	5.12	1.17	0.24748
ABCF2	0.07	9.76	1.17	0.24751
CSPP1	0.16	8.05	1.17	0.24753
MPZL1	-0.17	9.64	-1.17	0.24754
AL138724.2	0.14	5.20	1.17	0.24757
CDC14B	-0.13	6.86	-1.17	0.24759
ENTPD3-AS1	0.14	5.79	1.17	0.24759
DPM1	-0.10	7.83	-1.17	0.24764
DDX11	0.30	8.24	1.17	0.24768
LLPH	0.08	7.99	1.17	0.24771
NIPAL3	0.09	8.92	1.17	0.24775
CSF1	-0.14	7.79	-1.17	0.24775
GNG5	-0.12	6.60	-1.17	0.24776
CRIP3	0.20	6.28	1.17	0.24777
GAS6	0.15	7.30	1.17	0.24788
EDA	0.18	5.45	1.17	0.24797
HNMT	-0.13	7.22	-1.17	0.24804
LONP2	0.08	9.36	1.17	0.24812
SLC9A7P1	-0.14	5.82	-1.17	0.24834
AHI1	-0.14	5.53	-1.17	0.24850
EIF4A3	0.07	9.97	1.17	0.24856
DNAJC7	0.08	9.92	1.17	0.24866
SACS	-0.14	6.53	-1.17	0.24872
KCND3	-0.14	5.01	-1.17	0.24896
CYTH2	0.09	9.65	1.17	0.24897
RBM25	0.15	10.41	1.17	0.24912
TSC22D1	-0.10	9.07	-1.17	0.24917
AL662907.1	0.12	5.41	1.17	0.24919
DUSP11	-0.08	7.92	-1.17	0.24921
RNF149	-0.17	11.85	-1.17	0.24921
SLC2A1-AS1	-0.16	5.24	-1.17	0.24928
TNFSF14	-0.14	9.56	-1.17	0.24929
RAMP1	0.14	5.36	1.16	0.24945

TRAV39	-0.15	5.39	-1.16	0.24947
KAT6A	-0.12	10.22	-1.16	0.24952
ELP1	-0.10	8.25	-1.16	0.24959
LINC02631	-0.16	5.47	-1.16	0.24959
TBX6	0.14	5.55	1.16	0.24965
AC107068.1	0.12	5.40	1.16	0.24967
MORN3	0.14	6.91	1.16	0.24970
CHST12	0.13	9.56	1.16	0.24980
RPS7P10	0.18	6.88	1.16	0.24986
DPP9	0.07	10.01	1.16	0.24990
SLC1A5	0.14	9.93	1.16	0.24991
KIF20B	0.14	6.46	1.16	0.24992
FAM50B	0.11	7.63	1.16	0.24997
KLHL36	0.06	9.80	1.16	0.24998
C22orf34	0.14	6.91	1.16	0.25008
PBLD	-0.11	5.48	-1.16	0.25015
BISPR	-0.15	7.50	-1.16	0.25017
KIF23	-0.11	5.07	-1.16	0.25019
WHAMM	-0.06	8.30	-1.16	0.25023
FRG1CP	0.14	7.27	1.16	0.25035
AC008074.2	-0.15	5.10	-1.16	0.25045
IGHV4-34	0.32	8.05	1.16	0.25052
LIG3	-0.08	8.13	-1.16	0.25053
HBG1	0.51	7.03	1.16	0.25057
S100B	0.38	6.79	1.16	0.25057
TRNT1	-0.12	6.52	-1.16	0.25059
COIL	0.10	7.11	1.16	0.25069
TPT1P4	0.17	9.03	1.16	0.25079
SEPTIN7P2	0.14	6.74	1.16	0.25080
KIAA0930	0.12	11.57	1.16	0.25102
SH3TC1	0.11	9.64	1.16	0.25114
ZMPSTE24	-0.10	7.78	-1.16	0.25120
MYO9A	0.10	8.03	1.16	0.25134
RPS29P3	0.18	6.45	1.16	0.25148
C3orf62	-0.14	8.90	-1.16	0.25153
GLCE	-0.12	6.17	-1.16	0.25160
FCGR1CP	-0.24	5.91	-1.16	0.25177
SNHG3	-0.12	7.59	-1.16	0.25214
PDCL3	0.11	7.47	1.16	0.25224
AC022706.1	0.15	6.93	1.16	0.25236
AC006333.1	-0.12	5.32	-1.16	0.25247
CTSL	-0.20	7.59	-1.16	0.25248
ENKD1	0.11	7.42	1.16	0.25252
TRAV16	0.13	5.40	1.16	0.25254
RPL9P7	0.18	8.93	1.16	0.25258
NLGN2	0.13	6.23	1.16	0.25258
EBPL	0.10	7.21	1.16	0.25271
FAM193A	-0.06	9.25	-1.16	0.25277
VSIG10L	0.13	5.08	1.16	0.25278
TP53BP1	-0.08	8.34	-1.16	0.25279

GNB2	0.13	13.10	1.16	0.25282
P3H1	0.08	9.03	1.16	0.25285
FAM104B	0.11	6.81	1.16	0.25287
SPIRE1	-0.11	5.39	-1.16	0.25289
HHAT	-0.09	5.61	-1.16	0.25303
SMAD5	-0.11	6.82	-1.16	0.25309
PTPRN	0.15	5.53	1.16	0.25312
CDKN2A	0.13	5.48	1.16	0.25323
HOXA1	-0.11	5.31	-1.16	0.25325
VWF	0.22	7.41	1.16	0.25333
VASH1-AS1	0.11	6.89	1.16	0.25334
POU6F1	0.14	8.04	1.16	0.25339
MS4A3	-0.21	6.33	-1.15	0.25342
SUV39H1	0.10	7.95	1.15	0.25350
MECP2	0.06	10.78	1.15	0.25355
PSME3	-0.07	10.23	-1.15	0.25359
P3H2	-0.17	4.91	-1.15	0.25377
NPIPB3	-0.18	7.77	-1.15	0.25391
TRIM58	0.29	11.17	1.15	0.25397
CD151	0.20	10.02	1.15	0.25403
DISP1	-0.12	6.03	-1.15	0.25430
CLUH	0.09	9.37	1.15	0.25439
TBCA	0.10	8.55	1.15	0.25448
VPS13D	-0.07	9.38	-1.15	0.25449
MT-TV	0.20	6.43	1.15	0.25454
AC026877.1	-0.19	6.95	-1.15	0.25456
UBE2V2	0.09	7.80	1.15	0.25458
FAM53A	0.14	4.98	1.15	0.25461
FLT3LG	0.15	5.35	1.15	0.25462
XRN2	-0.07	10.58	-1.15	0.25463
PKD1	0.10	9.49	1.15	0.25464
EPRS1	0.08	9.90	1.15	0.25484
STIP1	0.08	10.48	1.15	0.25491
EMC3-AS1	0.14	5.93	1.15	0.25519
MTG2	0.08	9.27	1.15	0.25525
KNDC1	-0.21	5.66	-1.15	0.25530
RBMS2	-0.13	6.91	-1.15	0.25535
IL1B	-0.19	9.40	-1.15	0.25539
SCRIB	0.09	8.83	1.15	0.25540
AC022966.1	0.08	9.65	1.15	0.25548
UQCRQ	0.11	9.15	1.15	0.25569
DOCK9-DT	0.13	5.29	1.15	0.25570
ETS1	-0.12	11.97	-1.15	0.25573
ZNF710	-0.10	9.87	-1.15	0.25573
C9orf139	-0.16	8.62	-1.15	0.25574
PPP4R2	-0.13	8.13	-1.15	0.25615
RNF207	-0.17	5.37	-1.15	0.25615
PKN3	-0.14	6.33	-1.15	0.25616
DZANK1	-0.11	5.43	-1.15	0.25619
CALR	0.10	12.82	1.15	0.25625

FOS	-0.20	10.18	-1.15	0.25630
ST13	0.09	10.53	1.15	0.25645
CYB5R1	0.08	9.58	1.15	0.25652
LINC00921	-0.11	7.30	-1.15	0.25662
AC112496.1	0.22	5.54	1.15	0.25670
SPG7	0.09	10.70	1.15	0.25672
RPL24P4	0.16	7.50	1.15	0.25675
C17orf97	-0.29	5.28	-1.15	0.25679
SF1	0.05	12.26	1.15	0.25683
AC015871.3	-0.17	7.07	-1.15	0.25683
THNSL2	-0.23	5.76	-1.15	0.25699
RECQL4	0.12	6.03	1.15	0.25703
SMPD4	0.08	9.45	1.15	0.25713
NPIPA7	-0.11	6.30	-1.15	0.25722
ABR	-0.07	11.40	-1.15	0.25734
LRRC23	0.10	6.57	1.15	0.25738
BRD2	-0.05	12.15	-1.15	0.25746
IL6ST	-0.15	8.24	-1.14	0.25760
DEPDC5	0.07	8.54	1.14	0.25767
AC144652.1	0.10	5.88	1.14	0.25770
ETS2	-0.15	9.59	-1.14	0.25790
ADRA2A	-0.13	5.07	-1.14	0.25799
LARP4	-0.10	6.67	-1.14	0.25801
AC026410.1	0.10	5.43	1.14	0.25808
AC092017.2	0.11	5.70	1.14	0.25817
MRPL44	-0.07	8.38	-1.14	0.25824
B4GALT4	-0.09	7.36	-1.14	0.25824
HS6ST1	0.08	8.27	1.14	0.25825
LAMTOR4	0.14	11.69	1.14	0.25828
JTB	0.15	7.41	1.14	0.25843
AC097637.2	0.15	5.14	1.14	0.25846
BCL2L1	0.26	12.38	1.14	0.25847
BTN2A2	-0.09	8.46	-1.14	0.25862
MTIF2	0.09	7.68	1.14	0.25872
ZMYM6	-0.09	6.99	-1.14	0.25881
NME7	0.11	5.92	1.14	0.25900
ZBED3	0.14	6.33	1.14	0.25910
PSME4	-0.10	9.36	-1.14	0.25914
TCTN1	-0.10	6.68	-1.14	0.25918
MCU	-0.08	7.49	-1.14	0.25921
PSAT1	-0.17	5.84	-1.14	0.25936
SSRP1	0.08	10.72	1.14	0.25958
AC091729.3	0.16	5.56	1.14	0.25959
TFDP1	-0.13	9.09	-1.14	0.25961
MAGEE1	-0.12	6.16	-1.14	0.25964
RSF1	0.12	9.08	1.14	0.25971
FAM133DP	0.14	5.69	1.14	0.25975
BX322650.1	-0.14	5.05	-1.14	0.25984
PLCL2	-0.09	9.69	-1.14	0.25990
TMEM216	0.11	7.82	1.14	0.26003

AC002091.2	-0.15	5.06	-1.14	0.26005
PREB	0.07	9.94	1.14	0.26016
EPHX2	0.19	8.21	1.14	0.26020
FNBP1L	-0.12	5.11	-1.14	0.26032
FRMD3	-0.14	6.01	-1.14	0.26034
XPO7	-0.17	9.52	-1.14	0.26054
RAD51C	-0.12	6.79	-1.14	0.26057
GVINP1	-0.12	8.40	-1.14	0.26064
LPP	0.13	8.98	1.14	0.26067
LIMK2	-0.20	11.29	-1.14	0.26068
PTBP1	0.06	11.55	1.14	0.26075
AC107890.1	0.12	5.13	1.14	0.26083
DNMT3A	-0.09	9.08	-1.14	0.26090
ZAP70	0.11	11.91	1.14	0.26100
AC107075.1	0.15	6.36	1.13	0.26164
GINS3	-0.10	5.86	-1.13	0.26164
ENTPD6	0.10	9.81	1.13	0.26164
AP001160.1	0.17	5.05	1.13	0.26168
SIGLEC12	-0.25	5.25	-1.13	0.26193
JUP	-0.31	9.42	-1.13	0.26193
ELOVL1	0.07	10.55	1.13	0.26196
FBXO25	0.11	8.02	1.13	0.26197
GM2A	-0.13	9.49	-1.13	0.26197
SFMBT1	0.10	7.36	1.13	0.26199
EMP1	-0.16	5.34	-1.13	0.26215
CHMP4B	0.09	11.25	1.13	0.26219
CADM4	-0.16	6.40	-1.13	0.26223
RELA	0.06	10.92	1.13	0.26232
ZNF527	-0.10	5.79	-1.13	0.26233
AL139020.1	0.21	6.22	1.13	0.26236
ARF5	0.09	10.89	1.13	0.26238
ADTRP	0.20	6.18	1.13	0.26242
TTC12	0.12	6.81	1.13	0.26266
GIMAP6	-0.10	10.91	-1.13	0.26281
ERAL1	0.09	8.69	1.13	0.26291
WDR19	-0.12	6.56	-1.13	0.26300
SH3YL1	-0.15	7.79	-1.13	0.26301
NCBP1	-0.08	7.95	-1.13	0.26307
NIP7	-0.09	7.95	-1.13	0.26309
DHX58	-0.21	9.08	-1.13	0.26315
CCDC59	0.09	7.55	1.13	0.26317
DHRS7	-0.10	10.07	-1.13	0.26320
EGFL7	0.13	5.77	1.13	0.26330
ANKRD44-IT1	0.24	6.68	1.13	0.26347
C1RL	-0.15	9.96	-1.13	0.26348
POLR3K	0.10	7.51	1.13	0.26355
GIMAP5	-0.16	6.76	-1.13	0.26363
POGLUT1	-0.09	7.23	-1.13	0.26393
RHEBL1	0.10	5.83	1.13	0.26398
FAM122A	-0.09	7.10	-1.13	0.26413

DHX33	-0.09	7.12	-1.13	0.26414
PRR3	0.09	7.64	1.13	0.26417
KREMEN1	-0.30	8.94	-1.13	0.26426
TP53I3	-0.14	6.78	-1.13	0.26429
IL17RE	-0.18	5.55	-1.13	0.26430
CLEC16A	0.06	9.15	1.13	0.26442
GCM1	-0.14	4.98	-1.13	0.26448
GSR	-0.07	9.10	-1.13	0.26448
GNMT	0.16	6.01	1.13	0.26455
STK16	0.09	9.18	1.13	0.26470
SFPQ	-0.06	10.61	-1.13	0.26476
TIPIN	-0.10	5.32	-1.13	0.26478
CCDC32	0.07	8.54	1.13	0.26479
AC011462.5	0.12	6.12	1.13	0.26493
EXOSC2	0.08	7.99	1.13	0.26495
HEXD	0.13	9.18	1.13	0.26499
ZNF618	-0.16	6.06	-1.13	0.26515
RIPOR3	0.21	6.17	1.13	0.26517
CCNB2	-0.12	5.17	-1.13	0.26522
AVIL	-0.20	7.69	-1.13	0.26526
SRD5A3	0.12	6.65	1.13	0.26536
CDK5RAP2	0.06	9.61	1.13	0.26551
MAGT1	-0.08	8.46	-1.13	0.26553
RAD54L	-0.13	4.94	-1.13	0.26557
ACAT2	0.07	7.72	1.13	0.26562
AC006254.1	-0.15	6.01	-1.13	0.26569
UNC5CL	-0.14	5.99	-1.13	0.26571
AGAP3	0.11	9.42	1.13	0.26572
HS1BP3	0.10	9.52	1.13	0.26575
MAP4K4	-0.10	11.02	-1.12	0.26580
CYP2S1	0.19	7.52	1.12	0.26587
TATDN2	0.09	9.28	1.12	0.26616
ZHX3	0.09	7.06	1.12	0.26618
ZNF484	-0.11	5.72	-1.12	0.26619
GP1BA	0.19	7.83	1.12	0.26620
FCRLA	0.21	8.58	1.12	0.26637
IGKC	0.27	13.52	1.12	0.26638
PI4KAP1	-0.24	7.46	-1.12	0.26656
IDH2	0.11	10.15	1.12	0.26657
MED29	0.06	9.76	1.12	0.26679
RPL21P116	0.14	5.76	1.12	0.26702
ACTN4	0.11	12.87	1.12	0.26703
SLC38A7	0.11	8.07	1.12	0.26706
RMDN1	0.07	7.56	1.12	0.26707
EIF4B	0.07	12.06	1.12	0.26711
RPL3P9	0.11	5.42	1.12	0.26712
HDAC8	0.08	6.40	1.12	0.26730
AC138035.1	-0.23	7.20	-1.12	0.26748
MSTO1	0.11	7.17	1.12	0.26752
MIER3	-0.13	5.92	-1.12	0.26753

RPSAP54	0.11	6.48	1.12	0.26759
AC009120.2	-0.15	6.77	-1.12	0.26759
DIDO1	-0.09	10.06	-1.12	0.26759
PCOLCE	0.13	6.57	1.12	0.26770
CD34	0.17	5.30	1.12	0.26787
ITGAM	-0.15	12.34	-1.12	0.26797
RASA4CP	0.11	6.60	1.12	0.26799
PEF1	0.10	10.30	1.12	0.26801
RAPH1	-0.11	5.20	-1.12	0.26801
VDAC1	-0.08	9.41	-1.12	0.26806
POFUT1	-0.09	8.31	-1.12	0.26810
SRP9	-0.10	8.37	-1.12	0.26812
REPS2	-0.20	8.91	-1.12	0.26815
FGFRL1	-0.14	7.23	-1.12	0.26817
RNF2	-0.11	7.03	-1.12	0.26825
TM9SF4	-0.07	10.49	-1.12	0.26825
TOR4A	-0.10	9.19	-1.12	0.26834
LFNG	0.11	12.15	1.12	0.26843
CD300A	-0.12	11.58	-1.12	0.26844
MPHOSPH9	-0.14	6.71	-1.12	0.26851
GNAI2	0.10	14.17	1.12	0.26855
MGAT4A	-0.10	9.27	-1.12	0.26865
HIF1AN	-0.06	9.89	-1.12	0.26880
CENPO	0.10	6.90	1.12	0.26881
ICE2	-0.10	7.66	-1.12	0.26893
SMAP2	-0.16	13.40	-1.12	0.26893
F2R	-0.17	7.79	-1.12	0.26909
ANAPC4	-0.08	7.70	-1.12	0.26914
HSPE1P18	0.23	6.49	1.12	0.26915
SRSF6	-0.07	9.50	-1.12	0.26916
IGHV3-7	0.29	7.82	1.12	0.26938
TAX1BP1	-0.08	9.97	-1.12	0.26944
CD70	0.15	5.58	1.12	0.26949
CACNA1D	-0.13	5.02	-1.12	0.26956
METTL17	-0.11	8.59	-1.12	0.26962
SLC50A1	0.10	8.70	1.12	0.26970
SND1	0.09	11.46	1.12	0.26973
TNNI2	0.14	7.69	1.12	0.26974
GNPDA1	0.09	8.31	1.12	0.26975
TRAPPC4	0.10	8.88	1.12	0.26977
AC015813.6	-0.11	6.16	-1.12	0.26997
FRA10AC1	0.10	7.15	1.11	0.27007
KDM5B	-0.11	8.61	-1.11	0.27030
TPRG1L	0.08	10.79	1.11	0.27033
GAPDHP63	0.12	5.78	1.11	0.27037
IDUA	0.12	8.02	1.11	0.27042
CD22	0.20	10.19	1.11	0.27056
FAM107B	-0.07	10.68	-1.11	0.27056
NAPG	-0.08	7.49	-1.11	0.27064
QDPR	0.10	7.96	1.11	0.27068

FLT4	0.19	6.67	1.11	0.27077
ZNF286A	0.11	6.05	1.11	0.27087
PAQR8	0.11	8.54	1.11	0.27097
ZNF558	-0.12	6.54	-1.11	0.27109
C1orf216	0.09	7.66	1.11	0.27113
MRTFA	0.07	11.37	1.11	0.27132
LINC01238	-0.15	5.91	-1.11	0.27138
MTHFS	0.13	5.54	1.11	0.27138
EPPK1	0.18	6.00	1.11	0.27153
PECAM1	-0.10	13.01	-1.11	0.27155
CROCC	0.12	8.29	1.11	0.27157
C19orf44	0.12	6.00	1.11	0.27158
SNRPF	0.11	8.40	1.11	0.27160
YLPM1	-0.07	9.59	-1.11	0.27163
ARRDC1-AS1	0.11	7.24	1.11	0.27167
C20orf27	0.13	10.04	1.11	0.27171
RDH5	-0.15	5.36	-1.11	0.27181
AC023161.2	-0.11	5.30	-1.11	0.27183
ZC4H2	0.10	6.42	1.11	0.27187
SYT17	0.15	6.04	1.11	0.27197
IGLV3-1	0.24	8.14	1.11	0.27203
AL365357.1	0.31	7.99	1.11	0.27207
PM20D2	-0.11	6.19	-1.11	0.27218
ATP5MD	0.12	7.82	1.11	0.27221
ZNF318	-0.10	8.40	-1.11	0.27228
FAM118A	0.26	8.52	1.11	0.27232
USP47	-0.08	8.96	-1.11	0.27235
LINC01765	-0.17	5.72	-1.11	0.27237
IMPAD1	-0.09	8.06	-1.11	0.27239
ZNF790-AS1	0.12	5.37	1.11	0.27246
ACTN4P1	0.12	6.62	1.11	0.27247
LINC002481	0.12	7.34	1.11	0.27252
PRR34	0.09	5.41	1.11	0.27254
POU2AF1	0.16	8.55	1.11	0.27264
AC015726.1	-0.13	5.54	-1.11	0.27267
TRIM59	-0.09	6.47	-1.11	0.27270
AF201337.1	-0.12	5.45	-1.11	0.27279
ZNF785	0.10	7.24	1.11	0.27280
ZNF738	-0.13	5.51	-1.11	0.27284
PLCL1	-0.14	5.72	-1.11	0.27286
GTPBP1	-0.10	10.46	-1.11	0.27289
RAD50	0.13	5.23	1.11	0.27292
CCDC138	0.10	5.26	1.11	0.27295
MAD2L1BP	-0.09	8.67	-1.11	0.27319
DDAH2	0.11	9.24	1.11	0.27321
ABCC4	-0.16	6.70	-1.11	0.27328
CCN3	-0.19	6.58	-1.11	0.27347
TSPOAP1	-0.17	8.59	-1.11	0.27357
IGLV9-49	0.18	5.45	1.11	0.27371
MIGA1	0.10	6.80	1.11	0.27382

TESC	0.25	11.30	1.11	0.27399
DNAJC18	0.10	6.03	1.11	0.27401
AC010186.3	-0.11	7.01	-1.11	0.27404
ZNF639	-0.10	7.02	-1.11	0.27405
CDC26	0.09	7.11	1.11	0.27409
LNX2	-0.13	5.96	-1.11	0.27410
SRP9P1	-0.13	5.09	-1.11	0.27410
SMIM12	0.09	9.05	1.11	0.27414
NAA30	-0.11	7.00	-1.11	0.27415
GMEB1	0.09	8.45	1.11	0.27419
DCUN1D3	-0.09	6.82	-1.11	0.27423
IQCC	0.12	5.55	1.11	0.27427
AATBC	-0.17	8.47	-1.10	0.27446
VDR	-0.12	8.69	-1.10	0.27455
TTC39B	0.12	7.43	1.10	0.27455
DDX31	0.08	7.54	1.10	0.27455
ABCC6	-0.14	6.27	-1.10	0.27456
PCED1B-AS1	0.12	10.51	1.10	0.27461
PITPNA	-0.09	10.99	-1.10	0.27467
PKD1P5	0.22	5.26	1.10	0.27475
DOLK	0.09	7.35	1.10	0.27494
FOXN2	-0.12	7.29	-1.10	0.27494
MLLT6	0.07	12.31	1.10	0.27499
ZNF718	-0.12	5.87	-1.10	0.27514
NUP160	-0.12	8.03	-1.10	0.27515
MT-TP	0.14	6.00	1.10	0.27543
SCD5	0.13	5.51	1.10	0.27559
SNHG15	0.09	7.85	1.10	0.27571
NUDT4	-0.13	7.57	-1.10	0.27579
APOL4	-0.22	5.08	-1.10	0.27585
ZNF566	0.11	6.48	1.10	0.27610
VILL	0.08	8.84	1.10	0.27611
AL359532.1	-0.15	5.22	-1.10	0.27612
ATP6AP1	0.08	11.52	1.10	0.27613
LINC00299	0.14	6.57	1.10	0.27620
H3P36	-0.14	7.02	-1.10	0.27624
RPL35P1	0.13	6.26	1.10	0.27646
APOM	0.12	5.59	1.10	0.27666
PPT2	0.11	6.31	1.10	0.27681
SLC49A4	-0.15	7.01	-1.10	0.27681
GOSR1	-0.06	8.53	-1.10	0.27681
MRPL3	0.09	7.93	1.10	0.27687
MOB3A	0.11	13.13	1.10	0.27696
ALG1	0.10	8.32	1.10	0.27697
RPS3AP26	0.19	8.48	1.10	0.27705
KIF21A	-0.15	6.78	-1.10	0.27707
BRD8	-0.09	9.50	-1.10	0.27707
SLC39A9	-0.07	8.81	-1.10	0.27719
ST13P19	0.11	5.03	1.10	0.27729
GDE1	0.15	10.53	1.10	0.27730

DUT	0.08	8.71	1.10	0.27730
EIF2S3B	-0.17	6.18	-1.10	0.27731
HCP5	-0.07	11.12	-1.10	0.27733
NRIR	-0.24	5.39	-1.10	0.27750
DCAKD	0.08	8.21	1.10	0.27751
RHBDL1	0.13	5.04	1.10	0.27761
LAMP5	-0.16	6.01	-1.10	0.27786
PSMB7	0.10	9.93	1.10	0.27791
ACOT2	-0.11	6.15	-1.10	0.27794
CSF2RA	-0.14	10.47	-1.10	0.27796
EMC7	-0.08	8.75	-1.10	0.27796
PDIA6	-0.08	10.31	-1.10	0.27803
QPCT	-0.20	10.28	-1.10	0.27807
LMAN1	-0.13	7.34	-1.10	0.27811
NDUFA4	0.10	9.56	1.10	0.27812
SHFL	-0.12	10.40	-1.10	0.27812
FPR3	-0.18	5.60	-1.10	0.27817
IFI6	-0.32	9.90	-1.10	0.27832
AC009362.1	0.11	5.24	1.10	0.27832
PSPC1	-0.06	8.64	-1.10	0.27839
BMP1	0.12	6.79	1.10	0.27849
SNX22	0.19	7.95	1.09	0.27866
ZSWIM3	0.08	7.44	1.09	0.27890
USP18	-0.25	5.53	-1.09	0.27913
RRN3	-0.09	8.02	-1.09	0.27920
ILF2	0.08	9.95	1.09	0.27921
CMC1	0.12	7.00	1.09	0.27922
STRAP	-0.05	9.12	-1.09	0.27926
MOB3C	-0.09	9.28	-1.09	0.27928
H3P47	-0.14	7.33	-1.09	0.27932
TRIP10	0.12	6.56	1.09	0.27943
RANBP3	0.06	10.24	1.09	0.27946
IPO11	-0.11	6.34	-1.09	0.27955
ZNF81	-0.12	5.63	-1.09	0.27956
PFKFB3	-0.23	9.88	-1.09	0.27962
AC017104.1	0.13	5.54	1.09	0.27964
ST13P5	0.10	5.06	1.09	0.27964
EVC	-0.15	4.96	-1.09	0.27966
HOMER2	0.15	6.40	1.09	0.27967
SNX11	-0.10	10.03	-1.09	0.27972
PMS2P4	-0.12	5.08	-1.09	0.27978
TRAPPC13	-0.10	6.57	-1.09	0.27985
KHK	0.12	6.83	1.09	0.27991
PNPT1	-0.14	7.26	-1.09	0.27992
GTF2I	-0.12	8.19	-1.09	0.27992
TMEM140	-0.15	11.04	-1.09	0.27996
ANKRD55	0.19	6.69	1.09	0.28007
TRAF1	0.11	9.83	1.09	0.28008
AC092324.2	0.16	6.83	1.09	0.28010
BLOC1S2	-0.09	8.59	-1.09	0.28011

C3AR1	-0.16	9.18	-1.09	0.28013
ASB7	-0.09	7.61	-1.09	0.28014
CRELD1	-0.15	5.69	-1.09	0.28015
ISM1	0.18	5.47	1.09	0.28016
C11orf95	-0.09	6.13	-1.09	0.28016
SLC45A4	-0.17	10.51	-1.09	0.28019
IDI1	-0.14	9.18	-1.09	0.28020
COMMD9	0.10	9.48	1.09	0.28029
DHRS7B	0.09	7.95	1.09	0.28033
PLCXD1	0.12	8.11	1.09	0.28038
LANCL3	-0.13	5.01	-1.09	0.28044
AIM2	-0.15	7.34	-1.09	0.28049
PMS2P1	0.08	7.10	1.09	0.28060
TIMM8B	0.12	7.82	1.09	0.28067
EEF1AKNMT	0.07	9.25	1.09	0.28070
RAD9A	0.09	8.83	1.09	0.28073
USP37	-0.10	6.60	-1.09	0.28076
ST6GALNAC3	-0.17	5.34	-1.09	0.28076
IL18R1	-0.28	7.38	-1.09	0.28098
LINC01232	-0.10	6.32	-1.09	0.28102
RTL6	-0.11	8.05	-1.09	0.28116
ANKRD10	-0.07	9.34	-1.09	0.28125
SLC24A3	0.18	6.27	1.09	0.28125
MYO7B	0.20	7.91	1.09	0.28130
LRPAP1	0.09	11.22	1.09	0.28133
UBR7	-0.07	7.83	-1.09	0.28133
PPA2	0.08	8.12	1.09	0.28145
LRSAM1	0.07	8.97	1.09	0.28163
ALYREF	0.10	9.15	1.09	0.28165
AC103563.7	0.12	5.50	1.09	0.28172
PPAN	0.09	7.01	1.09	0.28174
NCALD	-0.13	7.93	-1.09	0.28174
ERLIN1	-0.17	8.23	-1.09	0.28176
MMP25-AS1	0.16	8.81	1.09	0.28177
SPR	0.13	5.44	1.09	0.28179
HPS6	0.09	9.53	1.09	0.28205
FKBP11	0.09	8.41	1.09	0.28213
AKAP8L	0.07	10.28	1.09	0.28220
TESMIN	-0.10	6.31	-1.09	0.28221
SMIM8	0.09	6.31	1.09	0.28225
SMIM25	-0.18	11.13	-1.09	0.28226
RBSN	-0.07	8.03	-1.09	0.28237
FBXL16	0.14	7.65	1.09	0.28256
AL732372.2	-0.20	6.83	-1.09	0.28258
CCNB1IP1	0.09	7.72	1.09	0.28262
LINC00526	-0.11	5.46	-1.09	0.28290
USP44	0.14	5.07	1.09	0.28290
DHX40	-0.08	7.76	-1.09	0.28295
ETF1	-0.05	9.56	-1.09	0.28295
KIF1C	0.07	9.47	1.08	0.28306

CDKN1B	-0.09	9.91	-1.08	0.28307
GLUD2	-0.14	5.48	-1.08	0.28330
SLC5A2	0.13	5.00	1.08	0.28332
PDZK1IP1	0.27	10.49	1.08	0.28341
L1CAM	0.11	5.42	1.08	0.28349
AC106028.3	-0.13	5.39	-1.08	0.28357
RBBP8	-0.09	6.62	-1.08	0.28363
AC138932.4	0.10	6.01	1.08	0.28363
IFT43	0.11	6.85	1.08	0.28363
AMDHD1	0.13	4.87	1.08	0.28374
MORC2-AS1	0.16	5.59	1.08	0.28379
H2BC5	-0.17	6.86	-1.08	0.28389
CCR5	-0.13	8.58	-1.08	0.28398
NPEPL1	0.14	8.47	1.08	0.28404
LPCAT1	-0.09	11.51	-1.08	0.28406
YES1	-0.11	5.53	-1.08	0.28407
DNAAF5	0.08	7.64	1.08	0.28408
BTF3P4	0.09	5.48	1.08	0.28408
AC134878.2	0.16	5.64	1.08	0.28448
MAP9	0.17	5.76	1.08	0.28456
NPHP3	0.13	6.98	1.08	0.28458
H3-3B	-0.13	13.62	-1.08	0.28467
ZNF416	0.11	6.45	1.08	0.28473
CR1L	-0.12	5.03	-1.08	0.28478
SGMS1-AS1	0.10	6.68	1.08	0.28478
VENTX	-0.15	7.93	-1.08	0.28492
SLA	-0.16	11.97	-1.08	0.28506
ZBTB4	0.08	10.11	1.08	0.28516
LZTS3	0.15	5.49	1.08	0.28519
RAB40B	0.12	6.10	1.08	0.28519
RASGRP2	0.09	12.54	1.08	0.28550
TRIM26	-0.07	10.20	-1.08	0.28551
FYB1	0.14	12.15	1.08	0.28554
SLC41A1	-0.10	7.48	-1.08	0.28558
LINC00467	0.08	5.59	1.08	0.28569
CPLANE2	0.12	6.11	1.08	0.28569
CTSW	0.17	12.10	1.08	0.28581
SERPINB6	0.11	10.28	1.08	0.28582
FIP1L1	0.07	8.43	1.08	0.28596
RNF185	-0.07	9.17	-1.08	0.28600
AP000787.1	0.16	6.41	1.08	0.28617
NBPF11	-0.13	6.93	-1.08	0.28621
JMJD7-PLA2G4B	-0.19	6.57	-1.08	0.28634
OVGP1	-0.14	6.02	-1.08	0.28638
LGR6	-0.19	7.50	-1.08	0.28644
ATRN	-0.09	8.17	-1.08	0.28651
TPT1P9	0.15	9.80	1.08	0.28659
INPP1	-0.06	7.34	-1.08	0.28662
RPL34P34	0.15	6.74	1.08	0.28668
MLH3	0.11	8.34	1.08	0.28669

SORT1	-0.17	9.47	-1.08	0.28673
PRELID1P1	0.11	5.78	1.08	0.28697
HLA-DRB9	0.14	5.70	1.08	0.28698
HSP90AA2P	0.14	7.38	1.08	0.28703
AL450384.2	-0.13	5.39	-1.08	0.28707
TRBV6-5	0.16	7.18	1.08	0.28730
ZNF768	0.11	7.91	1.08	0.28731
CCT5P2	-0.14	5.51	-1.08	0.28731
PJA1	0.09	8.15	1.08	0.28734
IGLV7-43	0.24	6.13	1.08	0.28736
AC009119.1	-0.13	5.79	-1.07	0.28739
SEC14L2	0.15	5.13	1.07	0.28752
TMEM229B	0.09	9.48	1.07	0.28753
PDGFRB	-0.21	7.15	-1.07	0.28780
ZC3H18	0.08	10.14	1.07	0.28792
FAM32A	0.07	10.34	1.07	0.28797
ZNF649	-0.13	5.98	-1.07	0.28802
APIP	-0.07	7.01	-1.07	0.28815
ZNF148	-0.10	8.03	-1.07	0.28816
RIDA	0.09	6.04	1.07	0.28829
SH3KBP1	-0.06	11.44	-1.07	0.28833
TRBJ2-1	0.14	6.03	1.07	0.28835
DCAF13	-0.09	7.43	-1.07	0.28838
RHBDF2	-0.11	10.63	-1.07	0.28845
E2F4	0.06	10.86	1.07	0.28846
AC008443.1	-0.09	5.83	-1.07	0.28847
EIF4EBP3	0.12	5.09	1.07	0.28892
GABBR1	-0.18	9.58	-1.07	0.28901
TIGD6	-0.11	5.71	-1.07	0.28918
MED20	-0.09	7.29	-1.07	0.28931
HERC6	-0.17	7.81	-1.07	0.28936
MNT	0.08	9.68	1.07	0.28958
NFYB	-0.09	7.20	-1.07	0.28968
TUT1	0.11	7.44	1.07	0.28970
AL109766.1	-0.14	5.16	-1.07	0.29000
TEC	-0.12	5.58	-1.07	0.29001
SELP	0.16	7.59	1.07	0.29005
ATP5PDP4	0.10	5.99	1.07	0.29034
STAU2	-0.10	6.94	-1.07	0.29048
LINC00092	-0.14	5.08	-1.07	0.29051
GPX3	-0.19	6.40	-1.07	0.29051
TMEM127	-0.08	11.43	-1.07	0.29055
CARD19	0.10	9.56	1.07	0.29062
NBEAL2	-0.16	13.34	-1.07	0.29063
TRAV8-6	-0.13	6.31	-1.07	0.29084
AC007182.2	0.17	7.61	1.07	0.29087
ZNF219	0.13	6.04	1.07	0.29091
CTNNBIP1	0.09	8.63	1.07	0.29099
C6orf226	0.11	6.10	1.07	0.29106
DBI	0.10	9.85	1.07	0.29143

AC006033.2	-0.14	6.31	-1.07	0.29144
SFT2D3	-0.07	7.17	-1.07	0.29150
AC073548.1	-0.16	5.85	-1.07	0.29161
PTENP1	-0.16	6.45	-1.07	0.29161
STAT3	-0.12	12.13	-1.07	0.29175
CEROX1	0.22	7.18	1.07	0.29178
RPL41P2	0.12	7.19	1.06	0.29188
USP7	-0.05	10.39	-1.06	0.29223
AL627309.5	-0.19	5.37	-1.06	0.29232
PHF13	0.08	8.07	1.06	0.29240
KIFAP3	-0.07	7.26	-1.06	0.29249
ZNF317	-0.06	9.31	-1.06	0.29255
CEP83	-0.11	5.60	-1.06	0.29262
PRMT1P1	0.11	5.11	1.06	0.29265
APEX2	0.08	9.02	1.06	0.29266
LRRN3	0.24	6.61	1.06	0.29275
IGHJ6	0.21	7.50	1.06	0.29277
TMPRSS3	0.13	5.34	1.06	0.29278
PHTF2	-0.11	6.82	-1.06	0.29293
ARL6IP5	-0.08	10.76	-1.06	0.29298
TFAM	-0.08	7.58	-1.06	0.29304
CHD7	0.15	9.40	1.06	0.29326
RN7SL368P	0.17	5.53	1.06	0.29326
RAPGEF1	-0.07	11.35	-1.06	0.29336
CIRBP	0.09	11.69	1.06	0.29336
NPIPB5	-0.16	11.77	-1.06	0.29344
AC007877.1	-0.23	6.25	-1.06	0.29361
VPS13A	-0.10	6.86	-1.06	0.29367
STT3A	-0.07	9.49	-1.06	0.29371
TRPC4AP	-0.08	10.97	-1.06	0.29383
BRPF1	-0.06	9.53	-1.06	0.29388
BACE1-AS	-0.11	6.47	-1.06	0.29402
PTPRF	0.16	5.79	1.06	0.29410
PLK3	0.12	8.78	1.06	0.29425
CLECL1	-0.15	5.18	-1.06	0.29432
NDUFA9	0.11	7.80	1.06	0.29437
GLULP4	-0.15	5.46	-1.06	0.29448
IGLV3-27	0.25	5.26	1.06	0.29450
OSTF1P1	-0.14	5.73	-1.06	0.29451
TMEM59	-0.08	10.38	-1.06	0.29455
TOMM20P2	0.14	5.75	1.06	0.29458
EIF1AXP1	-0.13	5.44	-1.06	0.29463
MEOX1	0.15	6.16	1.06	0.29466
AL583856.1	-0.11	5.03	-1.06	0.29467
GPR27	-0.20	7.81	-1.06	0.29468
EIF4EBP2	-0.07	11.17	-1.06	0.29468
TMEM104	0.08	9.02	1.06	0.29471
SLC7A1	-0.09	8.03	-1.06	0.29479
RHEX	-0.14	5.25	-1.06	0.29479
NUGGC	0.14	6.44	1.06	0.29479

AL049840.6	0.10	7.23	1.06	0.29480
NOMO1	0.10	9.39	1.06	0.29485
MAP1LC3A	0.14	7.54	1.06	0.29514
SERTAD2	-0.10	8.99	-1.06	0.29518
GORASP1	-0.09	8.96	-1.06	0.29522
FAM174A	-0.12	6.44	-1.06	0.29531
DDX23	0.05	11.00	1.06	0.29532
SPRY3	-0.11	5.42	-1.06	0.29544
TNFAIP1	-0.07	8.11	-1.06	0.29565
MFSD5	0.10	9.53	1.06	0.29567
PLA2G12A	0.11	7.00	1.06	0.29570
C5AR1	-0.16	12.74	-1.06	0.29581
MRPL19	-0.11	6.72	-1.06	0.29592
LY6G5C	0.10	6.95	1.06	0.29593
PEX11B	0.06	8.45	1.06	0.29599
LENG9	0.11	6.08	1.06	0.29600
GLI1	0.12	4.90	1.06	0.29610
DYNLT3	-0.10	6.69	-1.06	0.29611
EIF3FP3	0.14	8.59	1.06	0.29611
CBWD3	-0.14	6.06	-1.06	0.29612
ERVK13-1	-0.13	6.46	-1.06	0.29617
PRKAG2-AS1	0.10	6.17	1.06	0.29619
HLA-A	0.13	15.69	1.06	0.29632
ADAMTS7P1	0.15	5.25	1.05	0.29638
PLA2G7	-0.16	6.38	-1.05	0.29641
PPIAP6	0.09	5.13	1.05	0.29642
ABHD5	-0.16	9.32	-1.05	0.29644
TAPT1-AS1	-0.11	5.44	-1.05	0.29657
ITGA7	-0.13	5.44	-1.05	0.29661
BZW2	0.08	7.97	1.05	0.29677
PLD2	0.10	8.55	1.05	0.29681
AL392172.1	0.10	5.20	1.05	0.29681
AC097658.2	0.10	6.52	1.05	0.29692
IGKV3-20	0.22	9.26	1.05	0.29695
CLDN15	0.12	8.27	1.05	0.29713
MANSC1	-0.22	8.38	-1.05	0.29721
LINC00665	-0.12	6.13	-1.05	0.29732
ARHGEF10L	-0.13	8.73	-1.05	0.29733
SCAMP1	-0.09	6.78	-1.05	0.29733
AC127502.2	-0.19	6.35	-1.05	0.29737
AC112191.2	0.13	6.24	1.05	0.29746
NONO	-0.05	11.43	-1.05	0.29750
ZNF7	-0.07	7.64	-1.05	0.29757
EEF1A1P10	0.09	5.60	1.05	0.29763
KLHL8	0.12	7.91	1.05	0.29768
ACSL5	-0.08	9.41	-1.05	0.29769
GEN1	-0.12	5.96	-1.05	0.29772
ZSCAN16-AS1	0.11	6.64	1.05	0.29773
OLIG1	0.15	7.98	1.05	0.29779
ITLN1	0.21	5.85	1.05	0.29783

CHCHD4	0.08	7.26	1.05	0.29788
CSTB	0.11	10.59	1.05	0.29794
TMEM135	-0.11	6.20	-1.05	0.29816
AC024075.2	0.11	7.27	1.05	0.29820
CALM2	-0.09	11.32	-1.05	0.29854
SMPD2	0.10	7.63	1.05	0.29855
HBA2	0.29	13.53	1.05	0.29865
FARS2	0.11	7.91	1.05	0.29881
APTR	-0.11	6.20	-1.05	0.29915
ADAMTSL4-AS2	-0.19	6.80	-1.05	0.29931
TPM3	-0.06	13.04	-1.05	0.29947
AHSP	0.32	8.65	1.05	0.29951
RIOX1	0.08	8.01	1.05	0.29970
OTUD6B-AS1	-0.08	7.10	-1.05	0.29972
UBE2G2	0.06	10.29	1.05	0.29986
PTPN2	-0.07	8.63	-1.05	0.30004
ASB3	-0.08	7.13	-1.05	0.30007
MAGOH	0.07	8.26	1.05	0.30007
AL050341.2	-0.11	5.72	-1.05	0.30015
RAC2	0.09	14.05	1.05	0.30032
C9orf64	-0.09	6.70	-1.05	0.30050
WFIKKN1	0.14	5.07	1.05	0.30051
SLC23A2	-0.11	8.58	-1.05	0.30052
FAM238A	-0.13	5.34	-1.05	0.30053
RPL4P5	0.14	8.13	1.05	0.30055
CCDC28A	-0.08	9.00	-1.05	0.30056
GPATCH1	0.06	7.79	1.05	0.30066
AC006970.1	-0.15	5.62	-1.05	0.30069
WASHC3	0.08	7.54	1.05	0.30075
DEK	-0.09	9.21	-1.05	0.30075
TFEC	-0.12	6.65	-1.05	0.30077
LAPTM5	0.09	14.67	1.04	0.30090
ZNF512B	0.10	7.95	1.04	0.30091
C11orf71	0.08	6.29	1.04	0.30094
ASPH	-0.22	7.18	-1.04	0.30101
RPS15AP24	0.15	6.85	1.04	0.30110
AC005332.5	0.12	7.15	1.04	0.30110
KIAA0100	-0.06	11.07	-1.04	0.30118
TTC22	0.09	7.59	1.04	0.30118
ENG	0.15	9.44	1.04	0.30146
H4C8	-0.16	6.54	-1.04	0.30149
UGDH	-0.10	6.67	-1.04	0.30153
SNW1	-0.06	9.70	-1.04	0.30154
AL162274.2	-0.11	5.35	-1.04	0.30156
EBAG9	-0.08	7.47	-1.04	0.30163
FAHD2CP	0.10	6.10	1.04	0.30168
UBA5	-0.08	6.80	-1.04	0.30178
TRIM13	0.09	7.73	1.04	0.30198
TACC3	0.10	11.45	1.04	0.30207
ADGRE1	-0.12	9.36	-1.04	0.30209

DBNDD1	0.18	6.69	1.04	0.30212
USP35	-0.11	6.99	-1.04	0.30243
CDK14	-0.16	6.80	-1.04	0.30247
AP2A1	0.08	11.48	1.04	0.30300
TRAF3IP1	-0.10	6.35	-1.04	0.30308
TMBIM4	0.12	8.18	1.04	0.30311
HDC	-0.20	7.76	-1.04	0.30313
ODR4	-0.10	6.82	-1.04	0.30322
AL049636.1	-0.11	5.01	-1.04	0.30324
CLCN7	0.10	10.53	1.04	0.30332
TRGV2	-0.17	5.30	-1.04	0.30363
SCAI	0.09	6.10	1.04	0.30364
PSEN2	-0.10	7.04	-1.04	0.30367
BTD	0.07	8.00	1.04	0.30375
ZNF154	-0.16	5.66	-1.04	0.30391
MCM6	-0.09	8.37	-1.04	0.30415
ZNF75A	0.10	7.72	1.04	0.30417
DDX20	-0.09	7.29	-1.04	0.30429
WIPI2	0.06	10.97	1.04	0.30430
P2RX1	-0.17	10.13	-1.04	0.30434
CD84	0.12	9.47	1.04	0.30451
ALDH3A2	-0.08	8.38	-1.04	0.30465
C8orf31	0.14	5.04	1.04	0.30466
TRIM39	0.07	7.96	1.04	0.30486
CRYL1	0.10	8.24	1.04	0.30487
RIN1	0.16	7.45	1.04	0.30490
CYB561D2	0.08	8.55	1.04	0.30511
SELENBP1	0.33	10.43	1.04	0.30511
TP53I13	0.12	8.32	1.04	0.30528
PBDC1	0.09	7.71	1.04	0.30535
CLPB	0.09	8.20	1.04	0.30536
AC125238.2	-0.09	5.14	-1.04	0.30538
DLL1	-0.14	5.52	-1.04	0.30538
AL117379.1	-0.12	5.28	-1.03	0.30551
SYPL1	-0.10	8.30	-1.03	0.30554
SLC20A2	-0.08	7.45	-1.03	0.30568
RAB11B-AS1	0.11	5.78	1.03	0.30577
CAPN7	-0.11	6.75	-1.03	0.30593
COQ3	0.09	5.69	1.03	0.30599
ADCK5	0.11	7.06	1.03	0.30608
RAB6B	0.16	6.40	1.03	0.30609
TAFA1	0.13	5.51	1.03	0.30612
RPS3AP25	0.11	5.50	1.03	0.30623
ANAPC7	0.06	8.95	1.03	0.30627
HBM	0.35	10.21	1.03	0.30628
NKG7	0.19	12.99	1.03	0.30629
RRP8	0.07	8.54	1.03	0.30636
DLAT	-0.08	6.92	-1.03	0.30646
RWDD3	-0.12	5.15	-1.03	0.30648
AC145098.2	-0.16	5.77	-1.03	0.30650

BDP1	-0.11	6.79	-1.03	0.30650
PPP6R3	-0.06	9.32	-1.03	0.30653
FLT3	-0.18	6.28	-1.03	0.30666
LDAH	0.08	6.93	1.03	0.30675
AC138207.4	-0.12	5.06	-1.03	0.30687
G2E3	-0.09	6.30	-1.03	0.30688
AL138824.1	-0.14	5.21	-1.03	0.30692
NRBP1	0.06	11.10	1.03	0.30697
INO80E	0.10	9.66	1.03	0.30704
RNF135	-0.09	8.99	-1.03	0.30709
NUP188	0.07	9.85	1.03	0.30713
C3	-0.17	6.52	-1.03	0.30730
USP10	-0.13	10.89	-1.03	0.30732
S100A6	0.12	12.38	1.03	0.30736
UPRT	-0.09	6.44	-1.03	0.30738
AC074132.1	-0.14	5.12	-1.03	0.30743
CSTF3	-0.08	7.28	-1.03	0.30750
NDST1	-0.14	10.14	-1.03	0.30764
IFNG	-0.14	5.14	-1.03	0.30776
ZNF283	0.10	5.84	1.03	0.30777
AL109618.1	-0.13	5.27	-1.03	0.30785
BTBD11	0.09	7.77	1.03	0.30791
TUFT1	-0.10	6.03	-1.03	0.30801
RSAD1	0.08	9.02	1.03	0.30807
RPS3AP6	0.19	9.02	1.03	0.30815
PDXK	0.10	10.96	1.03	0.30818
DTNBP1	0.09	8.56	1.03	0.30822
CACNA1H	0.15	6.00	1.03	0.30825
LMNB2	0.09	8.49	1.03	0.30835
JPH4	-0.10	4.93	-1.03	0.30842
ATAT1	0.10	6.47	1.03	0.30862
TMEM60	0.08	7.76	1.03	0.30867
TNFRSF25	0.15	9.42	1.03	0.30871
KANSL1L	-0.13	5.76	-1.03	0.30875
CBX5	0.09	8.81	1.03	0.30886
PDCD10	-0.09	8.17	-1.03	0.30887
TRDV1	-0.22	5.61	-1.03	0.30907
RBBP6	0.09	9.55	1.03	0.30911
ARID5A	0.11	10.12	1.03	0.30918
IGLV2-18	0.26	5.49	1.03	0.30923
PIGT	0.07	10.37	1.03	0.30925
SYMPK	0.07	10.61	1.03	0.30927
ABCA2	-0.11	10.82	-1.03	0.30932
CAP1P2	-0.15	6.98	-1.03	0.30942
CDH26	-0.14	5.61	-1.03	0.30946
PLEKHA4	0.10	5.63	1.03	0.30979
LMNA	0.14	9.27	1.03	0.30989
CHRNB1	0.09	7.69	1.03	0.30993
AC010168.2	-0.12	5.21	-1.03	0.30994
AC099336.1	0.24	8.87	1.03	0.31001

EXOSC3	0.09	6.68	1.02	0.31016
AL445363.3	0.10	5.37	1.02	0.31020
AC092746.1	-0.18	5.05	-1.02	0.31030
A1BG-AS1	0.09	7.19	1.02	0.31037
AC090409.1	0.14	5.88	1.02	0.31051
PSMD6	-0.07	8.85	-1.02	0.31059
GIGYF1	0.09	10.70	1.02	0.31066
TSPY26P	0.11	5.66	1.02	0.31067
EMILIN2	-0.11	11.12	-1.02	0.31097
PIGC	0.10	8.49	1.02	0.31104
CYCS	-0.06	8.29	-1.02	0.31109
ANKRD36B	0.13	5.40	1.02	0.31122
RAB36	-0.19	6.34	-1.02	0.31144
FAM160A2	-0.11	9.57	-1.02	0.31144
OSBPL2	-0.10	10.33	-1.02	0.31149
ZMAT1	-0.14	6.59	-1.02	0.31149
CHRNA2	-0.19	5.44	-1.02	0.31155
MSR1	-0.18	5.29	-1.02	0.31156
RXRB	0.06	10.29	1.02	0.31158
ATP5F1BP1	-0.10	5.07	-1.02	0.31162
SLC30A7	-0.10	7.56	-1.02	0.31174
ZBTB10	-0.11	5.90	-1.02	0.31180
RGS2	-0.19	13.15	-1.02	0.31188
ACACB	0.08	8.14	1.02	0.31194
DNAJC11	0.07	9.24	1.02	0.31197
DDX3Y	-0.48	7.56	-1.02	0.31212
HK3	-0.13	11.62	-1.02	0.31216
QSOX1	0.11	10.77	1.02	0.31231
ZMYM1	-0.11	5.70	-1.02	0.31240
FRG1HP	0.09	6.59	1.02	0.31242
GSTM4	0.18	7.62	1.02	0.31257
TCEAL8	0.10	7.54	1.02	0.31262
SMIM14	-0.07	8.66	-1.02	0.31278
RNASE2	-0.17	8.50	-1.02	0.31281
AC008969.1	-0.13	5.94	-1.02	0.31290
MOV10	-0.14	9.99	-1.02	0.31315
GGA3	-0.07	10.45	-1.02	0.31316
DGLUCY	-0.10	10.90	-1.02	0.31317
FZD2	-0.13	7.63	-1.02	0.31320
SLC25A45	0.11	9.02	1.02	0.31325
IGF1R	-0.15	9.63	-1.02	0.31328
PROCA1	0.12	7.29	1.02	0.31335
TMED4	-0.05	9.65	-1.02	0.31338
ZNF431	-0.08	7.60	-1.02	0.31347
TNFRSF9	-0.13	6.67	-1.02	0.31353
SLC6A16	0.10	6.09	1.02	0.31358
CHMP2A	0.11	11.49	1.02	0.31361
CEP63	-0.08	9.07	-1.02	0.31363
AC106795.1	0.12	6.91	1.02	0.31363
AFG1L	-0.10	5.16	-1.02	0.31363

ZRANB1	-0.10	8.47	-1.02	0.31370
TLN2	-0.10	5.29	-1.02	0.31380
TNRC6B	-0.09	9.63	-1.02	0.31391
MED15	0.06	11.47	1.02	0.31399
AOC3	-0.17	7.08	-1.02	0.31401
TFB1M	-0.09	6.81	-1.02	0.31402
IFI30	-0.15	5.77	-1.02	0.31410
ISL2	-0.16	6.86	-1.02	0.31428
SBF2-AS1	0.09	5.22	1.02	0.31440
MGST1	-0.14	6.90	-1.02	0.31470
COL7A1	-0.13	5.43	-1.02	0.31470
SARDH	0.13	6.39	1.01	0.31505
SCRN1	-0.11	7.98	-1.01	0.31505
AC244669.1	-0.11	5.83	-1.01	0.31508
CXCR4	0.08	12.11	1.01	0.31537
AC103691.1	0.14	6.67	1.01	0.31542
FASN	0.10	9.45	1.01	0.31548
HDLBP	0.06	11.99	1.01	0.31550
STOML1	0.08	7.24	1.01	0.31555
ZFP28	-0.09	5.60	-1.01	0.31569
BTG1P1	-0.12	5.30	-1.01	0.31570
PDCD7	0.06	8.41	1.01	0.31576
AL117335.1	-0.13	5.74	-1.01	0.31590
MOB3B	-0.09	5.83	-1.01	0.31598
S1PR2	0.11	6.68	1.01	0.31600
SNAI3-AS1	0.11	6.46	1.01	0.31614
CCT4	0.08	10.41	1.01	0.31621
P3H4	0.09	6.50	1.01	0.31629
ENTPD5	-0.11	6.75	-1.01	0.31652
NDUFS2	0.05	10.23	1.01	0.31655
COA5	-0.07	7.41	-1.01	0.31660
SIRPD	-0.13	5.42	-1.01	0.31682
SLC6A8	0.28	9.74	1.01	0.31719
PDE7B	-0.12	5.04	-1.01	0.31720
LAMTOR1	0.10	11.15	1.01	0.31723
NT5DC2	0.18	7.00	1.01	0.31734
TIMP1	0.14	11.42	1.01	0.31768
KAT14	0.07	8.20	1.01	0.31772
PRDX6	0.17	11.36	1.01	0.31773
SMIM35	-0.10	5.57	-1.01	0.31773
SUGP2	-0.11	9.95	-1.01	0.31774
USP9Y	-0.37	6.72	-1.01	0.31786
MAGOHB	-0.11	6.30	-1.01	0.31789
EPM2AIP1	-0.11	7.35	-1.01	0.31808
IGHJ4	0.22	8.56	1.01	0.31823
C20orf96	0.11	6.22	1.01	0.31839
KAT2A	0.10	9.31	1.01	0.31850
TECPR2	-0.15	10.42	-1.01	0.31860
ADAMTSL5	0.13	5.53	1.01	0.31861
FAM43A	0.11	8.45	1.01	0.31873

PCMT1	-0.07	9.31	-1.01	0.31886
MFF	0.06	8.90	1.01	0.31891
SMCO4	-0.11	8.22	-1.01	0.31891
PARP1	0.08	10.87	1.01	0.31893
GATAD1	-0.07	7.98	-1.01	0.31898
ZNF324B	0.09	6.39	1.01	0.31900
AEN	0.10	8.37	1.01	0.31901
ARHGAP25	-0.08	12.43	-1.01	0.31908
GMPPB	0.06	8.30	1.01	0.31921
CHTF8	0.09	5.17	1.01	0.31934
AL162231.2	0.10	5.30	1.01	0.31948
RNF125	0.10	8.51	1.01	0.31950
PRRC2B	0.05	11.72	1.00	0.31973
TBC1D4	-0.14	7.57	-1.00	0.31994
TRAF3IP3	0.06	11.76	1.00	0.32001
ZNF202	-0.09	7.41	-1.00	0.32048
AC110749.1	0.11	6.07	1.00	0.32053
EGLN3	0.10	5.91	1.00	0.32059
AC092279.3	0.16	5.55	1.00	0.32060
CSRNP1	-0.15	9.45	-1.00	0.32081
SLC25A12	-0.07	7.79	-1.00	0.32084
SLC25A16	0.09	7.03	1.00	0.32090
COX5B	0.10	10.92	1.00	0.32093
GUCD1	0.09	10.68	1.00	0.32097
AL512625.3	-0.11	5.21	-1.00	0.32103
NGDN	0.07	8.29	1.00	0.32106
XPC	-0.06	10.31	-1.00	0.32118
MED17	-0.07	6.45	-1.00	0.32120
AC007342.2	-0.16	5.77	-1.00	0.32122
GATC	0.08	7.86	1.00	0.32126
CDK7	-0.08	7.02	-1.00	0.32140
VPS35L	0.08	8.92	1.00	0.32146
RAB7A	-0.10	11.96	-1.00	0.32166
SEPTIN4	-0.15	6.47	-1.00	0.32176
CCDC157	0.11	6.24	1.00	0.32192
KRT73-AS1	0.17	5.43	1.00	0.32206
AC026748.1	0.10	5.25	1.00	0.32208
NUDT16	-0.12	9.21	-1.00	0.32209
OLFML2B	-0.10	5.33	-1.00	0.32225
UNC45B	0.13	4.94	1.00	0.32230
CNTLN	0.10	5.74	1.00	0.32241
LINC00824	0.13	5.32	1.00	0.32244
RPL7P6	0.09	5.40	1.00	0.32258
SNX8	0.10	8.59	1.00	0.32263
ZNF205	-0.15	5.27	-1.00	0.32267
DMKN	0.11	5.18	1.00	0.32275
CASP7	-0.10	7.55	-1.00	0.32299
CRY2	0.06	9.07	1.00	0.32310
CREBL2	-0.08	8.77	-1.00	0.32312
CBR3	0.14	6.57	1.00	0.32313

CLNS1A	0.07	8.91	1.00	0.32322
AP001636.3	-0.11	5.23	-1.00	0.32325
MAN2B1	0.08	11.79	1.00	0.32331
SF3A3	0.07	9.86	1.00	0.32340
MAST4	0.12	7.19	1.00	0.32366
CNNM3	-0.06	9.42	-1.00	0.32381
SIGLEC9	-0.11	10.01	-1.00	0.32383
GCNT4	-0.14	6.01	-1.00	0.32392
MTERF3	-0.10	6.39	-1.00	0.32394
PHIP	0.15	9.41	1.00	0.32417
SLC24A1	-0.10	5.92	-1.00	0.32418
WDR3	-0.10	7.27	-1.00	0.32424
HMGB2P1	-0.12	5.06	-1.00	0.32425
FAM120B	-0.05	9.02	-1.00	0.32431
HLA-J	0.30	8.93	0.99	0.32448
DGKZP1	0.12	8.13	0.99	0.32450
AL356000.1	-0.11	5.64	-0.99	0.32457
ZNF780A	-0.06	7.41	-0.99	0.32468
ALS2CL	0.17	7.80	0.99	0.32475
ZNF652	-0.06	9.93	-0.99	0.32475
GPSM1	0.10	6.12	0.99	0.32477
FIGNL2	0.10	5.20	0.99	0.32480
C17orf80	-0.07	7.29	-0.99	0.32486
ACE	0.12	6.01	0.99	0.32488
SETD4	-0.08	7.65	-0.99	0.32489
AL031274.1	-0.11	5.29	-0.99	0.32500
NFIX	0.24	9.21	0.99	0.32502
FTH1P22	-0.13	5.59	-0.99	0.32513
MIR4453HG	-0.13	5.26	-0.99	0.32516
ALAS2	0.35	12.88	0.99	0.32520
SIGLEC17P	-0.15	7.33	-0.99	0.32522
AC021683.5	-0.18	5.71	-0.99	0.32531
AL356488.3	0.14	5.33	0.99	0.32539
VPS33B	0.10	6.21	0.99	0.32546
LMO4	-0.08	8.84	-0.99	0.32547
AC104463.2	-0.13	5.25	-0.99	0.32555
PSMG4	0.09	7.22	0.99	0.32567
SLC35E2B	-0.12	9.80	-0.99	0.32571
ZNF271P	0.09	8.23	0.99	0.32580
ZNF506	-0.08	8.20	-0.99	0.32584
VEPH1	0.12	5.08	0.99	0.32585
ZNF420	0.10	6.46	0.99	0.32590
SFXN4	0.11	7.04	0.99	0.32596
TCEA1	-0.09	7.82	-0.99	0.32608
FAM95C	-0.12	5.31	-0.99	0.32662
SERPINF1	0.14	6.80	0.99	0.32668
PRSS53	-0.11	5.54	-0.99	0.32690
CDYL	0.09	7.97	0.99	0.32706
ZNF516	-0.12	9.71	-0.99	0.32727
BRICD5	0.12	7.06	0.99	0.32728

REPIN1	0.07	9.89	0.99	0.32730
ADD2	0.11	6.69	0.99	0.32737
EEF1A1P14	0.11	6.52	0.99	0.32741
ESYT1	0.07	11.98	0.99	0.32744
BUD31	0.10	10.19	0.99	0.32744
DARS2	-0.09	6.98	-0.99	0.32758
TMEM208	0.11	8.80	0.99	0.32776
CALM2P2	-0.13	6.29	-0.99	0.32777
PI4KA	-0.06	10.90	-0.99	0.32809
PDCL	-0.08	7.35	-0.99	0.32816
RNY1	0.20	5.23	0.99	0.32817
CCP110	-0.09	7.14	-0.99	0.32818
AKR1A1	0.09	9.76	0.99	0.32821
WASF1	-0.12	5.07	-0.99	0.32829
NLN	-0.09	6.79	-0.99	0.32833
CSDE1	-0.06	12.00	-0.99	0.32833
PI4KB	0.05	10.96	0.99	0.32837
LINC00894	-0.14	6.10	-0.99	0.32843
SLC25A39	0.27	14.62	0.99	0.32848
CNIH3	0.14	5.96	0.99	0.32848
STK17B	-0.09	10.23	-0.99	0.32848
KAT2B	-0.15	8.49	-0.99	0.32869
AC240565.4	-0.21	7.46	-0.99	0.32878
HSPH1	-0.08	8.66	-0.99	0.32890
DLG1	0.11	7.70	0.99	0.32891
TERF2	0.06	8.75	0.99	0.32906
AL592295.5	0.14	5.46	0.99	0.32910
ITGB3	0.22	8.71	0.99	0.32910
MAPK9	-0.06	8.09	-0.99	0.32916
ACADSB	-0.09	6.92	-0.98	0.32921
VPS45	-0.06	8.32	-0.98	0.32922
NDUFA12	0.10	8.90	0.98	0.32935
COL6A2	-0.17	9.55	-0.98	0.32955
PRAM1	0.12	11.52	0.98	0.32958
DTX4	0.10	7.53	0.98	0.32968
HNRNPA3	-0.06	10.27	-0.98	0.32973
HRK	-0.15	5.20	-0.98	0.32975
AC022968.1	0.11	5.41	0.98	0.33001
RFK	-0.10	6.62	-0.98	0.33021
AC005670.3	-0.10	6.85	-0.98	0.33036
ARHGAP27P1-BPTFP1-KPNA2P3	-0.12	7.10	-0.98	0.33037
HSPA1L	0.08	7.29	0.98	0.33042
FDXACB1	-0.09	5.48	-0.98	0.33044
AL365475.1	0.16	5.97	0.98	0.33045
AC231533.2	-0.10	5.71	-0.98	0.33046
UPK3A	0.17	6.16	0.98	0.33047
TBRG4	0.07	9.41	0.98	0.33051
ARHGEF4	-0.10	5.63	-0.98	0.33068
POLR2J2	0.14	5.22	0.98	0.33069
ANKRD22	-0.23	6.32	-0.98	0.33071

AL844908.1	-0.11	6.04	-0.98	0.33073
MRPL49	0.06	9.25	0.98	0.33079
PFDN1	0.08	8.54	0.98	0.33082
FBXL20	-0.08	8.82	-0.98	0.33082
ACTB	0.10	17.44	0.98	0.33098
AC008763.1	-0.12	5.67	-0.98	0.33108
PSTPIP1	0.09	11.75	0.98	0.33122
FAM86FP	-0.12	5.12	-0.98	0.33123
SELPLG	0.11	14.29	0.98	0.33130
EXD2	-0.09	6.44	-0.98	0.33188
MAP3K10	0.10	7.57	0.98	0.33189
ARHGEF1	0.08	13.53	0.98	0.33205
SUPT4H1P2	-0.11	5.32	-0.98	0.33207
ZNRD1ASP	-0.11	6.05	-0.98	0.33208
VAMP1	-0.13	8.94	-0.98	0.33223
RPL39	0.14	9.14	0.98	0.33223
ZNF514	-0.11	6.85	-0.98	0.33229
OASL	-0.25	9.38	-0.98	0.33232
CYB561	0.09	8.82	0.98	0.33249
CCDC43	-0.07	6.75	-0.98	0.33256
TMEM161B-AS1	-0.10	6.90	-0.98	0.33282
LRRC8C	-0.07	8.72	-0.98	0.33291
GGH	-0.10	5.21	-0.98	0.33296
AC107464.3	0.10	5.12	0.98	0.33304
NUDT2	0.12	7.78	0.98	0.33324
NUDT13	0.12	5.38	0.98	0.33326
C2CD2	-0.10	6.73	-0.98	0.33331
BAG6	0.09	12.57	0.98	0.33331
NCK2	0.08	9.84	0.98	0.33341
PLIN3	0.10	10.94	0.98	0.33350
LINC01962	0.13	4.95	0.98	0.33358
AC022167.3	0.17	6.20	0.98	0.33361
INO80D	-0.08	7.90	-0.98	0.33377
AL117336.2	-0.12	4.97	-0.98	0.33384
IGHV3-20	-0.28	6.16	-0.98	0.33386
EML2-AS1	0.15	5.51	0.98	0.33392
LINC02580	0.14	5.79	0.98	0.33401
LAX1	0.11	7.83	0.98	0.33401
ZKSCAN8	-0.08	7.32	-0.97	0.33442
IL18RAP	-0.26	9.63	-0.97	0.33443
P2RY12	-0.12	5.59	-0.97	0.33452
TVP23B	-0.10	6.65	-0.97	0.33454
RPSAP47	0.12	6.22	0.97	0.33461
AC009113.1	-0.08	5.50	-0.97	0.33465
PLXNB2	-0.13	11.91	-0.97	0.33469
NBPF9	-0.12	6.65	-0.97	0.33473
RPL5P5	0.10	5.35	0.97	0.33473
DDTL	0.12	5.45	0.97	0.33477
AC110285.2	-0.14	5.59	-0.97	0.33485
ALG14	0.10	5.74	0.97	0.33486

PRPF38B	0.08	9.91	0.97	0.33496
FBXO4	-0.08	6.27	-0.97	0.33506
IGKV3-11	0.19	8.43	0.97	0.33524
STK11IP	0.08	9.69	0.97	0.33535
GAK	0.06	11.26	0.97	0.33541
BCL2A1	-0.19	9.21	-0.97	0.33543
RHNO1	0.07	7.70	0.97	0.33545
PPM1L	-0.10	6.82	-0.97	0.33564
GRINA	0.15	12.39	0.97	0.33578
TRAM2-AS1	-0.10	5.29	-0.97	0.33589
ZBTB5	-0.08	7.43	-0.97	0.33591
YBX3	0.24	12.48	0.97	0.33595
MBNL2	-0.08	6.93	-0.97	0.33602
GATAD2B	-0.06	9.86	-0.97	0.33604
RGL2	-0.13	11.43	-0.97	0.33605
ANKRD46	-0.09	6.16	-0.97	0.33617
MIAT	-0.19	8.61	-0.97	0.33619
QSOX2	-0.11	8.03	-0.97	0.33633
RPS6KL1	0.14	5.43	0.97	0.33635
ATP8A2	0.11	5.45	0.97	0.33639
H3P16	-0.15	8.67	-0.97	0.33641
ZNF429	-0.17	7.50	-0.97	0.33653
MYOM1	-0.11	6.31	-0.97	0.33661
ANKRD10-IT1	0.12	5.67	0.97	0.33661
CNTNAP3	-0.22	6.76	-0.97	0.33665
CC2D1B	0.06	9.72	0.97	0.33669
GLUL	-0.13	12.69	-0.97	0.33669
IMPDH1P6	-0.11	5.27	-0.97	0.33674
PTGDR	-0.12	6.90	-0.97	0.33682
GTF2B	-0.07	8.66	-0.97	0.33713
GYG1	-0.14	9.19	-0.97	0.33722
LTK	0.17	7.30	0.97	0.33724
ZNF629	0.09	7.43	0.97	0.33726
SS18L1	-0.10	6.85	-0.97	0.33728
HPGD	-0.11	5.61	-0.97	0.33746
U73166.1	0.17	5.10	0.97	0.33770
PCBD1	0.10	7.31	0.97	0.33771
AC068522.1	0.15	5.27	0.97	0.33784
CEP104	-0.06	8.33	-0.97	0.33787
GOLPH3L	-0.08	7.18	-0.97	0.33790
ANKRD37	-0.11	5.69	-0.97	0.33795
AC104651.1	-0.13	5.49	-0.97	0.33800
CHAF1A	0.08	8.07	0.97	0.33805
OBSCN	0.15	8.97	0.97	0.33809
KIFC2	0.14	8.29	0.97	0.33812
VWA7	0.12	5.66	0.97	0.33819
TPTEP1	0.16	6.56	0.97	0.33836
ADSS1	-0.12	5.50	-0.97	0.33844
RBM4B	0.06	7.89	0.97	0.33852
AL157895.2	-0.12	5.13	-0.97	0.33853

EID2B	0.13	5.44	0.97	0.33858
SH2B1	0.10	9.95	0.97	0.33860
MFSD9	-0.12	7.31	-0.97	0.33863
TMEM248	0.05	10.49	0.97	0.33869
MAP3K20	-0.10	7.58	-0.97	0.33870
PPM1A	-0.12	9.81	-0.97	0.33876
GET1	0.11	6.01	0.97	0.33877
MMAA	0.11	6.30	0.97	0.33888
GNB1	-0.06	12.51	-0.97	0.33892
RPP30	-0.10	7.67	-0.97	0.33895
CCDC28B	0.12	7.23	0.97	0.33903
H3P14	-0.08	5.09	-0.96	0.33904
ITGA6	-0.13	8.16	-0.96	0.33909
DCST2	-0.14	5.31	-0.96	0.33917
LINC02289	-0.21	7.20	-0.96	0.33920
DIRAS1	0.15	5.41	0.96	0.33921
HOXB-AS1	0.16	5.73	0.96	0.33936
IGLV1-36	0.20	5.40	0.96	0.33938
SUPT16H	-0.05	9.89	-0.96	0.33947
TRIM44	0.08	9.84	0.96	0.33951
ARPIN	-0.18	6.84	-0.96	0.33957
AC005747.1	-0.15	11.10	-0.96	0.33975
RBM14	0.05	9.88	0.96	0.34003
TFRC	-0.13	7.37	-0.96	0.34004
ZNF668	0.11	7.67	0.96	0.34009
AL391834.2	-0.10	5.10	-0.96	0.34013
RPS4XP13	0.14	6.87	0.96	0.34025
JAGN1	0.09	8.19	0.96	0.34027
KCNG1	0.19	5.93	0.96	0.34034
IGHV4-61	0.21	7.02	0.96	0.34041
Z95327.2	-0.15	5.27	-0.96	0.34047
PRRT1	0.13	5.40	0.96	0.34073
SLC30A9	-0.07	7.48	-0.96	0.34080
GON4L	-0.06	9.67	-0.96	0.34088
AC009318.1	0.11	5.86	0.96	0.34110
GATA1	0.20	8.71	0.96	0.34125
EXOC7	0.05	11.12	0.96	0.34132
BSDC1	-0.05	9.86	-0.96	0.34140
NUDT3	0.06	10.00	0.96	0.34152
LIPT2	0.10	5.42	0.96	0.34177
AC113935.1	0.15	8.92	0.96	0.34191
SUGT1	-0.08	7.84	-0.96	0.34192
RBBP9	-0.09	6.00	-0.96	0.34197
RIOK3	-0.14	9.33	-0.96	0.34200
TMEM209	-0.10	6.63	-0.96	0.34203
MRPL46	-0.10	5.19	-0.96	0.34203
ZKSCAN2	0.07	6.36	0.96	0.34213
ACAD8	-0.06	8.41	-0.96	0.34213
CPSF3	0.06	8.39	0.96	0.34225
RNU7-40P	0.14	4.99	0.96	0.34246

ATG4B	0.07	10.36	0.96	0.34250
NR2F6	0.12	5.93	0.96	0.34250
AC079336.5	0.14	5.12	0.96	0.34251
TCEANC	-0.11	6.62	-0.96	0.34254
CD247	0.10	11.92	0.96	0.34257
ZNF528-AS1	0.11	5.63	0.96	0.34260
OLMALINC	0.09	5.46	0.96	0.34262
DPY19L4	-0.11	5.61	-0.96	0.34265
IDH3B	0.07	10.26	0.96	0.34274
VHL	0.08	9.08	0.96	0.34274
SH3GL1P1	0.11	5.22	0.96	0.34277
TRGV3	-0.16	5.55	-0.96	0.34284
PGDP1	-0.15	7.02	-0.96	0.34287
SCPEP1	-0.09	10.36	-0.96	0.34342
AF274858.1	0.08	5.07	0.96	0.34342
B4GALT1	-0.08	10.81	-0.96	0.34342
C5orf30	0.11	5.88	0.96	0.34343
AL008721.2	-0.09	5.38	-0.96	0.34343
TPST1	-0.22	6.76	-0.96	0.34345
EXOC3L1	-0.19	5.24	-0.96	0.34348
EBF4	0.13	6.03	0.96	0.34361
NEIL1	0.14	7.22	0.96	0.34363
TBC1D15	-0.09	8.08	-0.96	0.34363
HSPA5	-0.07	11.92	-0.96	0.34365
FASTKD5	-0.06	8.09	-0.96	0.34374
DPH3	-0.09	7.98	-0.96	0.34380
TLCD2	0.11	5.07	0.96	0.34390
TBCCD1	-0.08	6.37	-0.95	0.34414
AQP10	0.20	6.44	0.95	0.34416
IGSF9B	0.14	7.15	0.95	0.34424
CISD2	0.10	7.36	0.95	0.34428
UBE2E2	-0.09	7.19	-0.95	0.34428
AC107884.1	-0.12	5.65	-0.95	0.34432
HEY1	-0.14	5.18	-0.95	0.34433
UBE2D3-AS1	-0.08	5.19	-0.95	0.34443
SCD	-0.12	6.37	-0.95	0.34447
TRBV4-1	0.20	6.55	0.95	0.34447
CCDC102A	0.11	7.26	0.95	0.34513
TRPM6	-0.17	6.92	-0.95	0.34521
ZNF615	-0.10	6.06	-0.95	0.34527
AC131212.3	0.10	5.21	0.95	0.34550
TRBV27	0.15	6.57	0.95	0.34555
ZNF562	0.07	7.77	0.95	0.34557
CBX4	0.08	8.74	0.95	0.34560
IDH3A	-0.08	8.51	-0.95	0.34562
DCAF15	0.06	10.07	0.95	0.34562
NUDCD2	0.09	7.05	0.95	0.34564
AP001992.1	-0.13	6.07	-0.95	0.34575
SVBP	0.11	7.84	0.95	0.34576
AC010468.1	0.15	6.75	0.95	0.34577

AC024060.2	-0.11	5.27	-0.95	0.34582
ITGA5	-0.09	11.81	-0.95	0.34584
PPP2R5A	-0.09	9.64	-0.95	0.34602
RANBP10	0.13	9.52	0.95	0.34612
CLPTM1L	0.06	10.62	0.95	0.34614
AC104083.1	-0.10	5.05	-0.95	0.34615
RAD17	-0.07	7.28	-0.95	0.34622
MDK	-0.13	5.47	-0.95	0.34634
KMO	-0.13	6.46	-0.95	0.34635
SLC2A3	-0.15	11.43	-0.95	0.34637
FCGR1A	-0.24	8.47	-0.95	0.34650
NSUN3	-0.12	7.44	-0.95	0.34659
FAM98A	-0.08	6.83	-0.95	0.34668
AC078991.1	0.10	5.43	0.95	0.34676
CCDC66	0.08	7.22	0.95	0.34678
TGFBI	-0.12	11.66	-0.95	0.34687
SLC5A9	-0.18	5.42	-0.95	0.34702
MTG1	0.11	7.06	0.95	0.34708
ACTR1A	0.06	10.98	0.95	0.34717
GPSM2	-0.10	5.83	-0.95	0.34764
KIAA1522	-0.11	6.21	-0.95	0.34771
RGS16	0.12	5.27	0.95	0.34773
DNAH1	-0.12	9.40	-0.95	0.34775
LRPPRC	-0.07	9.19	-0.95	0.34787
DNAJC27	-0.08	5.79	-0.95	0.34798
CXorf56	0.07	7.80	0.95	0.34800
CCNY	-0.06	10.50	-0.95	0.34808
NUMA1	0.06	12.21	0.95	0.34810
TMEM185A	0.07	7.14	0.95	0.34811
NAAA	-0.12	9.92	-0.95	0.34830
GRAMD4	-0.09	8.92	-0.95	0.34835
KAT8	0.06	10.19	0.95	0.34857
ANKMY2	-0.07	7.05	-0.95	0.34873
VRK1	0.07	7.24	0.95	0.34875
AC005332.6	-0.07	8.09	-0.95	0.34884
AC022306.1	-0.09	5.00	-0.95	0.34891
TMEM263	0.11	6.86	0.95	0.34902
GGTA1P	-0.12	7.07	-0.94	0.34930
AL031777.2	-0.13	5.49	-0.94	0.34933
GCNA	-0.11	6.21	-0.94	0.34935
HNRNPKP2	-0.12	6.56	-0.94	0.34943
CCNE1	0.09	5.53	0.94	0.34946
CAPG	0.12	11.34	0.94	0.34954
CLN8	-0.09	7.74	-0.94	0.34956
EIF5AL1	0.09	7.38	0.94	0.34959
AC016700.2	0.11	5.35	0.94	0.34961
DOCK9	-0.16	7.88	-0.94	0.34962
FANCM	-0.08	5.48	-0.94	0.34965
AC098583.1	0.09	5.28	0.94	0.34965
AC132938.4	0.09	5.29	0.94	0.34995

TMEM150A	0.08	7.78	0.94	0.35000
GEMIN7	0.08	8.23	0.94	0.35012
TMEM191A	0.10	5.82	0.94	0.35020
AC100861.1	0.09	6.25	0.94	0.35020
MYADM	-0.12	12.42	-0.94	0.35023
DYNC1I2P1	-0.09	6.04	-0.94	0.35027
ERI1	-0.09	6.98	-0.94	0.35032
EMC2	-0.09	6.99	-0.94	0.35035
MACO1	-0.09	8.36	-0.94	0.35037
ORMDL2	0.09	8.66	0.94	0.35040
POLR3A	0.06	8.45	0.94	0.35060
SEC14L1P1	-0.12	6.21	-0.94	0.35061
NOD1	-0.07	8.36	-0.94	0.35076
KMT2E	-0.08	10.35	-0.94	0.35079
BTF3P5	0.09	5.50	0.94	0.35088
BLMH	0.07	8.63	0.94	0.35095
SMC4	0.13	8.76	0.94	0.35101
HNRNPC	-0.05	12.23	-0.94	0.35111
SEC14L4	0.14	5.09	0.94	0.35112
RPL5P11	-0.11	5.06	-0.94	0.35113
GIMAP7	0.09	11.18	0.94	0.35116
PRKCB	-0.07	11.80	-0.94	0.35146
ADCY10P1	-0.13	6.67	-0.94	0.35152
SDAD1	-0.07	8.42	-0.94	0.35161
AL445189.2	0.11	5.44	0.94	0.35180
LINC00528	-0.11	7.89	-0.94	0.35180
PCDH12	-0.10	5.62	-0.94	0.35185
PRMT2	0.06	11.26	0.94	0.35194
CPNE2	0.10	9.18	0.94	0.35197
MAN1C1	0.11	8.53	0.94	0.35205
THTPA	0.10	6.26	0.94	0.35222
METTL6	0.07	6.50	0.94	0.35227
LRRC20	0.09	6.55	0.94	0.35242
GBF1	-0.05	10.47	-0.94	0.35261
ARID3B	-0.08	9.28	-0.94	0.35274
ARL11	-0.11	8.69	-0.94	0.35280
BTLA	0.12	6.85	0.94	0.35303
EHHADH	-0.11	5.02	-0.94	0.35305
GTF2F2	-0.07	7.35	-0.94	0.35309
LILRP2	-0.12	5.71	-0.94	0.35316
AC009961.4	-0.14	5.43	-0.94	0.35319
NIT1	-0.06	8.83	-0.94	0.35320
ALKBH5	0.06	10.38	0.94	0.35320
HTRA2	0.06	9.06	0.94	0.35323
CBX3P9	-0.10	5.26	-0.94	0.35329
MLLT11	-0.09	5.89	-0.94	0.35339
IQSEC1	-0.09	12.34	-0.94	0.35379
AC021016.2	-0.10	7.36	-0.94	0.35401
AL031587.5	0.10	6.50	0.94	0.35404
KLHL24	-0.08	8.22	-0.93	0.35431

IGKV3D-11	0.16	5.62	0.93	0.35432
HACD2	-0.08	7.58	-0.93	0.35446
CDK20	0.10	5.79	0.93	0.35463
ZBTB48	0.07	9.52	0.93	0.35465
HLA-DOA	0.13	9.63	0.93	0.35485
PIK3CA	-0.08	7.78	-0.93	0.35498
ZDHHC11	-0.12	5.14	-0.93	0.35511
LINC00996	-0.14	5.23	-0.93	0.35512
KCNMB4	0.11	5.73	0.93	0.35514
CCDC58	0.09	5.70	0.93	0.35516
AC064836.3	0.11	5.06	0.93	0.35530
NARS2	0.08	6.53	0.93	0.35535
RUSC1	0.09	8.08	0.93	0.35553
ZNF26	-0.08	7.06	-0.93	0.35579
PLEKHG5	0.15	6.09	0.93	0.35580
MARCHF3	0.13	6.20	0.93	0.35580
TRAV9-2	0.10	6.60	0.93	0.35598
MFN2	-0.12	10.54	-0.93	0.35600
DNAJA1P3	-0.12	5.54	-0.93	0.35602
PTCH2	-0.10	5.20	-0.93	0.35604
TBC1D5	-0.07	9.08	-0.93	0.35608
KIR3DX1	0.13	5.23	0.93	0.35610
NDRG2	0.11	8.11	0.93	0.35612
SIK2	-0.06	7.95	-0.93	0.35614
TXLNGY	-0.36	6.73	-0.93	0.35623
AC093388.1	-0.11	4.99	-0.93	0.35634
EIF5P1	-0.09	5.06	-0.93	0.35635
MAGI3	-0.12	5.09	-0.93	0.35644
TIAL1	-0.05	9.55	-0.93	0.35655
SPOP	0.05	9.97	0.93	0.35664
F13A1	-0.16	10.35	-0.93	0.35688
MYO1G	0.07	12.65	0.93	0.35691
PLAUR	-0.13	10.76	-0.93	0.35692
AC113398.1	0.15	8.70	0.93	0.35725
NPM1	0.08	11.14	0.93	0.35727
SUPT4H1	-0.09	5.91	-0.93	0.35735
LINC00239	0.08	5.09	0.93	0.35747
AC073869.1	-0.09	8.33	-0.93	0.35756
PIN4	0.09	7.19	0.93	0.35765
HNRNPUP1	-0.11	6.19	-0.93	0.35778
AL021707.8	-0.12	5.67	-0.93	0.35784
ABLIM3	0.16	7.83	0.93	0.35793
RAB5C	0.09	10.66	0.93	0.35806
METTL1	0.08	5.89	0.93	0.35807
STAG3L5P	-0.13	6.24	-0.93	0.35812
FAM241A	-0.10	6.22	-0.93	0.35815
NECAB3	0.10	6.80	0.93	0.35816
TSPAN15	0.14	5.71	0.93	0.35888
GPC2	0.13	5.60	0.93	0.35890
H3P6	-0.13	9.26	-0.93	0.35896

ZBED5-AS1	0.08	6.67	0.93	0.35898
WDCP	-0.08	7.04	-0.92	0.35927
DIAPH2	-0.08	7.46	-0.92	0.35934
AC011379.2	0.10	4.97	0.92	0.35941
NUTF2P2	0.09	5.38	0.92	0.35943
TANGO6	-0.07	7.82	-0.92	0.35946
RPL12P6	0.11	5.64	0.92	0.35946
AGAP7P	-0.18	7.40	-0.92	0.35951
CFAP45	-0.15	6.78	-0.92	0.35960
CDC40	-0.08	7.68	-0.92	0.35961
PEG13	0.09	6.82	0.92	0.35965
ABCC2	-0.14	5.59	-0.92	0.35970
DERL3	0.18	7.19	0.92	0.35977
ANKRD44-AS1	0.15	5.99	0.92	0.35979
ITGAX	-0.13	13.57	-0.92	0.35981
SLC35B4	-0.07	6.99	-0.92	0.35985
UBAP1	-0.11	10.43	-0.92	0.36004
HLA-DQA2	-0.46	8.09	-0.92	0.36011
CD69	-0.10	6.58	-0.92	0.36022
CHD8	-0.05	10.50	-0.92	0.36025
PHF10	-0.07	8.66	-0.92	0.36034
FBXO6	-0.13	8.31	-0.92	0.36048
AC092279.1	-0.11	5.81	-0.92	0.36049
CAMK2D	-0.09	7.62	-0.92	0.36049
AL590064.1	0.12	5.20	0.92	0.36061
MALINC1	0.10	4.99	0.92	0.36068
FTCDNL1	0.10	5.00	0.92	0.36074
TMEM18	0.07	8.29	0.92	0.36076
AC106872.6	0.09	5.11	0.92	0.36082
ANG	-0.11	5.74	-0.92	0.36086
SLC25A4	0.09	6.56	0.92	0.36096
CNKSR1	0.15	5.80	0.92	0.36102
TAF4B	-0.12	5.85	-0.92	0.36113
SSU72	0.07	11.39	0.92	0.36118
SYNJ2	-0.09	7.17	-0.92	0.36149
PAIP2	-0.07	10.43	-0.92	0.36162
SRSF9	0.06	10.32	0.92	0.36163
TRIM47	0.11	6.86	0.92	0.36178
C11orf74	-0.10	5.18	-0.92	0.36183
PLAU	-0.13	5.25	-0.92	0.36192
ABRAXAS2	-0.07	7.50	-0.92	0.36193
CCDC78	0.12	5.35	0.92	0.36206
NPM1P39	-0.11	6.13	-0.92	0.36207
TMEM272	-0.19	6.07	-0.92	0.36215
FAF2	-0.06	8.70	-0.92	0.36220
AC000123.3	-0.11	5.07	-0.92	0.36224
PPIF	-0.08	10.20	-0.92	0.36236
AL592293.2	-0.09	5.28	-0.92	0.36237
KIAA2026	0.11	8.67	0.92	0.36249
MTF2	-0.07	7.60	-0.92	0.36252

IGHV2-70	0.16	5.47	0.92	0.36257
ASB2	0.10	6.70	0.92	0.36258
SF3B3	0.06	11.07	0.92	0.36259
HNRNPA1P8	-0.08	5.39	-0.92	0.36281
CYSLTR1	-0.11	7.24	-0.92	0.36285
ZNF846	0.09	5.62	0.92	0.36286
TMEM65	-0.08	7.35	-0.92	0.36290
ECI2	0.08	8.05	0.92	0.36290
RNF20	-0.06	9.21	-0.92	0.36290
SNRPGP2	-0.08	5.08	-0.92	0.36291
GK5	-0.11	7.31	-0.92	0.36298
HNRNPR	-0.07	10.28	-0.92	0.36301
RBM26	0.09	8.78	0.92	0.36330
TMCO3	-0.10	8.92	-0.92	0.36341
ANKMY1	0.06	8.23	0.92	0.36351
PHLPP2	-0.10	6.54	-0.92	0.36352
RPL30P7	0.09	5.73	0.92	0.36357
PABPC1L	-0.15	8.61	-0.92	0.36363
ERCC6L2	-0.07	7.53	-0.92	0.36370
BAIAP2L2	0.10	5.44	0.92	0.36373
ADAM23	-0.14	5.45	-0.92	0.36379
NPIPB4	-0.14	10.66	-0.92	0.36382
TTN	-0.16	5.98	-0.92	0.36386
LINC01513	-0.13	5.12	-0.92	0.36402
HPCAL1	0.08	10.53	0.92	0.36414
DLG5	-0.13	6.95	-0.92	0.36423
SAV1	-0.09	7.25	-0.92	0.36425
PDE8A	-0.11	7.20	-0.92	0.36426
CMAS	0.07	6.93	0.92	0.36429
MKNK2	0.08	12.25	0.92	0.36430
GBP3	-0.18	7.42	-0.92	0.36435
MTRNR2L12	0.13	7.72	0.92	0.36442
CCDC9B	-0.11	6.15	-0.91	0.36446
BCORL1	-0.08	8.61	-0.91	0.36447
GNG11	0.17	8.28	0.91	0.36448
RPL41	0.11	11.91	0.91	0.36458
EPS8L1	0.10	5.57	0.91	0.36469
PDGFA	0.13	5.68	0.91	0.36479
PPFIBP2	-0.09	8.33	-0.91	0.36484
ORAI2	0.10	11.33	0.91	0.36485
AC114402.1	0.08	4.98	0.91	0.36490
SIRT7	0.07	10.35	0.91	0.36491
STING1	0.08	11.20	0.91	0.36498
HSH2D	-0.12	11.52	-0.91	0.36501
NEDD8	0.10	9.38	0.91	0.36507
KDM5C	-0.08	10.79	-0.91	0.36514
C21orf62-AS1	0.10	5.53	0.91	0.36527
RAB3D	-0.11	10.88	-0.91	0.36544
CXorf40A	0.08	7.92	0.91	0.36559
AJ003147.3	0.15	6.16	0.91	0.36560

CLEC2D	-0.09	9.37	-0.91	0.36574
LINC00667	-0.09	7.97	-0.91	0.36581
TAZ	0.09	9.71	0.91	0.36585
CZIB	0.06	8.92	0.91	0.36594
PPP1R8	-0.05	8.77	-0.91	0.36599
DHX37	0.07	8.98	0.91	0.36619
ABI3	0.12	10.52	0.91	0.36623
CDC5L	-0.06	8.75	-0.91	0.36628
ARHGAP27	-0.10	12.07	-0.91	0.36638
SPTB	0.23	8.71	0.91	0.36642
GYPE	0.15	5.05	0.91	0.36647
BOLA3	-0.10	6.31	-0.91	0.36661
PEBP1P2	0.10	5.16	0.91	0.36675
ARHGEF40	-0.17	10.73	-0.91	0.36679
RPA2P2	-0.09	5.19	-0.91	0.36682
AC130371.2	0.10	5.29	0.91	0.36684
FGD3	0.08	13.06	0.91	0.36688
CYTH1	-0.06	12.01	-0.91	0.36691
PHF6	-0.09	6.31	-0.91	0.36696
SEMA4C	0.08	8.88	0.91	0.36696
RPL39P3	0.14	8.03	0.91	0.36704
P2RY2	-0.12	6.48	-0.91	0.36706
CCDC18	0.09	6.44	0.91	0.36717
TPCN2	-0.10	8.30	-0.91	0.36722
MORF4L1P1	-0.10	7.94	-0.91	0.36763
RGS12	0.08	7.48	0.91	0.36764
TNFRSF10C	-0.16	11.93	-0.91	0.36770
BRSK1	0.09	5.42	0.91	0.36783
MTX3	-0.08	6.61	-0.91	0.36793
DENND2D	0.07	11.03	0.91	0.36794
KIR3DL2	0.16	6.93	0.91	0.36808
FFAR2	-0.17	12.11	-0.91	0.36811
C5	-0.10	5.52	-0.91	0.36816
MRPL10	0.07	9.53	0.91	0.36827
DVL3	-0.07	9.60	-0.91	0.36830
GYPC	0.19	13.44	0.91	0.36833
VPS41	-0.05	9.10	-0.91	0.36833
EHD4	-0.07	9.12	-0.91	0.36834
LINC00494	0.11	5.62	0.91	0.36836
ITGA9-AS1	0.11	5.32	0.91	0.36839
AL049872.1	0.07	5.53	0.91	0.36845
HARBI1	0.09	6.22	0.91	0.36848
NOA1	0.06	8.52	0.91	0.36860
NR1D2	-0.10	7.50	-0.91	0.36860
PEX6	0.13	9.54	0.91	0.36863
AC009093.6	0.10	5.02	0.91	0.36865
AC016831.1	-0.11	5.10	-0.91	0.36881
RPS4XP6	0.14	6.30	0.91	0.36885
RETN	-0.17	7.03	-0.91	0.36889
AC064799.1	0.13	7.72	0.91	0.36895

SPARC	0.16	10.91	0.91	0.36953
RPARP-AS1	0.08	7.91	0.91	0.36954
C9orf40	0.09	6.18	0.91	0.36955
ZDHHC5	-0.05	10.32	-0.90	0.36976
ATF7IP2	0.09	6.69	0.90	0.36981
YBX1	0.09	13.52	0.90	0.36981
AKAP1	0.08	8.03	0.90	0.36998
GNAO1	0.14	7.09	0.90	0.37003
AC003072.1	0.08	6.58	0.90	0.37004
CNOT9	-0.05	8.75	-0.90	0.37013
HEXIM1	-0.07	9.38	-0.90	0.37031
MTMR9	-0.07	6.99	-0.90	0.37032
MORN2	0.11	5.07	0.90	0.37041
CDK5RAP3	-0.15	8.30	-0.90	0.37044
AMPD2	-0.11	12.00	-0.90	0.37050
TRBV10-2	0.15	5.36	0.90	0.37063
RPS4XP3	0.10	5.75	0.90	0.37066
DHX30	0.06	10.13	0.90	0.37072
BID	-0.08	10.22	-0.90	0.37077
ZNF335	0.06	10.06	0.90	0.37082
IGLV1-47	0.20	7.18	0.90	0.37101
ALKBH8	-0.10	6.19	-0.90	0.37104
AL121753.2	0.07	6.49	0.90	0.37108
PPP1R3F	0.09	6.82	0.90	0.37129
ABCC13	0.19	5.86	0.90	0.37137
BSCL2	0.08	6.17	0.90	0.37143
TMEM99	0.09	6.13	0.90	0.37149
CAPN15	0.08	9.46	0.90	0.37165
FLVCR1	0.11	7.06	0.90	0.37166
RPL34P31	0.12	6.36	0.90	0.37169
EPB42	0.24	8.95	0.90	0.37181
SLC5A6	0.06	8.09	0.90	0.37197
FLCN	-0.09	8.96	-0.90	0.37200
B3GLCT	-0.08	6.14	-0.90	0.37212
ZNF684	0.10	5.32	0.90	0.37217
TCEA1P2	-0.13	5.23	-0.90	0.37238
IGLV8-61	0.22	6.44	0.90	0.37243
CIR1	0.12	10.40	0.90	0.37245
ESRRA	0.06	9.21	0.90	0.37252
NAA50	-0.07	9.13	-0.90	0.37283
GNA12	0.13	9.92	0.90	0.37286
RABGAP1	-0.06	8.71	-0.90	0.37304
PHOSPHO1	0.20	12.52	0.90	0.37314
DCAF10	0.10	8.27	0.90	0.37366
KCNJ14	0.11	5.31	0.90	0.37368
TAMM41	-0.09	6.83	-0.90	0.37375
EEF1A1	0.09	16.20	0.90	0.37379
IQSEC2	-0.11	7.69	-0.90	0.37380
MICA	-0.09	8.15	-0.90	0.37385
U2AF1L5	0.10	6.56	0.90	0.37387

TCAP	0.11	5.19	0.90	0.37391
KIAA0319L	-0.12	11.17	-0.90	0.37403
AC011498.7	0.10	5.53	0.90	0.37415
S100A13	0.10	5.60	0.90	0.37422
ZNF100	-0.09	5.92	-0.90	0.37425
TMEM241	-0.09	5.73	-0.90	0.37426
FKBP5	-0.19	10.36	-0.90	0.37433
SGO2	-0.10	5.21	-0.90	0.37435
Z98886.1	-0.13	5.67	-0.90	0.37438
PRDX1	0.10	10.01	0.90	0.37442
SPACA9	0.09	5.81	0.90	0.37449
AC008725.1	0.11	5.52	0.90	0.37450
GEMIN4	0.07	8.37	0.90	0.37471
EIF1P5	0.10	6.73	0.90	0.37475
C16orf87	-0.08	5.76	-0.90	0.37477
FP565260.1	-0.12	6.76	-0.90	0.37479
MPHOSPH6	0.07	6.76	0.89	0.37503
C1RL-AS1	-0.12	6.59	-0.89	0.37525
LINC02724	-0.11	5.81	-0.89	0.37526
AJM1	0.12	7.01	0.89	0.37529
CDYL2	-0.09	6.55	-0.89	0.37537
STS	-0.10	6.88	-0.89	0.37542
WSB2	-0.08	9.09	-0.89	0.37549
TIFAB	-0.11	5.26	-0.89	0.37553
DMTN	0.23	12.94	0.89	0.37563
MIER2	0.08	8.25	0.89	0.37584
MREG	-0.09	5.62	-0.89	0.37607
SCARF1	-0.17	8.23	-0.89	0.37644
CXCR1	-0.17	13.28	-0.89	0.37658
EMILIN1	0.10	5.22	0.89	0.37670
VAMP5	0.11	9.12	0.89	0.37675
HSP90AA1	0.09	12.14	0.89	0.37677
EMC1	-0.06	8.66	-0.89	0.37689
XCR1	-0.12	5.17	-0.89	0.37693
STK24	-0.05	10.69	-0.89	0.37702
ADNP2	-0.07	7.63	-0.89	0.37709
LINC02482	0.12	5.41	0.89	0.37715
ARIH1	-0.06	9.01	-0.89	0.37720
GDF7	0.14	5.22	0.89	0.37762
MARCKS	-0.13	9.76	-0.89	0.37762
CDHR2	-0.10	5.01	-0.89	0.37765
KLHL17	0.12	7.36	0.89	0.37769
NCOA1	-0.09	10.47	-0.89	0.37770
BLVRA	-0.12	8.79	-0.89	0.37793
TBC1D9	-0.11	8.23	-0.89	0.37815
ARSA	0.09	10.22	0.89	0.37825
CENPF	-0.10	5.78	-0.89	0.37832
SNHG14	0.10	7.47	0.89	0.37834
IGHV3-33	0.21	7.77	0.89	0.37844
RNASEH1-AS1	0.08	5.97	0.89	0.37859

FKBP1C	-0.09	7.07	-0.89	0.37861
AC016596.3	0.14	6.46	0.89	0.37873
G3BP1	-0.05	9.96	-0.89	0.37875
ECHDC2	0.13	9.01	0.89	0.37882
PSMB3	0.10	11.19	0.89	0.37887
SLC7A8	0.19	6.00	0.89	0.37894
ZER1	0.10	11.05	0.89	0.37895
ZSCAN16	-0.07	6.96	-0.89	0.37896
CBWD2	-0.10	6.41	-0.89	0.37897
RETREG2	0.08	11.17	0.89	0.37900
NPRL2	0.07	9.32	0.89	0.37901
NDC80	-0.09	5.91	-0.89	0.37902
SPG21	-0.06	10.22	-0.89	0.37908
DLEU7	-0.13	5.49	-0.89	0.37909
KLHL25	0.09	6.75	0.89	0.37916
CCNDBP1	-0.09	10.66	-0.89	0.37917
AGPAT4	-0.10	7.83	-0.89	0.37946
RPH3A	0.20	7.94	0.89	0.37966
UBQLNL	-0.13	5.09	-0.89	0.37984
AC068205.2	0.11	5.10	0.89	0.37996
CAMKMT	0.09	5.97	0.89	0.37999
UBA6-AS1	0.08	7.01	0.89	0.38003
CDC27	-0.07	7.54	-0.88	0.38026
TPT1-AS1	0.09	8.20	0.88	0.38029
CHIC2	-0.11	7.69	-0.88	0.38043
PAXBP1-AS1	0.09	5.54	0.88	0.38045
TNFRSF12A	0.10	5.99	0.88	0.38047
ACVR2B	0.10	6.17	0.88	0.38048
DMPK	0.10	8.29	0.88	0.38054
NDEL1	-0.11	10.58	-0.88	0.38060
BEND3	-0.09	5.28	-0.88	0.38069
NRL	0.07	5.48	0.88	0.38080
LINC01560	-0.08	5.19	-0.88	0.38092
KIAA0586	-0.08	7.40	-0.88	0.38099
SOWAHD	0.09	7.69	0.88	0.38107
LRP3	0.12	7.30	0.88	0.38122
ZNF528	-0.07	6.67	-0.88	0.38125
RPS6KA4	0.09	10.16	0.88	0.38134
ZNF490	0.07	5.86	0.88	0.38157
EFEMP2	-0.15	6.78	-0.88	0.38158
AC090686.1	0.11	6.34	0.88	0.38162
AC092299.1	0.13	6.08	0.88	0.38171
TIGD2	-0.09	5.79	-0.88	0.38176
KRIT1	-0.07	6.54	-0.88	0.38182
AC008677.1	0.10	6.11	0.88	0.38193
CDK11A	0.11	6.87	0.88	0.38193
LIN9	-0.08	5.07	-0.88	0.38209
HPCAL4	0.14	6.45	0.88	0.38227
ALDH18A1	-0.06	8.30	-0.88	0.38236
SPDL1	-0.09	6.11	-0.88	0.38238

WDR70	0.06	8.13	0.88	0.38239
SYNRG	-0.06	9.99	-0.88	0.38248
LEMD2	0.05	9.97	0.88	0.38253
FUBP3	-0.14	7.56	-0.88	0.38260
TOX2	0.13	5.74	0.88	0.38276
HEATR1	-0.09	8.09	-0.88	0.38292
CYTIP	-0.06	10.78	-0.88	0.38301
RPL12P15	0.07	5.23	0.88	0.38314
COX7A2	0.08	9.45	0.88	0.38321
UPF3AP2	0.08	5.19	0.88	0.38323
BASP1-AS1	-0.14	5.21	-0.88	0.38325
SLC7A6	-0.10	9.45	-0.88	0.38337
AC105250.1	-0.12	5.93	-0.88	0.38337
GORASP2	0.06	9.46	0.88	0.38337
PSMA2	0.08	6.37	0.88	0.38354
DDX56	0.07	9.92	0.88	0.38375
IPP	0.13	6.12	0.88	0.38382
AKTIP	-0.08	8.06	-0.88	0.38420
IGKV1-12	0.17	5.89	0.88	0.38427
LINC01624	0.12	5.47	0.88	0.38428
IK	0.06	11.06	0.88	0.38429
BTNL8	-0.21	8.13	-0.88	0.38465
AC067942.1	0.16	7.23	0.88	0.38489
TATDN1	0.09	6.34	0.88	0.38489
YWHAEP1	0.09	5.08	0.88	0.38494
AC109449.1	0.12	5.48	0.88	0.38496
FAM24B	-0.11	5.48	-0.88	0.38503
AC005840.1	0.08	5.51	0.88	0.38512
UBE2Z	-0.05	9.94	-0.88	0.38533
CCPG1	-0.11	7.83	-0.88	0.38553
CEP44	-0.08	6.21	-0.88	0.38554
CUBN	-0.10	5.99	-0.87	0.38562
LILRB2	-0.10	12.14	-0.87	0.38583
GABARAP	0.10	8.26	0.87	0.38584
MAFF	-0.09	6.80	-0.87	0.38625
GRSF1	-0.06	8.89	-0.87	0.38630
TMEM254-AS1	0.11	5.03	0.87	0.38640
RFC1	-0.06	8.66	-0.87	0.38658
GALNT10	-0.06	9.59	-0.87	0.38659
OIP5-AS1	0.08	9.46	0.87	0.38665
AC091230.1	0.08	9.51	0.87	0.38668
AC008467.1	-0.12	5.68	-0.87	0.38672
TOB1	-0.10	8.70	-0.87	0.38683
TRMT5	0.08	6.40	0.87	0.38683
HAUS3	-0.08	6.74	-0.87	0.38700
AC016831.6	-0.12	5.40	-0.87	0.38715
LRBA	-0.06	8.64	-0.87	0.38723
ABCD2	-0.11	6.01	-0.87	0.38735
PIK3R1	-0.08	9.58	-0.87	0.38736
TSHZ1	-0.10	8.27	-0.87	0.38742

FANCL	-0.09	5.40	-0.87	0.38746
LGALS3BP	0.15	9.66	0.87	0.38746
PRMT9	-0.09	5.94	-0.87	0.38767
GALM	0.10	8.99	0.87	0.38776
AC099489.1	-0.17	10.18	-0.87	0.38795
ZSWIM4	0.07	6.90	0.87	0.38796
SLC25A30	-0.08	6.98	-0.87	0.38806
METRN	0.12	6.29	0.87	0.38810
AP000346.2	-0.10	5.59	-0.87	0.38834
GBAP1	-0.10	6.50	-0.87	0.38840
UBE2T	0.10	5.24	0.87	0.38841
NMB	0.10	5.71	0.87	0.38844
FOXO3B	0.09	5.68	0.87	0.38885
LYAR	0.07	8.72	0.87	0.38889
ZNF331	0.08	7.40	0.87	0.38941
TRAV8-1	0.14	5.73	0.87	0.38945
RPL17P36	0.10	6.35	0.87	0.38952
RPAP3	-0.06	8.00	-0.87	0.38964
TSPAN5	0.16	9.15	0.87	0.38985
CFAP58	-0.12	5.39	-0.87	0.38988
KIAA1143	-0.08	7.32	-0.87	0.39021
ZDHHC21	-0.08	6.01	-0.87	0.39032
KRTCAP3	0.11	5.94	0.87	0.39032
PFN2	-0.09	5.40	-0.87	0.39050
URB1-AS1	0.09	5.24	0.87	0.39053
AC024995.1	0.10	5.06	0.87	0.39059
MDH1	0.06	9.27	0.87	0.39076
GNAZ	0.13	8.25	0.87	0.39079
GMDS-DT	0.07	6.24	0.87	0.39102
NRDC	-0.08	10.99	-0.86	0.39106
PKIA	-0.11	6.57	-0.86	0.39110
EXOC5	-0.08	7.60	-0.86	0.39127
RASGRP4	-0.12	11.87	-0.86	0.39144
AL157713.1	-0.09	5.31	-0.86	0.39145
AC110994.3	0.12	6.90	0.86	0.39153
ARPC1A	0.08	7.26	0.86	0.39161
GPS2	0.10	5.73	0.86	0.39182
ZBP1	-0.14	9.69	-0.86	0.39183
AC005165.2	0.13	6.84	0.86	0.39188
AC009948.1	0.10	5.61	0.86	0.39189
AL358472.1	0.12	6.29	0.86	0.39190
ZNF568	-0.08	6.17	-0.86	0.39191
UBXN10	0.11	5.81	0.86	0.39195
TCN1	-0.16	6.89	-0.86	0.39196
AC132192.2	-0.10	5.36	-0.86	0.39203
TMEM237	-0.08	5.24	-0.86	0.39208
AL357055.1	0.09	5.80	0.86	0.39214
SIAE	-0.09	6.86	-0.86	0.39214
NPIPP1	0.10	6.73	0.86	0.39224
C10orf105	-0.15	7.91	-0.86	0.39230

PRKCQ	0.08	9.21	0.86	0.39243
SELENOWP1	0.11	5.10	0.86	0.39250
IGHJ2	0.18	5.48	0.86	0.39250
TPGS2	0.14	9.53	0.86	0.39251
RP9P	0.09	5.27	0.86	0.39254
UBE2Q2	-0.08	8.04	-0.86	0.39257
PPP2R3B	0.10	8.13	0.86	0.39265
EIF4A1P2	0.11	6.77	0.86	0.39282
KCNK5	-0.08	4.97	-0.86	0.39285
SNRPEP2	0.10	5.14	0.86	0.39289
PIP4K2B	0.04	10.03	0.86	0.39292
FAM98B	0.08	7.10	0.86	0.39292
DNM1	0.12	5.85	0.86	0.39307
AL136038.7	0.10	5.29	0.86	0.39364
ZNF180	-0.07	6.84	-0.86	0.39366
LINC01001	-0.17	7.82	-0.86	0.39372
CABIN1	0.05	10.93	0.86	0.39373
ZC3H12D	0.09	7.85	0.86	0.39381
GPATCH11	0.08	7.50	0.86	0.39385
RPS4XP2	0.15	7.72	0.86	0.39386
PIGB	-0.09	7.55	-0.86	0.39404
AOPEP	0.08	6.16	0.86	0.39417
MAGEF1	0.08	7.41	0.86	0.39419
BTK	-0.06	9.90	-0.86	0.39420
OPRL1	-0.08	8.58	-0.86	0.39432
SNX25	-0.08	6.36	-0.86	0.39444
PSRC1	-0.09	7.04	-0.86	0.39445
KIF3B	0.06	8.85	0.86	0.39447
SERBP1P1	-0.09	5.51	-0.86	0.39449
NOL3	-0.10	5.77	-0.86	0.39452
HMGN4	-0.06	9.78	-0.86	0.39454
CACNB4	-0.14	5.55	-0.86	0.39455
AL049714.1	-0.08	5.27	-0.86	0.39466
KIZ	-0.06	8.06	-0.86	0.39466
SENP5	-0.05	8.37	-0.86	0.39500
ALDH3B1	0.09	10.47	0.86	0.39502
TANC2	-0.11	7.28	-0.86	0.39505
IGKV3-15	0.19	8.17	0.86	0.39521
SECISBP2	-0.06	9.73	-0.86	0.39523
AC008750.2	-0.12	5.81	-0.86	0.39528
COX16	0.08	5.25	0.86	0.39529
RPL17P43	0.11	6.21	0.86	0.39533
PLEKHM1P1	-0.11	9.37	-0.86	0.39550
POP5	0.07	7.89	0.86	0.39562
ISCU	0.06	10.47	0.86	0.39566
TPD52	0.11	6.67	0.86	0.39570
SETDB2	-0.06	7.38	-0.86	0.39575
QRICH2	0.09	5.98	0.86	0.39583
EIF4BP6	0.11	6.95	0.86	0.39592
LTV1	-0.09	6.87	-0.86	0.39606

C12orf49	-0.05	8.76	-0.86	0.39614
LPAR1	-0.12	6.10	-0.86	0.39632
SFRP2	0.21	6.26	0.86	0.39640
RPS26P28	0.14	5.28	0.86	0.39642
VBP1	-0.06	8.02	-0.86	0.39643
AP001803.1	-0.10	5.19	-0.86	0.39644
AL121944.2	0.10	5.06	0.86	0.39645
BTF3P13	0.07	5.26	0.86	0.39646
STON2	-0.09	5.92	-0.85	0.39666
CRELD2	0.06	9.02	0.85	0.39669
IGFBP3	0.15	7.06	0.85	0.39677
AC009831.1	0.09	5.09	0.85	0.39680
DNAJC16	0.07	7.06	0.85	0.39681
AFF2	-0.10	5.20	-0.85	0.39690
TPCN1	0.09	9.82	0.85	0.39704
TMEM107	0.06	7.27	0.85	0.39712
FEN1	0.07	7.65	0.85	0.39715
RPL24P8	0.14	7.81	0.85	0.39717
NRP2	-0.10	5.07	-0.85	0.39719
IGHV3-30	0.22	8.19	0.85	0.39724
MRE11	-0.07	7.54	-0.85	0.39725
ATP5MC2P3	-0.10	5.11	-0.85	0.39732
NOMO2	-0.09	7.78	-0.85	0.39751
UHRF1	0.09	5.22	0.85	0.39762
AC007728.2	-0.12	6.51	-0.85	0.39768
AC006483.1	-0.14	6.69	-0.85	0.39782
BCAT1	-0.11	5.92	-0.85	0.39793
RRM1	-0.07	7.70	-0.85	0.39793
HOOK1	-0.10	5.50	-0.85	0.39807
RHCE	0.14	5.03	0.85	0.39810
AC004890.1	0.08	5.61	0.85	0.39826
NCAM1	-0.13	7.78	-0.85	0.39836
ADAMTSL4-AS1	-0.15	6.60	-0.85	0.39838
GPR89A	0.08	5.90	0.85	0.39843
GDPGP1	-0.08	5.93	-0.85	0.39852
CUL2	-0.05	8.09	-0.85	0.39867
AC135050.3	-0.11	5.26	-0.85	0.39890
RAB11FIP2	-0.08	6.34	-0.85	0.39931
IQCN	-0.15	7.59	-0.85	0.39947
VMAC	0.08	6.71	0.85	0.39948
CACNA1A	0.11	5.58	0.85	0.39949
AC090589.1	0.12	7.38	0.85	0.39951
AC024909.1	-0.07	6.13	-0.85	0.39954
THADA	0.06	8.74	0.85	0.39958
PIAS3	-0.06	7.96	-0.85	0.39968
CPA3	-0.14	6.07	-0.85	0.39969
AL512353.2	0.10	5.69	0.85	0.39980
AGTRAP	0.10	11.15	0.85	0.39992
CXCL10	-0.15	5.09	-0.85	0.40011
DDX10	-0.06	7.22	-0.85	0.40017

TUBB4A	0.11	6.45	0.85	0.40020
CXCR2P1	-0.13	8.29	-0.85	0.40035
DIAPH1	-0.04	12.47	-0.85	0.40040
SHLD3	-0.10	5.11	-0.85	0.40045
ADCY9	-0.08	8.09	-0.85	0.40052
INTS4	0.05	8.73	0.85	0.40060
AC092384.3	0.11	4.93	0.85	0.40084
SNX9	-0.09	7.67	-0.85	0.40085
WDR48	-0.06	8.95	-0.85	0.40100
IFT52	0.05	7.73	0.85	0.40106
EPHA4	-0.10	6.73	-0.85	0.40108
SETD5	-0.07	9.89	-0.85	0.40151
AC138969.1	-0.08	6.41	-0.85	0.40158
AL162377.1	0.10	5.83	0.85	0.40170
GUCY1B1	-0.13	6.07	-0.85	0.40177
AC009506.2	-0.12	7.58	-0.85	0.40195
TEDC1	0.10	6.83	0.85	0.40197
POLR3G	0.13	6.16	0.84	0.40227
MAP3K2-DT	-0.10	5.23	-0.84	0.40230
TMEM138	0.06	8.44	0.84	0.40246
WDR45	0.09	9.82	0.84	0.40253
MARCHF1	-0.09	8.38	-0.84	0.40260
C2orf15	0.12	5.19	0.84	0.40262
AC083862.2	-0.12	7.92	-0.84	0.40275
LDHAP7	0.08	5.07	0.84	0.40279
BLM	0.08	6.54	0.84	0.40296
ZSWIM5	-0.10	5.69	-0.84	0.40296
ANAPC5	0.04	10.76	0.84	0.40299
EEF1AKMT1	0.10	5.49	0.84	0.40304
AC011899.3	-0.11	5.68	-0.84	0.40312
ACTG1P1	0.11	5.73	0.84	0.40324
ATG16L1	-0.05	8.66	-0.84	0.40347
DCLRE1B	-0.06	7.23	-0.84	0.40362
NFE2L1	-0.05	10.57	-0.84	0.40363
HEMK1	0.08	8.66	0.84	0.40363
AKAP17A	-0.08	10.37	-0.84	0.40371
RIPK3	-0.08	8.94	-0.84	0.40374
TMED10	-0.06	10.29	-0.84	0.40385
CDK6	-0.09	7.68	-0.84	0.40389
AC124248.1	0.11	5.46	0.84	0.40390
N4BP3	0.11	6.39	0.84	0.40391
RELL2	0.10	7.73	0.84	0.40435
RPL7AP31	0.11	6.45	0.84	0.40439
C4orf50	-0.11	5.10	-0.84	0.40451
OTUD5	0.06	11.34	0.84	0.40457
PRSS23	-0.17	8.28	-0.84	0.40459
ATP5PF	0.08	8.52	0.84	0.40476
PPIAP11	0.08	5.03	0.84	0.40494
PRIM2	-0.08	5.97	-0.84	0.40496
SEC24C	-0.04	11.30	-0.84	0.40500

AC099535.1	0.13	7.97	0.84	0.40511
SRFBP1	-0.07	5.60	-0.84	0.40511
NNT-AS1	0.08	6.40	0.84	0.40538
AC010883.2	-0.09	5.56	-0.84	0.40539
EEF1A1P29	-0.07	5.27	-0.84	0.40544
CEP170B	0.11	5.76	0.84	0.40548
MTCO2P2	0.11	5.15	0.84	0.40559
AL391825.1	-0.12	5.44	-0.84	0.40563
CCT6P1	-0.08	6.21	-0.84	0.40573
ZDHHC18	-0.12	12.57	-0.84	0.40579
HNRNPA1P21	-0.09	5.71	-0.84	0.40586
SLC35B2	0.06	8.99	0.84	0.40604
LINC00989	-0.12	6.90	-0.84	0.40618
NTSR1	-0.16	5.51	-0.84	0.40619
RNASET2	0.11	11.61	0.84	0.40627
MMEL1	0.11	5.28	0.84	0.40629
ELOBP2	0.08	5.71	0.84	0.40635
KCNH3	-0.12	7.24	-0.84	0.40636
RPRD2	0.06	9.63	0.84	0.40641
ANKRD42	-0.07	6.15	-0.84	0.40649
VAMP4	-0.07	7.19	-0.84	0.40653
PRKACB	-0.10	8.56	-0.84	0.40657
LRRC37A	-0.12	5.34	-0.84	0.40666
GTF2A2	0.07	8.07	0.84	0.40671
DNAJC15	0.09	7.91	0.84	0.40689
IGKV2-24	0.20	6.14	0.84	0.40690
MTHFD2L	0.08	5.54	0.84	0.40691
AC022149.1	-0.11	5.90	-0.84	0.40696
ASB1	-0.06	8.79	-0.84	0.40700
AL022345.4	0.11	5.16	0.84	0.40712
SMARCE1P5	-0.09	5.42	-0.84	0.40716
SLC27A2	-0.09	4.89	-0.84	0.40721
AP002478.1	-0.11	5.42	-0.84	0.40731
TANGO2	0.08	10.20	0.84	0.40732
PAM16	-0.11	7.07	-0.84	0.40747
UQCRFS1P1	0.08	5.09	0.83	0.40757
GABARAPL2	-0.09	10.09	-0.83	0.40794
AC027309.2	0.10	6.80	0.83	0.40794
SEC11C	0.09	8.28	0.83	0.40801
AC104794.3	0.08	7.22	0.83	0.40816
POLE3	0.06	9.33	0.83	0.40828
ZNF681	0.09	5.53	0.83	0.40830
HOXA-AS2	0.09	5.00	0.83	0.40837
IER3IP1	0.08	6.50	0.83	0.40851
ATP1B1	-0.09	5.63	-0.83	0.40854
DLEU2	-0.10	7.35	-0.83	0.40869
SUCLA2	-0.07	7.06	-0.83	0.40872
NDRG1	-0.06	11.31	-0.83	0.40881
GUCY1A1	-0.11	5.40	-0.83	0.40904
PPP1CB	-0.07	9.77	-0.83	0.40910

TPPP3	0.16	8.12	0.83	0.40923
CCND3	0.07	12.41	0.83	0.40933
RPL34P18	0.15	8.06	0.83	0.40947
ZG16B	-0.10	5.45	-0.83	0.40960
NAPA	-0.09	10.75	-0.83	0.40964
PRDM11	-0.07	6.82	-0.83	0.40971
SH3BGRL2	0.14	7.46	0.83	0.40977
LINC01772	-0.08	5.26	-0.83	0.40991
AC098591.3	0.11	7.01	0.83	0.40991
RN7SL364P	0.11	5.07	0.83	0.41000
LSMEM1	-0.11	6.42	-0.83	0.41003
RNF123	0.10	9.95	0.83	0.41005
FCER1G	-0.11	12.09	-0.83	0.41011
AC008736.1	-0.08	5.19	-0.83	0.41022
MYB	0.08	6.15	0.83	0.41022
IGKV1-5	0.19	8.50	0.83	0.41023
IGHJ1	0.11	5.13	0.83	0.41043
SARS2	0.08	5.42	0.83	0.41052
SIRPB1	-0.15	10.82	-0.83	0.41059
AMMECR1	0.08	6.75	0.83	0.41080
ADGRE4P	-0.16	6.55	-0.83	0.41085
WDR41	-0.06	7.65	-0.83	0.41116
CERS2	-0.05	10.93	-0.83	0.41126
CHI3L2	0.12	7.35	0.83	0.41150
GTPBP3	0.08	7.19	0.83	0.41152
CHAMP1	-0.08	7.51	-0.83	0.41174
AL133216.2	-0.10	6.56	-0.83	0.41177
CCDC170	-0.11	6.44	-0.83	0.41177
CROCCP2	0.10	8.33	0.83	0.41182
AC092164.1	-0.09	4.90	-0.83	0.41183
SPCS3	-0.05	9.75	-0.83	0.41185
FLACC1	0.10	5.31	0.83	0.41191
AC007237.1	0.09	5.02	0.83	0.41192
ZNF44	0.06	8.09	0.83	0.41192
KLF6	0.10	12.17	0.83	0.41193
RHOG	0.10	12.85	0.83	0.41195
PDP2	0.09	6.69	0.83	0.41199
TMEM79	0.07	7.56	0.83	0.41203
DENND4C	0.09	7.69	0.83	0.41204
ILKAP	0.06	8.50	0.83	0.41204
SLC10A7	-0.08	5.92	-0.83	0.41208
CFD	0.23	10.59	0.83	0.41211
COQ5	0.05	7.66	0.83	0.41213
ZKSCAN5	-0.05	7.64	-0.83	0.41217
LYPLA2P1	0.08	5.52	0.83	0.41223
XPO6	-0.12	13.47	-0.83	0.41223
AC007160.1	0.08	5.74	0.83	0.41226
SNX24	-0.07	5.61	-0.83	0.41229
YKT6	0.05	10.11	0.83	0.41230
SF3A3P2	-0.09	5.30	-0.83	0.41258

AC012645.4	-0.10	5.28	-0.83	0.41278
AC092490.1	-0.38	9.46	-0.82	0.41318
EED	-0.06	7.43	-0.82	0.41329
ATP6V0E1	0.09	11.21	0.82	0.41334
RPL26P19	0.14	8.56	0.82	0.41337
CLEC17A	0.14	6.78	0.82	0.41338
LEF1-AS1	0.12	5.21	0.82	0.41341
ANAPC1	-0.07	7.36	-0.82	0.41345
ZNF69	-0.08	5.46	-0.82	0.41349
RBPJ	0.11	10.29	0.82	0.41381
FANCI	-0.08	6.82	-0.82	0.41401
ZNF132	-0.14	5.72	-0.82	0.41402
HS3ST3B1	0.09	7.52	0.82	0.41427
COPS2	-0.08	7.67	-0.82	0.41431
ELL	-0.09	10.22	-0.82	0.41435
AC012676.5	-0.12	5.64	-0.82	0.41439
AC007611.1	0.09	5.18	0.82	0.41444
TMEM63C	-0.13	6.70	-0.82	0.41456
COG4	0.05	9.65	0.82	0.41469
TFB2M	-0.07	6.47	-0.82	0.41471
AC009779.3	-0.07	6.51	-0.82	0.41473
URB2	-0.08	6.93	-0.82	0.41497
PKHD1L1	-0.12	5.58	-0.82	0.41500
RNF4	-0.04	10.07	-0.82	0.41511
PRDM1	-0.09	9.07	-0.82	0.41513
KCTD1	-0.08	5.54	-0.82	0.41514
HEBP2	-0.07	9.89	-0.82	0.41520
RMDN3	-0.06	8.22	-0.82	0.41522
CYP2R1	0.07	6.77	0.82	0.41524
HNRNPD	-0.06	10.54	-0.82	0.41528
PALM2AKAP2	-0.10	7.65	-0.82	0.41531
AC005280.2	-0.13	6.22	-0.82	0.41547
DUSP1	-0.15	11.40	-0.82	0.41555
GPR160	-0.11	7.04	-0.82	0.41573
SPATA24	0.09	5.26	0.82	0.41580
MTUS1	0.12	5.37	0.82	0.41585
COTL1	0.08	14.50	0.82	0.41586
ZNF830	-0.04	8.32	-0.82	0.41589
SMUG1	0.07	7.95	0.82	0.41593
MTDHP3	-0.16	4.97	-0.82	0.41595
ESD	0.07	8.89	0.82	0.41618
MINDY1	-0.10	10.43	-0.82	0.41623
TRMT10A	-0.08	5.49	-0.82	0.41634
LASP1	-0.07	12.77	-0.82	0.41651
CAMKK2	-0.08	10.87	-0.82	0.41652
CAMP	0.22	8.47	0.82	0.41674
CTSG	-0.24	6.45	-0.82	0.41682
AL031775.1	-0.08	5.57	-0.82	0.41682
CCR5AS	0.14	5.39	0.82	0.41686
LINC00211	-0.13	5.83	-0.82	0.41699

ATP5MC1	0.10	8.78	0.82	0.41710
HLA-DRB1	0.12	13.34	0.82	0.41712
LGALS9DP	-0.09	5.59	-0.82	0.41723
AL118506.1	0.07	7.31	0.82	0.41726
NRSN2	0.10	5.67	0.82	0.41732
CYP2U1	-0.08	6.18	-0.82	0.41751
TUBAP2	-0.11	6.73	-0.82	0.41753
HCG27	-0.16	9.58	-0.82	0.41763
UBE3D	0.10	5.78	0.82	0.41764
CHMP3	0.07	9.27	0.82	0.41776
LAP3P2	-0.14	5.50	-0.82	0.41786
ZNF445	-0.08	8.20	-0.82	0.41800
MESD	0.05	8.90	0.82	0.41800
PPIL3	0.08	6.77	0.82	0.41800
AC132872.1	-0.11	6.79	-0.82	0.41806
AL627309.6	0.16	7.35	0.82	0.41813
DAGLB	0.06	9.25	0.82	0.41817
RASSF4	-0.09	9.94	-0.82	0.41824
GAS6-AS1	0.18	6.19	0.82	0.41832
ZNF182	-0.07	7.26	-0.82	0.41835
AUH	-0.07	6.30	-0.82	0.41836
TUBA1A	-0.11	12.05	-0.82	0.41841
STX1B	0.10	5.27	0.82	0.41850
AC106806.1	0.11	6.51	0.82	0.41864
PVT1	0.09	7.40	0.82	0.41870
ARPC3P1	-0.10	7.12	-0.82	0.41873
TBCAP1	0.10	5.92	0.81	0.41885
TFE3	-0.08	11.02	-0.81	0.41889
RBM8B	0.10	6.28	0.81	0.41897
TNRC6A	0.07	9.31	0.81	0.41909
BTBD19	-0.15	5.78	-0.81	0.41914
LOXHD1	-0.13	5.96	-0.81	0.41926
EIF4ENIF1	0.04	8.86	0.81	0.41934
RBKS	0.07	5.50	0.81	0.41960
PGAM1	-0.06	9.14	-0.81	0.41962
LYSMD4	0.08	6.93	0.81	0.41982
AC011005.1	0.09	6.83	0.81	0.42009
AL390039.1	0.09	5.26	0.81	0.42025
ZFHX2-AS1	0.11	5.61	0.81	0.42026
PDCD2L	-0.10	5.65	-0.81	0.42034
DGCR2	0.07	11.37	0.81	0.42036
ETFRF1	-0.08	6.37	-0.81	0.42037
SUPT3H	0.09	6.41	0.81	0.42042
CD1A	-0.09	5.60	-0.81	0.42047
SLU7	0.07	9.80	0.81	0.42065
SDCBP2-AS1	-0.08	5.96	-0.81	0.42085
DSEL	-0.10	5.39	-0.81	0.42115
TMEM42	0.07	7.42	0.81	0.42119
IFI35	-0.15	10.01	-0.81	0.42123
CBFB	-0.06	8.36	-0.81	0.42126

UNKL	-0.10	9.07	-0.81	0.42134
AC006369.1	0.10	6.01	0.81	0.42143
THEMIS	-0.11	7.03	-0.81	0.42149
MAF	-0.09	8.41	-0.81	0.42168
PHYH	0.07	6.84	0.81	0.42171
THBS3	-0.10	8.36	-0.81	0.42173
MRPL35	0.05	7.60	0.81	0.42174
MSMO1	-0.07	6.50	-0.81	0.42182
ISYNA1	0.09	8.51	0.81	0.42183
HMGB2	-0.11	11.01	-0.81	0.42184
CD4	0.09	12.72	0.81	0.42186
SOX8	0.13	5.73	0.81	0.42205
NDUFAF7	-0.07	7.07	-0.81	0.42208
SH2B2	0.11	9.00	0.81	0.42222
ARMCX5-GPRASP2	0.08	5.23	0.81	0.42233
MFHAS1	0.07	8.47	0.81	0.42243
COMMD5	0.08	8.98	0.81	0.42244
KCNIP2	0.12	5.95	0.81	0.42260
RAMACL	0.07	5.69	0.81	0.42271
AC244197.3	-0.12	5.05	-0.81	0.42275
HP	-0.20	7.58	-0.81	0.42289
C1orf159	0.09	7.26	0.81	0.42291
GOLGA2	-0.06	9.52	-0.81	0.42305
C11orf58	0.05	9.19	0.81	0.42359
FSD1	0.11	5.67	0.81	0.42360
SLC39A7	0.06	9.85	0.81	0.42368
AC007686.3	-0.10	6.32	-0.81	0.42370
ZNF765	-0.08	6.47	-0.81	0.42377
AFG3L1P	-0.07	7.84	-0.81	0.42384
RRAGD	-0.10	7.47	-0.81	0.42397
DHRS1	0.08	8.62	0.81	0.42398
GLRX5	0.17	10.15	0.81	0.42412
FXR1	-0.06	9.20	-0.81	0.42416
SF3B6	0.07	9.38	0.81	0.42421
HRH2	-0.15	11.85	-0.81	0.42422
RADX	-0.10	5.18	-0.80	0.42457
VCP	0.04	11.89	0.80	0.42475
ELAVL1	0.05	9.61	0.80	0.42483
EXOC6B	-0.08	6.37	-0.80	0.42486
ZSCAN22	0.06	6.55	0.80	0.42504
DHX29	-0.07	7.56	-0.80	0.42505
TBC1D16	-0.09	6.18	-0.80	0.42516
LPIN1	0.05	8.88	0.80	0.42533
GP6	0.11	6.33	0.80	0.42534
AC012510.1	0.09	5.96	0.80	0.42555
DGKA	0.08	11.41	0.80	0.42569
SAR1B	-0.05	7.95	-0.80	0.42577
RAB30	0.10	6.81	0.80	0.42579
ZNF586	-0.07	7.80	-0.80	0.42580
TPM2	0.13	7.58	0.80	0.42592

SERBP1P5	-0.10	5.84	-0.80	0.42593
RPS18P9	0.07	6.23	0.80	0.42604
SEC61B	0.10	8.69	0.80	0.42607
HYLS1	-0.07	6.70	-0.80	0.42629
CSF1R	-0.10	12.18	-0.80	0.42630
ANKRD33B	-0.13	6.70	-0.80	0.42637
ENC1	-0.14	7.38	-0.80	0.42640
TGFB3	0.12	5.64	0.80	0.42643
FAM214B	-0.10	10.50	-0.80	0.42665
GSPT2	0.06	7.00	0.80	0.42677
NDUFS1	-0.04	8.44	-0.80	0.42686
NAT10	0.06	9.65	0.80	0.42688
RILP	0.15	8.87	0.80	0.42713
ASTE1	-0.06	7.74	-0.80	0.42714
MPDU1	0.07	9.20	0.80	0.42720
PHF7	0.07	6.12	0.80	0.42723
LCOR	-0.06	8.72	-0.80	0.42724
FOXO1	0.06	8.86	0.80	0.42730
CKB	0.14	6.88	0.80	0.42771
ATG4C	-0.07	6.75	-0.80	0.42782
FP565260.6	-0.08	6.45	-0.80	0.42801
RAB9A	-0.06	7.73	-0.80	0.42810
EIF1AX	0.08	7.94	0.80	0.42814
FASTKD1	-0.06	6.54	-0.80	0.42816
UBE2H	-0.08	10.49	-0.80	0.42824
PPCS	-0.04	8.76	-0.80	0.42832
KDELR2	0.05	9.32	0.80	0.42852
AC131025.1	0.10	4.97	0.80	0.42852
CFAP410	0.10	8.02	0.80	0.42852
SCO2	-0.14	9.47	-0.80	0.42871
FRAT2	-0.11	11.79	-0.80	0.42873
LIMD1-AS1	-0.08	5.71	-0.80	0.42883
LINC01578	0.07	8.43	0.80	0.42884
IGKJ4	0.17	5.75	0.80	0.42894
WDR45B	-0.04	9.42	-0.80	0.42903
TNFRSF11A	-0.10	5.00	-0.80	0.42922
POTEM	0.09	5.93	0.80	0.42936
KLK1	0.14	5.84	0.80	0.42938
AC026748.3	-0.09	5.81	-0.80	0.42956
AC012615.1	0.08	6.34	0.80	0.42968
ZKSCAN4	-0.06	7.30	-0.80	0.42983
USP51	0.08	5.05	0.80	0.42988
GTPBP10	-0.07	6.89	-0.80	0.42995
CWC15	0.06	8.64	0.80	0.43014
AC112247.1	-0.09	5.29	-0.80	0.43018
BCAS3	-0.05	8.82	-0.79	0.43027
TENT5C	0.19	9.80	0.79	0.43035
ANAPC13	0.06	8.14	0.79	0.43067
R3HDM4	0.14	13.48	0.79	0.43071
S100P	0.26	10.69	0.79	0.43077

CFAP58-DT	-0.11	5.29	-0.79	0.43104
CYP1B1-AS1	-0.13	5.46	-0.79	0.43110
GID8	0.04	10.00	0.79	0.43110
SNORD89	0.11	5.75	0.79	0.43112
CUTALP	0.16	8.11	0.79	0.43117
AC008427.2	0.08	5.06	0.79	0.43127
RTCB	0.07	9.92	0.79	0.43131
PAQR6	-0.17	6.58	-0.79	0.43138
SCFD2	0.06	7.63	0.79	0.43144
MRPS22	-0.06	7.99	-0.79	0.43154
HSP90AB1	0.06	12.56	0.79	0.43158
MCUB	0.08	9.04	0.79	0.43164
TBC1D22B	0.09	8.34	0.79	0.43164
MYL6P2	0.06	5.30	0.79	0.43181
DHX35	-0.06	7.31	-0.79	0.43187
SLC25A28	-0.07	9.47	-0.79	0.43197
AC005622.1	-0.11	5.36	-0.79	0.43206
ZNF614	-0.07	6.21	-0.79	0.43210
RBM6	-0.07	10.45	-0.79	0.43221
PHYHD1	0.14	5.09	0.79	0.43226
AC017035.1	0.11	5.33	0.79	0.43236
WDHD1	-0.08	5.24	-0.79	0.43236
TNFRSF10D	0.08	6.91	0.79	0.43241
OR2A20P	0.10	4.89	0.79	0.43248
SH3D21	0.10	6.41	0.79	0.43256
AC068338.2	0.09	5.39	0.79	0.43258
HSPA8P8	-0.12	6.56	-0.79	0.43294
CCL3	-0.12	6.68	-0.79	0.43305
ETAA1	-0.07	6.18	-0.79	0.43324
NOC3L	-0.09	6.38	-0.79	0.43325
INTS6-AS1	-0.09	5.39	-0.79	0.43325
RAC1	0.08	11.51	0.79	0.43330
CEBPB	-0.12	12.04	-0.79	0.43344
AC063947.2	-0.09	5.14	-0.79	0.43345
NPM1P24	0.11	5.38	0.79	0.43358
ALDH1B1	-0.08	6.60	-0.79	0.43368
CARD16	-0.10	10.07	-0.79	0.43373
SLC2A9	-0.08	7.07	-0.79	0.43375
SLC18B1	-0.07	7.37	-0.79	0.43375
GPC1	0.08	4.96	0.79	0.43376
ZFP30	-0.07	5.50	-0.79	0.43387
PLGLB1	-0.08	4.95	-0.79	0.43391
ANKRD9	0.15	7.31	0.79	0.43393
JMY	-0.07	6.48	-0.79	0.43404
METTL3	-0.07	8.66	-0.79	0.43409
AL354877.1	-0.11	5.60	-0.79	0.43411
FAM117A	0.09	10.62	0.79	0.43416
PPP1R32	0.09	6.69	0.79	0.43422
GRIPAP1	0.06	10.67	0.79	0.43423
GRK3	-0.07	9.20	-0.79	0.43432

ZNF213	0.06	8.61	0.79	0.43432
CWC27	0.07	7.43	0.79	0.43453
PMS2	-0.07	6.87	-0.79	0.43463
CNN2P6	-0.10	5.38	-0.79	0.43469
AP3D1	0.04	11.38	0.79	0.43469
DIP2C	-0.09	5.79	-0.79	0.43473
IFT27	0.07	7.62	0.79	0.43479
MTCH2	-0.06	8.34	-0.79	0.43524
WNT10B	0.11	6.27	0.79	0.43540
ZNF890P	0.12	5.20	0.79	0.43541
SNAP29	0.05	9.43	0.79	0.43552
HOPX	-0.11	8.55	-0.79	0.43553
TUBBP1	-0.08	5.69	-0.79	0.43563
AL009174.1	0.10	7.51	0.79	0.43577
NOL6	0.06	9.23	0.79	0.43588
AP000926.2	-0.08	5.30	-0.79	0.43592
EEF1DP5	0.09	5.85	0.78	0.43617
MMP14	-0.11	6.62	-0.78	0.43618
KPNA5	0.08	6.37	0.78	0.43633
AC022494.1	0.11	5.43	0.78	0.43652
AC004381.3	0.13	5.44	0.78	0.43656
DYSF	-0.15	12.79	-0.78	0.43658
SNAPC5	0.06	7.44	0.78	0.43663
N6AMT1	-0.08	6.13	-0.78	0.43667
DIPK1B	-0.11	5.94	-0.78	0.43679
FKTN	-0.07	6.00	-0.78	0.43680
PSMD12	-0.06	7.69	-0.78	0.43682
NFRKB	-0.05	9.55	-0.78	0.43684
RTKN2	-0.10	5.73	-0.78	0.43686
CCNI	0.07	12.09	0.78	0.43696
GDPD5	0.09	9.45	0.78	0.43713
CCDC96	0.07	5.70	0.78	0.43718
P2RY11	0.09	6.83	0.78	0.43720
LIMA1	-0.08	6.88	-0.78	0.43726
TAL1	0.16	8.72	0.78	0.43726
GLA	-0.06	8.27	-0.78	0.43730
NGRN	-0.07	7.19	-0.78	0.43732
AC010883.1	-0.09	5.49	-0.78	0.43752
AVPI1	0.10	5.57	0.78	0.43777
ATP6V0A2	-0.06	7.70	-0.78	0.43783
NDUFAF5	-0.09	6.19	-0.78	0.43804
RPS27AP11	0.09	6.33	0.78	0.43812
RPS6P25	0.11	7.24	0.78	0.43816
TOMM20P4	-0.10	5.07	-0.78	0.43852
PPIL2	0.04	10.03	0.78	0.43881
ATP1A1	0.05	12.24	0.78	0.43916
ISG20L2	0.04	9.77	0.78	0.43924
USP54	-0.07	5.87	-0.78	0.43937
NFAT5	-0.10	7.02	-0.78	0.43939
AP002414.4	0.08	5.66	0.78	0.43946

CHROMR	-0.08	5.91	-0.78	0.43959
RNF121	0.06	8.01	0.78	0.43973
PTK2B	-0.07	12.51	-0.78	0.44002
RBM8A	0.06	10.27	0.78	0.44005
WHAMMP3	-0.09	6.26	-0.78	0.44017
KCMF1	-0.05	9.61	-0.78	0.44019
ARMCX4	-0.09	5.87	-0.78	0.44038
NCR1	0.11	7.80	0.78	0.44040
DGCR8	-0.06	9.09	-0.78	0.44050
PUS3	0.06	6.89	0.78	0.44052
KCTD7	-0.07	8.04	-0.78	0.44054
SSR4P1	0.08	5.38	0.78	0.44056
LRRC46	-0.08	5.40	-0.78	0.44058
SPSB1	0.09	6.34	0.78	0.44071
LARP1	-0.04	10.56	-0.78	0.44071
SYTL4	-0.10	5.72	-0.78	0.44074
ZNF770	-0.08	7.58	-0.78	0.44080
D2HGDH	0.11	8.54	0.78	0.44091
NFAM1	-0.10	12.97	-0.78	0.44095
LANCL2	-0.07	7.12	-0.78	0.44102
TOLLIP-AS1	-0.09	5.13	-0.78	0.44107
BICDL1	0.08	8.70	0.78	0.44129
TEX22	-0.09	5.09	-0.78	0.44133
AC026471.4	0.08	5.42	0.78	0.44162
ARHGEF35-AS1	0.10	5.70	0.78	0.44165
TPRN	0.09	7.14	0.78	0.44165
DAGLA	-0.10	6.36	-0.78	0.44167
TMEM38A	0.09	6.52	0.78	0.44170
TUBB2A	-0.28	6.86	-0.78	0.44175
WDR5	0.05	8.85	0.78	0.44178
PEAR1	-0.12	6.92	-0.78	0.44181
GPR146	0.14	8.29	0.77	0.44197
AC016773.2	0.11	5.38	0.77	0.44210
ST20	-0.13	7.15	-0.77	0.44228
SNRPCP2	0.08	5.31	0.77	0.44229
AL049873.1	0.13	9.59	0.77	0.44237
RRAGA	0.04	9.47	0.77	0.44243
FAAHP1	0.11	6.08	0.77	0.44245
ATP6V1E1P1	0.08	5.60	0.77	0.44248
ZNF2	-0.06	6.37	-0.77	0.44259
CTSB	0.09	13.06	0.77	0.44296
WRN	-0.08	6.50	-0.77	0.44305
ETV6	-0.07	10.06	-0.77	0.44313
DDX6	-0.07	10.63	-0.77	0.44330
MIR9-3HG	0.10	6.47	0.77	0.44337
THEMIS2	-0.08	12.65	-0.77	0.44338
CFAP36	0.07	7.44	0.77	0.44345
DENND6B	0.09	7.68	0.77	0.44346
CRISP3	-0.17	5.75	-0.77	0.44346
JDP2	-0.10	9.31	-0.77	0.44352

BORA	-0.07	6.02	-0.77	0.44353
TRPV4	-0.10	5.29	-0.77	0.44358
SLC4A1	0.25	12.09	0.77	0.44358
TTPAL	-0.06	7.83	-0.77	0.44367
IL1R2	-0.21	9.94	-0.77	0.44369
RTL8A	0.07	7.78	0.77	0.44371
C14orf93	0.05	8.23	0.77	0.44381
AC069281.2	-0.13	6.94	-0.77	0.44384
CDC42SE2	-0.06	10.95	-0.77	0.44427
C15orf61	0.07	6.67	0.77	0.44438
SMIM3	-0.09	8.13	-0.77	0.44472
EHBP1L1	-0.07	12.34	-0.77	0.44475
LINC00342	-0.11	6.99	-0.77	0.44480
BTF3P10	0.10	6.18	0.77	0.44491
L2HGDH	0.08	5.27	0.77	0.44502
MEI1	0.08	8.61	0.77	0.44503
EIF4G1	-0.05	11.47	-0.77	0.44504
MTATP6P1	-0.06	12.17	-0.77	0.44506
SDAD1P1	0.06	5.26	0.77	0.44511
SLC9A8	-0.11	10.00	-0.77	0.44515
INVS	-0.06	6.71	-0.77	0.44519
EEF1A1P38	0.06	5.17	0.77	0.44532
ANK3	-0.10	6.29	-0.77	0.44538
AL353807.5	-0.09	6.56	-0.77	0.44554
MTBP	-0.08	5.01	-0.77	0.44560
ACOT4	0.09	5.48	0.77	0.44585
AC130456.3	0.19	9.50	0.77	0.44599
AP1S1	0.07	7.79	0.77	0.44602
IRF2BP2	-0.05	10.16	-0.77	0.44610
CD1C	0.09	8.02	0.77	0.44615
AC093297.2	-0.08	5.85	-0.77	0.44622
PSTK	0.07	5.64	0.77	0.44629
MRAS	-0.13	6.51	-0.77	0.44631
AL035411.1	-0.09	5.30	-0.77	0.44640
ZNF559	-0.08	7.01	-0.77	0.44642
OS9	0.07	12.94	0.77	0.44694
ZNF35	0.08	6.19	0.77	0.44704
POMP	0.07	8.59	0.77	0.44712
AC092718.4	0.08	5.19	0.77	0.44714
FAAH	0.11	7.70	0.77	0.44714
KIAA1586	-0.08	5.63	-0.77	0.44714
AF131215.6	0.09	6.45	0.77	0.44719
IPPK	-0.05	6.90	-0.77	0.44719
ZNF385A	0.11	11.05	0.77	0.44725
POLE	-0.05	9.14	-0.77	0.44734
SMYD3	0.07	6.89	0.77	0.44736
SP110	-0.09	11.06	-0.77	0.44740
AC026412.1	0.08	5.73	0.77	0.44753
SLC25A29	0.15	8.65	0.77	0.44759
KLHL28	-0.08	6.64	-0.76	0.44783

AC092747.4	-0.07	6.04	-0.76	0.44797
TC2N	-0.09	8.34	-0.76	0.44802
DCAF1	-0.06	8.54	-0.76	0.44819
AC083862.3	-0.11	6.36	-0.76	0.44831
IGLV7-46	0.18	6.23	0.76	0.44834
GIT1	0.06	10.19	0.76	0.44836
ARHGAP30	0.05	13.10	0.76	0.44851
PRELID1	0.08	12.08	0.76	0.44861
TPMT	0.07	7.51	0.76	0.44869
ADSL	0.07	6.06	0.76	0.44891
HROB	-0.14	5.08	-0.76	0.44903
IGLJ2	0.12	5.35	0.76	0.44922
UBTD1	0.10	7.74	0.76	0.44926
NSMCE2	0.10	7.36	0.76	0.44928
IST1	-0.04	11.51	-0.76	0.44930
LIN7C	-0.05	6.96	-0.76	0.44939
AC132942.1	0.06	5.12	0.76	0.44940
ASPRV1	-0.12	7.97	-0.76	0.44944
TMTC4	-0.08	6.13	-0.76	0.44947
SIGLEC8	0.15	6.27	0.76	0.44970
FAM71F2	-0.13	6.15	-0.76	0.44980
AC007342.9	0.11	6.01	0.76	0.44988
ZDHHC23	-0.08	5.52	-0.76	0.45000
CD200	0.12	5.78	0.76	0.45009
EIF2A	0.05	9.35	0.76	0.45010
LTA4H	-0.07	11.34	-0.76	0.45012
IGHG3	0.25	9.55	0.76	0.45021
GNPDA2	-0.07	5.98	-0.76	0.45050
IGKV1D-13	-0.19	5.35	-0.76	0.45057
AGPAT3	-0.06	10.39	-0.76	0.45064
MTSS2	-0.08	5.57	-0.76	0.45076
SLC2A6	0.10	9.33	0.76	0.45079
ALG5	-0.07	7.35	-0.76	0.45079
LINC01138	-0.08	7.17	-0.76	0.45098
ADCY6	-0.10	5.03	-0.76	0.45099
ATP5MC2P4	0.08	6.89	0.76	0.45113
EIF1AY	-0.39	7.63	-0.76	0.45115
AC100810.1	0.12	8.23	0.76	0.45115
RSPH3	-0.08	6.98	-0.76	0.45122
BORCS7	0.08	7.52	0.76	0.45127
EVC2	-0.09	5.14	-0.76	0.45133
SGSM2	0.07	9.71	0.76	0.45139
NIBAN3	0.14	9.21	0.76	0.45143
PLEKHN1	0.08	5.24	0.76	0.45143
ANP32BP1	0.10	6.90	0.76	0.45149
MMP17	0.13	6.80	0.76	0.45151
TXNL1	0.05	8.97	0.76	0.45164
ANO7L1	0.13	5.35	0.76	0.45166
IGKV1-6	0.15	5.96	0.76	0.45172
ADGRB1	0.09	5.39	0.76	0.45191

ABCD4	0.05	9.09	0.76	0.45210
EDRF1	-0.07	7.65	-0.76	0.45244
COX20	-0.05	8.20	-0.76	0.45256
HCG11	-0.07	7.26	-0.76	0.45260
TNFAIP8	0.05	10.02	0.76	0.45260
TUBB6	0.14	7.46	0.76	0.45272
RALBP1	0.10	10.79	0.76	0.45274
IFT57	0.08	7.34	0.76	0.45276
ADRB2	-0.09	8.67	-0.76	0.45300
RGS14	0.08	11.71	0.76	0.45302
BLOC1S5	0.07	7.46	0.76	0.45309
AL049834.1	0.08	5.27	0.76	0.45320
NSUN7	-0.14	6.44	-0.76	0.45330
SCLY	-0.09	5.37	-0.76	0.45331
POMZP3	0.13	7.03	0.76	0.45336
CBR1	-0.08	8.57	-0.76	0.45343
NCK1-DT	0.07	6.03	0.76	0.45347
RABL2B	0.05	8.05	0.75	0.45372
DNAJB12	0.05	10.20	0.75	0.45377
HGF	-0.08	5.82	-0.75	0.45408
TXNDC9	-0.07	7.07	-0.75	0.45413
AL669831.1	-0.13	8.05	-0.75	0.45418
AL034550.2	0.09	5.67	0.75	0.45431
ZBTB8OSP2	-0.06	5.00	-0.75	0.45432
EZR	0.06	11.54	0.75	0.45454
SLF1	0.10	7.30	0.75	0.45482
BAHCC1	-0.09	6.12	-0.75	0.45485
ZNF561	-0.06	7.45	-0.75	0.45497
NAP1L5	0.07	5.06	0.75	0.45498
SWAP70	-0.09	8.31	-0.75	0.45501
SMTN	0.07	6.41	0.75	0.45523
KRT5	-0.13	5.62	-0.75	0.45531
CDC42BPG	0.07	6.77	0.75	0.45538
PRR5L	-0.09	9.13	-0.75	0.45544
MT-CO2	0.07	15.13	0.75	0.45553
PYGL	-0.13	11.68	-0.75	0.45555
FADS2	0.21	8.17	0.75	0.45555
SEMA4D	-0.06	12.68	-0.75	0.45559
SSBP2	-0.08	7.43	-0.75	0.45567
CRIP1P2	0.09	5.19	0.75	0.45582
IL2RB	-0.09	11.58	-0.75	0.45586
CCT5P1	-0.06	5.27	-0.75	0.45591
IMPA1	-0.09	6.54	-0.75	0.45596
ZNF696	0.06	7.03	0.75	0.45603
NME2P1	0.10	6.20	0.75	0.45608
RGPD2	0.10	5.39	0.75	0.45629
PEX26	0.04	8.87	0.75	0.45647
AL158050.1	-0.08	5.32	-0.75	0.45661
PSMD2	0.04	10.82	0.75	0.45679
CXCR6	0.11	7.34	0.75	0.45708

CLCN5	-0.08	6.46	-0.75	0.45722
DNM1P47	-0.10	5.06	-0.75	0.45725
PTS	0.07	5.65	0.75	0.45727
BCL3	0.12	10.80	0.75	0.45740
CCDC163	-0.12	5.59	-0.75	0.45748
RPL21P44	0.13	5.85	0.75	0.45749
FCER1A	-0.15	8.66	-0.75	0.45753
ITGB2-AS1	0.12	10.75	0.75	0.45772
MCOLN1	0.10	9.53	0.75	0.45786
RCC2	-0.05	10.62	-0.75	0.45794
RPL30P14	0.13	7.50	0.75	0.45801
TMEM126A	0.06	7.02	0.75	0.45828
CHRNE	-0.11	6.47	-0.75	0.45828
FAM167A	0.14	5.73	0.75	0.45829
C16orf91	0.08	6.94	0.75	0.45838
ORC3	-0.06	6.97	-0.75	0.45844
GTF2H2	-0.11	5.72	-0.75	0.45846
SLTM	0.06	10.30	0.75	0.45846
ZFP92	0.07	5.98	0.75	0.45847
MILR1	-0.10	7.13	-0.75	0.45848
RIC8A	0.04	11.03	0.75	0.45858
SEPTIN7P7	-0.09	4.95	-0.75	0.45859
RPL7AP66	0.10	6.98	0.75	0.45861
F8A2	-0.07	4.99	-0.75	0.45867
HEXB	-0.06	9.93	-0.75	0.45872
RNF7	0.05	8.26	0.75	0.45880
SCAP	0.05	10.89	0.75	0.45891
SPEF2	-0.07	5.85	-0.75	0.45892
MTND5P11	-0.07	5.86	-0.75	0.45899
DNAJC24	-0.08	6.26	-0.75	0.45901
PRR34-AS1	0.09	5.70	0.75	0.45920
LY86	0.11	9.35	0.75	0.45922
GLMP	0.09	8.92	0.75	0.45929
SLC27A1	0.08	8.58	0.75	0.45946
METTL22	-0.06	7.91	-0.75	0.45953
RPL13AP3	0.06	5.50	0.74	0.45966
ZNF384	-0.04	9.74	-0.74	0.45969
NADK2	-0.06	6.45	-0.74	0.45999
RPS18P12	0.10	6.90	0.74	0.46003
CLSTN3	0.08	10.18	0.74	0.46015
MARCHF5	-0.08	7.41	-0.74	0.46021
ATP10A	-0.07	8.92	-0.74	0.46025
IGFBP2	0.17	5.09	0.74	0.46063
SDHAF4	-0.07	5.39	-0.74	0.46065
TMEM218	-0.06	6.93	-0.74	0.46072
AC008481.1	-0.08	5.80	-0.74	0.46104
AURKA	-0.08	5.71	-0.74	0.46108
TCEA3	0.13	7.29	0.74	0.46121
PARP3	0.07	7.53	0.74	0.46132
XYLB	-0.09	4.94	-0.74	0.46140

AC096992.2	-0.08	5.03	-0.74	0.46158
AC145207.5	0.06	6.13	0.74	0.46182
COX7A2L	0.06	9.51	0.74	0.46193
CCDC69	0.05	11.67	0.74	0.46195
ILF3	0.05	11.78	0.74	0.46200
DESI1	0.04	9.05	0.74	0.46206
DDOST	0.05	10.86	0.74	0.46209
FAM83H	0.07	6.07	0.74	0.46213
SAAL1	0.06	6.37	0.74	0.46235
SESN3	0.09	9.17	0.74	0.46244
MPO	0.21	8.16	0.74	0.46247
LEXM	-0.10	5.33	-0.74	0.46255
AC006205.2	0.15	5.14	0.74	0.46255
MZB1	0.16	8.22	0.74	0.46257
GHRLOS	0.11	5.47	0.74	0.46271
CLEC11A	-0.10	6.36	-0.74	0.46284
ZNF547	-0.08	5.26	-0.74	0.46298
IGLV3-10	0.19	6.04	0.74	0.46301
KLHDC2	-0.06	8.30	-0.74	0.46316
AL356535.1	0.10	6.37	0.74	0.46319
OSBP2	0.19	9.13	0.74	0.46329
CASP17P	-0.09	6.32	-0.74	0.46331
WASHC2C	-0.05	9.29	-0.74	0.46336
TNFSF13	-0.08	7.14	-0.74	0.46353
STOM	-0.09	10.17	-0.74	0.46355
TFDP2	0.07	8.76	0.74	0.46368
TMEM19	0.07	7.29	0.74	0.46378
COQ10B	-0.06	8.37	-0.74	0.46388
LINC02352	-0.10	6.68	-0.74	0.46392
CCRL2	-0.07	6.59	-0.74	0.46394
PLCG1	0.08	10.94	0.74	0.46395
BBS9	0.05	6.88	0.74	0.46421
NTN5	0.09	5.14	0.74	0.46422
THOC7	0.08	8.16	0.74	0.46427
BAIAP2	0.08	7.54	0.74	0.46428
KARS1	0.06	10.97	0.74	0.46429
CCT6P2	-0.08	5.64	-0.74	0.46439
THUMPD2	-0.09	6.12	-0.74	0.46445
AP000942.1	0.07	5.40	0.74	0.46451
PTTG1	0.09	6.48	0.74	0.46465
ZNF280D	-0.07	6.25	-0.74	0.46468
MTA3	0.06	7.32	0.74	0.46474
SRRM2	-0.07	13.63	-0.74	0.46477
RNF166	-0.07	11.29	-0.74	0.46478
FRMD8	0.05	10.11	0.74	0.46478
PYROXD2	-0.06	7.52	-0.74	0.46478
TMEM8B	0.06	7.40	0.74	0.46481
PAX8	-0.16	5.49	-0.74	0.46482
PRKCH	-0.06	10.35	-0.74	0.46512
RO60	-0.07	7.82	-0.74	0.46518

DBNL	0.06	12.22	0.74	0.46541
BAHD1	0.04	9.39	0.74	0.46551
SPX	0.10	5.39	0.74	0.46562
OSCAR	0.11	10.63	0.73	0.46571
UQCRBP1	0.10	6.71	0.73	0.46571
EIF2S2P3	0.08	5.13	0.73	0.46607
TRDC	-0.14	8.65	-0.73	0.46610
CCDC125	-0.08	7.82	-0.73	0.46622
ALDOAP2	0.09	7.00	0.73	0.46624
IRGQ	0.06	7.50	0.73	0.46628
AC012467.2	0.07	5.96	0.73	0.46640
PEX10	0.06	7.38	0.73	0.46651
CDHR1	0.09	6.10	0.73	0.46651
OGG1	0.06	8.70	0.73	0.46674
MAFG	0.08	8.86	0.73	0.46678
UBE2C	-0.09	5.80	-0.73	0.46691
THEM5	0.12	5.02	0.73	0.46693
ATP5F1CP1	-0.09	5.82	-0.73	0.46695
DTYMK	0.09	7.86	0.73	0.46696
DPY19L2P2	-0.08	5.13	-0.73	0.46696
HCFC2	-0.07	6.24	-0.73	0.46704
AL512378.1	0.14	5.28	0.73	0.46710
GPR82	-0.08	5.29	-0.73	0.46726
HSPA1A	-0.09	10.81	-0.73	0.46764
CNR2	0.10	7.61	0.73	0.46765
RPL41P5	0.10	8.37	0.73	0.46766
ANKRD11	0.05	11.05	0.73	0.46770
BCL11B	0.08	9.99	0.73	0.46773
TCFL5	-0.05	7.10	-0.73	0.46775
RAB20	-0.13	7.77	-0.73	0.46798
RFX2	-0.13	9.13	-0.73	0.46808
MYH9	-0.07	14.87	-0.73	0.46810
AL450998.2	-0.07	6.67	-0.73	0.46812
AL008727.1	0.09	5.00	0.73	0.46819
SHCBP1	-0.08	5.34	-0.73	0.46840
VSIG1	0.09	6.59	0.73	0.46847
GALNS	-0.07	9.11	-0.73	0.46871
TRAPPC12	0.05	9.71	0.73	0.46889
IER2	0.06	11.45	0.73	0.46894
CCT5	-0.04	10.59	-0.73	0.46897
LINC00173	-0.14	7.75	-0.73	0.46918
TOGARAM2	-0.10	6.78	-0.73	0.46921
CLEC2L	0.13	5.70	0.73	0.46930
ANKRD13D	0.08	11.81	0.73	0.46934
AL139220.2	0.14	6.19	0.73	0.46943
FUNDC2	0.08	9.48	0.73	0.46981
AL513320.1	0.08	5.65	0.73	0.46983
MRPL30	-0.06	7.83	-0.73	0.46989
FAHD2A	0.07	7.45	0.73	0.46991
MARVELD1	-0.09	7.99	-0.73	0.46996

GSTM1	-0.31	6.49	-0.73	0.47002
TSPAN32	0.07	9.55	0.73	0.47010
UBAP2L	-0.04	10.59	-0.73	0.47012
ATP5MC2P1	0.08	5.41	0.73	0.47027
STYXL1	0.07	8.47	0.73	0.47048
RAB33A	0.10	6.85	0.73	0.47053
C14orf119	0.04	9.24	0.73	0.47088
FANCC	-0.07	6.18	-0.73	0.47090
ULK4	0.07	6.11	0.73	0.47092
TOX4P1	-0.09	5.24	-0.73	0.47099
MED10	0.05	8.85	0.73	0.47101
DAB2IP	-0.09	4.93	-0.73	0.47122
PNP	0.07	10.21	0.73	0.47126
PLAAT3	0.10	7.44	0.73	0.47126
MST1	-0.08	5.97	-0.73	0.47134
RBM41	0.07	6.21	0.73	0.47135
ACP2	0.07	8.99	0.73	0.47139
VSIG4	-0.17	6.53	-0.73	0.47158
ZNF141	-0.08	6.96	-0.72	0.47177
LINC00339	-0.08	6.70	-0.72	0.47194
BRF2	0.04	7.83	0.72	0.47195
HSBP1L1	0.09	6.51	0.72	0.47207
PPP5C	0.05	9.00	0.72	0.47231
C16orf70	-0.05	8.76	-0.72	0.47239
ATRAID	0.06	9.69	0.72	0.47241
RBX1	0.06	9.31	0.72	0.47261
UBE2K	-0.05	8.61	-0.72	0.47262
DDB2	-0.06	8.62	-0.72	0.47268
ECE1	-0.09	11.94	-0.72	0.47274
HOXC4	0.10	5.54	0.72	0.47280
NAIF1	0.04	8.05	0.72	0.47297
LARS2	-0.05	7.69	-0.72	0.47308
EEPD1	0.08	9.33	0.72	0.47310
TAF13	-0.06	6.48	-0.72	0.47311
SP140	-0.07	9.51	-0.72	0.47332
BTF3P12	0.07	5.61	0.72	0.47333
ZCCHC18	0.07	5.51	0.72	0.47333
BANP	0.05	9.29	0.72	0.47334
MT-CO3	0.07	15.10	0.72	0.47340
LARGE2	0.09	6.97	0.72	0.47355
APBB1IP	-0.08	11.95	-0.72	0.47362
RRAS2	-0.08	6.45	-0.72	0.47364
ZBTB22	0.05	8.61	0.72	0.47366
LAT2	-0.08	11.74	-0.72	0.47397
PERP	0.07	5.62	0.72	0.47398
SLC39A11	-0.06	7.92	-0.72	0.47399
USP30-AS1	0.09	6.25	0.72	0.47407
MMP8	-0.19	6.06	-0.72	0.47409
SLC46A1	-0.08	6.29	-0.72	0.47413
UBC	0.06	14.08	0.72	0.47424

EWSR1	0.04	11.55	0.72	0.47424
ZNF32	0.07	8.04	0.72	0.47441
TM2D1	0.05	7.11	0.72	0.47450
AC073264.3	-0.09	4.93	-0.72	0.47455
TDP1	0.06	8.27	0.72	0.47456
SNX29	-0.05	9.50	-0.72	0.47478
NKIRAS2	0.05	9.59	0.72	0.47485
AL360181.2	-0.09	5.28	-0.72	0.47494
MICAL2	-0.12	10.84	-0.72	0.47501
TG	-0.10	5.02	-0.72	0.47512
LINC02680	0.11	5.05	0.72	0.47513
BATF3	-0.10	6.73	-0.72	0.47531
NAA20	0.05	7.69	0.72	0.47540
AC098614.3	-0.08	5.63	-0.72	0.47552
HMGN2P3	0.09	6.47	0.72	0.47555
ZCCHC3	0.03	9.07	0.72	0.47567
BOK	-0.15	5.98	-0.72	0.47571
CD14	0.10	13.35	0.72	0.47584
PSAP	-0.07	15.67	-0.72	0.47593
PLCD1	0.05	8.63	0.72	0.47595
MAK16	-0.06	6.87	-0.72	0.47595
SYNJ2BP	-0.06	6.78	-0.72	0.47596
AL513343.1	0.09	5.16	0.72	0.47601
MARS1	-0.05	10.88	-0.72	0.47615
LYPD2	-0.21	6.70	-0.72	0.47632
SETDB1	-0.03	9.95	-0.72	0.47638
UBA52P5	0.11	8.11	0.72	0.47659
CLN3	-0.06	7.00	-0.72	0.47662
ORC4	-0.06	7.13	-0.72	0.47693
TP53INP2	-0.12	8.21	-0.72	0.47702
YWHAB	0.06	12.64	0.72	0.47706
TNPO3	-0.05	9.81	-0.72	0.47708
RPSAP8	0.07	5.48	0.72	0.47711
AC124944.2	-0.09	4.95	-0.72	0.47712
RPS17P2	-0.11	6.10	-0.72	0.47731
OPA3	0.05	8.22	0.72	0.47754
DDX18	-0.06	9.23	-0.72	0.47765
RBM45	-0.07	6.32	-0.72	0.47771
AC111170.4	0.11	5.92	0.72	0.47775
TEX10	0.06	7.40	0.72	0.47775
PSMA5	0.06	9.51	0.72	0.47776
TMEM214	0.04	10.27	0.71	0.47788
LRRC3	0.09	5.88	0.71	0.47790
TIMM8A	-0.07	5.74	-0.71	0.47809
LTBP1	-0.13	6.61	-0.71	0.47845
AC090517.5	-0.08	4.96	-0.71	0.47870
LDHD	0.10	5.61	0.71	0.47885
HOTAIRM1	-0.11	6.43	-0.71	0.47892
KPTN	-0.09	7.38	-0.71	0.47897
HLTF	-0.08	6.21	-0.71	0.47915

COPS4	0.06	8.02	0.71	0.47942
SLC16A3	0.09	11.97	0.71	0.47943
HLA-S	0.09	5.20	0.71	0.47946
APMAP	-0.11	11.91	-0.71	0.47953
C1QB	-0.17	6.38	-0.71	0.47961
UTY	-0.28	6.81	-0.71	0.47969
RPS4XP14	0.10	6.28	0.71	0.47987
MAP1LC3B	-0.07	10.44	-0.71	0.47988
AC103988.1	-0.07	5.39	-0.71	0.48026
RNF11	-0.12	9.33	-0.71	0.48028
LRFN1	0.08	8.52	0.71	0.48028
TRBV12-5	0.10	5.19	0.71	0.48031
ZDHHC24	0.05	8.07	0.71	0.48032
AC096677.1	0.07	5.97	0.71	0.48034
NFX1	0.05	9.19	0.71	0.48038
DOK3	-0.11	12.20	-0.71	0.48047
C12orf73	0.07	6.03	0.71	0.48057
RPL31P11	0.06	5.25	0.71	0.48069
IGLC2	0.21	11.35	0.71	0.48096
SNX3	-0.08	10.34	-0.71	0.48104
PDE6D	0.05	8.04	0.71	0.48107
BNIPL	-0.09	5.61	-0.71	0.48112
CLK3	0.04	10.49	0.71	0.48129
TRAV38-2DV8	0.10	6.12	0.71	0.48137
AC006942.1	0.09	5.07	0.71	0.48164
ALDOAP1	0.06	6.41	0.71	0.48190
TWNK	0.07	7.62	0.71	0.48191
PDIA3P1	-0.09	7.58	-0.71	0.48201
RAMAC	-0.07	7.57	-0.71	0.48204
KLF3-AS1	-0.10	5.50	-0.71	0.48216
AL022328.4	0.09	5.53	0.71	0.48249
NSFL1C	0.07	10.62	0.71	0.48255
PIGS	0.04	10.21	0.71	0.48284
ANK1	0.17	9.12	0.71	0.48292
MTND2P28	-0.11	10.32	-0.71	0.48302
TMEM259	0.06	11.38	0.71	0.48312
TBC1D20	-0.03	9.36	-0.71	0.48318
HLA-DRB5	-0.19	11.53	-0.71	0.48327
KIR2DL1	-0.14	6.63	-0.71	0.48331
LINC00954	0.13	6.60	0.71	0.48332
AL158071.3	0.11	5.27	0.71	0.48336
ARRB1	-0.06	10.69	-0.71	0.48347
IARS1	-0.06	8.65	-0.71	0.48350
CLIP2	0.06	8.55	0.71	0.48358
HILPDA	0.08	5.54	0.71	0.48386
PNKD	0.07	9.79	0.71	0.48387
AC020898.1	0.08	5.96	0.71	0.48388
CYP2D7	-0.11	6.88	-0.71	0.48391
RELT	-0.08	10.07	-0.70	0.48400
CARF	-0.07	5.71	-0.70	0.48402

FBXL13	-0.10	5.60	-0.70	0.48402
STAMBPL1	0.07	6.99	0.70	0.48414
HACL1	-0.05	7.76	-0.70	0.48415
ZNF260	-0.07	6.27	-0.70	0.48426
KCTD10	-0.05	9.00	-0.70	0.48438
SNHG20	0.07	6.47	0.70	0.48462
NDC1	0.06	6.82	0.70	0.48470
ARL13B	-0.07	5.46	-0.70	0.48497
IGHG4	0.20	8.35	0.70	0.48499
COA6-AS1	-0.09	5.39	-0.70	0.48505
CDH23	-0.07	9.49	-0.70	0.48510
COL18A1	0.16	9.39	0.70	0.48516
ITSN1	-0.10	6.55	-0.70	0.48531
MAN2B2	-0.05	10.45	-0.70	0.48540
DDB1	0.04	11.34	0.70	0.48566
PISD	-0.10	11.32	-0.70	0.48571
CRADD	-0.06	6.67	-0.70	0.48572
MTFR2	-0.08	4.98	-0.70	0.48572
CCDC6	0.05	8.18	0.70	0.48604
TBL2	-0.05	7.97	-0.70	0.48608
RFX7	-0.08	7.04	-0.70	0.48614
LBX2-AS1	0.09	6.33	0.70	0.48624
CABP4	-0.07	5.20	-0.70	0.48634
LYRM2	0.06	8.07	0.70	0.48650
RCCD1	0.07	6.22	0.70	0.48679
ZNF623	-0.05	7.37	-0.70	0.48703
MAP11	-0.08	10.12	-0.70	0.48727
CEBPE	0.11	7.32	0.70	0.48753
AP001372.2	0.07	5.55	0.70	0.48756
MYNN	-0.06	6.92	-0.70	0.48769
UBE2B	-0.09	9.48	-0.70	0.48770
ABHD17AP3	-0.06	5.12	-0.70	0.48785
RMI2	-0.09	5.63	-0.70	0.48795
AC243960.1	0.08	7.98	0.70	0.48797
NCAPD2P1	-0.08	5.03	-0.70	0.48809
TRMT112P6	0.08	6.32	0.70	0.48831
MAPKAPK5-AS1	0.06	7.80	0.70	0.48836
SLC44A1	-0.06	8.07	-0.70	0.48838
BDH2	-0.06	6.08	-0.70	0.48840
RAB28	-0.05	6.85	-0.70	0.48841
RNF145	-0.05	9.86	-0.70	0.48843
SS18L2	0.06	8.44	0.70	0.48855
STARD5	-0.07	6.07	-0.70	0.48857
RNF115	0.05	8.52	0.70	0.48863
TNFRSF21	-0.11	5.27	-0.70	0.48868
WTAP	-0.05	10.19	-0.70	0.48910
NOL11	-0.06	7.92	-0.70	0.48928
AC104964.4	-0.08	4.98	-0.70	0.48933
RRP12	-0.09	10.35	-0.70	0.48943
SFXN3	-0.05	9.12	-0.70	0.48957

LINC00680	0.09	6.29	0.70	0.48965
DBF4B	-0.08	6.58	-0.70	0.48965
FUT4	-0.06	6.79	-0.70	0.48970
ADCY3	0.06	8.14	0.70	0.49008
AP1G2	0.06	11.45	0.69	0.49040
TMEM53	0.07	6.35	0.69	0.49064
FBF1	0.06	6.76	0.69	0.49081
C5orf24	-0.05	6.87	-0.69	0.49086
NAP1L4P1	-0.07	5.25	-0.69	0.49089
CNOT3	0.05	10.50	0.69	0.49095
CIITA	-0.09	10.31	-0.69	0.49097
TARBP1	-0.07	7.54	-0.69	0.49105
VDAC2	0.04	9.10	0.69	0.49130
RPL10P1	0.06	5.57	0.69	0.49150
AL390066.2	0.11	6.27	0.69	0.49152
RPS24P8	0.10	7.30	0.69	0.49170
ZNF703	0.11	7.88	0.69	0.49177
ZNF197	-0.05	6.82	-0.69	0.49178
DXO	0.07	7.89	0.69	0.49178
ZNF101	0.06	9.00	0.69	0.49199
MIS18A	0.07	6.49	0.69	0.49200
RPS6KA2	0.05	7.03	0.69	0.49238
FNTB	0.07	7.19	0.69	0.49242
METTL23	0.06	7.41	0.69	0.49244
ADGRA3	0.10	5.21	0.69	0.49244
GIPR	0.10	6.15	0.69	0.49253
FOXD2-AS1	-0.06	6.90	-0.69	0.49270
AL357079.1	-0.09	5.06	-0.69	0.49274
UBE2N	-0.05	8.96	-0.69	0.49277
PAICS	0.06	7.77	0.69	0.49281
DHRS13	0.12	9.05	0.69	0.49327
CACNA2D4	0.06	8.18	0.69	0.49350
AC016394.4	-0.08	5.08	-0.69	0.49350
RNF157	0.09	8.82	0.69	0.49360
MSANTD2	-0.07	6.14	-0.69	0.49370
C9orf85	0.07	6.86	0.69	0.49372
RPP25	0.07	6.95	0.69	0.49399
IGKV1-8	-0.12	5.88	-0.69	0.49403
AC005480.2	0.06	5.11	0.69	0.49417
CACNB1	0.07	7.04	0.69	0.49427
NELFCD	0.04	9.81	0.69	0.49427
CTLA4	0.10	6.41	0.69	0.49431
STYX	-0.07	7.50	-0.69	0.49438
ZNF346	0.06	7.96	0.69	0.49440
AC242988.2	0.09	5.77	0.69	0.49443
CDKN2B	0.08	5.40	0.69	0.49461
ZSCAN21	0.06	7.00	0.69	0.49461
BUD13	0.04	8.44	0.69	0.49466
PLXND1	-0.07	10.66	-0.69	0.49487
SREBF1	-0.06	9.80	-0.69	0.49494

LINC01801	0.08	5.10	0.69	0.49508
RAPGEFL1	0.11	7.32	0.69	0.49534
RNF144A	-0.08	7.51	-0.69	0.49550
P2RY6	-0.14	5.81	-0.69	0.49556
OAZ2	-0.07	11.43	-0.69	0.49574
EEF1AKMT2	0.06	5.71	0.69	0.49590
ADGRE5	-0.09	14.56	-0.69	0.49593
GCSAM	0.08	7.28	0.69	0.49603
ISG20	-0.09	11.45	-0.69	0.49617
CAMK1	0.09	8.84	0.69	0.49624
PER3	-0.08	6.09	-0.68	0.49643
EIF4A2P4	-0.11	5.98	-0.68	0.49645
GOLGA2P10	-0.09	5.72	-0.68	0.49660
POMT2	0.08	6.80	0.68	0.49673
CDKN2C	0.06	6.51	0.68	0.49676
FAM86EP	0.08	5.36	0.68	0.49696
PLEKHG1	-0.09	5.97	-0.68	0.49696
SSTR3	0.12	6.86	0.68	0.49710
SLC25A34	0.07	5.49	0.68	0.49737
LINC01215	-0.09	6.74	-0.68	0.49738
FOXRED2	0.07	6.20	0.68	0.49750
ZNF235	-0.06	5.95	-0.68	0.49763
TRIM69	-0.07	9.32	-0.68	0.49784
IGLV4-69	0.16	6.89	0.68	0.49794
ZNF665	0.07	5.50	0.68	0.49797
ZNF669	-0.07	6.04	-0.68	0.49814
ZRANB2	-0.07	8.17	-0.68	0.49851
USP40	0.06	7.75	0.68	0.49885
CLEC4C	-0.11	5.42	-0.68	0.49888
BAG2	0.07	5.91	0.68	0.49903
ZRANB3	-0.06	5.28	-0.68	0.49905
DENND10P1	-0.10	5.44	-0.68	0.49925
CARD8-AS1	-0.06	8.22	-0.68	0.49935
IGHJ3	0.12	6.29	0.68	0.49959
PER2	-0.08	6.79	-0.68	0.49967
IFITM2	-0.14	14.60	-0.68	0.49969
SF3A1	-0.04	11.65	-0.68	0.49989
RARA	-0.08	11.84	-0.68	0.50010
LINC01730	-0.11	5.05	-0.68	0.50023
FAM131A	-0.05	8.05	-0.68	0.50028
PDE1B	0.07	7.59	0.68	0.50031
PEX13	0.06	6.48	0.68	0.50035
AC005261.1	-0.07	6.67	-0.68	0.50037
LCAT	0.08	7.47	0.68	0.50042
LTC4S	0.07	6.07	0.68	0.50044
NPR2	-0.09	5.71	-0.68	0.50054
AC136475.1	-0.12	6.32	-0.68	0.50057
BRD7	-0.04	9.36	-0.68	0.50109
ERP27	-0.09	6.22	-0.68	0.50127
TOGARAM1	-0.08	6.12	-0.68	0.50134

AP002784.2	-0.10	7.89	-0.68	0.50137
COA1	-0.06	8.32	-0.68	0.50139
SUV39H2	-0.06	5.08	-0.68	0.50140
COX6B1P1	0.09	5.93	0.68	0.50155
RAB13	-0.08	5.22	-0.68	0.50161
PLXNA1	0.07	7.54	0.68	0.50162
CCR4	-0.08	7.56	-0.68	0.50174
AC068760.1	0.09	5.14	0.68	0.50177
RPSAP36	0.09	5.16	0.68	0.50178
UBAC1	0.05	9.66	0.68	0.50180
Z97192.3	0.08	5.28	0.68	0.50184
ACTG1P2	0.09	5.63	0.68	0.50188
CITED2	-0.04	9.75	-0.68	0.50189
SCNM1	0.06	9.68	0.68	0.50190
RPL41P1	0.09	7.14	0.68	0.50218
BEX4	0.05	7.80	0.68	0.50220
COX6A1P2	0.09	6.73	0.68	0.50229
USP31	0.05	6.13	0.68	0.50234
BANF1P3	0.07	5.39	0.68	0.50237
LINC02193	-0.09	5.27	-0.68	0.50250
ABHD18	0.07	7.33	0.68	0.50263
GGPS1	0.06	7.44	0.68	0.50267
ARID4A	0.09	8.95	0.68	0.50268
ANXA1	-0.05	11.76	-0.67	0.50278
SKIV2L	0.05	10.25	0.67	0.50287
PGS1	-0.11	10.17	-0.67	0.50327
RHOB	-0.08	10.13	-0.67	0.50337
HOXB4	0.08	6.28	0.67	0.50365
SLC35B1	0.04	8.53	0.67	0.50373
MAPKAPK3	-0.06	11.21	-0.67	0.50379
SLC25A17	-0.06	7.22	-0.67	0.50380
YBX1P1	0.09	8.51	0.67	0.50393
AC055822.1	0.08	5.73	0.67	0.50403
MT1L	-0.09	4.90	-0.67	0.50403
DENND2B	0.09	5.17	0.67	0.50404
ANKRD34A	0.06	5.80	0.67	0.50408
NPRL3	0.13	11.68	0.67	0.50416
SCFD1	-0.05	8.34	-0.67	0.50421
PTCH1	-0.08	7.67	-0.67	0.50423
ARHGDIB	0.06	14.48	0.67	0.50427
YPEL3	0.09	12.81	0.67	0.50438
LUC7L2	0.04	8.08	0.67	0.50441
CCNK	-0.04	9.91	-0.67	0.50462
NR4A2	-0.08	5.50	-0.67	0.50463
LTO1	-0.05	8.14	-0.67	0.50465
TEX30	-0.06	5.84	-0.67	0.50477
FAM151B	0.08	4.99	0.67	0.50484
IGFLR1	-0.09	7.50	-0.67	0.50485
AC241585.1	0.08	5.20	0.67	0.50504
KBTBD3	0.07	5.61	0.67	0.50515

WDR6	0.05	10.94	0.67	0.50524
TLE1	0.08	7.68	0.67	0.50529
GSPT1	0.12	10.73	0.67	0.50530
HIBCH	-0.06	6.36	-0.67	0.50542
RAB32	-0.08	9.23	-0.67	0.50548
TP53	0.05	10.27	0.67	0.50548
H1-0	-0.10	8.72	-0.67	0.50576
TBP	-0.04	7.55	-0.67	0.50584
TRBV12-3	0.09	6.72	0.67	0.50600
POLDIP3	0.03	11.00	0.67	0.50602
BIN3	0.07	9.98	0.67	0.50607
PLOD3	0.05	9.83	0.67	0.50616
DDX19A	0.06	7.88	0.67	0.50618
AC012085.1	-0.08	6.19	-0.67	0.50658
MRPL18	-0.04	8.56	-0.67	0.50665
ASCL2	-0.09	7.30	-0.67	0.50666
NUP37	0.05	6.88	0.67	0.50678
UBBP1	-0.10	5.73	-0.67	0.50692
FDX1	0.07	6.97	0.67	0.50699
MEAF6	0.04	8.88	0.67	0.50703
IL16	0.03	12.63	0.67	0.50709
SLC12A4	-0.05	8.47	-0.67	0.50718
AC022400.8	0.08	7.27	0.67	0.50730
VNN2	-0.13	12.32	-0.67	0.50741
ITGA1	-0.08	5.41	-0.67	0.50760
CABP5	0.09	6.49	0.67	0.50796
MIR320E	0.12	5.53	0.67	0.50797
SIN3A	-0.04	10.27	-0.67	0.50811
COTL1P2	-0.07	5.36	-0.67	0.50813
ZNF66	-0.10	5.16	-0.67	0.50818
ARPC5	-0.06	11.71	-0.67	0.50847
ABHD6	0.05	7.34	0.67	0.50867
KIAA1257	-0.09	6.70	-0.67	0.50876
DNTTIP1	0.07	10.07	0.67	0.50881
PXMP4	0.05	7.56	0.66	0.50909
LRRC59	-0.05	9.48	-0.66	0.50926
NALT1	-0.08	5.42	-0.66	0.50950
LMNTD2	0.09	6.31	0.66	0.50963
LAPTM4B	0.07	6.27	0.66	0.50963
AC022167.2	0.07	6.57	0.66	0.50981
LGALS4	0.07	5.15	0.66	0.50990
CFAP70	-0.08	5.24	-0.66	0.51007
CES1P1	-0.10	5.08	-0.66	0.51028
TRAV12-2	0.12	6.50	0.66	0.51049
RNPS1P1	0.07	6.22	0.66	0.51053
DEGS2	0.10	5.32	0.66	0.51054
KLHDC8B	-0.10	7.88	-0.66	0.51056
CLEC4F	-0.17	5.44	-0.66	0.51063
NIPA1	-0.06	7.05	-0.66	0.51072
STAP2	0.07	5.16	0.66	0.51077

GLYR1	-0.04	10.72	-0.66	0.51088
TRBV25-1	0.10	5.35	0.66	0.51092
WHRN	0.09	6.51	0.66	0.51104
ZNF316	0.06	9.97	0.66	0.51110
MIOS	-0.05	7.79	-0.66	0.51120
ANKAR	0.06	5.37	0.66	0.51120
MTX1	-0.08	8.64	-0.66	0.51130
HBEGF	-0.08	5.51	-0.66	0.51143
ZNF813	-0.06	6.39	-0.66	0.51148
BX890604.2	0.08	6.65	0.66	0.51150
LILRA2	-0.09	12.09	-0.66	0.51155
ADIPOR1P2	-0.13	6.11	-0.66	0.51166
AC007485.2	-0.08	5.61	-0.66	0.51190
AGGF1	-0.05	7.23	-0.66	0.51204
CCDC65	-0.08	6.90	-0.66	0.51206
ALAS1	-0.04	9.28	-0.66	0.51257
FLOT1	0.09	11.83	0.66	0.51271
TRMT61B	0.06	6.40	0.66	0.51326
PARP16	0.05	8.27	0.66	0.51341
RUSC1-AS1	0.07	5.16	0.66	0.51361
AC012569.1	0.08	4.99	0.66	0.51362
PACSIN2	-0.08	11.23	-0.66	0.51374
RPL7AP64	0.11	7.30	0.66	0.51381
SLAMF6	0.06	9.86	0.66	0.51383
COX5A	0.07	9.06	0.66	0.51400
PTP4A2	-0.04	11.48	-0.66	0.51427
ZNF169	-0.07	6.25	-0.66	0.51445
MC1R	-0.09	6.01	-0.66	0.51467
PTRH2	0.05	7.71	0.66	0.51471
ITM2A	-0.09	8.80	-0.66	0.51483
PSMG3	0.08	7.99	0.66	0.51529
APOL6	-0.09	10.41	-0.65	0.51540
TPTEP2	-0.08	6.01	-0.65	0.51545
HDGFL3	-0.09	5.76	-0.65	0.51550
TBCEL	-0.10	6.56	-0.65	0.51554
NMUR1	-0.10	7.87	-0.65	0.51564
RAD51	0.07	5.27	0.65	0.51577
ZMYND15	-0.09	6.70	-0.65	0.51583
PTGES3	-0.04	10.40	-0.65	0.51587
ACTG1P11	0.08	5.60	0.65	0.51594
PRPSAP1	0.04	8.12	0.65	0.51595
ZNF443	-0.06	5.36	-0.65	0.51607
SLC25A14	-0.05	6.36	-0.65	0.51614
TTI2	0.04	7.92	0.65	0.51621
ATP5MC3	0.06	9.50	0.65	0.51627
UBLCP1	-0.06	8.14	-0.65	0.51640
SIRT4	-0.07	5.32	-0.65	0.51640
TTI1	-0.04	8.25	-0.65	0.51650
MIR1244-2	0.08	6.94	0.65	0.51671
PICK1	0.05	8.29	0.65	0.51685

HAPLN3	0.09	8.53	0.65	0.51687
RPS6KA1	0.06	12.78	0.65	0.51703
RPL26L1	0.06	7.45	0.65	0.51718
RNF34	-0.03	9.52	-0.65	0.51719
C1orf74	-0.06	5.50	-0.65	0.51719
SEC61G	0.08	7.54	0.65	0.51719
FTL	0.09	16.24	0.65	0.51722
MSI2	0.05	8.39	0.65	0.51752
RSBN1	0.06	8.05	0.65	0.51762
MEGF6	-0.09	9.05	-0.65	0.51768
ARHGAP15	0.05	10.48	0.65	0.51777
CAMK2N1	0.08	6.13	0.65	0.51778
ERGIC2	0.07	7.96	0.65	0.51808
NT5DC1	-0.04	7.56	-0.65	0.51814
ZNF891	-0.07	5.48	-0.65	0.51825
MDM2	-0.07	8.48	-0.65	0.51847
INHBB	-0.14	5.18	-0.65	0.51853
RBMS1	-0.07	9.68	-0.65	0.51858
PELO	-0.06	8.38	-0.65	0.51861
GBP4	-0.12	9.44	-0.65	0.51862
H3-5	-0.10	7.81	-0.65	0.51884
NIFK	0.05	8.04	0.65	0.51908
ARID1B	-0.06	9.74	-0.65	0.51911
PDE4A	-0.07	8.15	-0.65	0.51914
RPL12P17	0.06	5.37	0.65	0.51919
FP236383.3	-0.07	5.00	-0.65	0.51929
NEFH	0.06	5.72	0.65	0.51930
TRIM27	-0.05	10.58	-0.65	0.51934
CTSE	0.12	5.04	0.65	0.51939
CHTOP	-0.03	10.25	-0.65	0.51947
IRF4	0.08	8.66	0.65	0.51950
LINC02207	-0.14	6.22	-0.65	0.51955
DDX11L2	0.14	6.06	0.65	0.51957
ASAP3	-0.06	5.11	-0.65	0.51958
ZFP37	-0.06	4.99	-0.65	0.51960
TUBG1	0.05	7.20	0.65	0.51978
CEP78	0.08	8.26	0.65	0.51994
AC016394.2	0.06	5.13	0.65	0.52009
HECTD3	0.04	9.72	0.65	0.52013
KYAT3	-0.04	7.47	-0.65	0.52014
AC016876.2	0.07	6.06	0.65	0.52022
STRN4	-0.05	10.77	-0.65	0.52025
EFNB1	0.07	7.37	0.65	0.52065
ACTG1P19	-0.05	5.27	-0.65	0.52072
RAB44	0.06	7.83	0.65	0.52080
ATP5F1EP2	0.09	7.22	0.65	0.52090
MISP3	0.08	6.66	0.65	0.52096
HACD3	-0.05	7.58	-0.65	0.52111
HYOU1	0.04	10.53	0.65	0.52112
PDE5A	-0.08	6.04	-0.65	0.52123

TTBK2	0.06	7.77	0.65	0.52136
COA6	-0.07	6.41	-0.65	0.52142
AL031846.1	-0.07	6.02	-0.65	0.52145
SEMA6A-AS1	-0.09	5.00	-0.65	0.52149
NFE4	-0.12	7.38	-0.65	0.52153
PHC2	-0.11	12.33	-0.65	0.52159
HERC4	-0.07	8.58	-0.65	0.52163
C1orf162	0.06	11.14	0.65	0.52171
IGHV3-64D	0.17	5.76	0.64	0.52184
IGHV3-13	0.13	5.52	0.64	0.52190
IGHV4-55	0.11	5.44	0.64	0.52193
AC011477.1	-0.06	5.75	-0.64	0.52212
SYNCRIP	-0.05	9.10	-0.64	0.52214
ZNF131	-0.05	7.92	-0.64	0.52225
AC110079.1	-0.10	7.51	-0.64	0.52268
VOPP1	0.05	9.69	0.64	0.52270
CDK16	0.04	9.44	0.64	0.52285
ZYX	0.08	14.14	0.64	0.52287
C5AR2	-0.09	10.21	-0.64	0.52313
RBM17P4	0.07	5.31	0.64	0.52329
PGGT1B	-0.05	7.69	-0.64	0.52335
KANTR	0.07	6.34	0.64	0.52364
CHCHD3	-0.05	7.99	-0.64	0.52367
VGLL4	0.04	8.73	0.64	0.52378
CDAN1	0.04	8.19	0.64	0.52379
CHN1	0.08	5.40	0.64	0.52391
BTF3L4	-0.05	7.69	-0.64	0.52395
TSNARE1	0.06	7.69	0.64	0.52405
RPS26P31	0.13	5.76	0.64	0.52409
SEMA7A	0.08	7.92	0.64	0.52416
TRBV5-4	0.09	6.76	0.64	0.52430
CACNA2D3	-0.07	6.97	-0.64	0.52431
AP002807.1	-0.09	8.27	-0.64	0.52443
TMEM14DP	0.07	5.12	0.64	0.52443
NECTIN1	-0.09	8.46	-0.64	0.52446
EIF3LP2	0.07	6.34	0.64	0.52469
ENGASE	-0.09	9.44	-0.64	0.52472
MKRN9P	-0.10	5.76	-0.64	0.52473
HGD	0.09	6.32	0.64	0.52475
RARS1	0.05	8.17	0.64	0.52483
AFAP1	-0.08	7.46	-0.64	0.52492
AL391244.2	0.06	5.50	0.64	0.52500
LSM6	0.05	7.85	0.64	0.52521
RPUSD4	0.04	8.17	0.64	0.52532
AC024619.4	0.08	5.72	0.64	0.52534
TRAV27	-0.07	5.68	-0.64	0.52538
MTCO1P12	-0.08	10.76	-0.64	0.52549
MT-ATP6	0.05	13.85	0.64	0.52549
ZDHHC1	0.08	5.94	0.64	0.52575
ZNF557	0.05	7.06	0.64	0.52580

SLC7A7	-0.06	11.33	-0.64	0.52592
TNFSF13B	-0.11	9.76	-0.64	0.52605
AC010642.2	0.08	6.77	0.64	0.52621
WLS	0.10	9.06	0.64	0.52621
AC020651.2	-0.08	4.93	-0.64	0.52654
CSTF2	0.05	7.48	0.64	0.52657
COMMD3	0.06	6.75	0.64	0.52667
CKLF	-0.08	7.83	-0.64	0.52670
DBT	0.05	7.34	0.64	0.52689
ZNF280C	-0.07	5.02	-0.64	0.52693
AC060766.1	-0.07	5.04	-0.64	0.52714
PTP4A2P2	-0.08	5.69	-0.64	0.52718
ZNF613	0.05	6.47	0.64	0.52723
ZNF646	0.05	9.62	0.64	0.52746
PML	-0.08	10.46	-0.64	0.52747
ARHGAP22	0.08	5.82	0.64	0.52748
AK6	0.06	5.87	0.64	0.52752
LINC02210	-0.06	7.10	-0.64	0.52758
BSPRY	0.07	5.26	0.64	0.52769
TENM1	-0.12	5.49	-0.64	0.52774
AL590550.1	-0.10	4.96	-0.64	0.52790
SLC15A2	-0.09	6.12	-0.64	0.52801
FCN2	0.06	5.31	0.64	0.52803
AC004918.1	-0.07	5.89	-0.64	0.52804
RMRP	-0.09	5.11	-0.64	0.52804
DUSP28	0.05	6.47	0.64	0.52809
COA7	-0.06	6.49	-0.63	0.52827
VPS11	0.03	10.25	0.63	0.52829
HSD17B7	-0.05	7.20	-0.63	0.52838
ANXA2P2	-0.10	8.19	-0.63	0.52846
UBA7	-0.06	11.38	-0.63	0.52846
AMIGO1	0.09	7.97	0.63	0.52846
ENPP2	0.07	5.32	0.63	0.52855
PCSK4	0.09	5.04	0.63	0.52890
PRKY	-0.27	7.46	-0.63	0.52893
RAB27B	-0.08	6.48	-0.63	0.52900
WAS	0.07	13.25	0.63	0.52913
MYEF2	-0.10	5.56	-0.63	0.52941
ALOX12-AS1	0.08	6.77	0.63	0.52941
NPDC1	0.11	7.10	0.63	0.52942
ANXA2P1	-0.06	5.10	-0.63	0.52942
AC009093.2	-0.07	5.12	-0.63	0.52948
IQCB1	0.05	7.77	0.63	0.52965
PLAC8	0.07	10.37	0.63	0.52966
NLRX1	0.06	9.35	0.63	0.52971
SON	-0.06	11.23	-0.63	0.52977
MIEF1	-0.04	9.04	-0.63	0.52983
UBA1	0.04	12.86	0.63	0.52992
ASB16-AS1	0.05	7.76	0.63	0.52992
DNMBP	-0.07	7.40	-0.63	0.52999

ANKLE2	-0.03	9.72	-0.63	0.53000
B3GAT3P1	0.07	5.05	0.63	0.53015
AL161785.1	0.07	7.06	0.63	0.53018
SLC9A9	-0.04	7.86	-0.63	0.53019
RBAK	-0.06	6.82	-0.63	0.53029
RPL32P3	0.08	7.52	0.63	0.53042
AGAP14P	0.09	5.05	0.63	0.53055
AC133555.4	0.08	7.28	0.63	0.53057
TRMT6	-0.04	7.35	-0.63	0.53058
AMZ2	-0.04	9.29	-0.63	0.53062
AC055839.2	-0.08	7.59	-0.63	0.53090
CALM2P4	-0.09	5.91	-0.63	0.53118
APCDD1	-0.09	5.27	-0.63	0.53135
ACKR3	-0.07	6.39	-0.63	0.53141
PPP1R11	0.04	10.31	0.63	0.53176
SLC38A6	-0.06	5.83	-0.63	0.53183
SYF2	0.05	9.79	0.63	0.53192
PIM3	0.07	9.78	0.63	0.53192
PLK1	-0.07	6.21	-0.63	0.53202
IGHJ5	0.13	7.16	0.63	0.53217
GREM2	-0.09	4.90	-0.63	0.53223
NKAPL	0.08	5.21	0.63	0.53225
AC018607.1	0.11	5.11	0.63	0.53229
MAP1A	0.08	7.38	0.63	0.53240
FCGR2B	-0.10	8.67	-0.63	0.53245
LMO7	-0.09	6.43	-0.63	0.53247
DUSP10	-0.05	6.78	-0.63	0.53269
ZFAND3	-0.06	10.69	-0.63	0.53275
H3C10	-0.10	7.76	-0.63	0.53292
PXYLP1	-0.08	7.05	-0.63	0.53294
PPP2R2B	-0.08	7.01	-0.63	0.53306
MCM3	0.04	9.99	0.63	0.53315
MAST3	-0.06	11.72	-0.63	0.53339
AP001000.1	-0.09	5.89	-0.63	0.53362
KDM4A	0.04	8.57	0.63	0.53381
UTP20	-0.06	6.93	-0.63	0.53381
ALAD	-0.04	8.85	-0.63	0.53381
GLO1	0.07	7.92	0.63	0.53401
TYW5	0.06	6.61	0.63	0.53403
OLA1	0.05	8.09	0.63	0.53405
AC099343.3	-0.09	5.69	-0.63	0.53410
MTERF1	-0.05	6.53	-0.63	0.53410
AC037459.2	0.14	6.95	0.63	0.53429
CEP250-AS1	-0.06	6.49	-0.63	0.53452
IFIT1B	0.14	6.73	0.63	0.53466
LINC01094	-0.08	5.87	-0.62	0.53486
TTLL3	-0.09	8.82	-0.62	0.53497
LIN37	-0.08	6.08	-0.62	0.53529
CXCL8	-0.09	5.78	-0.62	0.53542
TRBJ1-1	0.09	5.66	0.62	0.53574

ZNF836	0.05	6.64	0.62	0.53594
RSU1	0.05	9.84	0.62	0.53599
EFNA1	0.09	5.29	0.62	0.53602
SYT11	-0.06	8.63	-0.62	0.53608
MBD6	-0.09	11.34	-0.62	0.53612
TMEM121	0.08	5.17	0.62	0.53639
TMEM163	0.08	4.92	0.62	0.53655
SUPT7L	-0.04	9.00	-0.62	0.53661
NRBP2	-0.09	6.98	-0.62	0.53668
NPTXR	0.08	7.62	0.62	0.53683
DUSP3	-0.05	8.87	-0.62	0.53689
ZCCHC7	0.05	7.92	0.62	0.53708
TRIM7	0.07	6.36	0.62	0.53712
MIR103A2	0.06	4.98	0.62	0.53714
NECTIN2	-0.22	7.59	-0.62	0.53721
AC084824.2	0.07	7.43	0.62	0.53724
IGKV2D-30	0.13	5.48	0.62	0.53730
FXYD6	-0.09	7.57	-0.62	0.53746
IFT20	-0.05	7.95	-0.62	0.53750
AC017104.6	-0.07	5.13	-0.62	0.53754
AC104996.2	-0.06	5.05	-0.62	0.53776
ZNF706	-0.04	8.99	-0.62	0.53796
AC116366.2	-0.09	5.72	-0.62	0.53798
FBLN2	0.10	6.27	0.62	0.53800
IFFO2	0.05	9.51	0.62	0.53817
ATXN2	0.06	9.05	0.62	0.53829
PIPSL	0.07	6.92	0.62	0.53839
TRAV8-2	-0.09	5.98	-0.62	0.53840
TMEM131L	-0.03	10.29	-0.62	0.53850
MS4A7	-0.09	8.88	-0.62	0.53852
SEC63P1	-0.06	5.02	-0.62	0.53870
AC004951.1	0.07	5.18	0.62	0.53874
PARP6	-0.04	9.27	-0.62	0.53878
AC100803.3	0.11	5.63	0.62	0.53883
AP000529.1	0.08	5.85	0.62	0.53888
TRMT2B	0.05	8.06	0.62	0.53922
AL122035.2	-0.08	5.02	-0.62	0.53929
AC027020.2	-0.10	6.58	-0.62	0.53953
SLC39A8	-0.07	6.83	-0.62	0.53959
GTPBP8	0.05	6.44	0.62	0.53960
YTHDF2P1	-0.06	5.01	-0.62	0.53961
AC107959.3	-0.09	7.08	-0.62	0.53966
MAD1L1	0.08	7.31	0.62	0.53977
LINC02033	0.07	5.06	0.62	0.53986
SPTBN1	-0.08	10.15	-0.62	0.53987
WDR12	-0.05	6.89	-0.62	0.53988
ZFP69	-0.05	6.10	-0.62	0.54021
AC010186.2	-0.06	6.17	-0.62	0.54026
NAIPP3	-0.07	5.21	-0.62	0.54035
KMT5C	0.07	7.32	0.62	0.54061

ZNF619	-0.05	6.38	-0.62	0.54062
IL18	0.06	6.92	0.62	0.54066
FAM200B	-0.05	7.91	-0.62	0.54107
TRIM52	-0.06	7.83	-0.62	0.54114
ZNF441	-0.07	6.07	-0.62	0.54115
AL035587.2	0.07	5.87	0.62	0.54115
PSMB9	-0.07	11.82	-0.62	0.54116
GFOD1	0.07	8.07	0.62	0.54121
CSTA	-0.08	10.01	-0.62	0.54124
GPR19	-0.08	5.16	-0.61	0.54137
CACYBPP2	-0.07	5.60	-0.61	0.54156
GPR89B	0.05	5.93	0.61	0.54172
CLTCL1	-0.09	7.27	-0.61	0.54196
GLRXP3	0.07	5.08	0.61	0.54200
AL445524.1	-0.08	5.92	-0.61	0.54205
HMGB1P5	-0.06	7.55	-0.61	0.54209
MORC4	-0.05	6.32	-0.61	0.54209
EIF1P7	-0.06	5.56	-0.61	0.54211
AC084036.1	0.07	4.89	0.61	0.54213
TEFM	-0.05	6.27	-0.61	0.54222
ELOA	-0.04	9.00	-0.61	0.54243
TCP1	-0.04	9.84	-0.61	0.54256
DPEP2	0.07	12.08	0.61	0.54259
ZNF529-AS1	-0.06	5.80	-0.61	0.54265
CST7	0.12	11.88	0.61	0.54272
RABL3	0.05	7.25	0.61	0.54272
GLTP	0.04	9.64	0.61	0.54273
SLPI	-0.13	7.80	-0.61	0.54277
RPL30P2	0.05	5.17	0.61	0.54322
SNRPD3	0.05	8.36	0.61	0.54330
NAA16	-0.06	7.26	-0.61	0.54336
NIPSNAP3B	-0.08	5.27	-0.61	0.54346
ATP6V0D1	0.06	12.35	0.61	0.54352
FAM177A1	-0.05	7.51	-0.61	0.54369
LIN7B	0.06	6.20	0.61	0.54383
LINC02284	-0.07	5.12	-0.61	0.54398
ZDHHC19	-0.11	5.62	-0.61	0.54439
TBC1D3L	-0.11	7.18	-0.61	0.54460
DOK4	0.09	6.67	0.61	0.54480
CD59	-0.08	8.82	-0.61	0.54490
STAB1	-0.09	10.96	-0.61	0.54508
TMUB2	0.06	10.39	0.61	0.54519
RRBP1	0.05	10.71	0.61	0.54522
AC135068.2	0.10	5.48	0.61	0.54533
ZNF569	-0.05	5.74	-0.61	0.54536
NXT2	-0.06	6.27	-0.61	0.54558
YAE1	-0.06	5.67	-0.61	0.54563
CACNG8	0.09	5.51	0.61	0.54565
RANP1	0.08	6.32	0.61	0.54570
LINC00884	0.07	5.25	0.61	0.54578

SH3BP2	-0.05	11.62	-0.61	0.54585
SERPINB1	-0.07	11.91	-0.61	0.54587
ZSWIM7	-0.04	7.78	-0.61	0.54588
GPN3	-0.05	6.63	-0.61	0.54591
FCRL5	-0.15	7.49	-0.61	0.54627
ST8SIA1	0.10	6.12	0.61	0.54649
ADAP2	0.07	8.45	0.61	0.54655
SH3BP5L	0.05	10.60	0.61	0.54657
CD24	-0.08	7.58	-0.61	0.54666
MT-TF	0.12	7.60	0.61	0.54667
ZNF79	-0.04	7.11	-0.61	0.54673
ALG1L13P	-0.10	6.98	-0.61	0.54716
KBTBD11	-0.05	8.28	-0.61	0.54730
OGFR	0.05	10.99	0.61	0.54744
AC092115.2	-0.09	6.69	-0.60	0.54783
PET100	0.06	8.13	0.60	0.54784
SLC9A3-AS1	-0.11	6.51	-0.60	0.54789
LGALS9B	-0.09	6.13	-0.60	0.54789
MRVI1-AS1	0.08	5.41	0.60	0.54805
SLC15A3	0.07	11.05	0.60	0.54811
ABCC1	-0.04	9.87	-0.60	0.54815
SNHG17	0.08	8.04	0.60	0.54818
MMP15	-0.08	5.15	-0.60	0.54820
DCTN1	0.04	10.96	0.60	0.54839
ITGAE	-0.06	7.80	-0.60	0.54863
ATP6V1G2	-0.07	5.04	-0.60	0.54872
MYL6P5	0.08	6.29	0.60	0.54898
FBP1	0.07	9.25	0.60	0.54923
SPATA2	0.04	8.31	0.60	0.54924
WAKMAR2	0.07	5.89	0.60	0.54930
ZNF540	0.08	5.85	0.60	0.54937
PYROXD1	-0.05	6.73	-0.60	0.54939
STAG3	-0.09	5.88	-0.60	0.54948
IGHV3-73	0.12	5.85	0.60	0.54958
EPHB4	-0.11	8.56	-0.60	0.54970
AL512306.2	-0.09	6.63	-0.60	0.54995
AL353763.2	-0.08	6.20	-0.60	0.54996
AL592295.1	0.05	5.30	0.60	0.55010
WDR43	-0.05	7.63	-0.60	0.55011
CTC1	-0.06	10.29	-0.60	0.55026
PPIL1	0.05	7.05	0.60	0.55026
LLGL1	0.04	8.72	0.60	0.55028
USP42	0.06	7.96	0.60	0.55037
NME1	0.07	7.10	0.60	0.55038
CAP1	-0.05	13.34	-0.60	0.55052
FRAT1	-0.08	10.59	-0.60	0.55065
CNST	-0.05	8.26	-0.60	0.55079
GAA	0.06	11.35	0.60	0.55080
TRBV6-6	0.09	6.65	0.60	0.55082
RGS19	0.06	11.41	0.60	0.55113

PIP4P1	-0.03	9.35	-0.60	0.55128
FAM161B	0.06	5.93	0.60	0.55130
SLC25A39P1	0.14	6.10	0.60	0.55166
TTLL1	0.05	7.11	0.60	0.55174
EIF3KP1	0.08	5.84	0.60	0.55182
CD244	-0.05	9.48	-0.60	0.55191
AL731571.1	0.07	4.95	0.60	0.55199
CCSER2	-0.05	8.18	-0.60	0.55219
LINC02325	0.07	5.68	0.60	0.55229
TNK2	0.06	11.02	0.60	0.55240
NUTM2D	0.08	5.42	0.60	0.55267
AC083873.1	0.07	5.76	0.60	0.55268
TBC1D3D	-0.07	6.40	-0.60	0.55268
HNRNPA3P6	-0.09	6.42	-0.60	0.55270
NHLH1	-0.07	5.03	-0.60	0.55287
DICER1-AS1	-0.08	6.01	-0.60	0.55293
TRPC2	0.08	5.11	0.60	0.55294
TTC3	0.04	10.09	0.60	0.55300
AL596087.1	-0.08	6.81	-0.60	0.55331
AL138995.1	-0.06	5.30	-0.60	0.55369
TPRKB	0.05	7.01	0.60	0.55379
CYTH3	-0.06	7.61	-0.60	0.55381
PCBP3	-0.07	5.70	-0.60	0.55404
WBP1L	0.04	10.62	0.60	0.55415
LILRP1	-0.10	6.65	-0.60	0.55416
AC111152.3	0.08	5.84	0.60	0.55421
FAAH2	-0.08	5.66	-0.59	0.55448
CALM1P2	0.07	5.35	0.59	0.55451
TNIK	-0.06	8.05	-0.59	0.55456
GSKIP	-0.07	7.25	-0.59	0.55479
LYRM1	-0.06	7.67	-0.59	0.55503
WASH6P	0.07	7.87	0.59	0.55506
FBXW8	-0.06	6.72	-0.59	0.55509
FNBP1	-0.03	11.63	-0.59	0.55510
Z97180.1	-0.07	5.15	-0.59	0.55511
TIMM10B	-0.04	7.95	-0.59	0.55533
LTF	0.25	9.95	0.59	0.55542
LAMTOR5-AS1	-0.07	5.04	-0.59	0.55555
ABCD1	0.05	8.92	0.59	0.55557
ZFP1	-0.06	5.88	-0.59	0.55569
RGPD1	0.07	5.03	0.59	0.55572
AC007384.1	0.05	6.13	0.59	0.55573
TTYH2	-0.05	9.16	-0.59	0.55575
SMAGP	0.07	6.41	0.59	0.55580
NARF	0.08	11.06	0.59	0.55585
TUBGCP6	0.06	10.38	0.59	0.55590
DRC7	-0.08	5.27	-0.59	0.55604
AC036176.1	0.06	4.98	0.59	0.55604
ASXL1	-0.04	9.91	-0.59	0.55610
AC241952.2	-0.08	5.97	-0.59	0.55636

SNX21	0.07	6.60	0.59	0.55643
HCFC1	0.04	10.31	0.59	0.55647
AL109809.4	-0.08	6.06	-0.59	0.55651
ST7L	0.05	6.50	0.59	0.55662
EMC1-AS1	0.06	5.19	0.59	0.55664
RAB3A	0.05	6.04	0.59	0.55677
AL031283.1	-0.08	5.50	-0.59	0.55684
MTMR2	-0.05	6.64	-0.59	0.55689
PSMD6-AS2	-0.08	5.58	-0.59	0.55707
TAF1C	0.07	9.49	0.59	0.55708
TRDMT1	-0.06	6.27	-0.59	0.55714
DDX42	-0.04	10.08	-0.59	0.55724
AC009533.1	0.10	5.84	0.59	0.55731
H4C4	0.09	5.09	0.59	0.55733
PCNA	0.05	8.42	0.59	0.55760
UGGT2	-0.07	5.06	-0.59	0.55771
MRFAP1L1	0.03	9.67	0.59	0.55774
CASP4	-0.07	11.69	-0.59	0.55792
KRT73	-0.13	6.11	-0.59	0.55792
ZGPAT	0.06	7.11	0.59	0.55799
SLX1B	0.05	5.30	0.59	0.55800
SCAMP1-AS1	0.05	7.13	0.59	0.55800
TMEM185B	0.06	8.95	0.59	0.55808
GAPDHP38	0.08	6.13	0.59	0.55811
ATP9B	0.04	8.35	0.59	0.55814
JHY	0.06	5.73	0.59	0.55836
KMT2E-AS1	0.08	6.31	0.59	0.55838
HYAL2	-0.05	6.84	-0.59	0.55838
PIP5K1A	-0.04	8.78	-0.59	0.55848
ATF4P3	0.07	6.40	0.59	0.55850
GPHN	0.05	6.38	0.59	0.55859
TMEM183B	0.06	6.03	0.59	0.55871
HSP90AB3P	-0.09	7.40	-0.59	0.55872
LAP3	-0.11	9.52	-0.59	0.55874
AIMP1	-0.04	8.46	-0.59	0.55878
KLHDC7B	0.07	7.73	0.59	0.55879
CAMK2G	0.05	10.81	0.59	0.55883
RRN3P1	-0.08	6.45	-0.59	0.55886
CDKAL1	-0.05	7.16	-0.59	0.55902
POTEKP	0.08	6.95	0.59	0.55919
CASTOR2	-0.06	7.80	-0.59	0.55924
TIAM2	-0.07	6.71	-0.59	0.55936
AL596244.1	0.05	5.65	0.59	0.55963
TTC39C	0.06	8.49	0.59	0.55964
PANK2	-0.04	9.55	-0.59	0.55978
SNED1	-0.07	6.71	-0.59	0.55981
ZC3H7B	-0.04	9.58	-0.59	0.55992
TRBV21-1	0.10	5.49	0.59	0.56002
CARMIL1	-0.07	5.98	-0.59	0.56015
ACBD6	-0.04	8.24	-0.59	0.56020

CAMSAP1	-0.04	8.17	-0.59	0.56021
SELENOI	-0.07	6.62	-0.59	0.56024
EXOSC6	0.05	7.77	0.59	0.56024
AL731557.1	-0.09	6.46	-0.59	0.56027
WASH2P	0.07	7.96	0.59	0.56028
C12orf42	0.08	5.52	0.59	0.56029
CEP72	0.06	6.00	0.59	0.56032
AL138721.1	0.07	5.24	0.59	0.56050
FAXDC2	0.12	8.83	0.59	0.56050
MFAP1	0.04	8.47	0.59	0.56064
PHACTR1	0.08	7.41	0.59	0.56074
LINC00926	0.11	9.00	0.59	0.56093
TUBA1B	0.04	11.50	0.59	0.56104
FBXO7	0.12	13.61	0.58	0.56125
RPL5P34	0.09	8.01	0.58	0.56128
ALMS1	-0.05	7.34	-0.58	0.56129
PRRC2A	0.04	12.25	0.58	0.56140
LINC02649	-0.12	6.14	-0.58	0.56142
HES4	-0.11	5.61	-0.58	0.56148
MYO1D	0.06	6.82	0.58	0.56175
AC006927.1	-0.08	5.19	-0.58	0.56176
SOX6	0.12	5.88	0.58	0.56189
AIFM1	-0.05	8.60	-0.58	0.56189
IGHV3-69-1	0.10	6.07	0.58	0.56209
ZNF34	-0.05	6.49	-0.58	0.56214
CIAPIN1P	0.07	5.09	0.58	0.56236
DHX57	0.04	8.17	0.58	0.56260
PLEKHG2	0.05	8.32	0.58	0.56264
AC136475.3	-0.13	8.08	-0.58	0.56264
AC009682.1	-0.09	4.95	-0.58	0.56266
CARNS1	0.08	7.88	0.58	0.56270
HCCS	-0.04	7.82	-0.58	0.56275
CDC37L1	-0.04	7.05	-0.58	0.56277
RILPL2	0.06	10.51	0.58	0.56285
UCHL3	0.05	5.77	0.58	0.56332
GDPD1	0.06	5.35	0.58	0.56355
TRAV13-2	0.08	5.74	0.58	0.56364
MAP3K3	-0.05	11.81	-0.58	0.56370
CPSF6	0.03	8.95	0.58	0.56374
MTHFD1	-0.05	8.34	-0.58	0.56375
PPP2CA	-0.04	9.96	-0.58	0.56386
PCAT1	-0.07	5.34	-0.58	0.56412
DIPK1A	0.06	6.40	0.58	0.56430
ZNF189	0.05	6.98	0.58	0.56432
ZNF418	-0.09	5.59	-0.58	0.56438
GANC	-0.06	7.38	-0.58	0.56449
FBXO41	0.06	8.20	0.58	0.56462
BCKDK	0.06	10.15	0.58	0.56469
SRP14-AS1	-0.05	5.77	-0.58	0.56484
AC023794.5	0.06	5.19	0.58	0.56496

TSPAN4	0.08	7.92	0.58	0.56512
ACKR1	0.12	6.05	0.58	0.56519
CCNT2-AS1	0.07	5.37	0.58	0.56523
ITPKC	0.07	8.47	0.58	0.56529
UQCRC2	-0.03	10.32	-0.58	0.56534
WDR83	0.04	7.36	0.58	0.56537
RPN1	0.05	11.69	0.58	0.56551
VAV2	0.06	8.70	0.58	0.56552
ODF3B	-0.11	9.08	-0.58	0.56556
TPST2	0.05	10.05	0.58	0.56561
AC004847.1	-0.05	8.11	-0.58	0.56573
STARD7-AS1	0.06	6.22	0.58	0.56603
AC009779.4	-0.07	5.26	-0.58	0.56612
TNK2-AS1	-0.07	5.72	-0.58	0.56615
ANKRD17	-0.05	9.06	-0.58	0.56628
HDAC10	0.07	6.61	0.58	0.56639
FCRL2	-0.12	7.24	-0.58	0.56649
SLC22A1	-0.09	6.68	-0.58	0.56658
JPT1	0.07	10.79	0.58	0.56659
AL359220.1	0.08	6.67	0.58	0.56665
SMPDL3B	-0.07	5.76	-0.58	0.56681
SLC25A5	0.04	10.99	0.58	0.56689
ZNF337	-0.05	8.11	-0.58	0.56695
MANEA	-0.07	5.98	-0.58	0.56723
IMMP1L	0.06	5.57	0.58	0.56745
NUDT17	0.06	6.26	0.58	0.56745
CBX8	0.05	6.83	0.58	0.56745
ZNF799	-0.06	5.74	-0.58	0.56758
DTWD1	0.05	6.02	0.58	0.56763
GALE	0.05	7.44	0.58	0.56763
SLC25A3P2	0.07	5.81	0.57	0.56783
RFLNB	0.06	10.91	0.57	0.56815
CYSTM1	-0.11	9.35	-0.57	0.56815
CDKN2D	0.07	11.13	0.57	0.56824
SLC25A19	0.06	7.70	0.57	0.56845
REEP5	-0.04	10.15	-0.57	0.56854
GTF2E2	-0.05	7.79	-0.57	0.56871
ATF4P4	-0.05	5.39	-0.57	0.56871
CALM2P3	-0.07	5.90	-0.57	0.56878
PPP1R26	-0.05	6.69	-0.57	0.56891
PDHB	-0.04	8.67	-0.57	0.56908
CENPL	-0.05	5.68	-0.57	0.56915
AC100786.1	0.07	6.45	0.57	0.56920
TIAF1	0.06	6.06	0.57	0.56941
H2AC11	-0.07	5.79	-0.57	0.56962
SMIM20	0.04	7.26	0.57	0.56966
GPR35	0.06	7.46	0.57	0.56969
DND1	0.06	5.25	0.57	0.56974
TRIAP1	0.05	7.80	0.57	0.56978
ELOBP1	0.08	5.82	0.57	0.56993

BTG2	-0.05	11.71	-0.57	0.56993
CDT1	0.08	5.99	0.57	0.56998
OTUD3	0.05	6.59	0.57	0.57006
RPS3AP5	0.08	6.74	0.57	0.57006
NAP1L2	-0.06	5.41	-0.57	0.57020
FZD5	-0.06	5.16	-0.57	0.57062
TMEM199	0.04	8.03	0.57	0.57064
STK40	-0.06	11.92	-0.57	0.57067
CEP57L1	-0.07	5.12	-0.57	0.57077
PAFAH1B2	0.04	9.69	0.57	0.57082
YJEFN3	-0.09	6.39	-0.57	0.57106
RBCK1	-0.05	11.22	-0.57	0.57111
ZNF204P	-0.06	5.51	-0.57	0.57112
SMARCC1	-0.04	9.74	-0.57	0.57115
NSA2	-0.05	9.96	-0.57	0.57122
FAM122C	-0.06	6.01	-0.57	0.57133
HLA-DMA	0.07	10.96	0.57	0.57143
AXL	0.07	5.08	0.57	0.57150
ZNF585B	-0.06	6.70	-0.57	0.57154
LACTB2	0.05	5.89	0.57	0.57159
ENSAP2	0.05	5.84	0.57	0.57216
MYO19	0.05	7.96	0.57	0.57218
FUCA1	-0.06	8.46	-0.57	0.57222
STPG1	0.05	6.23	0.57	0.57223
GBP1P1	-0.10	5.06	-0.57	0.57230
CAPN10	0.06	8.73	0.57	0.57231
HLCS	-0.04	6.64	-0.57	0.57246
FKBP3	0.04	8.00	0.57	0.57268
IL3RA	0.09	8.08	0.57	0.57268
TMEM9B-AS1	0.07	5.95	0.57	0.57279
EI24	0.04	8.53	0.57	0.57282
TRAV38-1	0.07	5.58	0.57	0.57325
DOCK6	-0.07	5.58	-0.57	0.57327
PNMA6A	-0.09	5.13	-0.57	0.57338
UBAP1L	-0.08	6.75	-0.57	0.57345
EOMES	-0.07	8.69	-0.57	0.57358
HLA-DRB6	-0.17	9.84	-0.57	0.57362
EPB41L2	-0.07	6.75	-0.57	0.57362
DROSHA	-0.04	8.30	-0.57	0.57387
PRSS30P	0.11	6.49	0.57	0.57419
SPATA5L1	-0.05	6.89	-0.57	0.57425
TSPAN14	0.04	11.98	0.57	0.57427
MMACHC	0.06	5.92	0.56	0.57487
GTF2IRD2B	-0.06	6.33	-0.56	0.57529
TPPP	0.07	6.26	0.56	0.57531
HS2ST1	0.06	6.95	0.56	0.57552
MFSD11	0.04	7.70	0.56	0.57557
DHX16	-0.03	9.85	-0.56	0.57558
RNF165	-0.09	6.08	-0.56	0.57574
CD160	0.09	7.45	0.56	0.57594

STAG3L1	-0.08	5.23	-0.56	0.57605
DBH-AS1	0.09	5.88	0.56	0.57611
ZNF397	-0.04	8.49	-0.56	0.57620
ACP1	-0.04	9.25	-0.56	0.57632
ST14	0.07	9.75	0.56	0.57633
TMEM273	-0.09	8.81	-0.56	0.57645
EEF1A1P12	0.06	7.74	0.56	0.57659
SLC25A46	-0.05	7.48	-0.56	0.57662
AC020978.7	0.06	4.97	0.56	0.57676
AC006970.2	0.09	6.80	0.56	0.57677
LSM3P3	0.06	5.18	0.56	0.57688
MLH1	0.03	8.39	0.56	0.57688
AL365203.2	0.07	6.45	0.56	0.57705
GMPR	0.14	9.58	0.56	0.57711
CCDC151	-0.08	5.03	-0.56	0.57731
ZMYND10	0.06	5.99	0.56	0.57746
PNMA1	-0.04	8.50	-0.56	0.57752
DLG4	0.06	8.35	0.56	0.57755
ZC3H15	0.04	8.47	0.56	0.57773
SUMO1	-0.04	9.12	-0.56	0.57776
JPX	0.06	8.12	0.56	0.57779
DCTN6	-0.05	6.58	-0.56	0.57811
ATP5F1AP10	0.05	5.12	0.56	0.57824
AL049840.5	-0.07	6.14	-0.56	0.57827
C14orf28	0.06	5.82	0.56	0.57847
POLR1A	-0.04	8.77	-0.56	0.57858
DGAT1	0.06	10.45	0.56	0.57861
SPATA41	0.06	5.33	0.56	0.57869
KIAA1324L	0.09	5.64	0.56	0.57879
COL6A3	0.08	5.71	0.56	0.57894
SLC12A7	-0.10	10.14	-0.56	0.57901
RAP1GDS1	-0.04	8.24	-0.56	0.57905
C12orf29	-0.05	6.25	-0.56	0.57911
RABIF	-0.04	8.02	-0.56	0.57917
AC232271.1	-0.08	5.73	-0.56	0.57920
FAM111A-DT	-0.04	6.01	-0.56	0.57959
CD180	0.07	8.89	0.56	0.57963
PPP1R2B	-0.06	5.18	-0.56	0.57965
NEMP2	-0.04	6.61	-0.56	0.57965
FAM131B	-0.07	5.73	-0.56	0.57970
CHKA	0.05	6.94	0.56	0.57975
MCEMP1	-0.15	8.69	-0.56	0.57975
CLC	0.13	9.69	0.56	0.57992
TBX19	-0.05	6.54	-0.56	0.57998
NFATC3	0.04	10.48	0.56	0.58001
ATAD1	-0.05	7.54	-0.56	0.58010
PDE2A	-0.07	5.64	-0.56	0.58022
AC090204.1	-0.05	5.07	-0.56	0.58035
LINC00863	-0.06	5.66	-0.56	0.58069
FAM120AOS	0.03	9.37	0.56	0.58083

BUB3	-0.03	9.57	-0.56	0.58100
ZFP36	-0.06	11.64	-0.56	0.58102
MYL6P3	0.08	6.48	0.56	0.58103
LSM1	-0.04	8.33	-0.56	0.58109
PPP6R2	0.04	10.39	0.56	0.58117
PPHLN1	0.03	8.84	0.56	0.58121
ZC3H12A	-0.04	8.33	-0.55	0.58136
CDK18	0.07	6.33	0.55	0.58142
IGHV3-74	0.14	7.45	0.55	0.58146
AP005482.2	0.08	5.56	0.55	0.58150
ZNF717	-0.06	5.90	-0.55	0.58165
AC005840.4	-0.06	5.67	-0.55	0.58167
AC009974.1	0.08	4.94	0.55	0.58169
CD1E	0.06	5.07	0.55	0.58172
SIGLEC15	-0.06	5.50	-0.55	0.58178
R3HCC1L	-0.03	8.49	-0.55	0.58181
TMEM25	0.06	7.04	0.55	0.58185
GZMB	0.10	11.19	0.55	0.58190
ZNF284	-0.05	5.72	-0.55	0.58194
COL8A2	-0.06	6.05	-0.55	0.58208
CMIP	-0.06	11.13	-0.55	0.58212
CASZ1	-0.04	7.53	-0.55	0.58214
TRAV1-1	0.08	5.19	0.55	0.58221
COX10-AS1	-0.06	5.99	-0.55	0.58230
RCN1	0.06	6.75	0.55	0.58245
GATA2	-0.09	8.25	-0.55	0.58249
FBXO48	-0.04	6.13	-0.55	0.58258
N4BP2L2-IT2	-0.07	5.91	-0.55	0.58268
COX18	0.05	6.70	0.55	0.58274
TNRC18	-0.05	10.75	-0.55	0.58288
ABHD15	0.04	8.73	0.55	0.58289
NTPCR	-0.06	7.58	-0.55	0.58290
LRRC57	-0.04	7.46	-0.55	0.58300
DENND4A	-0.05	8.53	-0.55	0.58307
SLC6A12	-0.06	6.79	-0.55	0.58315
VIM	0.05	14.60	0.55	0.58328
VTI1B	0.04	9.61	0.55	0.58334
TTLL4	-0.05	8.91	-0.55	0.58345
TCEAL4	0.06	7.30	0.55	0.58348
AL390728.6	-0.07	6.36	-0.55	0.58349
PSD	0.08	5.71	0.55	0.58367
EDC4	-0.04	9.79	-0.55	0.58376
GTF3C2	-0.03	9.69	-0.55	0.58379
TBC1D12	-0.06	5.28	-0.55	0.58390
ACADM	-0.06	7.00	-0.55	0.58406
USP6NL	-0.06	6.75	-0.55	0.58446
IGHV5-78	0.07	5.25	0.55	0.58457
EML2	0.03	9.20	0.55	0.58460
OGFOD1	0.05	8.45	0.55	0.58472
KIF19	-0.09	5.79	-0.55	0.58475

ATP5F1AP7	0.06	5.08	0.55	0.58481
MCCC1	-0.03	7.83	-0.55	0.58481
HSPD1P4	0.07	5.28	0.55	0.58496
AC126773.5	-0.05	5.14	-0.55	0.58503
AC009948.2	0.07	5.55	0.55	0.58509
HNRNPLP2	0.07	7.22	0.55	0.58537
SIGLEC5	0.09	6.58	0.55	0.58559
IRF5	-0.06	9.94	-0.55	0.58563
TMEM186	0.05	7.34	0.55	0.58564
AC078819.1	-0.05	5.75	-0.55	0.58577
JAKMIP3	0.07	4.96	0.55	0.58580
TUBGCP5	-0.06	6.79	-0.55	0.58601
CYB5B	-0.03	8.87	-0.55	0.58606
LOXL1-AS1	0.05	6.47	0.55	0.58639
ACTBP2	-0.08	8.75	-0.55	0.58640
GOLGA3	0.03	9.92	0.55	0.58642
RAD51-AS1	-0.07	7.00	-0.55	0.58660
IGKV1D-27	-0.07	5.05	-0.55	0.58664
ANKDD1A	0.08	5.80	0.55	0.58667
CORO2B	0.10	5.70	0.55	0.58675
ISG15	-0.17	9.72	-0.55	0.58684
EEF1A1P9	0.07	8.02	0.55	0.58687
AC023157.3	0.09	7.54	0.55	0.58698
CENPH	-0.06	5.79	-0.55	0.58705
NUMBL	0.06	7.09	0.55	0.58707
POPDC2	0.06	5.85	0.55	0.58729
SLC25A25	0.04	8.26	0.55	0.58731
AC025518.1	0.10	5.54	0.55	0.58738
DNAJC3-DT	0.06	5.95	0.55	0.58746
IGHV3-72	0.12	5.88	0.55	0.58746
POLR2D	-0.04	7.84	-0.55	0.58756
CPT1B	0.07	5.38	0.55	0.58765
PIGG	-0.05	8.99	-0.55	0.58769
ZNRF2	0.06	6.26	0.55	0.58775
LPCAT3	-0.05	9.61	-0.55	0.58786
TRG-AS1	-0.05	8.18	-0.55	0.58797
GAPDHP1	0.08	8.43	0.55	0.58798
DYNLL1	0.05	10.40	0.55	0.58804
ZNF595	-0.06	6.30	-0.54	0.58818
IL12RB1	0.05	9.79	0.54	0.58822
HMGN2P5	0.08	7.96	0.54	0.58832
NCAPD2	-0.03	10.00	-0.54	0.58834
SLC35F2	-0.05	5.92	-0.54	0.58835
LMBR1	-0.04	7.53	-0.54	0.58852
FKBP1A	-0.05	11.51	-0.54	0.58890
WASH5P	-0.08	5.19	-0.54	0.58896
RNPEP	0.05	10.71	0.54	0.58897
TMPRSS13	0.06	6.13	0.54	0.58901
SKP1	0.04	10.07	0.54	0.58904
KHDRBS1	-0.03	10.45	-0.54	0.58905

PANX2	-0.10	8.36	-0.54	0.58933
AC009403.1	-0.05	5.48	-0.54	0.58935
AC105219.1	0.05	5.65	0.54	0.58936
HOMER3	0.07	7.71	0.54	0.58971
ALG2	-0.04	7.73	-0.54	0.58993
RNF14	-0.06	7.45	-0.54	0.59008
TMEM106A	0.05	8.07	0.54	0.59020
HLA-DQB2	0.16	8.53	0.54	0.59031
CDCA7L	0.06	7.90	0.54	0.59044
DPY19L1	-0.05	7.30	-0.54	0.59053
DAPK2	-0.09	9.52	-0.54	0.59068
TRAV30	-0.06	5.16	-0.54	0.59076
TMEM176A	0.22	9.10	0.54	0.59089
LINC01503	-0.07	7.10	-0.54	0.59099
ERCC4	-0.04	6.03	-0.54	0.59101
HERC2P3	-0.10	5.41	-0.54	0.59130
PIP5K1B	-0.07	6.21	-0.54	0.59131
ST13P18	0.06	5.57	0.54	0.59138
ZFYVE19	0.05	8.95	0.54	0.59149
NDUFA5	0.04	7.41	0.54	0.59150
PARM1	-0.08	5.61	-0.54	0.59151
DR1	-0.03	9.30	-0.54	0.59155
AL136116.3	0.05	5.33	0.54	0.59155
HOMEZ	-0.04	7.66	-0.54	0.59157
CALHM6	-0.08	5.79	-0.54	0.59159
AC010328.2	-0.05	5.19	-0.54	0.59160
TMEM179B	0.04	9.32	0.54	0.59164
PSD4	0.04	12.38	0.54	0.59174
ZNHIT6	0.05	6.88	0.54	0.59181
TRAPPC5	-0.05	6.10	-0.54	0.59195
EEF1A1P16	0.07	6.92	0.54	0.59200
FDFT1	0.04	10.35	0.54	0.59208
ZDHHC3	-0.04	9.91	-0.54	0.59241
WDR89	0.06	7.33	0.54	0.59243
ADGRD1	-0.08	6.17	-0.54	0.59254
MYL12BP2	-0.07	5.95	-0.54	0.59258
FTLP2	0.08	7.51	0.54	0.59262
AL118516.1	0.05	5.40	0.54	0.59263
MEGF8	0.04	7.94	0.54	0.59295
ATP6V1H	0.03	8.56	0.54	0.59298
KANSL1-AS1	0.10	6.57	0.54	0.59307
MTRNR2L8	0.10	7.76	0.54	0.59309
AC010733.1	0.06	5.05	0.54	0.59322
CD48	0.05	11.66	0.54	0.59359
CENPBD1P1	0.05	8.37	0.54	0.59386
CCDC180	-0.07	5.31	-0.54	0.59408
ZNF33B	0.05	7.85	0.54	0.59437
ATP5F1C	-0.04	10.10	-0.54	0.59459
SLC11A1	-0.11	12.83	-0.54	0.59460
CIAO2A	-0.04	8.10	-0.54	0.59461

S100A12	-0.11	11.16	-0.54	0.59479
PWP1	0.04	8.49	0.53	0.59508
SMARCE1	-0.05	5.82	-0.53	0.59510
MRPS35	0.04	8.16	0.53	0.59517
IBTK	-0.05	7.50	-0.53	0.59550
PTPN4	-0.05	8.76	-0.53	0.59550
LITAF	-0.07	13.37	-0.53	0.59553
KBTBD7	-0.07	7.60	-0.53	0.59562
XK	0.11	6.34	0.53	0.59569
TRBV4-2	0.11	6.68	0.53	0.59571
ATP1B3P1	0.06	5.03	0.53	0.59573
HNRNPLP1	0.05	5.65	0.53	0.59574
WDR1	0.04	13.21	0.53	0.59577
HM13	0.05	10.97	0.53	0.59581
PLB1	-0.08	9.01	-0.53	0.59591
ULK1	-0.07	10.86	-0.53	0.59602
CCDC30	0.06	5.89	0.53	0.59608
AC100771.1	0.06	5.63	0.53	0.59641
IGHV1-24	0.08	5.59	0.53	0.59665
ZFP64	0.04	7.45	0.53	0.59700
SLC25A5-AS1	0.05	5.27	0.53	0.59704
UNG	0.05	7.60	0.53	0.59705
IFT172	0.04	7.44	0.53	0.59708
GTF3C4	-0.04	7.27	-0.53	0.59723
HGS	0.04	10.61	0.53	0.59723
PROB1	0.06	5.34	0.53	0.59731
USP22	-0.03	10.99	-0.53	0.59735
OSER1-DT	0.05	7.68	0.53	0.59750
PILRA	-0.07	11.99	-0.53	0.59758
SCAPER	0.04	7.19	0.53	0.59762
PTAFR	-0.07	12.86	-0.53	0.59785
TYK2	-0.05	12.20	-0.53	0.59795
FBXW9	0.06	6.69	0.53	0.59799
LRRC14	0.04	8.74	0.53	0.59821
NDUFC2	0.06	7.40	0.53	0.59824
ZNF548	-0.05	6.81	-0.53	0.59834
ZNF138	-0.05	5.88	-0.53	0.59853
ATP5PB	0.03	10.37	0.53	0.59857
RAPGEF3	0.08	5.01	0.53	0.59858
TIRAP	0.05	7.51	0.53	0.59866
LINC02458	-0.07	5.13	-0.53	0.59869
DUSP22	-0.04	10.20	-0.53	0.59884
AC114801.3	0.06	5.26	0.53	0.59911
ZNF74	0.05	7.22	0.53	0.59914
GLIPR2	-0.06	11.76	-0.53	0.59914
AL121723.1	-0.09	6.59	-0.53	0.59917
RPS26P15	0.11	5.71	0.53	0.59928
RPS8P10	0.05	5.41	0.53	0.59948
VPS35	0.04	10.13	0.53	0.59981
CD300LF	-0.06	10.36	-0.53	0.59989

WDR4	0.05	7.01	0.53	0.59995
NLRP2	0.11	6.84	0.53	0.60000
CLCF1	-0.06	7.19	-0.53	0.60010
CDK10	0.06	9.60	0.53	0.60059
IGLV2-14	0.12	8.92	0.53	0.60074
AK2	-0.03	9.69	-0.53	0.60079
ITPKB-IT1	0.06	5.82	0.53	0.60084
FAIM	-0.06	5.33	-0.53	0.60110
GIMAP4	-0.04	12.00	-0.53	0.60110
ERGIC1	-0.08	11.87	-0.53	0.60113
SDHAF3	-0.05	6.20	-0.53	0.60115
HAUS2	-0.04	6.97	-0.53	0.60120
PLCB2	-0.05	12.38	-0.53	0.60122
BX571818.1	0.07	5.33	0.53	0.60130
RPN2	0.04	11.47	0.53	0.60145
EEF1A1P24	0.05	5.53	0.53	0.60158
RN7SL473P	-0.08	5.42	-0.53	0.60159
PCYOX1L	-0.04	8.12	-0.52	0.60196
TBC1D3K	-0.06	6.39	-0.52	0.60200
AC079905.1	0.06	5.43	0.52	0.60200
METTL2A	0.05	7.04	0.52	0.60203
MICAL1	0.05	11.60	0.52	0.60212
ASF1A	0.05	7.40	0.52	0.60229
RAB3IL1	0.12	6.36	0.52	0.60245
NLRP12	-0.07	10.73	-0.52	0.60246
RNASEH2B	0.04	8.78	0.52	0.60251
PARN	-0.03	8.99	-0.52	0.60251
GSTO1	0.06	9.53	0.52	0.60256
SLC22A18AS	-0.07	5.15	-0.52	0.60285
TBKBP1	-0.08	9.42	-0.52	0.60302
DHX36	-0.04	7.93	-0.52	0.60302
HSPA8P9	-0.07	6.17	-0.52	0.60305
CSF3R	-0.09	15.89	-0.52	0.60305
CWC25	0.05	9.43	0.52	0.60308
HFE	-0.06	6.48	-0.52	0.60310
PRPF40B	0.05	6.75	0.52	0.60325
TMEM14B	-0.04	7.44	-0.52	0.60326
SLC4A3	0.07	5.62	0.52	0.60329
MARCHF8	0.10	11.32	0.52	0.60330
AC012636.1	-0.06	4.96	-0.52	0.60355
ANKRD52	0.03	9.49	0.52	0.60355
RAB43	-0.07	6.27	-0.52	0.60356
AL512649.1	-0.06	5.18	-0.52	0.60359
DCAF5	-0.03	9.84	-0.52	0.60360
AP004609.5	-0.04	5.54	-0.52	0.60367
SELENOS	-0.05	8.32	-0.52	0.60387
AP000944.7	-0.06	4.96	-0.52	0.60427
SLC2A3P2	-0.08	5.05	-0.52	0.60440
SNUPN	-0.04	7.26	-0.52	0.60448
DELE1	0.03	9.65	0.52	0.60450

WASH4P	0.06	5.21	0.52	0.60467
AL158201.1	-0.08	6.76	-0.52	0.60481
SORD	-0.04	7.34	-0.52	0.60481
EEF1AKMT3	0.06	5.41	0.52	0.60486
AC022211.3	-0.06	4.93	-0.52	0.60501
AC096921.2	0.05	5.26	0.52	0.60510
ALDH2	-0.06	10.01	-0.52	0.60515
RPL23AP7	0.09	6.52	0.52	0.60545
MAN2C1	-0.06	10.63	-0.52	0.60549
ZIK1	-0.06	6.21	-0.52	0.60553
TRMO	0.03	7.91	0.52	0.60560
RASGRP3	-0.06	6.72	-0.52	0.60561
ATPSCKMT	-0.04	6.22	-0.52	0.60575
SPPL3	0.03	9.48	0.52	0.60577
CAMK4	-0.07	8.27	-0.52	0.60580
AC084198.4	0.05	5.66	0.52	0.60581
SLAMF7	-0.06	9.98	-0.52	0.60581
RIMBP3	0.06	5.72	0.52	0.60595
KCNMB1	-0.08	7.03	-0.52	0.60597
VMA21	-0.05	8.21	-0.52	0.60609
UPF2	0.05	9.70	0.52	0.60610
FAM136A	-0.04	7.58	-0.52	0.60614
BMI1	0.06	6.95	0.52	0.60618
CUTC	-0.03	8.11	-0.52	0.60619
INTS12	-0.04	7.52	-0.52	0.60639
KDM5D	-0.30	8.23	-0.52	0.60656
ZNF142	0.04	8.90	0.52	0.60656
LRMDA	-0.09	7.18	-0.52	0.60656
MAPK8IP3	0.07	10.12	0.52	0.60659
FBXO33	0.05	8.92	0.52	0.60660
NAV1	0.06	6.63	0.52	0.60668
TARS1	-0.04	8.87	-0.52	0.60672
PAG1	0.05	9.53	0.52	0.60677
ELMO1	-0.04	10.47	-0.52	0.60692
SBK1	0.05	9.07	0.52	0.60695
SLAMF8	-0.08	6.80	-0.52	0.60703
MTDH	0.05	9.79	0.52	0.60705
PEX5	-0.03	8.47	-0.52	0.60709
RPAIN	0.04	8.29	0.52	0.60713
ZNF184	-0.05	6.64	-0.52	0.60733
CNP	0.05	9.85	0.52	0.60745
ERO1A	-0.04	9.46	-0.52	0.60784
SUSD2	0.06	5.13	0.52	0.60793
C1QTNF6	0.08	6.78	0.52	0.60796
AL691432.2	0.04	6.40	0.52	0.60796
ZNF578	-0.06	4.97	-0.52	0.60800
UBA52P3	0.06	6.30	0.52	0.60802
UCN	0.06	5.19	0.52	0.60802
DNAAF2	-0.06	5.73	-0.52	0.60810
IMMT	0.03	9.59	0.52	0.60816

ZNF486	-0.06	6.23	-0.52	0.60823
GNE	-0.05	6.97	-0.52	0.60831
AEBP2	-0.05	6.89	-0.52	0.60838
GNAQ	-0.06	9.32	-0.52	0.60840
MTRNR2L1	0.19	6.83	0.52	0.60862
ZNF259P1	-0.06	5.04	-0.52	0.60862
PRKAR2A-AS1	-0.05	5.17	-0.52	0.60867
ADGRG3	-0.10	12.23	-0.52	0.60872
ACTBP7	0.04	5.10	0.51	0.60877
AC016734.1	0.07	7.32	0.51	0.60891
LINC01353	-0.06	4.93	-0.51	0.60903
ZDHHC16	0.04	8.13	0.51	0.60908
ZNF425	0.05	5.63	0.51	0.60920
TMPO-AS1	-0.05	5.70	-0.51	0.60926
COPRS	0.06	6.60	0.51	0.60935
HEXD-IT1	-0.06	4.92	-0.51	0.60939
ACER3	0.05	7.78	0.51	0.60942
TIMM17BP1	0.05	5.11	0.51	0.60951
SUOX	-0.05	7.40	-0.51	0.60973
NT5DC3	-0.06	6.37	-0.51	0.60981
RIBC1	-0.05	5.01	-0.51	0.60983
OSM	-0.09	6.71	-0.51	0.60986
AC097263.1	0.07	5.33	0.51	0.61000
ZKSCAN1	-0.04	8.73	-0.51	0.61002
POTEG	-0.04	4.98	-0.51	0.61027
TARDBP	-0.03	9.43	-0.51	0.61039
SMYD4	0.05	7.66	0.51	0.61045
AP003084.1	0.05	5.09	0.51	0.61054
ADH5P4	-0.06	5.29	-0.51	0.61071
ADPRHL1	0.06	5.14	0.51	0.61073
HMGB1	-0.03	10.37	-0.51	0.61075
HDAC1	0.02	10.95	0.51	0.61086
LINC00685	0.09	5.73	0.51	0.61094
FTO	-0.04	7.80	-0.51	0.61106
MMS19	0.04	9.76	0.51	0.61114
ZSCAN9	-0.05	6.71	-0.51	0.61120
INPPL1	-0.04	10.88	-0.51	0.61122
FUNDC2P1	-0.06	5.01	-0.51	0.61126
AC098859.1	0.06	5.32	0.51	0.61131
PDLIM7	0.07	11.02	0.51	0.61131
PRKRA	-0.04	7.19	-0.51	0.61141
UNC93B1	-0.06	10.92	-0.51	0.61152
PARD3	0.06	5.95	0.51	0.61155
TPM3P8	-0.05	5.76	-0.51	0.61165
IP6K1	-0.04	10.98	-0.51	0.61179
HNRNPA1P39	0.05	5.14	0.51	0.61189
LRP5L	0.06	6.99	0.51	0.61204
TRBJ1-2	-0.07	5.24	-0.51	0.61205
CCDC102B	-0.05	5.16	-0.51	0.61216
ELP4	0.04	7.22	0.51	0.61218

ZNF213-AS1	0.05	7.19	0.51	0.61218
THOC1	-0.04	7.78	-0.51	0.61238
AC012066.1	0.06	5.03	0.51	0.61256
KIAA1328	0.05	6.08	0.51	0.61267
SEM1P1	0.06	5.00	0.51	0.61271
USF1	-0.04	11.14	-0.51	0.61274
COLGALT1	-0.03	10.48	-0.51	0.61280
DHCR24	0.07	7.04	0.51	0.61286
NUP85	-0.03	9.00	-0.51	0.61293
LINC01135	-0.06	4.94	-0.51	0.61326
SPHK1	0.06	7.11	0.51	0.61333
SMN1	-0.06	6.04	-0.51	0.61333
RAB24	-0.07	7.86	-0.51	0.61333
SRF	0.03	9.92	0.51	0.61350
PTPN6	0.04	13.69	0.51	0.61351
IBA57	0.04	7.41	0.51	0.61363
VTI1BP1	-0.05	5.39	-0.51	0.61371
AC009951.6	-0.06	5.22	-0.51	0.61376
AC092919.3	0.05	5.51	0.51	0.61387
NUDT6	-0.05	5.13	-0.51	0.61411
KLF7	0.06	9.09	0.51	0.61421
BICRA	0.03	8.71	0.51	0.61429
PLPBP	-0.03	8.89	-0.51	0.61447
AL627309.7	-0.11	8.79	-0.51	0.61451
DNASE1	0.04	9.03	0.51	0.61460
GHITM	-0.03	10.38	-0.51	0.61460
ZNF682	-0.05	5.66	-0.51	0.61464
CEACAM8	-0.15	6.36	-0.51	0.61474
LINC00539	0.06	5.71	0.51	0.61475
AC008764.6	0.06	5.23	0.51	0.61477
MED4	-0.03	8.74	-0.51	0.61508
COG7	0.03	8.31	0.51	0.61526
INTS14	0.03	8.30	0.51	0.61546
MAN1B1-DT	0.05	5.54	0.51	0.61554
HMGB1P10	0.05	5.49	0.51	0.61556
TRAPPC2	-0.04	7.61	-0.51	0.61558
YWHAE	-0.04	11.02	-0.51	0.61559
LINC02384	0.08	6.67	0.50	0.61576
ETFBKMT	0.05	5.06	0.50	0.61617
AC108704.2	0.09	5.67	0.50	0.61617
MIEF2	0.04	7.12	0.50	0.61627
LINC01547	0.06	5.06	0.50	0.61633
C10orf88	-0.05	6.16	-0.50	0.61636
SYK	-0.04	12.00	-0.50	0.61639
TMC4	-0.09	6.29	-0.50	0.61641
Z97353.1	0.04	5.14	0.50	0.61663
EXTL3	-0.06	10.31	-0.50	0.61668
INO80C	0.04	7.20	0.50	0.61703
LEF1	0.08	10.14	0.50	0.61706
FICD	-0.04	6.66	-0.50	0.61715

KIF21B	-0.04	11.31	-0.50	0.61741
RPL34P6	0.05	5.51	0.50	0.61756
UBXN11	0.06	10.56	0.50	0.61758
IDNK	-0.05	6.33	-0.50	0.61769
IGKV1D-39	-0.09	6.62	-0.50	0.61786
SRSF11	0.05	10.09	0.50	0.61799
AC005070.3	0.08	5.29	0.50	0.61810
AP002761.4	-0.07	7.22	-0.50	0.61830
TLE3	-0.07	12.31	-0.50	0.61834
PSMG2	0.03	8.87	0.50	0.61839
AC018735.1	-0.05	5.11	-0.50	0.61841
C2orf69	-0.05	6.24	-0.50	0.61847
CDK2	-0.04	7.06	-0.50	0.61874
AC007383.1	0.06	5.52	0.50	0.61885
MTRNR2L6	-0.06	5.21	-0.50	0.61887
LRRC61	0.05	7.40	0.50	0.61893
PRKCZ-AS1	0.05	5.36	0.50	0.61894
AL121603.2	-0.05	6.02	-0.50	0.61907
TMEM170A	0.04	7.62	0.50	0.61917
CAB39L	-0.04	6.19	-0.50	0.61927
ACAD9	0.04	8.68	0.50	0.61928
TRBV23-1	0.07	5.19	0.50	0.61928
COQ10A	0.05	6.88	0.50	0.61931
UTP18	0.04	8.02	0.50	0.61951
SPCS2	0.04	8.12	0.50	0.61991
CCNJL	-0.10	9.61	-0.50	0.61991
TBC1D9B	-0.03	10.51	-0.50	0.61993
TNIP3	0.06	5.14	0.50	0.61995
TMCC1-AS1	0.06	5.06	0.50	0.61996
HAT1	-0.04	7.91	-0.50	0.62005
ZNF658	-0.05	5.20	-0.50	0.62008
USP4	-0.04	11.02	-0.50	0.62008
RBM23	-0.04	10.82	-0.50	0.62024
WWC2	-0.07	5.30	-0.50	0.62025
MYL6P4	0.06	5.13	0.50	0.62030
RGL3	-0.09	5.85	-0.50	0.62030
TOB2	-0.03	9.76	-0.50	0.62037
SETMAR	0.04	6.21	0.50	0.62040
LSG1	0.03	8.96	0.50	0.62040
BAIAP3	-0.10	8.61	-0.50	0.62060
QRSL1	-0.04	7.75	-0.50	0.62061
ACSM3	-0.05	6.49	-0.50	0.62073
DYNC2H1	-0.05	5.19	-0.50	0.62078
AL354798.1	0.05	5.04	0.50	0.62087
LINC02397	0.06	5.28	0.50	0.62100
EXOSC8	0.05	7.44	0.50	0.62120
UFSP2	0.05	7.29	0.50	0.62128
RAB11FIP5	0.07	7.90	0.50	0.62128
CMTM8	-0.08	5.78	-0.50	0.62130
DOLPP1	0.04	7.47	0.50	0.62133

SMIM26	0.05	7.12	0.50	0.62138
IGHV3-43	0.13	6.21	0.50	0.62141
ZBTB41	-0.05	5.82	-0.50	0.62156
LY6G5B	-0.07	6.34	-0.50	0.62157
SBNO1-AS1	0.06	5.35	0.50	0.62191
MNAT1	-0.05	5.69	-0.50	0.62197
IGHV1-2	-0.17	7.51	-0.50	0.62199
KIR3DL1	0.09	7.25	0.50	0.62213
GCNT1	-0.05	7.11	-0.50	0.62241
AGAP9	0.08	5.91	0.50	0.62243
PPIAP29	-0.05	6.77	-0.49	0.62270
HCG25	-0.06	5.01	-0.49	0.62277
SERTAD3	0.05	8.74	0.49	0.62283
SEMA4A	0.06	11.37	0.49	0.62296
RPL23AP82	0.05	6.67	0.49	0.62303
TMX2	0.03	9.21	0.49	0.62304
LENG8	-0.07	11.86	-0.49	0.62307
RPL7AP50	0.07	6.88	0.49	0.62350
RASGRF2	0.07	7.23	0.49	0.62352
ZNF292	0.05	7.94	0.49	0.62353
SAFB2	0.03	10.13	0.49	0.62404
HMOX1	0.06	10.35	0.49	0.62405
GRAMD2B	-0.05	5.61	-0.49	0.62411
ADAMTS10	-0.07	7.57	-0.49	0.62419
SEPTIN10	-0.05	5.20	-0.49	0.62424
WBP2	0.06	12.27	0.49	0.62428
GPR68	0.07	8.55	0.49	0.62434
HLA-B	0.05	16.85	0.49	0.62436
ADAM22	-0.06	5.26	-0.49	0.62439
ARSD	0.05	8.63	0.49	0.62440
H2BC8	0.07	6.42	0.49	0.62445
RUSC2	-0.06	7.15	-0.49	0.62450
CA4	0.11	8.02	0.49	0.62462
KIFC3	0.06	7.77	0.49	0.62462
ZNF185	-0.07	9.58	-0.49	0.62470
LINC01480	0.07	5.50	0.49	0.62486
STAT6	-0.05	13.13	-0.49	0.62491
PCTP	-0.05	8.55	-0.49	0.62507
LINC01270	-0.11	7.54	-0.49	0.62516
SLCO3A1	-0.05	10.72	-0.49	0.62522
TOMM20	0.03	9.45	0.49	0.62524
PAX8-AS1	-0.18	7.96	-0.49	0.62531
MUC12-AS1	0.06	4.97	0.49	0.62538
AC012498.2	-0.06	5.06	-0.49	0.62547
TRPM2	0.06	8.75	0.49	0.62550
AC004552.1	0.15	7.13	0.49	0.62550
SIDT2	0.06	10.67	0.49	0.62572
COPS3	0.03	9.15	0.49	0.62579
CYP2D8P	0.06	5.20	0.49	0.62598
PHF5A	0.04	8.27	0.49	0.62632

SLC27A3	-0.06	9.99	-0.49	0.62663
ZBED6CL	0.03	7.79	0.49	0.62666
NEURL1B	0.06	5.09	0.49	0.62674
PA2G4P2	0.04	5.07	0.49	0.62685
NSMCE4A	-0.03	8.06	-0.49	0.62698
UTP11	0.03	7.89	0.49	0.62701
SEPTIN8	0.05	6.88	0.49	0.62726
BICDL2	-0.11	5.88	-0.49	0.62730
NUAK2	-0.05	10.63	-0.49	0.62766
ZNF800	-0.03	7.94	-0.49	0.62771
CEBPB-AS1	0.05	5.46	0.49	0.62771
TTC9C	0.03	8.18	0.49	0.62771
ACY3	0.06	5.32	0.49	0.62799
YBEY	-0.06	6.95	-0.49	0.62817
AKR1C3	0.08	7.45	0.49	0.62822
LRRN1	-0.09	5.29	-0.49	0.62829
ODC1	0.06	10.04	0.49	0.62849
GPN1	0.04	8.16	0.49	0.62853
PSMG1	0.05	6.79	0.49	0.62859
ADARB1	0.04	8.71	0.49	0.62870
SASH3	0.03	11.92	0.49	0.62890
AP005329.3	-0.05	5.55	-0.49	0.62901
ACVRL1	-0.06	5.69	-0.49	0.62906
TDRP	-0.07	5.33	-0.49	0.62920
SMAD3	-0.03	9.71	-0.49	0.62926
ELMO3	0.06	7.13	0.49	0.62928
TTC21A	-0.09	6.92	-0.49	0.62942
AC135050.6	-0.04	7.11	-0.49	0.62950
KLRD1	-0.07	9.77	-0.49	0.62955
MSRA	-0.05	8.48	-0.48	0.62979
AC090543.3	0.12	6.52	0.48	0.62992
CYREN	0.03	8.55	0.48	0.63000
NPIPA9	0.05	6.13	0.48	0.63002
IER5	-0.04	9.58	-0.48	0.63004
PRR13	0.05	10.77	0.48	0.63006
DUSP5	-0.07	7.86	-0.48	0.63010
ATP6V0A1	-0.05	9.89	-0.48	0.63011
POTEE	0.08	7.62	0.48	0.63034
MGA	-0.04	8.07	-0.48	0.63051
KLHL21	-0.05	10.69	-0.48	0.63051
XPA	0.04	7.10	0.48	0.63060
AC018647.2	-0.05	6.37	-0.48	0.63070
ZNF76	0.04	9.25	0.48	0.63073
CUL9	-0.04	9.60	-0.48	0.63096
TRAV4	0.06	6.57	0.48	0.63102
AC020688.1	0.05	5.61	0.48	0.63119
POLD4	0.04	7.70	0.48	0.63125
LAMC3	0.08	5.15	0.48	0.63135
STRBP	0.06	7.51	0.48	0.63151
PPM1F	-0.05	11.28	-0.48	0.63151

CCT7P1	0.05	5.09	0.48	0.63169
NT5DC4	-0.08	5.77	-0.48	0.63177
ZNF354B	-0.04	6.08	-0.48	0.63179
MSRB1P1	-0.06	5.93	-0.48	0.63185
AL162431.1	0.05	5.61	0.48	0.63190
AC122688.1	-0.06	5.09	-0.48	0.63192
TMEM150B	0.07	6.92	0.48	0.63205
GOLT1B	-0.04	6.87	-0.48	0.63209
AC093673.1	0.05	7.74	0.48	0.63210
AZIN2	0.06	6.01	0.48	0.63214
USP8	0.05	8.74	0.48	0.63234
MYL12A	-0.04	12.29	-0.48	0.63240
NUDCD1	-0.04	6.12	-0.48	0.63241
PAQR7	0.05	7.68	0.48	0.63247
ATG2A	-0.06	10.69	-0.48	0.63248
TMED2-DT	-0.05	5.16	-0.48	0.63259
CCDC183-AS1	-0.05	5.34	-0.48	0.63280
STMP1	0.06	9.07	0.48	0.63293
ADCK1	0.05	7.38	0.48	0.63302
FBXO42	-0.02	9.03	-0.48	0.63306
PFKM	0.03	7.48	0.48	0.63321
ZFAT	0.04	7.78	0.48	0.63323
DPAGT1	0.04	8.64	0.48	0.63326
TAF12	0.03	8.58	0.48	0.63330
CEACAM4	-0.09	9.57	-0.48	0.63334
NATD1	-0.09	10.27	-0.48	0.63336
SPAG5	-0.03	6.20	-0.48	0.63339
LINC01359	-0.06	5.72	-0.48	0.63343
CORO6	-0.06	5.66	-0.48	0.63364
C8G	0.06	5.12	0.48	0.63370
PCSK6	0.10	7.23	0.48	0.63373
AC107027.3	-0.06	4.96	-0.48	0.63375
SPN	0.05	11.90	0.48	0.63399
OR2W3	0.13	8.18	0.48	0.63408
AC007342.7	-0.06	5.15	-0.48	0.63416
CHIT1	-0.12	6.14	-0.48	0.63418
HDGFP1	-0.06	5.23	-0.48	0.63424
FASTKD2	-0.03	7.90	-0.48	0.63431
TRAPPC3L	-0.06	5.25	-0.48	0.63435
ARHGAP24	-0.07	7.27	-0.48	0.63455
WBP2NL	0.05	5.13	0.48	0.63455
RPL7AP11	0.06	6.16	0.48	0.63487
ANP32AP1	-0.06	5.77	-0.48	0.63496
ARAP3	-0.06	10.72	-0.48	0.63497
IGKV4-1	0.09	9.31	0.48	0.63510
SNRPEP4	-0.05	5.13	-0.48	0.63517
LTB4R	-0.06	10.13	-0.48	0.63539
SEC14L5	-0.09	6.16	-0.48	0.63545
SIPA1L3	0.05	8.96	0.48	0.63576
TREML3P	0.11	6.43	0.48	0.63590

NPAS2	0.07	5.26	0.48	0.63593
CALM1P1	-0.06	5.93	-0.48	0.63597
ZW10	-0.03	6.80	-0.48	0.63609
TRMT10B	0.04	7.22	0.48	0.63621
COG1	0.03	9.18	0.48	0.63627
NXPE3	0.04	8.66	0.48	0.63632
NOM1	-0.04	7.57	-0.48	0.63658
SLX4	-0.03	7.97	-0.48	0.63666
SNHG1	-0.04	8.67	-0.48	0.63671
AC092670.1	0.04	5.36	0.48	0.63675
AC245407.2	0.08	6.62	0.47	0.63688
CBFA2T3	0.04	9.81	0.47	0.63691
MTRNR2L10	0.05	5.23	0.47	0.63704
ACIN1	-0.03	11.55	-0.47	0.63711
MED21	0.04	6.60	0.47	0.63722
IGKV3D-20	0.09	6.75	0.47	0.63743
GRK6P1	0.07	6.95	0.47	0.63758
CHST13	-0.07	7.09	-0.47	0.63760
MTRF1	-0.05	6.52	-0.47	0.63761
HLA-G	0.12	10.87	0.47	0.63772
PSMC2	0.03	9.02	0.47	0.63785
VIL1	-0.07	5.92	-0.47	0.63793
YBX3P1	0.11	7.36	0.47	0.63800
UTP23	0.04	7.19	0.47	0.63811
GTF2IP12	-0.07	7.02	-0.47	0.63824
CCDC154	-0.06	5.72	-0.47	0.63832
PRPSAP2	-0.03	8.34	-0.47	0.63840
PUM3	0.03	7.82	0.47	0.63847
NDRG3	0.03	9.07	0.47	0.63860
TTLL11	0.04	5.88	0.47	0.63877
DDX12P	-0.06	5.78	-0.47	0.63882
ZNF134	-0.03	7.98	-0.47	0.63891
RFC2	0.04	8.46	0.47	0.63902
HSF2	0.05	6.67	0.47	0.63932
BTBD8	0.05	5.06	0.47	0.63944
POLR2K	-0.04	7.47	-0.47	0.63968
CRYBB2P1	-0.06	7.14	-0.47	0.63983
SNRPGP10	0.05	5.35	0.47	0.64011
HSD3B7	0.06	5.91	0.47	0.64019
ZSWIM8	-0.05	10.81	-0.47	0.64025
AC138028.6	0.05	5.12	0.47	0.64042
ZNF786	0.04	7.33	0.47	0.64048
TRGV5	-0.09	5.18	-0.47	0.64054
AC079174.2	-0.06	5.51	-0.47	0.64055
MGST3	0.05	8.59	0.47	0.64055
NANP	-0.04	5.40	-0.47	0.64062
SUPT6H	0.02	11.34	0.47	0.64063
CDK5RAP1	0.03	8.37	0.47	0.64069
SPRYD7	-0.04	5.46	-0.47	0.64086
EXT2	-0.02	8.98	-0.47	0.64114

WASH9P	-0.07	6.81	-0.47	0.64115
UBB	0.10	14.50	0.47	0.64123
YY1AP1	-0.04	10.43	-0.47	0.64128
AAMDC	0.06	6.26	0.47	0.64152
IKBKGP1	0.05	5.69	0.47	0.64165
GALNT2	0.03	9.82	0.47	0.64173
CARNMT1	-0.05	5.93	-0.47	0.64192
INO80	-0.03	9.26	-0.47	0.64220
FCHSD1	0.04	9.91	0.47	0.64221
ALKBH6	-0.05	5.74	-0.47	0.64227
PPIAP22	0.06	9.31	0.47	0.64259
MFSD8	-0.05	6.87	-0.47	0.64261
CELA1	0.06	5.01	0.47	0.64262
AC133644.3	0.04	5.04	0.47	0.64295
THEM4	0.05	8.68	0.47	0.64301
LAT	0.08	6.59	0.47	0.64315
EEF1A1P22	-0.04	7.55	-0.47	0.64319
AL353708.3	-0.06	5.08	-0.47	0.64319
CARS2	-0.04	10.75	-0.47	0.64326
FUT10	-0.06	5.63	-0.47	0.64339
NKIRAS1	0.05	5.44	0.47	0.64362
JUNB	0.07	11.01	0.46	0.64402
RBL1	-0.05	6.89	-0.46	0.64417
HPF1	0.04	7.00	0.46	0.64432
ZNF552	0.06	7.25	0.46	0.64442
UAP1	-0.04	7.08	-0.46	0.64451
LMAN2L	0.03	7.84	0.46	0.64467
PTPDC1	-0.06	5.26	-0.46	0.64468
LEAP2	-0.05	5.21	-0.46	0.64474
NOLC1	-0.03	9.61	-0.46	0.64476
C17orf75	0.04	6.86	0.46	0.64482
UBE2L6	-0.08	11.53	-0.46	0.64484
IGHV3-49	0.08	6.24	0.46	0.64491
PRDM15	-0.05	7.57	-0.46	0.64493
AC008875.3	0.03	9.44	0.46	0.64496
RAB11A	-0.03	9.49	-0.46	0.64499
DERA	-0.04	7.59	-0.46	0.64507
AL139384.1	0.05	6.35	0.46	0.64529
C6orf163	0.05	5.03	0.46	0.64532
PRR13P5	-0.06	7.74	-0.46	0.64535
AC073073.1	-0.06	5.65	-0.46	0.64566
APOL1	-0.07	10.11	-0.46	0.64585
CRYZL1	0.05	7.16	0.46	0.64596
GPRC5C	-0.08	5.15	-0.46	0.64603
ELOC	0.03	7.95	0.46	0.64613
KCTD11	0.03	7.97	0.46	0.64619
ZFYVE1	-0.03	9.26	-0.46	0.64622
WNT11	-0.05	5.03	-0.46	0.64625
DLST	-0.02	9.89	-0.46	0.64628
AP000766.1	0.04	5.11	0.46	0.64629

TRNP1	-0.05	5.01	-0.46	0.64633
LINC00426	0.04	7.14	0.46	0.64634
AP001160.3	0.05	5.00	0.46	0.64646
NSUN5P2	0.08	8.53	0.46	0.64653
SMU1	0.02	8.96	0.46	0.64674
AC025171.2	0.04	6.67	0.46	0.64692
PAK1IP1	-0.03	6.67	-0.46	0.64715
NR1H3	-0.05	7.07	-0.46	0.64718
KIR2DL3	-0.08	6.36	-0.46	0.64720
ACTR8	0.03	7.63	0.46	0.64735
HMGN1	0.03	10.11	0.46	0.64739
DUSP7	0.03	9.30	0.46	0.64751
SMARCD3	0.05	9.61	0.46	0.64775
CD27-AS1	0.04	6.92	0.46	0.64781
PLA2G15	0.05	8.57	0.46	0.64795
SERPINI1	-0.05	5.61	-0.46	0.64813
PRDX4	0.06	7.38	0.46	0.64825
DOC2GP	-0.07	5.63	-0.46	0.64829
METTL9	-0.04	10.17	-0.46	0.64882
AC139530.1	-0.05	6.04	-0.46	0.64898
CHPT1	-0.08	8.78	-0.46	0.64902
POLRMTP1	0.06	6.11	0.46	0.64904
IGHV4-59	0.10	7.57	0.46	0.64905
SDR42E1	-0.05	5.80	-0.46	0.64929
POTEJ	0.05	6.31	0.46	0.64931
MMP11	0.06	5.79	0.46	0.64933
CLCN2	0.05	6.04	0.46	0.64944
TMOD4	-0.05	6.02	-0.46	0.64950
TRIM38	-0.04	10.67	-0.46	0.64962
SIGLEC10	0.06	11.11	0.46	0.64966
ZNF529	0.04	6.35	0.46	0.64979
LGALS9C	0.09	6.56	0.46	0.64986
CCNH	-0.03	7.61	-0.46	0.64997
AC025181.1	0.05	5.11	0.46	0.65023
SULT1A2	-0.08	6.20	-0.46	0.65031
RPS15AP11	0.05	5.42	0.46	0.65046
AC093627.4	-0.05	6.51	-0.46	0.65049
GPR108	0.04	10.76	0.46	0.65060
AC012313.1	-0.04	5.99	-0.46	0.65074
AL080243.2	0.05	6.99	0.46	0.65090
CLEC10A	0.10	8.65	0.45	0.65105
FAN1	0.06	7.80	0.45	0.65105
IGHV1-18	-0.10	7.77	-0.45	0.65129
SDC2	-0.06	5.13	-0.45	0.65135
CERK	0.03	10.89	0.45	0.65143
ECD	-0.03	8.14	-0.45	0.65144
CENPV	0.05	6.45	0.45	0.65161
ALG9	0.04	5.56	0.45	0.65166
LINC00937	-0.10	7.12	-0.45	0.65202
ZNRF1	-0.05	7.11	-0.45	0.65210

SPECC1L	0.03	8.87	0.45	0.65219
AC010878.1	-0.05	5.29	-0.45	0.65220
RFX1	-0.03	9.52	-0.45	0.65240
TOR1A	-0.03	8.88	-0.45	0.65257
UMPS	0.04	7.76	0.45	0.65277
AC104809.2	-0.11	7.60	-0.45	0.65294
PCCA	-0.04	6.76	-0.45	0.65296
NR4A1	-0.08	7.53	-0.45	0.65296
ITPR3	-0.04	10.05	-0.45	0.65304
LMLN	0.05	5.45	0.45	0.65304
RRAS	0.06	8.81	0.45	0.65310
ATP6V1G1	-0.03	10.27	-0.45	0.65324
RCSD1	0.03	11.58	0.45	0.65337
KDM7A-DT	0.07	7.65	0.45	0.65345
PAXIP1-AS1	0.04	6.47	0.45	0.65347
RHOA	-0.03	13.10	-0.45	0.65359
FUZ	0.04	8.21	0.45	0.65359
LIMS1	0.04	9.65	0.45	0.65395
HSPD1P1	-0.05	5.78	-0.45	0.65414
MUS81	-0.03	9.38	-0.45	0.65414
AP1AR	-0.05	6.22	-0.45	0.65417
MXI1	0.08	9.83	0.45	0.65438
CCDC134	0.05	5.76	0.45	0.65450
CENPT	0.05	9.56	0.45	0.65455
AC118758.3	-0.05	5.19	-0.45	0.65458
C1orf21	-0.07	7.83	-0.45	0.65466
CCDC18-AS1	0.08	7.44	0.45	0.65467
AC067930.3	-0.11	5.09	-0.45	0.65473
AC012645.1	-0.08	7.40	-0.45	0.65476
INPP5D	-0.03	12.53	-0.45	0.65482
CCR12P	0.09	6.26	0.45	0.65483
TTC28-AS1	-0.04	7.27	-0.45	0.65496
ZNF227	-0.03	7.61	-0.45	0.65509
SERF1B	-0.04	5.05	-0.45	0.65510
RFESD	-0.05	5.27	-0.45	0.65511
AL683807.1	-0.08	5.64	-0.45	0.65526
PPP1R16A	0.04	7.00	0.45	0.65538
TGFBR3	-0.06	8.25	-0.45	0.65558
RMND1	0.04	6.96	0.45	0.65558
ZC3HAV1	-0.04	10.52	-0.45	0.65561
NEU1	0.04	9.71	0.45	0.65564
AC008610.1	0.05	5.52	0.45	0.65583
CYB5A	-0.04	6.87	-0.45	0.65603
AC048341.1	-0.05	5.59	-0.45	0.65607
CCNI2	-0.05	5.42	-0.45	0.65611
SGTB	-0.04	7.36	-0.45	0.65614
SKI	-0.03	10.23	-0.45	0.65621
C17orf67	0.04	5.96	0.45	0.65627
OSER1	-0.04	9.69	-0.45	0.65642
EEF1A1P19	0.06	8.62	0.45	0.65648

CHIC1	0.05	6.19	0.45	0.65654
HSF4	0.06	6.40	0.45	0.65655
CEP164	-0.03	9.20	-0.45	0.65655
ADAM15	-0.05	10.19	-0.45	0.65663
LGALS8	-0.04	9.63	-0.45	0.65672
PRICKLE3	0.03	7.93	0.45	0.65677
RGS6	0.07	6.50	0.45	0.65685
LMNTD2-AS1	0.07	5.92	0.45	0.65702
ZBTB25	-0.05	7.58	-0.45	0.65721
GNL2	0.03	8.73	0.45	0.65724
RORC	0.07	7.04	0.45	0.65746
ADAMTS13	-0.06	5.28	-0.45	0.65763
AC011468.1	0.05	4.98	0.45	0.65785
BOLA2B	0.04	5.10	0.45	0.65795
SMIM24	0.09	6.79	0.45	0.65809
CDCA3	-0.05	5.14	-0.44	0.65844
TSPAN3	-0.05	8.61	-0.44	0.65853
AC111182.1	0.05	5.22	0.44	0.65855
TRIM16L	-0.05	5.42	-0.44	0.65864
AC073465.1	-0.06	5.19	-0.44	0.65868
VDAC1P1	-0.05	5.22	-0.44	0.65875
MBLAC2	-0.04	5.59	-0.44	0.65884
ASGR1	0.07	8.27	0.44	0.65893
ZNF56	0.05	5.23	0.44	0.65897
ZNF710-AS1	0.04	7.05	0.44	0.65901
BRI3P1	-0.04	5.27	-0.44	0.65919
PRKD2	0.04	12.15	0.44	0.65923
ENOSF1	-0.06	7.55	-0.44	0.65938
UBE2R2	-0.04	10.95	-0.44	0.65941
SETBP1	0.05	6.60	0.44	0.65976
MGAT5	-0.03	9.81	-0.44	0.65979
ZNF398	0.03	8.22	0.44	0.65993
AC245140.2	-0.06	5.26	-0.44	0.65999
PHETA1	0.05	7.34	0.44	0.66003
MRPL15	0.04	7.44	0.44	0.66007
POP1	0.04	6.55	0.44	0.66037
HIKESHI	0.03	7.89	0.44	0.66038
MYL12B	-0.04	11.40	-0.44	0.66044
LINC01679	0.06	5.16	0.44	0.66055
EFCAB11	-0.05	5.15	-0.44	0.66056
PRAMENP	0.06	5.21	0.44	0.66064
HLA-DRA	-0.05	13.83	-0.44	0.66077
NKAPD1	-0.03	7.74	-0.44	0.66077
GPAM	-0.04	5.87	-0.44	0.66087
FCGRT	0.05	12.55	0.44	0.66103
LRRC8A	0.04	9.03	0.44	0.66108
CAMK1D	0.04	10.54	0.44	0.66110
ARMH3	-0.03	8.49	-0.44	0.66134
LRRC41	0.03	9.46	0.44	0.66158
P2RY10	-0.05	8.49	-0.44	0.66160

SDHB	-0.03	9.55	-0.44	0.66160
POLA1	-0.04	6.55	-0.44	0.66189
TTC25	0.06	5.45	0.44	0.66201
ITPKB	-0.03	10.75	-0.44	0.66227
RPL12P8	0.05	5.51	0.44	0.66231
AC084880.1	0.05	5.86	0.44	0.66264
STX16-NPEPL1	-0.07	6.62	-0.44	0.66269
DCTD	0.03	9.19	0.44	0.66270
ZNF683	-0.15	8.18	-0.44	0.66273
MSS51	-0.05	5.69	-0.44	0.66284
PHLDB1	-0.05	4.96	-0.44	0.66310
CCNG1	-0.04	8.40	-0.44	0.66329
IFT46	0.03	6.93	0.44	0.66336
HOXB2	0.07	6.66	0.44	0.66343
LINC01619	-0.06	5.58	-0.44	0.66346
CMC2	-0.05	7.11	-0.44	0.66376
FAM228B	0.05	6.33	0.44	0.66382
ACTBP8	0.12	6.70	0.44	0.66391
SAMHD1	-0.04	12.66	-0.44	0.66394
ZNF264	0.03	8.46	0.44	0.66407
NFYC	0.03	10.29	0.44	0.66419
ZYG11B	-0.04	7.16	-0.44	0.66427
H2BC15	-0.06	5.22	-0.44	0.66457
DOCK8-AS1	-0.04	5.54	-0.44	0.66461
FTH1P8	0.07	8.70	0.44	0.66468
MS4A2	-0.07	5.92	-0.44	0.66492
SDF2	-0.03	9.29	-0.44	0.66503
USB1	-0.05	10.77	-0.44	0.66509
ACTR6	-0.04	6.62	-0.44	0.66512
RFPL2	0.06	5.05	0.43	0.66540
TSPAN9	0.07	7.29	0.43	0.66544
NRXN2	-0.06	5.13	-0.43	0.66545
DET1	0.05	5.07	0.43	0.66556
TAGLN2P1	-0.07	8.12	-0.43	0.66557
DHTKD1	0.04	8.74	0.43	0.66562
MPPE1	-0.04	9.96	-0.43	0.66568
POTEF	0.08	7.78	0.43	0.66581
ACTR5	0.04	7.74	0.43	0.66596
COPS8	0.03	7.77	0.43	0.66600
SLC30A4	-0.06	5.43	-0.43	0.66614
REST	0.04	8.31	0.43	0.66622
GLB1L2	-0.09	6.11	-0.43	0.66627
ZCCHC14	-0.04	7.66	-0.43	0.66658
SYNM	0.07	6.44	0.43	0.66659
ABCF2P1	-0.05	5.12	-0.43	0.66659
SYTL3	-0.05	9.79	-0.43	0.66667
C2CD2L	0.04	9.64	0.43	0.66673
DECR2	0.05	5.98	0.43	0.66685
PPCDC	-0.05	9.32	-0.43	0.66694
PPP1R3E	0.06	8.04	0.43	0.66695

GEMIN2	-0.04	6.16	-0.43	0.66697
AGL	-0.04	6.68	-0.43	0.66698
RPS26P11	0.09	5.85	0.43	0.66719
AC093627.5	0.04	5.68	0.43	0.66735
GADD45A	-0.07	7.17	-0.43	0.66757
CYP4F22	0.06	6.65	0.43	0.66769
EVA1B	0.05	6.69	0.43	0.66789
TNFRSF1B	0.04	13.89	0.43	0.66789
RPS26P47	0.09	6.05	0.43	0.66811
C19orf38	0.05	10.53	0.43	0.66816
STARD7	0.03	10.21	0.43	0.66819
LINC00957	0.04	7.27	0.43	0.66819
ADHFE1	-0.05	5.92	-0.43	0.66822
ZNF513	0.04	7.94	0.43	0.66825
PPME1	0.03	8.42	0.43	0.66845
TACSTD2	-0.08	5.24	-0.43	0.66859
ABCB4	0.06	5.65	0.43	0.66864
ALPL	-0.11	12.29	-0.43	0.66867
AC011933.1	0.05	5.15	0.43	0.66875
CNIH1	-0.04	7.90	-0.43	0.66876
RAD51B	-0.04	5.65	-0.43	0.66880
UBE2Q1	0.02	10.48	0.43	0.66880
PADI6	0.08	4.92	0.43	0.66883
SRSF3	-0.02	11.06	-0.43	0.66894
LINC02656	-0.06	5.14	-0.43	0.66894
RTCA	-0.03	7.60	-0.43	0.66898
ITIH1	0.05	5.01	0.43	0.66902
NRAV	-0.04	5.64	-0.43	0.66918
LIM2	0.05	4.99	0.43	0.66922
AC025262.2	0.06	5.20	0.43	0.66923
SRR	0.05	5.74	0.43	0.66933
GSN	-0.05	11.38	-0.43	0.66936
YPEL1	-0.04	8.20	-0.43	0.66937
HENMT1	0.04	8.28	0.43	0.66940
SNCA	0.11	9.77	0.43	0.66944
EIF3LP3	0.05	5.98	0.43	0.66954
TXNL4B	-0.03	8.09	-0.43	0.66955
CCNL2	-0.05	10.81	-0.43	0.66956
COX14	0.05	8.41	0.43	0.66959
AC011374.2	-0.05	5.14	-0.43	0.66966
AC135983.3	-0.06	5.68	-0.43	0.66974
AC025682.2	-0.06	5.63	-0.43	0.66977
ZNF195	-0.04	6.96	-0.43	0.66990
ABCE1	-0.04	7.69	-0.43	0.66996
CCDC153	-0.06	6.54	-0.43	0.67016
CROT	0.04	6.52	0.43	0.67055
TMEM63A	0.03	10.71	0.43	0.67063
LINC00987	-0.05	5.14	-0.43	0.67091
AC123595.1	0.06	5.25	0.43	0.67097
ARIH2OS	0.04	5.70	0.43	0.67104

TRBV12-4	0.07	6.64	0.43	0.67105
DCAF6	-0.04	8.66	-0.43	0.67119
EME2	0.05	7.75	0.43	0.67129
UBAP2	-0.03	8.68	-0.43	0.67135
DPF3	0.05	5.22	0.43	0.67139
PSMD8P1	-0.05	5.36	-0.43	0.67141
TPD52L2	-0.03	11.20	-0.43	0.67152
S100A10	0.05	11.15	0.43	0.67218
AC005306.1	-0.06	8.50	-0.43	0.67220
MIR3945HG	-0.09	6.55	-0.43	0.67223
PALB2	-0.05	6.74	-0.43	0.67223
SART3	-0.03	9.75	-0.43	0.67227
MIR600HG	0.06	6.77	0.43	0.67228
FHL1	0.05	7.88	0.43	0.67228
PSMD11	-0.02	9.09	-0.43	0.67230
PABPC1P4	-0.06	8.13	-0.43	0.67236
ZNF831	0.04	8.62	0.43	0.67245
AL353660.1	-0.06	5.60	-0.43	0.67247
ARL8A	0.04	9.84	0.43	0.67256
TMEM158	0.08	6.55	0.42	0.67281
LRRC37A4P	-0.07	6.94	-0.42	0.67296
FMNL1	-0.04	13.93	-0.42	0.67300
TRIM65	0.03	8.52	0.42	0.67317
LINC02018	0.05	5.07	0.42	0.67322
HAUS5	0.04	7.71	0.42	0.67325
VAV1	-0.03	11.42	-0.42	0.67353
NEURL1	0.08	7.25	0.42	0.67360
GMFG	0.05	12.57	0.42	0.67366
SLC44A2	-0.04	12.93	-0.42	0.67398
SEL1L3	-0.04	9.41	-0.42	0.67430
CDC42EP4	-0.04	7.28	-0.42	0.67441
TOMM70	-0.03	8.03	-0.42	0.67443
NDUFV2-AS1	0.04	5.69	0.42	0.67469
WDR90	-0.05	6.43	-0.42	0.67472
PPP1R12A	0.06	9.80	0.42	0.67476
PMEPA1	0.06	6.57	0.42	0.67534
MAGOH2P	0.05	5.26	0.42	0.67537
TIAM1	-0.05	8.69	-0.42	0.67539
SLIRP	0.04	7.60	0.42	0.67539
TAF9B	-0.04	6.37	-0.42	0.67564
KCNQ1	0.03	9.72	0.42	0.67566
RHOBTB3	-0.06	5.31	-0.42	0.67569
AL160191.2	0.06	5.39	0.42	0.67588
SRSF4	0.03	10.35	0.42	0.67589
KANK2	0.10	6.39	0.42	0.67591
TGDS	0.05	6.11	0.42	0.67598
RCL1	0.03	6.76	0.42	0.67629
IGKV1-39	-0.08	6.55	-0.42	0.67638
TRAV12-1	-0.06	6.38	-0.42	0.67680
YWHAH	-0.03	10.10	-0.42	0.67691

WWP2	-0.03	11.40	-0.42	0.67693
CHKB	0.05	6.05	0.42	0.67705
PAFAH2	-0.03	8.68	-0.42	0.67716
AKR1C1	0.06	5.04	0.42	0.67767
VPS52	-0.03	9.38	-0.42	0.67774
RPL7L1	0.03	9.39	0.42	0.67785
SNAPC3	-0.04	7.03	-0.42	0.67788
EIF3CL	0.06	7.79	0.42	0.67804
AP000763.3	0.06	5.90	0.42	0.67833
CES1	-0.10	8.31	-0.42	0.67844
CELSR1	0.06	6.21	0.42	0.67847
ABHD11	0.04	7.86	0.42	0.67853
SVIL-AS1	-0.04	6.77	-0.42	0.67863
POLR3B	-0.04	6.67	-0.42	0.67866
PSMC1	0.03	9.04	0.42	0.67867
ARMC2	-0.04	5.46	-0.42	0.67872
DCAF16	-0.03	7.47	-0.42	0.67927
AC023512.1	0.05	5.72	0.42	0.67929
PCNX3	-0.03	10.35	-0.42	0.67943
PGRMC2	-0.03	8.17	-0.42	0.67945
AC099494.2	0.05	5.08	0.42	0.67946
STIP1P3	0.05	5.78	0.42	0.67947
AC139795.1	-0.06	5.49	-0.42	0.67965
PGRMC1	-0.05	8.87	-0.41	0.67992
MLX	-0.03	10.33	-0.41	0.68012
TSTD3	0.05	5.16	0.41	0.68051
CCDC90B	0.03	7.49	0.41	0.68060
LPXN	0.03	10.11	0.41	0.68060
CTSH	0.05	11.06	0.41	0.68061
LCN2	0.13	9.08	0.41	0.68073
SLC33A1	0.04	6.79	0.41	0.68076
YIPF6	-0.03	7.43	-0.41	0.68077
NBPF15	-0.07	7.48	-0.41	0.68098
RASL11A	-0.08	6.26	-0.41	0.68101
AL391832.2	-0.06	6.39	-0.41	0.68107
RSRC1	-0.04	7.10	-0.41	0.68110
LINC00324	0.03	8.21	0.41	0.68110
NAPB	-0.03	6.79	-0.41	0.68110
KCNE1	-0.08	6.80	-0.41	0.68114
RBM17	-0.02	9.33	-0.41	0.68124
TMEM176B	0.17	9.58	0.41	0.68132
C2CD4D-AS1	-0.05	5.00	-0.41	0.68133
AL161421.1	0.05	5.00	0.41	0.68158
PPP2R3C	-0.04	8.77	-0.41	0.68161
NPM1P27	0.06	7.67	0.41	0.68166
BAZ2B	0.05	9.01	0.41	0.68173
IMPA2	0.05	10.05	0.41	0.68176
LAG3	0.08	8.01	0.41	0.68190
ORC5	-0.03	7.09	-0.41	0.68207
AMER1	-0.04	6.38	-0.41	0.68215

HDAC7	0.03	11.55	0.41	0.68232
ZNF829	0.04	5.56	0.41	0.68238
AKIP1	0.04	6.56	0.41	0.68254
NEK11	-0.04	5.16	-0.41	0.68262
NDUFB6	0.05	8.03	0.41	0.68270
TMED8	-0.06	7.61	-0.41	0.68293
EIF4E	-0.02	7.15	-0.41	0.68311
PILRB	-0.08	7.17	-0.41	0.68339
G0S2	-0.09	6.03	-0.41	0.68361
IRF1	-0.05	13.06	-0.41	0.68363
CIT	-0.05	5.23	-0.41	0.68369
DGKE	-0.04	7.08	-0.41	0.68375
INTS7	-0.03	7.20	-0.41	0.68403
POR	0.05	10.70	0.41	0.68405
IGKV2D-28	-0.08	5.88	-0.41	0.68407
MRPL50	-0.03	6.99	-0.41	0.68433
GPR25	0.05	5.26	0.41	0.68462
PLEKHA3	0.04	6.96	0.41	0.68468
STRADB	0.11	9.70	0.41	0.68493
LINC02693	0.04	6.40	0.41	0.68503
B3GNT7	-0.06	6.37	-0.41	0.68512
BTBD6P1	-0.05	5.16	-0.41	0.68515
RAB30-DT	0.04	6.26	0.41	0.68526
BRCC3	0.03	7.42	0.41	0.68539
ZNF319	-0.03	9.26	-0.41	0.68542
ADGRG1	-0.07	11.69	-0.41	0.68556
TXLNA	0.02	9.78	0.41	0.68565
AC245884.8	0.06	5.29	0.41	0.68568
AL513318.1	0.05	5.33	0.41	0.68568
HSPA7	-0.07	8.46	-0.41	0.68577
FAM126B	0.07	9.22	0.41	0.68584
AC018521.5	0.05	5.28	0.41	0.68590
MYL6P1	0.04	5.56	0.41	0.68596
TMEM184B	0.04	9.81	0.41	0.68602
IFT88	0.04	6.15	0.41	0.68630
TFAP2E	-0.03	5.73	-0.41	0.68640
IGHV2-5	0.09	6.48	0.41	0.68652
TRIM3	0.04	7.40	0.41	0.68668
SHC1	-0.02	9.72	-0.41	0.68675
MGRN1	0.04	10.93	0.41	0.68700
IFNLR1	-0.07	6.37	-0.41	0.68712
AC093726.2	-0.05	5.21	-0.40	0.68737
SERPINB10	-0.06	5.12	-0.40	0.68745
ATF7-NPFF	-0.05	5.16	-0.40	0.68751
AC084824.6	-0.04	5.04	-0.40	0.68772
AC020658.5	-0.06	6.02	-0.40	0.68774
ITGAV	-0.04	6.01	-0.40	0.68787
RASA4B	-0.05	6.29	-0.40	0.68793
LRP10	-0.05	13.36	-0.40	0.68801
PINK1	0.06	11.13	0.40	0.68809

MIR4435-2HG	0.05	7.67	0.40	0.68815
TMEM63B	0.06	8.84	0.40	0.68825
YEATS2	-0.02	8.88	-0.40	0.68826
CLEC1B	0.06	6.45	0.40	0.68826
PDK1	-0.05	7.45	-0.40	0.68827
SYVN1	0.03	10.26	0.40	0.68873
TIE1	0.05	5.21	0.40	0.68874
HCK	-0.05	12.79	-0.40	0.68888
RTEL1-TNFRSF6B	-0.05	5.11	-0.40	0.68891
TROAP	0.04	5.19	0.40	0.68897
NOCT	0.05	5.07	0.40	0.68903
KMT5A	0.03	8.10	0.40	0.68906
FANCD2	-0.04	7.41	-0.40	0.68934
JAKMIP2	-0.05	6.29	-0.40	0.68935
ZNF439	0.05	6.61	0.40	0.68940
AP4B1	-0.03	9.09	-0.40	0.68945
BRD3	-0.03	9.68	-0.40	0.68955
GNGT2	0.05	7.17	0.40	0.68958
DHX32	0.04	6.91	0.40	0.68979
NCF1	-0.07	12.88	-0.40	0.68980
TRAV35	0.04	5.47	0.40	0.68980
IGKV1D-16	0.06	5.69	0.40	0.68985
SMOX	0.08	9.34	0.40	0.68997
SKAP2	-0.06	9.73	-0.40	0.69001
CRYBG2	-0.05	5.95	-0.40	0.69008
SOCS1	-0.06	6.64	-0.40	0.69012
EIF4A1P4	0.04	6.04	0.40	0.69016
RRP1B	-0.04	8.67	-0.40	0.69023
PDLIM5	0.04	8.46	0.40	0.69038
SPOCD1	-0.07	6.70	-0.40	0.69050
AC005332.4	0.04	6.77	0.40	0.69081
MACROD2	0.09	5.55	0.40	0.69087
H2AC18	-0.05	5.56	-0.40	0.69098
AL355001.2	0.04	5.06	0.40	0.69104
HNRNPA1P48	0.06	8.06	0.40	0.69111
RRAGC	-0.03	7.89	-0.40	0.69113
MUC1	0.05	5.51	0.40	0.69114
SLC26A11	-0.05	8.13	-0.40	0.69130
CD28	0.05	8.66	0.40	0.69130
DUSP8	-0.05	6.21	-0.40	0.69131
MYEOV	0.06	5.58	0.40	0.69160
XRCC4	-0.03	6.52	-0.40	0.69176
STX16	-0.04	10.13	-0.40	0.69194
KIF5B	-0.03	9.22	-0.40	0.69203
PIK3C2B	-0.05	8.03	-0.40	0.69207
SDHC	-0.02	8.97	-0.40	0.69211
RAB29	0.03	9.44	0.40	0.69233
RAB2A	-0.03	9.20	-0.40	0.69234
TMEM242	-0.03	6.95	-0.40	0.69236
LRFN4	-0.06	6.22	-0.40	0.69245

THUMPD3-AS1	0.06	7.61	0.40	0.69254
ZNF852	0.04	5.51	0.40	0.69263
BBX	0.03	8.78	0.40	0.69271
RCN2	-0.04	7.11	-0.40	0.69288
COX15	0.03	8.39	0.40	0.69297
IGIP	-0.04	6.57	-0.40	0.69301
ATXN3	0.03	8.01	0.40	0.69310
GATAD2A	0.02	10.91	0.40	0.69316
FTH1P20	-0.05	7.48	-0.40	0.69354
WDR62	0.04	5.88	0.40	0.69368
UBE2O	0.05	9.88	0.40	0.69402
GNL3	-0.03	8.32	-0.40	0.69406
CLBA1	0.04	5.17	0.40	0.69409
FBXO9	0.05	9.61	0.40	0.69415
CEP95	-0.04	8.05	-0.40	0.69424
RPL31P63	0.05	6.70	0.40	0.69425
BAZ1A	-0.06	10.32	-0.40	0.69444
TSC22D3	-0.05	13.51	-0.39	0.69453
MIR1244-3	0.05	5.78	0.39	0.69463
GRAMD1A	-0.05	10.67	-0.39	0.69463
FTH1P12	0.03	5.81	0.39	0.69468
MYL12AP1	-0.05	6.05	-0.39	0.69481
ABL1	-0.03	9.06	-0.39	0.69483
HSPD1	-0.03	9.91	-0.39	0.69486
EDAR	-0.06	5.71	-0.39	0.69496
AC020612.1	0.04	5.85	0.39	0.69496
FZD3	0.04	5.55	0.39	0.69510
SCNN1D	0.05	6.11	0.39	0.69511
ALOX5AP	-0.08	11.92	-0.39	0.69513
CYP27A1	0.07	9.97	0.39	0.69513
RFTN1	0.03	9.95	0.39	0.69559
SERPINB9P1	-0.05	7.77	-0.39	0.69563
ADAL	0.04	5.87	0.39	0.69576
NKD1	0.05	5.41	0.39	0.69584
NUP50-DT	0.05	7.73	0.39	0.69590
IGHV3-48	0.09	6.97	0.39	0.69592
ZNF860	-0.05	5.04	-0.39	0.69604
BRI3	0.05	11.57	0.39	0.69618
NUDT5	0.04	9.59	0.39	0.69625
TBC1D31	-0.03	7.06	-0.39	0.69632
EEF1A1P6	0.06	11.36	0.39	0.69633
ZNF816	0.04	6.11	0.39	0.69633
BRD1	-0.03	9.42	-0.39	0.69633
FAM216A	-0.04	6.11	-0.39	0.69640
ANAPC10	0.03	5.19	0.39	0.69643
UBFD1	0.02	8.29	0.39	0.69644
KRR1	0.04	7.61	0.39	0.69663
CD8B	0.08	9.77	0.39	0.69677
MIDN	0.05	11.16	0.39	0.69681
CUL1	-0.03	9.82	-0.39	0.69686

COQ6	-0.03	7.63	-0.39	0.69692
MRS2	-0.03	7.43	-0.39	0.69715
LRRC25	-0.05	12.00	-0.39	0.69729
UBE2V1	-0.05	5.15	-0.39	0.69739
PIGO	-0.03	8.28	-0.39	0.69750
SCNN1A	-0.05	5.32	-0.39	0.69755
POGLUT3	-0.04	6.33	-0.39	0.69758
CLIP3	-0.05	5.39	-0.39	0.69761
ANTXRLP1	0.04	5.18	0.39	0.69767
RPAP2	-0.03	7.27	-0.39	0.69796
ELP3	0.03	9.08	0.39	0.69801
RN7SL1	0.08	5.53	0.39	0.69817
RAC1P2	0.05	7.66	0.39	0.69819
TVP23C	0.04	5.55	0.39	0.69845
LTBP2	-0.05	6.53	-0.39	0.69851
TXK	-0.05	7.56	-0.39	0.69855
GATD3B	-0.04	5.35	-0.39	0.69858
GDAP1	-0.04	5.28	-0.39	0.69871
ABO	-0.09	5.78	-0.39	0.69879
ABHD12	0.04	8.94	0.39	0.69892
FHL3	0.06	9.78	0.39	0.69922
DGKD	-0.04	10.98	-0.39	0.69928
LPAR2	-0.06	11.41	-0.39	0.69932
DUSP14	0.05	6.59	0.39	0.69933
FKBPL	0.04	6.62	0.39	0.69946
RPL13P5	0.04	5.69	0.39	0.69988
TMSB4X	0.04	14.88	0.39	0.70012
TTC9	-0.04	9.09	-0.39	0.70019
CKS1B	0.04	6.04	0.39	0.70020
GSDMB	0.06	8.32	0.39	0.70033
SQOR	-0.04	10.03	-0.39	0.70040
PLEKHA1	-0.04	7.34	-0.39	0.70040
USP27X	-0.04	6.27	-0.39	0.70041
MIA2	-0.02	8.65	-0.39	0.70051
MED22	0.03	9.47	0.39	0.70080
CRTAP	-0.03	10.50	-0.39	0.70080
AC026271.1	-0.04	5.47	-0.39	0.70081
CHTF18	0.04	7.38	0.39	0.70114
PIGP	0.04	6.34	0.39	0.70114
C11orf80	-0.05	6.43	-0.39	0.70143
FBXL14	-0.03	7.99	-0.39	0.70149
CA3-AS1	0.06	5.19	0.39	0.70175
DCAF8	0.03	9.11	0.38	0.70197
AC006001.3	0.03	7.94	0.38	0.70198
IL4R	-0.06	11.91	-0.38	0.70200
NCL	0.03	11.73	0.38	0.70231
AC073172.1	-0.08	5.72	-0.38	0.70237
ALDH6A1	0.03	6.66	0.38	0.70290
ESPN	0.11	8.20	0.38	0.70312
ZNF778	-0.03	7.08	-0.38	0.70319

ACRBP	0.05	9.05	0.38	0.70320
TBC1D1	-0.03	10.48	-0.38	0.70324
FAM20B	-0.02	8.30	-0.38	0.70348
KDM1A	0.02	9.11	0.38	0.70350
TMEM121B	0.07	7.87	0.38	0.70365
ORC1	-0.05	5.32	-0.38	0.70378
LINC02158	-0.06	6.69	-0.38	0.70379
AC060780.1	-0.04	7.12	-0.38	0.70383
ARMC12	-0.06	5.13	-0.38	0.70395
ABLIM2	-0.04	5.46	-0.38	0.70399
ZNF589	-0.03	8.16	-0.38	0.70402
SETP14	-0.06	6.28	-0.38	0.70406
NUP210	0.03	11.83	0.38	0.70418
SBNO1	0.02	9.28	0.38	0.70426
MOSMO	-0.03	6.49	-0.38	0.70435
YARS2	0.03	7.18	0.38	0.70437
PTGES	-0.06	5.41	-0.38	0.70489
NPIPA2	-0.03	5.87	-0.38	0.70490
RNF10	0.07	12.74	0.38	0.70506
WDR82	0.02	11.55	0.38	0.70507
GNRH1	-0.04	5.50	-0.38	0.70524
ZNF250	0.04	6.84	0.38	0.70527
ODF2	-0.02	9.13	-0.38	0.70551
INPP5B	-0.03	8.69	-0.38	0.70554
SUCLG1	0.02	9.19	0.38	0.70558
MYL4	0.11	9.45	0.38	0.70560
FYN	0.02	11.16	0.38	0.70561
AC020917.4	-0.06	7.07	-0.38	0.70567
BST2	0.06	11.10	0.38	0.70572
CDC7	-0.04	5.37	-0.38	0.70588
PKMYT1	0.04	5.21	0.38	0.70612
SECTM1	-0.06	12.02	-0.38	0.70623
AC021097.1	0.04	5.72	0.38	0.70629
SLC29A3	0.04	7.95	0.38	0.70631
MLEC	0.03	10.05	0.38	0.70636
ICA1	0.03	6.34	0.38	0.70637
E2F6	-0.03	6.21	-0.38	0.70640
CLEC9A	-0.04	5.35	-0.38	0.70647
TMSB10P2	0.04	5.37	0.38	0.70654
AC007780.1	-0.05	5.66	-0.38	0.70656
MSRB2	0.06	8.35	0.38	0.70660
LRRK2-DT	-0.05	5.94	-0.38	0.70661
AC009093.8	-0.06	5.98	-0.38	0.70671
COBLL1	-0.05	5.86	-0.38	0.70681
FGFBP3	0.03	5.29	0.38	0.70688
IGHV1-58	0.06	5.14	0.38	0.70711
ITPRIPL1	0.04	7.49	0.38	0.70717
MTHFD1L	-0.05	6.41	-0.38	0.70735
MRNIP	0.05	8.48	0.38	0.70744
ERCC3	0.02	9.13	0.38	0.70754

CXorf21	-0.04	8.40	-0.38	0.70754
CTPS1	-0.04	7.33	-0.38	0.70771
ME3	-0.05	5.61	-0.38	0.70771
HTRA1	0.06	5.26	0.38	0.70773
GOT1	-0.03	7.67	-0.38	0.70776
GAPDHP72	0.04	5.25	0.38	0.70781
HTATSF1	0.03	9.20	0.38	0.70795
TTC7A	0.03	10.74	0.38	0.70802
PPP1R13B	0.03	7.70	0.38	0.70806
JAML	-0.04	13.51	-0.38	0.70815
CETN3	0.03	6.08	0.38	0.70819
MUC20	0.07	5.76	0.38	0.70822
MYO18A	-0.03	10.42	-0.38	0.70825
MICU1	0.03	9.73	0.38	0.70838
AL031282.2	-0.05	5.34	-0.38	0.70841
AC093157.1	-0.04	6.20	-0.38	0.70842
RPL17P50	0.04	4.92	0.38	0.70845
CRYGS	0.05	6.02	0.38	0.70849
HDHD5	0.03	9.36	0.38	0.70860
CTSK	-0.04	6.60	-0.38	0.70877
RBBP7	-0.02	9.49	-0.38	0.70897
PCBD2	0.04	6.52	0.38	0.70904
LDHBP2	0.06	7.24	0.38	0.70907
EPB41	-0.03	11.45	-0.38	0.70907
C2orf68	-0.02	9.57	-0.38	0.70907
MIR3936HG	0.04	4.94	0.37	0.70927
INE1	-0.05	5.18	-0.37	0.70939
TRIM68	-0.03	7.36	-0.37	0.70945
IL17RC	-0.05	5.77	-0.37	0.70951
ADAMTS1	-0.06	5.68	-0.37	0.70962
WARS2	-0.03	6.42	-0.37	0.70966
SF3A3P1	-0.04	5.08	-0.37	0.70973
KCTD5	0.03	9.23	0.37	0.71001
PTGFRN	-0.06	5.64	-0.37	0.71026
MEAK7	0.03	7.01	0.37	0.71032
PIGZ	0.04	5.38	0.37	0.71052
EIF3IP1	0.04	5.18	0.37	0.71060
APOO	-0.03	6.18	-0.37	0.71061
TIGD3	0.06	8.38	0.37	0.71073
MUTYH	0.04	5.27	0.37	0.71074
TMBIM1	-0.03	11.49	-0.37	0.71088
ABHD17AP6	-0.03	5.29	-0.37	0.71098
GMPS	0.02	8.42	0.37	0.71099
ZNF140	0.03	6.81	0.37	0.71104
LEPROTL1	0.03	9.58	0.37	0.71112
MBNL1-AS1	-0.05	5.49	-0.37	0.71112
MED25	0.05	11.20	0.37	0.71114
INTS13	0.04	7.06	0.37	0.71132
KCNQ1OT1	-0.05	4.94	-0.37	0.71176
NUFIP1	0.03	5.80	0.37	0.71178

ATP5MDP1	0.04	5.46	0.37	0.71194
BCORP1	-0.09	5.38	-0.37	0.71195
SLC7A5	0.06	8.61	0.37	0.71201
SIAH2	0.04	9.66	0.37	0.71206
HNRNPKP1	-0.04	5.51	-0.37	0.71216
HAUS4	-0.04	8.05	-0.37	0.71225
CD226	0.04	8.12	0.37	0.71234
CLIC1	0.04	12.83	0.37	0.71292
GTF2IP20	0.05	6.84	0.37	0.71300
DOT1L	0.03	9.00	0.37	0.71303
F8A1	0.05	8.64	0.37	0.71338
RRP7BP	-0.05	6.89	-0.37	0.71339
ATG10	0.04	6.09	0.37	0.71348
GINM1	-0.03	7.85	-0.37	0.71359
CNIH2	0.05	5.21	0.37	0.71362
HSP90B2P	-0.04	7.05	-0.37	0.71380
EIF4BP3	-0.04	6.71	-0.37	0.71392
ZFYVE27	0.03	9.64	0.37	0.71417
MRPL16	0.03	8.64	0.37	0.71421
FTLP3	0.06	10.81	0.37	0.71435
STIMATE	0.04	5.05	0.37	0.71478
TMEM70	-0.03	7.36	-0.37	0.71485
NEK3	-0.03	6.04	-0.37	0.71488
RPL22P2	0.05	6.50	0.37	0.71489
RTCA-AS1	-0.05	5.75	-0.37	0.71494
AL133342.1	0.04	5.51	0.37	0.71512
SH3BGRL	-0.03	9.78	-0.37	0.71525
CRACR2A	0.02	7.94	0.37	0.71527
TMEM9B	-0.03	9.05	-0.37	0.71529
SEC22A	0.03	7.09	0.37	0.71534
AKAP7	0.03	6.53	0.37	0.71543
IGKV1-27	0.06	6.36	0.37	0.71548
ALG11	-0.03	5.41	-0.37	0.71551
CTSZ	-0.04	12.74	-0.37	0.71556
ZNF280B	0.04	5.15	0.37	0.71557
USP14	0.03	8.23	0.37	0.71559
NPIPB13	-0.06	8.59	-0.37	0.71569
ARF6	0.02	10.97	0.37	0.71580
AL355922.1	-0.04	5.54	-0.37	0.71593
DGAT2	-0.07	11.15	-0.37	0.71595
MUC20P1	-0.05	5.14	-0.37	0.71598
ADA	0.04	8.91	0.37	0.71600
ABHD10	-0.03	7.55	-0.37	0.71653
NRN1	-0.07	5.00	-0.37	0.71654
GRN	0.05	13.96	0.36	0.71667
GRIN2D	-0.05	5.27	-0.36	0.71670
HNRNPA1P30	0.04	5.02	0.36	0.71670
CLOCK	0.04	6.71	0.36	0.71677
LINC00205	-0.04	7.01	-0.36	0.71690
LAMB3	-0.04	5.27	-0.36	0.71709

NT5E	-0.06	6.28	-0.36	0.71713
EPHA2	0.05	5.20	0.36	0.71714
OR4D1	-0.04	5.67	-0.36	0.71718
ENY2	0.03	8.90	0.36	0.71748
NDUFB2-AS1	-0.04	5.27	-0.36	0.71751
SRGAP2C	-0.04	6.30	-0.36	0.71784
CIB2	0.04	5.89	0.36	0.71799
FITM1	0.04	5.52	0.36	0.71803
CBWD6	0.05	6.32	0.36	0.71823
AC093599.2	0.06	5.02	0.36	0.71829
FUNDC1	-0.04	6.33	-0.36	0.71845
LAMB2	-0.04	6.83	-0.36	0.71853
AKT1	0.03	11.82	0.36	0.71855
AC093827.4	-0.05	5.03	-0.36	0.71862
ZNF302	-0.05	7.22	-0.36	0.71867
GINS4	0.03	5.79	0.36	0.71875
ASCC1	0.03	7.32	0.36	0.71876
MPP5	-0.04	5.62	-0.36	0.71886
URI1	-0.03	8.86	-0.36	0.71913
MT1X	-0.04	5.56	-0.36	0.71923
GTF2IRD2	0.04	6.17	0.36	0.71925
AL358942.1	-0.04	5.03	-0.36	0.71927
SSR3	-0.03	9.30	-0.36	0.71931
CAMKK1	0.05	8.61	0.36	0.71936
ZNF84	-0.03	6.86	-0.36	0.71945
AP000866.1	0.04	5.23	0.36	0.71952
SLC16A13	0.04	6.62	0.36	0.71957
AL158801.6	0.04	5.63	0.36	0.71959
NQO1	-0.04	5.53	-0.36	0.71961
CROCCP3	-0.04	5.80	-0.36	0.71966
RPA1	0.02	10.23	0.36	0.71983
SFR1	-0.04	5.86	-0.36	0.71987
RTKN	0.05	5.55	0.36	0.71996
CHEK2	0.04	6.41	0.36	0.72007
CUL4A	-0.02	9.73	-0.36	0.72010
AC040160.1	-0.05	7.18	-0.36	0.72018
TAF1B	-0.03	5.90	-0.36	0.72024
KLHDC1	-0.04	5.06	-0.36	0.72047
ACTG1P23	-0.03	5.07	-0.36	0.72066
AIMP2	0.03	7.81	0.36	0.72075
FAM157A	-0.07	9.13	-0.36	0.72078
DNM2	-0.03	12.10	-0.36	0.72086
AC092384.1	0.03	5.32	0.36	0.72096
ARL14EP	-0.03	7.53	-0.36	0.72110
ARHGEF37	-0.07	5.02	-0.36	0.72126
CSNK2A1	-0.02	9.21	-0.36	0.72134
ROCK1	-0.04	9.40	-0.36	0.72148
ALKBH1	0.02	7.28	0.36	0.72155
ACYP1	0.04	5.85	0.36	0.72160
AC004057.1	0.11	7.54	0.36	0.72166

ZNF57	0.03	5.72	0.36	0.72180
HVCN1	0.03	10.43	0.36	0.72190
WDR55	-0.02	9.08	-0.36	0.72192
APOBR	0.05	11.88	0.36	0.72197
GPAT2	-0.09	5.88	-0.36	0.72209
GPR157	0.03	6.84	0.36	0.72250
IGKV1D-12	0.06	5.47	0.36	0.72281
ZNF440	0.03	7.00	0.36	0.72287
IGLV4-60	-0.07	5.33	-0.36	0.72303
WARS2-AS1	-0.03	6.30	-0.36	0.72308
TRAPPC2B	0.04	5.86	0.36	0.72335
NAXD	0.03	8.46	0.36	0.72354
TMEM143	0.03	6.80	0.36	0.72359
DAAM1	0.03	6.66	0.36	0.72360
ARHGAP1	0.02	11.26	0.36	0.72361
PDCL3P4	0.04	5.22	0.36	0.72362
ARHGAP10	0.04	7.15	0.36	0.72396
B9D2	0.04	8.23	0.36	0.72396
USP30	-0.03	6.87	-0.36	0.72401
YTHDF1	0.02	9.65	0.35	0.72414
AC011921.3	0.04	5.21	0.35	0.72421
MT2P1	-0.07	5.89	-0.35	0.72435
HNRNPDP1	-0.04	5.85	-0.35	0.72470
ZNF174	-0.02	7.55	-0.35	0.72473
BOD1L1	-0.04	10.80	-0.35	0.72509
TRIP11	0.02	7.76	0.35	0.72521
CDC16	0.02	9.11	0.35	0.72523
TINF2	0.02	10.39	0.35	0.72525
ERCC5	-0.05	5.12	-0.35	0.72531
IL12RB2	0.05	6.31	0.35	0.72558
ZNF37A	-0.03	7.37	-0.35	0.72568
COPZ2	0.04	5.77	0.35	0.72576
AL357143.1	-0.05	5.14	-0.35	0.72595
TRMT11	-0.03	5.87	-0.35	0.72608
SIAH1	-0.03	7.01	-0.35	0.72609
PARP2	0.03	6.90	0.35	0.72614
DENND10	0.03	8.65	0.35	0.72626
GAPDHP71	-0.03	5.19	-0.35	0.72637
CD200R1	-0.04	6.18	-0.35	0.72644
AZU1	0.10	6.66	0.35	0.72648
SLC35E2A	-0.07	8.83	-0.35	0.72648
CISD1	-0.04	6.52	-0.35	0.72675
ADIRF-AS1	0.04	5.62	0.35	0.72692
SDHAP1	-0.03	7.03	-0.35	0.72694
AL035661.1	-0.08	6.09	-0.35	0.72697
H2AW	-0.04	6.55	-0.35	0.72705
AL357033.3	0.04	5.55	0.35	0.72708
AC064805.2	0.04	5.18	0.35	0.72724
HLA-DPB1	0.05	13.20	0.35	0.72732
UBE2G1	-0.02	9.12	-0.35	0.72737

NDUFV2P1	-0.04	5.95	-0.35	0.72747
HES6	-0.04	5.69	-0.35	0.72756
TUBGCP4	0.04	7.62	0.35	0.72758
WDR86	0.04	5.26	0.35	0.72795
ADAM12	0.05	5.33	0.35	0.72797
ICOSLG	-0.05	5.85	-0.35	0.72814
AL390119.2	-0.04	5.02	-0.35	0.72829
SPON2	0.06	10.28	0.35	0.72835
VTI1A	-0.02	8.02	-0.35	0.72854
AL391058.1	0.04	5.11	0.35	0.72879
POTEI	0.03	5.67	0.35	0.72921
AL669831.3	0.04	5.11	0.35	0.72924
LINC00853	0.05	5.98	0.35	0.72930
COX5AP2	0.05	5.33	0.35	0.72942
NGFR	-0.04	5.14	-0.35	0.72955
YPEL4	0.07	5.96	0.35	0.72972
PEX3	-0.03	5.75	-0.35	0.72974
CAMTA2	-0.03	10.68	-0.35	0.72978
OXER1	-0.05	8.12	-0.35	0.72982
AC103591.1	-0.04	5.07	-0.35	0.72996
CEP19	-0.05	8.27	-0.35	0.73021
TSPEAR	-0.06	5.25	-0.35	0.73022
SPIN3	-0.03	6.26	-0.35	0.73025
AC004980.1	0.03	5.45	0.35	0.73058
KBTBD6	0.04	6.44	0.35	0.73072
TMEM69	-0.03	8.23	-0.35	0.73073
PARP1P1	-0.05	5.60	-0.35	0.73079
IGLV3-12	-0.10	5.54	-0.35	0.73083
POM121	-0.02	9.53	-0.35	0.73086
BMP8B	-0.04	5.71	-0.35	0.73094
LINC01504	-0.03	5.86	-0.35	0.73096
AL590762.1	-0.04	5.41	-0.35	0.73109
ORM1	0.11	7.52	0.35	0.73118
SPNS1	0.04	5.06	0.35	0.73140
EPOR	0.03	8.25	0.35	0.73147
TOB1-AS1	0.04	5.55	0.34	0.73160
MPC1	-0.02	8.02	-0.34	0.73161
GATB	0.03	7.66	0.34	0.73166
AC004846.1	-0.04	5.20	-0.34	0.73198
ZDHHC2	0.04	8.47	0.34	0.73198
FAM156A	-0.04	5.51	-0.34	0.73199
MFSD13A	-0.04	7.33	-0.34	0.73210
NMRK1	-0.03	7.86	-0.34	0.73216
CTDP1	0.03	9.41	0.34	0.73218
AC106886.3	-0.05	4.97	-0.34	0.73222
DENR	-0.02	8.60	-0.34	0.73237
C1orf174	-0.02	8.45	-0.34	0.73285
TAGLN2	0.04	14.40	0.34	0.73299
DNM3	-0.04	5.90	-0.34	0.73308
OTUD7B	0.03	6.77	0.34	0.73334

PECR	0.03	7.26	0.34	0.73334
GALNT8	0.05	5.27	0.34	0.73342
C2orf42	0.02	7.69	0.34	0.73342
DOK7	0.05	5.13	0.34	0.73351
POC1B-AS1	-0.03	5.46	-0.34	0.73378
NCBP2	-0.03	8.27	-0.34	0.73378
ARL1	0.02	7.74	0.34	0.73379
LINC02604	0.04	6.66	0.34	0.73380
FAM192BP	0.04	5.67	0.34	0.73380
UNC119	-0.03	10.84	-0.34	0.73389
PIM2	0.03	11.65	0.34	0.73394
BEND7	-0.06	5.60	-0.34	0.73397
TMEM198B	0.04	8.27	0.34	0.73407
ASH2L	-0.02	9.27	-0.34	0.73409
YOD1	-0.07	7.01	-0.34	0.73421
GET4	0.04	5.93	0.34	0.73438
TBC1D3G	0.03	6.40	0.34	0.73479
RPS2P32	0.03	5.08	0.34	0.73479
ZNF485	-0.03	5.78	-0.34	0.73479
ZNF18	-0.03	8.15	-0.34	0.73490
ARHGAP17	0.02	10.33	0.34	0.73520
EPHX1	0.04	7.47	0.34	0.73536
PHF24	-0.06	5.20	-0.34	0.73540
ARL5A	-0.03	6.96	-0.34	0.73549
IFITM1	-0.05	11.19	-0.34	0.73558
NQO2	0.06	10.00	0.34	0.73569
PSMC1P1	-0.04	6.63	-0.34	0.73572
RAD18	-0.02	6.76	-0.34	0.73574
BCAM	0.09	6.01	0.34	0.73577
NPIPB11	-0.05	7.90	-0.34	0.73585
IGLV2-11	-0.07	7.68	-0.34	0.73595
HDAC1P2	0.03	5.13	0.34	0.73601
TRAV10	0.05	5.41	0.34	0.73615
WNT5B	0.05	5.57	0.34	0.73619
AC135507.1	0.04	5.49	0.34	0.73644
C1orf127	0.04	5.01	0.34	0.73661
DSTN	-0.03	8.91	-0.34	0.73668
GIN1	-0.03	6.31	-0.34	0.73672
ZFP14	-0.03	6.76	-0.34	0.73675
LZTR1	-0.04	7.76	-0.34	0.73677
THBS1	0.07	9.00	0.34	0.73683
SRP72P2	0.03	5.08	0.34	0.73683
OLIG2	0.06	5.53	0.34	0.73691
PIEZO1	-0.03	11.10	-0.34	0.73696
NAGK	-0.04	11.93	-0.34	0.73717
SOCS2-AS1	-0.03	5.26	-0.34	0.73721
NINJ2-AS1	-0.03	7.69	-0.34	0.73738
RDH16	-0.04	5.36	-0.34	0.73745
TRMT10C	-0.03	7.22	-0.34	0.73746
MZF1	0.04	8.53	0.34	0.73758

VDAC3P1	-0.04	5.12	-0.34	0.73760
NFE2	0.06	12.23	0.34	0.73764
PA2G4P4	0.04	6.04	0.34	0.73798
ANXA7	-0.02	10.08	-0.34	0.73802
ASIC3	-0.05	5.97	-0.34	0.73838
ABCC3	-0.06	8.96	-0.34	0.73846
ANKRD24	-0.04	5.12	-0.34	0.73855
IRF7	-0.06	10.19	-0.34	0.73868
AC144831.1	-0.04	5.83	-0.34	0.73871
GPRASP1	0.05	7.04	0.34	0.73879
PNRC1	-0.03	11.46	-0.34	0.73884
ATXN2L	-0.03	10.92	-0.34	0.73884
KLHDC4	-0.03	9.41	-0.34	0.73889
SRCAP	-0.03	6.00	-0.34	0.73892
ATXN10	0.03	9.09	0.33	0.73910
PABPC3	-0.05	8.92	-0.33	0.73920
ASMTL-AS1	-0.05	6.47	-0.33	0.73940
WDR75	-0.03	7.76	-0.33	0.73948
AC007686.4	0.06	5.54	0.33	0.73963
UNC13D	0.03	12.41	0.33	0.73990
GCFC2	-0.03	6.37	-0.33	0.73996
ITGA6-AS1	0.05	5.67	0.33	0.74001
AC010683.1	-0.03	5.27	-0.33	0.74004
KRT72	0.10	7.25	0.33	0.74013
ZNF473	-0.02	6.84	-0.33	0.74030
ARID1A	0.03	11.61	0.33	0.74035
LAIR1	0.03	10.47	0.33	0.74050
VIRMA	-0.02	8.76	-0.33	0.74054
HSPA4	-0.02	9.52	-0.33	0.74101
RIMKLB	-0.04	5.96	-0.33	0.74107
IDO1	-0.08	6.22	-0.33	0.74110
AC022210.2	-0.04	4.93	-0.33	0.74142
EIF1B-AS1	0.03	5.69	0.33	0.74143
FAM209A	-0.04	5.36	-0.33	0.74153
LRRC1	0.03	5.36	0.33	0.74156
AC003102.1	0.05	6.24	0.33	0.74162
TRIM41	0.02	9.23	0.33	0.74191
TRAV12-3	0.04	6.15	0.33	0.74192
GNAL	-0.05	5.37	-0.33	0.74206
AC023232.1	-0.04	5.06	-0.33	0.74226
ERVK3-1	-0.03	6.67	-0.33	0.74247
SENCR	0.04	6.87	0.33	0.74248
LINC01089	0.05	8.71	0.33	0.74261
SNRNP27	0.03	7.07	0.33	0.74297
ARPC3	-0.03	11.89	-0.33	0.74314
TCIRG1	0.04	12.64	0.33	0.74346
AC027801.1	-0.06	5.73	-0.33	0.74347
NFKBIE	0.04	8.82	0.33	0.74350
DHX38	0.02	10.38	0.33	0.74368
ZNF160	-0.02	8.37	-0.33	0.74370

MED8	0.02	8.98	0.33	0.74370
LILRA5	-0.06	11.51	-0.33	0.74379
TMEM175	0.03	9.47	0.33	0.74379
KAT6B	-0.03	8.29	-0.33	0.74385
YBX1P10	-0.04	7.13	-0.33	0.74392
RHOBTB1	-0.04	5.90	-0.33	0.74428
PSMB3P2	0.04	5.61	0.33	0.74450
RTP5	0.05	5.50	0.33	0.74454
LINC02772	-0.07	5.43	-0.33	0.74461
ZFYVE9	0.03	5.60	0.33	0.74468
GPR183	0.04	8.72	0.33	0.74478
DHX8	-0.02	10.16	-0.33	0.74484
RNF5P1	-0.04	5.58	-0.33	0.74514
RBM43	-0.04	6.47	-0.33	0.74524
AC078883.1	-0.04	5.88	-0.33	0.74524
CEACAM3	-0.05	10.73	-0.33	0.74525
PTX3	-0.04	5.24	-0.33	0.74533
RAD1	-0.02	7.17	-0.33	0.74559
C16orf54	-0.03	11.28	-0.33	0.74560
PSME2	0.04	10.64	0.33	0.74574
SERPINE2	-0.04	5.31	-0.33	0.74577
FLVCR1-DT	0.05	6.49	0.33	0.74585
AC005261.2	-0.03	7.97	-0.33	0.74587
TBC1D19	0.03	5.20	0.33	0.74595
UBE2E3	-0.03	8.39	-0.33	0.74597
DAP	0.03	10.60	0.33	0.74612
CCDC50	-0.03	8.03	-0.33	0.74612
AK3	0.03	7.40	0.33	0.74624
SYNGR1	0.04	7.53	0.32	0.74668
TXNDC17	0.03	8.26	0.32	0.74668
BX539320.1	-0.04	5.00	-0.32	0.74678
OXCT1	0.03	7.45	0.32	0.74688
SLC29A1	0.05	8.35	0.32	0.74695
PLIN5	-0.07	7.81	-0.32	0.74716
IGKV1D-8	-0.04	5.17	-0.32	0.74746
EIF3J-DT	0.03	7.16	0.32	0.74752
AC093752.1	-0.04	6.47	-0.32	0.74757
LSM12	-0.03	6.31	-0.32	0.74796
TSGA10	0.03	5.45	0.32	0.74814
SAPCD2	-0.05	5.79	-0.32	0.74838
RAN	-0.03	10.84	-0.32	0.74847
FOXO4	-0.04	10.03	-0.32	0.74865
TNS2	0.04	5.10	0.32	0.74866
AC018371.2	0.04	5.56	0.32	0.74868
MIF4GD	0.03	9.27	0.32	0.74871
EEF1A2	0.04	5.12	0.32	0.74875
EXOSC9	0.02	7.96	0.32	0.74886
ALG6	-0.02	6.83	-0.32	0.74889
PDCD5	-0.03	7.96	-0.32	0.74931
MTERF4	0.03	8.93	0.32	0.74933

IFT80	0.03	5.54	0.32	0.74935
CPAMD8	0.05	7.17	0.32	0.74938
BLCAP	0.02	9.82	0.32	0.74948
ZHX1	-0.03	6.99	-0.32	0.74965
RGMB	0.05	6.31	0.32	0.74967
IGHG1	-0.12	11.19	-0.32	0.74978
NKAPP1	-0.03	5.05	-0.32	0.74979
AL109615.4	-0.04	5.28	-0.32	0.74980
SRSF7	-0.02	10.06	-0.32	0.74994
MYBPC3	-0.05	7.95	-0.32	0.74995
GUSBP1	-0.03	6.09	-0.32	0.74996
ZNF747	0.03	6.92	0.32	0.75014
EPS15L1	0.02	9.97	0.32	0.75015
ARPC2	-0.03	13.24	-0.32	0.75031
MATN1-AS1	-0.04	6.31	-0.32	0.75041
GPR162	-0.05	8.59	-0.32	0.75044
AC092652.2	0.04	5.00	0.32	0.75049
AC023424.2	-0.03	5.26	-0.32	0.75055
BPI	-0.11	7.99	-0.32	0.75057
CIP2A	0.04	5.10	0.32	0.75075
LMCD1	0.04	5.05	0.32	0.75076
PTCD2	-0.03	6.20	-0.32	0.75103
AC009126.1	0.03	5.55	0.32	0.75114
TMEM45B	-0.05	6.60	-0.32	0.75117
GTDC1	-0.04	7.58	-0.32	0.75143
NET1	-0.04	6.39	-0.32	0.75166
PEX12	-0.03	6.25	-0.32	0.75168
KBTBD8	-0.04	5.78	-0.32	0.75177
SBF1P1	0.04	5.59	0.32	0.75186
CRABP2	0.04	5.46	0.32	0.75186
SLC22A23	-0.05	7.10	-0.32	0.75220
SYAP1	0.02	8.65	0.32	0.75221
WRNIP1	0.02	9.15	0.32	0.75221
TALDO1	0.04	13.60	0.32	0.75278
LDLRAD4	0.02	7.87	0.32	0.75287
GAR1	0.03	6.87	0.32	0.75289
H2BC11	-0.05	6.85	-0.32	0.75316
PCYOX1	-0.03	7.72	-0.32	0.75323
RPL17	-0.03	5.42	-0.32	0.75325
PPIP5K1	0.03	6.88	0.32	0.75332
AC018638.5	0.04	7.57	0.32	0.75345
PTPN23	0.02	9.92	0.32	0.75351
SETD9	-0.04	5.08	-0.32	0.75363
SLC4A5	-0.04	5.15	-0.32	0.75365
ZNF546	-0.03	5.51	-0.32	0.75367
MALT1	0.02	8.65	0.31	0.75421
ZNF542P	-0.03	6.64	-0.31	0.75433
SNHG30	0.03	6.06	0.31	0.75443
H2AZP3	0.03	5.20	0.31	0.75454
DCAF7	0.02	10.43	0.31	0.75476

KDM2B	0.02	9.14	0.31	0.75480
GAPDHP65	0.05	6.94	0.31	0.75482
ZNF823	0.03	5.51	0.31	0.75485
TXNDC15	-0.02	8.33	-0.31	0.75495
EIF1B	0.04	9.57	0.31	0.75496
CABLES1	0.04	4.98	0.31	0.75510
RETSAT	0.03	8.66	0.31	0.75518
CCDC51	0.02	6.95	0.31	0.75539
FKBP4	0.03	9.42	0.31	0.75542
KIT	-0.04	5.16	-0.31	0.75543
ABCC10	0.02	8.93	0.31	0.75551
LINC00671	0.05	5.71	0.31	0.75563
ZNF277	0.03	7.52	0.31	0.75567
AC119396.1	0.05	8.11	0.31	0.75590
GMCL1	-0.03	7.99	-0.31	0.75592
ADAMTS17	-0.04	5.27	-0.31	0.75595
RPL27AP	0.05	6.34	0.31	0.75596
DENND1A	-0.02	10.03	-0.31	0.75597
IGLV5-45	-0.06	5.46	-0.31	0.75631
LRRC27	0.04	6.77	0.31	0.75631
SHB	-0.04	4.96	-0.31	0.75631
TRAV26-1	-0.04	5.95	-0.31	0.75641
GID4	-0.03	7.79	-0.31	0.75646
SEH1L	-0.03	7.54	-0.31	0.75647
SMPD3	-0.05	7.49	-0.31	0.75655
PSPH	-0.04	5.74	-0.31	0.75693
CERS5	0.02	9.28	0.31	0.75708
ARMC10	0.02	7.40	0.31	0.75712
RAB3GAP1	-0.02	8.97	-0.31	0.75714
SLC25A25-AS1	-0.05	7.49	-0.31	0.75723
CDR2	-0.03	8.58	-0.31	0.75755
RSL24D1	0.03	8.19	0.31	0.75769
TBC1D32	-0.03	5.27	-0.31	0.75769
SMG5	-0.03	10.76	-0.31	0.75780
ST6GAL1	0.03	11.40	0.31	0.75782
IGKV2D-29	-0.07	5.61	-0.31	0.75784
AL162458.1	0.03	5.80	0.31	0.75791
TK2	0.02	9.16	0.31	0.75794
RNASE6	-0.05	9.85	-0.31	0.75806
PRKAG1	0.02	9.06	0.31	0.75810
TFCP2L1	0.05	5.42	0.31	0.75826
LY6E	0.06	11.53	0.31	0.75836
ZNF554	0.02	6.49	0.31	0.75837
JAK3	0.04	11.79	0.31	0.75851
HSPA8	-0.02	13.59	-0.31	0.75855
TBL1XR1	-0.02	9.23	-0.31	0.75862
TAS1R3	-0.04	5.28	-0.31	0.75871
TMCC2	0.08	8.14	0.31	0.75879
AC012513.3	-0.03	5.07	-0.31	0.75918
FTH1P10	0.04	8.25	0.31	0.75929

SMAP1	-0.02	8.82	-0.31	0.75930
CARD9	0.04	8.71	0.31	0.75950
GPR171	0.03	7.32	0.31	0.75955
UPK3B	-0.04	5.47	-0.31	0.75961
FOXO3	0.05	9.73	0.31	0.75964
ZNF620	-0.03	5.32	-0.31	0.75991
CEACAM21	0.04	8.52	0.31	0.75992
HNRNPA1P4	-0.03	5.37	-0.31	0.76023
KIAA1191	-0.02	8.85	-0.31	0.76035
AC007032.1	0.04	5.51	0.31	0.76035
HDAC2	-0.02	8.42	-0.31	0.76049
RAB1C	0.04	6.39	0.31	0.76050
ZNF281	-0.04	7.92	-0.31	0.76060
AL391416.1	-0.03	4.91	-0.31	0.76068
FBXL6	0.03	8.12	0.31	0.76074
CXCL1	-0.04	6.43	-0.31	0.76077
FCRL3	-0.05	9.11	-0.31	0.76086
UMAD1	-0.03	6.49	-0.31	0.76088
AL121839.2	-0.03	6.18	-0.31	0.76102
BTBD7	-0.02	7.79	-0.31	0.76104
RAI1	-0.03	8.20	-0.31	0.76106
SSPN	0.04	5.43	0.31	0.76121
DLGAP1-AS2	0.04	5.79	0.31	0.76127
PODN	0.05	5.55	0.31	0.76136
AC009779.5	0.03	5.35	0.31	0.76139
OFD1P17	-0.03	5.25	-0.31	0.76144
PIM1	-0.04	11.69	-0.31	0.76156
HIVEP3	-0.03	8.30	-0.30	0.76159
KIF5C	-0.04	6.11	-0.30	0.76168
LST1	0.04	12.46	0.30	0.76178
UBBP4	0.06	7.49	0.30	0.76182
DDX21	0.03	8.93	0.30	0.76182
CNBD2	-0.03	5.37	-0.30	0.76209
CHRNA10	0.04	6.09	0.30	0.76215
MADD	0.02	10.68	0.30	0.76228
HIGD1A	-0.03	6.76	-0.30	0.76233
AC138207.8	0.03	5.27	0.30	0.76260
RPS26P6	0.08	6.96	0.30	0.76289
CRTC3	0.03	9.77	0.30	0.76300
AC097523.1	0.03	5.45	0.30	0.76310
C1orf116	-0.04	5.37	-0.30	0.76316
MLLT3	0.03	7.52	0.30	0.76346
HDAC6	-0.02	9.45	-0.30	0.76347
AIFM2	0.03	6.53	0.30	0.76349
PLIN4	-0.07	8.51	-0.30	0.76358
PXN	-0.03	13.05	-0.30	0.76369
LRRC47	0.02	9.23	0.30	0.76370
PHF20L1	-0.04	8.84	-0.30	0.76387
DEF8	-0.03	11.20	-0.30	0.76421
CIDEB	-0.03	9.75	-0.30	0.76451

CLEC4O	0.09	5.45	0.30	0.76469
OGDH	0.02	11.13	0.30	0.76472
WDR53	0.02	6.81	0.30	0.76484
ZNF251	-0.03	6.91	-0.30	0.76493
RPL10P6	0.06	8.34	0.30	0.76513
AC004241.1	0.04	6.08	0.30	0.76531
SNRPD2P1	-0.03	5.44	-0.30	0.76545
RAPSN	0.04	5.27	0.30	0.76559
NEK6	-0.03	8.23	-0.30	0.76565
KIF9	-0.03	5.51	-0.30	0.76570
EEF1A1P3	0.04	5.73	0.30	0.76574
ZFAND1	-0.03	6.74	-0.30	0.76576
TOR3A	0.03	8.76	0.30	0.76610
ZDHHC8	0.03	8.31	0.30	0.76612
COCH	-0.04	5.51	-0.30	0.76619
PFN1P9	0.03	5.12	0.30	0.76622
BX284668.5	-0.03	6.82	-0.30	0.76624
ARL10	0.03	6.39	0.30	0.76628
FAM174B	-0.03	5.76	-0.30	0.76659
PGK1P2	-0.03	6.63	-0.30	0.76680
ST3GAL4	-0.05	9.22	-0.30	0.76682
LRP12	-0.04	5.02	-0.30	0.76693
SHMT1	-0.03	7.42	-0.30	0.76698
ATP8B2	0.03	10.57	0.30	0.76713
PIBF1	-0.03	6.37	-0.30	0.76732
CARS1	0.02	10.10	0.30	0.76737
C12orf66	0.02	6.24	0.30	0.76747
PRKACA	0.02	11.70	0.30	0.76783
AC005520.4	-0.04	5.32	-0.30	0.76788
CDC42	-0.02	11.82	-0.30	0.76806
SNX33	0.03	7.53	0.30	0.76808
AL356481.1	0.03	4.90	0.30	0.76819
ING4	0.02	9.18	0.30	0.76847
TNS1	0.08	9.83	0.30	0.76851
DUS4L	-0.03	5.07	-0.30	0.76853
AC103858.3	-0.05	6.82	-0.30	0.76857
CYP4F35P	0.04	5.02	0.30	0.76864
AASS	0.04	5.18	0.30	0.76868
AC079228.1	0.04	5.78	0.30	0.76899
PUS10	-0.03	5.68	-0.30	0.76915
AL035071.1	0.03	6.99	0.29	0.76919
AC245884.12	-0.05	7.21	-0.29	0.76919
EEF1A1P4	0.04	6.70	0.29	0.76947
GRK2	0.02	13.05	0.29	0.76954
RRAGB	-0.02	6.40	-0.29	0.76956
GMIP	-0.03	12.18	-0.29	0.76957
AC102953.2	0.03	5.19	0.29	0.76968
RBMX	0.02	9.84	0.29	0.76973
LAMC1	-0.04	6.11	-0.29	0.76974
NAP1L1P1	-0.04	6.64	-0.29	0.76979

AC135279.3	-0.03	5.17	-0.29	0.76980
ZSCAN2	-0.02	7.04	-0.29	0.76984
CNIH4	-0.03	8.20	-0.29	0.76990
TCN2	-0.05	8.13	-0.29	0.77032
AC245100.4	-0.03	5.26	-0.29	0.77038
IRAK1BP1	-0.03	5.15	-0.29	0.77050
DDX28	0.02	8.53	0.29	0.77053
NKX3-1	0.07	5.84	0.29	0.77081
TRBJ1-5	0.03	5.71	0.29	0.77086
LNPK	0.02	7.63	0.29	0.77129
PTGS1	0.04	10.17	0.29	0.77130
ZNF544	0.03	7.47	0.29	0.77140
INSL3	-0.03	6.44	-0.29	0.77149
ARVCF	-0.04	6.56	-0.29	0.77155
MXRA7	0.07	7.58	0.29	0.77157
SYNGAP1	-0.03	8.73	-0.29	0.77162
VPS9D1	-0.05	9.35	-0.29	0.77176
SLC43A2	-0.03	12.12	-0.29	0.77187
TRAM1	-0.02	9.42	-0.29	0.77187
HNRNPA1	0.02	12.19	0.29	0.77188
ACOT1	0.03	5.22	0.29	0.77189
BASP1	-0.06	11.94	-0.29	0.77190
B4GALT2	0.04	6.05	0.29	0.77210
AKT3	-0.02	7.91	-0.29	0.77214
RAVER2	0.03	5.38	0.29	0.77224
ZBED1	0.02	10.07	0.29	0.77236
PLEKHA2	-0.02	9.77	-0.29	0.77239
SEC31B	-0.05	8.39	-0.29	0.77250
CACNA1C-AS1	0.03	5.30	0.29	0.77258
EHMT1	-0.02	10.12	-0.29	0.77270
KIF3C	0.03	7.81	0.29	0.77293
METAP1D	0.03	6.34	0.29	0.77305
CA5B	-0.03	8.11	-0.29	0.77315
FMNL3	0.03	9.35	0.29	0.77339
LINC02012	0.03	5.02	0.29	0.77340
MSC	0.05	6.70	0.29	0.77381
TRPV1	-0.03	6.42	-0.29	0.77385
MTFR1L	0.02	8.91	0.29	0.77416
KDM5A	-0.02	10.16	-0.29	0.77417
TBXA2R	0.03	6.05	0.29	0.77432
PGAM4	-0.03	5.57	-0.29	0.77435
IRS2	0.06	9.43	0.29	0.77450
AP002495.1	0.03	5.39	0.29	0.77456
SYNE3	0.02	9.84	0.29	0.77472
CEP89	0.02	7.68	0.29	0.77473
TMEM38B	-0.03	5.66	-0.29	0.77480
AC025171.4	-0.03	5.46	-0.29	0.77486
TULP3	-0.02	6.70	-0.29	0.77486
ACVR1	0.03	7.59	0.29	0.77495
CEP70	0.03	5.22	0.29	0.77500

TNNT3	-0.04	6.22	-0.29	0.77510
CTBP1-DT	0.02	7.57	0.29	0.77515
PTPRM	0.04	6.57	0.29	0.77532
AL590226.2	-0.05	5.89	-0.29	0.77579
ADIPOR1	-0.06	12.81	-0.29	0.77587
TTC5	-0.02	6.93	-0.29	0.77603
PMP22	0.04	5.07	0.29	0.77605
FTH1	0.04	15.51	0.29	0.77606
FRG1BP	-0.03	6.78	-0.29	0.77608
IGHV1OR15-1	-0.03	4.97	-0.29	0.77608
ITPK1	-0.03	11.61	-0.29	0.77609
RPF1	0.02	8.52	0.29	0.77618
DRAXIN	0.05	6.38	0.29	0.77657
OSGEPL1	-0.03	5.99	-0.29	0.77662
MRI1	-0.03	8.17	-0.29	0.77674
ATG14	-0.03	7.46	-0.28	0.77685
AC096533.1	0.03	7.13	0.28	0.77706
BRD7P2	0.03	5.91	0.28	0.77729
RAPGEF6	0.03	7.31	0.28	0.77746
ZNF155	0.03	7.14	0.28	0.77748
SESN1	-0.03	8.36	-0.28	0.77755
ATP6V1E1	-0.02	10.69	-0.28	0.77780
TCL6	0.05	5.82	0.28	0.77782
SMIM30	0.03	6.29	0.28	0.77785
LRRC24	0.03	5.14	0.28	0.77791
FAM177B	0.03	5.21	0.28	0.77793
CYTOR	0.04	7.91	0.28	0.77800
AL157871.3	0.04	5.14	0.28	0.77827
TMEM88	0.05	7.08	0.28	0.77834
CELSR2	-0.04	6.62	-0.28	0.77841
RPS4Y1	-0.23	10.26	-0.28	0.77842
MGAT3	0.04	6.27	0.28	0.77844
TRBJ1-6	-0.04	5.42	-0.28	0.77915
MSRB3	0.03	5.89	0.28	0.77931
CD8B2	-0.04	5.41	-0.28	0.77974
C1R	-0.03	6.26	-0.28	0.77984
GAPDHP40	0.03	6.29	0.28	0.77986
MPC2	0.02	7.89	0.28	0.77987
FMO4	0.02	5.62	0.28	0.77990
DECR1	-0.02	9.26	-0.28	0.77992
SEPTIN7P6	-0.04	5.46	-0.28	0.78011
EIF3E	0.02	10.86	0.28	0.78037
IGLJ3	0.06	5.69	0.28	0.78041
TYROBP	0.04	13.64	0.28	0.78064
FTH1P3	0.02	5.83	0.28	0.78067
TRAV21	-0.03	6.62	-0.28	0.78069
PURB	-0.02	8.51	-0.28	0.78095
PPFIBP1	-0.03	5.06	-0.28	0.78095
AC083899.1	-0.02	5.57	-0.28	0.78099
CNNM2	0.02	6.92	0.28	0.78115

TP73-AS1	0.02	7.84	0.28	0.78133
RNMT	-0.02	8.61	-0.28	0.78138
TLN1	-0.03	14.51	-0.28	0.78139
PEX11A	0.03	5.48	0.28	0.78170
DNTTIP2	-0.02	9.05	-0.28	0.78183
LLCFC1	0.05	5.17	0.28	0.78189
ZNF510	-0.02	6.78	-0.28	0.78203
FOXM1	-0.03	5.60	-0.28	0.78203
CD2BP2-DT	0.03	5.72	0.28	0.78244
LINC01588	-0.03	7.01	-0.28	0.78247
TDRKH-AS1	-0.03	4.99	-0.28	0.78253
AC104390.1	0.03	5.25	0.28	0.78261
LINC02446	0.05	6.39	0.28	0.78285
XRCC6P2	0.03	7.13	0.28	0.78309
TULP4	-0.02	7.56	-0.28	0.78337
GDI2P2	-0.04	5.69	-0.28	0.78342
AC020659.1	0.05	7.02	0.28	0.78360
YWHAEP5	-0.03	5.46	-0.28	0.78363
ACAD10	-0.02	8.62	-0.28	0.78372
POLG	0.01	10.62	0.28	0.78402
PRKAR2B	-0.04	7.91	-0.28	0.78406
ACTBP9	-0.03	6.33	-0.28	0.78414
MTCH1	0.02	11.13	0.28	0.78416
PTK7	-0.03	5.93	-0.28	0.78424
TRAV5	-0.03	5.64	-0.28	0.78424
PPP1R15A	-0.04	10.72	-0.28	0.78428
ADPRM	-0.03	6.91	-0.28	0.78429
ARHGAP5	0.03	6.71	0.28	0.78441
KATNA1	0.02	7.80	0.27	0.78455
AC022182.3	-0.03	5.61	-0.27	0.78462
ACTG1P14	0.04	7.27	0.27	0.78467
SCMH1	0.03	7.43	0.27	0.78470
TBL1X	-0.02	10.91	-0.27	0.78476
ERH	-0.02	9.04	-0.27	0.78496
ZNF839	0.02	8.44	0.27	0.78517
SMIM10L1	-0.02	7.86	-0.27	0.78519
PKMP1	0.04	6.77	0.27	0.78532
TMEM169	-0.03	5.48	-0.27	0.78534
FOXN3-AS1	-0.03	5.61	-0.27	0.78559
SUFU	-0.02	9.14	-0.27	0.78579
SULT1A1	0.05	10.38	0.27	0.78583
HSP90B1	-0.02	11.22	-0.27	0.78605
METRNL	0.04	9.76	0.27	0.78616
AC040977.1	0.03	6.31	0.27	0.78624
TAF1A	0.03	5.36	0.27	0.78627
PSMA6	-0.02	6.04	-0.27	0.78637
ACER1	-0.03	4.98	-0.27	0.78657
PRPF8	-0.02	12.19	-0.27	0.78664
LIPT1	0.03	5.89	0.27	0.78666
ARL16	0.03	8.10	0.27	0.78668

LMBR1L	0.02	9.95	0.27	0.78668
INPP4B	-0.03	7.62	-0.27	0.78674
AL133477.1	-0.03	5.78	-0.27	0.78685
NDUFB9P2	0.04	5.25	0.27	0.78714
VIT	-0.04	5.02	-0.27	0.78717
CALM1	0.02	12.30	0.27	0.78720
ARRB2	-0.03	13.98	-0.27	0.78722
SOCS3	-0.06	9.00	-0.27	0.78737
HSP90AA4P	0.03	5.04	0.27	0.78744
AC004987.2	-0.03	6.00	-0.27	0.78757
GLOD4	-0.02	9.29	-0.27	0.78800
FPR1	-0.05	13.83	-0.27	0.78811
FOLR3	-0.08	9.37	-0.27	0.78821
CEACAM19	-0.04	5.70	-0.27	0.78836
GNG5P2	0.04	5.85	0.27	0.78867
HLX	0.04	8.68	0.27	0.78871
TARS3	-0.02	6.93	-0.27	0.78876
UCK2	0.02	7.15	0.27	0.78887
CES4A	-0.03	6.49	-0.27	0.78892
PYURF	0.02	8.20	0.27	0.78895
LSM11	-0.02	5.97	-0.27	0.78896
SEC22C	-0.02	8.17	-0.27	0.78914
ABRAXAS1	-0.02	6.94	-0.27	0.78914
GDI1	0.02	11.77	0.27	0.78918
TRAK2	-0.04	8.72	-0.27	0.78928
MKRN1	0.05	12.03	0.27	0.78961
UGP2	-0.02	9.70	-0.27	0.78979
PPP1R10	-0.02	10.58	-0.27	0.78980
PSMB2	0.02	9.57	0.27	0.78991
RDX	0.03	6.91	0.27	0.79011
SEMA3G	-0.03	4.94	-0.27	0.79031
MYO1A	-0.03	5.04	-0.27	0.79035
SDHAP3	-0.03	7.09	-0.27	0.79071
DERL2	0.02	8.88	0.27	0.79086
GGA2	-0.02	10.35	-0.27	0.79120
BRMS1L	-0.03	5.35	-0.27	0.79131
THOC5	0.02	10.22	0.27	0.79132
HNRNPAB	0.02	9.88	0.27	0.79137
CPQ	-0.03	9.49	-0.27	0.79137
TBCE	0.02	7.33	0.27	0.79153
TMEM91	0.04	9.00	0.27	0.79158
CAPN12	0.03	6.92	0.27	0.79164
AC004067.1	0.03	5.13	0.27	0.79171
CCL4	-0.04	8.35	-0.27	0.79171
OMA1	-0.02	6.91	-0.27	0.79187
EIF5AP4	0.03	5.80	0.27	0.79205
LRRC37A2	-0.04	6.87	-0.26	0.79208
AC025279.1	-0.03	5.05	-0.26	0.79215
EIF2AK1	0.03	11.12	0.26	0.79217
SLC17A9	0.04	7.36	0.26	0.79241

PFAS	-0.02	7.75	-0.26	0.79270
RGP1	-0.02	8.83	-0.26	0.79270
AC087741.1	-0.05	8.10	-0.26	0.79275
RPSAP3	0.03	5.49	0.26	0.79282
BCKDHB	-0.02	6.21	-0.26	0.79284
GLRX2	0.03	6.08	0.26	0.79285
AP006296.1	0.03	5.71	0.26	0.79308
LINC02422	0.03	5.98	0.26	0.79313
EIF4H	-0.01	11.27	-0.26	0.79317
CLUHP3	-0.04	7.93	-0.26	0.79346
MCM3AP	0.01	10.54	0.26	0.79352
RBM15	-0.02	7.78	-0.26	0.79359
SLC25A20	-0.02	9.25	-0.26	0.79368
ERCC8	-0.03	5.66	-0.26	0.79396
SCCPDH	-0.03	7.42	-0.26	0.79402
AC104297.1	0.03	4.92	0.26	0.79408
ZNF451	-0.02	7.51	-0.26	0.79411
NPM1P25	-0.03	5.07	-0.26	0.79418
AC010326.1	-0.03	6.07	-0.26	0.79425
GGT5	0.04	5.24	0.26	0.79427
UGGT1	0.02	10.05	0.26	0.79441
ACTG1P17	-0.03	5.29	-0.26	0.79447
CFAP20	0.02	8.05	0.26	0.79459
AP003498.1	-0.03	4.92	-0.26	0.79485
ZNF438	-0.04	8.44	-0.26	0.79533
SLC45A3	-0.04	6.88	-0.26	0.79541
EEF1A1P11	0.04	9.31	0.26	0.79570
GUSBP11	-0.05	7.36	-0.26	0.79576
RIOX2	-0.02	7.49	-0.26	0.79586
TRIOBP	0.02	10.42	0.26	0.79586
AP003071.5	0.03	5.11	0.26	0.79608
OLFM4	-0.10	6.04	-0.26	0.79613
PTK2	-0.03	6.80	-0.26	0.79613
NPM1P6	-0.03	5.65	-0.26	0.79662
NUTM2B-AS1	-0.03	7.20	-0.26	0.79670
CD300LB	0.03	9.61	0.26	0.79684
C2orf92	0.03	5.24	0.26	0.79714
ZNF501	-0.03	5.37	-0.26	0.79736
AK9	0.02	5.81	0.26	0.79738
PGGHG	-0.05	12.58	-0.26	0.79745
SCARF2	0.03	5.24	0.26	0.79755
ETFDH	-0.02	7.37	-0.26	0.79767
RAB8A	0.02	11.29	0.26	0.79770
RTTN	0.02	6.65	0.26	0.79802
TADA2A	-0.02	7.23	-0.26	0.79812
TRIM10	-0.06	6.58	-0.26	0.79829
ATP23	-0.03	6.00	-0.26	0.79835
LXN	-0.02	6.35	-0.26	0.79839
SUPV3L1	0.02	7.83	0.26	0.79843
NCF1C	0.04	11.71	0.26	0.79844

PDCD4-AS1	0.02	7.20	0.26	0.79851
NOP9	-0.02	9.03	-0.26	0.79881
SEPHS1	-0.02	8.29	-0.26	0.79898
SWT1	0.02	6.33	0.26	0.79901
DNAJC14	0.02	8.11	0.26	0.79908
TRBV6-2	0.05	6.89	0.26	0.79918
GCC2	-0.03	8.50	-0.26	0.79923
AC021231.2	0.03	5.78	0.26	0.79923
CD83	0.02	7.39	0.26	0.79933
LINC00910	0.03	6.50	0.26	0.79936
ACSS3	0.04	6.21	0.26	0.79940
VDAC3	0.02	9.56	0.26	0.79968
TMEM116	0.02	7.04	0.25	0.79977
MYO15B	-0.05	10.88	-0.25	0.79990
BFSP1	-0.04	5.68	-0.25	0.79995
YWHABP2	-0.04	5.80	-0.25	0.80000
C10orf143	-0.03	6.43	-0.25	0.80000
TOMM40L	-0.02	7.79	-0.25	0.80016
PGAM5	-0.02	8.02	-0.25	0.80018
AC087500.1	-0.03	5.51	-0.25	0.80018
ADGRA2	-0.03	6.34	-0.25	0.80020
SNRNP70	0.03	11.73	0.25	0.80030
MMRN1	-0.04	5.45	-0.25	0.80033
AC104619.3	-0.03	6.40	-0.25	0.80048
MPV17	0.02	8.64	0.25	0.80053
NUTM2B	0.03	5.08	0.25	0.80068
CATSPER2	0.03	5.60	0.25	0.80070
TNFRSF10A	0.02	7.37	0.25	0.80070
PI3	-0.08	9.33	-0.25	0.80075
CCNC	-0.02	7.11	-0.25	0.80080
DHX34	-0.03	10.68	-0.25	0.80108
MYOM2	0.10	7.91	0.25	0.80121
CALU	-0.02	7.67	-0.25	0.80143
FAM20C	0.04	7.98	0.25	0.80160
KIFC1	0.03	5.83	0.25	0.80167
HCLS1	0.03	13.74	0.25	0.80171
ZNNT1	0.03	6.48	0.25	0.80192
AC105749.1	-0.02	5.76	-0.25	0.80220
CYP2E1	0.03	5.18	0.25	0.80241
SUZ12P1	-0.02	6.10	-0.25	0.80247
CLDN9	0.04	6.78	0.25	0.80263
ABLIM1	0.03	10.02	0.25	0.80266
MARCO	-0.05	7.76	-0.25	0.80270
AC048382.6	0.03	5.99	0.25	0.80280
SAP30	-0.03	6.57	-0.25	0.80290
FITM2	0.03	6.36	0.25	0.80296
IGKV2-30	0.06	6.30	0.25	0.80298
POLA2	0.03	6.52	0.25	0.80298
SIN3B	0.02	10.12	0.25	0.80317
KLF8	-0.03	5.81	-0.25	0.80329

TEPSIN	0.03	9.93	0.25	0.80350
H2AZ2P1	0.03	5.95	0.25	0.80356
POMC	0.03	5.52	0.25	0.80368
TLK1	-0.02	8.53	-0.25	0.80378
ROBO3	-0.03	6.71	-0.25	0.80389
S100A11	0.04	13.80	0.25	0.80391
AL008729.1	-0.03	5.46	-0.25	0.80417
MRRF	0.02	7.50	0.25	0.80429
PSME2P6	0.03	5.18	0.25	0.80430
YTHDF2	-0.01	9.25	-0.25	0.80431
SPNS2	-0.03	5.99	-0.25	0.80432
C7orf31	-0.02	6.70	-0.25	0.80446
AC007383.2	-0.03	5.73	-0.25	0.80487
RTF2	0.02	11.12	0.25	0.80497
AC007342.5	0.03	6.01	0.25	0.80502
PLD6	-0.03	6.51	-0.25	0.80514
TERF2IP	0.02	10.05	0.25	0.80516
CCND2	-0.02	10.31	-0.25	0.80520
UCP3	-0.02	6.08	-0.25	0.80544
AC007342.4	0.04	5.37	0.25	0.80548
HINT1P1	0.03	5.46	0.25	0.80556
RPS24P6	0.02	5.04	0.25	0.80572
NPIPB12	-0.03	7.92	-0.25	0.80584
PDIA3	-0.02	11.08	-0.25	0.80588
ZSCAN26	-0.02	7.24	-0.25	0.80595
TNFRSF8	0.03	8.40	0.25	0.80599
PCCB	0.02	7.68	0.25	0.80612
ELANE	0.08	7.05	0.25	0.80614
UPB1	-0.02	5.60	-0.25	0.80616
CENPC	-0.02	7.07	-0.25	0.80631
KANSL2	-0.01	8.16	-0.25	0.80643
ANP32A	-0.02	11.51	-0.25	0.80675
FANCE	-0.02	6.83	-0.25	0.80694
PDHA1	-0.01	8.98	-0.25	0.80698
LINC01003	0.02	6.48	0.25	0.80698
GTF2IP13	-0.03	6.22	-0.25	0.80702
MTND1P23	-0.04	9.62	-0.25	0.80725
AP000944.5	-0.03	5.03	-0.25	0.80735
SP2	0.02	9.66	0.25	0.80741
CLEC4G	0.03	5.72	0.24	0.80750
L3MBTL1	0.03	6.19	0.24	0.80754
ING2	-0.02	6.89	-0.24	0.80760
CEP126	0.03	5.69	0.24	0.80762
RPS24P17	0.03	5.79	0.24	0.80793
LIPC	0.03	5.63	0.24	0.80815
PCNT	-0.02	9.19	-0.24	0.80822
NBAS	0.02	8.96	0.24	0.80826
WASH3P	0.04	7.93	0.24	0.80836
ATP6V0E1P2	-0.02	5.05	-0.24	0.80868
CNKSR2	0.03	5.28	0.24	0.80871

TMEM254	-0.02	7.51	-0.24	0.80873
PLEKHA7	-0.03	5.56	-0.24	0.80873
LINC01237	-0.03	4.92	-0.24	0.80882
STAG3L3	0.03	6.17	0.24	0.80883
CCT6A	-0.02	9.69	-0.24	0.80895
APOL3	0.02	10.70	0.24	0.80895
CRK	-0.03	9.11	-0.24	0.80902
DDR1	0.03	6.68	0.24	0.80906
POLR2M	0.03	7.15	0.24	0.80907
C18orf32	0.02	6.50	0.24	0.80920
BRAP	-0.02	8.33	-0.24	0.80942
PLXNB1	-0.03	5.61	-0.24	0.80949
IP6K2	-0.01	9.72	-0.24	0.80969
REELD1	0.03	5.21	0.24	0.80970
ABHD16A	0.03	6.37	0.24	0.80982
GPR18	0.02	7.67	0.24	0.81004
MAMDC4	-0.03	6.28	-0.24	0.81015
ATP5F1AP2	-0.02	5.42	-0.24	0.81024
NAA35	-0.02	6.88	-0.24	0.81042
BTG1	0.02	12.70	0.24	0.81043
LMBRD1	-0.02	8.94	-0.24	0.81048
LRTOMT	0.03	5.09	0.24	0.81048
MADCAM1	-0.03	5.04	-0.24	0.81057
AC020916.1	-0.04	9.08	-0.24	0.81064
CMPK1	0.02	9.21	0.24	0.81068
BCL9	-0.03	7.46	-0.24	0.81073
TMIGD3	-0.04	5.00	-0.24	0.81079
CNN2P9	0.03	6.51	0.24	0.81081
BPNT1	-0.03	6.71	-0.24	0.81085
MELTF	-0.02	5.05	-0.24	0.81087
UBE3A	0.03	8.56	0.24	0.81095
AC009133.3	0.03	6.36	0.24	0.81130
MRPL32	0.01	7.88	0.24	0.81130
TMBIM6	-0.02	13.20	-0.24	0.81132
BEND2	-0.04	5.88	-0.24	0.81135
CS	0.01	10.49	0.24	0.81143
SPATA5	-0.02	5.86	-0.24	0.81150
VSIG10	0.04	5.71	0.24	0.81159
AC139887.2	-0.03	6.64	-0.24	0.81187
MAP1LC3B2	-0.03	6.56	-0.24	0.81192
TCTN2	-0.02	6.02	-0.24	0.81201
PPP2R5B	0.03	8.73	0.24	0.81220
SLC25A33	0.02	6.06	0.24	0.81229
SPON1	0.03	5.07	0.24	0.81236
RDH13	0.02	6.61	0.24	0.81254
PEAK3	-0.04	8.76	-0.24	0.81259
NCKAP5L	0.03	8.63	0.24	0.81271
PTGDR2	-0.04	8.04	-0.24	0.81279
AFF3	0.04	7.24	0.24	0.81282
ACAA1	0.02	10.94	0.24	0.81285

FBXW7	0.02	8.16	0.24	0.81298
AC005229.4	-0.02	5.40	-0.24	0.81324
CDH24	-0.03	5.26	-0.24	0.81332
LRWD1	-0.03	8.90	-0.24	0.81352
AC097468.3	-0.02	5.31	-0.24	0.81369
PIF1	-0.03	5.70	-0.24	0.81373
FTH1P2	-0.04	9.63	-0.24	0.81374
PSME1	0.02	12.52	0.24	0.81383
TAF5	-0.02	6.09	-0.24	0.81392
CDK13	-0.02	9.40	-0.24	0.81412
AP2B1	0.01	11.18	0.24	0.81414
AC009237.14	0.03	4.98	0.24	0.81416
SNHG26	-0.03	5.24	-0.24	0.81420
RNF227	0.02	5.57	0.24	0.81427
AP3M2	0.02	7.36	0.24	0.81437
PDGFB	0.03	6.90	0.24	0.81448
AC010422.8	-0.03	6.00	-0.24	0.81453
EIF3C	0.03	8.31	0.24	0.81462
MBD5	-0.02	7.51	-0.24	0.81468
INPP5E	0.02	8.04	0.24	0.81488
LDHAP5	-0.03	5.46	-0.23	0.81515
HNRNPRP1	0.03	5.23	0.23	0.81520
IMPDH1	0.03	12.40	0.23	0.81524
KIF9-AS1	-0.02	5.58	-0.23	0.81530
AL121845.1	-0.03	5.06	-0.23	0.81541
RPS6KA5	0.03	8.40	0.23	0.81543
TTC3P1	-0.03	5.71	-0.23	0.81568
CFAP44	0.03	5.87	0.23	0.81580
JPT2	0.02	8.40	0.23	0.81588
VPS26B	0.01	10.24	0.23	0.81588
PITPNM2	0.02	8.77	0.23	0.81593
NIFK-AS1	0.02	6.33	0.23	0.81596
TMX2P1	-0.02	5.14	-0.23	0.81600
PLXNA3	-0.03	8.66	-0.23	0.81606
SGCB	0.02	5.65	0.23	0.81613
IGHV6-1	0.05	6.38	0.23	0.81613
AL356534.1	0.03	5.68	0.23	0.81618
NIBAN2	0.03	9.42	0.23	0.81651
ATXN7L3	0.01	10.56	0.23	0.81660
SEZ6L	-0.03	5.92	-0.23	0.81662
IGHV2-26	-0.05	5.61	-0.23	0.81690
TTC27	0.02	7.39	0.23	0.81697
TYMS	-0.04	6.02	-0.23	0.81710
ZNF790	0.02	5.59	0.23	0.81719
ARID3A	0.03	9.71	0.23	0.81724
UTP3	-0.02	8.77	-0.23	0.81747
AC147651.1	-0.04	6.54	-0.23	0.81768
CYP4V2	0.02	6.88	0.23	0.81769
RIOK1	0.02	7.78	0.23	0.81773
MB21D2	0.03	5.72	0.23	0.81774

NEK1	-0.02	6.29	-0.23	0.81783
SASS6	-0.02	5.22	-0.23	0.81786
AC060766.4	0.03	5.66	0.23	0.81795
PCGF6	0.02	5.57	0.23	0.81800
ARSK	0.02	5.57	0.23	0.81803
TCHP	-0.02	8.27	-0.23	0.81806
TBC1D2	0.02	9.18	0.23	0.81821
PIGBOS1	0.03	7.69	0.23	0.81854
AC115837.1	0.03	8.60	0.23	0.81858
MRPL58	0.02	8.03	0.23	0.81862
ZNF266	0.02	9.15	0.23	0.81864
HELLS	0.03	5.80	0.23	0.81876
RASA4	-0.02	5.71	-0.23	0.81878
KARS1P1	0.03	5.48	0.23	0.81881
VWA5A	0.02	7.10	0.23	0.81883
RNASEH1	-0.02	7.60	-0.23	0.81888
FAM221A	-0.03	5.20	-0.23	0.81904
MYRF	-0.03	5.61	-0.23	0.81904
SLC12A3	0.03	4.97	0.23	0.81905
TSPAN13	0.03	6.60	0.23	0.81906
EIF2AK3-DT	0.03	5.22	0.23	0.81922
LINC00649	-0.03	8.20	-0.23	0.81938
ZBTB11-AS1	-0.03	6.05	-0.23	0.81939
SYNGR3	-0.03	5.17	-0.23	0.81956
SMS	0.02	8.92	0.23	0.81961
WFDC21P	0.04	5.23	0.23	0.81975
ALDH9A1	-0.01	9.18	-0.23	0.81994
RPL10AP1	-0.02	5.01	-0.23	0.82006
CHST2	-0.03	9.44	-0.23	0.82027
GHRL	0.03	7.17	0.23	0.82057
CCDC91	-0.02	7.65	-0.23	0.82070
HLA-V	-0.06	5.22	-0.23	0.82097
TTC28	0.03	5.66	0.23	0.82104
MICAL3	-0.04	6.64	-0.23	0.82121
ZBTB12	0.03	5.04	0.23	0.82121
SGSM1	0.03	5.85	0.23	0.82123
MS4A1	-0.05	9.32	-0.23	0.82128
CDA	-0.04	11.29	-0.23	0.82144
AC108925.1	0.03	5.29	0.23	0.82151
RCAN3	0.03	9.72	0.23	0.82155
UST	0.02	6.19	0.23	0.82187
NETO2	0.02	6.16	0.23	0.82187
MAPT	0.03	5.21	0.23	0.82188
OLFM1	-0.06	6.32	-0.23	0.82190
TNRC6C-AS1	0.02	9.90	0.23	0.82212
SAFB	0.01	10.61	0.23	0.82216
AC009948.3	0.02	5.10	0.23	0.82218
TMEM234	0.02	7.90	0.23	0.82228
MSTO2P	0.03	5.77	0.23	0.82230
MIR762HG	0.02	5.83	0.23	0.82264

NOC2LP1	0.03	5.19	0.23	0.82265
SH2D1A	0.03	8.08	0.23	0.82265
EPB41L4A	0.03	5.46	0.23	0.82268
ZNF565	0.02	5.70	0.23	0.82274
CDCA5	0.02	5.76	0.23	0.82276
AC108673.2	-0.02	5.37	-0.22	0.82287
MT-ND1	0.02	14.67	0.22	0.82303
PRDX1P1	-0.03	5.53	-0.22	0.82309
LRIG1	-0.03	8.21	-0.22	0.82327
HMGN2P4	-0.02	5.38	-0.22	0.82333
FBXL12	0.02	7.91	0.22	0.82333
HNRNPCP3	0.02	5.26	0.22	0.82341
PGM1	0.02	9.05	0.22	0.82364
EID2	-0.02	6.99	-0.22	0.82365
COL9A3	0.04	6.27	0.22	0.82375
PCDH1	-0.04	6.75	-0.22	0.82377
PSMD14	-0.02	7.51	-0.22	0.82387
MTX1P1	0.03	5.28	0.22	0.82404
AL592295.4	-0.02	5.51	-0.22	0.82416
AC244090.1	0.02	5.58	0.22	0.82431
AC007342.3	-0.03	7.53	-0.22	0.82442
AC099063.4	0.02	5.39	0.22	0.82446
PRKCA	-0.03	8.29	-0.22	0.82446
AL596202.1	-0.03	4.93	-0.22	0.82449
ACADVL	0.02	11.60	0.22	0.82465
PPM1M	0.02	10.92	0.22	0.82467
AC244213.1	0.04	4.85	0.22	0.82482
ATG4A	-0.02	7.00	-0.22	0.82485
LYPLAL1	-0.03	6.54	-0.22	0.82489
SH3BP5	-0.02	9.94	-0.22	0.82489
AP003068.2	0.04	7.48	0.22	0.82498
USP12	-0.03	7.88	-0.22	0.82501
LILRB3	0.04	12.71	0.22	0.82501
CFL1P5	-0.02	5.86	-0.22	0.82511
SIGLEC7	-0.03	9.68	-0.22	0.82528
CHML	-0.02	5.75	-0.22	0.82528
TRBJ1-4	-0.03	5.28	-0.22	0.82531
GPALPP1	0.02	6.59	0.22	0.82531
NPIPA1	0.03	6.85	0.22	0.82539
TMEM14C	-0.02	8.22	-0.22	0.82540
RNF130	-0.02	11.43	-0.22	0.82548
ZNF433-AS1	-0.02	5.74	-0.22	0.82553
TMEM64	-0.02	6.51	-0.22	0.82580
MEMO1P1	0.02	5.03	0.22	0.82586
TP53I11	-0.03	10.49	-0.22	0.82588
OSGEP	0.01	9.28	0.22	0.82592
KANK3	-0.03	5.83	-0.22	0.82603
H6PD	-0.02	10.46	-0.22	0.82612
PPA1	0.03	8.69	0.22	0.82624
AC009078.1	-0.06	5.64	-0.22	0.82634

DOCK3	-0.03	5.09	-0.22	0.82636
HNRNPCP2	-0.03	7.97	-0.22	0.82669
ELP2	0.02	8.88	0.22	0.82683
USP53	-0.03	6.14	-0.22	0.82689
HADH	0.02	7.67	0.22	0.82694
Z95114.3	0.03	5.22	0.22	0.82697
LRRC75B	-0.03	5.40	-0.22	0.82698
SLC2A8	0.03	6.64	0.22	0.82706
FSTL4	-0.03	5.00	-0.22	0.82723
LIN52	-0.02	6.72	-0.22	0.82744
HAS3	0.02	5.37	0.22	0.82768
ZCCHC24	-0.02	7.13	-0.22	0.82793
H2AC8	-0.03	5.77	-0.22	0.82793
ANO8	-0.03	7.15	-0.22	0.82798
TDG	0.02	7.26	0.22	0.82802
LRRC8C-DT	-0.02	5.77	-0.22	0.82817
ZBTB7B	0.02	11.11	0.22	0.82818
FCF1	-0.02	8.05	-0.22	0.82819
NPHP4	-0.02	7.62	-0.22	0.82825
TSG101	0.02	8.86	0.22	0.82829
SFTPD	0.03	5.02	0.22	0.82837
MCM2	-0.02	8.05	-0.22	0.82838
ABCA7	0.03	11.45	0.22	0.82839
PARD6B	0.02	5.04	0.22	0.82847
SLC17A5	-0.02	7.42	-0.22	0.82847
SUMO1P3	-0.02	5.25	-0.22	0.82866
GPR153	-0.03	6.35	-0.22	0.82878
PTPRN2	-0.03	7.80	-0.22	0.82892
LILRB5	0.05	6.26	0.22	0.82910
KLRG1	0.03	8.06	0.22	0.82928
MT-ND2	-0.02	14.10	-0.22	0.82939
IGHV4-31	-0.07	6.57	-0.22	0.82940
ARHGAP11A	-0.02	5.58	-0.22	0.82945
VASH1	-0.04	8.60	-0.22	0.82949
TMSB4XP2	0.03	6.07	0.22	0.82960
EPN2	-0.02	6.97	-0.22	0.82964
AC116347.1	0.03	6.89	0.22	0.82987
AC005840.2	-0.03	5.11	-0.22	0.82997
CHRAC1	-0.02	8.36	-0.22	0.83011
CUX2	0.03	5.18	0.22	0.83016
HPS4	0.01	8.86	0.22	0.83023
PODXL	0.03	5.19	0.21	0.83083
BABAM2	0.02	8.73	0.21	0.83092
GRB2	-0.02	12.56	-0.21	0.83092
E2F1	0.02	6.48	0.21	0.83101
AC244034.1	-0.02	5.36	-0.21	0.83124
COX11	0.02	7.25	0.21	0.83137
NACA3P	0.03	8.05	0.21	0.83139
H4C5	-0.02	5.36	-0.21	0.83160
ZNF805	0.02	6.22	0.21	0.83189

AC104763.1	0.03	5.02	0.21	0.83190
PRKAB1	0.01	9.06	0.21	0.83202
TMEM268	-0.02	7.91	-0.21	0.83209
AC007318.1	-0.02	6.25	-0.21	0.83219
ZNF385D	-0.03	5.29	-0.21	0.83234
TMEM231	-0.02	5.10	-0.21	0.83238
NUP214	0.02	11.34	0.21	0.83253
THOC3	0.02	7.04	0.21	0.83255
PIGV	-0.01	7.66	-0.21	0.83259
SCAND2P	-0.02	7.47	-0.21	0.83264
TYMP	-0.03	12.30	-0.21	0.83284
CTIF	-0.02	7.70	-0.21	0.83307
ZDHHC11B	0.03	5.26	0.21	0.83311
AP001994.1	0.03	5.35	0.21	0.83315
AC012306.4	0.02	4.99	0.21	0.83334
DYNLL1P1	0.03	5.27	0.21	0.83350
ARAP1	-0.02	13.65	-0.21	0.83357
AC137932.2	0.03	6.68	0.21	0.83365
ZNF862	-0.02	8.96	-0.21	0.83371
PMS2P3	0.03	5.93	0.21	0.83389
RILPL1	0.03	6.68	0.21	0.83400
KCNC3	-0.03	7.06	-0.21	0.83403
TMSB4XP6	0.03	7.92	0.21	0.83414
EIF3L	0.02	10.91	0.21	0.83431
CNTNAP2	0.03	5.33	0.21	0.83432
AL122018.1	-0.02	5.24	-0.21	0.83436
SCYL3	0.02	7.99	0.21	0.83440
VPS16	0.02	9.91	0.21	0.83442
HNRNPA1P7	-0.03	7.68	-0.21	0.83461
GNAI2P1	0.03	6.79	0.21	0.83469
IL2RA	0.02	7.37	0.21	0.83492
SFXN5	0.02	8.65	0.21	0.83494
CDK2AP1	0.02	8.26	0.21	0.83506
TMEM147-AS1	-0.03	6.89	-0.21	0.83511
CD33	-0.02	9.52	-0.21	0.83524
SEC11B	-0.03	5.96	-0.21	0.83530
C6orf89	-0.01	10.65	-0.21	0.83536
GGNBP2	-0.01	9.78	-0.21	0.83538
PLXNA4	0.03	5.68	0.21	0.83543
ZNF808	0.02	6.57	0.21	0.83554
TCAF1	0.02	6.23	0.21	0.83561
ZFYVE28	-0.02	8.02	-0.21	0.83563
MAP3K6	-0.03	7.74	-0.21	0.83563
AC138409.2	0.03	5.44	0.21	0.83569
RBM4	-0.02	7.34	-0.21	0.83573
NKRF	0.02	6.86	0.21	0.83574
XBP1	0.02	10.70	0.21	0.83576
EEF1A1P25	0.02	5.70	0.21	0.83590
AC016065.1	0.02	5.64	0.21	0.83604
SLC45A1	0.02	5.16	0.21	0.83609

AC104162.2	-0.03	5.75	-0.21	0.83626
AC083798.2	0.02	5.81	0.21	0.83634
AC008429.2	0.02	5.29	0.21	0.83648
AC106795.2	0.03	5.66	0.21	0.83667
PGBD4	0.02	5.52	0.21	0.83679
SMKR1	0.02	5.29	0.21	0.83679
ZNF285	0.02	5.03	0.21	0.83697
ZNF354C	0.02	6.12	0.21	0.83700
TLCD4	0.03	5.06	0.21	0.83701
TERF1	0.02	7.32	0.21	0.83704
GLRX3	0.01	7.93	0.21	0.83711
CYBC1	0.02	12.16	0.21	0.83725
MRPS14	0.02	8.07	0.21	0.83752
EEF1A1P5	0.03	12.31	0.21	0.83778
SEMA4B	-0.02	10.90	-0.21	0.83780
CUX1	0.02	10.78	0.21	0.83784
AURKB	0.02	5.93	0.21	0.83793
NAGA	-0.02	10.48	-0.21	0.83811
OSBPL10	-0.03	6.65	-0.21	0.83817
LYPLA2P2	0.02	5.36	0.21	0.83820
STX1A	0.02	6.55	0.20	0.83868
CLDND2	0.03	6.44	0.20	0.83897
GPRIN1	0.03	5.75	0.20	0.83898
PGAM1P8	0.03	6.18	0.20	0.83902
AC073335.1	-0.02	5.92	-0.20	0.83910
FECH	0.04	8.75	0.20	0.83911
BORCS8	0.02	8.18	0.20	0.83914
GLRX3P2	0.02	5.06	0.20	0.83926
DHPS	-0.03	6.68	-0.20	0.83928
LILRB1	-0.02	10.89	-0.20	0.83931
MARK2	-0.02	11.10	-0.20	0.83936
CEP97	-0.02	5.93	-0.20	0.83965
GPAT4	0.01	10.12	0.20	0.83976
RASA3	0.01	11.55	0.20	0.83979
NOL12	0.03	7.23	0.20	0.83982
AC073130.3	0.03	5.21	0.20	0.83988
C3orf18	0.02	7.31	0.20	0.83995
RALGPS1	0.02	6.81	0.20	0.83996
AL365272.1	-0.03	5.47	-0.20	0.84004
TNFSF8	-0.02	8.18	-0.20	0.84020
MTND4P12	-0.06	7.21	-0.20	0.84023
MAPK7	-0.01	8.40	-0.20	0.84023
FAM168B	-0.01	10.35	-0.20	0.84024
LINC00278	-0.04	4.95	-0.20	0.84032
AC120114.3	-0.03	6.22	-0.20	0.84047
ASNSD1	0.02	7.38	0.20	0.84057
CACNG6	0.03	5.71	0.20	0.84062
F12	0.03	6.00	0.20	0.84070
CYP2T1P	0.03	6.15	0.20	0.84079
LY86-AS1	0.03	5.28	0.20	0.84082

SCP2	-0.02	8.74	-0.20	0.84096
ANKZF1	0.02	10.31	0.20	0.84108
ANP32C	0.03	6.50	0.20	0.84117
CPM	0.02	6.85	0.20	0.84120
GBA	0.02	9.79	0.20	0.84133
BAIAP2-DT	-0.02	8.42	-0.20	0.84137
POLR1B	-0.02	7.96	-0.20	0.84145
HECW2-AS1	0.02	5.32	0.20	0.84148
NASP	-0.01	9.41	-0.20	0.84151
LPP-AS2	0.02	5.29	0.20	0.84160
MAPRE3	-0.02	7.21	-0.20	0.84168
IGKV2-28	0.04	5.82	0.20	0.84171
ZNF507	0.02	7.10	0.20	0.84180
NPIPA5	0.03	6.50	0.20	0.84182
TXNDC12	-0.02	5.87	-0.20	0.84185
CHRM3-AS2	0.04	7.41	0.20	0.84189
FTH1P15	-0.02	5.84	-0.20	0.84197
CDKL1	0.02	5.84	0.20	0.84198
PSMA6P1	-0.03	5.80	-0.20	0.84227
FAM156B	-0.03	5.45	-0.20	0.84228
CLIC1P1	-0.02	7.24	-0.20	0.84255
AP000873.2	-0.03	5.19	-0.20	0.84256
FGFR1	-0.03	7.18	-0.20	0.84259
CHCHD2P6	0.02	5.70	0.20	0.84262
LINC00653	-0.03	5.26	-0.20	0.84278
HADHAP2	0.03	6.42	0.20	0.84290
ZBTB16	-0.03	7.91	-0.20	0.84302
AOC1	-0.05	5.93	-0.20	0.84305
KCNC4	-0.02	6.29	-0.20	0.84305
AL162253.2	0.03	5.05	0.20	0.84317
IQCG	-0.02	6.18	-0.20	0.84327
MAP7	-0.03	5.81	-0.20	0.84335
SURF4	0.01	10.76	0.20	0.84347
RASGEF1A	-0.02	7.26	-0.20	0.84353
CMTM2	-0.04	10.74	-0.20	0.84354
TAOK2	0.01	9.98	0.20	0.84367
UBQLN4	0.01	8.81	0.20	0.84371
AC124798.1	-0.02	5.35	-0.20	0.84373
AC104958.2	0.02	5.71	0.20	0.84380
CACNA2D2	-0.03	7.75	-0.20	0.84389
FAM124B	-0.02	5.09	-0.20	0.84389
UBXN8	-0.02	5.87	-0.20	0.84400
PKNOX1	-0.02	8.31	-0.20	0.84417
MAP3K14-AS1	0.02	6.37	0.20	0.84441
CFP	0.02	9.98	0.20	0.84455
PSMA3	-0.01	8.61	-0.20	0.84488
SLC25A35	-0.02	6.86	-0.20	0.84493
RPA2	-0.02	9.96	-0.20	0.84499
AC009961.1	0.02	5.16	0.20	0.84500
NRP1	0.03	5.49	0.20	0.84511

XRRA1	-0.03	6.57	-0.20	0.84533
KLHL14	0.03	5.47	0.20	0.84546
MAP4	0.01	10.27	0.20	0.84548
PNO1	0.02	6.52	0.20	0.84548
FAM193B	-0.03	10.65	-0.20	0.84554
MYL12BP1	-0.03	6.23	-0.20	0.84559
AC126474.2	-0.02	7.11	-0.20	0.84561
WASF2	0.02	11.96	0.20	0.84563
SCARB1	-0.02	7.65	-0.20	0.84573
AFMID	0.02	6.76	0.20	0.84577
SHROOM1	-0.04	6.97	-0.20	0.84580
PEX19	0.01	8.39	0.20	0.84581
MEX3D	0.02	5.93	0.20	0.84584
PLIN2	0.01	9.20	0.20	0.84595
IL7R	0.03	11.63	0.20	0.84612
ZNF888	-0.02	6.43	-0.19	0.84629
OTOF	-0.04	5.92	-0.19	0.84651
IL10RB	-0.02	6.25	-0.19	0.84659
FAM20A	0.03	5.19	0.19	0.84665
RPP38	-0.01	7.99	-0.19	0.84687
MPP2	-0.03	5.21	-0.19	0.84691
SMN2	-0.02	5.85	-0.19	0.84698
SLC7A6OS	-0.01	8.73	-0.19	0.84724
AL121871.1	0.03	7.06	0.19	0.84724
MICALL2	-0.02	5.90	-0.19	0.84730
POLR3D	-0.01	8.00	-0.19	0.84732
SCARNA20	-0.03	5.02	-0.19	0.84744
AC005244.1	0.02	5.24	0.19	0.84749
RPA3	-0.02	7.26	-0.19	0.84752
SZT2	-0.01	10.29	-0.19	0.84767
DIS3	0.02	8.13	0.19	0.84781
TNF	-0.02	7.79	-0.19	0.84782
NBR2	0.02	6.65	0.19	0.84820
ALG12	0.01	8.84	0.19	0.84837
ATP2B1-AS1	-0.02	5.70	-0.19	0.84849
GUF1	0.02	6.87	0.19	0.84855
YIPF1	0.02	9.20	0.19	0.84863
CD9	-0.04	8.42	-0.19	0.84884
VLDLR	0.03	5.26	0.19	0.84891
U2AF1	0.03	6.60	0.19	0.84893
RPL21P123	-0.03	5.08	-0.19	0.84898
RPL34P33	0.03	6.10	0.19	0.84901
KALRN	0.02	5.80	0.19	0.84909
AL158206.1	-0.02	5.47	-0.19	0.84910
SGSM3	-0.02	5.89	-0.19	0.84921
COL9A2	0.04	7.59	0.19	0.84931
MTURN	0.02	9.18	0.19	0.84933
SDCCAG8	0.01	7.85	0.19	0.84938
RALA	0.01	8.19	0.19	0.84939
ZNF584	-0.02	6.32	-0.19	0.84941

WDR97	0.03	5.73	0.19	0.84945
HSPE1	0.02	6.63	0.19	0.84948
SLC22A18	-0.03	8.70	-0.19	0.84951
STAT5A	0.01	11.63	0.19	0.84952
SLC35E1	0.01	9.21	0.19	0.84963
NSUN6	-0.02	6.42	-0.19	0.84970
TMEM128	0.01	6.94	0.19	0.84997
ARHGAP32	0.02	6.44	0.19	0.85007
VSTM1	0.04	8.11	0.19	0.85021
NDUFAF6	-0.02	6.00	-0.19	0.85021
ZNF766	-0.01	8.19	-0.19	0.85045
COL11A2	0.02	5.09	0.19	0.85060
ARID4B	0.02	9.61	0.19	0.85069
RPS26P3	-0.04	6.50	-0.19	0.85085
MRPL48	0.02	7.64	0.19	0.85088
LRCH3	0.01	8.93	0.19	0.85090
AL513477.1	-0.02	5.52	-0.19	0.85091
ZC2HC1A	0.02	5.20	0.19	0.85094
CD248	0.04	6.31	0.19	0.85138
FAM153A	0.04	6.18	0.19	0.85142
SERBP1	0.01	10.45	0.19	0.85144
FN3KRP	0.02	8.41	0.19	0.85148
ELMOD3	0.01	8.81	0.19	0.85149
TRDV2	0.04	5.88	0.19	0.85149
TDRKH	0.02	6.94	0.19	0.85156
EMBP1	0.03	6.01	0.19	0.85161
TPRG1	-0.03	6.60	-0.19	0.85161
RNF214	0.01	8.19	0.19	0.85172
ZNF841	-0.02	6.60	-0.19	0.85180
SLC2A1	0.03	9.68	0.19	0.85180
TNFSF4	-0.02	6.03	-0.19	0.85192
PIAS2	-0.02	7.32	-0.19	0.85194
LUC7L	0.02	8.86	0.19	0.85208
SNTB2	-0.02	7.50	-0.19	0.85208
GGCT	0.02	6.52	0.19	0.85218
PLCB3	0.02	8.65	0.19	0.85225
PAOX	-0.02	4.96	-0.19	0.85232
AC118344.4	-0.03	5.45	-0.19	0.85243
UACA	0.02	5.43	0.19	0.85268
LIX1L	-0.01	8.67	-0.19	0.85279
SEMA6C	0.02	6.08	0.19	0.85282
MIPEP	-0.02	6.15	-0.19	0.85290
TCP11L1	-0.02	6.43	-0.19	0.85297
HMGN3	0.02	8.48	0.19	0.85299
NR6A1	0.02	7.01	0.19	0.85300
TRBV29-1	0.03	7.61	0.19	0.85308
CEBPA-DT	0.02	5.40	0.19	0.85309
AC092428.1	0.02	4.99	0.19	0.85325
AC245452.1	-0.02	5.85	-0.19	0.85327
NELL2	0.03	8.87	0.19	0.85337

NRG1	0.04	7.02	0.19	0.85339
SLIT1	0.02	5.42	0.19	0.85346
NUDT9	-0.01	7.30	-0.19	0.85352
ATG7	-0.01	9.44	-0.19	0.85356
KANK1	0.03	6.35	0.19	0.85388
ZWILCH	0.02	5.71	0.19	0.85393
ISCA1	-0.02	7.46	-0.18	0.85416
RASD1	-0.02	5.04	-0.18	0.85418
DBIP1	0.02	5.57	0.18	0.85422
TMEM251	0.02	6.19	0.18	0.85433
AC020658.1	0.01	5.49	0.18	0.85442
EIF4BP7	-0.02	6.51	-0.18	0.85448
AL590764.1	0.02	5.96	0.18	0.85457
CKMT2-AS1	-0.02	6.02	-0.18	0.85478
LYSMD1	0.02	5.36	0.18	0.85487
TTC7B	-0.03	6.69	-0.18	0.85488
PLTP	0.02	6.29	0.18	0.85497
WDR45P1	-0.03	6.38	-0.18	0.85515
NCF1B	-0.03	10.73	-0.18	0.85521
TAF15	0.02	10.21	0.18	0.85532
COL5A3	0.06	6.43	0.18	0.85539
KMT2B	-0.01	11.08	-0.18	0.85541
SKA2	0.02	6.68	0.18	0.85550
IGBP1P1	-0.02	5.24	-0.18	0.85552
SUMO2	-0.01	9.65	-0.18	0.85616
CD8A	0.03	10.61	0.18	0.85634
BAK1P1	-0.02	5.34	-0.18	0.85637
ACTA2	-0.03	7.44	-0.18	0.85646
AL353807.3	-0.02	5.45	-0.18	0.85677
METTL5	0.02	7.16	0.18	0.85680
RGL4	0.03	9.61	0.18	0.85684
AL627171.3	0.02	5.12	0.18	0.85698
AC092117.1	-0.02	5.55	-0.18	0.85699
MAP3K12	-0.02	7.98	-0.18	0.85699
PGD	-0.02	12.52	-0.18	0.85718
AC024451.2	0.02	5.54	0.18	0.85727
CHMP4BP1	0.02	6.21	0.18	0.85735
DGCR11	-0.02	5.24	-0.18	0.85750
POM121C	-0.01	9.86	-0.18	0.85758
PTDSS1	0.01	10.30	0.18	0.85761
SUMF1	-0.01	8.75	-0.18	0.85765
AC010247.1	0.02	5.58	0.18	0.85771
KDM4B	-0.02	10.76	-0.18	0.85813
ZNF343	-0.01	6.65	-0.18	0.85818
SPATA33	0.02	6.49	0.18	0.85823
DCHS1	-0.03	7.53	-0.18	0.85824
TENT5A	-0.02	8.75	-0.18	0.85831
LRRC42	0.02	6.93	0.18	0.85836
NFKBIA	-0.02	10.63	-0.18	0.85855
FTH1P11	-0.03	8.45	-0.18	0.85856

ANKRD13B	-0.02	5.62	-0.18	0.85856
ABI2	-0.01	7.03	-0.18	0.85858
AC007238.1	0.02	7.23	0.18	0.85879
SPACA6	-0.03	6.37	-0.18	0.85885
AC022098.1	0.02	7.07	0.18	0.85895
ATP7B	0.02	5.30	0.18	0.85906
RDH14	-0.02	6.33	-0.18	0.85915
AC132872.5	-0.03	5.60	-0.18	0.85933
AC010327.6	0.02	6.38	0.18	0.85997
TMEM97	0.02	5.81	0.18	0.86008
AC005786.3	-0.03	5.54	-0.18	0.86021
GINS2	-0.02	5.57	-0.18	0.86025
BCL2	0.02	9.10	0.18	0.86026
GAS8	0.01	6.48	0.18	0.86031
AFTPH	0.01	9.65	0.18	0.86070
TPM4	-0.01	11.49	-0.18	0.86078
LINC02362	-0.02	5.01	-0.18	0.86082
TM7SF3	0.01	8.47	0.18	0.86093
FUCA2	-0.02	8.20	-0.18	0.86118
LINC02084	0.02	7.20	0.18	0.86121
AQP1	0.03	5.76	0.18	0.86122
SRPRB	0.02	8.75	0.18	0.86139
YEATS4	0.02	6.57	0.18	0.86149
SMYD2	-0.01	7.90	-0.18	0.86162
WIZ	0.01	8.82	0.18	0.86165
HSD17B7P2	0.02	5.72	0.18	0.86172
XKR8	-0.02	9.97	-0.17	0.86179
CISH	0.02	9.64	0.17	0.86181
IL10RA	0.01	12.23	0.17	0.86184
IL11RA	0.03	8.59	0.17	0.86188
POLR3C	0.01	8.59	0.17	0.86188
MMP25	-0.04	13.37	-0.17	0.86194
TTC38	0.02	9.73	0.17	0.86197
CBR3-AS1	0.02	5.38	0.17	0.86200
IL4I1	-0.02	6.55	-0.17	0.86200
IPO5P1	0.02	6.29	0.17	0.86201
ERICH6-AS1	0.02	5.35	0.17	0.86206
STEAP3	-0.02	6.71	-0.17	0.86209
RPS17	-0.02	6.02	-0.17	0.86224
IKZF4	-0.02	6.30	-0.17	0.86231
TIGIT	-0.02	8.92	-0.17	0.86233
MGST2	-0.02	7.88	-0.17	0.86233
AARS2	-0.01	8.09	-0.17	0.86236
PRXL2C	-0.02	6.84	-0.17	0.86243
IQCE	-0.02	8.55	-0.17	0.86255
AC015802.6	0.03	7.44	0.17	0.86259
WBP1	0.02	6.76	0.17	0.86273
ZNF226	0.01	7.63	0.17	0.86289
SLC25A51	-0.02	6.10	-0.17	0.86289
NACA2	0.02	8.63	0.17	0.86300

NRROS	-0.02	9.76	-0.17	0.86302
TRIP6	0.02	7.20	0.17	0.86303
NT5C3B	0.02	7.25	0.17	0.86322
FOXK1	0.01	9.32	0.17	0.86324
SAP18P2	0.02	5.28	0.17	0.86325
HSBP1	0.01	9.43	0.17	0.86347
LRRC75A	0.02	7.06	0.17	0.86359
RINT1	-0.02	7.08	-0.17	0.86359
PDZD11	-0.01	7.49	-0.17	0.86389
SNX12	0.01	8.59	0.17	0.86405
APOBEC3F	0.02	8.06	0.17	0.86422
XKR6	0.02	5.48	0.17	0.86426
HLA-H	-0.05	12.95	-0.17	0.86439
ARPC3P5	0.02	5.68	0.17	0.86443
IER5L	0.02	7.41	0.17	0.86450
RPL5P9	-0.02	5.36	-0.17	0.86456
CEBPZ	-0.01	8.50	-0.17	0.86456
RYR1	0.02	6.82	0.17	0.86460
TMCO6	0.01	8.42	0.17	0.86466
CD300C	-0.02	8.75	-0.17	0.86467
SQLE	-0.02	6.21	-0.17	0.86474
ASS1P1	-0.03	5.44	-0.17	0.86504
RPGRIP1	-0.02	7.28	-0.17	0.86508
RPL17P34	0.02	5.15	0.17	0.86508
AC007569.1	0.03	5.56	0.17	0.86524
HIPK2	-0.02	9.15	-0.17	0.86534
AL355581.1	-0.02	5.37	-0.17	0.86543
SLC46A2	-0.02	7.85	-0.17	0.86573
RABGGTB	0.01	7.88	0.17	0.86576
FAM219B	-0.01	8.80	-0.17	0.86580
AL603766.1	-0.02	5.45	-0.17	0.86599
HNRNPA1P52	0.02	5.14	0.17	0.86632
ACTR3B	0.02	6.80	0.17	0.86632
PDE4D	0.02	6.72	0.17	0.86636
STK17A	-0.01	9.33	-0.17	0.86644
TREX2	-0.02	5.37	-0.17	0.86659
IMMP2L	0.02	5.59	0.17	0.86659
GPR84	0.03	5.74	0.17	0.86667
FAM118B	0.01	7.73	0.17	0.86669
AC114491.1	-0.02	5.59	-0.17	0.86674
CSNK1D	-0.01	12.12	-0.17	0.86689
PDIA5	0.02	6.93	0.17	0.86721
SLFN12L	0.02	6.05	0.17	0.86733
AL732372.3	-0.03	6.41	-0.17	0.86734
C11orf54	-0.02	7.42	-0.17	0.86737
EIF2S3	0.01	10.27	0.17	0.86753
PTPRS	0.03	6.47	0.17	0.86778
SUSD4	0.02	6.44	0.17	0.86790
DPEP3	0.03	8.12	0.17	0.86793
L3MBTL2-AS1	-0.02	5.28	-0.17	0.86808

TRAV14DV4	0.03	6.04	0.17	0.86833
EXOSC10	-0.01	9.88	-0.17	0.86874
KLHL42	-0.01	7.01	-0.17	0.86874
MRPL42	-0.01	7.51	-0.17	0.86875
S1PR5	0.03	9.72	0.17	0.86898
RPH3AL	-0.02	6.20	-0.17	0.86900
IFI27	-0.04	6.17	-0.17	0.86906
NYNRIN	-0.02	5.02	-0.17	0.86912
AC055716.2	0.02	5.79	0.17	0.86913
PRF1	0.02	13.00	0.17	0.86924
TMCO1	-0.01	8.89	-0.17	0.86932
SBDS	-0.01	8.06	-0.17	0.86933
PTOV1-AS2	0.02	7.91	0.17	0.86942
SRRM1	0.01	9.92	0.17	0.86943
EEF1A1P17	-0.01	5.26	-0.16	0.86976
EEF2K	0.01	7.58	0.16	0.86990
AXIN2	-0.02	6.97	-0.16	0.87013
MPP1	-0.03	11.46	-0.16	0.87018
PDLIM1P1	0.02	5.03	0.16	0.87047
VKORC1L1	-0.01	7.68	-0.16	0.87053
DHRS12	-0.03	8.31	-0.16	0.87065
AL118558.3	-0.02	5.09	-0.16	0.87068
AC008014.1	-0.02	5.15	-0.16	0.87080
ZNF592	-0.01	10.77	-0.16	0.87096
AL359183.1	0.02	5.15	0.16	0.87114
ATAD5	-0.02	5.04	-0.16	0.87114
RXRA	-0.02	12.24	-0.16	0.87117
LARS1	-0.01	9.22	-0.16	0.87129
C17orf100	-0.02	5.35	-0.16	0.87135
CCZ1	0.02	7.54	0.16	0.87139
ZNF740	-0.01	9.48	-0.16	0.87159
RIOK2	0.01	7.22	0.16	0.87163
RUNDC3A	0.05	9.11	0.16	0.87167
NDUFB3	0.02	8.06	0.16	0.87170
IGHV3-66	-0.03	6.31	-0.16	0.87173
OSGIN1	-0.02	6.38	-0.16	0.87184
NAP1L3	0.02	5.33	0.16	0.87184
CTNNAL1	0.03	5.81	0.16	0.87186
PTMAP2	-0.02	8.19	-0.16	0.87205
SNRNP40	0.01	9.07	0.16	0.87205
ICAM4	0.02	6.90	0.16	0.87216
LINC00565	-0.02	6.15	-0.16	0.87228
PTK6	-0.01	5.33	-0.16	0.87233
DSC2	0.04	7.44	0.16	0.87240
CEP290	-0.02	6.47	-0.16	0.87245
CERS6	0.01	7.56	0.16	0.87257
SOAT2	0.02	5.12	0.16	0.87274
MT-CYB	-0.01	14.64	-0.16	0.87280
EIF2S1	-0.01	8.30	-0.16	0.87280
EP400P1	-0.02	7.28	-0.16	0.87353

ZNF525	-0.02	6.63	-0.16	0.87357
RTL5	-0.02	6.56	-0.16	0.87379
HEXA	-0.01	9.52	-0.16	0.87391
SH3BP4	-0.02	5.85	-0.16	0.87398
AC092375.2	-0.02	4.99	-0.16	0.87399
ADORA2B	-0.02	5.26	-0.16	0.87402
AC026462.1	-0.03	6.84	-0.16	0.87404
EML5	-0.02	5.10	-0.16	0.87406
TPM3P9	0.01	7.07	0.16	0.87414
ZNF670	-0.02	5.06	-0.16	0.87416
HMGN1P38	0.02	5.83	0.16	0.87422
AL451165.2	0.02	5.96	0.16	0.87424
TNFRSF1A	-0.02	12.64	-0.16	0.87434
SPECC1	-0.01	8.52	-0.16	0.87479
TRIM32	0.01	7.10	0.16	0.87487
Z83745.1	0.02	5.07	0.16	0.87491
EID3	-0.01	5.81	-0.16	0.87495
OFD1	-0.01	8.79	-0.16	0.87514
LAMTOR5	0.02	8.93	0.16	0.87517
JCHAIN	-0.05	8.73	-0.16	0.87558
CD63	-0.02	11.61	-0.16	0.87559
AC139795.2	0.02	5.39	0.16	0.87561
XCL1	0.04	6.23	0.16	0.87576
MYBPH	0.03	6.41	0.16	0.87586
MT-RNR1	0.02	15.51	0.16	0.87589
PARP10	-0.02	10.35	-0.16	0.87590
CFL1P2	0.02	6.99	0.16	0.87601
CDKN1C	0.03	8.41	0.16	0.87607
VCL	0.02	11.10	0.16	0.87615
IL5RA	-0.02	7.03	-0.16	0.87615
XKR7	0.02	5.01	0.16	0.87617
SNORC	0.01	5.12	0.16	0.87624
TTC39C-AS1	0.02	5.20	0.16	0.87628
TRBV3-1	0.03	7.12	0.16	0.87641
NINL	0.02	5.45	0.16	0.87646
IFFO1	0.02	10.00	0.16	0.87681
FLII	0.01	12.58	0.16	0.87691
GALNT6	-0.02	8.80	-0.16	0.87702
AL662844.4	-0.02	8.37	-0.16	0.87709
ATP1A3	0.02	5.87	0.16	0.87710
TMEM94	0.01	10.03	0.16	0.87737
MT-RNR2	-0.02	17.45	-0.16	0.87739
SLC25A37	-0.03	14.49	-0.15	0.87749
RPL12P27	-0.02	5.75	-0.15	0.87767
PACC1	-0.01	7.06	-0.15	0.87771
ZNF470	-0.01	5.65	-0.15	0.87780
HEMGN	0.03	7.59	0.15	0.87782
GABPB1-IT1	0.01	7.91	0.15	0.87785
CUL5	-0.01	7.26	-0.15	0.87802
PTCD3	-0.01	8.46	-0.15	0.87813

GPR137B	-0.01	7.25	-0.15	0.87815
ITGAL	0.01	13.45	0.15	0.87825
MCOLN2	-0.02	6.78	-0.15	0.87831
CASP9	0.01	8.23	0.15	0.87838
PSME3IP1	0.01	10.39	0.15	0.87864
ACTBP11	0.02	7.03	0.15	0.87871
PTPRK	0.02	5.36	0.15	0.87909
ENO3	-0.02	7.04	-0.15	0.87923
AL589843.2	0.02	5.29	0.15	0.87939
SCOC	-0.01	6.68	-0.15	0.87958
LYNX1	-0.03	6.63	-0.15	0.87971
RPL4P3	-0.02	5.90	-0.15	0.87983
TMSB4XP8	-0.02	10.83	-0.15	0.87989
ANGEL1	-0.01	7.94	-0.15	0.87993
PHETA2	0.02	6.43	0.15	0.87996
THAP2	-0.02	5.63	-0.15	0.88002
AC010618.3	0.01	5.20	0.15	0.88003
ZNF503	-0.02	5.49	-0.15	0.88019
ZNF320	-0.01	6.94	-0.15	0.88041
BMS1	-0.01	8.89	-0.15	0.88043
SPRYD4	0.02	6.58	0.15	0.88047
OPLAH	0.03	6.77	0.15	0.88051
HNRNPA1P12	-0.01	4.99	-0.15	0.88063
FTH1P5	0.02	7.46	0.15	0.88070
ZNF121	-0.01	7.68	-0.15	0.88084
SPC24	-0.02	5.04	-0.15	0.88088
IGLV1-40	-0.04	7.90	-0.15	0.88096
CEBPD	-0.03	10.52	-0.15	0.88105
NSUN5P1	-0.03	8.99	-0.15	0.88114
AP002954.1	0.03	5.55	0.15	0.88116
RPL30P3	-0.02	5.92	-0.15	0.88126
DPH2	0.01	8.06	0.15	0.88143
SSPO	0.02	5.69	0.15	0.88153
AC099796.1	0.02	6.00	0.15	0.88196
TRIT1	0.01	7.14	0.15	0.88200
MVB12B	-0.01	7.72	-0.15	0.88204
MED24	0.01	9.93	0.15	0.88206
SPATA20	0.02	8.62	0.15	0.88210
IPO9	-0.01	8.93	-0.15	0.88216
SLC4A1APP1	0.01	4.94	0.15	0.88225
TMSB4XP1	0.02	6.55	0.15	0.88236
NHLRC1	0.02	5.33	0.15	0.88244
RAB11FIP4	0.01	10.62	0.15	0.88278
AC040970.1	0.02	6.79	0.15	0.88280
SNHG12	-0.02	7.70	-0.15	0.88289
CLHC1	-0.02	5.21	-0.15	0.88316
MRPS25	0.01	8.48	0.15	0.88318
INKA2-AS1	0.02	5.81	0.15	0.88324
BCAR3	0.02	5.46	0.15	0.88324
DNMT1	0.01	10.49	0.15	0.88351

ATG5	-0.01	7.97	-0.15	0.88365
RPL5P23	-0.02	6.90	-0.15	0.88384
MROH6	0.02	7.58	0.15	0.88396
AC091982.2	-0.02	5.07	-0.15	0.88405
TMEM230	-0.01	9.17	-0.15	0.88414
C3orf86	0.03	8.15	0.15	0.88425
CD101	-0.02	8.96	-0.15	0.88456
AC104117.2	-0.02	5.80	-0.15	0.88485
MLC1	-0.02	8.74	-0.15	0.88501
GTSE1	-0.02	5.34	-0.15	0.88505
IGHV1-69D	-0.04	6.86	-0.15	0.88522
HLA-F	0.01	12.87	0.14	0.88542
TRIM35	-0.01	8.29	-0.14	0.88555
PAQR4	-0.02	7.16	-0.14	0.88556
KCNN4	0.02	8.58	0.14	0.88560
ZNF814	0.02	6.63	0.14	0.88561
MT-TE	-0.01	5.09	-0.14	0.88561
AC079922.2	0.01	6.48	0.14	0.88569
MTFR1	0.01	5.78	0.14	0.88576
AC129492.5	0.01	5.05	0.14	0.88585
CEBPA	-0.02	9.61	-0.14	0.88593
FBXO21	-0.01	7.97	-0.14	0.88606
AGA	-0.01	7.18	-0.14	0.88608
FHOD1	-0.01	9.91	-0.14	0.88624
CEP152	-0.01	6.38	-0.14	0.88633
FOXN3	-0.01	10.24	-0.14	0.88635
C1QC	0.02	5.36	0.14	0.88640
CSNK1G2P1	-0.01	5.10	-0.14	0.88651
SDSL	-0.02	6.25	-0.14	0.88666
GSEC	-0.03	6.80	-0.14	0.88693
FDPSP1	-0.02	5.08	-0.14	0.88694
LCMT2	-0.01	6.79	-0.14	0.88708
RARA-AS1	-0.02	7.38	-0.14	0.88722
AC009022.1	-0.02	5.47	-0.14	0.88730
CEP41	-0.01	6.83	-0.14	0.88734
AMBRA1	0.01	9.29	0.14	0.88770
SUB1	0.01	9.71	0.14	0.88786
MYLK-AS1	0.01	4.98	0.14	0.88787
CTTN	0.02	8.41	0.14	0.88788
SRP72	0.01	9.30	0.14	0.88798
SLC37A2	-0.02	9.26	-0.14	0.88800
TTTY14	-0.03	4.98	-0.14	0.88807
AL133444.1	0.02	5.22	0.14	0.88811
S100A8	-0.03	14.88	-0.14	0.88829
FCN1	0.02	14.72	0.14	0.88836
SAMD12	-0.02	5.03	-0.14	0.88836
PIK3R3	0.02	5.96	0.14	0.88843
MID1IP1	0.01	10.31	0.14	0.88849
SCAF4	0.01	9.34	0.14	0.88891
YRDC	-0.01	6.95	-0.14	0.88893

RFC4	-0.01	6.32	-0.14	0.88909
MOCS3	0.01	6.95	0.14	0.88932
CDC20	0.02	5.79	0.14	0.88959
AL135818.1	-0.01	6.86	-0.14	0.88962
IFT74	-0.01	5.74	-0.14	0.88968
DNASE1L2	0.02	5.56	0.14	0.88984
ZNF880	0.01	6.34	0.14	0.88989
REXO5	0.01	5.26	0.14	0.89000
MID2	0.02	5.50	0.14	0.89005
ARL17B	-0.03	5.76	-0.14	0.89019
JAZF1	0.02	9.19	0.14	0.89026
ROPN1L	0.03	7.28	0.14	0.89034
TUBBP5	-0.02	5.13	-0.14	0.89048
RALGPS2	-0.02	7.90	-0.14	0.89061
KHSRPP1	-0.02	5.18	-0.14	0.89076
AC004943.2	0.01	5.22	0.14	0.89079
ARHGAP6	-0.02	5.64	-0.14	0.89083
TKT	0.01	13.92	0.14	0.89090
ZNF16	-0.01	6.76	-0.14	0.89119
DCAF12	0.03	11.35	0.14	0.89120
BPGM	-0.03	8.26	-0.14	0.89130
CCDC136	0.01	5.97	0.14	0.89149
ZNF467	0.02	10.71	0.14	0.89150
B3GNTL1	0.02	9.01	0.14	0.89166
IFT122	-0.01	8.07	-0.14	0.89175
CKAP4	0.02	10.63	0.14	0.89183
AL354714.2	-0.02	5.48	-0.14	0.89197
SPAG8	0.01	5.42	0.14	0.89206
FIG4	-0.01	8.57	-0.14	0.89208
CCDC191	-0.01	6.46	-0.14	0.89211
VASP	0.02	12.47	0.14	0.89213
MRPL13	-0.01	7.23	-0.14	0.89220
GRPEL2	-0.01	6.93	-0.14	0.89225
AMIGO2	-0.01	6.89	-0.14	0.89227
DNAJC5	0.01	11.60	0.14	0.89228
AC018755.5	-0.02	5.06	-0.14	0.89294
AC093227.1	0.02	5.04	0.14	0.89303
VAPA	-0.01	9.64	-0.14	0.89311
DCP1B	0.01	7.70	0.13	0.89376
AC138150.2	-0.02	6.20	-0.13	0.89377
TIMM10	-0.02	7.96	-0.13	0.89379
EIF2B3	0.01	7.39	0.13	0.89401
MAPKAPK2	0.01	11.50	0.13	0.89408
CEACAM6	0.04	5.83	0.13	0.89430
LETM1	-0.01	8.94	-0.13	0.89437
AL365361.1	-0.01	7.70	-0.13	0.89455
RRP36	0.01	8.86	0.13	0.89457
LINC00672	0.02	5.94	0.13	0.89467
RPL10P9	-0.04	10.19	-0.13	0.89467
SSB	0.01	8.99	0.13	0.89470

VIPAS39	0.01	8.41	0.13	0.89485
PBX1	-0.02	6.95	-0.13	0.89488
GPR15	0.03	5.68	0.13	0.89504
CNOT4	-0.01	7.65	-0.13	0.89533
EAF2	0.02	6.36	0.13	0.89563
MOAP1	0.01	9.00	0.13	0.89573
PSME2P2	-0.02	6.64	-0.13	0.89580
GNPNAT1	0.01	6.54	0.13	0.89603
AC127502.1	0.02	5.03	0.13	0.89611
ILF2P1	0.01	5.12	0.13	0.89651
INSR	0.02	7.57	0.13	0.89653
TSPYL1	0.01	9.80	0.13	0.89659
AC008537.3	0.01	5.09	0.13	0.89678
ALDH5A1	-0.02	7.43	-0.13	0.89719
ZP3	0.02	6.13	0.13	0.89731
TSPYL5	0.02	5.50	0.13	0.89731
AL356968.2	0.03	6.04	0.13	0.89734
RPS26P8	0.03	6.73	0.13	0.89739
CAND2	0.02	5.25	0.13	0.89762
ZC3H4	-0.01	9.73	-0.13	0.89763
AP002748.4	0.01	5.76	0.13	0.89781
AC124068.1	-0.02	5.28	-0.13	0.89785
LOXL1	-0.02	5.22	-0.13	0.89788
ZNF273	-0.01	5.49	-0.13	0.89796
PRPF4	0.01	8.82	0.13	0.89796
TAF9	-0.01	8.11	-0.13	0.89815
CCR9	-0.02	5.68	-0.13	0.89820
PLGRKT	0.01	6.85	0.13	0.89827
ECHDC3	0.02	6.76	0.13	0.89829
PTPRA	0.01	10.03	0.13	0.89831
WASH8P	0.03	8.62	0.13	0.89832
CFL1P4	-0.01	5.70	-0.13	0.89836
TEF	-0.01	7.59	-0.13	0.89850
CATIP-AS1	-0.02	5.42	-0.13	0.89850
NINJ1	-0.02	11.69	-0.13	0.89860
SNHG10	0.02	5.85	0.13	0.89874
PCGF1	0.01	7.91	0.13	0.89896
EFCAB2	-0.02	6.18	-0.13	0.89901
TTC16	0.02	8.04	0.13	0.89906
PPM1B	-0.01	8.54	-0.13	0.89912
SPATA2L	0.01	7.10	0.13	0.89912
POLR3E	-0.01	8.61	-0.13	0.89914
FAM217B	-0.01	8.47	-0.13	0.89921
AC080112.5	0.02	5.20	0.13	0.89932
CCT6P3	0.02	5.99	0.13	0.89939
CLEC18A	-0.02	5.11	-0.13	0.89945
RNASE1	0.03	5.25	0.13	0.89992
CDKN2AIPNL	-0.01	6.99	-0.13	0.89997
CNTNAP1	-0.02	6.45	-0.13	0.90004
RNF41	-0.01	9.25	-0.13	0.90011

HNRNPUL1	0.01	11.94	0.13	0.90025
JUN	0.01	7.72	0.13	0.90043
AC012020.1	0.01	5.98	0.13	0.90049
DLGAP3	-0.01	5.22	-0.13	0.90050
EXO5	-0.01	6.96	-0.13	0.90050
AC090616.6	0.01	5.34	0.13	0.90054
SIGLEC6	0.02	5.20	0.13	0.90061
SLC49A3	-0.01	7.34	-0.13	0.90062
GRHL1	-0.01	5.10	-0.13	0.90063
EFHC1	0.02	6.15	0.13	0.90070
TLR10	0.02	7.38	0.13	0.90086
MAIP1	-0.01	6.36	-0.13	0.90090
YIPF3	0.01	11.11	0.13	0.90095
PRPF38A	-0.01	9.13	-0.12	0.90104
RNF208	-0.02	6.24	-0.12	0.90116
SEC11A	-0.01	10.02	-0.12	0.90119
TENT4B	0.01	7.34	0.12	0.90123
SPTA1	-0.02	5.57	-0.12	0.90129
ZFP90	0.01	8.16	0.12	0.90158
FAM204A	-0.01	7.93	-0.12	0.90161
AL049830.1	0.01	5.24	0.12	0.90169
ST20-AS1	0.02	6.35	0.12	0.90171
CCDC84	-0.02	8.44	-0.12	0.90195
AC006001.2	0.02	5.23	0.12	0.90196
AIG1	0.01	7.10	0.12	0.90197
KLRA1P	-0.02	5.37	-0.12	0.90218
AC007041.1	-0.01	5.07	-0.12	0.90219
EIF4A1P10	-0.02	7.82	-0.12	0.90220
GALNT14	-0.02	6.35	-0.12	0.90229
EIF1P3	0.01	6.70	0.12	0.90247
GALK2	0.01	7.26	0.12	0.90270
SPDYC	-0.03	5.19	-0.12	0.90280
TRAV24	0.02	5.25	0.12	0.90282
RBM12B	-0.01	6.82	-0.12	0.90318
B3GAT1	-0.02	7.64	-0.12	0.90324
IQCH-AS1	-0.01	5.77	-0.12	0.90341
WWC1	-0.01	5.09	-0.12	0.90376
ZNF549	-0.01	6.83	-0.12	0.90387
ZWINT	-0.01	5.61	-0.12	0.90393
CBLN3	0.02	5.81	0.12	0.90398
HIC1	0.01	6.18	0.12	0.90409
TBC1D27P	0.02	6.12	0.12	0.90411
AC073063.1	-0.01	5.93	-0.12	0.90422
MARK3	0.01	10.66	0.12	0.90426
AC004951.2	-0.01	5.44	-0.12	0.90438
SUB1P1	0.01	5.99	0.12	0.90454
PRUNE1	-0.01	8.43	-0.12	0.90483
DPF2	0.01	10.60	0.12	0.90497
CACYBP	-0.01	8.57	-0.12	0.90514
EIF4E2	0.01	9.97	0.12	0.90522

TACC1	-0.01	10.48	-0.12	0.90539
DLX4	-0.01	4.92	-0.12	0.90544
HSDL1	-0.01	7.73	-0.12	0.90559
MYO1F	0.01	14.76	0.12	0.90570
PSMB5	-0.01	8.61	-0.12	0.90576
TRMT44	0.01	7.11	0.12	0.90594
BCAS2	0.01	7.80	0.12	0.90598
AP001189.1	0.03	8.82	0.12	0.90633
MANEAL	-0.01	6.03	-0.12	0.90655
AC087499.4	-0.01	5.11	-0.12	0.90684
AC133552.2	0.01	5.20	0.12	0.90684
AL132780.4	0.02	4.97	0.12	0.90687
ZNF699	-0.01	6.25	-0.12	0.90691
TAF10	-0.01	6.79	-0.12	0.90694
AC020909.2	-0.02	5.23	-0.12	0.90702
SV2A	0.01	6.16	0.12	0.90721
LDHA	-0.01	10.90	-0.12	0.90725
AC007566.1	0.01	5.30	0.12	0.90736
KCNQ5	-0.01	5.18	-0.12	0.90741
KPNA2	-0.01	7.91	-0.12	0.90742
AC108479.1	0.01	5.11	0.12	0.90764
MIATNB	0.01	7.54	0.12	0.90766
ZNF616	-0.01	6.25	-0.12	0.90797
PSMA2P3	-0.01	5.60	-0.12	0.90806
SIK1B	-0.02	5.46	-0.12	0.90822
OXSM	0.01	6.41	0.12	0.90826
ARF3	0.01	10.89	0.12	0.90828
IPO5	0.01	9.38	0.12	0.90829
MBOAT7	-0.02	13.06	-0.12	0.90832
SEPHS2	-0.01	9.76	-0.12	0.90836
RAB19	0.02	5.31	0.12	0.90839
GANAB	-0.01	11.89	-0.12	0.90845
TRAV29DV5	-0.01	6.67	-0.12	0.90848
HSPA8P5	-0.02	6.99	-0.12	0.90875
AC099343.5	0.01	5.13	0.12	0.90877
RHBDD1	0.01	7.87	0.11	0.90907
FSTL3	-0.01	6.43	-0.11	0.90908
EFCAB8	0.02	5.14	0.11	0.90920
GZMA	0.02	10.69	0.11	0.90925
SLC43A3	-0.01	8.56	-0.11	0.90932
ST13P6	-0.01	5.61	-0.11	0.90938
AC021078.1	-0.01	6.09	-0.11	0.90938
GLB1L	-0.02	6.34	-0.11	0.90950
DYNC2LI1	0.01	5.40	0.11	0.90958
TMEM164	0.01	10.51	0.11	0.90962
MT2A	-0.03	9.47	-0.11	0.90965
ZNF512	-0.01	7.76	-0.11	0.90976
DEFA1B	0.04	7.50	0.11	0.90995
UTP14A	-0.01	8.27	-0.11	0.91007
COPG1	-0.01	10.87	-0.11	0.91026

AC083799.1	-0.02	7.10	-0.11	0.91038
UXS1	-0.01	9.07	-0.11	0.91043
HSPB11	-0.01	6.75	-0.11	0.91043
AC092809.4	-0.01	5.51	-0.11	0.91046
ETV2	-0.01	4.95	-0.11	0.91062
DGKK	0.02	5.64	0.11	0.91071
ACAA2	-0.01	9.02	-0.11	0.91077
VWCE	-0.03	8.40	-0.11	0.91077
PPP1R18	-0.01	12.79	-0.11	0.91080
ADGRG5	0.01	8.94	0.11	0.91085
HNRNPA1P10	0.01	7.47	0.11	0.91127
ZNF582-AS1	-0.01	5.24	-0.11	0.91128
ANXA2	0.01	12.32	0.11	0.91128
MT1E	0.02	5.87	0.11	0.91131
TRIM8	-0.01	10.91	-0.11	0.91136
TBC1D24	0.01	6.74	0.11	0.91136
ABHD17C	-0.01	5.79	-0.11	0.91137
AKNA	0.01	12.99	0.11	0.91140
TGM2	-0.02	7.27	-0.11	0.91147
RCAN1	-0.01	7.26	-0.11	0.91151
TXN	0.02	9.82	0.11	0.91166
VPS33A	-0.01	8.00	-0.11	0.91169
FGGY	-0.01	5.98	-0.11	0.91180
MAEA	-0.01	10.55	-0.11	0.91193
PTGR2	-0.01	5.20	-0.11	0.91195
PCYT1B	-0.01	5.57	-0.11	0.91203
FAM219A	-0.01	8.38	-0.11	0.91220
SPIN2B	0.01	5.57	0.11	0.91228
ACSS1	-0.01	10.18	-0.11	0.91231
DHRSX	-0.01	8.07	-0.11	0.91239
CCL4L2	-0.02	6.33	-0.11	0.91250
IGHMBP2	0.01	8.37	0.11	0.91256
AC084125.2	0.01	5.34	0.11	0.91259
CALHM2	-0.01	10.10	-0.11	0.91261
CACNA1F	0.01	5.39	0.11	0.91268
CCNB1	0.01	5.84	0.11	0.91268
NOTCH4	-0.01	6.04	-0.11	0.91269
STK32C	0.01	7.99	0.11	0.91279
ALOX15	-0.03	7.21	-0.11	0.91292
NAF1	-0.01	5.78	-0.11	0.91293
AL162586.1	-0.02	6.16	-0.11	0.91295
BAZ1B	-0.01	10.01	-0.11	0.91304
NPIPB2	-0.01	5.85	-0.11	0.91326
MPP6	0.02	5.96	0.11	0.91333
AC004069.1	0.01	5.76	0.11	0.91340
TEX261	0.01	9.97	0.11	0.91346
GP5	-0.02	5.70	-0.11	0.91351
ERBB2	0.01	8.23	0.11	0.91367
HSD17B12	-0.01	7.24	-0.11	0.91369
SLC39A14	0.01	6.41	0.11	0.91384

HLA-C	0.01	16.41	0.11	0.91394
MINCR	-0.01	5.74	-0.11	0.91399
DNASE1L1	-0.01	9.67	-0.11	0.91400
B9D1	-0.01	5.10	-0.11	0.91401
ZSCAN18	-0.01	8.36	-0.11	0.91425
ATXN7L1	-0.01	7.92	-0.11	0.91429
CACNA1C-AS2	0.01	5.71	0.11	0.91445
ACSBG1	0.02	5.38	0.11	0.91452
TLNRD1	-0.01	8.78	-0.11	0.91472
CYTH4	0.01	13.01	0.11	0.91488
ELOA-AS1	0.01	6.13	0.11	0.91497
HADHAP1	-0.01	5.71	-0.11	0.91498
HSPA1B	0.01	7.91	0.11	0.91499
ZDHHC14	-0.01	6.85	-0.11	0.91504
MOK	-0.01	5.39	-0.11	0.91506
GGACT	0.01	6.07	0.11	0.91510
PIGH	0.01	6.81	0.11	0.91528
PROSER3	0.01	7.44	0.11	0.91534
WDYHV1	-0.01	6.07	-0.11	0.91545
PRMT6	0.01	6.86	0.11	0.91545
LINS1	-0.01	7.47	-0.11	0.91554
AC002524.1	-0.01	5.14	-0.11	0.91561
DSN1	0.01	7.04	0.11	0.91568
LINC00899	-0.01	6.46	-0.11	0.91571
H2AZ1	-0.01	10.84	-0.11	0.91582
ARMCX2	0.01	5.80	0.11	0.91589
HYPK	0.02	5.66	0.11	0.91600
NDUFB4P12	0.01	5.37	0.11	0.91601
TIMM21	-0.01	6.86	-0.11	0.91604
WDTC1	0.01	11.77	0.11	0.91604
IGLV2-8	-0.02	7.58	-0.11	0.91627
SLC2A13	-0.01	5.06	-0.11	0.91629
RASSF1	-0.01	10.67	-0.11	0.91644
AC025171.5	0.01	5.56	0.11	0.91654
C18orf21	-0.01	7.15	-0.11	0.91673
JOSD1	-0.01	9.39	-0.10	0.91699
NUP93	-0.01	9.37	-0.10	0.91714
MKKS	-0.01	7.74	-0.10	0.91732
SPAG5-AS1	0.01	5.07	0.10	0.91758
GSTM2	0.02	7.40	0.10	0.91762
UPP1	-0.01	10.04	-0.10	0.91780
MCUR1	-0.01	7.38	-0.10	0.91784
STAC3	-0.01	8.02	-0.10	0.91791
DCAF17	0.01	6.56	0.10	0.91793
SLCO4A1	-0.02	4.97	-0.10	0.91795
ZNF252P	-0.01	7.23	-0.10	0.91796
SREBF2	0.01	10.35	0.10	0.91813
RPS26	0.04	11.83	0.10	0.91832
DYRK2	0.01	9.09	0.10	0.91835
ASF1B	-0.01	8.33	-0.10	0.91860

WASH7P	-0.01	5.53	-0.10	0.91860
STAT4	-0.01	9.27	-0.10	0.91865
ZNF780B	-0.01	6.73	-0.10	0.91879
GRM2	0.01	4.95	0.10	0.91881
AHDC1	0.01	8.04	0.10	0.91882
OARD1	0.01	8.07	0.10	0.91889
EXOC4	0.01	9.09	0.10	0.91892
MAPK13	-0.01	10.09	-0.10	0.91892
RPF2	-0.01	7.03	-0.10	0.91896
PPP1R2	0.01	9.07	0.10	0.91938
KRT8P12	-0.01	6.65	-0.10	0.91944
BOD1	-0.01	7.44	-0.10	0.91947
RCN3	0.02	9.16	0.10	0.91961
ATG13	0.01	10.81	0.10	0.91983
RAB37	-0.01	11.33	-0.10	0.91987
AGO3	0.01	7.75	0.10	0.91996
DNHD1	0.02	8.09	0.10	0.92027
PUS7L	0.01	6.39	0.10	0.92029
TRAT1	0.01	7.51	0.10	0.92041
MHENCR	-0.01	8.02	-0.10	0.92049
MCPH1-AS1	-0.01	5.01	-0.10	0.92057
TRAF3	-0.01	9.30	-0.10	0.92062
MCPH1	0.01	7.43	0.10	0.92070
FAM157B	0.02	5.52	0.10	0.92084
NADSYN1	0.01	10.85	0.10	0.92091
ZFYVE21	-0.01	7.80	-0.10	0.92110
NLRP6	-0.02	8.45	-0.10	0.92169
ZPR1	-0.01	8.87	-0.10	0.92193
ZNF502	-0.01	5.54	-0.10	0.92203
LEO1	-0.01	7.74	-0.10	0.92251
C15orf39	-0.01	12.03	-0.10	0.92254
UBBP2	0.02	5.55	0.10	0.92261
FHL2	0.01	5.57	0.10	0.92268
ARF1	0.01	12.83	0.10	0.92284
ORM2	0.02	5.20	0.10	0.92341
C22orf39	0.01	8.73	0.10	0.92342
MPRIP	0.01	10.39	0.10	0.92354
AC018638.2	0.01	7.29	0.10	0.92358
KCNK7	-0.01	5.57	-0.10	0.92371
BTF3P7	0.01	5.30	0.10	0.92388
IGHV3-21	0.02	7.34	0.10	0.92401
TRAV25	0.01	5.42	0.10	0.92403
POLR3F	0.01	6.55	0.10	0.92422
SLC9A1	-0.01	10.16	-0.10	0.92435
LY96	-0.02	8.01	-0.10	0.92435
C4orf3	-0.01	10.38	-0.10	0.92437
AGER	-0.01	9.66	-0.10	0.92454
APH1B	-0.01	8.18	-0.10	0.92466
SLC35A2	-0.01	8.31	-0.09	0.92482
COX7B	-0.01	9.08	-0.09	0.92485

AC099343.4	0.01	6.58	0.09	0.92499
CIPC	-0.01	6.77	-0.09	0.92509
SLC25A53	-0.01	6.50	-0.09	0.92517
AMN	-0.01	5.47	-0.09	0.92528
AP000547.3	0.02	7.05	0.09	0.92529
IMPDH1P5	-0.01	5.79	-0.09	0.92531
AL928654.4	0.01	5.04	0.09	0.92551
LINC01871	0.02	7.78	0.09	0.92560
CHST14	-0.01	8.01	-0.09	0.92565
CSE1L	0.01	8.59	0.09	0.92575
PID1	0.02	7.80	0.09	0.92580
ING1	0.01	8.62	0.09	0.92589
FGFR1OP2	-0.01	8.65	-0.09	0.92609
FANCG	0.01	7.26	0.09	0.92612
GON7	-0.01	6.43	-0.09	0.92613
ZNF721	-0.01	7.57	-0.09	0.92643
FOSB	0.01	6.45	0.09	0.92650
ARHGEF12	0.02	8.53	0.09	0.92663
ZNF263	-0.01	8.60	-0.09	0.92679
COX17P1	0.01	5.21	0.09	0.92688
TUBE1	-0.01	6.37	-0.09	0.92689
NMNAT3	-0.01	5.45	-0.09	0.92712
DYNC1I2	0.01	8.84	0.09	0.92726
MDC1	-0.01	8.86	-0.09	0.92746
AC159540.2	0.01	5.12	0.09	0.92750
CCDC114	-0.01	5.39	-0.09	0.92755
CR2	0.01	6.43	0.09	0.92776
S1PR1	-0.01	11.06	-0.09	0.92788
FOXD2	0.01	5.01	0.09	0.92790
NPIPA3	0.01	5.79	0.09	0.92791
NUDT7	-0.01	5.68	-0.09	0.92797
USP13	0.01	6.30	0.09	0.92803
NPC2	0.01	10.87	0.09	0.92805
SARAF	-0.01	12.21	-0.09	0.92809
ABHD17AP5	-0.01	5.06	-0.09	0.92828
PSIP1	-0.01	9.13	-0.09	0.92830
ST3GAL1	0.01	11.12	0.09	0.92847
DNAJA4	-0.01	7.99	-0.09	0.92885
ZNF396	-0.01	5.71	-0.09	0.92899
AC092368.3	0.01	6.37	0.09	0.92907
IGHV1-46	0.02	7.03	0.09	0.92926
AC022506.2	-0.01	5.38	-0.09	0.92935
AC136624.1	-0.01	5.49	-0.09	0.92959
PROCR	0.01	5.80	0.09	0.92962
MXD3	-0.01	8.33	-0.09	0.92964
PPP1R3G	-0.01	5.00	-0.09	0.92966
PRRG4	0.02	7.97	0.09	0.92973
CBFA2T2	0.01	8.71	0.09	0.92974
AC091804.1	-0.01	5.12	-0.09	0.93010
GYPB	-0.02	6.04	-0.09	0.93048

H2AZP2	0.01	5.23	0.09	0.93058
TRAV22	-0.01	5.62	-0.09	0.93092
IRF2BPL	0.01	9.56	0.09	0.93093
DUSP16	-0.01	8.73	-0.09	0.93103
ZNF135	0.01	5.39	0.09	0.93110
SNX20	-0.01	11.07	-0.09	0.93126
RIC8B	-0.01	6.60	-0.09	0.93142
DACT1	-0.02	5.22	-0.09	0.93146
ERICH1	-0.01	8.64	-0.09	0.93179
E2F5	0.01	6.06	0.09	0.93184
RBM19	0.01	8.74	0.09	0.93188
AL137003.1	-0.01	5.30	-0.09	0.93192
CELF1	0.01	10.53	0.09	0.93203
ZNF461	0.01	6.08	0.09	0.93206
C1QA	0.02	7.48	0.09	0.93213
SLC19A1	0.01	10.35	0.09	0.93214
ZNF687	0.01	9.80	0.09	0.93218
NFKB1	0.01	10.46	0.09	0.93247
COX7A2P2	0.01	5.25	0.09	0.93255
E2F2	-0.02	8.30	-0.08	0.93264
AC008875.2	0.01	5.20	0.08	0.93289
CANT1	-0.01	11.02	-0.08	0.93291
PHLDA1	-0.01	5.90	-0.08	0.93291
CFH	0.01	6.07	0.08	0.93296
FBXO32	-0.01	7.71	-0.08	0.93305
PRKX	0.01	9.18	0.08	0.93315
MMP9	-0.02	11.91	-0.08	0.93320
WWOX	-0.01	6.56	-0.08	0.93324
LGALS2	0.02	9.42	0.08	0.93338
REC8	-0.01	9.30	-0.08	0.93342
HSPA8P1	-0.01	6.69	-0.08	0.93348
PPOX	0.01	8.33	0.08	0.93361
AC136475.2	0.01	5.35	0.08	0.93366
IGHV3-23	0.02	9.29	0.08	0.93371
COPS5	0.00	8.68	0.08	0.93388
INPP5F	0.01	6.79	0.08	0.93390
RWDD4P2	-0.01	5.08	-0.08	0.93394
DDX52	0.01	7.69	0.08	0.93398
NCKAP1L	0.00	11.94	-0.08	0.93408
RET	0.01	5.03	0.08	0.93410
H1-2	-0.01	9.39	-0.08	0.93430
FGFBP2	0.02	10.25	0.08	0.93435
MSRB1	-0.01	12.70	-0.08	0.93439
DKC1	0.00	9.25	0.08	0.93447
ITGB1BP1	-0.01	8.63	-0.08	0.93472
BMPR1A	-0.01	5.46	-0.08	0.93483
SAYSD1	0.01	7.63	0.08	0.93490
TBC1D3E	-0.01	6.29	-0.08	0.93492
MPHOSPH10	0.01	8.07	0.08	0.93499
MTCO3P12	0.01	8.23	0.08	0.93509

POC1A	0.01	5.90	0.08	0.93519
MRPL47	0.01	7.16	0.08	0.93521
ACAT1	-0.01	7.91	-0.08	0.93534
SHLD2	-0.01	7.00	-0.08	0.93534
AP1M2	0.01	5.94	0.08	0.93542
SERPINE1	0.01	5.46	0.08	0.93560
ATPAF1	0.01	8.10	0.08	0.93575
AL592148.1	-0.01	5.55	-0.08	0.93599
AC068473.5	-0.01	5.82	-0.08	0.93630
EIF3LP1	0.01	6.04	0.08	0.93640
ZNF329	-0.01	6.99	-0.08	0.93652
EIF3KP3	0.01	5.14	0.08	0.93657
WDR73	0.01	8.25	0.08	0.93661
SEMA3B	0.01	5.22	0.08	0.93662
ATP1A1-AS1	-0.01	5.50	-0.08	0.93664
EHD2	0.01	5.00	0.08	0.93683
RARS2	0.00	7.89	-0.08	0.93684
APBB3	-0.01	8.48	-0.08	0.93687
FAM117B	0.01	7.96	0.08	0.93688
AATK	0.01	9.64	0.08	0.93690
KCNK13	0.01	5.37	0.08	0.93695
DIS3L	-0.01	8.29	-0.08	0.93697
C7orf61	0.01	5.36	0.08	0.93713
GPS2P1	-0.01	6.64	-0.08	0.93746
ADORA3	-0.01	5.85	-0.08	0.93746
PLBD2	-0.01	9.02	-0.08	0.93753
AL591895.1	0.01	7.12	0.08	0.93758
MLLT1	0.01	10.45	0.08	0.93770
CHD2	-0.01	11.01	-0.08	0.93774
RAB2B	-0.01	7.83	-0.08	0.93777
BX322234.1	0.01	5.44	0.08	0.93790
C21orf58	0.01	6.30	0.08	0.93795
PPP2R5E	-0.01	8.17	-0.08	0.93807
MMGT1	0.01	8.43	0.08	0.93809
CLK2P1	-0.01	5.53	-0.08	0.93813
AC064805.1	0.01	7.37	0.08	0.93815
BANK1	-0.01	8.05	-0.08	0.93836
MYO9B	-0.01	12.65	-0.08	0.93841
ARHGEF9	0.01	7.95	0.08	0.93859
TMEM106C	0.01	7.59	0.08	0.93879
AL031729.1	0.01	6.83	0.08	0.93895
RIMS3	-0.01	6.27	-0.08	0.93921
RER1	0.00	10.67	-0.08	0.93923
WDR27	-0.01	6.48	-0.08	0.93930
AC004801.1	0.01	5.25	0.08	0.93946
SGPP1	-0.01	6.75	-0.08	0.93977
TRAV19	0.01	5.96	0.08	0.93977
PIP4K2A	0.01	11.38	0.08	0.93989
KMT2A	-0.01	9.70	-0.08	0.93996
GOLGA2P5	0.01	6.88	0.08	0.94024

TMEM50B	-0.01	8.63	-0.08	0.94024
EARS2	-0.01	7.32	-0.08	0.94044
PRSS21	0.02	5.67	0.07	0.94055
PROS1	0.01	5.39	0.07	0.94076
ZNF835	-0.01	5.44	-0.07	0.94085
CEP68	0.01	7.63	0.07	0.94089
LRP5	-0.01	5.91	-0.07	0.94093
AC015912.3	0.01	5.58	0.07	0.94107
ITGA10	-0.01	5.41	-0.07	0.94108
GLIS2	0.01	5.11	0.07	0.94110
TCF19	-0.01	7.47	-0.07	0.94121
PABPC1P1	0.01	5.57	0.07	0.94130
C1orf43	0.00	10.44	0.07	0.94139
SNHG5	-0.01	9.75	-0.07	0.94143
FABP5	-0.01	5.57	-0.07	0.94161
TMEM183A	0.00	8.71	0.07	0.94172
PTPN2P1	0.01	5.13	0.07	0.94183
TOP1	-0.01	10.31	-0.07	0.94187
TOP3B	-0.01	8.73	-0.07	0.94233
LRG1	0.02	10.86	0.07	0.94235
GNAS	0.01	13.91	0.07	0.94242
MT-ND6	-0.01	8.09	-0.07	0.94244
PKMP5	0.01	5.84	0.07	0.94272
FASLG	-0.01	7.04	-0.07	0.94272
SPI1	-0.01	13.47	-0.07	0.94294
OLA1P1	-0.01	5.39	-0.07	0.94297
TRGV8	0.01	5.05	0.07	0.94303
RBMS1P1	0.01	5.45	0.07	0.94306
CDHR3	0.01	5.46	0.07	0.94319
AL513165.1	-0.01	5.67	-0.07	0.94327
ST3GAL5	-0.01	8.54	-0.07	0.94334
DDX11L10	0.03	6.82	0.07	0.94355
SDCBP2	0.01	5.59	0.07	0.94360
PARS2	-0.01	6.53	-0.07	0.94377
APOLD1	-0.01	5.56	-0.07	0.94392
DBN1	-0.01	8.34	-0.07	0.94409
RUNX1	0.00	9.46	0.07	0.94426
TRIM62	0.00	8.31	-0.07	0.94430
NUTM2A-AS1	-0.01	7.77	-0.07	0.94439
KIAA1324	0.02	9.69	0.07	0.94445
MT-ND4	-0.01	15.48	-0.07	0.94451
LRCH4	-0.01	9.98	-0.07	0.94466
AC002310.1	0.01	5.28	0.07	0.94473
LINC00174	-0.01	7.17	-0.07	0.94485
AL450063.1	0.01	6.26	0.07	0.94488
LINC02610	-0.01	5.23	-0.07	0.94499
TDRD9	0.01	6.14	0.07	0.94499
STK10	0.00	13.32	-0.07	0.94510
C19orf12	0.01	8.28	0.07	0.94512
PDIA4	0.01	10.09	0.07	0.94532

ARHGAP33	-0.01	6.96	-0.07	0.94550
TMA16	0.01	6.78	0.07	0.94560
PLA2G6	-0.01	8.99	-0.07	0.94561
IGKV1-33	-0.01	5.58	-0.07	0.94561
DNAJC21	0.00	8.14	-0.07	0.94566
ERMARD	0.01	7.64	0.07	0.94568
EIF2S2P4	-0.01	6.51	-0.07	0.94588
DEFA1	0.02	7.51	0.07	0.94589
C18orf54	0.01	5.02	0.07	0.94605
AKIRIN1	0.00	9.82	-0.07	0.94612
BEGAIN	-0.01	5.89	-0.07	0.94618
JRK	0.00	8.34	-0.07	0.94665
TRIM46	0.01	6.58	0.07	0.94682
STXBP1	0.01	5.90	0.07	0.94686
AC006449.7	0.01	6.73	0.07	0.94697
INTS10	0.00	9.10	-0.07	0.94705
AC005332.3	-0.01	5.08	-0.07	0.94720
PPP1R16B	0.01	10.10	0.07	0.94758
WDR77	0.00	8.09	-0.07	0.94764
XPO5	0.00	8.44	-0.07	0.94765
B3GNT8	-0.01	8.57	-0.07	0.94768
RAB35	0.00	10.75	0.07	0.94786
CPT1A	-0.01	10.66	-0.07	0.94786
ADAM8	0.01	13.02	0.07	0.94787
LUCAT1	-0.01	9.81	-0.07	0.94788
FLOT2	0.01	13.36	0.07	0.94824
IAH1	0.00	8.15	-0.07	0.94840
PLOD1	-0.01	10.40	-0.06	0.94853
AC121761.1	-0.01	5.44	-0.06	0.94868
ZNF534	-0.01	5.10	-0.06	0.94935
CCDC24	-0.01	5.99	-0.06	0.94937
TNFRSF17	0.01	6.03	0.06	0.94937
MAP3K9	0.01	6.40	0.06	0.94963
FCGBP	-0.01	8.24	-0.06	0.94964
IL15RA	0.01	7.79	0.06	0.94968
ZNF850	-0.01	5.12	-0.06	0.94968
RAB43P1	0.01	5.15	0.06	0.94976
RAB4B	-0.01	6.00	-0.06	0.94978
AC002467.1	0.01	6.19	0.06	0.94990
NECAB2	0.01	7.53	0.06	0.94990
CADM1	-0.01	5.50	-0.06	0.95015
TRAV17	0.01	6.35	0.06	0.95023
CPSF2	0.00	8.52	-0.06	0.95029
LRIG2	0.00	7.78	0.06	0.95030
EGF	-0.01	5.84	-0.06	0.95043
GDI2	0.00	11.23	-0.06	0.95056
EIF2B1	0.00	9.36	0.06	0.95068
GADD45B	0.01	9.73	0.06	0.95069
TOP3A	0.00	9.79	0.06	0.95070
MEF2D	0.00	10.55	-0.06	0.95072

ZNF630	-0.01	5.45	-0.06	0.95077
ATP6AP1L	-0.01	5.16	-0.06	0.95081
GEMIN5	0.00	7.94	0.06	0.95084
AAGAB	0.00	8.64	-0.06	0.95093
AC110998.1	0.01	5.28	0.06	0.95096
CHP1	0.00	9.83	-0.06	0.95102
PRMT5	0.00	8.93	-0.06	0.95113
TRA2B	0.00	9.95	-0.06	0.95120
PLPPR2	-0.01	10.65	-0.06	0.95122
HLA-DQA1	-0.01	10.93	-0.06	0.95130
DDIT3	0.01	8.75	0.06	0.95134
KATNAL1	-0.01	5.81	-0.06	0.95140
IGHV3-53	0.01	6.67	0.06	0.95144
TRIM16	0.01	5.84	0.06	0.95148
CBX3	0.00	9.38	0.06	0.95150
LYG1	0.01	5.30	0.06	0.95167
TMEM159	0.00	7.33	-0.06	0.95176
PATJ	0.01	6.95	0.06	0.95177
TCTA	0.00	8.98	0.06	0.95183
QPRT	-0.01	6.22	-0.06	0.95188
C17orf49	0.01	5.75	0.06	0.95196
ALPK3	0.01	5.15	0.06	0.95197
LINC00944	-0.01	5.76	-0.06	0.95217
STRADA	-0.01	7.57	-0.06	0.95219
ALDH4A1	-0.01	6.89	-0.06	0.95224
SDC4	0.01	5.81	0.06	0.95249
IGHGP	0.02	7.74	0.06	0.95295
THNSL1	0.01	5.96	0.06	0.95298
AC079944.2	0.01	7.34	0.06	0.95300
CD300H	0.01	7.48	0.06	0.95309
CLK2	0.00	9.95	0.06	0.95348
AC008105.3	0.01	9.89	0.06	0.95349
LGALS9	0.01	11.21	0.06	0.95355
IGFBP6	0.01	5.16	0.06	0.95379
NIPAL2	-0.01	6.84	-0.06	0.95449
AL512504.1	-0.01	5.15	-0.06	0.95456
CAMLG	0.00	8.77	0.06	0.95459
CHPF2	0.00	10.41	-0.06	0.95460
DDX11L9	-0.01	5.84	-0.06	0.95471
PYHIN1	0.01	9.04	0.06	0.95486
CNOT11	0.00	8.61	-0.06	0.95496
PCGF5	-0.01	10.66	-0.06	0.95508
PYGM	-0.01	7.42	-0.06	0.95519
CDC42BPB	-0.01	8.39	-0.06	0.95527
ZNF599	0.01	5.72	0.06	0.95534
ACCS	-0.01	8.09	-0.06	0.95540
ST3GAL2	-0.01	10.50	-0.06	0.95553
CELSR3	-0.01	6.54	-0.06	0.95559
YIPF5	0.00	8.45	0.06	0.95562
ZBTB46	0.01	5.96	0.06	0.95570

LSM8	0.00	8.19	-0.06	0.95573
AL109918.2	0.01	5.14	0.06	0.95577
TNFRSF14-AS1	-0.01	7.58	-0.05	0.95639
TMEM86A	-0.01	5.84	-0.05	0.95642
SERF1A	-0.01	4.92	-0.05	0.95643
DDHD2	-0.01	7.52	-0.05	0.95645
MTCO1P40	-0.01	7.05	-0.05	0.95662
NAE1	0.00	7.85	-0.05	0.95676
MPZ	-0.01	6.93	-0.05	0.95678
AL021707.6	-0.01	6.65	-0.05	0.95696
AL354702.1	-0.01	5.58	-0.05	0.95709
ZNF8	0.01	6.91	0.05	0.95720
RNF31	0.01	5.63	0.05	0.95723
GZF1	0.00	6.86	0.05	0.95738
DPH7	0.00	8.57	0.05	0.95744
PRPS1	0.00	8.80	-0.05	0.95751
TMEM119	-0.01	5.04	-0.05	0.95768
WHAMMP2	0.01	6.51	0.05	0.95770
ITGB3BP	0.00	5.96	0.05	0.95788
ZNF85	0.00	5.66	-0.05	0.95880
SGPP2	-0.01	6.02	-0.05	0.95885
ZMYND8	0.00	9.39	0.05	0.95886
AC107959.1	0.00	7.17	-0.05	0.95895
AC009303.4	-0.01	4.99	-0.05	0.95903
ZNF383	0.00	6.81	0.05	0.95908
BRIX1	0.00	7.29	-0.05	0.95937
PSMG3-AS1	-0.01	7.15	-0.05	0.95937
TMEM41B	0.00	7.39	-0.05	0.95945
SELENOK	0.00	8.71	-0.05	0.95978
ABTB1	0.01	13.34	0.05	0.95989
TCTN3	0.00	8.05	-0.05	0.95998
SLITRK4	0.01	5.65	0.05	0.96009
PTMAP4	0.00	6.14	0.05	0.96037
ADGRB2	-0.01	5.59	-0.05	0.96048
WBP11	0.00	9.91	-0.05	0.96049
PLXDC1	0.01	7.13	0.05	0.96050
ASGR2	0.01	8.77	0.05	0.96056
CYB561D1	0.00	9.24	0.05	0.96057
AC090948.3	-0.01	5.29	-0.05	0.96066
B3GNT9	0.00	6.49	-0.05	0.96069
RRM2	0.01	6.17	0.05	0.96077
MTAP	0.00	6.83	0.05	0.96085
GMNN	0.00	5.77	0.05	0.96086
SAP30L-AS1	0.01	5.40	0.05	0.96095
TAF11	0.00	7.94	0.05	0.96119
AC004812.2	-0.01	5.36	-0.05	0.96123
FARP2	-0.01	7.59	-0.05	0.96147
FAM229A	-0.01	7.67	-0.05	0.96155
FTH1P23	-0.01	7.33	-0.05	0.96158
MSH3	0.00	6.99	-0.05	0.96180

HSP90B3P	0.00	6.41	-0.05	0.96189
BPHL	-0.01	6.51	-0.05	0.96191
ZDHHC9	0.00	6.20	-0.05	0.96195
PEX7	0.00	5.59	0.05	0.96199
SMIM11A	0.00	5.02	-0.05	0.96226
ZNF563	0.00	5.61	0.05	0.96232
BX640514.2	-0.01	4.96	-0.05	0.96263
SET	0.00	10.74	0.05	0.96266
STAG3L2	-0.01	5.95	-0.05	0.96273
H2AC19	0.01	5.48	0.05	0.96295
HMGXB3	0.00	8.75	-0.05	0.96305
GFRA2	0.01	6.02	0.05	0.96312
REXO2	-0.01	6.92	-0.05	0.96322
ZNF582	0.00	5.67	0.05	0.96326
MYZAP	-0.01	5.37	-0.05	0.96369
PEX11G	0.01	5.01	0.05	0.96387
AC087672.2	0.01	5.19	0.05	0.96391
SOGA1	0.01	7.83	0.05	0.96393
YBX1P2	0.01	7.35	0.05	0.96417
EXD3	0.00	6.85	-0.05	0.96418
TRAV2	0.01	5.94	0.04	0.96460
REEP4	0.00	9.25	0.04	0.96472
CIR1P1	-0.01	5.64	-0.04	0.96473
ZNF626	0.00	6.07	-0.04	0.96481
ATP6V0B	-0.01	12.08	-0.04	0.96483
HMGN2P6	0.00	5.47	-0.04	0.96506
SPATS2	0.00	6.40	-0.04	0.96518
OAF	-0.01	8.45	-0.04	0.96519
OTULIN	0.00	8.19	-0.04	0.96525
FSCN1	0.00	6.78	-0.04	0.96525
LIN54	0.00	6.86	0.04	0.96546
TM2D2	0.00	8.03	-0.04	0.96547
AC114490.3	-0.01	5.23	-0.04	0.96549
S100A9	0.01	16.54	0.04	0.96552
UNC13B	-0.01	5.24	-0.04	0.96575
CUEDC1	0.01	8.47	0.04	0.96589
ASRGL1	0.01	7.52	0.04	0.96591
NME6	0.00	7.93	0.04	0.96607
DNAI2	-0.01	5.24	-0.04	0.96611
PSMB8	0.00	11.71	0.04	0.96614
AC073857.1	0.00	5.57	-0.04	0.96617
ANKRD36BP2	0.01	5.20	0.04	0.96624
SLC9A3R2	0.01	6.16	0.04	0.96625
ARL3	0.00	7.44	0.04	0.96633
AL135999.1	-0.01	7.54	-0.04	0.96645
TSEN34	-0.01	10.06	-0.04	0.96648
ARSG	0.00	8.00	-0.04	0.96650
PABPC1	0.00	14.17	-0.04	0.96650
LINC00888	0.00	5.23	-0.04	0.96652
ARID5B	0.00	7.80	0.04	0.96677

PSMA4	0.00	9.82	0.04	0.96682
TOM1L2	0.00	9.21	0.04	0.96688
LINC01422	0.00	5.51	0.04	0.96697
PLPP5	-0.01	7.84	-0.04	0.96698
BET1	0.00	6.95	0.04	0.96700
LBHD1	0.00	6.11	-0.04	0.96713
SUB1P3	0.00	5.45	0.04	0.96715
AC022916.2	-0.01	6.64	-0.04	0.96717
AL354989.2	0.00	5.58	-0.04	0.96720
STN1	0.00	9.04	-0.04	0.96725
ARHGAP35	0.00	9.43	-0.04	0.96727
AL359918.2	0.01	6.52	0.04	0.96727
CWF19L1	0.00	8.79	-0.04	0.96730
PHKA2	0.00	9.90	0.04	0.96734
CHI3L1	0.01	11.67	0.04	0.96736
TCP1P1	0.00	5.50	0.04	0.96739
RHEB	0.00	7.97	-0.04	0.96752
KAZN	0.01	6.66	0.04	0.96754
RBP7	-0.01	9.42	-0.04	0.96759
H2AZ2	0.00	9.95	0.04	0.96760
PLEKHM1	0.00	10.42	0.04	0.96774
OGFRP1	0.00	5.87	-0.04	0.96792
COQ9	0.00	8.44	-0.04	0.96809
SLC1A7	-0.01	6.25	-0.04	0.96812
NEPRO	0.00	8.17	0.04	0.96820
ICMT	0.00	9.01	0.04	0.96826
IGKV1D-33	-0.01	5.64	-0.04	0.96829
HTT	0.00	10.73	0.04	0.96840
AC018644.1	0.01	5.47	0.04	0.96848
GDPD3	-0.01	8.04	-0.04	0.96849
ARL4A	0.01	6.46	0.04	0.96854
ARHGAP9	0.00	12.93	-0.04	0.96855
RPL5P4	0.00	6.56	0.04	0.96863
NME8	0.01	6.77	0.04	0.96864
WRAP73	0.00	9.21	-0.04	0.96879
TRBV11-3	-0.01	5.18	-0.04	0.96881
TUFMP1	0.00	6.05	-0.04	0.96906
NIF3L1	0.00	7.38	-0.04	0.96914
BCS1L	0.00	7.83	-0.04	0.96940
TRBV24-1	-0.01	5.72	-0.04	0.96951
LINC02785	0.00	5.32	0.04	0.96996
CCNF	0.00	6.57	-0.04	0.96999
AC010175.1	0.01	5.22	0.04	0.97021
EPHA1-AS1	0.01	5.44	0.04	0.97029
TPM1	0.00	8.15	0.04	0.97046
DENND1B	0.00	6.45	-0.04	0.97057
HEBP1	0.00	8.55	0.04	0.97062
GUCA1B	0.00	6.28	0.04	0.97066
AC046176.1	0.00	5.96	0.04	0.97071
SRC	0.00	8.98	-0.04	0.97089

UBXN7	0.00	9.12	-0.04	0.97090
ZNF691	0.00	7.80	-0.04	0.97094
MZF1-AS1	0.00	5.23	0.04	0.97095
PITRM1	0.00	9.52	0.04	0.97102
CDC123	0.00	9.39	-0.04	0.97105
PARVG	0.00	12.07	-0.04	0.97110
EID1	0.00	9.22	0.04	0.97117
EDEM2	0.00	9.80	-0.04	0.97128
PMS2CL	0.00	6.68	0.04	0.97130
FURIN	0.00	11.64	-0.04	0.97139
ACTL10	0.00	5.78	-0.04	0.97163
NEURL4	0.00	8.75	0.04	0.97179
C19orf71	-0.01	5.88	-0.04	0.97194
SLC16A5	0.00	9.21	-0.04	0.97196
C2orf76	0.00	5.91	0.04	0.97206
TRBV2	0.01	6.81	0.04	0.97211
AL359644.1	0.00	5.40	-0.04	0.97217
RAB27A	0.00	10.28	0.03	0.97241
MAX	0.00	11.20	-0.03	0.97253
MAPKBP1	0.00	8.38	-0.03	0.97275
GADD45G	0.00	5.39	0.03	0.97275
AC138932.2	0.00	5.25	0.03	0.97280
LINC02762	0.00	5.73	0.03	0.97284
FNDC11	0.00	5.23	0.03	0.97302
NAP1L4P3	0.00	6.25	0.03	0.97303
AP005018.2	0.00	5.28	0.03	0.97325
DTD2	0.00	5.78	0.03	0.97326
ZNF692	0.00	9.16	0.03	0.97339
RGS9	-0.01	7.23	-0.03	0.97385
FANK1	0.00	4.95	-0.03	0.97386
SH3PXD2A	0.00	7.22	-0.03	0.97399
PPP3CB	0.00	9.00	-0.03	0.97410
LINC01004	0.00	5.27	-0.03	0.97423
LINC00861	0.00	9.92	0.03	0.97426
SEC16A	0.00	11.05	0.03	0.97428
HNRNPA1P35	0.00	5.80	-0.03	0.97446
CDC42EP1	0.00	6.92	0.03	0.97459
EIF5A2	0.00	5.73	0.03	0.97468
XCL2	0.01	6.91	0.03	0.97495
CPT2	0.00	8.24	0.03	0.97496
FOCAD	0.00	7.91	-0.03	0.97513
SSH3	0.00	9.00	-0.03	0.97526
HNRNPA3P5	0.00	5.75	-0.03	0.97527
MAD2L1	0.00	5.54	0.03	0.97544
H3P1	0.00	5.17	-0.03	0.97553
ZXDB	0.00	6.61	-0.03	0.97555
RALGDS	0.00	8.26	0.03	0.97581
AC004687.1	0.01	10.25	0.03	0.97597
SUMO2P1	0.00	5.36	0.03	0.97623
ZNF43	0.00	6.88	0.03	0.97648

SH2D3C	0.00	11.63	0.03	0.97663
CENPE	0.00	5.20	-0.03	0.97666
TBXAS1	0.00	11.25	0.03	0.97681
PTGDS	-0.01	7.15	-0.03	0.97714
AIF1	0.00	12.44	0.03	0.97728
NFU1	0.00	6.96	0.03	0.97735
ZNF347	0.00	6.03	0.03	0.97755
WDR86-AS1	0.00	6.47	0.03	0.97761
DEFA3	-0.01	9.31	-0.03	0.97777
MT-TW	0.00	6.42	0.03	0.97784
TRAF5	0.00	8.35	-0.03	0.97789
MFAP3L	0.00	6.76	-0.03	0.97799
KRT8	0.00	5.00	-0.03	0.97804
METTL21A	0.00	7.39	-0.03	0.97843
METTL18	0.00	6.71	0.03	0.97849
TRAV8-3	0.00	6.55	-0.03	0.97855
DBF4	0.00	6.28	-0.03	0.97862
DAG1	0.00	8.12	0.03	0.97870
ALG8	0.00	7.52	0.03	0.97884
SLC5A5	0.00	5.15	-0.03	0.97888
IATPR	0.00	5.10	0.03	0.97903
ZNF875	0.00	8.40	-0.03	0.97919
IGHV1-3	0.01	6.64	0.03	0.97940
HELB	0.00	8.09	-0.03	0.97944
MCEE	0.00	5.97	-0.03	0.97949
SCML4	0.00	9.24	-0.03	0.97957
UBXN4	0.00	9.43	-0.03	0.97966
SNRPA1	0.00	8.02	0.03	0.97977
PLEKHO2	0.00	12.22	-0.03	0.97989
ACTG1P10	0.00	5.19	-0.03	0.97990
ZC3H8	0.00	6.82	-0.02	0.98044
FLNB	0.00	9.21	0.02	0.98048
CHD9	0.00	7.53	0.02	0.98052
JAG2	0.00	5.16	-0.02	0.98055
DGKH	0.00	5.83	0.02	0.98061
SAMD4B	0.00	9.51	0.02	0.98066
MRPS6	0.00	8.12	-0.02	0.98099
MTRF1L	0.00	6.74	0.02	0.98104
VIPR1	0.00	8.90	-0.02	0.98115
RBBP4P1	0.00	5.24	-0.02	0.98118
MARS2	0.00	6.34	0.02	0.98121
NAP1L1P3	0.00	5.94	0.02	0.98124
ERC1	0.00	8.26	-0.02	0.98153
DEFA4	-0.01	7.47	-0.02	0.98157
IGLV6-57	0.01	6.97	0.02	0.98164
ADARB2	0.01	5.27	0.02	0.98180
CHAD	0.00	5.23	-0.02	0.98186
ZCCHC9	0.00	7.39	-0.02	0.98195
PVALB	0.00	5.59	-0.02	0.98203
UNK	0.00	8.69	0.02	0.98212

AP001889.1	0.00	5.67	-0.02	0.98214
PHYKPL	0.00	10.25	-0.02	0.98215
CALD1	0.00	5.27	0.02	0.98228
CNDP2	0.00	10.99	0.02	0.98231
PCDH9	0.00	5.47	-0.02	0.98247
COPS7B	0.00	9.11	-0.02	0.98250
H4C14	0.00	5.13	0.02	0.98275
ATP2A1	0.00	5.53	-0.02	0.98276
LTBR	0.00	10.44	-0.02	0.98292
PNISR	0.00	10.32	-0.02	0.98297
OSTC	0.00	7.96	-0.02	0.98310
CEP295	0.00	6.92	0.02	0.98324
RXYLT1	0.00	6.65	0.02	0.98328
NDFIP1	0.00	9.71	-0.02	0.98340
NCOA5	0.00	9.62	-0.02	0.98352
BNIP3L	0.00	10.99	0.02	0.98354
SLC26A6	0.00	8.57	-0.02	0.98366
HAR1A	0.00	5.90	-0.02	0.98381
AC108073.1	0.00	5.86	-0.02	0.98399
RPL15P18	0.00	5.49	-0.02	0.98400
LINC02019	0.00	5.76	0.02	0.98409
MBNL3	0.00	9.78	0.02	0.98412
TMEM44-AS1	0.00	5.91	-0.02	0.98417
EIF1AD	0.00	8.41	0.02	0.98420
C19orf54	0.00	7.76	-0.02	0.98447
LENG8-AS1	0.00	5.84	0.02	0.98455
MRPS30	0.00	7.97	0.02	0.98473
RPS15AP38	0.00	6.06	-0.02	0.98473
ANKRD23	0.00	5.66	0.02	0.98474
WDFY4	0.00	10.66	0.02	0.98483
CD3G	0.00	9.06	-0.02	0.98497
POLG2	0.00	7.02	0.02	0.98501
PSMC3IP	0.00	5.80	0.02	0.98524
CSNK2A3	0.00	5.15	-0.02	0.98531
IGHV4-39	0.00	8.14	0.02	0.98561
LAMA5	0.00	6.17	-0.02	0.98561
AL096870.10	0.00	5.87	-0.02	0.98572
FAM76A	0.00	7.00	-0.02	0.98573
C13orf46	0.00	5.26	0.02	0.98575
ATP5F1AP3	0.00	6.27	0.02	0.98577
PDSS2	0.00	6.84	0.02	0.98585
FAM89A	0.00	5.44	-0.02	0.98599
AC022182.2	0.00	6.09	-0.02	0.98603
SLC12A9	0.00	11.48	0.02	0.98613
UEVLD	0.00	6.88	-0.02	0.98613
RNASE3	0.00	6.63	-0.02	0.98622
EPHB3	0.00	6.14	0.02	0.98634
SLC25A5P2	0.00	5.22	-0.02	0.98660
ZNF248	0.00	6.52	0.02	0.98671
TAGLN	0.00	8.53	-0.02	0.98680

CCDC71L	0.00	8.25	0.02	0.98701
ERV3-1	0.00	7.17	0.02	0.98711
H2BC12	0.00	8.47	-0.02	0.98716
SHISA5	0.00	12.51	-0.02	0.98718
SLC2A5	0.00	6.23	-0.02	0.98723
DKK3	0.00	5.15	-0.02	0.98758
USP39	0.00	9.64	-0.02	0.98760
PTP4A2P1	0.00	5.95	-0.02	0.98762
PCBP1	0.00	13.22	-0.02	0.98765
LARP1B	0.00	6.74	-0.02	0.98768
GTF3C1	0.00	10.45	0.02	0.98775
UBL5P2	0.00	6.38	-0.02	0.98776
SRP14	0.00	11.07	-0.02	0.98777
F11R	0.00	9.43	0.02	0.98790
SMIM10L2A	0.00	5.10	0.02	0.98797
PPIEL	0.00	7.39	0.01	0.98814
PDCD2	0.00	8.74	0.01	0.98818
SNHG4	0.00	5.17	0.01	0.98827
HLA-E	0.00	16.20	0.01	0.98836
PDCD4	0.00	10.19	-0.01	0.98863
C20orf197	0.00	5.48	0.01	0.98863
SRP14P2	0.00	5.73	0.01	0.98864
BCCIP	0.00	8.24	0.01	0.98874
PEA15	0.00	9.91	-0.01	0.98877
SEPSECS-AS1	0.00	5.45	0.01	0.98884
GPER1	0.00	5.93	-0.01	0.98899
GCN1	0.00	10.63	-0.01	0.98909
SLC18A2	0.00	5.04	0.01	0.98929
FES	0.00	11.39	-0.01	0.98932
SPAG16	0.00	5.38	0.01	0.98980
MRPS28	0.00	5.63	0.01	0.98983
FUS	0.00	11.71	-0.01	0.98996
LRRFIP1P1	0.00	5.23	0.01	0.98998
HAUS1	0.00	7.02	-0.01	0.98998
AP000757.1	0.00	5.18	-0.01	0.99010
AC125494.2	0.00	5.46	-0.01	0.99013
AC009133.1	0.00	5.91	0.01	0.99017
ASPHD2	0.00	7.50	-0.01	0.99022
RBM28	0.00	8.43	-0.01	0.99027
ZNF14	0.00	7.47	-0.01	0.99030
DCUN1D2	0.00	7.47	-0.01	0.99031
NCF4	0.00	12.87	0.01	0.99043
TRGC2	0.00	8.90	0.01	0.99064
ITGB2	0.00	15.16	0.01	0.99074
RASA4DP	0.00	5.16	0.01	0.99080
IZUMO4	0.00	6.49	0.01	0.99100
KLC1	0.00	8.77	-0.01	0.99104
TIMM23	0.00	8.51	-0.01	0.99106
ENDOD1	0.00	8.09	0.01	0.99114
AC092017.1	0.00	5.25	0.01	0.99117

PGLYRP1	0.00	9.72	0.01	0.99127
NFATC2	0.00	9.69	-0.01	0.99159
AL590617.2	0.00	5.52	-0.01	0.99172
H1FX-AS1	0.00	5.34	0.01	0.99189
AL136295.7	0.00	6.19	0.01	0.99220
KLHL7	0.00	6.86	0.01	0.99223
RFT1	0.00	7.66	-0.01	0.99225
TMEM30B	0.00	5.23	-0.01	0.99226
EPB41L5	0.00	5.49	0.01	0.99237
RTN4IP1	0.00	6.15	0.01	0.99259
AL353622.1	0.00	5.70	-0.01	0.99269
CAPN10-DT	0.00	5.73	-0.01	0.99281
MYH3	0.00	6.67	-0.01	0.99287
ZHX2	0.00	9.48	0.01	0.99294
ZNF845	0.00	7.20	0.01	0.99300
LINC01569	0.00	5.98	-0.01	0.99303
TXNP6	0.00	5.92	-0.01	0.99314
MALSU1	0.00	7.40	-0.01	0.99327
REPS1	0.00	8.56	0.01	0.99334
AC005839.1	0.00	5.37	-0.01	0.99345
AL391832.3	0.00	5.05	0.01	0.99345
APPBP2	0.00	7.76	-0.01	0.99353
AKAP9	0.00	9.24	0.01	0.99358
AC132812.1	0.00	5.85	-0.01	0.99393
GAPDHP70	0.00	5.38	-0.01	0.99394
GPRC5D-AS1	0.00	4.97	0.01	0.99396
BMF	0.00	9.63	0.01	0.99406
RBM27	0.00	7.05	0.01	0.99436
SRI	0.00	9.56	0.01	0.99447
NEXN	0.00	7.15	0.01	0.99474
IGKV1-16	0.00	6.22	0.01	0.99474
TBX21	0.00	10.24	-0.01	0.99476
SOCS7	0.00	7.74	0.01	0.99499
STAP1	0.00	6.49	-0.01	0.99505
APOBEC3H	0.00	6.53	0.01	0.99507
HLA-DPA1	0.00	13.09	0.01	0.99518
CRAMP1	0.00	7.71	-0.01	0.99519
GZMH	0.00	10.90	0.01	0.99533
ZNRD2-AS1	0.00	5.44	0.01	0.99549
OPN3	0.00	7.19	0.01	0.99564
EEF1A1P13	0.00	8.84	-0.01	0.99568
RSKR	0.00	6.90	0.01	0.99571
TDRD3	0.00	7.38	0.01	0.99573
EEF1E1	0.00	5.41	0.01	0.99578
LAMP1	0.00	12.27	0.01	0.99588
HNRNPA1L2	0.00	7.31	-0.01	0.99591
ZNF587B	0.00	6.87	0.01	0.99597
ZNF275	0.00	7.88	0.00	0.99605
LINC00623	0.00	8.34	0.00	0.99605
SOX13	0.00	7.53	0.00	0.99624

ZNF543	0.00	5.43	0.00	0.99678
ANPEP	0.00	12.77	0.00	0.99686
FTH1P7	0.00	8.86	0.00	0.99693
TRDJ1	0.00	5.02	0.00	0.99700
EFHD2	0.00	13.37	0.00	0.99707
ZNF276	0.00	10.80	0.00	0.99717
NUP35	0.00	5.68	0.00	0.99727
PADI4	0.00	11.82	0.00	0.99729
PIPOX	0.00	5.04	0.00	0.99747
EXOG	0.00	6.41	0.00	0.99755
PNPO	0.00	6.68	0.00	0.99769
DNAJC19	0.00	6.98	0.00	0.99772
PIK3R6	0.00	8.25	0.00	0.99792
LINC01943	0.00	5.20	0.00	0.99802
ACOT11	0.00	5.63	0.00	0.99812
LILRB4	0.00	9.24	0.00	0.99812
RPL5P29	0.00	6.28	0.00	0.99842
HLA-DMB	0.00	10.68	0.00	0.99856
RMC1	0.00	8.78	0.00	0.99861
DLG3	0.00	7.48	0.00	0.99869
NDFIP2	0.00	5.15	0.00	0.99884
SBNO2	0.00	11.64	0.00	0.99902
GPBAR1	0.00	9.10	0.00	0.99919
VSIR	0.00	14.12	0.00	0.99919
CST3	0.00	12.77	0.00	0.99926
RGS3	0.00	10.12	0.00	0.99929
NUDT15	0.00	5.73	0.00	0.99937
ZNF419	0.00	7.02	0.00	0.99939
DPH6	0.00	5.62	0.00	0.99939
STAG3L4	0.00	5.94	0.00	0.99980
MT-TS1	0.00	5.39	0.00	0.99985
FTH1P16	0.00	6.55	0.00	0.99985
MTX2	0.00	6.13	0.00	0.99996

nd noCAD

adj.P.Val
0.0849
0.0849
0.0849
0.0849
0.0849
0.0849
0.0849
0.0849
0.0849
0.0849
0.0849
0.0849
0.0849
0.0849
0.0849
0.0849
0.0851
0.0859
0.0859
0.0859
0.0862
0.0864
0.0864
0.0864
0.0864
0.0864
0.0864
0.0864
0.0864
0.0864
0.0864
0.0865
0.0885
0.0885
0.0885
0.0885
0.0903
0.0903
0.0923
0.0923
0.0923
0.0923
0.0923
0.0923
0.0923

0.0923
0.0923
0.0923
0.0935
0.0935
0.0935
0.0935
0.0935
0.0935
0.0935
0.0935
0.0935
0.0935
0.0935
0.0935
0.0935
0.0935
0.0935
0.0935
0.0935
0.0935
0.0935
0.0935
0.0935
0.0935
0.0935
0.0944
0.0950
0.0950
0.0950
0.0982
0.0982
0.1011
0.1011
0.1011
0.1011
0.1022
0.1022
0.1022
0.1022
0.1022
0.1022
0.1022
0.1022
0.1022
0.1022
0.1022
0.1022
0.1022
0.1022

0.1022
0.1022
0.1024
0.1024
0.1024
0.1024
0.1024
0.1024
0.1027
0.1027
0.1027
0.1027
0.1027
0.1027
0.1027
0.1027
0.1027
0.1027
0.1027
0.1027
0.1027
0.1027
0.1027
0.1027
0.1027
0.1027
0.1027
0.1027
0.1027
0.1027
0.1027
0.1027
0.1027
0.1027
0.1027
0.1027
0.1027
0.1027
0.1027
0.1027
0.1027
0.1027
0.1027
0.1027
0.1027
0.1027
0.1027
0.1027
0.1027
0.1027

0.1027
0.1027
0.1027
0.1027
0.1027
0.1027
0.1027
0.1027
0.1027
0.1027
0.1027
0.1027
0.1027
0.1027
0.1027
0.1027
0.1027
0.1027
0.1027
0.1027
0.1027
0.1027
0.1027
0.1027
0.1027
0.1027
0.1027
0.1027
0.1027
0.1027
0.1027
0.1027
0.1040
0.1040
0.1040
0.1047
0.1047
0.1047
0.1047
0.1047
0.1047
0.1047
0.1047
0.1047
0.1047
0.1047
0.1047
0.1047
0.1047
0.1047

0.1051
0.1051
0.1051
0.1052
0.1052
0.1052
0.1052
0.1052
0.1052
0.1052
0.1052
0.1052
0.1052
0.1052
0.1052
0.1052
0.1052
0.1052
0.1052
0.1052
0.1052
0.1052
0.1052
0.1052
0.1052
0.1052
0.1052
0.1052
0.1052
0.1056
0.1056
0.1056
0.1056
0.1060
0.1060
0.1060
0.1063
0.1066
0.1066
0.1066
0.1067
0.1072
0.1072
0.1072
0.1072
0.1072
0.1072
0.1072
0.1072
0.1072

0.1072
0.1072
0.1073
0.1077
0.1083
0.1087
0.1087
0.1087
0.1087
0.1087
0.1087
0.1087
0.1087
0.1090
0.1090
0.1090
0.1090
0.1090
0.1090
0.1090
0.1092
0.1092
0.1092
0.1092
0.1103
0.1103
0.1103
0.1103
0.1103
0.1103
0.1106
0.1108
0.1113
0.1113
0.1113
0.1114
0.1121
0.1127
0.1133
0.1133
0.1134
0.1134
0.1134
0.1134
0.1134
0.1134
0.1134
0.1134
0.1134
0.1134

0.1134
0.1134
0.1134
0.1134
0.1134
0.1134
0.1134
0.1134
0.1134
0.1134
0.1134
0.1134
0.1134
0.1134
0.1134
0.1134
0.1134
0.1134
0.1134
0.1134
0.1134
0.1134
0.1134
0.1134
0.1134
0.1134
0.1134
0.1134
0.1134
0.1134
0.1134
0.1134
0.1134
0.1134
0.1134
0.1134
0.1134
0.1134
0.1134
0.1134
0.1134
0.1134
0.1134
0.1134
0.1134
0.1134
0.1134
0.1134
0.1134
0.1134

0.1134
0.1134
0.1134
0.1134
0.1134
0.1134
0.1134
0.1134
0.1134
0.1134
0.1134
0.1134
0.1134
0.1134
0.1134
0.1134
0.1134
0.1134
0.1134
0.1134
0.1134
0.1134
0.1134
0.1134
0.1134
0.1134
0.1134
0.1134
0.1134
0.1134
0.1134
0.1134
0.1134
0.1134
0.1134
0.1134
0.1134
0.1134
0.1134
0.1134
0.1134
0.1134
0.1134
0.1134
0.1134
0.1134
0.1134
0.1134
0.1134
0.1134

0.1134
0.1134
0.1134
0.1134
0.1134
0.1144
0.1146
0.1146
0.1146
0.1146
0.1146
0.1146
0.1146
0.1146
0.1146
0.1151
0.1154
0.1154
0.1154
0.1154
0.1160
0.1162
0.1162
0.1162
0.1162
0.1162
0.1162
0.1162
0.1162
0.1162
0.1162
0.1162
0.1163
0.1163
0.1163
0.1163
0.1168
0.1180
0.1190
0.1190
0.1193
0.1193
0.1193
0.1193
0.1193
0.1193
0.1193
0.1193
0.1193
0.1193

0.1193
0.1193
0.1193
0.1193
0.1193
0.1193
0.1193
0.1193
0.1193
0.1193
0.1193
0.1193
0.1193
0.1193
0.1193
0.1193
0.1193
0.1193
0.1193
0.1193
0.1193
0.1193
0.1193
0.1193
0.1193
0.1193
0.1193
0.1193
0.1193
0.1193
0.1193
0.1193
0.1193
0.1193
0.1193
0.1194
0.1202
0.1202
0.1202
0.1203
0.1206
0.1206
0.1206
0.1206
0.1208
0.1208
0.1208
0.1208
0.1208
0.1208

0.1208
0.1208
0.1208
0.1208
0.1208
0.1208
0.1208
0.1208
0.1208
0.1208
0.1208
0.1208
0.1208
0.1208
0.1208
0.1209
0.1209
0.1209
0.1209
0.1209
0.1209
0.1209
0.1209
0.1209
0.1209
0.1209
0.1209
0.1209
0.1209
0.1209
0.1209
0.1209
0.1209
0.1214
0.1217
0.1218
0.1218
0.1218
0.1221
0.1221
0.1222
0.1222
0.1224
0.1224
0.1224
0.1224
0.1224
0.1224
0.1224
0.1224

0.1227
0.1230
0.1230
0.1231
0.1231
0.1236
0.1236
0.1236
0.1237
0.1237
0.1243
0.1243
0.1258
0.1258
0.1258
0.1258
0.1258
0.1258
0.1260
0.1260
0.1260
0.1266
0.1274
0.1274
0.1274
0.1274
0.1274
0.1274
0.1274
0.1274
0.1274
0.1274
0.1275
0.1275
0.1275
0.1275
0.1275
0.1275
0.1275
0.1275
0.1275
0.1280
0.1284
0.1284
0.1284
0.1285
0.1285
0.1285
0.1285
0.1292

0.1292
0.1292
0.1292
0.1292
0.1292
0.1294
0.1295
0.1295
0.1296
0.1297
0.1298
0.1298
0.1298
0.1298
0.1298
0.1298
0.1302
0.1302
0.1302
0.1304
0.1309
0.1313
0.1314
0.1314
0.1314
0.1314
0.1314
0.1314
0.1314
0.1314
0.1314
0.1314
0.1314
0.1314
0.1314
0.1323
0.1323
0.1323
0.1326
0.1326
0.1326
0.1326
0.1326
0.1326
0.1326
0.1326
0.1326
0.1326
0.1328
0.1328

0.1328
0.1328
0.1332
0.1332
0.1332
0.1332
0.1332
0.1332
0.1332
0.1332
0.1332
0.1332
0.1332
0.1332
0.1332
0.1332
0.1332
0.1332
0.1332
0.1332
0.1332
0.1332
0.1332
0.1332
0.1332
0.1332
0.1332
0.1332
0.1332
0.1332
0.1332
0.1332
0.1332
0.1332
0.1332
0.1332
0.1332
0.1332
0.1332
0.1332
0.1332
0.1332
0.1332
0.1332
0.1332
0.1332
0.1332
0.1332
0.1332
0.1332

0.1332
0.1332
0.1332
0.1332
0.1332
0.1335
0.1335
0.1335
0.1335
0.1335
0.1335
0.1335
0.1335
0.1335
0.1335
0.1335
0.1335
0.1335
0.1335
0.1335
0.1335
0.1335
0.1335
0.1335
0.1335
0.1335
0.1335
0.1335
0.1335
0.1335
0.1335
0.1335
0.1335
0.1335
0.1335
0.1335
0.1335
0.1335
0.1335
0.1335
0.1335
0.1335
0.1335
0.1335
0.1335
0.1335
0.1335
0.1335
0.1335
0.1335

0.1335
0.1335
0.1335
0.1335
0.1335
0.1335
0.1337
0.1337
0.1339
0.1339
0.1342
0.1342
0.1342
0.1344
0.1344
0.1344
0.1344
0.1344
0.1344
0.1344
0.1344
0.1344
0.1345
0.1345
0.1345
0.1345
0.1345
0.1345
0.1345
0.1345
0.1345
0.1345
0.1345
0.1345
0.1354
0.1354
0.1354
0.1354
0.1354
0.1354
0.1354
0.1354
0.1354
0.1354
0.1354
0.1354
0.1354
0.1359
0.1361
0.1361

0.1361
0.1361
0.1361
0.1361
0.1361
0.1361
0.1361
0.1361
0.1375
0.1375
0.1375
0.1377
0.1378
0.1378
0.1378
0.1378
0.1378
0.1378
0.1378
0.1378
0.1378
0.1384
0.1385
0.1386
0.1386
0.1387
0.1387
0.1387
0.1387
0.1393
0.1393
0.1393
0.1393
0.1393
0.1393
0.1393
0.1393
0.1393
0.1393
0.1393
0.1393
0.1393
0.1393
0.1393
0.1393
0.1394
0.1398
0.1407
0.1409
0.1409

0.1409
0.1409
0.1409
0.1409
0.1409
0.1409
0.1409
0.1409
0.1409
0.1409
0.1409
0.1409
0.1409
0.1409
0.1409
0.1409
0.1410
0.1410
0.1410
0.1410
0.1415
0.1415
0.1420
0.1425
0.1428
0.1428
0.1428
0.1428
0.1428
0.1428
0.1428
0.1428
0.1428
0.1428
0.1428
0.1428
0.1428
0.1428
0.1428
0.1428
0.1428
0.1428
0.1428
0.1428
0.1428
0.1428
0.1428
0.1429
0.1429
0.1429
0.1429

0.1429
0.1429
0.1429
0.1429
0.1429
0.1429
0.1429
0.1429
0.1429
0.1429
0.1429
0.1429
0.1429
0.1429
0.1429
0.1429
0.1429
0.1429
0.1429
0.1429
0.1429
0.1429
0.1429
0.1429
0.1429
0.1429
0.1429
0.1429
0.1429
0.1429
0.1429
0.1429
0.1429
0.1429
0.1429
0.1429
0.1431
0.1434
0.1434
0.1435
0.1436
0.1436
0.1436
0.1436
0.1436
0.1436
0.1436
0.1436
0.1436
0.1436

0.1437
0.1439
0.1439
0.1439
0.1439
0.1439
0.1439
0.1440
0.1440
0.1440
0.1442
0.1443
0.1443
0.1443
0.1446
0.1448
0.1451
0.1453
0.1453
0.1453
0.1453
0.1464
0.1466
0.1467
0.1472
0.1474
0.1475
0.1475
0.1475
0.1476
0.1476
0.1476
0.1477
0.1478
0.1478
0.1485
0.1489
0.1489
0.1489
0.1489
0.1489
0.1489
0.1489
0.1489
0.1489
0.1489
0.1489
0.1491
0.1492
0.1492

0.1492
0.1494
0.1498
0.1498
0.1498
0.1498
0.1498
0.1498
0.1498
0.1498
0.1498
0.1498
0.1498
0.1498
0.1498
0.1498
0.1498
0.1498
0.1498
0.1499
0.1500
0.1500
0.1500
0.1500
0.1500
0.1500
0.1500
0.1500
0.1500
0.1500
0.1500
0.1500
0.1500
0.1500
0.1500
0.1500
0.1500
0.1500
0.1500
0.1500
0.1500
0.1500
0.1500
0.1500
0.1500
0.1500
0.1500
0.1500
0.1500
0.1500

0.1501
0.1501
0.1501
0.1501
0.1501
0.1501
0.1501
0.1501
0.1503
0.1504
0.1504
0.1504
0.1504
0.1505
0.1508
0.1508
0.1508
0.1508
0.1508
0.1508
0.1508
0.1508
0.1508
0.1508
0.1508
0.1509
0.1509
0.1509
0.1509
0.1509
0.1509
0.1510
0.1510
0.1511
0.1511
0.1512
0.1512
0.1512
0.1512
0.1512
0.1512
0.1512
0.1512
0.1512
0.1512
0.1512
0.1515
0.1515
0.1515
0.1515

0.1515
0.1515
0.1515
0.1515
0.1515
0.1515
0.1516
0.1516
0.1516
0.1516
0.1518
0.1518
0.1519
0.1522
0.1524
0.1524
0.1524
0.1524
0.1524
0.1524
0.1524
0.1524
0.1524
0.1526
0.1526
0.1526
0.1526
0.1527
0.1529
0.1532
0.1533
0.1533
0.1533
0.1533
0.1533
0.1534
0.1535
0.1535
0.1535
0.1535
0.1535
0.1536
0.1536
0.1536
0.1536
0.1536
0.1536
0.1536
0.1536
0.1536

0.1536
0.1536
0.1536
0.1536
0.1536
0.1536
0.1536
0.1536
0.1536
0.1536
0.1536
0.1536
0.1536
0.1536
0.1536
0.1536
0.1536
0.1536
0.1536
0.1536
0.1536
0.1536
0.1536
0.1536
0.1536
0.1536
0.1536
0.1536
0.1536
0.1536
0.1536
0.1536
0.1536
0.1536
0.1536
0.1536
0.1536
0.1536
0.1536
0.1536
0.1536
0.1536
0.1536
0.1536
0.1538
0.1538
0.1538
0.1539
0.1539
0.1539

0.1539
0.1539
0.1540
0.1540
0.1547
0.1551
0.1551
0.1551
0.1551
0.1551
0.1553
0.1553
0.1555
0.1555
0.1560
0.1563
0.1563
0.1563
0.1563
0.1563
0.1563
0.1563
0.1563
0.1563
0.1567
0.1568
0.1568
0.1568
0.1568
0.1568
0.1568
0.1568
0.1568
0.1568
0.1568
0.1568
0.1568
0.1568
0.1568
0.1568
0.1568
0.1568
0.1568
0.1568
0.1568
0.1570
0.1571
0.1573
0.1574
0.1574

0.1576
0.1577
0.1577
0.1577
0.1577
0.1577
0.1577
0.1577
0.1577
0.1577
0.1577
0.1577
0.1577
0.1577
0.1577
0.1577
0.1577
0.1577
0.1577
0.1579
0.1579
0.1579
0.1579
0.1579
0.1580
0.1580
0.1584
0.1586
0.1586
0.1587
0.1587
0.1588
0.1588
0.1588
0.1588
0.1588
0.1588
0.1588
0.1588
0.1588
0.1588
0.1588
0.1588
0.1588
0.1588
0.1589
0.1589
0.1589
0.1589
0.1589

0.1589
0.1589
0.1589
0.1589
0.1589
0.1589
0.1589
0.1589
0.1589
0.1589
0.1589
0.1589
0.1589
0.1589
0.1589
0.1589
0.1589
0.1589
0.1589
0.1590
0.1590
0.1596
0.1596
0.1596
0.1596
0.1596
0.1596
0.1596
0.1596
0.1597
0.1597
0.1597
0.1597
0.1597
0.1597
0.1597
0.1597
0.1597
0.1597
0.1598
0.1598
0.1598
0.1599
0.1599
0.1600
0.1602
0.1604
0.1604
0.1604
0.1604

0.1604
0.1607
0.1607
0.1607
0.1607
0.1607
0.1608
0.1608
0.1608
0.1608
0.1609
0.1609
0.1610
0.1610
0.1611
0.1615
0.1615
0.1615
0.1615
0.1615
0.1615
0.1619
0.1619
0.1619
0.1619
0.1619
0.1620
0.1620
0.1620
0.1620
0.1620
0.1620
0.1620
0.1620
0.1622
0.1624
0.1624
0.1624
0.1624
0.1628
0.1629
0.1633
0.1633
0.1633
0.1633
0.1633
0.1633
0.1633
0.1633
0.1633

0.1633
0.1636
0.1636
0.1640
0.1640
0.1642
0.1645
0.1645
0.1645
0.1645
0.1645
0.1645
0.1647
0.1649
0.1649
0.1650
0.1650
0.1650
0.1651
0.1651
0.1653
0.1655
0.1655
0.1659
0.1660
0.1662
0.1662
0.1664
0.1664
0.1664
0.1664
0.1665
0.1666
0.1667
0.1667
0.1667
0.1667
0.1667
0.1667
0.1667
0.1667
0.1667
0.1667
0.1667
0.1667
0.1668
0.1668
0.1668
0.1668
0.1668

0.1668
0.1668
0.1669
0.1669
0.1670
0.1670
0.1671
0.1674
0.1674
0.1674
0.1674
0.1675
0.1675
0.1679
0.1680
0.1682
0.1684
0.1687
0.1689
0.1689
0.1689
0.1690
0.1690
0.1692
0.1694
0.1696
0.1696
0.1696
0.1696
0.1697
0.1697
0.1697
0.1697
0.1697
0.1697
0.1697
0.1697
0.1700
0.1701
0.1701
0.1701
0.1701
0.1702
0.1703
0.1703
0.1704
0.1704
0.1704
0.1706
0.1706

0.1709
0.1709
0.1709
0.1710
0.1716
0.1716
0.1716
0.1716
0.1718
0.1718
0.1718
0.1718
0.1718
0.1718
0.1721
0.1721
0.1722
0.1723
0.1723
0.1723
0.1723
0.1723
0.1723
0.1725
0.1725
0.1727
0.1727
0.1727
0.1728
0.1728
0.1731
0.1731
0.1732
0.1732
0.1732
0.1732
0.1733
0.1733
0.1733
0.1733
0.1733
0.1733
0.1733
0.1733
0.1733
0.1733
0.1733
0.1733
0.1735
0.1735

0.1735
0.1736
0.1739
0.1739
0.1739
0.1741
0.1741
0.1741
0.1741
0.1741
0.1743
0.1745
0.1745
0.1745
0.1747
0.1747
0.1747
0.1747
0.1747
0.1747
0.1747
0.1747
0.1747
0.1747
0.1747
0.1747
0.1747
0.1747
0.1747
0.1747
0.1747
0.1747
0.1747
0.1747
0.1747
0.1747
0.1747
0.1747
0.1747
0.1747
0.1748
0.1749
0.1751
0.1753
0.1753
0.1753
0.1753
0.1753
0.1753
0.1753
0.1756

0.1756
0.1756
0.1756
0.1756
0.1756
0.1756
0.1756
0.1758
0.1758
0.1758
0.1758
0.1758
0.1758
0.1758
0.1762
0.1764
0.1767
0.1767
0.1767
0.1767
0.1767
0.1767
0.1768
0.1771
0.1771
0.1778
0.1778
0.1778
0.1778
0.1778
0.1778
0.1779
0.1779
0.1779
0.1779
0.1779
0.1779
0.1779
0.1779
0.1779
0.1779
0.1779
0.1779
0.1779
0.1779
0.1779
0.1780
0.1780
0.1783
0.1783

0.1783
0.1783
0.1783
0.1783
0.1783
0.1783
0.1783
0.1783
0.1783
0.1783
0.1783
0.1785
0.1785
0.1785
0.1785
0.1785
0.1785
0.1785
0.1785
0.1785
0.1785
0.1785
0.1785
0.1785
0.1785
0.1787
0.1787
0.1787
0.1787
0.1789
0.1790
0.1792
0.1792
0.1792
0.1794
0.1795
0.1795
0.1795
0.1795
0.1795
0.1795
0.1798
0.1800
0.1800
0.1800
0.1800
0.1800
0.1809
0.1809
0.1809

0.1810
0.1810
0.1814
0.1814
0.1814
0.1814
0.1814
0.1814
0.1814
0.1814
0.1814
0.1814
0.1814
0.1814
0.1814
0.1814
0.1814
0.1814
0.1814
0.1814
0.1814
0.1814
0.1814
0.1814
0.1814
0.1815
0.1815
0.1816
0.1816
0.1823
0.1825
0.1825
0.1829
0.1830
0.1830
0.1830
0.1830
0.1830
0.1830
0.1830
0.1830
0.1830
0.1830
0.1830
0.1830
0.1830
0.1830
0.1830
0.1830
0.1830

0.1830
0.1830
0.1830
0.1830
0.1830
0.1830
0.1830
0.1830
0.1836
0.1836
0.1837
0.1837
0.1837
0.1842
0.1842
0.1842
0.1842
0.1848
0.1850
0.1851
0.1851
0.1853
0.1859
0.1860
0.1864
0.1864
0.1866
0.1867
0.1867
0.1868
0.1868
0.1868
0.1868
0.1869
0.1875
0.1875
0.1875
0.1875
0.1875
0.1875
0.1875
0.1876
0.1876
0.1876
0.1881
0.1881
0.1881
0.1881
0.1881
0.1882

0.1882
0.1883
0.1886
0.1886
0.1886
0.1886
0.1887
0.1888
0.1888
0.1888
0.1888
0.1888
0.1888
0.1889
0.1890
0.1890
0.1890
0.1890
0.1890
0.1893
0.1893
0.1894
0.1894
0.1894
0.1894
0.1894
0.1894
0.1894
0.1894
0.1894
0.1894
0.1894
0.1894
0.1894
0.1894
0.1894
0.1894
0.1895
0.1895
0.1895
0.1897
0.1897
0.1897
0.1899
0.1902
0.1902
0.1902
0.1903
0.1903
0.1904

0.1904
0.1904
0.1904
0.1904
0.1906
0.1906
0.1906
0.1906
0.1911
0.1911
0.1911
0.1913
0.1914
0.1914
0.1917
0.1917
0.1917
0.1919
0.1924
0.1930
0.1941
0.1947
0.1947
0.1947
0.1950
0.1951
0.1951
0.1952
0.1952
0.1954
0.1954
0.1954
0.1954
0.1954
0.1954
0.1954
0.1954
0.1954
0.1954
0.1954
0.1954
0.1954
0.1957
0.1958
0.1959
0.1959
0.1959
0.1959
0.1959
0.1959

0.1959
0.1959
0.1959
0.1959
0.1959
0.1959
0.1959
0.1959
0.1959
0.1959
0.1960
0.1962
0.1963
0.1963
0.1963
0.1963
0.1967
0.1967
0.1968
0.1970
0.1970
0.1974
0.1974
0.1974
0.1974
0.1979
0.1981
0.1981
0.1981
0.1981
0.1982
0.1984
0.1984
0.1984
0.1985
0.1987
0.1989
0.1989
0.1992
0.1993
0.1994
0.1994
0.1996
0.1997
0.1997
0.2001
0.2003
0.2003
0.2003
0.2003

0.2003
0.2003
0.2004
0.2004
0.2004
0.2004
0.2006
0.2008
0.2008
0.2008
0.2009
0.2009
0.2010
0.2010
0.2010
0.2010
0.2010
0.2010
0.2010
0.2010
0.2010
0.2010
0.2010
0.2010
0.2010
0.2010
0.2010
0.2010
0.2010
0.2011
0.2011
0.2011
0.2011
0.2011
0.2011
0.2013
0.2015
0.2015
0.2015
0.2015
0.2015
0.2015
0.2015
0.2016
0.2016
0.2020
0.2021
0.2021
0.2021
0.2021

0.2021
0.2021
0.2022
0.2024
0.2025
0.2028
0.2028
0.2034
0.2034
0.2034
0.2036
0.2036
0.2036
0.2036
0.2037
0.2037
0.2039
0.2039
0.2041
0.2043
0.2044
0.2044
0.2047
0.2050
0.2051
0.2051
0.2053
0.2053
0.2053
0.2053
0.2053
0.2054
0.2054
0.2059
0.2059
0.2059
0.2059
0.2059
0.2059
0.2061
0.2062
0.2063
0.2063
0.2063
0.2063
0.2063
0.2063
0.2064
0.2064
0.2064

0.2064
0.2064
0.2064
0.2067
0.2067
0.2067
0.2068
0.2068
0.2068
0.2071
0.2071
0.2071
0.2071
0.2071
0.2071
0.2071
0.2073
0.2074
0.2074
0.2078
0.2078
0.2080
0.2080
0.2080
0.2080
0.2080
0.2080
0.2080
0.2080
0.2080
0.2080
0.2080
0.2080
0.2080
0.2080
0.2080
0.2080
0.2080
0.2080
0.2080
0.2086
0.2086
0.2087
0.2087
0.2088
0.2088
0.2088
0.2088
0.2089
0.2089

0.2089
0.2089
0.2089
0.2093
0.2093
0.2094
0.2095
0.2095
0.2095
0.2095
0.2095
0.2095
0.2095
0.2095
0.2095
0.2095
0.2095
0.2095
0.2095
0.2096
0.2096
0.2096
0.2105
0.2105
0.2105
0.2110
0.2110
0.2111
0.2111
0.2111
0.2111
0.2111
0.2113
0.2113
0.2113
0.2113
0.2113
0.2113
0.2113
0.2113
0.2114
0.2114
0.2114
0.2114
0.2114
0.2114
0.2117
0.2117
0.2117
0.2118

0.2123
0.2123
0.2123
0.2128
0.2128
0.2128
0.2132
0.2132
0.2132
0.2133
0.2136
0.2138
0.2138
0.2138
0.2138
0.2141
0.2141
0.2141
0.2142
0.2142
0.2142
0.2143
0.2143
0.2143
0.2146
0.2146
0.2146
0.2146
0.2146
0.2146
0.2146
0.2149
0.2150
0.2152
0.2152
0.2156
0.2156
0.2156
0.2158
0.2158
0.2158
0.2158
0.2160
0.2160
0.2164
0.2166
0.2168
0.2170
0.2171
0.2172

0.2175
0.2175
0.2175
0.2177
0.2180
0.2180
0.2180
0.2183
0.2183
0.2183
0.2183
0.2184
0.2185
0.2186
0.2186
0.2188
0.2188
0.2190
0.2190
0.2191
0.2194
0.2198
0.2200
0.2201
0.2204
0.2204
0.2208
0.2208
0.2208
0.2210
0.2210
0.2210
0.2210
0.2211
0.2213
0.2214
0.2214
0.2216
0.2219
0.2222
0.2223
0.2223
0.2223
0.2223
0.2223
0.2223
0.2223
0.2223
0.2223
0.2223

0.2223
0.2224
0.2224
0.2225
0.2229
0.2229
0.2229
0.2229
0.2229
0.2229
0.2231
0.2231
0.2231
0.2231
0.2232
0.2232
0.2233
0.2233
0.2236
0.2236
0.2236
0.2236
0.2238
0.2238
0.2238
0.2238
0.2238
0.2238
0.2238
0.2238
0.2238
0.2239
0.2242
0.2243
0.2243
0.2243
0.2249
0.2249
0.2250
0.2250
0.2252
0.2252
0.2252
0.2252
0.2252
0.2252
0.2252
0.2252
0.2252
0.2252

0.2252
0.2252
0.2252
0.2252
0.2252
0.2256
0.2256
0.2257
0.2257
0.2257
0.2257
0.2257
0.2257
0.2259
0.2259
0.2260
0.2261
0.2263
0.2263
0.2263
0.2263
0.2263
0.2264
0.2264
0.2264
0.2264
0.2267
0.2269
0.2269
0.2269
0.2269
0.2269
0.2269
0.2269
0.2271
0.2271
0.2273
0.2273
0.2275
0.2275
0.2275
0.2275
0.2275
0.2275
0.2275
0.2275
0.2275
0.2275
0.2275
0.2275

0.2275
0.2275
0.2275
0.2275
0.2275
0.2275
0.2275
0.2277
0.2277
0.2277
0.2277
0.2277
0.2278
0.2278
0.2278
0.2278
0.2278
0.2278
0.2278
0.2278
0.2278
0.2279
0.2279
0.2279
0.2279
0.2279
0.2279
0.2279
0.2279
0.2279
0.2279
0.2279
0.2279
0.2279
0.2279
0.2279
0.2279
0.2279
0.2279
0.2279
0.2279
0.2280
0.2280
0.2280
0.2280
0.2281
0.2281
0.2281
0.2281
0.2281

0.2281
0.2281
0.2281
0.2281
0.2281
0.2281
0.2281
0.2281
0.2282
0.2282
0.2283
0.2283
0.2283
0.2283
0.2284
0.2284
0.2284
0.2284
0.2285
0.2285
0.2285
0.2286
0.2286
0.2286
0.2289
0.2289
0.2291
0.2291
0.2293
0.2293
0.2293
0.2294
0.2294
0.2294
0.2296
0.2297
0.2297
0.2297
0.2297
0.2297
0.2297
0.2297
0.2297
0.2297
0.2298
0.2301
0.2302
0.2302
0.2304
0.2304

0.2305
0.2305
0.2312
0.2313
0.2315
0.2318
0.2318
0.2318
0.2318
0.2319
0.2320
0.2324
0.2324
0.2325
0.2325
0.2325
0.2326
0.2326
0.2326
0.2326
0.2326
0.2326
0.2326
0.2326
0.2326
0.2327
0.2327
0.2328
0.2329
0.2329
0.2329
0.2329
0.2329
0.2329
0.2329
0.2329
0.2330
0.2333
0.2333
0.2333
0.2333
0.2335
0.2335
0.2335
0.2336
0.2337
0.2338
0.2338
0.2342
0.2343

0.2344
0.2347
0.2347
0.2347
0.2347
0.2347
0.2347
0.2347
0.2347
0.2347
0.2347
0.2351
0.2352
0.2352
0.2354
0.2354
0.2354
0.2355
0.2356
0.2356
0.2358
0.2359
0.2364
0.2366
0.2366
0.2366
0.2366
0.2366
0.2366
0.2366
0.2366
0.2366
0.2366
0.2366
0.2366
0.2367
0.2371
0.2371
0.2371
0.2371
0.2371
0.2372
0.2374
0.2374
0.2375
0.2375
0.2375
0.2379
0.2379
0.2379

0.2379
0.2379
0.2379
0.2379
0.2379
0.2379
0.2379
0.2380
0.2380
0.2380
0.2381
0.2381
0.2381
0.2381
0.2382
0.2384
0.2387
0.2391
0.2391
0.2394
0.2395
0.2396
0.2396
0.2399
0.2400
0.2400
0.2400
0.2401
0.2401
0.2401
0.2402
0.2403
0.2403
0.2404
0.2407
0.2407
0.2407
0.2407
0.2407
0.2409
0.2410
0.2410
0.2410
0.2411
0.2411
0.2411
0.2412
0.2412
0.2412
0.2412

0.2412
0.2412
0.2412
0.2412
0.2415
0.2418
0.2418
0.2421
0.2421
0.2422
0.2423
0.2423
0.2423
0.2423
0.2423
0.2423
0.2423
0.2423
0.2423
0.2423
0.2423
0.2423
0.2423
0.2424
0.2424
0.2424
0.2424
0.2424
0.2425
0.2428
0.2428
0.2428
0.2428
0.2428
0.2433
0.2434
0.2434
0.2434
0.2435
0.2436
0.2438
0.2438
0.2438
0.2438
0.2438
0.2438
0.2438
0.2438
0.2438
0.2438

0.2438
0.2438
0.2438
0.2439
0.2439
0.2439
0.2439
0.2439
0.2440
0.2440
0.2440
0.2440
0.2440
0.2440
0.2440
0.2440
0.2441
0.2441
0.2441
0.2441
0.2441
0.2441
0.2441
0.2441
0.2441
0.2441
0.2441
0.2441
0.2441
0.2441
0.2441
0.2441
0.2441
0.2441
0.2442
0.2442
0.2442
0.2442
0.2442
0.2442
0.2442
0.2442
0.2442
0.2442
0.2443
0.2447
0.2447
0.2448
0.2448
0.2449

0.2452
0.2453
0.2454
0.2454
0.2454
0.2454
0.2454
0.2454
0.2454
0.2457
0.2457
0.2457
0.2457
0.2457
0.2457
0.2457
0.2459
0.2459
0.2460
0.2463
0.2465
0.2472
0.2472
0.2473
0.2473
0.2475
0.2475
0.2476
0.2478
0.2478
0.2478
0.2478
0.2479
0.2480
0.2481
0.2483
0.2483
0.2484
0.2486
0.2487
0.2488
0.2488
0.2488
0.2491
0.2491
0.2491
0.2492
0.2493
0.2495
0.2495

0.2495
0.2495
0.2495
0.2495
0.2495
0.2499
0.2499
0.2500
0.2500
0.2502
0.2502
0.2502
0.2504
0.2504
0.2504
0.2504
0.2504
0.2505
0.2505
0.2505
0.2505
0.2505
0.2505
0.2505
0.2505
0.2505
0.2508
0.2508
0.2508
0.2508
0.2508
0.2508
0.2509
0.2509
0.2509
0.2512
0.2512
0.2512
0.2513
0.2514
0.2516
0.2517
0.2517
0.2517
0.2517
0.2517
0.2517
0.2517
0.2517
0.2517

0.2517
0.2517
0.2517
0.2517
0.2517
0.2517
0.2517
0.2517
0.2517
0.2517
0.2517
0.2517
0.2517
0.2517
0.2517
0.2517
0.2518
0.2518
0.2518
0.2518
0.2518
0.2519
0.2520
0.2520
0.2520
0.2520
0.2520
0.2520
0.2520
0.2520
0.2520
0.2520
0.2520
0.2520
0.2520
0.2520
0.2520
0.2520
0.2520
0.2520
0.2520
0.2520
0.2520
0.2520
0.2520
0.2520
0.2520
0.2520
0.2520
0.2520

0.2521
0.2521
0.2521
0.2521
0.2521
0.2521
0.2521
0.2523
0.2523
0.2523
0.2523
0.2524
0.2525
0.2525
0.2525
0.2525
0.2525
0.2525
0.2525
0.2525
0.2525
0.2525
0.2525
0.2525
0.2525
0.2525
0.2525
0.2525
0.2525
0.2525
0.2525
0.2530
0.2530
0.2530
0.2530
0.2530
0.2533
0.2533
0.2533
0.2533
0.2533
0.2533
0.2533
0.2533
0.2533
0.2535
0.2535
0.2535
0.2535
0.2536

0.2536
0.2536
0.2537
0.2537
0.2537
0.2537
0.2538
0.2538
0.2538
0.2538
0.2538
0.2538
0.2538
0.2538
0.2538
0.2541
0.2545
0.2547
0.2547
0.2547
0.2547
0.2548
0.2548
0.2548
0.2549
0.2549
0.2550
0.2550
0.2550
0.2550
0.2550
0.2550
0.2550
0.2551
0.2551
0.2551
0.2552
0.2553
0.2553
0.2553
0.2553
0.2554
0.2555
0.2558
0.2558
0.2558
0.2560
0.2560
0.2560
0.2561

0.2564
0.2565
0.2565
0.2565
0.2566
0.2567
0.2567
0.2567
0.2567
0.2567
0.2567
0.2567
0.2567
0.2567
0.2567
0.2568
0.2569
0.2572
0.2573
0.2577
0.2579
0.2579
0.2580
0.2582
0.2582
0.2583
0.2584
0.2584
0.2584
0.2587
0.2587
0.2587
0.2587
0.2587
0.2590
0.2590
0.2590
0.2591
0.2593
0.2593
0.2593
0.2593
0.2593
0.2593
0.2593
0.2593
0.2597
0.2597
0.2598
0.2598

0.2598
0.2598
0.2598
0.2598
0.2601
0.2606
0.2609
0.2610
0.2610
0.2610
0.2610
0.2612
0.2612
0.2612
0.2612
0.2612
0.2614
0.2614
0.2616
0.2617
0.2617
0.2617
0.2618
0.2620
0.2621
0.2624
0.2624
0.2625
0.2625
0.2625
0.2625
0.2625
0.2625
0.2629
0.2632
0.2634
0.2634
0.2634
0.2634
0.2634
0.2634
0.2636
0.2636
0.2638
0.2638
0.2638
0.2638
0.2638
0.2638
0.2639

0.2639
0.2639
0.2639
0.2639
0.2639
0.2639
0.2639
0.2640
0.2640
0.2640
0.2640
0.2640
0.2640
0.2640
0.2640
0.2640
0.2640
0.2640
0.2640
0.2640
0.2640
0.2643
0.2643
0.2643
0.2644
0.2645
0.2645
0.2646
0.2646
0.2646
0.2646
0.2646
0.2646
0.2646
0.2646
0.2646
0.2646
0.2646
0.2646
0.2646
0.2646
0.2646
0.2646
0.2646
0.2647
0.2648
0.2650
0.2650
0.2650
0.2654

0.2654
0.2654
0.2654
0.2654
0.2654
0.2654
0.2654
0.2654
0.2654
0.2654
0.2654
0.2654
0.2654
0.2654
0.2654
0.2654
0.2654
0.2655
0.2655
0.2655
0.2655
0.2655
0.2655
0.2655
0.2655
0.2655
0.2655
0.2655
0.2655
0.2655
0.2655
0.2655
0.2655
0.2655
0.2655
0.2655
0.2656
0.2656
0.2656
0.2657
0.2657
0.2657
0.2657
0.2657
0.2657
0.2657
0.2669
0.2669
0.2671
0.2671

0.2672
0.2674
0.2674
0.2674
0.2674
0.2675
0.2675
0.2676
0.2676
0.2676
0.2676
0.2677
0.2677
0.2677
0.2677
0.2677
0.2677
0.2678
0.2678
0.2678
0.2678
0.2678
0.2679
0.2680
0.2684
0.2684
0.2684
0.2685
0.2685
0.2685
0.2686
0.2686
0.2688
0.2688
0.2688
0.2689
0.2691
0.2691
0.2695
0.2696
0.2698
0.2699
0.2700
0.2703
0.2704
0.2704
0.2704
0.2704
0.2704
0.2704

0.2704
0.2704
0.2704
0.2704
0.2704
0.2706
0.2707
0.2707
0.2707
0.2709
0.2709
0.2711
0.2714
0.2717
0.2717
0.2717
0.2717
0.2717
0.2722
0.2722
0.2722
0.2722
0.2722
0.2723
0.2723
0.2730
0.2732
0.2732
0.2732
0.2732
0.2732
0.2736
0.2736
0.2736
0.2736
0.2736
0.2738
0.2742
0.2743
0.2744
0.2744
0.2744
0.2744
0.2746
0.2746
0.2747
0.2748
0.2748
0.2748
0.2748

0.2750
0.2753
0.2753
0.2754
0.2756
0.2757
0.2758
0.2759
0.2759
0.2759
0.2759
0.2759
0.2760
0.2760
0.2763
0.2763
0.2764
0.2767
0.2767
0.2768
0.2768
0.2768
0.2768
0.2770
0.2770
0.2772
0.2772
0.2772
0.2772
0.2772
0.2772
0.2772
0.2772
0.2772
0.2773
0.2774
0.2778
0.2779
0.2781
0.2782
0.2782
0.2782
0.2782
0.2784
0.2784
0.2784
0.2784
0.2784
0.2784
0.2784

0.2785
0.2786
0.2786
0.2786
0.2786
0.2787
0.2787
0.2789
0.2789
0.2789
0.2790
0.2792
0.2792
0.2796
0.2796
0.2799
0.2799
0.2799
0.2799
0.2799
0.2800
0.2803
0.2803
0.2803
0.2803
0.2803
0.2803
0.2803
0.2803
0.2803
0.2803
0.2803
0.2803
0.2803
0.2803
0.2803
0.2803
0.2803
0.2803
0.2803
0.2803
0.2803
0.2803
0.2803
0.2803
0.2803
0.2803
0.2803
0.2803
0.2803
0.2803

0.2803
0.2804
0.2805
0.2805
0.2805
0.2805
0.2807
0.2807
0.2807
0.2807
0.2807
0.2808
0.2810
0.2810
0.2810
0.2810
0.2810
0.2811
0.2811
0.2813
0.2813
0.2813
0.2814
0.2814
0.2814
0.2815
0.2815
0.2815
0.2816
0.2817
0.2817
0.2817
0.2817
0.2817
0.2817
0.2817
0.2817
0.2817
0.2817
0.2817
0.2817
0.2817
0.2817
0.2817
0.2817
0.2817
0.2817
0.2817
0.2817
0.2817

0.2817
0.2817
0.2817
0.2817
0.2817
0.2817
0.2817
0.2818
0.2818
0.2818
0.2819
0.2820
0.2821
0.2821
0.2821
0.2821
0.2822
0.2823
0.2823
0.2823
0.2823
0.2823
0.2824
0.2824
0.2824
0.2824
0.2824
0.2824
0.2825
0.2825
0.2825
0.2825
0.2825
0.2825
0.2825
0.2825
0.2826
0.2827
0.2832
0.2832
0.2834
0.2834
0.2836
0.2837
0.2839
0.2839
0.2843
0.2843
0.2843
0.2843

0.2843
0.2843
0.2845
0.2850
0.2850
0.2850
0.2851
0.2851
0.2851
0.2851
0.2851
0.2851
0.2851
0.2851
0.2851
0.2851
0.2851
0.2851
0.2851
0.2851
0.2851
0.2851
0.2851
0.2851
0.2851
0.2853
0.2853
0.2853
0.2856
0.2857
0.2857
0.2857
0.2858
0.2858
0.2858
0.2858
0.2858
0.2861
0.2862
0.2867
0.2869
0.2869
0.2869
0.2869
0.2869
0.2869
0.2873
0.2873
0.2875
0.2875

0.2875
0.2875
0.2875
0.2875
0.2875
0.2875
0.2876
0.2877
0.2877
0.2877
0.2877
0.2877
0.2877
0.2877
0.2877
0.2877
0.2878
0.2879
0.2879
0.2879
0.2879
0.2880
0.2880
0.2880
0.2880
0.2880
0.2880
0.2880
0.2880
0.2880
0.2880
0.2880
0.2880
0.2880
0.2880
0.2880
0.2880
0.2880
0.2880
0.2880
0.2880
0.2880
0.2880
0.2880
0.2880
0.2880
0.2881
0.2885
0.2885
0.2885

0.2886
0.2886
0.2887
0.2890
0.2890
0.2890
0.2892
0.2895
0.2895
0.2898
0.2898
0.2901
0.2901
0.2903
0.2907
0.2908
0.2914
0.2914
0.2914
0.2914
0.2914
0.2914
0.2914
0.2914
0.2915
0.2916
0.2916
0.2916
0.2916
0.2916
0.2918
0.2920
0.2920
0.2922
0.2925
0.2925
0.2927
0.2927
0.2928
0.2929
0.2929
0.2932
0.2932
0.2932
0.2932
0.2935
0.2936
0.2938
0.2938
0.2939

0.2939
0.2940
0.2942
0.2946
0.2946
0.2946
0.2946
0.2948
0.2948
0.2948
0.2948
0.2948
0.2948
0.2948
0.2948
0.2948
0.2951
0.2951
0.2951
0.2951
0.2951
0.2951
0.2951
0.2951
0.2951
0.2951
0.2951
0.2951
0.2951
0.2951
0.2951
0.2951
0.2951
0.2951
0.2951
0.2952
0.2952
0.2952
0.2952
0.2952
0.2952
0.2952
0.2954
0.2954
0.2954
0.2954
0.2954
0.2957
0.2957
0.2957

0.2961
0.2961
0.2961
0.2961
0.2961
0.2961
0.2961
0.2963
0.2965
0.2968
0.2968
0.2969
0.2969
0.2969
0.2969
0.2969
0.2969
0.2971
0.2971
0.2972
0.2973
0.2975
0.2980
0.2981
0.2981
0.2981
0.2983
0.2983
0.2984
0.2985
0.2986
0.2987
0.2987
0.2987
0.2987
0.2987
0.2991
0.2991
0.2992
0.2992
0.2994
0.2994
0.2994
0.2994
0.2996
0.3000
0.3000
0.3001
0.3001
0.3001

0.3002
0.3002
0.3002
0.3002
0.3002
0.3002
0.3003
0.3003
0.3004
0.3005
0.3006
0.3008
0.3008
0.3010
0.3012
0.3012
0.3012
0.3012
0.3012
0.3014
0.3014
0.3016
0.3020
0.3021
0.3021
0.3021
0.3021
0.3021
0.3021
0.3025
0.3026
0.3028
0.3028
0.3028
0.3028
0.3028
0.3028
0.3028
0.3028
0.3028
0.3028
0.3029
0.3029
0.3030
0.3030
0.3030
0.3030
0.3033
0.3033
0.3033

0.3033
0.3033
0.3034
0.3034
0.3034
0.3034
0.3034
0.3034
0.3035
0.3035
0.3037
0.3037
0.3038
0.3042
0.3046
0.3046
0.3046
0.3046
0.3046
0.3046
0.3046
0.3046
0.3046
0.3046
0.3046
0.3046
0.3046
0.3046
0.3046
0.3048
0.3050
0.3050
0.3050
0.3050
0.3050
0.3050
0.3051
0.3054
0.3054
0.3054
0.3054
0.3058
0.3058
0.3058
0.3058
0.3059
0.3060
0.3063
0.3067
0.3068

0.3069
0.3069
0.3073
0.3074
0.3075
0.3077
0.3077
0.3077
0.3077
0.3078
0.3078
0.3078
0.3078
0.3081
0.3083
0.3084
0.3087
0.3088
0.3089
0.3093
0.3094
0.3094
0.3095
0.3095
0.3095
0.3095
0.3095
0.3095
0.3096
0.3096
0.3099
0.3100
0.3101
0.3101
0.3102
0.3102
0.3102
0.3104
0.3104
0.3104
0.3107
0.3108
0.3108
0.3108
0.3108
0.3108
0.3109
0.3109
0.3109
0.3110

0.3110
0.3110
0.3110
0.3110
0.3110
0.3110
0.3110
0.3111
0.3112
0.3112
0.3112
0.3112
0.3112
0.3114
0.3114
0.3115
0.3117
0.3118
0.3120
0.3120
0.3120
0.3120
0.3120
0.3120
0.3120
0.3120
0.3120
0.3122
0.3126
0.3126
0.3126
0.3128
0.3128
0.3128
0.3129
0.3129
0.3129
0.3129
0.3129
0.3129
0.3129
0.3129
0.3130
0.3130
0.3136
0.3136
0.3136
0.3136
0.3136
0.3138

0.3141
0.3142
0.3142
0.3143
0.3143
0.3143
0.3144
0.3144
0.3144
0.3144
0.3145
0.3145
0.3145
0.3145
0.3145
0.3145
0.3145
0.3145
0.3145
0.3145
0.3147
0.3147
0.3147
0.3151
0.3151
0.3151
0.3153
0.3155
0.3155
0.3157
0.3157
0.3157
0.3157
0.3158
0.3161
0.3161
0.3161
0.3161
0.3162
0.3162
0.3162
0.3162
0.3163
0.3163
0.3163
0.3163
0.3163
0.3165
0.3168
0.3168

0.3168
0.3168
0.3170
0.3171
0.3171
0.3171
0.3171
0.3173
0.3173
0.3174
0.3174
0.3175
0.3178
0.3185
0.3186
0.3188
0.3188
0.3190
0.3190
0.3191
0.3194
0.3195
0.3195
0.3195
0.3195
0.3196
0.3197
0.3197
0.3197
0.3203
0.3203
0.3203
0.3203
0.3203
0.3203
0.3203
0.3207
0.3207
0.3209
0.3212
0.3218
0.3218
0.3218
0.3218
0.3218
0.3218
0.3218
0.3221
0.3222
0.3222

0.3222
0.3224
0.3224
0.3225
0.3225
0.3225
0.3230
0.3230
0.3231
0.3231
0.3231
0.3231
0.3231
0.3231
0.3232
0.3234
0.3236
0.3238
0.3238
0.3238
0.3238
0.3238
0.3239
0.3239
0.3241
0.3241
0.3241
0.3246
0.3247
0.3247
0.3247
0.3247
0.3247
0.3247
0.3247
0.3247
0.3247
0.3249
0.3249
0.3249
0.3249
0.3249
0.3249
0.3251
0.3252
0.3252
0.3252
0.3253
0.3253
0.3256

0.3256
0.3256
0.3258
0.3263
0.3263
0.3264
0.3264
0.3264
0.3264
0.3265
0.3268
0.3268
0.3269
0.3269
0.3269
0.3269
0.3269
0.3270
0.3270
0.3273
0.3273
0.3273
0.3275
0.3275
0.3275
0.3278
0.3278
0.3278
0.3283
0.3285
0.3286
0.3286
0.3289
0.3289
0.3289
0.3289
0.3290
0.3290
0.3291
0.3291
0.3291
0.3291
0.3291
0.3291
0.3291
0.3292
0.3294
0.3295
0.3295
0.3296

0.3297
0.3297
0.3302
0.3302
0.3302
0.3302
0.3302
0.3303
0.3303
0.3303
0.3304
0.3306
0.3308
0.3309
0.3309
0.3309
0.3311
0.3311
0.3313
0.3315
0.3315
0.3315
0.3315
0.3316
0.3317
0.3319
0.3320
0.3322
0.3322
0.3322
0.3324
0.3326
0.3327
0.3327
0.3327
0.3327
0.3327
0.3328
0.3328
0.3328
0.3328
0.3328
0.3328
0.3328
0.3330
0.3330
0.3331
0.3331
0.3331
0.3331

0.3331
0.3331
0.3335
0.3339
0.3339
0.3339
0.3339
0.3341
0.3341
0.3342
0.3342
0.3342
0.3342
0.3342
0.3342
0.3347
0.3347
0.3347
0.3347
0.3347
0.3347
0.3347
0.3347
0.3347
0.3348
0.3349
0.3349
0.3349
0.3349
0.3349
0.3349
0.3351
0.3351
0.3356
0.3357
0.3357
0.3358
0.3358
0.3358
0.3358
0.3360
0.3361
0.3362
0.3364
0.3366
0.3366
0.3366
0.3366
0.3366
0.3366

0.3366
0.3366
0.3366
0.3369
0.3370
0.3373
0.3377
0.3377
0.3379
0.3379
0.3379
0.3379
0.3379
0.3379
0.3379
0.3379
0.3379
0.3379
0.3379
0.3379
0.3379
0.3379
0.3379
0.3379
0.3381
0.3381
0.3381
0.3381
0.3381
0.3382
0.3385
0.3387
0.3387
0.3387
0.3388
0.3388
0.3388
0.3388
0.3390
0.3390
0.3390
0.3396
0.3396
0.3399
0.3401
0.3401
0.3402
0.3402
0.3402
0.3402

0.3402
0.3402
0.3402
0.3402
0.3402
0.3402
0.3403
0.3405
0.3405
0.3409
0.3411
0.3411
0.3412
0.3414
0.3415
0.3415
0.3415
0.3415
0.3415
0.3416
0.3416
0.3416
0.3419
0.3419
0.3419
0.3419
0.3419
0.3422
0.3424
0.3432
0.3433
0.3433
0.3433
0.3433
0.3434
0.3434
0.3435
0.3435
0.3435
0.3435
0.3435
0.3435
0.3435
0.3435
0.3435
0.3435
0.3435
0.3435
0.3436
0.3437

0.3437
0.3438
0.3439
0.3439
0.3442
0.3445
0.3445
0.3446
0.3447
0.3447
0.3447
0.3447
0.3447
0.3452
0.3453
0.3454
0.3455
0.3455
0.3455
0.3455
0.3455
0.3458
0.3458
0.3458
0.3458
0.3458
0.3458
0.3459
0.3462
0.3462
0.3462
0.3465
0.3465
0.3465
0.3466
0.3468
0.3468
0.3468
0.3469
0.3469
0.3469
0.3471
0.3472
0.3472
0.3472
0.3472
0.3472
0.3472
0.3472
0.3472

0.3477
0.3477
0.3479
0.3480
0.3480
0.3482
0.3484
0.3485
0.3485
0.3485
0.3485
0.3485
0.3486
0.3486
0.3486
0.3486
0.3487
0.3489
0.3490
0.3494
0.3495
0.3496
0.3496
0.3498
0.3500
0.3500
0.3500
0.3500
0.3500
0.3500
0.3500
0.3500
0.3500
0.3501
0.3503
0.3504
0.3504
0.3504
0.3505
0.3505
0.3506
0.3506
0.3507
0.3508
0.3509
0.3509
0.3512
0.3518
0.3518
0.3518

0.3520
0.3520
0.3520
0.3520
0.3522
0.3524
0.3524
0.3524
0.3524
0.3524
0.3527
0.3527
0.3527
0.3527
0.3527
0.3529
0.3529
0.3533
0.3533
0.3533
0.3533
0.3533
0.3533
0.3533
0.3534
0.3535
0.3537
0.3537
0.3538
0.3538
0.3538
0.3538
0.3538
0.3538
0.3543
0.3548
0.3549
0.3549
0.3549
0.3549
0.3549
0.3549
0.3549
0.3549
0.3550
0.3552
0.3552
0.3557
0.3558
0.3558

0.3559
0.3560
0.3560
0.3560
0.3565
0.3565
0.3566
0.3567
0.3567
0.3567
0.3567
0.3567
0.3568
0.3568
0.3569
0.3569
0.3572
0.3572
0.3572
0.3574
0.3574
0.3574
0.3574
0.3574
0.3575
0.3575
0.3575
0.3577
0.3577
0.3577
0.3581
0.3581
0.3581
0.3581
0.3581
0.3581
0.3581
0.3581
0.3581
0.3581
0.3581
0.3583
0.3583
0.3585
0.3585
0.3585
0.3585
0.3585
0.3585
0.3587

0.3589
0.3590
0.3590
0.3590
0.3590
0.3590
0.3590
0.3593
0.3593
0.3593
0.3593
0.3593
0.3594
0.3594
0.3597
0.3600
0.3600
0.3601
0.3603
0.3605
0.3606
0.3610
0.3611
0.3611
0.3611
0.3611
0.3611
0.3612
0.3614
0.3614
0.3615
0.3615
0.3615
0.3617
0.3617
0.3617
0.3617
0.3620
0.3623
0.3623
0.3623
0.3623
0.3623
0.3623
0.3623
0.3623
0.3623
0.3625
0.3625
0.3625

0.3625
0.3625
0.3626
0.3626
0.3626
0.3631
0.3634
0.3640
0.3643
0.3644
0.3644
0.3644
0.3645
0.3645
0.3645
0.3645
0.3645
0.3645
0.3645
0.3646
0.3646
0.3646
0.3648
0.3648
0.3649
0.3649
0.3651
0.3652
0.3652
0.3652
0.3652
0.3654
0.3654
0.3655
0.3655
0.3656
0.3657
0.3658
0.3659
0.3659
0.3659
0.3659
0.3659
0.3659
0.3660
0.3660
0.3660
0.3661
0.3661
0.3663

0.3663
0.3663
0.3663
0.3665
0.3665
0.3666
0.3666
0.3666
0.3667
0.3667
0.3667
0.3667
0.3669
0.3673
0.3675
0.3675
0.3675
0.3675
0.3676
0.3677
0.3677
0.3677
0.3680
0.3680
0.3680
0.3681
0.3681
0.3682
0.3685
0.3686
0.3686
0.3687
0.3687
0.3687
0.3687
0.3689
0.3689
0.3689
0.3689
0.3695
0.3701
0.3703
0.3705
0.3705
0.3705
0.3705
0.3705
0.3705
0.3705
0.3705

0.3705
0.3706
0.3706
0.3708
0.3708
0.3710
0.3710
0.3710
0.3710
0.3711
0.3713
0.3714
0.3714
0.3714
0.3714
0.3714
0.3714
0.3714
0.3716
0.3718
0.3719
0.3722
0.3722
0.3726
0.3726
0.3726
0.3726
0.3726
0.3727
0.3730
0.3730
0.3732
0.3734
0.3736
0.3736
0.3737
0.3737
0.3737
0.3737
0.3737
0.3738
0.3738
0.3740
0.3740
0.3741
0.3741
0.3742
0.3742
0.3747
0.3747

0.3747
0.3747
0.3747
0.3747
0.3747
0.3747
0.3747
0.3748
0.3749
0.3750
0.3750
0.3750
0.3750
0.3750
0.3750
0.3751
0.3755
0.3756
0.3756
0.3756
0.3756
0.3756
0.3757
0.3757
0.3758
0.3759
0.3759
0.3759
0.3759
0.3760
0.3760
0.3760
0.3762
0.3763
0.3763
0.3763
0.3765
0.3765
0.3767
0.3767
0.3767
0.3768
0.3768
0.3768
0.3768
0.3771
0.3773
0.3774
0.3774
0.3774

0.3774
0.3774
0.3774
0.3774
0.3776
0.3779
0.3779
0.3779
0.3779
0.3780
0.3780
0.3780
0.3782
0.3783
0.3783
0.3783
0.3783
0.3783
0.3783
0.3783
0.3785
0.3786
0.3786
0.3786
0.3789
0.3789
0.3791
0.3794
0.3794
0.3794
0.3795
0.3795
0.3796
0.3799
0.3801
0.3803
0.3804
0.3804
0.3805
0.3805
0.3805
0.3805
0.3805
0.3805
0.3806
0.3806
0.3807
0.3808
0.3809
0.3809

0.3809
0.3809
0.3812
0.3812
0.3812
0.3814
0.3814
0.3814
0.3815
0.3815
0.3815
0.3815
0.3815
0.3815
0.3818
0.3818
0.3818
0.3819
0.3819
0.3819
0.3823
0.3823
0.3823
0.3823
0.3823
0.3824
0.3824
0.3824
0.3824
0.3825
0.3827
0.3827
0.3827
0.3827
0.3827
0.3828
0.3828
0.3828
0.3830
0.3830
0.3830
0.3830
0.3830
0.3832
0.3833
0.3834
0.3835
0.3836
0.3837
0.3837

0.3837
0.3839
0.3839
0.3840
0.3840
0.3840
0.3840
0.3840
0.3840
0.3840
0.3841
0.3841
0.3841
0.3841
0.3841
0.3841
0.3841
0.3842
0.3842
0.3843
0.3845
0.3845
0.3847
0.3847
0.3848
0.3849
0.3852
0.3852
0.3852
0.3852
0.3855
0.3855
0.3856
0.3856
0.3857
0.3857
0.3858
0.3859
0.3859
0.3861
0.3861
0.3863
0.3863
0.3865
0.3867
0.3867
0.3867
0.3870
0.3872
0.3874

0.3875
0.3875
0.3877
0.3879
0.3879
0.3879
0.3879
0.3879
0.3879
0.3879
0.3881
0.3881
0.3881
0.3883
0.3887
0.3887
0.3887
0.3888
0.3889
0.3891
0.3892
0.3892
0.3894
0.3894
0.3894
0.3894
0.3896
0.3901
0.3901
0.3901
0.3901
0.3901
0.3901
0.3902
0.3902
0.3902
0.3906
0.3907
0.3907
0.3910
0.3910
0.3910
0.3910
0.3910
0.3910
0.3910
0.3910
0.3911
0.3912
0.3912

0.3912
0.3912
0.3912
0.3913
0.3913
0.3913
0.3913
0.3913
0.3913
0.3913
0.3913
0.3914
0.3914
0.3914
0.3915
0.3915
0.3915
0.3915
0.3915
0.3915
0.3915
0.3916
0.3916
0.3916
0.3916
0.3918
0.3918
0.3918
0.3918
0.3918
0.3918
0.3919
0.3920
0.3921
0.3922
0.3922
0.3922
0.3922
0.3922
0.3922
0.3923
0.3925
0.3925
0.3928
0.3928
0.3932
0.3932
0.3932
0.3932
0.3932

0.3932
0.3932
0.3932
0.3932
0.3932
0.3932
0.3932
0.3932
0.3932
0.3933
0.3936
0.3942
0.3942
0.3944
0.3944
0.3945
0.3945
0.3945
0.3945
0.3945
0.3946
0.3946
0.3949
0.3950
0.3953
0.3954
0.3955
0.3958
0.3960
0.3960
0.3964
0.3964
0.3966
0.3966
0.3969
0.3969
0.3969
0.3971
0.3972
0.3972
0.3973
0.3973
0.3974
0.3974
0.3974
0.3976
0.3976
0.3976
0.3976
0.3977

0.3977
0.3977
0.3977
0.3977
0.3977
0.3977
0.3977
0.3977
0.3977
0.3977
0.3977
0.3977
0.3977
0.3977
0.3981
0.3981
0.3981
0.3981
0.3981
0.3987
0.3987
0.3987
0.3987
0.3987
0.3987
0.3987
0.3987
0.3989
0.3989
0.3989
0.3989
0.3989
0.3990
0.3990
0.3990
0.3991
0.3992
0.3992
0.3992
0.3992
0.3992
0.3992
0.3992
0.3992
0.3992
0.3996
0.3996
0.3996
0.3996
0.3997

0.4000
0.4000
0.4000
0.4001
0.4003
0.4004
0.4005
0.4009
0.4009
0.4009
0.4009
0.4009
0.4009
0.4010
0.4010
0.4013
0.4013
0.4013
0.4013
0.4014
0.4018
0.4018
0.4023
0.4023
0.4023
0.4023
0.4023
0.4023
0.4023
0.4023
0.4023
0.4023
0.4023
0.4023
0.4024
0.4024
0.4024
0.4024
0.4024
0.4024
0.4024
0.4024
0.4024
0.4024
0.4024
0.4024
0.4024
0.4028
0.4029
0.4029

0.4029
0.4029
0.4030
0.4030
0.4035
0.4035
0.4035
0.4035
0.4035
0.4035
0.4035
0.4035
0.4035
0.4035
0.4035
0.4035
0.4035
0.4035
0.4035
0.4035
0.4035
0.4041
0.4041
0.4041
0.4041
0.4041
0.4041
0.4041
0.4043
0.4043
0.4047
0.4047
0.4047
0.4048
0.4048
0.4048
0.4048
0.4048
0.4048
0.4050
0.4050
0.4050
0.4050
0.4053
0.4053
0.4055
0.4058
0.4060
0.4061
0.4063

0.4063
0.4064
0.4064
0.4064
0.4064
0.4064
0.4064
0.4064
0.4064
0.4064
0.4065
0.4070
0.4070
0.4070
0.4070
0.4070
0.4072
0.4072
0.4072
0.4073
0.4073
0.4073
0.4073
0.4073
0.4073
0.4073
0.4073
0.4073
0.4073
0.4073
0.4073
0.4073
0.4073
0.4073
0.4073
0.4073
0.4073
0.4074
0.4074
0.4074
0.4074
0.4076
0.4076
0.4076
0.4076
0.4079
0.4080
0.4080
0.4082
0.4082

0.4083
0.4083
0.4085
0.4086
0.4087
0.4087
0.4087
0.4088
0.4088
0.4089
0.4092
0.4092
0.4092
0.4092
0.4092
0.4096
0.4097
0.4097
0.4099
0.4103
0.4103
0.4108
0.4108
0.4108
0.4108
0.4110
0.4110
0.4110
0.4110
0.4112
0.4112
0.4112
0.4113
0.4115
0.4115
0.4115
0.4115
0.4115
0.4115
0.4115
0.4115
0.4115
0.4115
0.4115
0.4115
0.4115
0.4118
0.4119
0.4121
0.4121

0.4122
0.4122
0.4122
0.4123
0.4123
0.4123
0.4123
0.4127
0.4128
0.4128
0.4129
0.4133
0.4133
0.4133
0.4136
0.4141
0.4141
0.4142
0.4143
0.4144
0.4146
0.4146
0.4146
0.4146
0.4146
0.4146
0.4148
0.4150
0.4150
0.4150
0.4150
0.4150
0.4150
0.4155
0.4155
0.4155
0.4155
0.4155
0.4155
0.4156
0.4156
0.4156
0.4156
0.4156
0.4161
0.4161
0.4162
0.4162
0.4163
0.4165

0.4165
0.4165
0.4168
0.4168
0.4168
0.4170
0.4170
0.4170
0.4172
0.4172
0.4173
0.4173
0.4173
0.4175
0.4175
0.4176
0.4177
0.4178
0.4180
0.4180
0.4183
0.4183
0.4183
0.4183
0.4183
0.4183
0.4184
0.4184
0.4184
0.4184
0.4184
0.4184
0.4184
0.4184
0.4184
0.4184
0.4187
0.4188
0.4189
0.4189
0.4189
0.4189
0.4191
0.4193
0.4193
0.4193
0.4195
0.4195
0.4197
0.4198

0.4199
0.4199
0.4199
0.4199
0.4199
0.4204
0.4206
0.4206
0.4206
0.4206
0.4206
0.4206
0.4206
0.4206
0.4207
0.4210
0.4212
0.4212
0.4213
0.4214
0.4217
0.4217
0.4220
0.4220
0.4222
0.4222
0.4223
0.4223
0.4226
0.4227
0.4228
0.4229
0.4229
0.4229
0.4229
0.4229
0.4229
0.4229
0.4229
0.4229
0.4229
0.4229
0.4229
0.4229
0.4229
0.4229
0.4229
0.4229
0.4229
0.4229

0.4229
0.4233
0.4234
0.4237
0.4237
0.4237
0.4238
0.4238
0.4240
0.4241
0.4241
0.4243
0.4243
0.4244
0.4244
0.4244
0.4256
0.4256
0.4259
0.4260
0.4260
0.4260
0.4260
0.4260
0.4260
0.4263
0.4263
0.4263
0.4263
0.4263
0.4263
0.4263
0.4263
0.4265
0.4267
0.4271
0.4272
0.4272
0.4272
0.4272
0.4272
0.4272
0.4272
0.4274
0.4274
0.4274
0.4274
0.4275
0.4277
0.4281

0.4281
0.4281
0.4281
0.4284
0.4285
0.4286
0.4286
0.4286
0.4286
0.4286
0.4286
0.4288
0.4288
0.4288
0.4288
0.4289
0.4290
0.4294
0.4294
0.4294
0.4294
0.4299
0.4301
0.4301
0.4301
0.4301
0.4302
0.4302
0.4303
0.4303
0.4303
0.4303
0.4303
0.4303
0.4303
0.4303
0.4303
0.4303
0.4303
0.4303
0.4303
0.4303
0.4303
0.4304
0.4304
0.4305
0.4305
0.4305
0.4305
0.4305

0.4306
0.4309
0.4309
0.4309
0.4309
0.4309
0.4309
0.4309
0.4309
0.4309
0.4309
0.4309
0.4309
0.4309
0.4309
0.4312
0.4312
0.4312
0.4312
0.4312
0.4312
0.4313
0.4313
0.4313
0.4313
0.4313
0.4313
0.4313
0.4313
0.4313
0.4313
0.4313
0.4313
0.4313
0.4313
0.4313
0.4313
0.4313
0.4313
0.4313
0.4313
0.4313
0.4313
0.4313
0.4315
0.4315
0.4317
0.4317
0.4317
0.4317

0.4317
0.4317
0.4317
0.4319
0.4319
0.4319
0.4319
0.4319
0.4323
0.4323
0.4323
0.4324
0.4328
0.4328
0.4328
0.4328
0.4328
0.4329
0.4330
0.4330
0.4331
0.4331
0.4331
0.4331
0.4331
0.4331
0.4339
0.4340
0.4340
0.4341
0.4341
0.4342
0.4343
0.4346
0.4348
0.4348
0.4350
0.4351
0.4354
0.4354
0.4358
0.4358
0.4359
0.4363
0.4366
0.4366
0.4366
0.4366
0.4367
0.4369

0.4370
0.4372
0.4374
0.4375
0.4375
0.4375
0.4375
0.4375
0.4375
0.4377
0.4377
0.4377
0.4379
0.4380
0.4380
0.4380
0.4380
0.4382
0.4383
0.4384
0.4384
0.4384
0.4384
0.4386
0.4387
0.4387
0.4387
0.4388
0.4393
0.4393
0.4393
0.4394
0.4395
0.4395
0.4395
0.4395
0.4395
0.4395
0.4395
0.4396
0.4398
0.4398
0.4398
0.4398
0.4398
0.4398
0.4400
0.4401
0.4401
0.4404

0.4404
0.4404
0.4404
0.4404
0.4404
0.4405
0.4407
0.4407
0.4413
0.4413
0.4413
0.4413
0.4414
0.4414
0.4414
0.4415
0.4416
0.4416
0.4416
0.4416
0.4416
0.4422
0.4422
0.4422
0.4422
0.4422
0.4422
0.4422
0.4422
0.4422
0.4422
0.4423
0.4427
0.4427
0.4428
0.4429
0.4429
0.4429
0.4429
0.4430
0.4430
0.4431
0.4432
0.4436
0.4438
0.4438
0.4438
0.4439
0.4439
0.4439

0.4439
0.4439
0.4439
0.4440
0.4444
0.4444
0.4448
0.4449
0.4450
0.4451
0.4456
0.4456
0.4459
0.4459
0.4459
0.4460
0.4460
0.4460
0.4460
0.4461
0.4461
0.4461
0.4461
0.4461
0.4461
0.4461
0.4462
0.4463
0.4464
0.4464
0.4467
0.4467
0.4467
0.4467
0.4467
0.4467
0.4467
0.4468
0.4468
0.4469
0.4469
0.4471
0.4471
0.4471
0.4472
0.4475
0.4475
0.4475
0.4476
0.4476

0.4476
0.4478
0.4479
0.4481
0.4482
0.4482
0.4482
0.4484
0.4487
0.4487
0.4487
0.4487
0.4488
0.4493
0.4493
0.4493
0.4493
0.4493
0.4493
0.4493
0.4493
0.4498
0.4498
0.4498
0.4498
0.4498
0.4499
0.4499
0.4499
0.4499
0.4499
0.4499
0.4499
0.4499
0.4499
0.4499
0.4499
0.4499
0.4499
0.4499
0.4499
0.4499
0.4499
0.4499
0.4499
0.4499
0.4499
0.4499
0.4501
0.4501

0.4501
0.4501
0.4501
0.4503
0.4503
0.4503
0.4503
0.4503
0.4503
0.4505
0.4505
0.4505
0.4505
0.4505
0.4505
0.4505
0.4505
0.4505
0.4505
0.4505
0.4505
0.4505
0.4505
0.4505
0.4505
0.4505
0.4505
0.4505
0.4508
0.4508
0.4508
0.4508
0.4508
0.4509
0.4509
0.4509
0.4510
0.4510
0.4512
0.4512
0.4512
0.4512
0.4512
0.4512
0.4512
0.4512
0.4512
0.4512
0.4512
0.4512
0.4512

0.4513
0.4513
0.4513
0.4513
0.4513
0.4513
0.4513
0.4513
0.4513
0.4513
0.4513
0.4513
0.4513
0.4513
0.4515
0.4515
0.4515
0.4515
0.4515
0.4515
0.4515
0.4515
0.4517
0.4517
0.4517
0.4518
0.4518
0.4518
0.4518
0.4518
0.4520
0.4520
0.4520
0.4521
0.4521
0.4521
0.4526
0.4526
0.4526
0.4529
0.4529
0.4529
0.4529
0.4529
0.4529
0.4531
0.4532
0.4532
0.4532
0.4532

0.4532
0.4532
0.4532
0.4532
0.4532
0.4533
0.4533
0.4533
0.4533
0.4536
0.4538
0.4539
0.4540
0.4541
0.4541
0.4545
0.4545
0.4547
0.4549
0.4549
0.4549
0.4549
0.4549
0.4549
0.4551
0.4553
0.4555
0.4555
0.4555
0.4557
0.4557
0.4557
0.4557
0.4557
0.4558
0.4558
0.4558
0.4560
0.4561
0.4564
0.4564
0.4564
0.4564
0.4564
0.4564
0.4564
0.4564
0.4564
0.4564
0.4567

0.4567
0.4567
0.4567
0.4567
0.4567
0.4568
0.4569
0.4570
0.4570
0.4570
0.4572
0.4574
0.4574
0.4577
0.4579
0.4579
0.4580
0.4581
0.4582
0.4583
0.4583
0.4584
0.4584
0.4584
0.4584
0.4589
0.4590
0.4590
0.4590
0.4590
0.4590
0.4593
0.4594
0.4596
0.4597
0.4598
0.4598
0.4600
0.4600
0.4600
0.4600
0.4600
0.4602
0.4602
0.4604
0.4605
0.4605
0.4605
0.4605
0.4605

0.4605
0.4605
0.4605
0.4605
0.4605
0.4605
0.4605
0.4605
0.4605
0.4605
0.4605
0.4607
0.4607
0.4611
0.4611
0.4611
0.4612
0.4612
0.4612
0.4612
0.4612
0.4612
0.4614
0.4615
0.4615
0.4615
0.4615
0.4615
0.4615
0.4615
0.4615
0.4615
0.4615
0.4615
0.4616
0.4619
0.4621
0.4621
0.4622
0.4622
0.4622
0.4622
0.4622
0.4626
0.4626
0.4628
0.4628
0.4629
0.4630
0.4630

0.4630
0.4632
0.4632
0.4632
0.4632
0.4632
0.4632
0.4638
0.4638
0.4638
0.4638
0.4638
0.4638
0.4638
0.4639
0.4639
0.4641
0.4641
0.4643
0.4643
0.4644
0.4648
0.4650
0.4650
0.4653
0.4653
0.4655
0.4655
0.4655
0.4655
0.4655
0.4655
0.4656
0.4659
0.4659
0.4659
0.4659
0.4659
0.4662
0.4664
0.4664
0.4665
0.4665
0.4665
0.4665
0.4665
0.4665
0.4665
0.4665
0.4665

0.4665
0.4665
0.4668
0.4668
0.4668
0.4668
0.4668
0.4668
0.4669
0.4671
0.4674
0.4678
0.4682
0.4684
0.4686
0.4686
0.4687
0.4687
0.4687
0.4687
0.4687
0.4687
0.4688
0.4688
0.4689
0.4689
0.4690
0.4695
0.4695
0.4695
0.4695
0.4695
0.4696
0.4697
0.4697
0.4697
0.4697
0.4697
0.4698
0.4700
0.4700
0.4700
0.4700
0.4702
0.4702
0.4705
0.4705
0.4705
0.4705
0.4705

0.4705
0.4705
0.4705
0.4705
0.4705
0.4705
0.4705
0.4705
0.4705
0.4705
0.4705
0.4705
0.4705
0.4706
0.4706
0.4708
0.4708
0.4708
0.4709
0.4710
0.4711
0.4711
0.4712
0.4713
0.4714
0.4714
0.4714
0.4714
0.4714
0.4714
0.4714
0.4714
0.4714
0.4717
0.4717
0.4718
0.4722
0.4722
0.4722
0.4722
0.4722
0.4722
0.4722
0.4722
0.4722
0.4722
0.4722
0.4722
0.4722
0.4724

0.4726
0.4726
0.4726
0.4726
0.4726
0.4726
0.4727
0.4727
0.4727
0.4727
0.4727
0.4727
0.4727
0.4727
0.4728
0.4730
0.4730
0.4730
0.4731
0.4731
0.4732
0.4735
0.4736
0.4738
0.4738
0.4738
0.4741
0.4741
0.4741
0.4741
0.4741
0.4741
0.4741
0.4741
0.4743
0.4743
0.4744
0.4744
0.4746
0.4746
0.4746
0.4746
0.4746
0.4746
0.4748
0.4748
0.4748
0.4748
0.4748
0.4748

0.4748
0.4748
0.4749
0.4749
0.4751
0.4751
0.4752
0.4757
0.4757
0.4757
0.4757
0.4757
0.4757
0.4759
0.4759
0.4759
0.4763
0.4763
0.4763
0.4768
0.4768
0.4768
0.4768
0.4768
0.4769
0.4769
0.4769
0.4771
0.4771
0.4778
0.4778
0.4779
0.4779
0.4779
0.4779
0.4779
0.4779
0.4780
0.4781
0.4781
0.4781
0.4782
0.4782
0.4782
0.4782
0.4782
0.4784
0.4785
0.4787
0.4787

0.4792
0.4792
0.4792
0.4792
0.4792
0.4792
0.4794
0.4794
0.4794
0.4799
0.4800
0.4800
0.4802
0.4803
0.4803
0.4803
0.4805
0.4808
0.4808
0.4808
0.4808
0.4808
0.4808
0.4808
0.4810
0.4811
0.4811
0.4812
0.4812
0.4815
0.4815
0.4815
0.4815
0.4820
0.4820
0.4827
0.4827
0.4828
0.4829
0.4829
0.4831
0.4832
0.4832
0.4832
0.4833
0.4833
0.4833
0.4833
0.4833
0.4833

0.4833
0.4833
0.4833
0.4833
0.4833
0.4833
0.4834
0.4834
0.4834
0.4834
0.4834
0.4835
0.4835
0.4835
0.4835
0.4835
0.4835
0.4835
0.4837
0.4837
0.4838
0.4841
0.4841
0.4842
0.4842
0.4847
0.4847
0.4847
0.4847
0.4847
0.4847
0.4847
0.4847
0.4847
0.4847
0.4847
0.4848
0.4855
0.4857
0.4857
0.4857
0.4859
0.4859
0.4859
0.4860
0.4861
0.4861
0.4862
0.4863
0.4864

0.4864
0.4864
0.4864
0.4864
0.4865
0.4866
0.4866
0.4868
0.4868
0.4868
0.4868
0.4868
0.4868
0.4868
0.4868
0.4868
0.4868
0.4868
0.4868
0.4869
0.4872
0.4873
0.4874
0.4874
0.4875
0.4875
0.4875
0.4875
0.4875
0.4875
0.4878
0.4878
0.4878
0.4881
0.4881
0.4881
0.4881
0.4881
0.4882
0.4882
0.4882
0.4882
0.4883
0.4884
0.4884
0.4884
0.4888
0.4888
0.4888
0.4888

0.4888
0.4892
0.4892
0.4892
0.4895
0.4896
0.4896
0.4902
0.4902
0.4904
0.4904
0.4904
0.4904
0.4904
0.4904
0.4905
0.4905
0.4905
0.4907
0.4907
0.4912
0.4912
0.4912
0.4912
0.4912
0.4918
0.4921
0.4922
0.4923
0.4925
0.4926
0.4927
0.4932
0.4932
0.4932
0.4932
0.4932
0.4932
0.4932
0.4932
0.4932
0.4932
0.4932
0.4932
0.4932
0.4932
0.4932
0.4933
0.4934
0.4935

0.4936
0.4936
0.4936
0.4937
0.4937
0.4940
0.4941
0.4942
0.4945
0.4945
0.4947
0.4947
0.4947
0.4948
0.4950
0.4950
0.4953
0.4955
0.4956
0.4957
0.4957
0.4959
0.4959
0.4961
0.4963
0.4964
0.4968
0.4968
0.4970
0.4971
0.4972
0.4974
0.4975
0.4978
0.4981
0.4981
0.4981
0.4981
0.4981
0.4982
0.4982
0.4982
0.4983
0.4983
0.4983
0.4983
0.4983
0.4985
0.4985
0.4985

0.4985
0.4985
0.4985
0.4985
0.4985
0.4985
0.4985
0.4985
0.4985
0.4987
0.4987
0.4987
0.4987
0.4988
0.4988
0.4988
0.4988
0.4988
0.4988
0.4988
0.4988
0.4988
0.4988
0.4988
0.4988
0.4988
0.4988
0.4988
0.4988
0.4989
0.4989
0.4991
0.4992
0.4992
0.4993
0.4994
0.4997
0.4997
0.4997
0.4997
0.5000
0.5000
0.5000
0.5000
0.5000
0.5000
0.5000
0.5000
0.5000
0.5000

0.5000
0.5000
0.5000
0.5000
0.5000
0.5001
0.5001
0.5001
0.5001
0.5001
0.5001
0.5001
0.5001
0.5001
0.5004
0.5004
0.5010
0.5012
0.5012
0.5012
0.5015
0.5015
0.5015
0.5015
0.5015
0.5015
0.5015
0.5016
0.5016
0.5018
0.5020
0.5022
0.5022
0.5023
0.5023
0.5024
0.5024
0.5026
0.5026
0.5026
0.5027
0.5027
0.5028
0.5028
0.5028
0.5029
0.5029
0.5029
0.5029
0.5030

0.5030
0.5030
0.5033
0.5035
0.5035
0.5035
0.5038
0.5038
0.5038
0.5039
0.5039
0.5040
0.5042
0.5042
0.5042
0.5044
0.5044
0.5044
0.5044
0.5048
0.5048
0.5050
0.5051
0.5055
0.5055
0.5057
0.5058
0.5058
0.5060
0.5060
0.5060
0.5061
0.5061
0.5062
0.5062
0.5062
0.5062
0.5062
0.5062
0.5062
0.5062
0.5062
0.5062
0.5062
0.5062
0.5062
0.5062
0.5062
0.5062
0.5064

0.5067
0.5068
0.5071
0.5074
0.5077
0.5077
0.5077
0.5077
0.5079
0.5079
0.5079
0.5080
0.5082
0.5082
0.5083
0.5087
0.5087
0.5089
0.5090
0.5090
0.5090
0.5091
0.5091
0.5092
0.5092
0.5092
0.5092
0.5092
0.5093
0.5093
0.5093
0.5096
0.5096
0.5096
0.5096
0.5096
0.5096
0.5096
0.5096
0.5096
0.5096
0.5096
0.5096
0.5096
0.5096
0.5098
0.5099
0.5099
0.5099
0.5099

0.5099
0.5099
0.5099
0.5099
0.5100
0.5100
0.5102
0.5102
0.5102
0.5102
0.5102
0.5106
0.5106
0.5107
0.5107
0.5107
0.5107
0.5107
0.5107
0.5110
0.5110
0.5112
0.5114
0.5114
0.5114
0.5114
0.5114
0.5115
0.5115
0.5115
0.5115
0.5115
0.5119
0.5119
0.5119
0.5119
0.5124
0.5124
0.5124
0.5126
0.5132
0.5134
0.5134
0.5134
0.5145
0.5145
0.5145
0.5146
0.5146
0.5146

0.5148
0.5148
0.5148
0.5148
0.5151
0.5151
0.5155
0.5155
0.5155
0.5157
0.5159
0.5159
0.5159
0.5159
0.5159
0.5164
0.5165
0.5166
0.5166
0.5167
0.5167
0.5167
0.5167
0.5167
0.5168
0.5170
0.5171
0.5176
0.5177
0.5178
0.5179
0.5179
0.5179
0.5180
0.5180
0.5180
0.5180
0.5180
0.5180
0.5180
0.5180
0.5180
0.5180
0.5180
0.5181
0.5182
0.5182
0.5182
0.5187
0.5187

0.5187
0.5187
0.5188
0.5188
0.5189
0.5192
0.5193
0.5193
0.5193
0.5193
0.5193
0.5195
0.5195
0.5195
0.5196
0.5196
0.5196
0.5196
0.5196
0.5196
0.5196
0.5196
0.5196
0.5196
0.5198
0.5199
0.5199
0.5199
0.5201
0.5201
0.5203
0.5203
0.5203
0.5204
0.5208
0.5209
0.5214
0.5214
0.5214
0.5218
0.5218
0.5218
0.5218
0.5218
0.5218
0.5218
0.5218
0.5218
0.5218
0.5218

0.5218
0.5218
0.5218
0.5218
0.5218
0.5218
0.5218
0.5219
0.5219
0.5221
0.5221
0.5221
0.5221
0.5224
0.5225
0.5227
0.5227
0.5229
0.5230
0.5234
0.5236
0.5236
0.5236
0.5236
0.5236
0.5236
0.5236
0.5236
0.5236
0.5236
0.5236
0.5236
0.5237
0.5237
0.5237
0.5240
0.5240
0.5240
0.5240
0.5240
0.5240
0.5241
0.5243
0.5244
0.5245
0.5248
0.5249
0.5249
0.5249
0.5249

0.5250
0.5251
0.5252
0.5252
0.5253
0.5256
0.5256
0.5258
0.5260
0.5261
0.5261
0.5262
0.5262
0.5263
0.5263
0.5263
0.5263
0.5263
0.5263
0.5263
0.5263
0.5265
0.5266
0.5267
0.5267
0.5267
0.5268
0.5269
0.5269
0.5270
0.5277
0.5277
0.5277
0.5277
0.5277
0.5277
0.5277
0.5278
0.5279
0.5281
0.5281
0.5281
0.5281
0.5281
0.5281
0.5281
0.5285
0.5285
0.5286
0.5286

0.5286
0.5288
0.5288
0.5288
0.5290
0.5290
0.5294
0.5294
0.5297
0.5297
0.5297
0.5297
0.5297
0.5297
0.5297
0.5297
0.5297
0.5297
0.5297
0.5297
0.5297
0.5297
0.5297
0.5297
0.5297
0.5297
0.5297
0.5297
0.5297
0.5297
0.5297
0.5299
0.5300
0.5301
0.5302
0.5306
0.5308
0.5309
0.5310
0.5311
0.5315
0.5315
0.5316
0.5316
0.5316
0.5316
0.5316
0.5316
0.5316
0.5319

0.5319
0.5319
0.5319
0.5319
0.5319
0.5319
0.5319
0.5320
0.5320
0.5320
0.5320
0.5320
0.5320
0.5320
0.5321
0.5321
0.5321
0.5321
0.5321
0.5323
0.5323
0.5323
0.5323
0.5323
0.5323
0.5323
0.5325
0.5326
0.5326
0.5330
0.5332
0.5332
0.5334
0.5337
0.5337
0.5338
0.5340
0.5347
0.5349
0.5351
0.5351
0.5351
0.5351
0.5351
0.5351
0.5351
0.5351
0.5351
0.5351
0.5351

0.5351
0.5351
0.5351
0.5351
0.5354
0.5354
0.5354
0.5355
0.5355
0.5356
0.5356
0.5356
0.5356
0.5357
0.5357
0.5357
0.5360
0.5363
0.5363
0.5364
0.5368
0.5368
0.5368
0.5368
0.5368
0.5368
0.5368
0.5368
0.5368
0.5368
0.5368
0.5372
0.5372
0.5377
0.5378
0.5378
0.5378
0.5378
0.5378
0.5379
0.5381
0.5381
0.5381
0.5381
0.5381
0.5387
0.5387
0.5387
0.5387
0.5388

0.5388
0.5391
0.5391
0.5393
0.5393
0.5393
0.5393
0.5393
0.5393
0.5393
0.5395
0.5395
0.5397
0.5398
0.5399
0.5400
0.5400
0.5403
0.5403
0.5403
0.5407
0.5408
0.5408
0.5408
0.5409
0.5409
0.5409
0.5409
0.5409
0.5411
0.5411
0.5411
0.5413
0.5414
0.5415
0.5419
0.5420
0.5420
0.5420
0.5420
0.5422
0.5425
0.5425
0.5425
0.5425
0.5426
0.5426
0.5427
0.5428
0.5429

0.5429
0.5431
0.5431
0.5432
0.5432
0.5436
0.5436
0.5436
0.5436
0.5436
0.5436
0.5437
0.5438
0.5439
0.5450
0.5450
0.5450
0.5451
0.5453
0.5453
0.5453
0.5453
0.5453
0.5453
0.5455
0.5455
0.5455
0.5455
0.5455
0.5455
0.5455
0.5455
0.5460
0.5462
0.5463
0.5464
0.5464
0.5464
0.5464
0.5464
0.5464
0.5464
0.5465
0.5468
0.5468
0.5468
0.5469
0.5475
0.5475
0.5477

0.5477
0.5477
0.5477
0.5477
0.5477
0.5479
0.5479
0.5479
0.5479
0.5481
0.5481
0.5481
0.5481
0.5483
0.5483
0.5483
0.5485
0.5485
0.5486
0.5486
0.5487
0.5489
0.5489
0.5489
0.5489
0.5489
0.5489
0.5489
0.5489
0.5490
0.5490
0.5494
0.5494
0.5494
0.5494
0.5496
0.5496
0.5499
0.5499
0.5502
0.5505
0.5505
0.5505
0.5505
0.5505
0.5505
0.5508
0.5509
0.5509
0.5509

0.5509
0.5509
0.5509
0.5511
0.5513
0.5513
0.5513
0.5513
0.5513
0.5513
0.5513
0.5513
0.5513
0.5513
0.5513
0.5513
0.5514
0.5515
0.5515
0.5515
0.5515
0.5517
0.5519
0.5519
0.5520
0.5520
0.5521
0.5521
0.5521
0.5521
0.5525
0.5525
0.5526
0.5526
0.5526
0.5526
0.5526
0.5526
0.5526
0.5526
0.5529
0.5531
0.5535
0.5535
0.5535
0.5535
0.5536
0.5536
0.5537
0.5537

0.5538
0.5540
0.5541
0.5543
0.5543
0.5546
0.5546
0.5546
0.5546
0.5546
0.5546
0.5546
0.5546
0.5546
0.5546
0.5546
0.5547
0.5547
0.5547
0.5549
0.5549
0.5549
0.5550
0.5550
0.5550
0.5550
0.5550
0.5550
0.5550
0.5551
0.5551
0.5551
0.5551
0.5551
0.5551
0.5551
0.5551
0.5551
0.5551
0.5551
0.5551
0.5551
0.5551
0.5555
0.5555
0.5556
0.5559
0.5560
0.5563
0.5564

0.5564
0.5564
0.5564
0.5564
0.5564
0.5564
0.5564
0.5564
0.5564
0.5564
0.5564
0.5564
0.5566
0.5566
0.5566
0.5566
0.5566
0.5567
0.5567
0.5568
0.5570
0.5570
0.5571
0.5572
0.5572
0.5577
0.5580
0.5581
0.5582
0.5583
0.5585
0.5585
0.5585
0.5585
0.5586
0.5586
0.5590
0.5593
0.5594
0.5594
0.5594
0.5594
0.5595
0.5595
0.5595
0.5595
0.5595
0.5595
0.5595
0.5595

0.5595
0.5595
0.5595
0.5598
0.5598
0.5599
0.5602
0.5602
0.5602
0.5602
0.5602
0.5602
0.5602
0.5602
0.5602
0.5602
0.5602
0.5604
0.5604
0.5604
0.5605
0.5608
0.5612
0.5613
0.5613
0.5613
0.5613
0.5613
0.5613
0.5613
0.5613
0.5613
0.5613
0.5613
0.5613
0.5613
0.5613
0.5613
0.5613
0.5613
0.5613
0.5613
0.5613
0.5613
0.5613
0.5613
0.5613
0.5613
0.5613

0.5613
0.5613
0.5613
0.5613
0.5613
0.5613
0.5613
0.5614
0.5614
0.5614
0.5615
0.5615
0.5616
0.5616
0.5616
0.5616
0.5616
0.5616
0.5620
0.5620
0.5621
0.5621
0.5621
0.5621
0.5621
0.5621
0.5623
0.5624
0.5624
0.5624
0.5624
0.5624
0.5624
0.5624
0.5624
0.5628
0.5629
0.5629
0.5629
0.5629
0.5629
0.5630
0.5633
0.5633
0.5633
0.5637
0.5637
0.5637
0.5637
0.5637

0.5637
0.5641
0.5641
0.5642
0.5643
0.5643
0.5643
0.5643
0.5643
0.5644
0.5644
0.5645
0.5645
0.5645
0.5645
0.5645
0.5645
0.5645
0.5652
0.5653
0.5653
0.5654
0.5654
0.5654
0.5654
0.5656
0.5658
0.5658
0.5658
0.5658
0.5663
0.5663
0.5663
0.5663
0.5664
0.5664
0.5665
0.5665
0.5667
0.5667
0.5669
0.5669
0.5671
0.5671
0.5672
0.5672
0.5673
0.5673
0.5673
0.5673

0.5673
0.5676
0.5676
0.5677
0.5677
0.5679
0.5679
0.5679
0.5679
0.5679
0.5679
0.5680
0.5680
0.5685
0.5686
0.5687
0.5687
0.5688
0.5688
0.5689
0.5689
0.5689
0.5689
0.5689
0.5689
0.5689
0.5697
0.5698
0.5701
0.5703
0.5703
0.5706
0.5707
0.5707
0.5712
0.5712
0.5712
0.5717
0.5718
0.5718
0.5718
0.5718
0.5718
0.5718
0.5721
0.5721
0.5721
0.5722
0.5723
0.5729

0.5729
0.5729
0.5730
0.5730
0.5732
0.5732
0.5733
0.5739
0.5740
0.5741
0.5742
0.5742
0.5742
0.5742
0.5742
0.5742
0.5742
0.5742
0.5745
0.5745
0.5745
0.5748
0.5748
0.5749
0.5749
0.5749
0.5752
0.5752
0.5752
0.5754
0.5754
0.5756
0.5757
0.5757
0.5757
0.5757
0.5757
0.5757
0.5757
0.5757
0.5757
0.5757
0.5757
0.5757
0.5757
0.5757
0.5757
0.5757
0.5757
0.5757

0.5757
0.5758
0.5763
0.5763
0.5763
0.5763
0.5763
0.5765
0.5768
0.5768
0.5768
0.5769
0.5769
0.5769
0.5769
0.5769
0.5769
0.5769
0.5769
0.5769
0.5769
0.5769
0.5770
0.5770
0.5770
0.5770
0.5770
0.5772
0.5772
0.5774
0.5774
0.5774
0.5775
0.5775
0.5778
0.5778
0.5779
0.5779
0.5779
0.5779
0.5780
0.5780
0.5780
0.5780
0.5780
0.5780
0.5780
0.5780
0.5780
0.5780

0.5780
0.5781
0.5784
0.5784
0.5790
0.5790
0.5791
0.5793
0.5799
0.5801
0.5804
0.5804
0.5806
0.5806
0.5808
0.5810
0.5810
0.5810
0.5811
0.5814
0.5814
0.5814
0.5814
0.5814
0.5814
0.5814
0.5814
0.5814
0.5814
0.5814
0.5814
0.5815
0.5815
0.5815
0.5816
0.5816
0.5816
0.5816
0.5816
0.5820
0.5820
0.5820
0.5820
0.5820
0.5820
0.5820
0.5822
0.5825
0.5825
0.5825

0.5825
0.5830
0.5831
0.5840
0.5840
0.5840
0.5840
0.5841
0.5841
0.5842
0.5846
0.5846
0.5846
0.5847
0.5849
0.5853
0.5853
0.5854
0.5854
0.5854
0.5856
0.5858
0.5858
0.5860
0.5860
0.5860
0.5863
0.5863
0.5864
0.5864
0.5864
0.5864
0.5864
0.5866
0.5866
0.5868
0.5869
0.5871
0.5871
0.5872
0.5872
0.5872
0.5872
0.5872
0.5872
0.5872
0.5872
0.5872
0.5872
0.5872

0.5872
0.5872
0.5874
0.5875
0.5876
0.5876
0.5876
0.5876
0.5877
0.5877
0.5877
0.5879
0.5879
0.5879
0.5879
0.5879
0.5879
0.5881
0.5883
0.5883
0.5883
0.5884
0.5885
0.5885
0.5886
0.5886
0.5886
0.5886
0.5887
0.5887
0.5890
0.5891
0.5891
0.5891
0.5892
0.5892
0.5895
0.5895
0.5895
0.5895
0.5895
0.5895
0.5895
0.5896
0.5896
0.5902
0.5902
0.5902
0.5902
0.5903

0.5905
0.5905
0.5906
0.5907
0.5909
0.5909
0.5909
0.5909
0.5914
0.5915
0.5915
0.5917
0.5919
0.5919
0.5919
0.5919
0.5919
0.5919
0.5919
0.5919
0.5920
0.5921
0.5921
0.5922
0.5922
0.5924
0.5924
0.5926
0.5926
0.5926
0.5929
0.5929
0.5931
0.5931
0.5932
0.5934
0.5934
0.5934
0.5934
0.5935
0.5935
0.5935
0.5935
0.5935
0.5935
0.5935
0.5935
0.5935
0.5935
0.5935

0.5935
0.5936
0.5937
0.5937
0.5937
0.5937
0.5938
0.5941
0.5942
0.5947
0.5947
0.5951
0.5951
0.5951
0.5956
0.5956
0.5956
0.5956
0.5957
0.5958
0.5958
0.5958
0.5960
0.5961
0.5961
0.5963
0.5963
0.5964
0.5967
0.5967
0.5967
0.5971
0.5976
0.5976
0.5976
0.5978
0.5981
0.5981
0.5981
0.5981
0.5981
0.5983
0.5983
0.5986
0.5988
0.5990
0.5991
0.5992
0.5992
0.5993

0.5993
0.5993
0.5993
0.5993
0.5993
0.5993
0.5993
0.5994
0.5996
0.5997
0.5999
0.5999
0.6003
0.6006
0.6006
0.6014
0.6014
0.6014
0.6014
0.6017
0.6017
0.6018
0.6018
0.6018
0.6018
0.6018
0.6019
0.6019
0.6019
0.6019
0.6020
0.6021
0.6024
0.6025
0.6027
0.6028
0.6028
0.6028
0.6030
0.6031
0.6032
0.6032
0.6034
0.6034
0.6034
0.6035
0.6038
0.6039
0.6039
0.6039

0.6039
0.6039
0.6040
0.6041
0.6045
0.6047
0.6047
0.6047
0.6047
0.6050
0.6050
0.6050
0.6050
0.6050
0.6052
0.6052
0.6053
0.6053
0.6053
0.6053
0.6053
0.6053
0.6053
0.6053
0.6053
0.6054
0.6054
0.6055
0.6055
0.6055
0.6055
0.6056
0.6056
0.6056
0.6056
0.6057
0.6058
0.6058
0.6059
0.6059
0.6059
0.6059
0.6059
0.6061
0.6070
0.6070
0.6074
0.6076
0.6078
0.6078

0.6078
0.6078
0.6078
0.6078
0.6078
0.6080
0.6082
0.6082
0.6085
0.6085
0.6085
0.6085
0.6085
0.6085
0.6085
0.6085
0.6085
0.6085
0.6085
0.6085
0.6085
0.6088
0.6089
0.6090
0.6090
0.6092
0.6092
0.6092
0.6092
0.6092
0.6092
0.6093
0.6096
0.6096
0.6097
0.6097
0.6097
0.6101
0.6103
0.6103
0.6103
0.6103
0.6103
0.6103
0.6103
0.6103
0.6103
0.6104
0.6104
0.6104

0.6104
0.6104
0.6104
0.6104
0.6106
0.6107
0.6109
0.6109
0.6109
0.6119
0.6119
0.6120
0.6120
0.6120
0.6121
0.6121
0.6121
0.6121
0.6124
0.6124
0.6128
0.6129
0.6129
0.6130
0.6132
0.6132
0.6132
0.6132
0.6133
0.6134
0.6134
0.6134
0.6137
0.6137
0.6137
0.6137
0.6137
0.6137
0.6144
0.6144
0.6144
0.6144
0.6144
0.6144
0.6144
0.6144
0.6144
0.6144
0.6144
0.6144

0.6145
0.6146
0.6149
0.6150
0.6150
0.6150
0.6150
0.6152
0.6154
0.6155
0.6155
0.6155
0.6155
0.6155
0.6155
0.6156
0.6156
0.6157
0.6157
0.6157
0.6157
0.6157
0.6157
0.6157
0.6157
0.6157
0.6157
0.6158
0.6158
0.6163
0.6163
0.6163
0.6163
0.6163
0.6166
0.6169
0.6169
0.6170
0.6170
0.6170
0.6170
0.6170
0.6170
0.6170
0.6170
0.6171
0.6173
0.6173
0.6173
0.6173

0.6173
0.6173
0.6173
0.6173
0.6173
0.6173
0.6173
0.6174
0.6174
0.6174
0.6174
0.6174
0.6174
0.6174
0.6174
0.6174
0.6176
0.6176
0.6177
0.6177
0.6177
0.6180
0.6184
0.6184
0.6184
0.6184
0.6185
0.6185
0.6185
0.6186
0.6186
0.6190
0.6191
0.6191
0.6195
0.6197
0.6197
0.6198
0.6199
0.6203
0.6203
0.6203
0.6203
0.6203
0.6203
0.6203
0.6204
0.6204
0.6205
0.6206

0.6206
0.6206
0.6206
0.6206
0.6206
0.6206
0.6206
0.6206
0.6206
0.6206
0.6206
0.6206
0.6206
0.6212
0.6212
0.6212
0.6212
0.6212
0.6212
0.6212
0.6212
0.6212
0.6212
0.6212
0.6213
0.6213
0.6214
0.6217
0.6217
0.6217
0.6217
0.6217
0.6217
0.6217
0.6217
0.6217
0.6217
0.6228
0.6229
0.6229
0.6231
0.6231
0.6231
0.6231
0.6231
0.6231
0.6231
0.6231
0.6231
0.6231

0.6231
0.6231
0.6233
0.6233
0.6233
0.6233
0.6234
0.6234
0.6234
0.6234
0.6236
0.6237
0.6238
0.6238
0.6238
0.6239
0.6241
0.6241
0.6242
0.6249
0.6249
0.6249
0.6249
0.6251
0.6251
0.6251
0.6251
0.6251
0.6251
0.6254
0.6254
0.6258
0.6259
0.6259
0.6260
0.6261
0.6262
0.6264
0.6264
0.6264
0.6264
0.6264
0.6264
0.6264
0.6264
0.6264
0.6264
0.6264
0.6264
0.6269

0.6269
0.6269
0.6269
0.6269
0.6269
0.6269
0.6269
0.6269
0.6269
0.6269
0.6272
0.6272
0.6274
0.6274
0.6274
0.6275
0.6275
0.6275
0.6275
0.6275
0.6275
0.6280
0.6280
0.6281
0.6283
0.6283
0.6283
0.6283
0.6283
0.6284
0.6286
0.6287
0.6289
0.6291
0.6293
0.6293
0.6295
0.6295
0.6295
0.6295
0.6295
0.6295
0.6295
0.6295
0.6295
0.6296
0.6303
0.6306
0.6306
0.6309

0.6309
0.6311
0.6313
0.6313
0.6315
0.6317
0.6317
0.6317
0.6317
0.6317
0.6319
0.6319
0.6322
0.6324
0.6324
0.6324
0.6324
0.6324
0.6324
0.6324
0.6324
0.6324
0.6324
0.6324
0.6325
0.6326
0.6326
0.6327
0.6328
0.6329
0.6332
0.6332
0.6332
0.6336
0.6336
0.6337
0.6338
0.6339
0.6340
0.6342
0.6342
0.6343
0.6343
0.6343
0.6343
0.6343
0.6343
0.6355
0.6355
0.6355

0.6355
0.6359
0.6359
0.6359
0.6359
0.6359
0.6359
0.6359
0.6359
0.6359
0.6359
0.6359
0.6359
0.6359
0.6359
0.6359
0.6361
0.6362
0.6363
0.6363
0.6364
0.6364
0.6364
0.6364
0.6366
0.6366
0.6366
0.6366
0.6366
0.6366
0.6367
0.6368
0.6369
0.6369
0.6374
0.6375
0.6375
0.6377
0.6377
0.6377
0.6377
0.6378
0.6378
0.6379
0.6379
0.6379
0.6380
0.6380
0.6380
0.6380

0.6380
0.6380
0.6380
0.6380
0.6380
0.6380
0.6380
0.6380
0.6380
0.6380
0.6380
0.6380
0.6385
0.6386
0.6386
0.6386
0.6387
0.6387
0.6387
0.6387
0.6387
0.6387
0.6387
0.6389
0.6389
0.6389
0.6389
0.6389
0.6389
0.6389
0.6389
0.6389
0.6389
0.6389
0.6389
0.6390
0.6391
0.6391
0.6391
0.6391
0.6391
0.6391
0.6392
0.6392
0.6392
0.6393
0.6394
0.6396
0.6398
0.6398

0.6399
0.6400
0.6400
0.6401
0.6401
0.6404
0.6404
0.6404
0.6405
0.6405
0.6405
0.6406
0.6407
0.6407
0.6407
0.6407
0.6407
0.6407
0.6407
0.6407
0.6407
0.6407
0.6408
0.6408
0.6414
0.6414
0.6414
0.6416
0.6416
0.6416
0.6417
0.6417
0.6417
0.6417
0.6417
0.6417
0.6417
0.6417
0.6417
0.6417
0.6417
0.6417
0.6417
0.6417
0.6417
0.6417
0.6419
0.6419
0.6419
0.6419

0.6427
0.6427
0.6427
0.6430
0.6430
0.6430
0.6432
0.6432
0.6432
0.6432
0.6434
0.6434
0.6435
0.6435
0.6435
0.6436
0.6436
0.6437
0.6437
0.6437
0.6439
0.6439
0.6439
0.6442
0.6443
0.6443
0.6443
0.6445
0.6445
0.6445
0.6446
0.6448
0.6448
0.6449
0.6449
0.6452
0.6452
0.6452
0.6452
0.6457
0.6457
0.6459
0.6460
0.6468
0.6468
0.6468
0.6468
0.6468
0.6468
0.6468

0.6468
0.6469
0.6470
0.6470
0.6470
0.6470
0.6470
0.6470
0.6470
0.6470
0.6470
0.6470
0.6472
0.6472
0.6472
0.6472
0.6475
0.6477
0.6477
0.6477
0.6478
0.6478
0.6478
0.6478
0.6480
0.6482
0.6486
0.6491
0.6493
0.6494
0.6494
0.6494
0.6495
0.6495
0.6496
0.6497
0.6497
0.6497
0.6502
0.6502
0.6502
0.6502
0.6502
0.6505
0.6508
0.6509
0.6509
0.6509
0.6510
0.6510

0.6510
0.6510
0.6510
0.6510
0.6510
0.6510
0.6510
0.6510
0.6510
0.6510
0.6510
0.6510
0.6510
0.6510
0.6510
0.6510
0.6514
0.6517
0.6519
0.6520
0.6520
0.6520
0.6524
0.6524
0.6524
0.6524
0.6524
0.6524
0.6524
0.6524
0.6525
0.6526
0.6528
0.6528
0.6529
0.6530
0.6530
0.6531
0.6534
0.6534
0.6534
0.6534
0.6535
0.6535
0.6535
0.6535
0.6537
0.6539
0.6539
0.6539

0.6539
0.6540
0.6540
0.6541
0.6543
0.6545
0.6546
0.6547
0.6547
0.6547
0.6547
0.6547
0.6547
0.6547
0.6549
0.6552
0.6552
0.6557
0.6557
0.6557
0.6557
0.6562
0.6565
0.6565
0.6565
0.6565
0.6565
0.6566
0.6569
0.6571
0.6571
0.6572
0.6574
0.6574
0.6580
0.6580
0.6581
0.6583
0.6583
0.6583
0.6583
0.6583
0.6583
0.6583
0.6586
0.6587
0.6588
0.6588
0.6588
0.6588

0.6588
0.6588
0.6591
0.6592
0.6594
0.6594
0.6595
0.6595
0.6597
0.6597
0.6597
0.6597
0.6604
0.6604
0.6612
0.6612
0.6612
0.6613
0.6616
0.6616
0.6621
0.6621
0.6621
0.6623
0.6623
0.6624
0.6625
0.6625
0.6628
0.6628
0.6628
0.6631
0.6632
0.6632
0.6633
0.6633
0.6633
0.6633
0.6633
0.6633
0.6633
0.6633
0.6633
0.6633
0.6633
0.6633
0.6633
0.6633
0.6634
0.6635

0.6635
0.6635
0.6635
0.6635
0.6635
0.6635
0.6635
0.6636
0.6636
0.6636
0.6636
0.6636
0.6638
0.6646
0.6646
0.6646
0.6646
0.6646
0.6646
0.6646
0.6649
0.6649
0.6649
0.6649
0.6649
0.6649
0.6649
0.6649
0.6649
0.6649
0.6649
0.6649
0.6649
0.6649
0.6654
0.6654
0.6654
0.6655
0.6655
0.6655
0.6655
0.6655
0.6657
0.6658
0.6658
0.6658
0.6658
0.6659
0.6659
0.6661

0.6662
0.6662
0.6662
0.6662
0.6662
0.6662
0.6662
0.6662
0.6664
0.6664
0.6664
0.6664
0.6664
0.6665
0.6666
0.6666
0.6666
0.6666
0.6666
0.6666
0.6666
0.6666
0.6668
0.6669
0.6670
0.6671
0.6672
0.6672
0.6673
0.6673
0.6675
0.6676
0.6676
0.6676
0.6676
0.6678
0.6681
0.6687
0.6688
0.6688
0.6688
0.6688
0.6688
0.6688
0.6688
0.6688
0.6689
0.6690
0.6693
0.6693

0.6693
0.6694
0.6695
0.6695
0.6695
0.6696
0.6698
0.6698
0.6700
0.6700
0.6700
0.6706
0.6707
0.6708
0.6709
0.6710
0.6710
0.6715
0.6715
0.6716
0.6717
0.6717
0.6717
0.6718
0.6718
0.6719
0.6719
0.6719
0.6719
0.6720
0.6721
0.6724
0.6725
0.6725
0.6725
0.6725
0.6725
0.6725
0.6725
0.6725
0.6725
0.6732
0.6732
0.6733
0.6733
0.6733
0.6735
0.6736
0.6736
0.6736

0.6737
0.6737
0.6740
0.6740
0.6740
0.6740
0.6741
0.6741
0.6742
0.6742
0.6743
0.6745
0.6746
0.6746
0.6746
0.6746
0.6746
0.6746
0.6746
0.6746
0.6746
0.6746
0.6747
0.6747
0.6748
0.6748
0.6748
0.6748
0.6748
0.6749
0.6749
0.6749
0.6750
0.6750
0.6752
0.6752
0.6757
0.6757
0.6758
0.6759
0.6760
0.6760
0.6761
0.6762
0.6762
0.6763
0.6763
0.6764
0.6767
0.6768

0.6769
0.6770
0.6771
0.6773
0.6773
0.6773
0.6773
0.6774
0.6774
0.6774
0.6774
0.6774
0.6774
0.6774
0.6774
0.6774
0.6777
0.6778
0.6778
0.6781
0.6781
0.6784
0.6784
0.6784
0.6784
0.6784
0.6784
0.6784
0.6784
0.6784
0.6784
0.6784
0.6784
0.6784
0.6784
0.6784
0.6784
0.6784
0.6784
0.6784
0.6784
0.6784
0.6784
0.6784
0.6784
0.6784
0.6784
0.6784
0.6788

0.6791
0.6796
0.6796
0.6796
0.6796
0.6796
0.6796
0.6796
0.6796
0.6801
0.6803
0.6803
0.6806
0.6806
0.6806
0.6806
0.6806
0.6807
0.6808
0.6808
0.6808
0.6810
0.6810
0.6810
0.6810
0.6810
0.6810
0.6810
0.6810
0.6810
0.6810
0.6812
0.6812
0.6814
0.6814
0.6814
0.6814
0.6814
0.6814
0.6814
0.6817
0.6817
0.6818
0.6819
0.6819
0.6822
0.6822
0.6822
0.6822
0.6823

0.6824
0.6824
0.6825
0.6825
0.6825
0.6827
0.6827
0.6827
0.6827
0.6829
0.6829
0.6830
0.6830
0.6830
0.6830
0.6830
0.6830
0.6830
0.6830
0.6830
0.6830
0.6830
0.6831
0.6833
0.6833
0.6833
0.6834
0.6834
0.6834
0.6836
0.6836
0.6837
0.6837
0.6840
0.6840
0.6843
0.6847
0.6848
0.6848
0.6848
0.6848
0.6848
0.6848
0.6848
0.6850
0.6852
0.6856
0.6856
0.6856
0.6856

0.6857
0.6857
0.6858
0.6858
0.6858
0.6858
0.6858
0.6858
0.6858
0.6858
0.6858
0.6860
0.6860
0.6861
0.6863
0.6863
0.6863
0.6865
0.6866
0.6866
0.6867
0.6867
0.6868
0.6876
0.6876
0.6876
0.6876
0.6876
0.6877
0.6877
0.6877
0.6879
0.6879
0.6879
0.6879
0.6883
0.6886
0.6886
0.6886
0.6888
0.6888
0.6889
0.6890
0.6890
0.6893
0.6894
0.6894
0.6894
0.6894
0.6894

0.6894
0.6895
0.6895
0.6897
0.6897
0.6897
0.6897
0.6897
0.6900
0.6901
0.6902
0.6902
0.6904
0.6904
0.6904
0.6904
0.6904
0.6904
0.6910
0.6911
0.6913
0.6913
0.6913
0.6913
0.6914
0.6915
0.6916
0.6916
0.6916
0.6918
0.6918
0.6918
0.6918
0.6919
0.6919
0.6922
0.6923
0.6923
0.6925
0.6926
0.6928
0.6928
0.6928
0.6931
0.6931
0.6931
0.6932
0.6936
0.6936
0.6936

0.6939
0.6939
0.6939
0.6939
0.6939
0.6940
0.6940
0.6940
0.6940
0.6941
0.6941
0.6941
0.6941
0.6943
0.6943
0.6944
0.6944
0.6944
0.6945
0.6945
0.6946
0.6946
0.6946
0.6946
0.6946
0.6946
0.6951
0.6952
0.6953
0.6953
0.6953
0.6953
0.6954
0.6954
0.6955
0.6955
0.6955
0.6955
0.6955
0.6955
0.6955
0.6955
0.6955
0.6956
0.6956
0.6956
0.6956
0.6956
0.6956
0.6956

0.6956
0.6958
0.6959
0.6959
0.6959
0.6959
0.6959
0.6965
0.6966
0.6966
0.6967
0.6967
0.6968
0.6969
0.6970
0.6970
0.6973
0.6973
0.6975
0.6976
0.6976
0.6976
0.6976
0.6976
0.6976
0.6976
0.6976
0.6976
0.6976
0.6977
0.6977
0.6977
0.6977
0.6977
0.6977
0.6977
0.6977
0.6980
0.6983
0.6983
0.6986
0.6986
0.6986
0.6991
0.6995
0.7000
0.7000
0.7001
0.7001
0.7002

0.7003
0.7005
0.7008
0.7008
0.7009
0.7009
0.7010
0.7010
0.7010
0.7010
0.7010
0.7010
0.7010
0.7010
0.7010
0.7010
0.7011
0.7011
0.7011
0.7012
0.7012
0.7015
0.7015
0.7016
0.7016
0.7016
0.7016
0.7016
0.7016
0.7016
0.7016
0.7018
0.7019
0.7021
0.7021
0.7021
0.7021
0.7021
0.7021
0.7022
0.7027
0.7028
0.7028
0.7028
0.7028
0.7028
0.7028
0.7028
0.7028
0.7028

0.7028
0.7028
0.7028
0.7028
0.7028
0.7028
0.7029
0.7029
0.7035
0.7036
0.7040
0.7040
0.7040
0.7040
0.7040
0.7040
0.7040
0.7040
0.7040
0.7040
0.7040
0.7042
0.7042
0.7044
0.7044
0.7047
0.7048
0.7048
0.7049
0.7049
0.7049
0.7049
0.7049
0.7050
0.7050
0.7056
0.7056
0.7056
0.7056
0.7056
0.7056
0.7056
0.7056
0.7056
0.7056
0.7056
0.7056
0.7057
0.7058
0.7061

0.7062
0.7062
0.7064
0.7065
0.7065
0.7065
0.7066
0.7067
0.7068
0.7070
0.7072
0.7073
0.7073
0.7073
0.7073
0.7073
0.7073
0.7073
0.7073
0.7075
0.7076
0.7077
0.7078
0.7078
0.7078
0.7078
0.7078
0.7082
0.7082
0.7083
0.7083
0.7083
0.7083
0.7085
0.7085
0.7085
0.7085
0.7085
0.7085
0.7085
0.7085
0.7085
0.7085
0.7085
0.7085
0.7085
0.7086
0.7086
0.7086
0.7088

0.7090
0.7095
0.7095
0.7095
0.7095
0.7096
0.7096
0.7096
0.7098
0.7098
0.7098
0.7100
0.7100
0.7100
0.7100
0.7100
0.7100
0.7103
0.7103
0.7107
0.7107
0.7108
0.7108
0.7108
0.7111
0.7114
0.7114
0.7115
0.7115
0.7115
0.7117
0.7118
0.7118
0.7118
0.7118
0.7118
0.7118
0.7118
0.7118
0.7120
0.7120
0.7120
0.7120
0.7120
0.7120
0.7123
0.7125
0.7126
0.7128
0.7132

0.7133
0.7133
0.7133
0.7134
0.7134
0.7134
0.7134
0.7136
0.7138
0.7138
0.7139
0.7139
0.7139
0.7139
0.7139
0.7139
0.7139
0.7139
0.7139
0.7139
0.7139
0.7139
0.7139
0.7139
0.7139
0.7140
0.7140
0.7140
0.7140
0.7140
0.7142
0.7142
0.7142
0.7144
0.7145
0.7146
0.7146
0.7150
0.7150
0.7151
0.7151
0.7151
0.7155
0.7155
0.7156
0.7160
0.7160
0.7161
0.7162
0.7163

0.7165
0.7168
0.7168
0.7168
0.7168
0.7168
0.7168
0.7168
0.7170
0.7170
0.7170
0.7170
0.7170
0.7170
0.7172
0.7173
0.7175
0.7175
0.7176
0.7176
0.7176
0.7176
0.7176
0.7178
0.7178
0.7179
0.7179
0.7179
0.7179
0.7179
0.7179
0.7180
0.7180
0.7180
0.7180
0.7180
0.7180
0.7180
0.7180
0.7180
0.7180
0.7180
0.7180
0.7180
0.7180
0.7180
0.7180
0.7180
0.7184
0.7184

0.7187
0.7188
0.7189
0.7189
0.7189
0.7194
0.7194
0.7194
0.7194
0.7194
0.7195
0.7196
0.7196
0.7197
0.7197
0.7197
0.7197
0.7197
0.7197
0.7197
0.7198
0.7198
0.7200
0.7203
0.7203
0.7203
0.7203
0.7203
0.7203
0.7206
0.7206
0.7206
0.7206
0.7206
0.7208
0.7209
0.7212
0.7214
0.7214
0.7214
0.7216
0.7216
0.7216
0.7216
0.7217
0.7221
0.7221
0.7221
0.7221
0.7221

0.7222
0.7222
0.7222
0.7223
0.7226
0.7226
0.7230
0.7230
0.7230
0.7230
0.7230
0.7231
0.7231
0.7231
0.7231
0.7231
0.7231
0.7233
0.7235
0.7237
0.7237
0.7238
0.7241
0.7241
0.7241
0.7243
0.7243
0.7243
0.7243
0.7243
0.7245
0.7245
0.7245
0.7245
0.7245
0.7245
0.7245
0.7245
0.7246
0.7247
0.7247
0.7247
0.7247
0.7249
0.7249
0.7249
0.7249
0.7249
0.7249
0.7250

0.7250
0.7252
0.7252
0.7252
0.7252
0.7254
0.7255
0.7255
0.7256
0.7256
0.7256
0.7258
0.7259
0.7259
0.7259
0.7260
0.7260
0.7261
0.7261
0.7261
0.7261
0.7261
0.7261
0.7262
0.7264
0.7264
0.7266
0.7266
0.7269
0.7269
0.7269
0.7269
0.7269
0.7269
0.7272
0.7274
0.7274
0.7274
0.7274
0.7274
0.7274
0.7275
0.7275
0.7277
0.7282
0.7285
0.7286
0.7287
0.7287
0.7289

0.7291
0.7291
0.7291
0.7292
0.7292
0.7293
0.7294
0.7294
0.7296
0.7296
0.7296
0.7296
0.7296
0.7296
0.7296
0.7297
0.7298
0.7299
0.7302
0.7302
0.7302
0.7303
0.7304
0.7305
0.7308
0.7311
0.7311
0.7311
0.7311
0.7312
0.7317
0.7317
0.7320
0.7321
0.7322
0.7323
0.7323
0.7323
0.7323
0.7323
0.7323
0.7323
0.7323
0.7324
0.7325
0.7325
0.7325
0.7325
0.7325
0.7325

0.7325
0.7326
0.7326
0.7327
0.7328
0.7331
0.7331
0.7334
0.7334
0.7334
0.7334
0.7334
0.7336
0.7337
0.7338
0.7338
0.7338
0.7338
0.7342
0.7342
0.7343
0.7343
0.7344
0.7346
0.7349
0.7352
0.7355
0.7358
0.7358
0.7359
0.7359
0.7360
0.7361
0.7361
0.7362
0.7362
0.7362
0.7362
0.7362
0.7362
0.7362
0.7363
0.7363
0.7363
0.7363
0.7369
0.7371
0.7371
0.7372
0.7373

0.7373
0.7373
0.7373
0.7378
0.7382
0.7385
0.7386
0.7386
0.7386
0.7386
0.7386
0.7386
0.7390
0.7391
0.7391
0.7392
0.7392
0.7392
0.7392
0.7394
0.7394
0.7398
0.7398
0.7398
0.7398
0.7398
0.7400
0.7400
0.7400
0.7400
0.7406
0.7408
0.7408
0.7409
0.7409
0.7409
0.7412
0.7412
0.7413
0.7413
0.7413
0.7413
0.7413
0.7413
0.7413
0.7414
0.7414
0.7414
0.7417
0.7417

0.7418
0.7422
0.7423
0.7423
0.7425
0.7427
0.7427
0.7427
0.7429
0.7429
0.7431
0.7431
0.7432
0.7433
0.7433
0.7435
0.7435
0.7436
0.7439
0.7439
0.7440
0.7441
0.7443
0.7444
0.7444
0.7446
0.7451
0.7455
0.7455
0.7456
0.7456
0.7458
0.7459
0.7462
0.7462
0.7462
0.7464
0.7466
0.7466
0.7466
0.7466
0.7466
0.7466
0.7466
0.7466
0.7467
0.7467
0.7474
0.7474
0.7474

0.7474
0.7474
0.7474
0.7474
0.7474
0.7474
0.7474
0.7474
0.7474
0.7474
0.7474
0.7474
0.7474
0.7474
0.7477
0.7477
0.7477
0.7477
0.7477
0.7478
0.7479
0.7479
0.7479
0.7480
0.7480
0.7486
0.7486
0.7490
0.7490
0.7490
0.7490
0.7490
0.7490
0.7490
0.7490
0.7490
0.7490
0.7490
0.7490
0.7490
0.7491
0.7491
0.7492
0.7492
0.7492
0.7493
0.7493
0.7493
0.7495
0.7496

0.7496
0.7496
0.7496
0.7496
0.7496
0.7496
0.7500
0.7500
0.7501
0.7501
0.7501
0.7501
0.7501
0.7506
0.7506
0.7506
0.7507
0.7509
0.7509
0.7509
0.7509
0.7510
0.7511
0.7512
0.7514
0.7518
0.7518
0.7519
0.7519
0.7519
0.7522
0.7524
0.7525
0.7525
0.7528
0.7530
0.7533
0.7533
0.7533
0.7535
0.7536
0.7538
0.7540
0.7541
0.7541
0.7541
0.7541
0.7542
0.7542
0.7542

0.7543
0.7543
0.7544
0.7544
0.7544
0.7544
0.7545
0.7546
0.7546
0.7546
0.7546
0.7546
0.7549
0.7550
0.7550
0.7557
0.7558
0.7566
0.7567
0.7569
0.7569
0.7570
0.7570
0.7570
0.7571
0.7575
0.7576
0.7579
0.7579
0.7580
0.7586
0.7587
0.7587
0.7587
0.7587
0.7587
0.7588
0.7588
0.7588
0.7588
0.7588
0.7589
0.7590
0.7590
0.7590
0.7590
0.7590
0.7591
0.7593
0.7594

0.7594
0.7595
0.7595
0.7595
0.7595
0.7595
0.7595
0.7599
0.7600
0.7600
0.7600
0.7600
0.7603
0.7603
0.7604
0.7606
0.7606
0.7606
0.7606
0.7606
0.7609
0.7609
0.7609
0.7609
0.7609
0.7609
0.7609
0.7609
0.7609
0.7609
0.7609
0.7609
0.7609
0.7609
0.7609
0.7611
0.7613
0.7614
0.7614
0.7614
0.7614
0.7614
0.7619
0.7619
0.7620
0.7620
0.7621
0.7621
0.7621
0.7622

0.7623
0.7623
0.7623
0.7623
0.7623
0.7623
0.7623
0.7623
0.7624
0.7624
0.7624
0.7626
0.7626
0.7627
0.7632
0.7632
0.7633
0.7633
0.7636
0.7638
0.7638
0.7641
0.7641
0.7641
0.7641
0.7642
0.7642
0.7643
0.7643
0.7643
0.7644
0.7644
0.7644
0.7644
0.7644
0.7645
0.7645
0.7645
0.7645
0.7645
0.7646
0.7646
0.7649
0.7649
0.7649
0.7649
0.7649
0.7649
0.7652
0.7652

0.7653
0.7654
0.7655
0.7655
0.7659
0.7659
0.7659
0.7659
0.7661
0.7661
0.7663
0.7663
0.7663
0.7665
0.7665
0.7665
0.7665
0.7665
0.7666
0.7666
0.7666
0.7666
0.7666
0.7666
0.7666
0.7666
0.7667
0.7667
0.7667
0.7667
0.7667
0.7667
0.7668
0.7672
0.7672
0.7672
0.7673
0.7675
0.7675
0.7675
0.7675
0.7675
0.7676
0.7676
0.7676
0.7676
0.7676
0.7676
0.7676
0.7676

0.7676
0.7676
0.7676
0.7676
0.7677
0.7678
0.7678
0.7678
0.7678
0.7678
0.7681
0.7684
0.7686
0.7686
0.7691
0.7691
0.7691
0.7691
0.7692
0.7692
0.7692
0.7692
0.7692
0.7693
0.7693
0.7693
0.7695
0.7695
0.7697
0.7697
0.7698
0.7698
0.7701
0.7704
0.7704
0.7704
0.7704
0.7705
0.7705
0.7705
0.7705
0.7705
0.7707
0.7709
0.7710
0.7713
0.7713
0.7717
0.7719
0.7722

0.7724
0.7724
0.7724
0.7724
0.7724
0.7727
0.7729
0.7729
0.7729
0.7731
0.7731
0.7732
0.7732
0.7732
0.7732
0.7732
0.7732
0.7734
0.7734
0.7734
0.7736
0.7737
0.7737
0.7737
0.7739
0.7740
0.7740
0.7740
0.7740
0.7740
0.7742
0.7742
0.7742
0.7742
0.7742
0.7746
0.7746
0.7748
0.7748
0.7748
0.7748
0.7748
0.7748
0.7748
0.7748
0.7748
0.7752
0.7752
0.7753
0.7755

0.7755
0.7755
0.7758
0.7758
0.7758
0.7758
0.7758
0.7758
0.7758
0.7759
0.7761
0.7763
0.7763
0.7763
0.7763
0.7763
0.7763
0.7763
0.7763
0.7764
0.7766
0.7766
0.7766
0.7766
0.7766
0.7766
0.7766
0.7766
0.7772
0.7772
0.7772
0.7773
0.7773
0.7775
0.7776
0.7777
0.7783
0.7785
0.7787
0.7788
0.7790
0.7790
0.7790
0.7791
0.7791
0.7792
0.7792
0.7792
0.7792
0.7792

0.7792
0.7792
0.7792
0.7792
0.7796
0.7798
0.7798
0.7798
0.7798
0.7798
0.7798
0.7804
0.7805
0.7806
0.7810
0.7810
0.7810
0.7810
0.7810
0.7810
0.7810
0.7810
0.7810
0.7812
0.7815
0.7816
0.7819
0.7821
0.7821
0.7821
0.7821
0.7821
0.7821
0.7822
0.7823
0.7825
0.7825
0.7826
0.7826
0.7826
0.7826
0.7826
0.7826
0.7826
0.7827
0.7828
0.7829
0.7829
0.7829
0.7832

0.7833
0.7833
0.7838
0.7838
0.7838
0.7839
0.7839
0.7841
0.7842
0.7843
0.7844
0.7844
0.7844
0.7844
0.7845
0.7845
0.7845
0.7846
0.7849
0.7854
0.7854
0.7854
0.7857
0.7857
0.7857
0.7857
0.7860
0.7860
0.7860
0.7862
0.7863
0.7863
0.7863
0.7863
0.7863
0.7865
0.7866
0.7866
0.7866
0.7866
0.7866
0.7866
0.7866
0.7866
0.7866
0.7866
0.7867
0.7867
0.7867
0.7870

0.7870
0.7870
0.7870
0.7870
0.7870
0.7871
0.7871
0.7871
0.7872
0.7872
0.7873
0.7873
0.7873
0.7873
0.7876
0.7876
0.7876
0.7876
0.7876
0.7876
0.7876
0.7876
0.7876
0.7876
0.7876
0.7877
0.7877
0.7877
0.7877
0.7877
0.7877
0.7877
0.7877
0.7877
0.7877
0.7877
0.7877
0.7877
0.7879
0.7880
0.7880
0.7881
0.7884
0.7884
0.7884
0.7884
0.7884
0.7884
0.7884
0.7884

0.7884
0.7884
0.7884
0.7884
0.7884
0.7884
0.7884
0.7885
0.7885
0.7886
0.7887
0.7889
0.7889
0.7891
0.7891
0.7891
0.7891
0.7891
0.7891
0.7894
0.7894
0.7894
0.7894
0.7896
0.7896
0.7898
0.7899
0.7899
0.7899
0.7899
0.7899
0.7899
0.7899
0.7899
0.7905
0.7908
0.7908
0.7908
0.7908
0.7908
0.7909
0.7911
0.7913
0.7913
0.7913
0.7914
0.7915
0.7915
0.7916
0.7917

0.7919
0.7919
0.7919
0.7919
0.7919
0.7919
0.7919
0.7919
0.7919
0.7919
0.7920
0.7923
0.7924
0.7924
0.7925
0.7926
0.7926
0.7926
0.7926
0.7926
0.7928
0.7928
0.7928
0.7931
0.7932
0.7932
0.7932
0.7933
0.7933
0.7933
0.7935
0.7938
0.7938
0.7938
0.7940
0.7941
0.7942
0.7942
0.7942
0.7943
0.7945
0.7945
0.7945
0.7947
0.7949
0.7949
0.7949
0.7949
0.7949
0.7949

0.7949
0.7949
0.7949
0.7949
0.7950
0.7955
0.7955
0.7955
0.7955
0.7955
0.7957
0.7957
0.7957
0.7957
0.7957
0.7958
0.7959
0.7959
0.7959
0.7959
0.7964
0.7964
0.7964
0.7964
0.7964
0.7964
0.7966
0.7967
0.7967
0.7968
0.7968
0.7972
0.7972
0.7973
0.7974
0.7974
0.7974
0.7974
0.7976
0.7980
0.7980
0.7980
0.7987
0.7992
0.7992
0.7994
0.7994
0.7994
0.7995
0.7997

0.7998
0.7998
0.7998
0.7999
0.7999
0.8000
0.8001
0.8001
0.8001
0.8001
0.8001
0.8001
0.8003
0.8003
0.8005
0.8006
0.8006
0.8006
0.8007
0.8007
0.8007
0.8011
0.8012
0.8012
0.8014
0.8014
0.8014
0.8015
0.8015
0.8017
0.8017
0.8017
0.8017
0.8017
0.8017
0.8019
0.8019
0.8019
0.8019
0.8019
0.8019
0.8019
0.8021
0.8021
0.8021
0.8021
0.8022
0.8023
0.8027
0.8028

0.8029
0.8029
0.8029
0.8029
0.8029
0.8029
0.8030
0.8030
0.8030
0.8030
0.8030
0.8030
0.8030
0.8030
0.8030
0.8030
0.8030
0.8030
0.8030
0.8030
0.8030
0.8030
0.8030
0.8031
0.8032
0.8032
0.8032
0.8033
0.8033
0.8033
0.8033
0.8033
0.8034
0.8034
0.8035
0.8035
0.8035
0.8035
0.8035
0.8035
0.8037
0.8037
0.8037
0.8038
0.8040
0.8045
0.8045
0.8045
0.8045
0.8045

0.8045
0.8045
0.8046
0.8046
0.8047
0.8050
0.8051
0.8051
0.8051
0.8052
0.8052
0.8054
0.8054
0.8057
0.8057
0.8057
0.8058
0.8058
0.8058
0.8058
0.8059
0.8059
0.8059
0.8059
0.8059
0.8061
0.8061
0.8061
0.8061
0.8061
0.8062
0.8062
0.8062
0.8062
0.8063
0.8063
0.8063
0.8063
0.8064
0.8064
0.8064
0.8064
0.8064
0.8065
0.8068
0.8068
0.8068
0.8068
0.8068
0.8068

0.8070
0.8070
0.8070
0.8074
0.8076
0.8078
0.8079
0.8079
0.8081
0.8081
0.8082
0.8083
0.8084
0.8084
0.8084
0.8084
0.8084
0.8084
0.8084
0.8084
0.8084
0.8084
0.8084
0.8084
0.8084
0.8084
0.8084
0.8084
0.8085
0.8086
0.8086
0.8086
0.8089
0.8089
0.8089
0.8089
0.8089
0.8089
0.8093
0.8093
0.8093
0.8093
0.8094
0.8098
0.8101
0.8103
0.8106
0.8108
0.8108
0.8108

0.8109
0.8112
0.8112
0.8112
0.8114
0.8114
0.8114
0.8114
0.8114
0.8114
0.8114
0.8114
0.8114
0.8114
0.8114
0.8115
0.8115
0.8119
0.8121
0.8124
0.8124
0.8124
0.8124
0.8125
0.8125
0.8125
0.8125
0.8126
0.8127
0.8127
0.8129
0.8129
0.8129
0.8131
0.8131
0.8132
0.8133
0.8133
0.8133
0.8133
0.8133
0.8134
0.8136
0.8136
0.8136
0.8136
0.8137
0.8139
0.8142
0.8143

0.8143
0.8143
0.8143
0.8149
0.8151
0.8151
0.8151
0.8151
0.8151
0.8151
0.8151
0.8151
0.8151
0.8152
0.8153
0.8153
0.8153
0.8157
0.8157
0.8157
0.8157
0.8157
0.8159
0.8159
0.8159
0.8159
0.8159
0.8159
0.8161
0.8161
0.8161
0.8161
0.8161
0.8161
0.8161
0.8161
0.8161
0.8161
0.8161
0.8161
0.8161
0.8161
0.8161
0.8161
0.8161
0.8163
0.8168
0.8169
0.8169
0.8169

0.8170
0.8171
0.8171
0.8171
0.8172
0.8172
0.8172
0.8174
0.8174
0.8174
0.8174
0.8174
0.8174
0.8174
0.8174
0.8174
0.8174
0.8174
0.8174
0.8174
0.8174
0.8174
0.8174
0.8174
0.8175
0.8175
0.8175
0.8175
0.8175
0.8175
0.8175
0.8175
0.8175
0.8176
0.8176
0.8176
0.8176
0.8176
0.8176
0.8178
0.8179
0.8181
0.8181
0.8181
0.8181
0.8181
0.8181
0.8181
0.8182
0.8182

0.8182
0.8182
0.8182
0.8182
0.8183
0.8183
0.8183
0.8183
0.8183
0.8185
0.8185
0.8185
0.8186
0.8186
0.8186
0.8186
0.8186
0.8187
0.8188
0.8188
0.8188
0.8188
0.8189
0.8189
0.8192
0.8193
0.8193
0.8193
0.8194
0.8194
0.8194
0.8195
0.8195
0.8195
0.8195
0.8195
0.8195
0.8195
0.8195
0.8195
0.8196
0.8196
0.8196
0.8197
0.8198
0.8198
0.8199
0.8199
0.8199
0.8199

0.8199
0.8201
0.8202
0.8202
0.8202
0.8202
0.8202
0.8202
0.8203
0.8205
0.8205
0.8205
0.8205
0.8206
0.8206
0.8207
0.8207
0.8207
0.8208
0.8211
0.8211
0.8212
0.8212
0.8212
0.8212
0.8212
0.8212
0.8212
0.8212
0.8212
0.8216
0.8217
0.8218
0.8218
0.8218
0.8218
0.8218
0.8220
0.8223
0.8223
0.8223
0.8223
0.8223
0.8223
0.8223
0.8225
0.8225
0.8229
0.8229
0.8229

0.8232
0.8233
0.8233
0.8234
0.8235
0.8236
0.8237
0.8238
0.8238
0.8238
0.8238
0.8238
0.8241
0.8241
0.8241
0.8241
0.8241
0.8242
0.8242
0.8242
0.8242
0.8242
0.8242
0.8243
0.8244
0.8244
0.8244
0.8244
0.8244
0.8244
0.8244
0.8244
0.8244
0.8244
0.8244
0.8244
0.8244
0.8244
0.8244
0.8246
0.8246
0.8247
0.8247
0.8247
0.8248
0.8248
0.8248
0.8248
0.8248
0.8248

0.8248
0.8248
0.8249
0.8249
0.8252
0.8252
0.8252
0.8254
0.8256
0.8256
0.8259
0.8259
0.8259
0.8260
0.8260
0.8260
0.8260
0.8263
0.8263
0.8263
0.8268
0.8268
0.8268
0.8268
0.8268
0.8268
0.8268
0.8268
0.8268
0.8268
0.8268
0.8268
0.8268
0.8268
0.8268
0.8269
0.8269
0.8271
0.8271
0.8271
0.8271
0.8271
0.8271
0.8271
0.8271
0.8271
0.8273
0.8273
0.8275
0.8279

0.8282
0.8282
0.8282
0.8283
0.8283
0.8283
0.8286
0.8286
0.8288
0.8288
0.8288
0.8288
0.8291
0.8293
0.8293
0.8293
0.8295
0.8295
0.8295
0.8296
0.8298
0.8298
0.8298
0.8299
0.8299
0.8299
0.8300
0.8300
0.8300
0.8302
0.8302
0.8302
0.8302
0.8302
0.8302
0.8302
0.8302
0.8304
0.8305
0.8305
0.8305
0.8306
0.8306
0.8308
0.8308
0.8310
0.8310
0.8310
0.8311
0.8311

0.8312
0.8312
0.8312
0.8312
0.8312
0.8312
0.8312
0.8312
0.8312
0.8312
0.8313
0.8313
0.8313
0.8313
0.8313
0.8314
0.8316
0.8316
0.8316
0.8316
0.8316
0.8316
0.8316
0.8316
0.8316
0.8316
0.8316
0.8316
0.8317
0.8317
0.8317
0.8317
0.8320
0.8320
0.8320
0.8320
0.8320
0.8320
0.8320
0.8321
0.8321
0.8325
0.8325
0.8325
0.8326
0.8326
0.8328
0.8328
0.8331
0.8332

0.8332
0.8332
0.8333
0.8334
0.8334
0.8334
0.8336
0.8336
0.8336
0.8336
0.8337
0.8337
0.8338
0.8338
0.8339
0.8341
0.8341
0.8341
0.8341
0.8342
0.8343
0.8343
0.8343
0.8344
0.8345
0.8345
0.8346
0.8346
0.8347
0.8348
0.8348
0.8349
0.8350
0.8353
0.8354
0.8356
0.8357
0.8360
0.8360
0.8360
0.8360
0.8360
0.8360
0.8360
0.8360
0.8360
0.8360
0.8361
0.8362
0.8364

0.8364
0.8365
0.8365
0.8367
0.8368
0.8368
0.8370
0.8372
0.8372
0.8373
0.8375
0.8375
0.8375
0.8378
0.8378
0.8379
0.8379
0.8379
0.8379
0.8380
0.8382
0.8387
0.8388
0.8389
0.8389
0.8389
0.8389
0.8389
0.8389
0.8389
0.8389
0.8389
0.8389
0.8389
0.8389
0.8389
0.8389
0.8391
0.8391
0.8391
0.8394
0.8395
0.8395
0.8395
0.8395
0.8395
0.8395
0.8395
0.8395
0.8395

0.8395
0.8395
0.8396
0.8396
0.8398
0.8400
0.8401
0.8401
0.8401
0.8402
0.8402
0.8403
0.8406
0.8406
0.8407
0.8408
0.8409
0.8409
0.8415
0.8415
0.8415
0.8415
0.8415
0.8417
0.8417
0.8417
0.8417
0.8417
0.8418
0.8418
0.8419
0.8419
0.8420
0.8423
0.8423
0.8424
0.8424
0.8424
0.8426
0.8427
0.8427
0.8427
0.8430
0.8430
0.8430
0.8430
0.8431
0.8431
0.8435
0.8435

0.8435
0.8435
0.8437
0.8438
0.8440
0.8440
0.8440
0.8440
0.8440
0.8440
0.8440
0.8441
0.8442
0.8442
0.8442
0.8442
0.8442
0.8446
0.8447
0.8447
0.8447
0.8448
0.8448
0.8448
0.8448
0.8448
0.8448
0.8448
0.8448
0.8448
0.8448
0.8449
0.8449
0.8449
0.8449
0.8450
0.8451
0.8451
0.8451
0.8451
0.8451
0.8453
0.8454
0.8454
0.8454
0.8454
0.8454
0.8454
0.8454
0.8454

0.8454
0.8454
0.8454
0.8455
0.8455
0.8455
0.8455
0.8457
0.8458
0.8458
0.8460
0.8462
0.8464
0.8465
0.8466
0.8469
0.8469
0.8469
0.8470
0.8470
0.8470
0.8470
0.8470
0.8470
0.8470
0.8472
0.8472
0.8473
0.8473
0.8476
0.8476
0.8477
0.8477
0.8477
0.8477
0.8479
0.8479
0.8479
0.8479
0.8479
0.8480
0.8480
0.8480
0.8481
0.8481
0.8481
0.8481
0.8484
0.8485
0.8485

0.8485
0.8488
0.8489
0.8491
0.8491
0.8494
0.8494
0.8494
0.8494
0.8494
0.8497
0.8499
0.8499
0.8499
0.8499
0.8502
0.8502
0.8502
0.8502
0.8503
0.8504
0.8504
0.8507
0.8507
0.8507
0.8509
0.8510
0.8510
0.8510
0.8512
0.8512
0.8512
0.8512
0.8512
0.8512
0.8512
0.8513
0.8514
0.8514
0.8515
0.8515
0.8515
0.8517
0.8517
0.8517
0.8517
0.8517
0.8517
0.8517
0.8517

0.8517
0.8517
0.8519
0.8520
0.8522
0.8523
0.8524
0.8524
0.8524
0.8524
0.8524
0.8524
0.8524
0.8524
0.8524
0.8524
0.8524
0.8524
0.8524
0.8524
0.8524
0.8524
0.8524
0.8524
0.8524
0.8524
0.8524
0.8524
0.8524
0.8524
0.8524
0.8524
0.8524
0.8524
0.8524
0.8524
0.8524
0.8524
0.8524
0.8524
0.8524
0.8524
0.8524
0.8525
0.8525
0.8527
0.8531
0.8531
0.8534
0.8534
0.8534

0.8534
0.8535
0.8535
0.8535
0.8535
0.8535
0.8535
0.8539
0.8539
0.8539
0.8539
0.8539
0.8539
0.8539
0.8539
0.8539
0.8539
0.8539
0.8540
0.8542
0.8543
0.8543
0.8544
0.8544
0.8544
0.8547
0.8547
0.8548
0.8551
0.8554
0.8554
0.8554
0.8556
0.8556
0.8556
0.8562
0.8562
0.8562
0.8562
0.8563
0.8563
0.8563
0.8564
0.8564
0.8564
0.8564
0.8567
0.8568
0.8572
0.8572

0.8572
0.8573
0.8574
0.8580
0.8580
0.8580
0.8580
0.8581
0.8584
0.8584
0.8584
0.8584
0.8584
0.8584
0.8584
0.8584
0.8589
0.8589
0.8589
0.8589
0.8589
0.8589
0.8591
0.8594
0.8596
0.8598
0.8598
0.8598
0.8598
0.8598
0.8598
0.8598
0.8598
0.8598
0.8598
0.8598
0.8598
0.8598
0.8598
0.8599
0.8599
0.8599
0.8600
0.8600
0.8600
0.8600
0.8600
0.8601
0.8603
0.8603

0.8605
0.8605
0.8606
0.8606
0.8607
0.8609
0.8610
0.8613
0.8615
0.8615
0.8615
0.8615
0.8617
0.8617
0.8617
0.8619
0.8622
0.8622
0.8625
0.8625
0.8625
0.8625
0.8626
0.8627
0.8627
0.8627
0.8627
0.8627
0.8627
0.8627
0.8627
0.8627
0.8627
0.8628
0.8630
0.8631
0.8632
0.8633
0.8633
0.8636
0.8636
0.8639
0.8639
0.8639
0.8641
0.8641
0.8641
0.8641
0.8641
0.8641

0.8641
0.8641
0.8641
0.8641
0.8641
0.8645
0.8645
0.8646
0.8646
0.8646
0.8646
0.8646
0.8648
0.8648
0.8648
0.8648
0.8648
0.8648
0.8648
0.8648
0.8648
0.8648
0.8649
0.8649
0.8649
0.8649
0.8649
0.8649
0.8650
0.8651
0.8654
0.8654
0.8654
0.8654
0.8654
0.8654
0.8654
0.8654
0.8654
0.8654
0.8657
0.8658
0.8659
0.8660
0.8660
0.8660
0.8661
0.8661
0.8661
0.8661

0.8661
0.8662
0.8662
0.8663
0.8664
0.8664
0.8664
0.8664
0.8668
0.8669
0.8672
0.8672
0.8672
0.8672
0.8672
0.8672
0.8673
0.8673
0.8673
0.8673
0.8673
0.8673
0.8673
0.8673
0.8673
0.8673
0.8673
0.8673
0.8673
0.8673
0.8678
0.8678
0.8678
0.8678
0.8678
0.8678
0.8678
0.8678
0.8678
0.8678
0.8678
0.8678
0.8678
0.8678
0.8678
0.8678
0.8679
0.8680
0.8680
0.8680

0.8680
0.8682
0.8683
0.8684
0.8684
0.8684
0.8684
0.8684
0.8684
0.8687
0.8687
0.8688
0.8688
0.8689
0.8689
0.8689
0.8689
0.8690
0.8691
0.8692
0.8694
0.8694
0.8694
0.8694
0.8695
0.8699
0.8701
0.8701
0.8701
0.8701
0.8701
0.8701
0.8701
0.8701
0.8703
0.8703
0.8703
0.8706
0.8706
0.8708
0.8709
0.8711
0.8712
0.8712
0.8712
0.8715
0.8715
0.8721
0.8722
0.8722

0.8722
0.8722
0.8724
0.8724
0.8725
0.8725
0.8725
0.8725
0.8725
0.8725
0.8725
0.8725
0.8726
0.8726
0.8726
0.8726
0.8731
0.8731
0.8732
0.8732
0.8733
0.8733
0.8734
0.8734
0.8734
0.8734
0.8734
0.8734
0.8734
0.8735
0.8736
0.8736
0.8736
0.8736
0.8736
0.8736
0.8736
0.8736
0.8736
0.8736
0.8736
0.8736
0.8736
0.8737
0.8737
0.8739
0.8739
0.8740
0.8740
0.8740

0.8740
0.8740
0.8740
0.8740
0.8740
0.8740
0.8740
0.8740
0.8740
0.8740
0.8740
0.8740
0.8740
0.8740
0.8740
0.8740
0.8740
0.8740
0.8741
0.8742
0.8742
0.8742
0.8742
0.8742
0.8742
0.8744
0.8745
0.8745
0.8745
0.8745
0.8745
0.8745
0.8748
0.8751
0.8751
0.8752
0.8752
0.8752
0.8752
0.8752
0.8752
0.8752
0.8752
0.8752
0.8752
0.8752
0.8752
0.8754
0.8758
0.8758

0.8758
0.8758
0.8758
0.8758
0.8759
0.8759
0.8760
0.8766
0.8766
0.8766
0.8769
0.8769
0.8770
0.8770
0.8770
0.8771
0.8772
0.8774
0.8774
0.8775
0.8779
0.8779
0.8779
0.8779
0.8779
0.8779
0.8779
0.8779
0.8779
0.8779
0.8779
0.8779
0.8779
0.8779
0.8779
0.8779
0.8780
0.8780
0.8780
0.8780
0.8780
0.8780
0.8785
0.8785
0.8785
0.8785
0.8785
0.8785
0.8786
0.8786

0.8786
0.8786
0.8786
0.8789
0.8789
0.8792
0.8793
0.8793
0.8795
0.8795
0.8796
0.8796
0.8796
0.8796
0.8796
0.8796
0.8796
0.8796
0.8798
0.8798
0.8798
0.8798
0.8798
0.8798
0.8798
0.8798
0.8798
0.8798
0.8798
0.8798
0.8799
0.8799
0.8799
0.8799
0.8799
0.8800
0.8800
0.8802
0.8803
0.8803
0.8803
0.8804
0.8804
0.8805
0.8806
0.8807
0.8808
0.8808
0.8808
0.8808

0.8809
0.8809
0.8809
0.8809
0.8810
0.8814
0.8817
0.8817
0.8818
0.8818
0.8820
0.8820
0.8820
0.8820
0.8820
0.8820
0.8823
0.8823
0.8823
0.8824
0.8824
0.8825
0.8828
0.8828
0.8832
0.8832
0.8832
0.8832
0.8832
0.8834
0.8835
0.8835
0.8837
0.8837
0.8837
0.8837
0.8837
0.8837
0.8837
0.8837
0.8837
0.8840
0.8840
0.8840
0.8840
0.8840
0.8840
0.8842
0.8842
0.8842

0.8842
0.8842
0.8842
0.8846
0.8846
0.8847
0.8848
0.8848
0.8850
0.8852
0.8856
0.8856
0.8856
0.8857
0.8858
0.8858
0.8858
0.8858
0.8858
0.8859
0.8860
0.8860
0.8860
0.8863
0.8863
0.8863
0.8863
0.8863
0.8863
0.8863
0.8863
0.8864
0.8865
0.8867
0.8867
0.8867
0.8867
0.8867
0.8869
0.8869
0.8869
0.8869
0.8869
0.8869
0.8869
0.8870
0.8875
0.8876
0.8876
0.8878

0.8878
0.8878
0.8878
0.8878
0.8878
0.8878
0.8878
0.8878
0.8878
0.8878
0.8878
0.8878
0.8878
0.8878
0.8879
0.8880
0.8883
0.8883
0.8883
0.8883
0.8886
0.8887
0.8887
0.8888
0.8888
0.8888
0.8888
0.8888
0.8888
0.8888
0.8888
0.8888
0.8889
0.8889
0.8891
0.8891
0.8891
0.8891
0.8891
0.8891
0.8891
0.8891
0.8891
0.8891
0.8893
0.8893
0.8894
0.8894
0.8894
0.8894

0.8894
0.8894
0.8897
0.8897
0.8901
0.8901
0.8901
0.8901
0.8901
0.8901
0.8901
0.8901
0.8901
0.8901
0.8902
0.8903
0.8904
0.8905
0.8906
0.8908
0.8908
0.8908
0.8908
0.8908
0.8909
0.8909
0.8910
0.8910
0.8915
0.8915
0.8915
0.8917
0.8917
0.8918
0.8918
0.8918
0.8920
0.8920
0.8921
0.8922
0.8924
0.8924
0.8925
0.8928
0.8929
0.8932
0.8932
0.8932
0.8932
0.8932

0.8932
0.8932
0.8932
0.8932
0.8932
0.8935
0.8937
0.8937
0.8937
0.8937
0.8938
0.8938
0.8940
0.8940
0.8940
0.8940
0.8940
0.8942
0.8942
0.8942
0.8942
0.8942
0.8942
0.8942
0.8942
0.8943
0.8943
0.8943
0.8947
0.8947
0.8948
0.8948
0.8948
0.8950
0.8953
0.8953
0.8953
0.8957
0.8958
0.8960
0.8961
0.8961
0.8961
0.8961
0.8961
0.8961
0.8961
0.8961
0.8964
0.8964

0.8964
0.8964
0.8964
0.8964
0.8964
0.8964
0.8964
0.8964
0.8964
0.8964
0.8964
0.8965
0.8965
0.8966
0.8966
0.8966
0.8966
0.8966
0.8966
0.8967
0.8967
0.8969
0.8970
0.8970
0.8972
0.8974
0.8974
0.8974
0.8974
0.8974
0.8976
0.8976
0.8976
0.8982
0.8982
0.8982
0.8984
0.8984
0.8984
0.8984
0.8985
0.8985
0.8985
0.8985
0.8985
0.8990
0.8991
0.8992
0.8992
0.8993

0.8993
0.8993
0.8993
0.8993
0.8993
0.8994
0.8994
0.8995
0.8995
0.8995
0.8995
0.8996
0.8996
0.8996
0.8996
0.8996
0.8996
0.8996
0.8997
0.8997
0.8997
0.8997
0.8997
0.8997
0.8998
0.9002
0.9002
0.9002
0.9002
0.9002
0.9005
0.9005
0.9005
0.9005
0.9005
0.9005
0.9005
0.9005
0.9005
0.9005
0.9007
0.9007
0.9007
0.9007
0.9007
0.9007
0.9008
0.9008
0.9012
0.9012

0.9012
0.9013
0.9013
0.9013
0.9013
0.9015
0.9015
0.9018
0.9018
0.9018
0.9018
0.9018
0.9019
0.9019
0.9019
0.9019
0.9019
0.9019
0.9019
0.9019
0.9019
0.9020
0.9020
0.9020
0.9020
0.9020
0.9020
0.9020
0.9020
0.9020
0.9020
0.9020
0.9022
0.9022
0.9023
0.9023
0.9026
0.9028
0.9029
0.9029
0.9029
0.9031
0.9031
0.9031
0.9032
0.9032
0.9032
0.9033
0.9036
0.9039

0.9040
0.9040
0.9041
0.9041
0.9043
0.9044
0.9045
0.9045
0.9045
0.9045
0.9045
0.9045
0.9047
0.9047
0.9047
0.9047
0.9047
0.9047
0.9050
0.9052
0.9052
0.9052
0.9052
0.9053
0.9054
0.9054
0.9055
0.9058
0.9058
0.9059
0.9059
0.9060
0.9061
0.9061
0.9061
0.9061
0.9061
0.9061
0.9064
0.9065
0.9065
0.9065
0.9065
0.9065
0.9065
0.9065
0.9065
0.9065
0.9065
0.9065

0.9065
0.9065
0.9065
0.9070
0.9070
0.9070
0.9070
0.9072
0.9072
0.9075
0.9075
0.9075
0.9075
0.9075
0.9075
0.9075
0.9075
0.9075
0.9075
0.9075
0.9075
0.9075
0.9077
0.9077
0.9077
0.9078
0.9078
0.9078
0.9078
0.9079
0.9081
0.9082
0.9082
0.9084
0.9084
0.9088
0.9088
0.9090
0.9090
0.9091
0.9091
0.9092
0.9092
0.9092
0.9092
0.9092
0.9092
0.9092
0.9092
0.9092

0.9092
0.9092
0.9094
0.9097
0.9097
0.9097
0.9097
0.9097
0.9097
0.9097
0.9097
0.9097
0.9101
0.9101
0.9102
0.9102
0.9104
0.9106
0.9107
0.9107
0.9107
0.9108
0.9108
0.9108
0.9108
0.9108
0.9108
0.9110
0.9110
0.9110
0.9110
0.9110
0.9117
0.9119
0.9121
0.9121
0.9121
0.9121
0.9121
0.9121
0.9123
0.9125
0.9125
0.9126
0.9126
0.9126
0.9128
0.9128
0.9128
0.9129

0.9130
0.9130
0.9130
0.9132
0.9133
0.9133
0.9133
0.9133
0.9137
0.9137
0.9137
0.9137
0.9139
0.9142
0.9144
0.9144
0.9145
0.9145
0.9145
0.9146
0.9146
0.9146
0.9146
0.9146
0.9146
0.9146
0.9146
0.9147
0.9148
0.9148
0.9148
0.9148
0.9148
0.9149
0.9151
0.9151
0.9151
0.9151
0.9153
0.9155
0.9155
0.9156
0.9157
0.9157
0.9157
0.9157
0.9157
0.9157
0.9157
0.9157

0.9157
0.9157
0.9158
0.9159
0.9159
0.9159
0.9159
0.9160
0.9161
0.9161
0.9166
0.9166
0.9167
0.9168
0.9170
0.9170
0.9170
0.9170
0.9170
0.9170
0.9170
0.9170
0.9170
0.9170
0.9171
0.9174
0.9175
0.9175
0.9175
0.9177
0.9178
0.9178
0.9182
0.9183
0.9185
0.9185
0.9185
0.9185
0.9185
0.9186
0.9186
0.9186
0.9186
0.9186
0.9187
0.9187
0.9187
0.9187
0.9187
0.9189

0.9190
0.9190
0.9190
0.9190
0.9190
0.9190
0.9192
0.9192
0.9192
0.9195
0.9195
0.9195
0.9195
0.9197
0.9197
0.9197
0.9197
0.9197
0.9197
0.9197
0.9198
0.9198
0.9198
0.9201
0.9206
0.9206
0.9208
0.9208
0.9208
0.9208
0.9209
0.9209
0.9209
0.9214
0.9214
0.9215
0.9218
0.9219
0.9219
0.9220
0.9220
0.9220
0.9220
0.9223
0.9224
0.9224
0.9224
0.9224
0.9224
0.9224

0.9224
0.9227
0.9227
0.9227
0.9227
0.9227
0.9227
0.9227
0.9227
0.9227
0.9227
0.9229
0.9229
0.9229
0.9229
0.9229
0.9229
0.9229
0.9229
0.9229
0.9229
0.9230
0.9230
0.9230
0.9230
0.9230
0.9230
0.9230
0.9230
0.9230
0.9233
0.9233
0.9235
0.9236
0.9236
0.9236
0.9238
0.9241
0.9242
0.9242
0.9243
0.9243
0.9243
0.9243
0.9243
0.9243
0.9243
0.9243
0.9244
0.9245

0.9246
0.9246
0.9247
0.9248
0.9248
0.9248
0.9249
0.9249
0.9249
0.9249
0.9249
0.9250
0.9254
0.9254
0.9254
0.9254
0.9254
0.9254
0.9256
0.9256
0.9256
0.9257
0.9257
0.9257
0.9257
0.9257
0.9257
0.9257
0.9257
0.9258
0.9259
0.9262
0.9262
0.9262
0.9262
0.9262
0.9264
0.9264
0.9264
0.9264
0.9264
0.9264
0.9264
0.9267
0.9269
0.9269
0.9269
0.9269
0.9270
0.9270

0.9270
0.9270
0.9270
0.9270
0.9270
0.9270
0.9270
0.9270
0.9270
0.9270
0.9272
0.9272
0.9273
0.9273
0.9274
0.9275
0.9275
0.9275
0.9275
0.9275
0.9275
0.9275
0.9275
0.9275
0.9275
0.9275
0.9275
0.9275
0.9275
0.9275
0.9276
0.9277
0.9277
0.9277
0.9277
0.9278
0.9278
0.9278
0.9281
0.9281
0.9281
0.9282
0.9283
0.9283
0.9284
0.9284
0.9284
0.9284
0.9284
0.9284

0.9285
0.9287
0.9287
0.9289
0.9289
0.9289
0.9289
0.9290
0.9290
0.9290
0.9290
0.9290
0.9290
0.9291
0.9291
0.9291
0.9291
0.9292
0.9292
0.9293
0.9295
0.9295
0.9295
0.9295
0.9295
0.9295
0.9296
0.9296
0.9296
0.9296
0.9296
0.9296
0.9296
0.9296
0.9296
0.9296
0.9296
0.9299
0.9299
0.9299
0.9302
0.9302
0.9302
0.9303
0.9303
0.9304
0.9304
0.9304
0.9304
0.9304

0.9304
0.9304
0.9304
0.9304
0.9304
0.9304
0.9305
0.9306
0.9306
0.9306
0.9306
0.9306
0.9306
0.9306
0.9306
0.9306
0.9306
0.9306
0.9306
0.9306
0.9307
0.9307
0.9307
0.9309
0.9309
0.9310
0.9311
0.9312
0.9313
0.9316
0.9317
0.9319
0.9319
0.9319
0.9319
0.9319
0.9319
0.9320
0.9320
0.9320
0.9321
0.9321
0.9321
0.9321
0.9322
0.9322
0.9322
0.9322
0.9322
0.9323

0.9323
0.9323
0.9323
0.9323
0.9323
0.9324
0.9325
0.9325
0.9326
0.9326
0.9326
0.9326
0.9327
0.9327
0.9327
0.9327
0.9328
0.9329
0.9329
0.9329
0.9329
0.9329
0.9329
0.9329
0.9329
0.9329
0.9329
0.9329
0.9329
0.9329
0.9329
0.9329
0.9329
0.9329
0.9329
0.9329
0.9329
0.9329
0.9329
0.9329
0.9329
0.9329
0.9331
0.9331
0.9331
0.9331
0.9331
0.9331
0.9332
0.9332

0.9332
0.9335
0.9335
0.9335
0.9335
0.9335
0.9335
0.9335
0.9336
0.9338
0.9339
0.9339
0.9339
0.9339
0.9339
0.9339
0.9339
0.9339
0.9339
0.9339
0.9339
0.9339
0.9339
0.9339
0.9339
0.9341
0.9341
0.9342
0.9343
0.9344
0.9344
0.9344
0.9344
0.9344
0.9345
0.9345
0.9347
0.9347
0.9348
0.9348
0.9348
0.9354
0.9354
0.9354
0.9354
0.9356
0.9356
0.9356
0.9358
0.9360

0.9360
0.9360
0.9360
0.9361
0.9362
0.9362
0.9362
0.9362
0.9362
0.9362
0.9363
0.9365
0.9365
0.9365
0.9366
0.9367
0.9367
0.9367
0.9368
0.9369
0.9369
0.9369
0.9369
0.9369
0.9369
0.9369
0.9369
0.9369
0.9371
0.9371
0.9371
0.9371
0.9371
0.9371
0.9371
0.9371
0.9371
0.9371
0.9371
0.9371
0.9371
0.9371
0.9371
0.9371
0.9371
0.9371
0.9371
0.9372
0.9373
0.9373

0.9374
0.9374
0.9375
0.9376
0.9376
0.9376
0.9376
0.9376
0.9376
0.9376
0.9376
0.9377
0.9380
0.9381
0.9381
0.9381
0.9381
0.9382
0.9382
0.9382
0.9387
0.9388
0.9388
0.9388
0.9388
0.9388
0.9388
0.9388
0.9388
0.9388
0.9388
0.9389
0.9389
0.9389
0.9389
0.9389
0.9389
0.9389
0.9389
0.9389
0.9389
0.9389
0.9389
0.9389
0.9390
0.9391
0.9391
0.9391
0.9391
0.9391

0.9391
0.9391
0.9391
0.9391
0.9391
0.9391
0.9391
0.9391
0.9391
0.9391
0.9391
0.9391
0.9391
0.9391
0.9391
0.9391
0.9391
0.9391
0.9393
0.9393
0.9394
0.9394
0.9394
0.9394
0.9395
0.9396
0.9396
0.9396
0.9396
0.9396
0.9397
0.9397
0.9397
0.9397
0.9397
0.9397
0.9397
0.9397
0.9397
0.9397
0.9397
0.9397
0.9398
0.9400
0.9401
0.9403
0.9403
0.9403
0.9403
0.9403

0.9403
0.9403
0.9403
0.9403
0.9403
0.9403
0.9403
0.9403
0.9403
0.9403
0.9403
0.9403
0.9403
0.9404
0.9405
0.9406
0.9408
0.9408
0.9408
0.9410
0.9410
0.9410
0.9411
0.9411
0.9411
0.9411
0.9411
0.9411
0.9411
0.9412
0.9412
0.9412
0.9416
0.9416
0.9416
0.9416
0.9416
0.9416
0.9416
0.9416
0.9416
0.9416
0.9416
0.9416
0.9416
0.9416
0.9416
0.9416
0.9416
0.9416

0.9416
0.9416
0.9416
0.9416
0.9416
0.9416
0.9419
0.9419
0.9419
0.9419
0.9421
0.9422
0.9422
0.9422
0.9422
0.9422
0.9422
0.9422
0.9423
0.9423
0.9423
0.9423
0.9423
0.9423
0.9423
0.9423
0.9423
0.9423
0.9423
0.9423
0.9423
0.9423
0.9423
0.9423
0.9424
0.9424
0.9424
0.9425
0.9426
0.9426
0.9426
0.9426
0.9426
0.9426
0.9426
0.9426
0.9426
0.9426
0.9426
0.9426

0.9426
0.9426
0.9426
0.9426
0.9429
0.9429
0.9430
0.9430
0.9430
0.9430
0.9431
0.9431
0.9431
0.9432
0.9432
0.9432
0.9433
0.9434
0.9434
0.9434
0.9434
0.9434
0.9434
0.9434
0.9440
0.9441
0.9441
0.9441
0.9443
0.9443
0.9443
0.9443
0.9443
0.9443
0.9445
0.9445
0.9445
0.9446
0.9446
0.9446
0.9446
0.9446
0.9448
0.9448
0.9448
0.9448
0.9448
0.9448
0.9448
0.9448

0.9448
0.9448
0.9450
0.9450
0.9450
0.9451
0.9451
0.9452
0.9459
0.9459
0.9459
0.9459
0.9459
0.9459
0.9462
0.9462
0.9462
0.9462
0.9462
0.9462
0.9462
0.9462
0.9462
0.9462
0.9462
0.9462
0.9462
0.9462
0.9462
0.9462
0.9462
0.9462
0.9462
0.9462
0.9462
0.9462
0.9462
0.9462
0.9462
0.9462
0.9462
0.9462
0.9462
0.9462
0.9462
0.9462
0.9462
0.9462
0.9463
0.9463
0.9463
0.9463

0.9463
0.9463
0.9463
0.9463
0.9463
0.9465
0.9465
0.9465
0.9467
0.9469
0.9469
0.9469
0.9469
0.9469
0.9469
0.9469
0.9469
0.9469
0.9469
0.9469
0.9469
0.9470
0.9470
0.9470
0.9471
0.9472
0.9472
0.9474
0.9474
0.9474
0.9475
0.9477
0.9477
0.9477
0.9477
0.9477
0.9477
0.9477
0.9477
0.9477
0.9478
0.9481
0.9481
0.9481
0.9481
0.9482
0.9484
0.9484
0.9484
0.9485

0.9487
0.9490
0.9490
0.9490
0.9490
0.9490
0.9490
0.9490
0.9490
0.9490
0.9490
0.9490
0.9490
0.9490
0.9493
0.9494
0.9496
0.9496
0.9498
0.9498
0.9499
0.9499
0.9499
0.9500
0.9500
0.9500
0.9500
0.9501
0.9501
0.9501
0.9501
0.9501
0.9501
0.9501
0.9501
0.9501
0.9501
0.9501
0.9501
0.9501
0.9502
0.9502
0.9502
0.9502
0.9502
0.9503
0.9503
0.9503
0.9503
0.9510

0.9510
0.9510
0.9510
0.9510
0.9510
0.9510
0.9510
0.9510
0.9510
0.9510
0.9510
0.9510
0.9510
0.9514
0.9514
0.9514
0.9514
0.9515
0.9515
0.9518
0.9518
0.9518
0.9518
0.9518
0.9518
0.9518
0.9518
0.9518
0.9518
0.9518
0.9518
0.9518
0.9518
0.9518
0.9518
0.9522
0.9522
0.9522
0.9522
0.9522
0.9524
0.9524
0.9524
0.9525
0.9525
0.9525
0.9525
0.9525
0.9526
0.9526

0.9526
0.9526
0.9526
0.9526
0.9528
0.9528
0.9532
0.9533
0.9534
0.9535
0.9535
0.9535
0.9535
0.9535
0.9535
0.9535
0.9535
0.9536
0.9537
0.9537
0.9537
0.9537
0.9538
0.9538
0.9538
0.9538
0.9538
0.9539
0.9539
0.9539
0.9539
0.9540
0.9541
0.9543
0.9543
0.9543
0.9543
0.9543
0.9543
0.9544
0.9544
0.9544
0.9547
0.9547
0.9547
0.9548
0.9548
0.9548
0.9548
0.9550

0.9551
0.9553
0.9553
0.9553
0.9554
0.9554
0.9557
0.9559
0.9560
0.9560
0.9561
0.9561
0.9561
0.9561
0.9561
0.9561
0.9561
0.9562
0.9562
0.9562
0.9562
0.9562
0.9562
0.9563
0.9563
0.9563
0.9563
0.9564
0.9565
0.9566
0.9566
0.9567
0.9568
0.9568
0.9568
0.9571
0.9571
0.9571
0.9571
0.9571
0.9571
0.9571
0.9571
0.9571
0.9571
0.9571
0.9571
0.9571
0.9575
0.9575

0.9576
0.9578
0.9579
0.9579
0.9579
0.9580
0.9580
0.9581
0.9581
0.9581
0.9582
0.9582
0.9583
0.9583
0.9584
0.9584
0.9584
0.9584
0.9586
0.9586
0.9586
0.9587
0.9587
0.9587
0.9587
0.9587
0.9587
0.9587
0.9587
0.9587
0.9587
0.9587
0.9587
0.9587
0.9587
0.9593
0.9594
0.9594
0.9599
0.9599
0.9599
0.9601
0.9601
0.9602
0.9603
0.9603
0.9603
0.9603
0.9603
0.9603

0.9603
0.9603
0.9604
0.9607
0.9609
0.9610
0.9610
0.9612
0.9612
0.9615
0.9615
0.9615
0.9616
0.9619
0.9619
0.9619
0.9619
0.9619
0.9620
0.9620
0.9620
0.9620
0.9620
0.9620
0.9620
0.9620
0.9620
0.9620
0.9620
0.9620
0.9620
0.9620
0.9620
0.9620
0.9621
0.9621
0.9622
0.9622
0.9622
0.9622
0.9622
0.9622
0.9622
0.9622
0.9622
0.9622
0.9626
0.9626
0.9626
0.9626

0.9626
0.9626
0.9626
0.9626
0.9626
0.9626
0.9626
0.9626
0.9626
0.9626
0.9627
0.9627
0.9627
0.9627
0.9627
0.9627
0.9627
0.9627
0.9629
0.9629
0.9629
0.9629
0.9629
0.9629
0.9629
0.9630
0.9630
0.9630
0.9630
0.9631
0.9633
0.9633
0.9633
0.9636
0.9636
0.9637
0.9640
0.9641
0.9641
0.9641
0.9641
0.9641
0.9641
0.9641
0.9642
0.9643
0.9645
0.9646
0.9647
0.9647

0.9648
0.9648
0.9649
0.9650
0.9650
0.9650
0.9650
0.9654
0.9655
0.9656
0.9656
0.9656
0.9656
0.9656
0.9656
0.9657
0.9657
0.9657
0.9657
0.9657
0.9658
0.9658
0.9659
0.9659
0.9659
0.9659
0.9659
0.9659
0.9659
0.9659
0.9659
0.9659
0.9659
0.9661
0.9661
0.9663
0.9663
0.9663
0.9663
0.9663
0.9663
0.9663
0.9663
0.9663
0.9663
0.9663
0.9664
0.9665
0.9666
0.9666

0.9666
0.9666
0.9666
0.9666
0.9666
0.9666
0.9666
0.9666
0.9666
0.9666
0.9667
0.9667
0.9667
0.9667
0.9667
0.9667
0.9667
0.9667
0.9667
0.9667
0.9667
0.9667
0.9668
0.9668
0.9668
0.9668
0.9668
0.9668
0.9668
0.9668
0.9668
0.9668
0.9668
0.9668
0.9668
0.9668
0.9668
0.9668
0.9668
0.9668
0.9668
0.9668
0.9668
0.9669
0.9669
0.9669
0.9669
0.9669
0.9670
0.9670
0.9670

0.9670
0.9670
0.9670
0.9670
0.9672
0.9672
0.9673
0.9673
0.9673
0.9673
0.9673
0.9673
0.9673
0.9673
0.9673
0.9673
0.9674
0.9674
0.9674
0.9674
0.9674
0.9674
0.9674
0.9674
0.9674
0.9674
0.9674
0.9674
0.9674
0.9674
0.9675
0.9675
0.9676
0.9676
0.9678
0.9680
0.9681
0.9682
0.9683
0.9683
0.9683
0.9683
0.9683
0.9683
0.9683
0.9683
0.9684
0.9685
0.9685
0.9685

0.9685
0.9685
0.9685
0.9685
0.9685
0.9685
0.9685
0.9685
0.9685
0.9688
0.9688
0.9688
0.9689
0.9691
0.9691
0.9691
0.9693
0.9693
0.9694
0.9694
0.9694
0.9694
0.9694
0.9695
0.9695
0.9696
0.9702
0.9704
0.9704
0.9708
0.9708
0.9708
0.9708
0.9709
0.9714
0.9714
0.9714
0.9714
0.9715
0.9716
0.9716
0.9716
0.9717
0.9717
0.9717
0.9717
0.9718
0.9719
0.9719
0.9719

0.9720
0.9720
0.9720
0.9720
0.9720
0.9720
0.9722
0.9722
0.9722
0.9722
0.9722
0.9722
0.9723
0.9723
0.9723
0.9725
0.9725
0.9726
0.9727
0.9727
0.9727
0.9728
0.9729
0.9730
0.9730
0.9730
0.9731
0.9731
0.9731
0.9731
0.9731
0.9731
0.9731
0.9731
0.9731
0.9731
0.9732
0.9736
0.9737
0.9737
0.9738
0.9738
0.9738
0.9738
0.9738
0.9738
0.9738
0.9738
0.9741
0.9745

0.9745
0.9747
0.9747
0.9748
0.9748
0.9749
0.9750
0.9750
0.9751
0.9751
0.9751
0.9751
0.9751
0.9751
0.9751
0.9751
0.9751
0.9753
0.9754
0.9754
0.9755
0.9755
0.9755
0.9755
0.9755
0.9755
0.9755
0.9755
0.9755
0.9755
0.9755
0.9756
0.9756
0.9756
0.9756
0.9756
0.9756
0.9756
0.9756
0.9756
0.9757
0.9757
0.9757
0.9757
0.9759
0.9759
0.9759
0.9759
0.9759
0.9759

0.9759
0.9759
0.9759
0.9759
0.9759
0.9761
0.9761
0.9763
0.9764
0.9764
0.9764
0.9764
0.9764
0.9764
0.9764
0.9764
0.9764
0.9764
0.9764
0.9764
0.9764
0.9764
0.9765
0.9767
0.9767
0.9767
0.9767
0.9767
0.9767
0.9767
0.9767
0.9767
0.9767
0.9767
0.9767
0.9767
0.9768
0.9768
0.9770
0.9771
0.9772
0.9773
0.9773
0.9773
0.9775
0.9776
0.9776
0.9777
0.9777
0.9779

0.9779
0.9780
0.9781
0.9782
0.9782
0.9782
0.9782
0.9782
0.9782
0.9782
0.9782
0.9782
0.9782
0.9782
0.9783
0.9784
0.9784
0.9784
0.9787
0.9787
0.9787
0.9787
0.9789
0.9789
0.9790
0.9790
0.9790
0.9790
0.9790
0.9791
0.9791
0.9792
0.9792
0.9793
0.9794
0.9795
0.9796
0.9796
0.9796
0.9796
0.9796
0.9797
0.9797
0.9797
0.9797
0.9797
0.9797
0.9797
0.9797
0.9798

0.9798
0.9798
0.9798
0.9798
0.9798
0.9798
0.9799
0.9799
0.9800
0.9800
0.9800
0.9804
0.9805
0.9805
0.9805
0.9806
0.9806
0.9808
0.9808
0.9808
0.9808
0.9808
0.9808
0.9808
0.9808
0.9811
0.9812
0.9813
0.9814
0.9819
0.9819
0.9819
0.9819
0.9819
0.9819
0.9819
0.9819
0.9819
0.9819
0.9819
0.9819
0.9819
0.9819
0.9819
0.9819
0.9819
0.9819
0.9819
0.9819
0.9819

0.9819
0.9819
0.9819
0.9819
0.9819
0.9819
0.9819
0.9819
0.9819
0.9819
0.9819
0.9819
0.9819
0.9819
0.9819
0.9819
0.9819
0.9819
0.9819
0.9819
0.9819
0.9819
0.9820
0.9820
0.9820
0.9822
0.9825
0.9825
0.9825
0.9825
0.9828
0.9828
0.9828
0.9830
0.9835
0.9835
0.9835
0.9835
0.9836
0.9837
0.9837
0.9838
0.9838
0.9838
0.9838
0.9838
0.9838
0.9838
0.9838
0.9838

0.9838
0.9838
0.9842
0.9842
0.9842
0.9842
0.9843
0.9844
0.9844
0.9845
0.9846
0.9846
0.9846
0.9847
0.9847
0.9847
0.9847
0.9847
0.9848
0.9856
0.9856
0.9856
0.9857
0.9857
0.9857
0.9858
0.9858
0.9858
0.9861
0.9862
0.9862
0.9862
0.9863
0.9863
0.9863
0.9863
0.9863
0.9863
0.9863
0.9863
0.9863
0.9863
0.9863
0.9863
0.9865
0.9865
0.9866
0.9866
0.9866
0.9867

0.9867
0.9867
0.9867
0.9867
0.9869
0.9869
0.9871
0.9871
0.9871
0.9873
0.9873
0.9873
0.9873
0.9873
0.9877
0.9878
0.9878
0.9878
0.9879
0.9879
0.9880
0.9880
0.9880
0.9880
0.9880
0.9880
0.9880
0.9880
0.9880
0.9880
0.9880
0.9880
0.9880
0.9880
0.9880
0.9880
0.9880
0.9880
0.9880
0.9880
0.9880
0.9880
0.9880
0.9880
0.9880
0.9880
0.9880
0.9880
0.9880
0.9880

0.9880
0.9880
0.9880
0.9880
0.9880
0.9880
0.9880
0.9880
0.9880
0.9880
0.9880
0.9880
0.9880
0.9880
0.9880
0.9880
0.9880
0.9880
0.9880
0.9880
0.9881
0.9882
0.9882
0.9882
0.9882
0.9882
0.9882
0.9882
0.9882
0.9882
0.9882
0.9882
0.9882
0.9882
0.9882
0.9882
0.9884
0.9884
0.9886
0.9887
0.9890
0.9890
0.9892
0.9892
0.9892
0.9892
0.9892
0.9892
0.9892
0.9892

0.9892
0.9892
0.9892
0.9892
0.9892
0.9892
0.9892
0.9892
0.9892
0.9892
0.9894
0.9895
0.9896
0.9896
0.9896
0.9896
0.9896
0.9897
0.9898
0.9898
0.9898
0.9898
0.9898
0.9899
0.9899
0.9900
0.9900
0.9901
0.9904
0.9904
0.9905
0.9905
0.9905
0.9905
0.9905
0.9906
0.9907
0.9907
0.9909
0.9909
0.9910
0.9910
0.9910
0.9911
0.9911
0.9911
0.9913
0.9914
0.9916
0.9918

0.9918
0.9918
0.9919
0.9922
0.9922
0.9922
0.9924
0.9924
0.9925
0.9925
0.9925
0.9925
0.9925
0.9928
0.9928
0.9928
0.9928
0.9928
0.9929
0.9929
0.9929
0.9930
0.9931
0.9931
0.9931
0.9932
0.9932
0.9932
0.9932
0.9932
0.9936
0.9936
0.9936
0.9936
0.9936
0.9936
0.9938
0.9938
0.9938
0.9938
0.9938
0.9938
0.9940
0.9940
0.9940
0.9940
0.9940
0.9940
0.9940
0.9940

0.9940
0.9940
0.9941
0.9941
0.9941
0.9941
0.9943
0.9943
0.9943
0.9943
0.9944
0.9945
0.9945
0.9945
0.9945
0.9945
0.9946
0.9947
0.9947
0.9947
0.9947
0.9947
0.9947
0.9947
0.9949
0.9949
0.9949
0.9949
0.9949
0.9949
0.9949
0.9949
0.9951
0.9951
0.9952
0.9952
0.9952
0.9952
0.9952
0.9952
0.9952
0.9952
0.9952
0.9952
0.9952
0.9952
0.9953
0.9955
0.9955
0.9956

0.9957
0.9957
0.9957
0.9957
0.9957
0.9957
0.9957
0.9957
0.9957
0.9957
0.9957
0.9957
0.9957
0.9957
0.9957
0.9958
0.9958
0.9958
0.9959
0.9959
0.9959
0.9959
0.9959
0.9959
0.9960
0.9960
0.9961
0.9962
0.9962
0.9964
0.9964
0.9964
0.9964
0.9964
0.9964
0.9964
0.9964
0.9964
0.9964
0.9964
0.9964
0.9964
0.9966
0.9966
0.9966
0.9966
0.9966
0.9966
0.9966
0.9966

0.9967
0.9969
0.9970
0.9971
0.9972
0.9972
0.9972
0.9972
0.9972
0.9974
0.9974
0.9974
0.9974
0.9974
0.9974
0.9974
0.9975
0.9975
0.9975
0.9975
0.9975
0.9975
0.9975
0.9977
0.9977
0.9977
0.9978
0.9980
0.9980
0.9981
0.9981
0.9981
0.9982
0.9982
0.9982
0.9982
0.9982
0.9983
0.9983
0.9983
0.9983
0.9983
0.9983
0.9983
0.9983
0.9983
0.9983
0.9983
0.9983
0.9984

0.9989
0.9989
0.9989
0.9989
0.9989
0.9989
0.9989
0.9989
0.9991
0.9991
0.9991
0.9991
0.9992
0.9992
0.9992
0.9992
0.9995
0.9995
0.9995
0.9995
0.9996
0.9997
0.9997
0.9997
0.9997
0.9997
0.9997
0.9997
0.9997
0.9999
0.9999
0.9999
1.0000
