# Supplementary material for: Whole-Blood Transcriptional Profiles Enable Early Prediction of the Presence of Coronary Atherosclerosis and High-Risk Plaque Features at Coronary CT Angiography
Source: Biomedicines. 2022 Jun 2;10(6):1309. doi: 10.3390/biomedicines10061309 (PMC9219643; doi:10.3390/biomedicines10061309)
Supplement: Supplementary file 1 [file biomedicines-10-01309-s001.zip › Supplementary Table S2.pdf]

Supplementary Table S2. *Results of functional enrichment analysis*

CAD.vs.noCAD

NAME	Gene Ontology ID	SIZE	ES	NES	NOM p-val	q-value
CYTOPLASMIC TRANSLATION	GO:0002181	107	0.59	7.37	0.00E+00	0.00E+00
RRNA PROCESSING	GO:0006364	160	0.33	4.77	0.00E+00	0.00E+00
RRNA METABOLIC PROCESS	GO:0016072	168	0.31	4.71	0.00E+00	0.00E+00
OXIDATIVE PHOSPHORYLATION	GO:0006119	89	0.36	4.04	0.00E+00	0.00E+00
RIBOSOMAL LARGE SUBUNIT BIOGENESIS	GO:0042273	66	0.40	3.79	0.00E+00	0.00E+00
RIBOSOME ASSEMBLY	GO:0042255	51	0.43	3.73	0.00E+00	0.00E+00
ATP METABOLIC PROCESS	GO:0046034	139	0.26	3.66	0.00E+00	0.00E+00
MITOCHONDRIAL ATP SYNTHESIS COUPLED ELECTRON TRANSPORT	GO:0042775	66	0.38	3.66	0.00E+00	0.00E+00
AEROBIC ELECTRON TRANSPORT CHAIN	GO:0019646	64	0.38	3.64	0.00E+00	0.00E+00
ATP SYNTHESIS COUPLED ELECTRON TRANSPORT	GO:0042773	66	0.38	3.55	0.00E+00	0.00E+00
AEROBIC RESPIRATION	GO:0009060	115	0.28	3.54	0.00E+00	0.00E+00
CELLULAR RESPIRATION	GO:0045333	128	0.26	3.45	0.00E+00	0.00E+00
MITOCHONDRIAL RESPIRATORY CHAIN COMPLEX ASSEMBLY	GO:0033108	87	0.31	3.39	0.00E+00	0.00E+00
RESPIRATORY ELECTRON TRANSPORT CHAIN	GO:0022904	75	0.32	3.37	0.00E+00	0.00E+00
MITOCHONDRIAL ELECTRON TRANSPORT, NADH TO UBIQUINONE	GO:0006120	40	0.45	3.37	0.00E+00	0.00E+00
NADH DEHYDROGENASE COMPLEX ASSEMBLY	GO:0010257	56	0.38	3.36	0.00E+00	0.00E+00
MITOCHONDRIAL RESPIRATORY CHAIN COMPLEX I ASSEMBLY	GO:0032981	56	0.38	3.32	0.00E+00	0.00E+00
ELECTRON TRANSPORT CHAIN	GO:0022900	80	0.31	3.28	0.00E+00	0.00E+00
RIBOSOMAL SMALL SUBUNIT BIOGENESIS	GO:0042274	63	0.35	3.22	0.00E+00	0.00E+00
HUMORAL IMMUNE RESPONSE MEDIATED BY CIRCULATING IMMUNOGLOBULIN	GO:0002455	59	0.34	3.13	0.00E+00	3.45E-05
COMPLEMENT ACTIVATION, CLASSICAL PATHWAY	GO:0006958	57	0.34	3.09	0.00E+00	4.83E-05
ENERGY DERIVATION BY OXIDATION OF ORGANIC COMPOUNDS	GO:0015980	149	0.22	3.08	0.00E+00	4.61E-05
HUMORAL IMMUNE RESPONSE	GO:0006959	140	0.22	3.02	0.00E+00	4.41E-05
PHAGOCYTOSIS, RECOGNITION	GO:0006910	61	0.33	3.00	0.00E+00	4.22E-05
COMPLEMENT ACTIVATION	GO:0006956	70	0.30	2.96	0.00E+00	6.74E-05
RIBONUCLEOPROTEIN COMPLEX ASSEMBLY	GO:0022618	154	0.20	2.95	0.00E+00	7.73E-05
RIBONUCLEOPROTEIN COMPLEX SUBUNIT ORGANIZATION	GO:0071826	160	0.20	2.83	0.00E+00	1.73E-04
B CELL MEDIATED IMMUNITY	GO:0019724	79	0.26	2.69	0.00E+00	5.32E-04
IMMUNOGLOBULIN MEDIATED IMMUNE RESPONSE	GO:0016064	76	0.25	2.62	0.00E+00	7.24E-04

ADAPTIVE IMMUNE RESPONSE BASED ON SOMATIC RECOMBINATION OF IMMUNE RECEPTORS BUILT FROM IMMUNOGLOBULIN SUPERFAMILY DOMAINS	GO:0002460	108	0.21	2.53	0.00E+00	1.55E-03
MATURATION OF SSU-RRNA	GO:0030490	41	0.33	2.49	0.00E+00	1.86E-03
MITOCHONDRIAL TRANSPORT	GO:0006839	162	0.17	2.49	0.00E+00	1.85E-03
NUCLEOSIDE TRIPHOSPHATE BIOSYNTHETIC PROCESS	GO:0009142	37	0.34	2.49	0.00E+00	1.84E-03
PHAGOCYTOSIS, ENGULFMENT	GO:0006911	84	0.22	2.37	0.00E+00	3.67E-03
SPLICEOSOMAL SNRNP ASSEMBLY	GO:0000387	35	0.33	2.32	0.00E+00	5.20E-03
CELL RECOGNITION	GO:0008037	103	0.20	2.30	0.00E+00	5.91E-03
PLASMA MEMBRANE INVAGINATION	GO:0099024	91	0.20	2.25	2.08E-03	7.72E-03
LYMPHOCYTE MEDIATED IMMUNITY	GO:0002449	108	0.18	2.21	2.00E-03	9.68E-03
RIBONUCLEOSIDE TRIPHOSPHATE BIOSYNTHETIC PROCESS	GO:0009201	31	0.33	2.21	4.02E-03	9.59E-03
ESTABLISHMENT OF PROTEIN LOCALIZATION TO MITOCHONDRION	GO:0072655	60	0.24	2.12	0.00E+00	1.74E-02
RRNA MODIFICATION	GO:0000154	31	0.31	2.11	0.00E+00	1.79E-02
TRNA PROCESSING	GO:0008033	103	0.18	2.07	1.98E-03	2.19E-02
B CELL RECEPTOR SIGNALING PATHWAY	GO:0050853	88	0.19	2.04	2.03E-03	2.59E-02
PURINE NUCLEOSIDE DIPHOSPHATE METABOLIC PROCESS	GO:0009135	32	0.29	2.03	4.23E-03	2.70E-02
MEMBRANE INVAGINATION	GO:0010324	98	0.17	1.99	8.06E-03	3.40E-02
PROTON TRANSMEMBRANE TRANSPORT	GO:1902600	41	0.27	1.99	3.94E-03	3.36E-02
POSITIVE REGULATION OF B CELL ACTIVATION	GO:0050871	102	0.17	1.98	2.02E-03	3.36E-02
MITOCHONDRIAL TRANSMEMBRANE TRANSPORT	GO:1990542	79	0.19	1.97	1.44E-02	3.52E-02
MITOCHONDRIAL TRANSLATION	GO:0032543	47	0.24	1.94	6.30E-03	4.07E-02
MITOCHONDRIAL MEMBRANE ORGANIZATION	GO:0007006	81	0.18	1.92	1.02E-02	4.72E-02
PROTEIN TARGETING TO MITOCHONDRION	GO:0006626	50	0.23	1.90	2.02E-02	5.09E-02
NUCLEOTIDE PHOSPHORYLATION	GO:0046939	31	0.28	1.88	6.41E-03	5.53E-02
PURINE RIBONUCLEOSIDE DIPHOSPHATE METABOLIC PROCESS	GO:0009179	31	0.28	1.87	1.22E-02	5.75E-02
TRNA METABOLIC PROCESS	GO:0006399	147	0.13	1.86	1.00E-02	5.99E-02
LEUKOCYTE MEDIATED IMMUNITY	GO:0002443	125	0.14	1.82	3.84E-03	7.14E-02
PROTEIN LOCALIZATION TO MITOCHONDRION	GO:0070585	65	0.19	1.80	8.05E-03	7.79E-02
HEXOSE METABOLIC PROCESS	GO:0019318	63	0.19	1.80	1.72E-02	7.75E-02
PROTEIN FOLDING	GO:0006457	145	0.13	1.79	1.83E-02	8.09E-02
MITOCHONDRIAL GENE EXPRESSION	GO:0140053	78	0.17	1.75	2.29E-02	9.93E-02
NUCLEOSIDE DIPHOSPHATE PHOSPHORYLATION	GO:0006165	30	0.26	1.75	1.71E-02	9.92E-02
ADAPTIVE IMMUNE RESPONSE	GO:0002250	130	0.13	1.72	2.12E-02	1.13E-01

MATURATION OF 5.8S RRNA	GO:0000460	31	0.25	1.70	3.07E-02	1.25E-01
PROTEIN INSERTION INTO MEMBRANE	GO:0051205	43	0.21	1.69	2.42E-02	1.25E-01
GLUTAMINE FAMILY AMINO ACID METABOLIC PROCESS	GO:0009064	32	0.25	1.67	3.09E-02	1.40E-01
REGULATION OF MITOCHONDRIAL MEMBRANE PERMEABILITY	GO:0046902	39	0.22	1.66	2.75E-02	1.43E-01
CYTOCHROME COMPLEX ASSEMBLY	GO:0017004	32	0.25	1.66	3.31E-02	1.43E-01
TRANSLATIONAL INITIATION	GO:0006413	46	0.21	1.64	3.81E-02	1.48E-01
MONOSACCHARIDE METABOLIC PROCESS	GO:0005996	73	0.16	1.64	2.61E-02	1.47E-01
RIBONUCLEOSIDE DIPHOSPHATE METABOLIC PROCESS	GO:0009185	34	0.23	1.63	3.67E-02	1.55E-01
REGULATION OF MEMBRANE PERMEABILITY	GO:0090559	45	0.21	1.63	4.17E-02	1.55E-01
NUCLEOSIDE DIPHOSPHATE METABOLIC PROCESS	GO:0009132	42	0.21	1.60	4.82E-02	1.74E-01
RESPONSE TO HEXOSE	GO:0009746	38	0.20	1.52	4.64E-02	2.48E-01
GLUCOSE METABOLIC PROCESS	GO:0006006	42	0.19	1.51	7.47E-02	2.57E-01
RNA MODIFICATION	GO:0009451	137	0.11	1.50	6.17E-02	2.66E-01
RESPONSE TO MONOSACCHARIDE	GO:0034284	38	0.20	1.49	5.97E-02	2.72E-01
PURINE RIBONUCLEOSIDE TRIPHOSPHATE METABOLIC PROCESS	GO:0009205	39	0.20	1.45	8.98E-02	3.13E-01
NEGATIVE REGULATION OF UBIQUITIN-DEPENDENT PROTEIN CATABOLIC PROCESS	GO:2000059	39	0.20	1.45	9.29E-02	3.16E-01
PROTEIN TRANSMEMBRANE IMPORT INTO INTRACELLULAR ORGANELLE	GO:0044743	30	0.21	1.41	9.36E-02	3.63E-01
ANTIMICROBIAL HUMORAL RESPONSE	GO:0019730	48	0.17	1.41	1.24E-01	3.68E-01
DNA-TEMPLATED TRANSCRIPTION, ELONGATION	GO:0006354	35	0.20	1.40	1.15E-01	3.72E-01
CELLULAR RESPONSE TO HEAT	GO:0034605	36	0.20	1.40	1.08E-01	3.72E-01
NUCLEOSIDE TRIPHOSPHATE METABOLIC PROCESS	GO:0009141	55	0.16	1.37	1.20E-01	4.08E-01
NUCLEOTIDE CATABOLIC PROCESS	GO:0009166	44	0.17	1.37	1.25E-01	4.05E-01
AMINO ACID ACTIVATION	GO:0043038	38	0.19	1.37	1.20E-01	4.07E-01
TRNA AMINOACYLATION	GO:0043039	38	0.19	1.37	1.32E-01	4.04E-01
DICARBOXYLIC ACID METABOLIC PROCESS	GO:0043648	54	0.16	1.36	1.26E-01	4.07E-01
AMINO ACID TRANSPORT	GO:0006865	48	0.17	1.35	1.21E-01	4.12E-01
RESPONSE TO GLUCOSE	GO:0009749	36	0.19	1.33	1.26E-01	4.49E-01
REGULATION OF MITOCHONDRIAL MEMBRANE POTENTIAL	GO:0051881	39	0.18	1.33	1.33E-01	4.45E-01
NCRNA 3'-END PROCESSING	GO:0043628	35	0.19	1.33	1.57E-01	4.42E-01
REACTIVE OXYGEN SPECIES METABOLIC PROCESS	GO:0072593	51	0.16	1.33	1.45E-01	4.40E-01
PROTEIN TARGETING	GO:0006605	185	0.08	1.31	1.50E-01	4.59E-01
POSITIVE REGULATION OF INTRINSIC APOPTOTIC SIGNALING PATHWAY	GO:2001244	35	0.19	1.29	1.59E-01	4.97E-01
RNA METHYLATION	GO:0001510	69	0.13	1.29	1.78E-01	4.93E-01

DEFENSE RESPONSE TO BACTERIUM	GO:0042742	148	0.09	1.28	1.75E-01	5.01E-01
PYRUVATE METABOLIC PROCESS	GO:0006090	36	0.18	1.28	1.79E-01	4.97E-01
RESPONSE TO CARBOHYDRATE	GO:0009743	43	0.16	1.28	1.52E-01	4.93E-01
RIBONUCLEOSIDE TRIPHOSPHATE METABOLIC PROCESS	GO:0009199	43	0.16	1.27	1.71E-01	5.04E-01
POSITIVE REGULATION OF TRANSLATION	GO:0045727	98	0.11	1.27	1.90E-01	4.99E-01
RESPONSE TO TEMPERATURE STIMULUS	GO:0009266	69	0.13	1.27	1.73E-01	4.94E-01
TRNA MODIFICATION	GO:0006400	73	0.13	1.27	1.79E-01	4.92E-01
RESPONSE TO HEAT	GO:0009408	50	0.15	1.26	1.99E-01	5.12E-01
REGULATION OF GENERATION OF PRECURSOR METABOLITES AND ENERGY	GO:0043467	81	0.12	1.25	1.76E-01	5.21E-01
SMALL MOLECULE CATABOLIC PROCESS	GO:0044282	200	0.07	1.24	1.93E-01	5.37E-01
VIRION ASSEMBLY	GO:0019068	31	0.18	1.23	2.17E-01	5.48E-01
SNRNA METABOLIC PROCESS	GO:0016073	36	0.17	1.23	2.08E-01	5.52E-01
TRNA AMINOACYLATION FOR PROTEIN TRANSLATION	GO:0006418	35	0.18	1.22	2.31E-01	5.50E-01
RENAL SYSTEM PROCESS	GO:0003014	40	0.16	1.22	2.21E-01	5.54E-01
ANTIMICROBIAL HUMORAL IMMUNE RESPONSE MEDIATED BY ANTIMICROBIAL PEPTIDE	GO:0061844	32	0.18	1.20	2.35E-01	5.81E-01
TETRAPYRROLE METABOLIC PROCESS	GO:0033013	37	0.17	1.20	2.58E-01	5.80E-01
REGULATION OF B CELL ACTIVATION	GO:0050864	133	0.09	1.20	2.47E-01	5.75E-01
PIGMENT METABOLIC PROCESS	GO:0042440	37	0.17	1.20	2.16E-01	5.72E-01
POSITIVE REGULATION OF ESTABLISHMENT OF PROTEIN LOCALIZATION TO MITOCHONDRION	GO:1903749	33	0.17	1.19	2.39E-01	5.90E-01
NEGATIVE REGULATION OF PROTEOLYSIS INVOLVED IN CELLULAR PROTEIN CATABOLIC PROCESS	GO:1903051	49	0.15	1.19	2.42E-01	5.86E-01
INTRACELLULAR PROTEIN TRANSMEMBRANE TRANSPORT	GO:0065002	42	0.15	1.19	2.27E-01	5.82E-01
INNER MITOCHONDRIAL MEMBRANE ORGANIZATION	GO:0007007	32	0.18	1.18	2.37E-01	5.83E-01
STEROID BIOSYNTHETIC PROCESS	GO:0006694	53	0.14	1.18	2.43E-01	5.80E-01
PLATELET AGGREGATION	GO:0070527	30	0.18	1.18	2.39E-01	5.77E-01
RETINA HOMEOSTASIS	GO:0001895	36	0.16	1.18	2.46E-01	5.78E-01
CELLULAR KETONE METABOLIC PROCESS	GO:0042180	43	0.15	1.18	2.49E-01	5.76E-01
CELLULAR AMINO ACID METABOLIC PROCESS	GO:0006520	158	0.08	1.16	2.71E-01	6.04E-01
CHAPERONE-MEDIATED PROTEIN FOLDING	GO:0061077	50	0.14	1.16	2.85E-01	6.02E-01
NEGATIVE REGULATION OF PROTEASOMAL PROTEIN CATABOLIC PROCESS	GO:1901799	37	0.16	1.16	2.42E-01	5.98E-01
NEGATIVE REGULATION OF PROTEIN CATABOLIC PROCESS	GO:0042177	84	0.11	1.16	2.44E-01	5.96E-01

TRNA METHYLATION	GO:0030488	33	0.17	1.15	2.55E-01	6.01E-01
TRANSCRIPTION ELONGATION FROM RNA POLYMERASE II PROMOTER	GO:0006368	33	0.17	1.15	2.61E-01	5.98E-01
NUCLEOSIDE PHOSPHATE CATABOLIC PROCESS	GO:1901292	51	0.13	1.15	2.70E-01	5.98E-01
ORGANIC ACID CATABOLIC PROCESS	GO:0016054	136	0.09	1.15	2.53E-01	5.97E-01
PURINE NUCLEOSIDE TRIPHOSPHATE METABOLIC PROCESS	GO:0009144	42	0.15	1.14	2.64E-01	6.08E-01
REGULATION OF UBIQUITIN-PROTEIN TRANSFERASE ACTIVITY	GO:0051438	43	0.15	1.13	2.93E-01	6.18E-01
FATTY ACID OXIDATION	GO:0019395	55	0.13	1.13	2.82E-01	6.14E-01
VIRAL GENE EXPRESSION	GO:0019080	47	0.14	1.13	3.04E-01	6.14E-01
CHROMATIN ASSEMBLY OR DISASSEMBLY	GO:0006333	82	0.10	1.13	2.98E-01	6.17E-01
CARBOXYLIC ACID CATABOLIC PROCESS	GO:0046395	133	0.08	1.13	2.90E-01	6.14E-01
REGULATION OF CHOLESTEROL TRANSPORT	GO:0032374	31	0.17	1.13	2.96E-01	6.12E-01
REGULATION OF STEROL TRANSPORT	GO:0032371	31	0.17	1.12	3.10E-01	6.09E-01
NCRNA TRANSCRIPTION	GO:0098781	31	0.17	1.12	3.24E-01	6.10E-01
NEGATIVE REGULATION OF RESPONSE TO ENDOPLASMIC RETICULUM STRESS	GO:1903573	35	0.16	1.12	3.21E-01	6.16E-01
ALPHA-AMINO ACID METABOLIC PROCESS	GO:1901605	89	0.10	1.12	3.20E-01	6.15E-01
MITOCHONDRIAL RNA METABOLIC PROCESS	GO:0000959	41	0.15	1.10	3.24E-01	6.38E-01
REGULATION OF TOR SIGNALING	GO:0032006	80	0.10	1.10	3.22E-01	6.46E-01
SENSORY PERCEPTION OF MECHANICAL STIMULUS	GO:0050954	41	0.14	1.10	3.24E-01	6.42E-01
HOMOTYPIC CELL-CELL ADHESION	GO:0034109	34	0.16	1.09	3.28E-01	6.38E-01
POSITIVE REGULATION OF CELLULAR AMIDE METABOLIC PROCESS	GO:0034250	118	0.09	1.09	3.34E-01	6.51E-01
REGULATION OF ATP METABOLIC PROCESS	GO:1903578	53	0.12	1.08	3.24E-01	6.59E-01
CHROMATIN ORGANIZATION	GO:0006325	183	0.07	1.08	3.42E-01	6.60E-01
REGULATION OF T CELL RECEPTOR SIGNALING PATHWAY	GO:0050856	33	0.16	1.08	3.52E-01	6.56E-01
NEGATIVE REGULATION OF PROTEIN BINDING	GO:0032091	61	0.12	1.08	3.29E-01	6.55E-01
POSITIVE REGULATION OF TOR SIGNALING	GO:0032008	38	0.15	1.07	3.64E-01	6.56E-01
ANTIGEN RECEPTOR-MEDIATED SIGNALING PATHWAY	GO:0050851	151	0.07	1.07	3.69E-01	6.56E-01
PROTEIN TRANSMEMBRANE TRANSPORT	GO:0071806	43	0.14	1.07	3.59E-01	6.62E-01
PROTEIN K63-LINKED UBIQUITINATION	GO:0070534	39	0.15	1.06	3.69E-01	6.62E-01
PLATELET ACTIVATION	GO:0030168	60	0.12	1.06	3.60E-01	6.62E-01
LIPID OXIDATION	GO:0034440	58	0.12	1.06	3.56E-01	6.62E-01
BASE-EXCISION REPAIR	GO:0006284	33	0.16	1.05	3.77E-01	6.68E-01
REGULATION OF PROTEIN TARGETING	GO:1903533	59	0.12	1.05	3.96E-01	6.82E-01
PYRIMIDINE-CONTAINING COMPOUND METABOLIC PROCESS	GO:0072527	51	0.13	1.04	3.82E-01	6.80E-01

REGULATION OF PURINE NUCLEOTIDE METABOLIC PROCESS	GO:1900542	45	0.13	1.04	3.75E-01	6.82E-01
OLEFINIC COMPOUND METABOLIC PROCESS	GO:0120254	53	0.12	1.04	3.81E-01	6.82E-01
CARBOHYDRATE METABOLIC PROCESS	GO:0005975	196	0.06	1.04	4.04E-01	6.84E-01
CARBOHYDRATE BIOSYNTHETIC PROCESS	GO:0016051	54	0.12	1.02	4.01E-01	7.08E-01
PROTEIN MATURATION	GO:0051604	156	0.07	1.02	4.14E-01	7.15E-01
REGULATION OF INSULIN SECRETION	GO:0050796	56	0.12	1.01	3.98E-01	7.18E-01
NUCLEOSOME ORGANIZATION	GO:0034728	80	0.10	1.01	4.16E-01	7.14E-01
REGULATION OF TRANSCRIPTION BY RNA POLYMERASE I	GO:0006356	30	0.16	1.01	4.33E-01	7.13E-01
NUCLEIC ACID PHOSPHODIESTER BOND HYDROLYSIS	GO:0090305	98	0.09	1.00	4.41E-01	7.24E-01
TELOMERE MAINTENANCE	GO:0000723	64	0.11	1.00	4.12E-01	7.20E-01
REGULATION OF FIBROBLAST PROLIFERATION	GO:0048145	36	0.14	1.00	4.30E-01	7.18E-01
CELL KILLING	GO:0001906	40	0.13	1.00	4.18E-01	7.23E-01
REGULATION OF NUCLEOTIDE METABOLIC PROCESS	GO:0006140	46	0.12	1.00	4.35E-01	7.23E-01
POSITIVE REGULATION OF PROTEIN LOCALIZATION TO NUCLEUS	GO:1900182	55	0.12	0.99	4.47E-01	7.28E-01
MONOCARBOXYLIC ACID CATABOLIC PROCESS	GO:0072329	74	0.10	0.99	4.74E-01	7.34E-01
PROTEIN TARGETING TO MEMBRANE	GO:0006612	70	0.10	0.98	4.47E-01	7.35E-01
NEGATIVE REGULATION OF CELLULAR PROTEIN CATABOLIC PROCESS	GO:1903363	59	0.11	0.98	4.64E-01	7.40E-01
FATTY ACID CATABOLIC PROCESS	GO:0009062	66	0.10	0.98	4.80E-01	7.45E-01
CARBOHYDRATE CATABOLIC PROCESS	GO:0016052	61	0.11	0.97	4.72E-01	7.47E-01
DEFENSE RESPONSE TO GRAM-NEGATIVE BACTERIUM	GO:0050829	36	0.13	0.96	5.10E-01	7.79E-01
DNA MODIFICATION	GO:0006304	57	0.11	0.95	5.01E-01	7.81E-01
SENSORY PERCEPTION OF SOUND	GO:0007605	39	0.13	0.95	5.08E-01	7.79E-01
ORGANOPHOSPHATE CATABOLIC PROCESS	GO:0046434	82	0.09	0.95	5.01E-01	7.78E-01
TUMOR NECROSIS FACTOR-MEDIATED SIGNALING PATHWAY	GO:0033209	33	0.14	0.95	5.01E-01	7.73E-01
REGULATION OF ESTABLISHMENT OF PROTEIN LOCALIZATION TO MITOCHONDRION	GO:1903747	42	0.13	0.94	5.14E-01	7.80E-01
EPITHELIAL TUBE FORMATION	GO:0072175	31	0.14	0.94	5.25E-01	7.78E-01
FATTY ACID BETA-OXIDATION	GO:0006635	45	0.12	0.94	5.10E-01	7.79E-01
POSITIVE REGULATION OF APOPTOTIC SIGNALING PATHWAY	GO:2001235	66	0.10	0.94	5.21E-01	7.79E-01
NEGATIVE REGULATION OF HISTONE MODIFICATION	GO:0031057	30	0.14	0.93	5.37E-01	7.85E-01
APOPTOTIC MITOCHONDRIAL CHANGES	GO:0008637	39	0.13	0.93	5.18E-01	7.82E-01
INTRINSIC APOPTOTIC SIGNALING PATHWAY BY P53 CLASS MEDIATOR	GO:0072332	31	0.14	0.93	5.37E-01	7.83E-01
REGULATION OF MEMBRANE POTENTIAL	GO:0042391	144	0.07	0.93	5.59E-01	7.87E-01
MORPHOGENESIS OF EMBRYONIC EPITHELIUM	GO:0016331	30	0.14	0.92	5.33E-01	7.88E-01

REGULATION OF PEPTIDE SECRETION	GO:0002791	66	0.10	0.92	5.33E-01	7.88E-01
REGULATION OF PROTEIN TARGETING TO MITOCHONDRION	GO:1903214	36	0.13	0.92	5.53E-01	7.86E-01
PURINE RIBONUCLEOTIDE BIOSYNTHETIC PROCESS	GO:0009152	90	0.08	0.92	5.57E-01	7.86E-01
TELOMERE ORGANIZATION	GO:0032200	66	0.09	0.90	5.85E-01	8.16E-01
POSITIVE REGULATION OF MITOCHONDRION ORGANIZATION	GO:0010822	59	0.10	0.89	5.68E-01	8.32E-01
POSTSYNAPSE ORGANIZATION	GO:0099173	33	0.13	0.89	6.02E-01	8.33E-01
REGULATION OF INTRINSIC APOPTOTIC SIGNALING PATHWAY	GO:2001242	110	0.07	0.89	5.83E-01	8.30E-01
REGULATION OF PEPTIDE TRANSPORT	GO:0090087	68	0.09	0.88	5.90E-01	8.36E-01
MEMBRANE ASSEMBLY	GO:0071709	34	0.13	0.88	6.25E-01	8.35E-01
CELLULAR LIPID CATABOLIC PROCESS	GO:0044242	120	0.07	0.88	5.85E-01	8.33E-01
REGULATION OF LIPID TRANSPORT	GO:0032368	56	0.10	0.88	5.62E-01	8.35E-01
MEMBRANE BIOGENESIS	GO:0044091	37	0.12	0.87	6.06E-01	8.43E-01
ESTABLISHMENT OF PROTEIN LOCALIZATION TO MEMBRANE	GO:0090150	160	0.06	0.87	6.23E-01	8.40E-01
NUCLEOSIDE METABOLIC PROCESS	GO:0009116	31	0.13	0.87	5.99E-01	8.39E-01
RESPONSE TO OXIDATIVE STRESS	GO:0006979	194	0.05	0.87	6.14E-01	8.35E-01
CELLULAR AMINO ACID CATABOLIC PROCESS	GO:0009063	53	0.10	0.87	6.30E-01	8.36E-01
ICOSANOID METABOLIC PROCESS	GO:0006690	59	0.10	0.86	6.13E-01	8.35E-01
PROTEIN TETRAMERIZATION	GO:0051262	49	0.10	0.86	6.48E-01	8.34E-01
CELLULAR MODIFIED AMINO ACID METABOLIC PROCESS	GO:0006575	84	0.08	0.86	6.34E-01	8.37E-01
REGULATION OF MITOCHONDRION ORGANIZATION	GO:0010821	107	0.07	0.86	6.49E-01	8.35E-01
PROTEIN-DNA COMPLEX SUBUNIT ORGANIZATION	GO:0071824	120	0.07	0.86	6.19E-01	8.34E-01
REGULATION OF ANTIGEN RECEPTOR-MEDIATED SIGNALING PATHWAY	GO:0050854	49	0.10	0.86	6.27E-01	8.33E-01
REGULATION OF PEPTIDE HORMONE SECRETION	GO:0090276	65	0.09	0.85	6.23E-01	8.31E-01
DNA PACKAGING	GO:0006323	90	0.08	0.85	6.71E-01	8.28E-01
CHOLESTEROL METABOLIC PROCESS	GO:0008203	50	0.10	0.85	6.54E-01	8.26E-01
NEUROTRANSMITTER SECRETION	GO:0007269	35	0.12	0.85	6.60E-01	8.33E-01
SIGNAL RELEASE FROM SYNAPSE	GO:0099643	35	0.12	0.84	6.52E-01	8.40E-01
RIBONUCLEOTIDE BIOSYNTHETIC PROCESS	GO:0009260	97	0.07	0.83	6.74E-01	8.51E-01
NUCLEOSIDE PHOSPHATE BIOSYNTHETIC PROCESS	GO:1901293	136	0.06	0.83	6.74E-01	8.60E-01
ALPHA-AMINO ACID CATABOLIC PROCESS	GO:1901606	35	0.12	0.82	6.96E-01	8.68E-01
RHO PROTEIN SIGNAL TRANSDUCTION	GO:0007266	41	0.11	0.82	6.79E-01	8.65E-01
POSITIVE REGULATION OF ACTIN FILAMENT BUNDLE ASSEMBLY	GO:0032233	38	0.11	0.82	7.06E-01	8.67E-01
NUCLEAR MEMBRANE ORGANIZATION	GO:0071763	31	0.13	0.81	6.91E-01	8.82E-01

MITOTIC METAPHASE PLATE CONGRESSION	GO:0007080	44	0.10	0.80	6.86E-01	8.78E-01
SECONDARY ALCOHOL METABOLIC PROCESS	GO:1902652	56	0.09	0.80	7.02E-01	8.86E-01
REGULATION OF BODY FLUID LEVELS	GO:0050878	143	0.06	0.80	7.38E-01	8.85E-01
POSITIVE REGULATION OF STRESS FIBER ASSEMBLY	GO:0051496	34	0.11	0.79	7.46E-01	8.96E-01
CANONICAL WNT SIGNALING PATHWAY	GO:0060070	34	0.12	0.79	7.34E-01	8.93E-01
RESPONSE TO TOXIC SUBSTANCE	GO:0009636	52	0.09	0.79	7.23E-01	8.92E-01
RIBOSE PHOSPHATE BIOSYNTHETIC PROCESS	GO:0046390	103	0.07	0.78	7.37E-01	8.91E-01
REGULATION OF NOTCH SIGNALING PATHWAY	GO:0008593	54	0.09	0.78	7.54E-01	8.89E-01
NUCLEAR ENVELOPE ORGANIZATION	GO:0006998	39	0.10	0.78	7.66E-01	8.94E-01
VITAMIN METABOLIC PROCESS	GO:0006766	54	0.09	0.77	7.66E-01	9.01E-01
GLYCOSYL COMPOUND METABOLIC PROCESS	GO:1901657	49	0.09	0.77	7.66E-01	9.02E-01
ENDOTHELIAL CELL MIGRATION	GO:0043542	34	0.11	0.77	7.44E-01	9.02E-01
PURINE-CONTAINING COMPOUND BIOSYNTHETIC PROCESS	GO:0072522	106	0.06	0.76	7.94E-01	9.11E-01
NEURAL TUBE DEVELOPMENT	GO:0021915	32	0.11	0.76	7.60E-01	9.10E-01
BIOLOGICAL PROCESS INVOLVED IN INTERACTION WITH SYMBIONT	GO:0051702	55	0.09	0.75	7.82E-01	9.11E-01
CELLULAR IRON ION HOMEOSTASIS	GO:0006879	39	0.10	0.75	7.68E-01	9.11E-01
NEUROTRANSMITTER TRANSPORT	GO:0006836	44	0.09	0.74	8.07E-01	9.24E-01
NEGATIVE REGULATION OF STRESS-ACTIVATED MAPK CASCADE	GO:0032873	32	0.11	0.74	7.76E-01	9.20E-01
CHROMATIN ASSEMBLY	GO:0031497	63	0.08	0.73	8.20E-01	9.26E-01
DETOXIFICATION	GO:0098754	32	0.11	0.73	8.12E-01	9.28E-01
REGULATION OF RELEASE OF CYTOCHROME C FROM MITOCHONDRIA	GO:0090199	37	0.10	0.73	8.37E-01	9.28E-01
PROTEIN LOCALIZATION TO CELL JUNCTION	GO:1902414	31	0.11	0.72	8.38E-01	9.28E-01
NUCLEOTIDE BIOSYNTHETIC PROCESS	GO:0009165	133	0.05	0.72	8.12E-01	9.25E-01
NEGATIVE REGULATION OF STRESS-ACTIVATED PROTEIN KINASE SIGNALING CASCADE	GO:0070303	32	0.11	0.72	7.88E-01	9.22E-01
LIPID CATABOLIC PROCESS	GO:0016042	132	0.05	0.72	8.37E-01	9.23E-01
RNA PHOSPHODIESTER BOND HYDROLYSIS	GO:0090501	61	0.08	0.71	8.00E-01	9.27E-01
ORGANELLE DISASSEMBLY	GO:1903008	67	0.07	0.70	8.56E-01	9.44E-01
REGULATION OF CILIUM ASSEMBLY	GO:1902017	61	0.07	0.69	8.34E-01	9.46E-01
POSITIVE REGULATION OF MONONUCLEAR CELL PROLIFERATION	GO:0032946	78	0.07	0.69	8.70E-01	9.47E-01
POSITIVE REGULATION OF LYMPHOCYTE PROLIFERATION	GO:0050671	77	0.07	0.68	8.76E-01	9.52E-01
GLYCOLIPID BIOSYNTHETIC PROCESS	GO:0009247	45	0.09	0.67	8.56E-01	9.59E-01
POSITIVE REGULATION OF IMMUNOGLOBULIN PRODUCTION	GO:0002639	31	0.10	0.67	8.75E-01	9.59E-01
ACTION POTENTIAL	GO:0001508	31	0.10	0.67	8.84E-01	9.56E-01

METAPHASE PLATE CONGRESSION	GO:0051310	48	0.08	0.66	9.09E-01	9.66E-01
RESPONSE TO RETINOIC ACID	GO:0032526	32	0.10	0.65	8.89E-01	9.71E-01
ERYTHROCYTE HOMEOSTASIS	GO:0034101	45	0.08	0.63	9.00E-01	9.87E-01
NCRNA CATABOLIC PROCESS	GO:0034661	30	0.10	0.63	9.12E-01	9.83E-01
HEART PROCESS	GO:0003015	33	0.09	0.62	9.40E-01	9.87E-01
POSITIVE REGULATION OF PROTEIN DEPHOSPHORYLATION	GO:0035307	31	0.10	0.61	9.06E-01	9.94E-01
POSITIVE REGULATION OF HORMONE SECRETION	GO:0046887	36	0.09	0.61	9.48E-01	9.93E-01
COAGULATION	GO:0050817	81	0.05	0.58	9.75E-01	1.00E+00
UNSATURATED FATTY ACID METABOLIC PROCESS	GO:0033559	59	0.06	0.58	9.64E-01	1.00E+00
BLOOD COAGULATION	GO:0007596	81	0.05	0.57	9.57E-01	1.00E+00
LIPID HOMEOSTASIS	GO:0055088	74	0.06	0.57	9.73E-01	1.00E+00
DNA DAMAGE RESPONSE, SIGNAL TRANSDUCTION BY P53 CLASS MEDIATOR	GO:0030330	33	0.08	0.56	9.67E-01	1.00E+00
FATTY ACID METABOLIC PROCESS	GO:0006631	180	0.03	0.54	9.71E-01	1.00E+00
PROTEIN N-LINKED GLYCOSYLATION	GO:0006487	54	0.06	0.54	9.76E-01	1.00E+00
HEMOSTASIS	GO:0007599	85	0.05	0.53	9.82E-01	1.00E+00
POSITIVE REGULATION OF HEMOPOIESIS	GO:1903708	87	0.05	0.53	9.78E-01	1.00E+00
APPENDAGE DEVELOPMENT	GO:0048736	33	0.08	0.53	9.84E-01	1.00E+00
LIMB DEVELOPMENT	GO:0060173	33	0.08	0.52	9.84E-01	9.97E-01
POSITIVE REGULATION OF LEUKOCYTE DIFFERENTIATION	GO:1902107	87	0.05	0.52	9.83E-01	9.94E-01
HISTONE H3 ACETYLATION	GO:0043966	40	0.06	0.48	9.92E-01	1.00E+00
ENSHEATHMENT OF NEURONS	GO:0007272	39	0.05	0.42	1.00E+00	1.00E+00
AXON ENSHEATHMENT	GO:0008366	39	0.05	0.40	1.00E+00	1.00E+00
REGULATION OF G PROTEIN-COUPLED RECEPTOR SIGNALING PATHWAY	GO:0008277	62	-0.04	-0.41	1.00E+00	9.99E-01
POSITIVE REGULATION OF T CELL PROLIFERATION	GO:0042102	57	-0.05	-0.43	1.00E+00	9.99E-01
MYELINATION	GO:0042552	38	-0.06	-0.47	9.98E-01	9.96E-01
REGULATION OF OXIDOREDUCTASE ACTIVITY	GO:0051341	48	-0.06	-0.48	9.96E-01	9.96E-01
REGULATION OF GLUCOSE METABOLIC PROCESS	GO:0010906	54	-0.06	-0.50	9.87E-01	9.93E-01
ACTIN FILAMENT BUNDLE ASSEMBLY	GO:0051017	34	-0.07	-0.50	9.94E-01	9.94E-01
DNA BIOSYNTHETIC PROCESS	GO:0071897	48	-0.06	-0.53	9.82E-01	9.86E-01
POSITIVE REGULATION OF ALPHA-BETA T CELL ACTIVATION	GO:0046635	39	-0.07	-0.53	9.84E-01	9.86E-01
REGULATION OF IMMUNOGLOBULIN PRODUCTION	GO:0002637	39	-0.07	-0.53	9.80E-01	9.87E-01
NUCLEOSOME ASSEMBLY	GO:0006334	45	-0.07	-0.53	9.77E-01	9.88E-01
POSITIVE REGULATION OF INTRACELLULAR TRANSPORT	GO:0032388	149	-0.04	-0.54	9.84E-01	9.86E-01

IRON ION HOMEOSTASIS	GO:0055072	46	-0.07	-0.55	9.73E-01	9.82E-01
NEGATIVE REGULATION OF DNA METABOLIC PROCESS	GO:0051053	81	-0.05	-0.55	9.69E-01	9.80E-01
REGULATION OF SYNAPTIC PLASTICITY	GO:0048167	67	-0.06	-0.56	9.65E-01	9.77E-01
T CELL DIFFERENTIATION	GO:0030217	62	-0.06	-0.56	9.78E-01	9.77E-01
REGULATION OF B CELL PROLIFERATION	GO:0030888	39	-0.08	-0.56	9.71E-01	9.77E-01
POSITIVE REGULATION OF LYMPHOCYTE DIFFERENTIATION	GO:0045621	59	-0.06	-0.57	9.51E-01	9.76E-01
MULTICELLULAR ORGANISMAL SIGNALING	GO:0035637	36	-0.08	-0.57	9.71E-01	9.77E-01
POSITIVE REGULATION OF SYNAPTIC TRANSMISSION	GO:0050806	39	-0.08	-0.57	9.63E-01	9.76E-01
NEGATIVE REGULATION OF RESPONSE TO DNA DAMAGE STIMULUS	GO:2001021	62	-0.06	-0.57	9.57E-01	9.76E-01
NEURAL NUCLEUS DEVELOPMENT	GO:0048857	35	-0.08	-0.57	9.64E-01	9.75E-01
REGULATION OF ALTERNATIVE MRNA SPLICING, VIA SPLICEOSOME	GO:0000381	39	-0.08	-0.58	9.68E-01	9.74E-01
SUBSTANTIA NIGRA DEVELOPMENT	GO:0021762	35	-0.08	-0.59	9.50E-01	9.68E-01
ARACHIDONIC ACID METABOLIC PROCESS	GO:0019369	33	-0.09	-0.59	9.38E-01	9.65E-01
ACTIN FILAMENT BUNDLE ORGANIZATION	GO:0061572	36	-0.08	-0.60	9.50E-01	9.61E-01
POSITIVE REGULATION OF LEUKOCYTE PROLIFERATION	GO:0070665	85	-0.06	-0.60	9.35E-01	9.61E-01
AMEBOIDAL-TYPE CELL MIGRATION	GO:0001667	68	-0.06	-0.61	9.43E-01	9.56E-01
EXECUTION PHASE OF APOPTOSIS	GO:0097194	30	-0.09	-0.62	9.49E-01	9.49E-01
REGULATION OF PROTEIN DEPHOSPHORYLATION	GO:0035304	64	-0.07	-0.62	9.30E-01	9.47E-01
POSITIVE REGULATION OF JNK CASCADE	GO:0046330	59	-0.07	-0.62	9.42E-01	9.47E-01
POSITIVE REGULATION OF LEUKOCYTE CELL-CELL ADHESION	GO:1903039	152	-0.04	-0.62	9.11E-01	9.48E-01
ACYLGLYCEROL METABOLIC PROCESS	GO:0006639	44	-0.08	-0.62	9.27E-01	9.45E-01
PURINE-CONTAINING COMPOUND CATABOLIC PROCESS	GO:0072523	31	-0.10	-0.62	9.12E-01	9.45E-01
NEUTRAL LIPID METABOLIC PROCESS	GO:0006638	44	-0.08	-0.63	9.23E-01	9.45E-01
NEGATIVE REGULATION OF MRNA METABOLIC PROCESS	GO:1903312	57	-0.07	-0.63	9.16E-01	9.42E-01
CARTILAGE DEVELOPMENT	GO:0051216	31	-0.10	-0.64	9.18E-01	9.37E-01
PRIMARY ALCOHOL METABOLIC PROCESS	GO:0034308	30	-0.10	-0.64	9.15E-01	9.35E-01
NEGATIVE REGULATION OF NEURON DEATH	GO:1901215	63	-0.07	-0.64	8.98E-01	9.31E-01
POSITIVE REGULATION OF CELL MORPHOGENESIS INVOLVED IN DIFFERENTIATION	GO:0010770	41	-0.09	-0.65	9.00E-01	9.30E-01
REGULATION OF DNA BINDING	GO:0051101	64	-0.07	-0.65	9.00E-01	9.31E-01
REGULATION OF NUCLEAR DIVISION	GO:0051783	73	-0.07	-0.65	9.01E-01	9.31E-01
REGULATION OF MITOTIC NUCLEAR DIVISION	GO:0007088	70	-0.07	-0.65	9.01E-01	9.26E-01
NEGATIVE REGULATION OF WNT SIGNALING PATHWAY	GO:0030178	85	-0.06	-0.66	9.04E-01	9.24E-01
PHOSPHATIDYLCHOLINE METABOLIC PROCESS	GO:0046470	37	-0.09	-0.66	9.02E-01	9.23E-01

NEGATIVE REGULATION OF CELL GROWTH	GO:0030308	97	-0.06	-0.66	8.87E-01	9.17E-01
PROTEIN-DNA COMPLEX ASSEMBLY	GO:0065004	81	-0.06	-0.66	8.94E-01	9.18E-01
POSITIVE REGULATION OF INTRACELLULAR PROTEIN TRANSPORT	GO:0090316	118	-0.05	-0.66	8.96E-01	9.16E-01
PROTEIN-CONTAINING COMPLEX DISASSEMBLY	GO:0032984	100	-0.06	-0.67	8.82E-01	9.16E-01
REGULATION OF CELLULAR CARBOHYDRATE METABOLIC PROCESS	GO:0010675	70	-0.07	-0.67	8.54E-01	9.08E-01
NEGATIVE REGULATION OF INTRINSIC APOPTOTIC SIGNALING PATHWAY	GO:2001243	68	-0.07	-0.68	8.62E-01	9.02E-01
PROTEIN STABILIZATION	GO:0050821	152	-0.05	-0.68	8.77E-01	9.01E-01
CELLULAR GLUCOSE HOMEOSTASIS	GO:0001678	38	-0.09	-0.68	8.75E-01	9.01E-01
POSITIVE REGULATION OF DNA BIOSYNTHETIC PROCESS	GO:2000573	52	-0.08	-0.68	8.53E-01	9.01E-01
REGULATION OF GRANULOCYTE CHEMOTAXIS	GO:0071622	30	-0.10	-0.68	8.54E-01	9.01E-01
PEROXISOME ORGANIZATION	GO:0007031	30	-0.11	-0.69	8.48E-01	8.97E-01
POSITIVE REGULATION OF T CELL ACTIVATION	GO:0050870	136	-0.05	-0.70	8.52E-01	8.86E-01
POSITIVE REGULATION OF T CELL DIFFERENTIATION	GO:0045582	50	-0.08	-0.70	8.55E-01	8.77E-01
MODULATION OF CHEMICAL SYNAPTIC TRANSMISSION	GO:0050804	123	-0.05	-0.70	8.44E-01	8.77E-01
RIBOSE PHOSPHATE METABOLIC PROCESS	GO:0019693	196	-0.04	-0.71	8.08E-01	8.73E-01
REGULATION OF TRANS-SYNAPTIC SIGNALING	GO:0099177	124	-0.05	-0.71	8.49E-01	8.69E-01
REGULATION OF DNA BIOSYNTHETIC PROCESS	GO:2000278	84	-0.07	-0.72	8.12E-01	8.59E-01
LONG-CHAIN FATTY ACID METABOLIC PROCESS	GO:0001676	67	-0.07	-0.72	8.28E-01	8.58E-01
REGULATION OF CELL ADHESION MEDIATED BY INTEGRIN	GO:0033628	30	-0.11	-0.73	8.33E-01	8.52E-01
SEX DIFFERENTIATION	GO:0007548	37	-0.10	-0.73	7.98E-01	8.51E-01
NEGATIVE REGULATION OF TELOMERE MAINTENANCE	GO:0032205	31	-0.11	-0.73	8.05E-01	8.47E-01
REGULATION OF HISTONE ACETYLATION	GO:0035065	40	-0.10	-0.73	8.02E-01	8.48E-01
MICROTUBULE BUNDLE FORMATION	GO:0001578	40	-0.10	-0.73	7.81E-01	8.47E-01
POSITIVE REGULATION OF ACTIN FILAMENT POLYMERIZATION	GO:0030838	57	-0.08	-0.74	7.86E-01	8.35E-01
ALPHA-BETA T CELL ACTIVATION	GO:0046631	30	-0.11	-0.74	7.70E-01	8.33E-01
GLYCOLIPID METABOLIC PROCESS	GO:0006664	65	-0.08	-0.74	7.67E-01	8.32E-01
TISSUE MIGRATION	GO:0090130	45	-0.09	-0.75	7.72E-01	8.30E-01
FATTY ACID BIOSYNTHETIC PROCESS	GO:0006633	61	-0.08	-0.75	7.74E-01	8.30E-01
REGULATION OF COAGULATION	GO:0050818	31	-0.12	-0.75	7.51E-01	8.30E-01
CHEMICAL SYNAPTIC TRANSMISSION	GO:0007268	114	-0.06	-0.75	7.79E-01	8.30E-01
CONNECTIVE TISSUE DEVELOPMENT	GO:0061448	51	-0.09	-0.75	7.91E-01	8.28E-01
SIGNAL RELEASE	GO:0023061	57	-0.08	-0.75	7.69E-01	8.23E-01
CHROMOSOME LOCALIZATION	GO:0050000	52	-0.09	-0.76	8.00E-01	8.22E-01

REGULATION OF HORMONE SECRETION	GO:0046883	81	-0.07	-0.76	7.85E-01	8.21E-01
DNA DUPLEX UNWINDING	GO:0032508	30	-0.11	-0.76	7.75E-01	8.21E-01
EPITHELIAL CELL MIGRATION	GO:0010631	44	-0.10	-0.76	7.86E-01	8.21E-01
MEIOSIS I CELL CYCLE PROCESS	GO:0061982	46	-0.09	-0.76	7.60E-01	8.19E-01
ANTEROGRADE TRANS-SYNAPTIC SIGNALING	GO:0098916	114	-0.06	-0.76	7.57E-01	8.18E-01
SYNAPTIC SIGNALING	GO:0099536	130	-0.06	-0.76	7.72E-01	8.16E-01
PROTEIN LOCALIZATION TO ENDOPLASMIC RETICULUM	GO:0070972	48	-0.09	-0.77	7.54E-01	8.12E-01
ERYTHROCYTE DIFFERENTIATION	GO:0030218	42	-0.10	-0.77	7.84E-01	8.12E-01
EPITHELIUM MIGRATION	GO:0090132	44	-0.10	-0.77	7.57E-01	8.11E-01
CELLULAR PROTEIN COMPLEX DISASSEMBLY	GO:0043624	37	-0.11	-0.77	7.64E-01	8.08E-01
CALCIUM ION TRANSPORT	GO:0006816	94	-0.07	-0.77	7.48E-01	8.08E-01
GLIAL CELL DIFFERENTIATION	GO:0010001	52	-0.09	-0.77	7.39E-01	8.05E-01
G2/M TRANSITION OF MITOTIC CELL CYCLE	GO:0000086	36	-0.11	-0.77	7.55E-01	8.05E-01
MEIOSIS I	GO:0007127	44	-0.10	-0.77	7.50E-01	8.05E-01
REGULATION OF TELOMERASE ACTIVITY	GO:0051972	43	-0.10	-0.78	7.41E-01	8.03E-01
WATER-SOLUBLE VITAMIN METABOLIC PROCESS	GO:0006767	44	-0.10	-0.78	7.24E-01	7.95E-01
MYELOID CELL HOMEOSTASIS	GO:0002262	52	-0.09	-0.78	7.55E-01	7.92E-01
DNA CONFORMATION CHANGE	GO:0071103	133	-0.06	-0.78	7.29E-01	7.92E-01
CELL CYCLE G2/M PHASE TRANSITION	GO:0044839	38	-0.11	-0.79	7.32E-01	7.86E-01
LIPOSACCHARIDE METABOLIC PROCESS	GO:1903509	66	-0.08	-0.79	7.50E-01	7.81E-01
REGULATION OF PEPTIDYL-THREONINE PHOSPHORYLATION	GO:0010799	31	-0.12	-0.79	7.28E-01	7.80E-01
NEGATIVE REGULATION OF CHEMOTAXIS	GO:0050922	30	-0.12	-0.80	7.32E-01	7.77E-01
SYNAPSE ORGANIZATION	GO:0050808	89	-0.07	-0.80	7.25E-01	7.77E-01
REGULATION OF DNA-DEPENDENT DNA REPLICATION	GO:0090329	36	-0.11	-0.80	7.03E-01	7.70E-01
ANTEROGRADE AXONAL TRANSPORT	GO:0008089	30	-0.12	-0.80	7.10E-01	7.65E-01
REGULATION OF HEMOSTASIS	GO:1900046	30	-0.12	-0.81	7.24E-01	7.61E-01
LYSOSOME LOCALIZATION	GO:0032418	34	-0.11	-0.81	6.91E-01	7.61E-01
ANTIGEN PROCESSING AND PRESENTATION OF EXOGENOUS PEPTIDE ANTIGEN	GO:0002478	31	-0.12	-0.81	7.04E-01	7.61E-01
ANTIGEN PROCESSING AND PRESENTATION OF PEPTIDE ANTIGEN	GO:0048002	51	-0.09	-0.81	7.13E-01	7.61E-01
NEGATIVE REGULATION OF CANONICAL WNT SIGNALING PATHWAY	GO:0090090	69	-0.08	-0.81	6.97E-01	7.60E-01
REGULATION OF LIPID LOCALIZATION	GO:1905952	77	-0.08	-0.81	6.71E-01	7.57E-01
REGULATION OF HEART RATE	GO:0002027	33	-0.12	-0.81	7.36E-01	7.57E-01
POSITIVE REGULATION OF PROTEIN TRANSPORT	GO:0051222	181	-0.05	-0.81	6.95E-01	7.57E-01

REGULATION OF BLOOD COAGULATION	GO:0030193	30	-0.12	-0.81	7.11E-01	7.57E-01
CHOLESTEROL HOMEOSTASIS	GO:0042632	40	-0.11	-0.82	6.65E-01	7.53E-01
REGULATION OF REPRODUCTIVE PROCESS	GO:2000241	36	-0.11	-0.82	6.92E-01	7.52E-01
LEUKOCYTE CELL-CELL ADHESION	GO:0007159	39	-0.11	-0.82	7.02E-01	7.52E-01
POSITIVE REGULATION OF ESTABLISHMENT OF PROTEIN LOCALIZATION	GO:1904951	197	-0.05	-0.82	7.08E-01	7.52E-01
PURINE NUCLEOTIDE METABOLIC PROCESS	GO:0006163	193	-0.05	-0.82	6.88E-01	7.51E-01
NUCLEOTIDE-EXCISION REPAIR	GO:0006289	48	-0.10	-0.82	6.78E-01	7.49E-01
PROCESS	GO:0031146	37	-0.11	-0.82	6.87E-01	7.49E-01
REGULATION OF LEUKOCYTE APOPTOTIC PROCESS	GO:2000106	45	-0.10	-0.82	6.71E-01	7.45E-01
REGULATION OF ACTIN FILAMENT BUNDLE ASSEMBLY	GO:0032231	61	-0.09	-0.83	6.80E-01	7.33E-01
POSITIVE REGULATION OF CELL-CELL ADHESION	GO:0022409	172	-0.05	-0.83	6.48E-01	7.31E-01
CELL-CELL JUNCTION ORGANIZATION	GO:0045216	72	-0.08	-0.84	6.65E-01	7.28E-01
MODULATION BY HOST OF SYMBIONT PROCESS	GO:0051851	45	-0.11	-0.84	6.46E-01	7.22E-01
TRANS-SYNAPTIC SIGNALING	GO:0099537	117	-0.07	-0.84	6.87E-01	7.20E-01
NEGATIVE REGULATION OF RESPONSE TO WOUNDING	GO:1903035	39	-0.11	-0.84	6.42E-01	7.18E-01
MUSCLE CELL DIFFERENTIATION	GO:0042692	68	-0.09	-0.85	6.35E-01	7.14E-01
RESPONSE TO LIGHT STIMULUS	GO:0009416	156	-0.06	-0.85	6.60E-01	7.12E-01
TUBE FORMATION	GO:0035148	39	-0.12	-0.85	6.78E-01	7.10E-01
REGULATION OF TORC1 SIGNALING	GO:1903432	33	-0.12	-0.85	6.21E-01	7.06E-01
REGULATION OF STRESS FIBER ASSEMBLY	GO:0051492	57	-0.09	-0.85	6.27E-01	7.05E-01
REGULATION OF RHO PROTEIN SIGNAL TRANSDUCTION	GO:0035023	52	-0.10	-0.85	6.43E-01	7.04E-01
POSITIVE REGULATION OF PROTEIN POLYMERIZATION	GO:0032273	84	-0.08	-0.85	6.50E-01	7.03E-01
REGULATION OF INTRACELLULAR PROTEIN TRANSPORT	GO:0033157	156	-0.06	-0.86	6.40E-01	6.99E-01
REGULATION OF MUSCLE CELL DIFFERENTIATION	GO:0051147	47	-0.11	-0.86	6.24E-01	7.00E-01
REGULATION OF MUSCLE CONTRACTION	GO:0006937	63	-0.09	-0.86	6.31E-01	6.98E-01
DNA REPLICATION	GO:0006260	126	-0.07	-0.86	6.22E-01	6.98E-01
POSITIVE REGULATION OF CALCIUM ION TRANSPORT	GO:0051928	47	-0.10	-0.86	6.44E-01	6.98E-01
NEGATIVE REGULATION OF CHROMOSOME ORGANIZATION	GO:2001251	56	-0.10	-0.86	6.19E-01	6.92E-01
POSITIVE REGULATION OF DEPHOSPHORYLATION	GO:0035306	37	-0.12	-0.87	6.28E-01	6.90E-01
RNA 3'-END PROCESSING	GO:0031123	77	-0.08	-0.87	6.25E-01	6.90E-01
RIBONUCLEOTIDE METABOLIC PROCESS	GO:0009259	188	-0.05	-0.87	6.05E-01	6.88E-01
CELLULAR PIGMENTATION	GO:0033059	34	-0.13	-0.87	5.70E-01	6.83E-01
ACTIN FILAMENT ORGANIZATION	GO:0007015	163	-0.06	-0.87	5.98E-01	6.80E-01

POSITIVE REGULATION OF PROTEIN SECRETION	GO:0050714	57	-0.10	-0.87	6.27E-01	6.80E-01
PROTEIN IMPORT	GO:0017038	105	-0.07	-0.87	5.96E-01	6.80E-01
REPLICATION FORK PROCESSING	GO:0031297	32	-0.13	-0.88	6.30E-01	6.78E-01
STEROL HOMEOSTASIS	GO:0055092	41	-0.11	-0.88	6.23E-01	6.78E-01
CARBOXYLIC ACID BIOSYNTHETIC PROCESS	GO:0046394	133	-0.07	-0.88	5.96E-01	6.72E-01
PURINE NUCLEOTIDE BIOSYNTHETIC PROCESS	GO:0006164	99	-0.08	-0.88	5.93E-01	6.73E-01
ORGANIC ACID BIOSYNTHETIC PROCESS	GO:0016053	133	-0.07	-0.88	5.93E-01	6.68E-01
ALPHA-AMINO ACID BIOSYNTHETIC PROCESS	GO:1901607	36	-0.12	-0.88	5.98E-01	6.68E-01
REGULATION OF ANIMAL ORGAN MORPHOGENESIS	GO:2000027	40	-0.12	-0.88	5.95E-01	6.68E-01
CELLULAR RESPONSE TO ACID CHEMICAL	GO:0071229	33	-0.13	-0.89	6.14E-01	6.66E-01
ESTABLISHMENT OF CHROMOSOME LOCALIZATION	GO:0051303	50	-0.10	-0.89	5.95E-01	6.67E-01
REGULATION OF INTERLEUKIN-2 PRODUCTION	GO:0032663	44	-0.11	-0.89	5.66E-01	6.61E-01
POSITIVE REGULATION OF CELLULAR PROTEIN LOCALIZATION	GO:1903829	191	-0.06	-0.89	5.87E-01	6.61E-01
PROTEIN PROCESSING	GO:0016485	101	-0.08	-0.90	5.75E-01	6.52E-01
REGULATED EXOCYTOSIS	GO:0045055	60	-0.10	-0.90	5.86E-01	6.49E-01
REGULATION OF LAMELLIPODIUM ORGANIZATION	GO:1902743	38	-0.12	-0.90	5.78E-01	6.48E-01
SUBSTRATE ADHESION-DEPENDENT CELL SPREADING	GO:0034446	30	-0.14	-0.90	5.71E-01	6.49E-01
ANTIGEN PROCESSING AND PRESENTATION OF EXOGENOUS ANTIGEN	GO:0019884	36	-0.13	-0.90	5.57E-01	6.48E-01
RETROGRADE VESICLE-MEDIATED TRANSPORT, GOLGI TO ENDOPLASMIC RETICULUM	GO:0006890	43	-0.12	-0.90	5.70E-01	6.48E-01
REGULATION OF MICROTUBULE POLYMERIZATION	GO:0031113	38	-0.12	-0.90	5.71E-01	6.44E-01
REGULATION OF CARBOHYDRATE BIOSYNTHETIC PROCESS	GO:0043255	48	-0.11	-0.90	5.60E-01	6.44E-01
CELLULAR TRANSITION METAL ION HOMEOSTASIS	GO:0046916	66	-0.09	-0.90	5.78E-01	6.43E-01
REGULATION OF TELOMERE MAINTENANCE VIA TELOMERASE	GO:0032210	48	-0.11	-0.90	5.54E-01	6.44E-01
POSITIVE REGULATION OF CYTOSKELETON ORGANIZATION	GO:0051495	135	-0.07	-0.90	5.65E-01	6.44E-01
REGULATION OF EPITHELIAL CELL APOPTOTIC PROCESS	GO:1904035	38	-0.12	-0.91	5.62E-01	6.43E-01
PURINE RIBONUCLEOTIDE METABOLIC PROCESS	GO:0009150	180	-0.06	-0.91	5.83E-01	6.43E-01
NEGATIVE REGULATION OF I-KAPPAB KINASE/NF-KAPPAB SIGNALING	GO:0043124	35	-0.13	-0.91	5.33E-01	6.42E-01
REGULATION OF MITOTIC SISTER CHROMATID SEGREGATION	GO:0033047	36	-0.13	-0.91	5.88E-01	6.40E-01
REGULATION OF REACTIVE OXYGEN SPECIES METABOLIC PROCESS	GO:2000377	79	-0.09	-0.91	5.40E-01	6.40E-01
CHROMATIN REMODELING	GO:0006338	136	-0.07	-0.91	5.43E-01	6.38E-01
POSITIVE REGULATION OF SUPRAMOLECULAR FIBER ORGANIZATION	GO:1902905	124	-0.07	-0.91	5.30E-01	6.36E-01
ACTIVATION OF CYSTEINE-TYPE ENDOPEPTIDASE ACTIVITY INVOLVED IN APOPTOTIC PROCESS	GO:0006919	59	-0.10	-0.91	5.48E-01	6.34E-01

CARBOHYDRATE DERIVATIVE TRANSPORT	GO:1901264	46	-0.12	-0.91	5.52E-01	6.34E-01
GLYCOSPHINGOLIPID METABOLIC PROCESS	GO:0006687	32	-0.14	-0.92	5.59E-01	6.32E-01
POSITIVE REGULATION OF LIPID TRANSPORT	GO:0032370	35	-0.13	-0.92	5.88E-01	6.30E-01
REGULATION OF MYELOID LEUKOCYTE DIFFERENTIATION	GO:0002761	57	-0.10	-0.92	5.68E-01	6.29E-01
REGULATION OF MUSCLE SYSTEM PROCESS	GO:0090257	85	-0.09	-0.92	5.31E-01	6.27E-01
POSITIVE REGULATION OF CELL CYCLE G1/S PHASE TRANSITION	GO:1902808	32	-0.14	-0.92	5.46E-01	6.24E-01
NEGATIVE REGULATION OF G2/M TRANSITION OF MITOTIC CELL CYCLE	GO:0010972	46	-0.12	-0.92	5.38E-01	6.24E-01
CELLULAR RESPONSE TO UV	GO:0034644	70	-0.09	-0.92	5.36E-01	6.24E-01
RESPONSE TO KETONE	GO:1901654	39	-0.13	-0.92	5.44E-01	6.25E-01
MONOCARBOXYLIC ACID TRANSPORT	GO:0015718	30	-0.14	-0.93	5.24E-01	6.21E-01
REGULATION OF LEUKOCYTE DIFFERENTIATION	GO:1902105	150	-0.06	-0.93	5.10E-01	6.16E-01
REGULATION OF CALCIUM-MEDIATED SIGNALING	GO:0050848	37	-0.13	-0.93	5.46E-01	6.15E-01
REGULATION OF ACTIN FILAMENT POLYMERIZATION	GO:0030833	107	-0.08	-0.93	5.44E-01	6.12E-01
HORMONE METABOLIC PROCESS	GO:0042445	49	-0.11	-0.93	5.08E-01	6.09E-01
SULFUR COMPOUND METABOLIC PROCESS	GO:0006790	198	-0.06	-0.93	5.04E-01	6.08E-01
REGULATION OF HORMONE LEVELS	GO:0010817	161	-0.06	-0.94	5.31E-01	6.03E-01
RESPONSE TO REACTIVE OXYGEN SPECIES	GO:0000302	86	-0.09	-0.94	5.10E-01	5.98E-01
POSITIVE REGULATION OF PROTEIN KINASE B SIGNALING	GO:0051897	62	-0.10	-0.94	5.11E-01	5.97E-01
NEGATIVE REGULATION OF TUMOR NECROSIS FACTOR SUPERFAMILY CYTOKINE PRODUCTION	GO:1903556	35	-0.14	-0.94	5.17E-01	5.95E-01
INTRINSIC APOPTOTIC SIGNALING PATHWAY IN RESPONSE TO DNA DAMAGE	GO:0008630	47	-0.12	-0.95	4.93E-01	5.94E-01
POSITIVE REGULATION OF PROTEIN LOCALIZATION TO MEMBRANE	GO:1905477	67	-0.10	-0.95	5.07E-01	5.94E-01
POSITIVE REGULATION OF CELL GROWTH	GO:0030307	74	-0.09	-0.95	5.19E-01	5.91E-01
'DE NOVO' POSTTRANSLATIONAL PROTEIN FOLDING	GO:0051084	31	-0.14	-0.95	4.76E-01	5.91E-01
REGULATION OF TELOMERE MAINTENANCE VIA TELOMERE LENGTHENING	GO:1904356	54	-0.11	-0.95	5.08E-01	5.91E-01
REGULATION OF CELL CYCLE G2/M PHASE TRANSITION	GO:1902749	67	-0.10	-0.95	5.21E-01	5.88E-01
DETECTION OF STIMULUS	GO:0051606	70	-0.10	-0.95	5.34E-01	5.87E-01
NEGATIVE REGULATION OF PROTEIN TRANSPORT	GO:0051224	59	-0.10	-0.95	4.80E-01	5.86E-01
NEGATIVE REGULATION OF DEPHOSPHORYLATION	GO:0035305	31	-0.15	-0.95	4.94E-01	5.84E-01
NEGATIVE REGULATION OF DEVELOPMENTAL GROWTH	GO:0048640	33	-0.14	-0.95	4.90E-01	5.85E-01
LIPOPROTEIN BIOSYNTHETIC PROCESS	GO:0042158	70	-0.10	-0.96	4.89E-01	5.80E-01
NEGATIVE REGULATION OF CELL CYCLE G2/M PHASE TRANSITION	GO:1902750	48	-0.12	-0.96	5.01E-01	5.78E-01
REGULATION OF ACTOMYOSIN STRUCTURE ORGANIZATION	GO:0110020	59	-0.11	-0.96	5.00E-01	5.76E-01

NEGATIVE REGULATION OF APOPTOTIC SIGNALING PATHWAY	GO:2001234	126	-0.07	-0.96	4.80E-01	5.75E-01
REGULATION OF INTERLEUKIN-12 PRODUCTION	GO:0032655	44	-0.12	-0.96	4.62E-01	5.74E-01
REGULATION OF MITOTIC SPINDLE ORGANIZATION	GO:0060236	31	-0.15	-0.97	4.88E-01	5.67E-01
IMMUNE RESPONSE-ACTIVATING SIGNAL TRANSDUCTION	GO:0002757	189	-0.06	-0.97	4.75E-01	5.66E-01
NEGATIVE REGULATION OF ESTABLISHMENT OF PROTEIN LOCALIZATION	GO:1904950	63	-0.10	-0.97	4.76E-01	5.63E-01
PROTEIN LIPIDATION	GO:0006497	70	-0.10	-0.97	4.68E-01	5.61E-01
POSITIVE REGULATION OF ENDOTHELIAL CELL PROLIFERATION	GO:0001938	44	-0.13	-0.97	4.94E-01	5.61E-01
RESPONSE TO AMINO ACID	GO:0043200	31	-0.14	-0.97	4.66E-01	5.61E-01
ACTIN FILAMENT-BASED MOVEMENT	GO:0030048	42	-0.12	-0.97	4.53E-01	5.61E-01
CORTICAL CYTOSKELETON ORGANIZATION	GO:0030865	38	-0.14	-0.97	4.87E-01	5.60E-01
MITOCHONDRION DISASSEMBLY	GO:0061726	38	-0.13	-0.97	4.74E-01	5.60E-01
REGULATION OF NIK/NF-KAPPAB SIGNALING	GO:1901222	67	-0.10	-0.98	4.57E-01	5.58E-01
AUTOPHAGY OF MITOCHONDRION	GO:0000422	38	-0.13	-0.98	4.59E-01	5.55E-01
STEM CELL DIFFERENTIATION	GO:0048863	56	-0.11	-0.98	4.49E-01	5.53E-01
MONOCARBOXYLIC ACID BIOSYNTHETIC PROCESS	GO:0072330	80	-0.09	-0.99	4.54E-01	5.44E-01
REGULATION OF TELOMERE MAINTENANCE	GO:0032204	72	-0.10	-0.99	4.58E-01	5.42E-01
POSITIVE REGULATION OF ENDOTHELIAL CELL MIGRATION	GO:0010595	72	-0.10	-0.99	4.55E-01	5.42E-01
OSSIFICATION	GO:0001503	87	-0.09	-0.99	4.29E-01	5.41E-01
PROTEIN LOCALIZATION TO PLASMA MEMBRANE	GO:0072659	128	-0.07	-0.99	4.39E-01	5.41E-01
REGULATION OF LIPID STORAGE	GO:0010883	36	-0.14	-0.99	4.61E-01	5.41E-01
REGULATION OF SUBSTRATE ADHESION-DEPENDENT CELL SPREADING	GO:1900024	39	-0.13	-0.99	4.56E-01	5.41E-01
POSITIVE REGULATION OF STRESS-ACTIVATED MAPK CASCADE	GO:0032874	78	-0.10	-0.99	4.56E-01	5.41E-01
MUSCLE SYSTEM PROCESS	GO:0003012	90	-0.09	-0.99	4.45E-01	5.35E-01
MIDBRAIN DEVELOPMENT	GO:0030901	45	-0.13	-0.99	4.46E-01	5.36E-01
NEGATIVE REGULATION OF WOUND HEALING	GO:0061045	30	-0.15	-0.99	4.20E-01	5.36E-01
NEGATIVE REGULATION OF ENDOCYTOSIS	GO:0045806	34	-0.14	-0.99	4.34E-01	5.35E-01
REGULATION OF CARBOHYDRATE METABOLIC PROCESS	GO:0006109	93	-0.09	-1.00	4.47E-01	5.32E-01
MALE GAMETE GENERATION	GO:0048232	125	-0.08	-1.00	4.29E-01	5.32E-01
GO ID NOT FOUND IN OBO FILE ONTOLOGY DEFINITIONS	GO:0042493	37	-0.14	-1.00	4.31E-01	5.28E-01
IMMUNE RESPONSE-ACTIVATING CELL SURFACE RECEPTOR SIGNALING PATHWAY	GO:0002429	189	-0.06	-1.00	4.25E-01	5.28E-01
CELLULAR RESPONSE TO LIGHT STIMULUS	GO:0071482	72	-0.10	-1.00	4.14E-01	5.27E-01
NEGATIVE REGULATION OF TUMOR NECROSIS FACTOR PRODUCTION	GO:0032720	33	-0.15	-1.00	4.73E-01	5.27E-01
PEPTIDYL-CYSTEINE MODIFICATION	GO:0018198	30	-0.15	-1.01	4.18E-01	5.23E-01

'DE NOVO' PROTEIN FOLDING	GO:0006458	35	-0.14	-1.01	4.31E-01	5.22E-01
STRIATED MUSCLE CELL DIFFERENTIATION	GO:0051146	48	-0.12	-1.01	4.40E-01	5.21E-01
PROTEOGLYCAN METABOLIC PROCESS	GO:0006029	41	-0.13	-1.01	4.37E-01	5.22E-01
SPERMATID DEVELOPMENT	GO:0007286	37	-0.14	-1.01	4.47E-01	5.22E-01
NEGATIVE REGULATION OF GROWTH	GO:0045926	117	-0.08	-1.01	4.21E-01	5.19E-01
STRIATED MUSCLE CONTRACTION	GO:0006941	35	-0.14	-1.01	4.28E-01	5.18E-01
GLIAL CELL DEVELOPMENT	GO:0021782	41	-0.13	-1.01	4.40E-01	5.18E-01
REGULATION OF PEPTIDYL-LYSINE ACETYLATION	GO:2000756	44	-0.12	-1.01	4.21E-01	5.18E-01
SPERMATOGENESIS	GO:0007283	121	-0.08	-1.01	4.11E-01	5.18E-01
MORPHOGENESIS OF AN EPITHELIUM	GO:0002009	114	-0.08	-1.01	4.23E-01	5.16E-01
REGULATION OF T CELL ACTIVATION	GO:0050863	200	-0.06	-1.02	4.03E-01	5.11E-01
POSITIVE REGULATION OF LIPID LOCALIZATION	GO:1905954	50	-0.12	-1.02	4.21E-01	5.11E-01
REGULATION OF AXON EXTENSION	GO:0030516	40	-0.13	-1.02	4.00E-01	5.10E-01
REGULATION OF PROTEIN KINASE B SIGNALING	GO:0051896	90	-0.09	-1.02	4.07E-01	5.10E-01
MUSCLE CELL DEVELOPMENT	GO:0055001	41	-0.14	-1.02	4.16E-01	5.09E-01
REGULATION OF MRNA SPLICING, VIA SPLICEOSOME	GO:0048024	71	-0.10	-1.02	4.08E-01	5.09E-01
SKIN DEVELOPMENT	GO:0043588	44	-0.13	-1.02	4.13E-01	5.07E-01
PROTEIN HOMOTETRAMERIZATION	GO:0051289	35	-0.15	-1.03	4.08E-01	4.93E-01
MUSCLE CONTRACTION	GO:0006936	75	-0.10	-1.03	4.05E-01	4.91E-01
SYNAPTIC VESICLE CYCLE	GO:0099504	47	-0.13	-1.03	3.70E-01	4.92E-01
REGULATION OF G2/M TRANSITION OF MITOTIC CELL CYCLE	GO:0010389	63	-0.11	-1.03	4.15E-01	4.91E-01
EPITHELIAL TUBE MORPHOGENESIS	GO:0060562	76	-0.10	-1.03	4.01E-01	4.91E-01
REGULATION OF T CELL DIFFERENTIATION	GO:0045580	81	-0.10	-1.03	3.77E-01	4.91E-01
POSITIVE REGULATION OF BLOOD VESSEL ENDOTHELIAL CELL MIGRATION	GO:0043536	38	-0.14	-1.04	3.88E-01	4.86E-01
REGULATION OF SIGNAL TRANSDUCTION BY P53 CLASS MEDIATOR	GO:1901796	70	-0.11	-1.04	3.93E-01	4.84E-01
CELLULAR AMINO ACID BIOSYNTHETIC PROCESS	GO:0008652	40	-0.14	-1.04	3.68E-01	4.83E-01
ENDOCYTIC RECYCLING	GO:0032456	52	-0.12	-1.04	3.95E-01	4.83E-01
CELLULAR RESPONSE TO TUMOR NECROSIS FACTOR	GO:0071356	78	-0.10	-1.04	3.74E-01	4.78E-01
NUCLEAR EXPORT	GO:0051168	97	-0.09	-1.05	3.80E-01	4.76E-01
REGULATION OF PROTEIN LOCALIZATION TO NUCLEUS	GO:1900180	77	-0.10	-1.05	4.03E-01	4.75E-01
MEMBRANE PROTEIN PROTEOLYSIS	GO:0033619	30	-0.16	-1.05	3.89E-01	4.74E-01
POSITIVE REGULATION OF PEPTIDYL-SERINE PHOSPHORYLATION	GO:0033138	54	-0.12	-1.05	3.77E-01	4.73E-01
SIGNAL TRANSDUCTION BY P53 CLASS MEDIATOR	GO:0072331	59	-0.12	-1.05	3.88E-01	4.72E-01

VESICLE-MEDIATED TRANSPORT IN SYNAPSE	GO:0099003	47	-0.13	-1.05	3.76E-01	4.72E-01
RNA LOCALIZATION	GO:0006403	103	-0.09	-1.05	4.20E-01	4.72E-01
REGULATION OF PRI-MIRNA TRANSCRIPTION BY RNA POLYMERASE II	GO:1902893	32	-0.16	-1.05	3.68E-01	4.71E-01
REGULATION OF CYTOKINE PRODUCTION INVOLVED IN IMMUNE RESPONSE	GO:0002718	63	-0.11	-1.05	3.69E-01	4.71E-01
REGULATION OF ACTIN POLYMERIZATION OR DEPOLYMERIZATION	GO:0008064	117	-0.08	-1.05	3.82E-01	4.71E-01
CELLULAR RESPONSE TO IONIZING RADIATION	GO:0071479	42	-0.14	-1.05	3.78E-01	4.71E-01
CARDIAC CHAMBER MORPHOGENESIS	GO:0003206	33	-0.15	-1.05	3.88E-01	4.70E-01
ORGANIC HYDROXY COMPOUND METABOLIC PROCESS	GO:1901615	186	-0.07	-1.05	3.60E-01	4.68E-01
REGULATION OF CELL AGING	GO:0090342	30	-0.16	-1.06	3.82E-01	4.68E-01
MODULATION OF PROCESS OF OTHER ORGANISM INVOLVED IN SYMBIOTIC INTERACTION	GO:0051817	63	-0.11	-1.06	3.73E-01	4.64E-01
TRANSITION METAL ION HOMEOSTASIS	GO:0055076	77	-0.10	-1.06	3.48E-01	4.64E-01
FATTY ACID DERIVATIVE METABOLIC PROCESS	GO:1901568	48	-0.13	-1.06	3.83E-01	4.64E-01
MITOTIC DNA INTEGRITY CHECKPOINT SIGNALING	GO:0044774	66	-0.11	-1.06	3.52E-01	4.64E-01
CELL-MATRIX ADHESION	GO:0007160	56	-0.12	-1.06	3.64E-01	4.64E-01
REGULATION OF ACTIN FILAMENT ORGANIZATION	GO:0110053	171	-0.07	-1.06	3.55E-01	4.61E-01
REGULATION OF CATION CHANNEL ACTIVITY	GO:2001257	68	-0.11	-1.06	3.65E-01	4.61E-01
POSITIVE REGULATION OF ANGIOGENESIS	GO:0045766	74	-0.11	-1.06	3.75E-01	4.59E-01
REGULATION OF RELEASE OF SEQUESTERED CALCIUM ION INTO CYTOSOL	GO:0051279	32	-0.16	-1.06	3.57E-01	4.59E-01
EMBRYONIC ORGAN MORPHOGENESIS	GO:0048562	50	-0.13	-1.06	3.60E-01	4.60E-01
PROTEIN LOCALIZATION TO LYSOSOME	GO:0061462	34	-0.15	-1.06	3.51E-01	4.60E-01
BONE DEVELOPMENT	GO:0060348	45	-0.13	-1.06	3.45E-01	4.60E-01
GLAND DEVELOPMENT	GO:0048732	63	-0.12	-1.06	3.74E-01	4.60E-01
CARBOHYDRATE HOMEOSTASIS	GO:0033500	73	-0.11	-1.07	3.54E-01	4.60E-01
NEGATIVE REGULATION OF ACTIN FILAMENT POLYMERIZATION	GO:0030837	30	-0.16	-1.07	3.70E-01	4.59E-01
DOUBLE-STRAND BREAK REPAIR VIA NONHOMOLOGOUS END JOINING	GO:0006303	38	-0.15	-1.07	3.47E-01	4.58E-01
POSITIVE REGULATION OF NUCLEOCYTOPLASMIC TRANSPORT	GO:0046824	42	-0.14	-1.07	3.41E-01	4.58E-01
NEGATIVE REGULATION OF MYELOID CELL DIFFERENTIATION	GO:0045638	37	-0.15	-1.07	3.68E-01	4.58E-01
POSITIVE REGULATION OF CELL DEVELOPMENT	GO:0010720	118	-0.08	-1.07	3.70E-01	4.57E-01
LYMPHOCYTE ACTIVATION INVOLVED IN IMMUNE RESPONSE	GO:0002285	51	-0.13	-1.07	3.51E-01	4.50E-01
OSTEOBLAST DIFFERENTIATION	GO:0001649	54	-0.12	-1.08	3.63E-01	4.47E-01
POSITIVE REGULATION OF VASCULATURE DEVELOPMENT	GO:1904018	74	-0.11	-1.08	3.33E-01	4.47E-01
POSITIVE REGULATION OF AXONOGENESIS	GO:0050772	30	-0.17	-1.08	3.54E-01	4.47E-01

REGULATION OF FAT CELL DIFFERENTIATION	GO:0045598	65	-0.12	-1.08	3.54E-01	4.46E-01
REGULATION OF HEART CONTRACTION	GO:0008016	66	-0.12	-1.08	3.34E-01	4.45E-01
RESPONSE TO UV	GO:0009411	111	-0.09	-1.08	3.45E-01	4.45E-01
NEGATIVE REGULATION OF VASCULATURE DEVELOPMENT	GO:1901343	57	-0.12	-1.08	3.41E-01	4.45E-01
MITOTIC DNA DAMAGE CHECKPOINT SIGNALING	GO:0044773	62	-0.11	-1.08	3.54E-01	4.45E-01
REGULATION OF PROTEIN POLYMERIZATION	GO:0032271	148	-0.08	-1.08	3.25E-01	4.45E-01
BIOLOGICAL PROCESS INVOLVED IN INTERACTION WITH HOST	GO:0051701	68	-0.11	-1.08	3.34E-01	4.44E-01
SODIUM ION TRANSPORT	GO:0006814	41	-0.14	-1.08	3.65E-01	4.44E-01
GLIOGENESIS	GO:0042063	69	-0.11	-1.08	3.24E-01	4.43E-01
RIBONUCLEOPROTEIN COMPLEX LOCALIZATION	GO:0071166	62	-0.12	-1.08	3.41E-01	4.41E-01
NEGATIVE REGULATION OF INTRACELLULAR TRANSPORT	GO:0032387	34	-0.16	-1.09	3.36E-01	4.40E-01
ALCOHOL BIOSYNTHETIC PROCESS	GO:0046165	56	-0.12	-1.09	3.53E-01	4.39E-01
RESPONSE TO ACID CHEMICAL	GO:0001101	36	-0.15	-1.09	3.20E-01	4.38E-01
ESTABLISHMENT OR MAINTENANCE OF CELL POLARITY	GO:0007163	111	-0.09	-1.09	3.24E-01	4.36E-01
REGULATION OF POTASSIUM ION TRANSMEMBRANE TRANSPORT	GO:1901379	33	-0.16	-1.09	3.37E-01	4.36E-01
REGULATION OF METAL ION TRANSPORT	GO:0010959	157	-0.07	-1.09	3.22E-01	4.36E-01
BIOLOGICAL PROCESS INVOLVED IN SYMBIOTIC INTERACTION	GO:0044403	122	-0.09	-1.09	3.31E-01	4.35E-01
SPERMATID DIFFERENTIATION	GO:0048515	41	-0.14	-1.09	3.31E-01	4.33E-01
REGULATION OF CALCIUM ION TRANSPORT INTO CYTOSOL	GO:0010522	39	-0.15	-1.09	3.31E-01	4.33E-01
NEGATIVE REGULATION OF ANGIOGENESIS	GO:0016525	57	-0.12	-1.09	3.36E-01	4.32E-01
NEGATIVE REGULATION OF BLOOD VESSEL MORPHOGENESIS	GO:2000181	57	-0.12	-1.09	3.42E-01	4.32E-01
REGULATION OF LYMPHOCYTE PROLIFERATION	GO:0050670	126	-0.08	-1.09	3.31E-01	4.32E-01
GO ID NOT FOUND IN OBO FILE ONTOLOGY DEFINITIONS	GO:0043044	31	-0.17	-1.09	3.31E-01	4.32E-01
REGULATION OF SEQUESTERING OF CALCIUM ION	GO:0051282	52	-0.13	-1.10	3.22E-01	4.29E-01
LIPOPROTEIN METABOLIC PROCESS	GO:0042157	79	-0.10	-1.10	3.14E-01	4.28E-01
REGULATION OF BLOOD VESSEL ENDOTHELIAL CELL MIGRATION	GO:0043535	60	-0.12	-1.10	3.15E-01	4.27E-01
REGULATION OF PROTEIN BINDING	GO:0043393	115	-0.09	-1.10	3.22E-01	4.22E-01
MITOTIC CELL CYCLE CHECKPOINT SIGNALING	GO:0007093	88	-0.10	-1.10	3.05E-01	4.22E-01
POSITIVE REGULATION OF CELL JUNCTION ASSEMBLY	GO:1901890	32	-0.17	-1.10	3.09E-01	4.21E-01
REGULATION OF EXTENT OF CELL GROWTH	GO:0061387	42	-0.14	-1.11	3.26E-01	4.12E-01
POSITIVE REGULATION OF PROTEIN ACETYLATION	GO:1901985	33	-0.16	-1.11	3.02E-01	4.11E-01
ORGANIC HYDROXY COMPOUND BIOSYNTHETIC PROCESS	GO:1901617	82	-0.10	-1.11	3.15E-01	4.12E-01
REGULATION OF SPINDLE ORGANIZATION	GO:0090224	34	-0.16	-1.11	3.05E-01	4.12E-01

CELL-CELL JUNCTION ASSEMBLY	GO:0007043	54	-0.13	-1.12	3.19E-01	4.09E-01
REGULATION OF LIPID KINASE ACTIVITY	GO:0043550	36	-0.16	-1.12	3.32E-01	4.07E-01
DNA METHYLATION OR DEMETHYLATION	GO:0044728	39	-0.15	-1.12	3.09E-01	4.07E-01
REGULATION OF TRANSLATIONAL INITIATION	GO:0006446	66	-0.12	-1.12	2.85E-01	4.07E-01
NEGATIVE REGULATION OF PROTEIN UBIQUITINATION	GO:0031397	60	-0.12	-1.12	2.99E-01	4.07E-01
RIBONUCLEOPROTEIN COMPLEX EXPORT FROM NUCLEUS	GO:0071426	61	-0.12	-1.12	3.07E-01	4.06E-01
GLUCOSE HOMEOSTASIS	GO:0042593	72	-0.11	-1.12	3.11E-01	4.02E-01
POSITIVE REGULATION OF CHEMOTAXIS	GO:0050921	69	-0.11	-1.12	3.05E-01	4.02E-01
STEROID METABOLIC PROCESS	GO:0008202	96	-0.10	-1.12	2.91E-01	4.02E-01
REGULATION OF DNA REPLICATION	GO:0006275	71	-0.11	-1.12	2.90E-01	4.01E-01
NEGATIVE REGULATION OF PROTEIN POLYMERIZATION	GO:0032272	39	-0.15	-1.12	3.09E-01	4.00E-01
POSITIVE REGULATION OF DEVELOPMENTAL GROWTH	GO:0048639	42	-0.15	-1.13	2.82E-01	3.99E-01
STEROL METABOLIC PROCESS	GO:0016125	55	-0.13	-1.13	2.89E-01	3.99E-01
PIGMENTATION	GO:0043473	46	-0.14	-1.13	2.99E-01	3.98E-01
TISSUE MORPHOGENESIS	GO:0048729	146	-0.08	-1.13	2.74E-01	3.97E-01
NEGATIVE REGULATION OF BINDING	GO:0051100	95	-0.10	-1.13	2.69E-01	3.94E-01
PROTEIN DEACETYLATION	GO:0006476	48	-0.14	-1.13	2.86E-01	3.94E-01
REGULATION OF CARDIAC MUSCLE CONTRACTION	GO:0055117	33	-0.17	-1.13	2.95E-01	3.91E-01
PROTEIN ACETYLATION	GO:0006473	97	-0.10	-1.13	2.80E-01	3.91E-01
REGULATION OF FATTY ACID METABOLIC PROCESS	GO:0019217	44	-0.14	-1.13	2.90E-01	3.91E-01
POSITIVE REGULATION OF NIK/NF-KAPPAB SIGNALING	GO:1901224	46	-0.14	-1.13	2.91E-01	3.91E-01
CELLULAR RESPONSE TO TOPOLOGICALLY INCORRECT PROTEIN	GO:0035967	72	-0.11	-1.13	2.88E-01	3.91E-01
PROTEOGLYCAN BIOSYNTHETIC PROCESS	GO:0030166	36	-0.16	-1.14	2.95E-01	3.91E-01
RESPONSE TO XENOBIOTIC STIMULUS	GO:0009410	36	-0.16	-1.14	2.90E-01	3.91E-01
REGULATION OF CENTROSOME CYCLE	GO:0046605	33	-0.16	-1.14	2.93E-01	3.91E-01
RIBONUCLEOSIDE BISPHOSPHATE METABOLIC PROCESS	GO:0033875	83	-0.11	-1.14	2.86E-01	3.91E-01
REGULATION OF MONONUCLEAR CELL PROLIFERATION	GO:0032944	127	-0.09	-1.14	2.86E-01	3.90E-01
NUCLEOSIDE BISPHOSPHATE METABOLIC PROCESS	GO:0033865	83	-0.11	-1.14	2.75E-01	3.90E-01
REGULATION OF ACTIN FILAMENT LENGTH	GO:0030832	118	-0.09	-1.14	2.90E-01	3.90E-01
NEGATIVE REGULATION OF CELL CYCLE G1/S PHASE TRANSITION	GO:1902807	50	-0.14	-1.14	2.91E-01	3.89E-01
PEPTIDYL-LYSINE METHYLATION	GO:0018022	60	-0.12	-1.14	2.83E-01	3.89E-01
POSITIVE REGULATION OF STRESS-ACTIVATED PROTEIN KINASE SIGNALING CASCADE	GO:0070304	80	-0.11	-1.14	2.81E-01	3.89E-01
DNA-TEMPLATED TRANSCRIPTION, INITIATION	GO:0006352	60	-0.13	-1.14	2.92E-01	3.89E-01

PURINE NUCLEOSIDE BISPHOSPHATE METABOLIC PROCESS	GO:0034032	83	-0.11	-1.14	2.79E-01	3.89E-01
POSITIVE REGULATION OF CELL-SUBSTRATE ADHESION	GO:0010811	58	-0.12	-1.14	2.81E-01	3.88E-01
CELLULAR RESPONSE TO AMINO ACID STARVATION	GO:0034198	36	-0.16	-1.14	2.83E-01	3.87E-01
REGULATION OF NEURON DIFFERENTIATION	GO:0045664	46	-0.14	-1.14	2.71E-01	3.87E-01
AMIDE TRANSPORT	GO:0042886	50	-0.14	-1.14	2.88E-01	3.87E-01
PROTEIN ALKYLATION	GO:0008213	88	-0.10	-1.14	2.90E-01	3.86E-01
GLYCOSAMINOGLYCAN METABOLIC PROCESS	GO:0030203	60	-0.12	-1.15	2.73E-01	3.83E-01
REGULATION OF NEUROTRANSMITTER LEVELS	GO:0001505	76	-0.11	-1.15	2.66E-01	3.82E-01
VIRAL LIFE CYCLE	GO:0019058	91	-0.10	-1.15	2.77E-01	3.82E-01
MAINTENANCE OF LOCATION	GO:0051235	107	-0.10	-1.15	2.93E-01	3.81E-01
REGULATION OF BIOLOGICAL PROCESS INVOLVED IN SYMBIOTIC INTERACTION	GO:0043903	51	-0.14	-1.15	2.71E-01	3.79E-01
MESENCHYMAL CELL DIFFERENTIATION	GO:0048762	65	-0.12	-1.15	2.68E-01	3.77E-01
MACROMOLECULE METHYLATION	GO:0043414	174	-0.08	-1.15	2.64E-01	3.76E-01
REGULATION OF DEFENSE RESPONSE TO VIRUS BY HOST	GO:0050691	31	-0.17	-1.16	2.42E-01	3.73E-01
REGULATION OF MITOTIC SISTER CHROMATID SEPARATION	GO:0010965	51	-0.14	-1.16	2.82E-01	3.73E-01
ACTIN POLYMERIZATION OR DEPOLYMERIZATION	GO:0008154	40	-0.16	-1.16	2.63E-01	3.72E-01
MUSCLE ORGAN DEVELOPMENT	GO:0007517	72	-0.11	-1.16	2.74E-01	3.70E-01
PROTEIN METHYLATION	GO:0006479	88	-0.10	-1.16	2.56E-01	3.67E-01
REGULATION OF PROTEIN LOCALIZATION TO MEMBRANE	GO:1905475	108	-0.10	-1.16	2.80E-01	3.67E-01
REGULATION OF PROTEIN SECRETION	GO:0050708	111	-0.09	-1.16	2.58E-01	3.66E-01
REGULATION OF PRODUCTION OF MOLECULAR MEDIATOR OF IMMUNE RESPONSE	GO:0002700	100	-0.10	-1.16	2.61E-01	3.67E-01
ALCOHOL METABOLIC PROCESS	GO:0006066	145	-0.08	-1.16	2.62E-01	3.67E-01
MEIOTIC NUCLEAR DIVISION	GO:0140013	53	-0.14	-1.16	2.48E-01	3.67E-01
POSITIVE REGULATION OF PROTEIN-CONTAINING COMPLEX ASSEMBLY	GO:0031334	162	-0.08	-1.16	2.41E-01	3.66E-01
CELLULAR RESPONSE TO GLUCOSE STARVATION	GO:0042149	35	-0.17	-1.17	2.62E-01	3.65E-01
NEGATIVE REGULATION OF CELLULAR CATABOLIC PROCESS	GO:0031330	173	-0.08	-1.17	2.43E-01	3.65E-01
CELLULAR RESPONSE TO METAL ION	GO:0071248	77	-0.11	-1.17	2.56E-01	3.65E-01
REGULATION OF MYELOID LEUKOCYTE MEDIATED IMMUNITY	GO:0002886	35	-0.16	-1.17	2.44E-01	3.66E-01
SMALL GTPASE MEDIATED SIGNAL TRANSDUCTION	GO:0007264	144	-0.08	-1.17	2.35E-01	3.65E-01
ISOPRENOID METABOLIC PROCESS	GO:0006720	40	-0.16	-1.17	2.54E-01	3.63E-01
MONONUCLEAR CELL PROLIFERATION	GO:0032943	31	-0.18	-1.17	2.43E-01	3.62E-01
REGULATION OF SMOOTHENED SIGNALING PATHWAY	GO:0008589	32	-0.17	-1.17	2.74E-01	3.62E-01
XENOBIOTIC METABOLIC PROCESS	GO:0006805	34	-0.17	-1.17	2.51E-01	3.60E-01

RNA EXPORT FROM NUCLEUS	GO:0006405	63	-0.13	-1.17	2.39E-01	3.60E-01
REGULATION OF CELL MORPHOGENESIS INVOLVED IN DIFFERENTIATION	GO:0010769	53	-0.14	-1.17	2.67E-01	3.60E-01
PROTEIN LOCALIZATION TO CHROMOSOME	GO:0034502	53	-0.13	-1.17	2.33E-01	3.60E-01
SKELETAL SYSTEM DEVELOPMENT	GO:0001501	120	-0.09	-1.17	2.54E-01	3.59E-01
REGULATION OF CHROMOSOME SEPARATION	GO:1905818	56	-0.13	-1.17	2.54E-01	3.59E-01
POSITIVE REGULATION OF LEUKOCYTE MIGRATION	GO:0002687	76	-0.11	-1.18	2.52E-01	3.58E-01
REGULATION OF SODIUM ION TRANSPORT	GO:0002028	34	-0.17	-1.18	2.65E-01	3.58E-01
REGULATION OF PROTEIN ACETYLATION	GO:1901983	51	-0.14	-1.18	2.46E-01	3.57E-01
REGULATION OF IMMUNOGLOBULIN MEDIATED IMMUNE RESPONSE	GO:0002889	31	-0.18	-1.18	2.43E-01	3.58E-01
DNA INTEGRITY CHECKPOINT SIGNALING	GO:0031570	87	-0.11	-1.18	2.46E-01	3.58E-01
MITOCHONDRION LOCALIZATION	GO:0051646	32	-0.18	-1.18	2.63E-01	3.58E-01
POSITIVE REGULATION OF SECRETION BY CELL	GO:1903532	121	-0.09	-1.18	2.57E-01	3.58E-01
POSITIVE REGULATION OF REACTIVE OXYGEN SPECIES METABOLIC PROCESS	GO:2000379	42	-0.15	-1.18	2.47E-01	3.58E-01
REGULATION OF CYCLIN-DEPENDENT PROTEIN KINASE ACTIVITY	GO:1904029	68	-0.12	-1.18	2.58E-01	3.58E-01
REGULATION OF SMALL MOLECULE METABOLIC PROCESS	GO:0062012	164	-0.08	-1.18	2.54E-01	3.59E-01
SENSORY ORGAN MORPHOGENESIS	GO:0090596	48	-0.14	-1.18	2.56E-01	3.59E-01
REGULATION OF STRIATED MUSCLE CONTRACTION	GO:0006942	40	-0.16	-1.18	2.53E-01	3.59E-01
SODIUM ION TRANSMEMBRANE TRANSPORT	GO:0035725	31	-0.17	-1.18	2.57E-01	3.59E-01
POSITIVE REGULATION OF ION TRANSMEMBRANE TRANSPORTER ACTIVITY	GO:0032414	46	-0.15	-1.18	2.54E-01	3.57E-01
REGULATION OF ALPHA-BETA T CELL DIFFERENTIATION	GO:0046637	40	-0.16	-1.18	2.43E-01	3.55E-01
LYMPHOCYTE MIGRATION	GO:0072676	31	-0.18	-1.19	2.34E-01	3.52E-01
REGULATION OF DNA RECOMBINATION	GO:0000018	71	-0.12	-1.19	2.42E-01	3.52E-01
REGULATION OF RECEPTOR SIGNALING PATHWAY VIA STAT	GO:1904892	39	-0.16	-1.19	2.47E-01	3.52E-01
REGULATION OF MITOTIC METAPHASE/ANAPHASE TRANSITION	GO:0030071	48	-0.15	-1.19	2.49E-01	3.51E-01
PROTEIN LOCALIZATION TO CELL PERIPHERY	GO:1990778	141	-0.09	-1.19	2.37E-01	3.48E-01
REGULATION OF ATPASE ACTIVITY	GO:0043462	32	-0.18	-1.19	2.38E-01	3.48E-01
SENSORY PERCEPTION	GO:0007600	101	-0.10	-1.19	2.76E-01	3.48E-01
POSITIVE REGULATION OF HISTONE MODIFICATION	GO:0031058	58	-0.13	-1.19	2.51E-01	3.46E-01
NEGATIVE REGULATION OF EPITHELIAL CELL MIGRATION	GO:0010633	38	-0.16	-1.19	2.37E-01	3.45E-01
POSITIVE REGULATION OF PROTEIN LOCALIZATION TO PLASMA MEMBRANE	GO:1903078	39	-0.16	-1.19	2.32E-01	3.45E-01
RNA CATABOLIC PROCESS	GO:0006401	136	-0.09	-1.19	2.20E-01	3.45E-01
RAS PROTEIN SIGNAL TRANSDUCTION	GO:0007265	115	-0.10	-1.19	2.49E-01	3.45E-01
REGULATION OF PROTEIN MATURATION	GO:1903317	32	-0.18	-1.19	2.48E-01	3.45E-01

POSITIVE REGULATION OF MYELOID CELL DIFFERENTIATION	GO:0045639	57	-0.13	-1.20	2.37E-01	3.44E-01
REGULATION OF POSTSYNAPSE ORGANIZATION	GO:0099175	32	-0.18	-1.20	2.37E-01	3.43E-01
FERTILIZATION	GO:0009566	33	-0.18	-1.20	2.31E-01	3.43E-01
PROTEIN ACYLATION	GO:0043543	133	-0.09	-1.20	2.27E-01	3.40E-01
LEUKOCYTE PROLIFERATION	GO:0070661	34	-0.17	-1.20	2.38E-01	3.39E-01
NEGATIVE REGULATION OF TOR SIGNALING	GO:0032007	34	-0.18	-1.20	2.33E-01	3.39E-01
DNA DAMAGE CHECKPOINT SIGNALING	GO:0000077	81	-0.11	-1.20	2.30E-01	3.38E-01
REGULATION OF CALCIUM ION TRANSPORT	GO:0051924	100	-0.10	-1.20	2.33E-01	3.38E-01
ACYL-COA METABOLIC PROCESS	GO:0006637	63	-0.13	-1.20	2.39E-01	3.37E-01
MEMBRANE LIPID BIOSYNTHETIC PROCESS	GO:0046467	89	-0.11	-1.21	2.13E-01	3.36E-01
CELLULAR RESPONSE TO RADIATION	GO:0071478	106	-0.10	-1.21	2.43E-01	3.36E-01
SELECTIVE AUTOPHAGY	GO:0061912	32	-0.18	-1.21	2.29E-01	3.36E-01
REGULATION OF DEPHOSPHORYLATION	GO:0035303	84	-0.11	-1.21	2.33E-01	3.36E-01
REGULATION OF CALCIUM ION TRANSMEMBRANE TRANSPORTER ACTIVITY	GO:1901019	39	-0.16	-1.21	2.17E-01	3.35E-01
DNA-DEPENDENT DNA REPLICATION	GO:0006261	103	-0.10	-1.21	2.27E-01	3.35E-01
POSITIVE REGULATION OF DNA REPAIR	GO:0045739	58	-0.13	-1.21	2.22E-01	3.36E-01
REGULATION OF SMOOTH MUSCLE CELL PROLIFERATION	GO:0048660	51	-0.14	-1.21	2.28E-01	3.36E-01
DNA GEOMETRIC CHANGE	GO:0032392	37	-0.17	-1.21	2.32E-01	3.34E-01
CELLULAR RESPONSE TO CALCIUM ION	GO:0071277	35	-0.17	-1.21	2.12E-01	3.31E-01
ESTABLISHMENT OF PROTEIN LOCALIZATION TO PLASMA MEMBRANE	GO:0061951	38	-0.16	-1.21	2.18E-01	3.30E-01
THIOESTER METABOLIC PROCESS	GO:0035383	63	-0.13	-1.21	2.16E-01	3.29E-01
EMBRYONIC ORGAN DEVELOPMENT	GO:0048568	89	-0.11	-1.22	2.23E-01	3.26E-01
REPRODUCTIVE STRUCTURE DEVELOPMENT	GO:0048608	60	-0.13	-1.22	2.23E-01	3.26E-01
POSITIVE REGULATION OF PLASMA MEMBRANE BOUNDED CELL PROJECTION ASSEMBLY	GO:0120034	71	-0.12	-1.22	2.22E-01	3.26E-01
POSITIVE REGULATION OF TELOMERE MAINTENANCE	GO:0032206	45	-0.15	-1.22	2.26E-01	3.26E-01
NEGATIVE REGULATION OF NEURON PROJECTION DEVELOPMENT	GO:0010977	49	-0.15	-1.22	2.25E-01	3.24E-01
INTERNAL PROTEIN AMINO ACID ACETYLATION	GO:0006475	81	-0.11	-1.22	2.10E-01	3.23E-01
REGULATION OF P38MAPK CASCADE	GO:1900744	32	-0.18	-1.22	2.17E-01	3.24E-01
METHYLATION	GO:0032259	181	-0.08	-1.22	1.93E-01	3.23E-01
ANTIGEN PROCESSING AND PRESENTATION	GO:0019882	71	-0.12	-1.22	1.88E-01	3.22E-01
REGULATION OF HISTONE MODIFICATION	GO:0031056	93	-0.11	-1.22	2.33E-01	3.22E-01
POSITIVE REGULATION OF SMALL MOLECULE METABOLIC PROCESS	GO:0062013	62	-0.13	-1.22	2.19E-01	3.22E-01

REGULATION OF MICROTUBULE POLYMERIZATION OR DEPOLYMERIZATION	GO:0031110	58	-0.14	-1.23	2.11E-01	3.21E-01
PHAGOCYTOSIS	GO:0006909	151	-0.09	-1.23	2.21E-01	3.19E-01
RESPONSE TO AMINO ACID STARVATION	GO:1990928	37	-0.17	-1.23	2.05E-01	3.19E-01
POSITIVE REGULATION OF TELOMERE MAINTENANCE VIA TELOMERASE	GO:0032212	30	-0.19	-1.23	2.20E-01	3.19E-01
REGULATION OF CD4-POSITIVE, ALPHA-BETA T CELL DIFFERENTIATION	GO:0043370	34	-0.17	-1.23	2.07E-01	3.19E-01
REGULATION OF CHEMOTAXIS	GO:0050920	110	-0.10	-1.23	2.24E-01	3.17E-01
POSITIVE REGULATION OF EXOCYTOSIS	GO:0045921	51	-0.14	-1.23	1.90E-01	3.16E-01
REGULATION OF PROTEIN IMPORT INTO NUCLEUS	GO:0042306	30	-0.19	-1.23	2.10E-01	3.17E-01
CARDIAC VENTRICLE DEVELOPMENT	GO:0003231	34	-0.18	-1.23	2.24E-01	3.17E-01
FATTY ACID DERIVATIVE BIOSYNTHETIC PROCESS	GO:1901570	33	-0.18	-1.23	1.86E-01	3.16E-01
VIRAL PROCESS	GO:0016032	157	-0.09	-1.23	1.98E-01	3.17E-01
PROTEIN DEACYLATION	GO:0035601	56	-0.14	-1.23	1.94E-01	3.16E-01
REGULATION OF TUMOR NECROSIS FACTOR-MEDIATED SIGNALING PATHWAY	GO:0010803	33	-0.18	-1.23	2.17E-01	3.17E-01
MULTIVESICULAR BODY SORTING PATHWAY	GO:0071985	35	-0.17	-1.23	2.31E-01	3.16E-01
REGULATION OF RECEPTOR SIGNALING PATHWAY VIA JAK-STAT	GO:0046425	36	-0.17	-1.23	1.91E-01	3.16E-01
REGULATION OF T CELL PROLIFERATION	GO:0042129	92	-0.11	-1.24	2.03E-01	3.15E-01
REPRODUCTIVE SYSTEM DEVELOPMENT	GO:0061458	60	-0.13	-1.24	2.16E-01	3.16E-01
REGULATION OF LEUKOCYTE PROLIFERATION	GO:0070663	136	-0.09	-1.24	2.04E-01	3.16E-01
NEGATIVE REGULATION OF PEPTIDYL-TYROSINE PHOSPHORYLATION	GO:0050732	37	-0.17	-1.24	2.15E-01	3.16E-01
REGULATION OF PH	GO:0006885	51	-0.15	-1.24	2.06E-01	3.16E-01
REGULATION OF DEFENSE RESPONSE TO VIRUS	GO:0050688	50	-0.15	-1.24	2.06E-01	3.16E-01
CAMERA-TYPE EYE DEVELOPMENT	GO:0043010	55	-0.14	-1.24	1.87E-01	3.16E-01
REGULATION OF PHOSPHOPROTEIN PHOSPHATASE ACTIVITY	GO:0043666	41	-0.17	-1.24	2.09E-01	3.16E-01
RESPONSE TO TOPOLOGICALLY INCORRECT PROTEIN	GO:0035966	90	-0.11	-1.24	1.96E-01	3.13E-01
ESTABLISHMENT OF CELL POLARITY	GO:0030010	64	-0.13	-1.24	2.13E-01	3.13E-01
MUSCLE STRUCTURE DEVELOPMENT	GO:0061061	135	-0.09	-1.24	1.91E-01	3.13E-01
REGULATION OF B CELL MEDIATED IMMUNITY	GO:0002712	32	-0.19	-1.24	1.96E-01	3.13E-01
HISTONE DEACETYLATION	GO:0016575	43	-0.16	-1.24	1.86E-01	3.11E-01
INORGANIC CATION TRANSMEMBRANE TRANSPORT	GO:0098662	183	-0.08	-1.25	1.89E-01	3.09E-01
REGULATION OF NERVOUS SYSTEM PROCESS	GO:0031644	40	-0.16	-1.25	1.72E-01	3.09E-01
POSITIVE REGULATION OF ION TRANSMEMBRANE TRANSPORT	GO:0034767	66	-0.13	-1.25	2.12E-01	3.08E-01
CELLULAR RESPONSE TO OXIDATIVE STRESS	GO:0034599	130	-0.09	-1.25	1.78E-01	3.08E-01
POSITIVE REGULATION OF CATION TRANSMEMBRANE TRANSPORT	GO:1904064	57	-0.14	-1.25	1.93E-01	3.08E-01

CELLULAR BIOGENIC AMINE METABOLIC PROCESS	GO:0006576	37	-0.18	-1.25	2.08E-01	3.07E-01
MRNA-CONTAINING RIBONUCLEOPROTEIN COMPLEX EXPORT FROM NUCLEUS	GO:0071427	45	-0.16	-1.25	1.81E-01	3.08E-01
REGULATION OF STEROID METABOLIC PROCESS	GO:0019218	40	-0.17	-1.25	1.93E-01	3.08E-01
POSITIVE REGULATION OF EPITHELIAL CELL PROLIFERATION	GO:0050679	69	-0.13	-1.25	2.01E-01	3.07E-01
RESPONSE TO PURINE-CONTAINING COMPOUND	GO:0014074	39	-0.17	-1.25	1.97E-01	3.07E-01
MITOTIC G2/M TRANSITION CHECKPOINT	GO:0044818	38	-0.18	-1.25	1.80E-01	3.06E-01
CELLULAR RESPONSE TO VIRUS	GO:0098586	34	-0.18	-1.25	1.98E-01	3.06E-01
GLYCOSAMINOGLYCAN BIOSYNTHETIC PROCESS	GO:0006024	46	-0.15	-1.25	1.81E-01	3.06E-01
REGULATION OF CYCLIN-DEPENDENT PROTEIN SERINE/THREONINE KINASE ACTIVITY	GO:0000079	66	-0.13	-1.25	1.80E-01	3.07E-01
MACROMOLECULE DEACYLATION	GO:0098732	56	-0.14	-1.25	1.96E-01	3.07E-01
CELL CYCLE CHECKPOINT SIGNALING	GO:0000075	109	-0.10	-1.25	1.95E-01	3.07E-01
PLASMA MEMBRANE ORGANIZATION	GO:0007009	61	-0.14	-1.25	1.81E-01	3.05E-01
HEART VALVE DEVELOPMENT	GO:0003170	30	-0.19	-1.26	1.95E-01	3.01E-01
CELLULAR AMINE METABOLIC PROCESS	GO:0044106	37	-0.18	-1.26	1.85E-01	3.01E-01
POLYOL METABOLIC PROCESS	GO:0019751	56	-0.14	-1.26	2.02E-01	3.00E-01
RECOMBINATION	GO:0010569	39	-0.16	-1.26	1.88E-01	3.00E-01
POSITIVE REGULATION OF DOUBLE-STRAND BREAK REPAIR	GO:2000781	33	-0.19	-1.26	1.92E-01	3.00E-01
CELLULAR RESPONSE TO XENOBIOTIC STIMULUS	GO:0071466	35	-0.18	-1.26	1.97E-01	2.99E-01
REGULATION OF LYMPHOCYTE DIFFERENTIATION	GO:0045619	96	-0.11	-1.26	1.98E-01	2.95E-01
REGULATION OF ENDOTHELIAL CELL MIGRATION	GO:0010594	104	-0.11	-1.27	1.73E-01	2.95E-01
TRANSCRIPTION INITIATION FROM RNA POLYMERASE II PROMOTER	GO:0006367	39	-0.17	-1.27	1.84E-01	2.95E-01
G1/S TRANSITION OF MITOTIC CELL CYCLE	GO:0000082	57	-0.14	-1.27	1.83E-01	2.92E-01
POSITIVE REGULATION OF INTERFERON-GAMMA PRODUCTION	GO:0032729	45	-0.16	-1.27	1.90E-01	2.92E-01
POSITIVE REGULATION OF LIPID METABOLIC PROCESS	GO:0045834	74	-0.13	-1.27	1.61E-01	2.91E-01
INTERNAL PEPTIDYL-LYSINE ACETYLATION	GO:0018393	79	-0.12	-1.27	1.84E-01	2.91E-01
CELLULAR RESPONSE TO UNFOLDED PROTEIN	GO:0034620	54	-0.15	-1.27	1.79E-01	2.90E-01
EXTRACELLULAR MATRIX ORGANIZATION	GO:0030198	76	-0.13	-1.27	1.75E-01	2.90E-01
EXTRACELLULAR STRUCTURE ORGANIZATION	GO:0043062	76	-0.13	-1.27	1.88E-01	2.90E-01
MONOVALENT INORGANIC CATION HOMEOSTASIS	GO:0055067	68	-0.13	-1.27	1.97E-01	2.90E-01
ENTRY INTO HOST	GO:0044409	31	-0.19	-1.27	1.69E-01	2.90E-01
POSITIVE REGULATION OF CYSTEINE-TYPE ENDOPEPTIDASE ACTIVITY INVOLVED IN APOPTOTIC PROCESS	GO:0043280	92	-0.11	-1.27	1.72E-01	2.90E-01
REGULATION OF EXTRINSIC APOPTOTIC SIGNALING PATHWAY	GO:2001236	83	-0.11	-1.27	1.84E-01	2.89E-01

RESPONSE TO ORGANOPHOSPHORUS	GO:0046683	39	-0.17	-1.28	1.73E-01	2.87E-01
POSITIVE REGULATION OF CELL CYCLE PHASE TRANSITION	GO:1901989	68	-0.13	-1.28	1.91E-01	2.87E-01
CALCIUM ION TRANSMEMBRANE TRANSPORT	GO:0070588	66	-0.14	-1.28	1.66E-01	2.84E-01
POSITIVE REGULATION OF T CELL MEDIATED IMMUNITY	GO:0002711	38	-0.17	-1.28	1.74E-01	2.84E-01
FAT CELL DIFFERENTIATION	GO:0045444	42	-0.17	-1.28	1.74E-01	2.84E-01
MRNA EXPORT FROM NUCLEUS	GO:0006406	45	-0.16	-1.28	1.48E-01	2.81E-01
MOVEMENT IN HOST ENVIRONMENT	GO:0052126	41	-0.17	-1.28	1.55E-01	2.81E-01
CELLULAR RESPONSE TO INORGANIC SUBSTANCE	GO:0071241	89	-0.12	-1.28	1.76E-01	2.81E-01
PEPTIDYL-LYSINE ACETYLATION	GO:0018394	82	-0.12	-1.29	1.72E-01	2.81E-01
EPITHELIAL TO MESENCHYMAL TRANSITION	GO:0001837	41	-0.17	-1.29	1.64E-01	2.80E-01
RETROGRADE TRANSPORT, ENDOSOME TO GOLGI	GO:0042147	83	-0.12	-1.29	1.70E-01	2.76E-01
HEART MORPHOGENESIS	GO:0003007	68	-0.14	-1.29	1.68E-01	2.76E-01
CELL JUNCTION ORGANIZATION	GO:0034330	180	-0.08	-1.29	1.56E-01	2.75E-01
AMINOGLYCAN METABOLIC PROCESS	GO:0006022	67	-0.13	-1.29	1.72E-01	2.75E-01
REGULATION OF INTRACELLULAR PH	GO:0051453	46	-0.16	-1.29	1.66E-01	2.76E-01
ANION TRANSMEMBRANE TRANSPORT	GO:0098656	75	-0.13	-1.30	1.58E-01	2.72E-01
CELLULAR RESPONSE TO OXYGEN LEVELS	GO:0071453	71	-0.13	-1.30	1.57E-01	2.69E-01
NEGATIVE REGULATION OF PROTEIN MODIFICATION BY SMALL PROTEIN CONJUGATION OR REMOVAL	GO:1903321	66	-0.14	-1.30	1.60E-01	2.69E-01
REGULATION OF ERBB SIGNALING PATHWAY	GO:1901184	53	-0.15	-1.30	1.66E-01	2.69E-01
CELL CYCLE G1/S PHASE TRANSITION	GO:0044843	59	-0.14	-1.30	1.68E-01	2.70E-01
AMINO ACID TRANSMEMBRANE TRANSPORT	GO:0003333	34	-0.19	-1.30	1.65E-01	2.68E-01
REGULATION OF METAPHASE/ANAPHASE TRANSITION OF CELL CYCLE	GO:1902099	49	-0.16	-1.30	1.70E-01	2.65E-01
LYMPHOCYTE DIFFERENTIATION	GO:0030098	110	-0.11	-1.30	1.67E-01	2.65E-01
REGULATION OF PROTEIN IMPORT	GO:1904589	33	-0.19	-1.31	1.48E-01	2.64E-01
POSITIVE REGULATION OF GROWTH	GO:0045927	95	-0.12	-1.31	1.50E-01	2.64E-01
REGULATION OF PLASMA MEMBRANE BOUNDED CELL PROJECTION ASSEMBLY	GO:0120032	142	-0.10	-1.31	1.56E-01	2.61E-01
NEGATIVE REGULATION OF CYSTEINE-TYPE ENDOPEPTIDASE ACTIVITY INVOLVED IN APOPTOTIC PROCESS	GO:0043154	44	-0.17	-1.31	1.52E-01	2.59E-01
MITOTIC CELL CYCLE PHASE TRANSITION	GO:0044772	115	-0.10	-1.31	1.54E-01	2.59E-01
REGULATION OF FILOPODIUM ASSEMBLY	GO:0051489	31	-0.19	-1.31	1.41E-01	2.59E-01
REGULATION OF RESPONSE TO ENDOPLASMIC RETICULUM STRESS	GO:1905897	64	-0.14	-1.31	1.51E-01	2.58E-01
POSITIVE REGULATION OF SECRETION	GO:0051047	124	-0.10	-1.31	1.42E-01	2.58E-01

NEGATIVE REGULATION OF G1/S TRANSITION OF MITOTIC CELL CYCLE	GO:2000134	47	-0.16	-1.31	1.41E-01	2.58E-01
REGULATION OF NEURON DEATH	GO:1901214	114	-0.11	-1.31	1.53E-01	2.58E-01
REGULATION OF ENDOTHELIAL CELL PROLIFERATION	GO:0001936	65	-0.14	-1.32	1.50E-01	2.57E-01
NEGATIVE REGULATION OF ORGANELLE ASSEMBLY	GO:1902116	33	-0.19	-1.32	1.48E-01	2.57E-01
CARBOHYDRATE DERIVATIVE CATABOLIC PROCESS	GO:1901136	106	-0.11	-1.32	1.62E-01	2.57E-01
CEREBRAL CORTEX DEVELOPMENT	GO:0021987	30	-0.20	-1.32	1.65E-01	2.57E-01
NEGATIVE REGULATION OF SMALL MOLECULE METABOLIC PROCESS	GO:0062014	44	-0.17	-1.32	1.66E-01	2.56E-01
REGULATION OF OSTEOBLAST DIFFERENTIATION	GO:0045667	46	-0.16	-1.32	1.33E-01	2.55E-01
POLYSACCHARIDE METABOLIC PROCESS	GO:0005976	31	-0.20	-1.32	1.49E-01	2.56E-01
CELLULAR RESPONSE TO LIPOPOLYSACCHARIDE	GO:0071222	91	-0.12	-1.32	1.40E-01	2.56E-01
CILIUM MOVEMENT	GO:0003341	35	-0.19	-1.32	1.47E-01	2.56E-01
REGULATION OF PLASMA LIPOPROTEIN PARTICLE LEVELS	GO:0097006	31	-0.20	-1.32	1.48E-01	2.56E-01
CYTOPLASMIC MICROTUBULE ORGANIZATION	GO:0031122	51	-0.16	-1.32	1.55E-01	2.56E-01
REGULATION OF PEPTIDYL-SERINE PHOSPHORYLATION	GO:0033135	69	-0.13	-1.32	1.37E-01	2.54E-01
REGULATION OF TRANSPORTER ACTIVITY	GO:0032409	128	-0.11	-1.32	1.62E-01	2.54E-01
MEIOTIC CELL CYCLE PROCESS	GO:1903046	63	-0.14	-1.32	1.53E-01	2.53E-01
REGULATION OF BLOOD CIRCULATION	GO:1903522	77	-0.13	-1.32	1.58E-01	2.53E-01
REGULATION OF DNA-TEMPLATED TRANSCRIPTION, ELONGATION	GO:0032784	43	-0.17	-1.33	1.51E-01	2.52E-01
REGULATION OF TRANSMEMBRANE TRANSPORTER ACTIVITY	GO:0022898	118	-0.10	-1.33	1.37E-01	2.51E-01
SENSORY PERCEPTION OF LIGHT STIMULUS	GO:0050953	39	-0.18	-1.33	1.38E-01	2.51E-01
CELL JUNCTION ASSEMBLY	GO:0034329	100	-0.11	-1.33	1.52E-01	2.51E-01
REGULATION OF CELLULAR PH	GO:0030641	49	-0.16	-1.33	1.48E-01	2.52E-01
REGULATION OF CENTROSOME DUPLICATION	GO:0010824	30	-0.21	-1.33	1.45E-01	2.50E-01
CARBOXYLIC ACID TRANSPORT	GO:0046942	84	-0.12	-1.33	1.43E-01	2.50E-01
CELLULAR RESPONSE TO REACTIVE OXYGEN SPECIES	GO:0034614	66	-0.14	-1.33	1.56E-01	2.50E-01
NEURON MIGRATION	GO:0001764	32	-0.20	-1.33	1.40E-01	2.49E-01
LYMPHOCYTE PROLIFERATION	GO:0046651	30	-0.20	-1.33	1.55E-01	2.48E-01
ACTOMYOSIN STRUCTURE ORGANIZATION	GO:0031032	55	-0.15	-1.33	1.43E-01	2.47E-01
POSITIVE REGULATION OF PROTEIN LOCALIZATION TO CELL PERIPHERY	GO:1904377	44	-0.17	-1.33	1.41E-01	2.47E-01
NEGATIVE REGULATION OF PROTEIN-CONTAINING COMPLEX ASSEMBLY	GO:0031333	78	-0.13	-1.34	1.37E-01	2.46E-01
NEGATIVE REGULATION OF SMALL GTPASE MEDIATED SIGNAL TRANSDUCTION	GO:0051058	31	-0.20	-1.34	1.33E-01	2.46E-01
REGULATION OF CELL PROJECTION ASSEMBLY	GO:0060491	142	-0.10	-1.34	1.34E-01	2.46E-01
POSITIVE REGULATION OF CYSTEINE-TYPE ENDOPEPTIDASE ACTIVITY	GO:2001056	105	-0.11	-1.34	1.22E-01	2.43E-01

SIGNAL TRANSDUCTION IN RESPONSE TO DNA DAMAGE	GO:0042770	104	-0.11	-1.34	1.37E-01	2.43E-01
NEGATIVE REGULATION OF CATION TRANSMEMBRANE TRANSPORT	GO:1904063	41	-0.17	-1.34	1.58E-01	2.40E-01
RNA TRANSPORT	GO:0050658	81	-0.13	-1.35	1.30E-01	2.39E-01
HISTONE METHYLATION	GO:0016571	61	-0.15	-1.35	1.29E-01	2.39E-01
REGULATION OF CELLULAR KETONE METABOLIC PROCESS	GO:0010565	66	-0.14	-1.35	1.35E-01	2.40E-01
REGULATION OF CATION TRANSMEMBRANE TRANSPORT	GO:1904062	145	-0.10	-1.35	1.45E-01	2.39E-01
REGULATION OF NUCLEOCYTOPLASMIC TRANSPORT	GO:0046822	64	-0.14	-1.35	1.13E-01	2.37E-01
POSITIVE REGULATION OF TELOMERE MAINTENANCE VIA TELOMERE LENGTHENING	GO:1904358	32	-0.20	-1.35	1.27E-01	2.36E-01
NEGATIVE REGULATION OF SUPRAMOLECULAR FIBER ORGANIZATION	GO:1902904	85	-0.12	-1.35	1.09E-01	2.35E-01
REGULATION OF CELLULAR RESPONSE TO OXIDATIVE STRESS	GO:1900407	44	-0.17	-1.35	1.26E-01	2.33E-01
REGULATION OF INTERFERON-GAMMA PRODUCTION	GO:0032649	67	-0.14	-1.36	1.06E-01	2.27E-01
REGULATION OF HEMOPOIESIS	GO:1903706	200	-0.08	-1.36	1.36E-01	2.27E-01
REGULATION OF DOUBLE-STRAND BREAK REPAIR	GO:2000779	67	-0.14	-1.36	1.20E-01	2.24E-01
NEGATIVE REGULATION OF PROTEOLYSIS	GO:0045861	136	-0.10	-1.37	1.01E-01	2.23E-01
POSITIVE REGULATION OF TRANSMEMBRANE RECEPTOR PROTEIN SERINE/THREONINE KINASE SIGNALING PATHWAY	GO:0090100	52	-0.16	-1.37	1.29E-01	2.23E-01
POSITIVE REGULATION OF MITOTIC CELL CYCLE PHASE TRANSITION	GO:1901992	53	-0.16	-1.37	1.09E-01	2.23E-01
POSITIVE REGULATION OF CYTOKINE PRODUCTION INVOLVED IN IMMUNE RESPONSE	GO:0002720	46	-0.17	-1.37	1.33E-01	2.22E-01
DOUBLE-STRAND BREAK REPAIR	GO:0006302	150	-0.10	-1.37	1.20E-01	2.21E-01
POSITIVE REGULATION OF VIRAL PROCESS	GO:0048524	55	-0.15	-1.37	1.19E-01	2.19E-01
DEVELOPMENTAL PROCESS INVOLVED IN REPRODUCTION	GO:0003006	194	-0.09	-1.37	1.24E-01	2.18E-01
REGULATION OF POSTTRANSCRIPTIONAL GENE SILENCING	GO:0060147	30	-0.21	-1.37	1.14E-01	2.18E-01
REGULATION OF JNK CASCADE	GO:0046328	89	-0.13	-1.37	1.28E-01	2.18E-01
CELL FATE COMMITMENT	GO:0045165	62	-0.15	-1.37	1.32E-01	2.18E-01
RNA STABILIZATION	GO:0043489	39	-0.19	-1.37	1.24E-01	2.18E-01
REGULATION OF TRANSCRIPTION FROM RNA POLYMERASE II PROMOTER IN RESPONSE TO STRESS	GO:0043618	37	-0.19	-1.38	1.08E-01	2.18E-01
REGULATION OF BMP SIGNALING PATHWAY	GO:0030510	36	-0.20	-1.38	9.90E-02	2.18E-01
HISTONE ACETYLATION	GO:0016573	76	-0.13	-1.38	1.21E-01	2.18E-01
NUCLEIC ACID TRANSPORT	GO:0050657	81	-0.13	-1.38	1.04E-01	2.18E-01
BMP SIGNALING PATHWAY	GO:0030509	34	-0.20	-1.38	1.10E-01	2.19E-01
NEGATIVE REGULATION OF CYTOKINE PRODUCTION	GO:0001818	161	-0.09	-1.38	1.02E-01	2.18E-01
EXTERNAL ENCAPSULATING STRUCTURE ORGANIZATION	GO:0045229	77	-0.13	-1.38	1.24E-01	2.17E-01

POSITIVE REGULATION OF DNA METABOLIC PROCESS	GO:0051054	145	-0.10	-1.38	1.06E-01	2.17E-01
POSITIVE REGULATION OF MONONUCLEAR CELL MIGRATION	GO:0071677	41	-0.19	-1.38	1.29E-01	2.17E-01
NEGATIVE REGULATION OF EXTRINSIC APOPTOTIC SIGNALING PATHWAY	GO:2001237	51	-0.16	-1.38	1.30E-01	2.17E-01
CELLULAR RESPONSE TO BMP STIMULUS	GO:0071773	34	-0.20	-1.38	1.29E-01	2.17E-01
RESPONSE TO BMP	GO:0071772	34	-0.20	-1.38	1.22E-01	2.15E-01
REGULATION OF POTASSIUM ION TRANSPORT	GO:0043266	36	-0.19	-1.38	1.03E-01	2.14E-01
RESPONSE TO HYDROGEN PEROXIDE	GO:0042542	48	-0.17	-1.38	1.09E-01	2.14E-01
CELLULAR COMPONENT ASSEMBLY INVOLVED IN MORPHOGENESIS	GO:0010927	34	-0.20	-1.38	1.25E-01	2.13E-01
SPLICEOSOMAL COMPLEX ASSEMBLY	GO:0000245	47	-0.17	-1.39	1.08E-01	2.13E-01
REGULATION OF CELL CYCLE G1/S PHASE TRANSITION	GO:1902806	95	-0.12	-1.39	9.86E-02	2.11E-01
MRNA CATABOLIC PROCESS	GO:0006402	110	-0.11	-1.39	1.23E-01	2.11E-01
REGULATION OF I-KAPPAB KINASE/NF-KAPPAB SIGNALING	GO:0043122	192	-0.09	-1.39	1.13E-01	2.11E-01
CENTROSOME CYCLE	GO:0007098	68	-0.14	-1.39	1.09E-01	2.10E-01
DEFENSE RESPONSE TO GRAM-POSITIVE BACTERIUM	GO:0050830	35	-0.19	-1.39	1.12E-01	2.09E-01
REGULATION OF GENE SILENCING BY RNA	GO:0060966	30	-0.21	-1.39	1.14E-01	2.09E-01
LYSOSOME ORGANIZATION	GO:0007040	47	-0.18	-1.39	1.23E-01	2.08E-01
SKELETAL SYSTEM MORPHOGENESIS	GO:0048705	36	-0.19	-1.39	1.15E-01	2.09E-01
REGULATION OF SISTER CHROMATID SEGREGATION	GO:0033045	57	-0.16	-1.39	1.16E-01	2.09E-01
AMINOGLYCAN BIOSYNTHETIC PROCESS	GO:0006023	48	-0.17	-1.39	1.22E-01	2.09E-01
IN UTERO EMBRYONIC DEVELOPMENT	GO:0001701	30	-0.22	-1.40	1.23E-01	2.08E-01
REGULATION OF CARBOHYDRATE CATABOLIC PROCESS	GO:0043470	33	-0.20	-1.40	9.26E-02	2.04E-01
INTRINSIC APOPTOTIC SIGNALING PATHWAY	GO:0097193	102	-0.12	-1.40	1.22E-01	2.03E-01
ORGANIC ACID TRANSPORT	GO:0015849	102	-0.12	-1.40	1.02E-01	2.02E-01
REGULATION OF OXIDATIVE STRESS-INDUCED CELL DEATH	GO:1903201	38	-0.19	-1.40	9.72E-02	2.01E-01
POSITIVE REGULATION OF LEUKOCYTE CHEMOTAXIS	GO:0002690	53	-0.17	-1.41	1.11E-01	2.01E-01
REGULATION OF CYSTEINE-TYPE ENDOPEPTIDASE ACTIVITY	GO:2000116	154	-0.10	-1.41	1.02E-01	2.01E-01
RESPONSE TO UNFOLDED PROTEIN	GO:0006986	70	-0.15	-1.41	1.02E-01	2.01E-01
MAINTENANCE OF LOCATION IN CELL	GO:0051651	64	-0.15	-1.41	1.01E-01	2.01E-01
T CELL RECEPTOR SIGNALING PATHWAY	GO:0050852	68	-0.14	-1.41	1.15E-01	2.01E-01
REGULATION OF DNA-TEMPLATED TRANSCRIPTION IN RESPONSE TO STRESS	GO:0043620	42	-0.18	-1.41	1.01E-01	2.01E-01
INORGANIC ION IMPORT ACROSS PLASMA MEMBRANE	GO:0099587	34	-0.21	-1.41	1.14E-01	2.00E-01
APOPTOTIC SIGNALING PATHWAY	GO:0097190	185	-0.09	-1.41	9.27E-02	2.00E-01
RESPONSE TO CALCIUM ION	GO:0051592	58	-0.16	-1.41	1.13E-01	1.97E-01

EPIDERMAL CELL DIFFERENTIATION	GO:0009913	34	-0.21	-1.41	1.05E-01	1.98E-01
CELLULAR POLYSACCHARIDE METABOLIC PROCESS	GO:0044264	30	-0.22	-1.41	8.97E-02	1.97E-01
FATTY-ACYL-COA METABOLIC PROCESS	GO:0035337	31	-0.21	-1.41	1.04E-01	1.96E-01
MICROTUBULE ORGANIZING CENTER ORGANIZATION	GO:0031023	80	-0.14	-1.41	1.00E-01	1.96E-01
T CELL ACTIVATION	GO:0042110	115	-0.11	-1.42	1.08E-01	1.96E-01
MODULATION OF PROCESS OF OTHER ORGANISM	GO:0035821	76	-0.14	-1.42	7.84E-02	1.96E-01
REGULATION OF PROTEIN PROCESSING	GO:0070613	30	-0.22	-1.42	1.03E-01	1.96E-01
POSITIVE REGULATION OF LEUKOCYTE MEDIATED CYTOTOXICITY	GO:0001912	33	-0.20	-1.42	8.50E-02	1.96E-01
REGULATION OF ALPHA-BETA T CELL ACTIVATION	GO:0046634	64	-0.15	-1.42	8.67E-02	1.96E-01
REGULATION OF LEUKOCYTE MIGRATION	GO:0002685	120	-0.11	-1.42	9.30E-02	1.95E-01
ESTABLISHMENT OF PROTEIN LOCALIZATION TO EXTRACELLULAR REGION	GO:0035592	49	-0.18	-1.42	9.43E-02	1.95E-01
RESPONSE TO IONIZING RADIATION	GO:0010212	81	-0.13	-1.42	9.68E-02	1.94E-01
INORGANIC CATION IMPORT ACROSS PLASMA MEMBRANE	GO:0098659	34	-0.21	-1.42	1.05E-01	1.94E-01
NEGATIVE REGULATION OF CYSTEINE-TYPE ENDOPEPTIDASE ACTIVITY	GO:2000117	50	-0.17	-1.42	9.76E-02	1.92E-01
POSITIVE REGULATION OF I-KAPPAB KINASE/NF-KAPPAB SIGNALING	GO:0043123	145	-0.10	-1.43	1.08E-01	1.91E-01
POSITIVE REGULATION OF MITOTIC CELL CYCLE	GO:0045931	71	-0.14	-1.43	9.36E-02	1.91E-01
POSITIVE REGULATION OF PEPTIDASE ACTIVITY	GO:0010952	130	-0.11	-1.43	8.55E-02	1.91E-01
CERAMIDE METABOLIC PROCESS	GO:0006672	57	-0.16	-1.43	8.98E-02	1.91E-01
NEGATIVE REGULATION OF LIPID METABOLIC PROCESS	GO:0045833	50	-0.17	-1.43	9.18E-02	1.91E-01
NEGATIVE REGULATION OF NEUROGENESIS	GO:0050768	47	-0.18	-1.43	1.06E-01	1.91E-01
POSITIVE REGULATION OF ERK1 AND ERK2 CASCADE	GO:0070374	91	-0.13	-1.43	9.83E-02	1.91E-01
POSITIVE REGULATION OF CELL KILLING	GO:0031343	39	-0.20	-1.43	1.12E-01	1.91E-01
EXTRINSIC APOPTOTIC SIGNALING PATHWAY	GO:0097191	60	-0.16	-1.43	1.16E-01	1.91E-01
REGULATION OF CALCIUM ION TRANSMEMBRANE TRANSPORT	GO:1903169	61	-0.16	-1.43	1.01E-01	1.91E-01
CHORDATE EMBRYONIC DEVELOPMENT	GO:0043009	90	-0.13	-1.43	9.62E-02	1.91E-01
NEGATIVE REGULATION OF GENE EXPRESSION, EPIGENETIC	GO:0045814	30	-0.22	-1.43	1.06E-01	1.90E-01
CELLULAR RESPONSE TO HYPOXIA	GO:0071456	57	-0.16	-1.43	8.51E-02	1.90E-01
CELLULAR RESPONSE TO INTERLEUKIN-1	GO:0071347	45	-0.18	-1.43	9.70E-02	1.90E-01
AXON GUIDANCE	GO:0007411	82	-0.14	-1.43	1.03E-01	1.90E-01
CELL MATURATION	GO:0048469	31	-0.21	-1.43	9.75E-02	1.90E-01
NEURON PROJECTION GUIDANCE	GO:0097485	82	-0.14	-1.43	1.07E-01	1.89E-01
PATHWAY	GO:0033143	48	-0.17	-1.44	9.50E-02	1.87E-01
VESICLE TRANSPORT ALONG MICROTUBULE	GO:0047496	33	-0.21	-1.44	8.79E-02	1.86E-01

PROTEIN-CONTAINING COMPLEX LOCALIZATION	GO:0031503	123	-0.11	-1.44	8.04E-02	1.86E-01
ENDOTHELIUM DEVELOPMENT	GO:0003158	47	-0.18	-1.44	8.69E-02	1.86E-01
LYTIC VACUOLE ORGANIZATION	GO:0080171	47	-0.18	-1.44	9.15E-02	1.85E-01
INTEGRIN-MEDIATED SIGNALING PATHWAY	GO:0007229	51	-0.17	-1.44	1.04E-01	1.84E-01
REGULATION OF BLOOD PRESSURE	GO:0008217	53	-0.17	-1.44	9.16E-02	1.83E-01
RESPONSE TO LIPOPOLYSACCHARIDE	GO:0032496	126	-0.11	-1.44	8.04E-02	1.82E-01
O-GLYCAN PROCESSING	GO:0016266	30	-0.23	-1.44	8.50E-02	1.82E-01
REGULATION OF CYSTEINE-TYPE ENDOPEPTIDASE ACTIVITY INVOLVED IN APOPTOTIC PROCESS	GO:0043281	134	-0.11	-1.45	9.70E-02	1.82E-01
REGULATION OF CELL SIZE	GO:0008361	77	-0.14	-1.45	8.65E-02	1.82E-01
DNA RECOMBINATION	GO:0006310	123	-0.11	-1.45	9.98E-02	1.82E-01
AMINE METABOLIC PROCESS	GO:0009308	41	-0.19	-1.45	1.06E-01	1.82E-01
SPINDLE ORGANIZATION	GO:0007051	114	-0.11	-1.45	1.23E-01	1.82E-01
ACTIVATION OF PROTEIN KINASE ACTIVITY	GO:0032147	68	-0.15	-1.45	9.07E-02	1.81E-01
MYELOID CELL ACTIVATION INVOLVED IN IMMUNE RESPONSE	GO:0002275	31	-0.22	-1.45	7.74E-02	1.80E-01
MULTICELLULAR ORGANISMAL REPRODUCTIVE PROCESS	GO:0048609	165	-0.10	-1.45	8.58E-02	1.80E-01
REGULATION OF PROTEIN LOCALIZATION TO PLASMA MEMBRANE	GO:1903076	68	-0.15	-1.45	8.27E-02	1.80E-01
CARBOXYLIC ACID TRANSMEMBRANE TRANSPORT	GO:1905039	62	-0.16	-1.45	1.03E-01	1.80E-01
POSITIVE REGULATION OF PRODUCTION OF MOLECULAR MEDIATOR OF IMMUNE RESPONSE	GO:0002702	76	-0.14	-1.45	8.78E-02	1.80E-01
CARDIAC CHAMBER DEVELOPMENT	GO:0003205	42	-0.19	-1.45	7.06E-02	1.80E-01
REGULATION OF CD4-POSITIVE, ALPHA-BETA T CELL ACTIVATION	GO:2000514	46	-0.18	-1.45	8.96E-02	1.80E-01
GERM CELL DEVELOPMENT	GO:0007281	53	-0.17	-1.45	9.07E-02	1.80E-01
REGULATION OF PHOSPHATIDYLINOSITOL 3-KINASE SIGNALING	GO:0014066	56	-0.17	-1.46	6.92E-02	1.79E-01
CYTOSOLIC CALCIUM ION TRANSPORT	GO:0060401	44	-0.19	-1.46	8.35E-02	1.78E-01
CELL CYCLE PHASE TRANSITION	GO:0044770	123	-0.11	-1.46	9.56E-02	1.77E-01
PROTEIN POLYMERIZATION	GO:0051258	65	-0.16	-1.46	8.25E-02	1.77E-01
CELLULAR RESPONSE TO MOLECULE OF BACTERIAL ORIGIN	GO:0071219	99	-0.13	-1.46	8.23E-02	1.77E-01
MEMBRANE LIPID METABOLIC PROCESS	GO:0006643	127	-0.11	-1.46	9.09E-02	1.77E-01
LIPID MODIFICATION	GO:0030258	108	-0.12	-1.46	8.50E-02	1.77E-01
EMBRYONIC MORPHOGENESIS	GO:0048598	133	-0.11	-1.46	9.85E-02	1.76E-01
ORGANIC ACID TRANSMEMBRANE TRANSPORT	GO:1903825	62	-0.16	-1.46	7.30E-02	1.75E-01
STEROL TRANSPORT	GO:0015918	36	-0.20	-1.46	8.65E-02	1.76E-01

REGULATION OF MRNA PROCESSING	GO:0050684	104	-0.12	-1.46	6.68E-02	1.74E-01
MESENCHYME DEVELOPMENT	GO:0060485	81	-0.14	-1.47	8.53E-02	1.74E-01
ORGANIC ANION TRANSPORT	GO:0015711	123	-0.11	-1.47	8.05E-02	1.74E-01
CELLULAR CALCIUM ION HOMEOSTASIS	GO:0006874	177	-0.09	-1.47	7.39E-02	1.73E-01
CALCIUM ION HOMEOSTASIS	GO:0055074	180	-0.09	-1.47	7.00E-02	1.73E-01
GAMETE GENERATION	GO:0007276	151	-0.10	-1.47	9.18E-02	1.73E-01
VISUAL PERCEPTION	GO:0007601	37	-0.20	-1.47	8.96E-02	1.74E-01
RESPONSE TO WOUNDING	GO:0009611	168	-0.10	-1.47	6.70E-02	1.74E-01
POSITIVE REGULATION OF BINDING	GO:0051099	100	-0.12	-1.47	8.47E-02	1.73E-01
POSITIVE REGULATION OF ENDOPEPTIDASE ACTIVITY	GO:0010950	124	-0.11	-1.47	8.14E-02	1.72E-01
REGULATION OF SIGNALING RECEPTOR ACTIVITY	GO:0010469	71	-0.15	-1.47	7.66E-02	1.72E-01
PROTEIN LOCALIZATION TO EXTRACELLULAR REGION	GO:0071692	53	-0.17	-1.47	7.17E-02	1.72E-01
NEGATIVE REGULATION OF AUTOPHAGY	GO:0010507	55	-0.17	-1.47	7.88E-02	1.72E-01
NEGATIVE REGULATION OF RNA CATABOLIC PROCESS	GO:1902369	47	-0.18	-1.47	8.73E-02	1.71E-01
NEGATIVE REGULATION OF EPITHELIAL CELL PROLIFERATION	GO:0050680	47	-0.19	-1.47	8.00E-02	1.71E-01
RESPONSE TO HYPOXIA	GO:0001666	99	-0.13	-1.48	8.06E-02	1.70E-01
WNT SIGNALING PATHWAY	GO:0016055	61	-0.16	-1.48	6.56E-02	1.69E-01
NEGATIVE REGULATION OF NEURON APOPTOTIC PROCESS	GO:0043524	33	-0.22	-1.48	7.71E-02	1.69E-01
POSITIVE REGULATION OF CANONICAL WNT SIGNALING PATHWAY	GO:0090263	61	-0.16	-1.48	7.47E-02	1.68E-01
REGULATION OF RESPONSE TO OXIDATIVE STRESS	GO:1902882	49	-0.18	-1.48	8.47E-02	1.67E-01
MRNA 3'-END PROCESSING	GO:0031124	40	-0.20	-1.48	7.56E-02	1.67E-01
EYE MORPHOGENESIS	GO:0048592	32	-0.22	-1.48	7.28E-02	1.66E-01
POSITIVE REGULATION OF RESPONSE TO CYTOKINE STIMULUS	GO:0060760	33	-0.22	-1.48	8.54E-02	1.66E-01
CHROMOSOME SEGREGATION	GO:0007059	170	-0.10	-1.48	7.20E-02	1.66E-01
CYTOSKELETON-DEPENDENT CYTOKINESIS	GO:0061640	66	-0.16	-1.48	7.32E-02	1.66E-01
CELL DIVISION	GO:0051301	103	-0.13	-1.48	7.85E-02	1.66E-01
CYTOKINESIS	GO:0000910	68	-0.15	-1.48	7.45E-02	1.66E-01
POSITIVE REGULATION OF ION TRANSPORT	GO:0043270	98	-0.13	-1.49	6.57E-02	1.66E-01
RIBONUCLEOSIDE BISPHOSPHATE BIOSYNTHETIC PROCESS	GO:0034030	43	-0.19	-1.49	6.80E-02	1.66E-01
POSITIVE REGULATION OF LYMPHOCYTE MEDIATED IMMUNITY	GO:0002708	67	-0.15	-1.49	6.30E-02	1.66E-01
ERAD PATHWAY	GO:0036503	82	-0.14	-1.49	9.26E-02	1.65E-01
SENSORY ORGAN DEVELOPMENT	GO:0007423	107	-0.12	-1.49	6.26E-02	1.65E-01
PROTEIN SECRETION	GO:0009306	49	-0.18	-1.49	6.13E-02	1.64E-01

WOUND HEALING	GO:0042060	141	-0.11	-1.49	7.72E-02	1.63E-01
NEGATIVE REGULATION OF NERVOUS SYSTEM DEVELOPMENT	GO:0051961	49	-0.18	-1.49	7.54E-02	1.63E-01
POSITIVE REGULATION OF PHOSPHATIDYLINOSITOL 3-KINASE SIGNALING	GO:0014068	39	-0.20	-1.49	7.52E-02	1.63E-01
REGULATION OF ION TRANSMEMBRANE TRANSPORTER ACTIVITY	GO:0032412	114	-0.12	-1.49	6.46E-02	1.63E-01
REGULATION OF CELLULAR PROTEIN CATABOLIC PROCESS	GO:1903362	191	-0.09	-1.50	7.06E-02	1.61E-01
POSITIVE REGULATION OF ADAPTIVE IMMUNE RESPONSE BASED ON SOMATIC RECOMBINATION OF IMMUNE RECEPTORS BUILT FROM IMMUNOGLOBULIN SUPERFAMILY DOMAINS	GO:0002824	62	-0.16	-1.50	7.44E-02	1.61E-01
MITOTIC SISTER CHROMATID SEGREGATION	GO:0000070	88	-0.14	-1.50	5.97E-02	1.61E-01
RESPONSE TO INTERLEUKIN-1	GO:0070555	60	-0.17	-1.50	8.51E-02	1.61E-01
NUCLEOSIDE BISPHOSPHATE BIOSYNTHETIC PROCESS	GO:0033866	43	-0.19	-1.50	9.04E-02	1.61E-01
POSITIVE REGULATION OF ADAPTIVE IMMUNE RESPONSE	GO:0002821	68	-0.16	-1.50	8.50E-02	1.61E-01
NEGATIVE REGULATION OF TRANSMEMBRANE TRANSPORT	GO:0034763	59	-0.16	-1.50	6.49E-02	1.61E-01
ACYL-COA BIOSYNTHETIC PROCESS	GO:0071616	30	-0.23	-1.50	7.52E-02	1.61E-01
NEGATIVE REGULATION OF CELL DEVELOPMENT	GO:0010721	66	-0.15	-1.50	7.44E-02	1.61E-01
DNA-DEPENDENT DNA REPLICATION MAINTENANCE OF FIDELITY	GO:0045005	40	-0.20	-1.50	7.17E-02	1.60E-01
MRNA TRANSPORT	GO:0051028	50	-0.18	-1.50	7.54E-02	1.59E-01
REGULATION OF RAS PROTEIN SIGNAL TRANSDUCTION	GO:0046578	102	-0.13	-1.50	5.77E-02	1.59E-01
TISSUE HOMEOSTASIS	GO:0001894	81	-0.14	-1.50	7.07E-02	1.60E-01
NEGATIVE REGULATION OF ION TRANSMEMBRANE TRANSPORT	GO:0034766	45	-0.19	-1.50	6.58E-02	1.60E-01
THIOESTER BIOSYNTHETIC PROCESS	GO:0035384	30	-0.23	-1.50	5.08E-02	1.60E-01
NEGATIVE REGULATION OF PEPTIDASE ACTIVITY	GO:0010466	78	-0.14	-1.50	8.08E-02	1.59E-01
REGULATION OF NITRIC OXIDE METABOLIC PROCESS	GO:0080164	34	-0.22	-1.50	6.88E-02	1.59E-01
TRANSITION METAL ION TRANSPORT	GO:0000041	51	-0.18	-1.50	5.79E-02	1.60E-01
AMINO SUGAR METABOLIC PROCESS	GO:0006040	30	-0.23	-1.51	6.67E-02	1.57E-01
PALLIUM DEVELOPMENT	GO:0021543	35	-0.22	-1.51	7.69E-02	1.56E-01
REGULATION OF LEUKOCYTE CELL-CELL ADHESION	GO:1903037	200	-0.09	-1.51	7.59E-02	1.56E-01
REGULATION OF LYMPHOCYTE MIGRATION	GO:2000401	38	-0.21	-1.52	7.54E-02	1.53E-01
NEGATIVE REGULATION OF MITOTIC CELL CYCLE	GO:0045930	136	-0.11	-1.52	6.35E-02	1.53E-01
MONONUCLEAR CELL MIGRATION	GO:0071674	54	-0.17	-1.52	8.63E-02	1.53E-01
NEGATIVE REGULATION OF CELL CYCLE PHASE TRANSITION	GO:1901988	143	-0.11	-1.52	6.65E-02	1.53E-01
MICROTUBULE POLYMERIZATION OR DEPOLYMERIZATION	GO:0031109	39	-0.20	-1.52	7.10E-02	1.52E-01
POSITIVE REGULATION OF MAP KINASE ACTIVITY	GO:0043406	62	-0.17	-1.52	7.19E-02	1.50E-01

MITOTIC CYTOKINESIS	GO:0000281	51	-0.18	-1.52	5.97E-02	1.50E-01
REGULATION OF SYSTEM PROCESS	GO:0044057	169	-0.10	-1.52	6.10E-02	1.50E-01
POSITIVE REGULATION OF CHROMOSOME ORGANIZATION	GO:2001252	71	-0.15	-1.52	4.75E-02	1.49E-01
MRNA STABILIZATION	GO:0048255	30	-0.23	-1.53	6.48E-02	1.47E-01
PURINE NUCLEOSIDE BISPHOSPHATE BIOSYNTHETIC PROCESS	GO:0034033	43	-0.19	-1.53	6.92E-02	1.47E-01
CELL-CELL SIGNALING BY WNT	GO:0198738	63	-0.17	-1.53	6.27E-02	1.47E-01
CELL-SUBSTRATE ADHESION	GO:0031589	83	-0.15	-1.53	6.80E-02	1.48E-01
REGULATION OF NEURON APOPTOTIC PROCESS	GO:0043523	57	-0.17	-1.53	6.57E-02	1.48E-01
CELLULAR RESPONSE TO DECREASED OXYGEN LEVELS	GO:0036294	60	-0.17	-1.53	5.91E-02	1.47E-01
PEPTIDYL-LYSINE MODIFICATION	GO:0018205	195	-0.10	-1.53	5.48E-02	1.47E-01
CERAMIDE BIOSYNTHETIC PROCESS	GO:0046513	33	-0.22	-1.53	6.45E-02	1.47E-01
HOMEOSTASIS OF NUMBER OF CELLS	GO:0048872	81	-0.14	-1.53	5.03E-02	1.47E-01
DENDRITE DEVELOPMENT	GO:0016358	37	-0.21	-1.53	5.25E-02	1.48E-01
REGULATION OF STRESS-ACTIVATED MAPK CASCADE	GO:0032872	124	-0.12	-1.53	7.50E-02	1.45E-01
NEGATIVE REGULATION OF CELL CYCLE PROCESS	GO:0010948	171	-0.10	-1.54	7.94E-02	1.45E-01
ESTABLISHMENT OF RNA LOCALIZATION	GO:0051236	83	-0.14	-1.54	4.98E-02	1.44E-01
MEIOTIC CELL CYCLE	GO:0051321	78	-0.15	-1.54	5.89E-02	1.44E-01
CHOLESTEROL TRANSPORT	GO:0030301	35	-0.22	-1.54	6.76E-02	1.43E-01
NEGATIVE REGULATION OF CELL PROJECTION ORGANIZATION	GO:0031345	77	-0.15	-1.54	6.45E-02	1.43E-01
CELLULAR DIVALENT INORGANIC CATION HOMEOSTASIS	GO:0072503	195	-0.09	-1.54	5.61E-02	1.42E-01
REGULATION OF EPIDERMAL GROWTH FACTOR RECEPTOR SIGNALING PATHWAY	GO:0042058	49	-0.19	-1.54	5.10E-02	1.42E-01
LEUKOCYTE DIFFERENTIATION	GO:0002521	167	-0.10	-1.54	4.92E-02	1.42E-01
REGULATION OF JUN KINASE ACTIVITY	GO:0043506	32	-0.23	-1.54	6.07E-02	1.42E-01
ORGANELLE FUSION	GO:0048284	83	-0.14	-1.54	5.59E-02	1.42E-01
CELLULAR MONOVALENT INORGANIC CATION HOMEOSTASIS	GO:0030004	58	-0.17	-1.54	6.34E-02	1.42E-01
NEGATIVE REGULATION OF MITOTIC CELL CYCLE PHASE TRANSITION	GO:1901991	108	-0.13	-1.54	5.10E-02	1.42E-01
EMBRYO DEVELOPMENT ENDING IN BIRTH OR EGG HATCHING	GO:0009792	94	-0.14	-1.55	6.15E-02	1.41E-01
REGULATION OF NITRIC OXIDE BIOSYNTHETIC PROCESS	GO:0045428	32	-0.22	-1.55	7.26E-02	1.41E-01
STRESS-ACTIVATED MAPK CASCADE	GO:0051403	38	-0.22	-1.55	6.41E-02	1.40E-01
NUCLEAR-TRANSCRIBED MRNA CATABOLIC PROCESS	GO:0000956	89	-0.14	-1.55	6.18E-02	1.39E-01
NEUTROPHIL CHEMOTAXIS	GO:0030593	36	-0.22	-1.55	6.00E-02	1.39E-01
REGULATION OF DNA REPAIR	GO:0006282	103	-0.13	-1.55	5.28E-02	1.39E-01
NEGATIVE REGULATION OF INFLAMMATORY RESPONSE	GO:0050728	68	-0.16	-1.55	5.20E-02	1.39E-01

REGULATION OF SYNAPSE STRUCTURE OR ACTIVITY	GO:0050803	71	-0.16	-1.55	4.98E-02	1.39E-01
MAINTENANCE OF PROTEIN LOCALIZATION IN ORGANELLE	GO:0072595	33	-0.23	-1.55	5.14E-02	1.38E-01
NUCLEUS ORGANIZATION	GO:0006997	88	-0.14	-1.55	5.13E-02	1.39E-01
CELLULAR DEFENSE RESPONSE	GO:0006968	35	-0.22	-1.56	4.89E-02	1.38E-01
MULTICELLULAR ORGANISM REPRODUCTION	GO:0032504	170	-0.10	-1.56	4.65E-02	1.35E-01
RESPONSE TO STEROID HORMONE	GO:0048545	64	-0.17	-1.56	3.76E-02	1.33E-01
ANATOMICAL STRUCTURE HOMEOSTASIS	GO:0060249	99	-0.13	-1.57	5.78E-02	1.32E-01
NEGATIVE REGULATION OF TRANSPORTER ACTIVITY	GO:0032410	40	-0.21	-1.57	5.79E-02	1.30E-01
POSITIVE REGULATION OF TRANSPORTER ACTIVITY	GO:0032411	53	-0.19	-1.57	5.68E-02	1.30E-01
REGULATION OF CIRCADIAN RHYTHM	GO:0042752	71	-0.16	-1.57	5.08E-02	1.30E-01
GLYCOSYLATION	GO:0070085	144	-0.11	-1.57	4.35E-02	1.30E-01
REGULATION OF LEUKOCYTE DEGRANULATION	GO:0043300	30	-0.24	-1.57	6.08E-02	1.30E-01
REGULATION OF MYELOID CELL DIFFERENTIATION	GO:0045637	107	-0.13	-1.57	3.99E-02	1.29E-01
RESPONSE TO OXYGEN LEVELS	GO:0070482	114	-0.12	-1.58	5.24E-02	1.28E-01
TRANSPORT ACROSS BLOOD-BRAIN BARRIER	GO:0150104	52	-0.19	-1.58	5.22E-02	1.27E-01
REGULATION OF INSULIN RECEPTOR SIGNALING PATHWAY	GO:0046626	34	-0.23	-1.58	5.30E-02	1.26E-01
RESPONSE TO TUMOR NECROSIS FACTOR	GO:0034612	96	-0.14	-1.58	6.53E-02	1.25E-01
ENDOPLASMIC RETICULUM UNFOLDED PROTEIN RESPONSE	GO:0030968	33	-0.23	-1.58	5.16E-02	1.24E-01
REGULATION OF EMBRYONIC DEVELOPMENT	GO:0045995	30	-0.24	-1.58	4.50E-02	1.25E-01
POSITIVE REGULATION OF ORGANELLE ASSEMBLY	GO:1902117	49	-0.19	-1.58	4.93E-02	1.25E-01
SPHINGOLIPID BIOSYNTHETIC PROCESS	GO:0030148	58	-0.18	-1.58	4.17E-02	1.25E-01
PROTEIN MONOUBIQUITINATION	GO:0006513	52	-0.19	-1.58	5.31E-02	1.24E-01
REGULATION OF TUBE DIAMETER	GO:0035296	32	-0.24	-1.58	4.09E-02	1.24E-01
REGULATION OF CANONICAL WNT SIGNALING PATHWAY	GO:0060828	135	-0.12	-1.59	6.20E-02	1.24E-01
AUTOPHAGY	GO:0006914	165	-0.11	-1.59	4.44E-02	1.24E-01
REGULATION OF G1/S TRANSITION OF MITOTIC CELL CYCLE	GO:2000045	80	-0.15	-1.59	3.93E-02	1.22E-01
PROCESS UTILIZING AUTOPHAGIC MECHANISM	GO:0061919	165	-0.11	-1.59	4.26E-02	1.21E-01
RESPONSE TO DECREASED OXYGEN LEVELS	GO:0036293	102	-0.14	-1.60	5.92E-02	1.20E-01
REGULATION OF CHROMOSOME ORGANIZATION	GO:0033044	160	-0.11	-1.60	5.47E-02	1.19E-01
B CELL ACTIVATION	GO:0042113	84	-0.15	-1.60	5.71E-02	1.19E-01
CIRCADIAN REGULATION OF GENE EXPRESSION	GO:0032922	52	-0.19	-1.60	5.59E-02	1.19E-01
EYE DEVELOPMENT	GO:0001654	79	-0.15	-1.60	4.10E-02	1.19E-01
VESICLE CYTOSKELETAL TRAFFICKING	GO:0099518	50	-0.19	-1.60	4.33E-02	1.18E-01

REGULATION OF TUBE SIZE	GO:0035150	32	-0.24	-1.60	5.77E-02	1.18E-01
REGULATION OF PROTEOLYSIS INVOLVED IN CELLULAR PROTEIN CATABOLIC PROCESS	GO:1903050	167	-0.11	-1.60	3.98E-02	1.18E-01
AXONAL TRANSPORT	GO:0098930	41	-0.21	-1.60	4.62E-02	1.17E-01
NEGATIVE REGULATION OF ION TRANSMEMBRANE TRANSPORTER ACTIVITY	GO:0032413	34	-0.23	-1.61	5.27E-02	1.15E-01
ESTABLISHMENT OF SPINDLE LOCALIZATION	GO:0051293	30	-0.25	-1.61	3.68E-02	1.16E-01
MAINTENANCE OF PROTEIN LOCATION	GO:0045185	68	-0.17	-1.61	4.14E-02	1.15E-01
REGULATION OF SYNAPSE ORGANIZATION	GO:0050807	69	-0.16	-1.61	4.22E-02	1.15E-01
NEGATIVE REGULATION OF CYTOSKELETON ORGANIZATION	GO:0051494	85	-0.15	-1.61	5.05E-02	1.14E-01
EXOCYTIC PROCESS	GO:0140029	37	-0.23	-1.61	3.15E-02	1.13E-01
REGULATION OF ENDOPEPTIDASE ACTIVITY	GO:0052548	199	-0.10	-1.62	4.66E-02	1.11E-01
NEUTROPHIL MIGRATION	GO:1990266	37	-0.23	-1.62	4.61E-02	1.11E-01
EXPORT FROM CELL	GO:0140352	185	-0.10	-1.62	4.23E-02	1.11E-01
POSITIVE REGULATION OF COLD-INDUCED THERMOGENESIS	GO:0120162	62	-0.17	-1.62	4.41E-02	1.11E-01
ORGANIC HYDROXY COMPOUND TRANSPORT	GO:0015850	59	-0.17	-1.62	5.05E-02	1.11E-01
RESPONSE TO OSMOTIC STRESS	GO:0006970	30	-0.25	-1.62	3.81E-02	1.11E-01
REGULATION OF MICROTUBULE-BASED PROCESS	GO:0032886	144	-0.12	-1.62	2.62E-02	1.11E-01
VASCULAR TRANSPORT	GO:0010232	52	-0.19	-1.62	3.75E-02	1.11E-01
REGULATION OF DENDRITE DEVELOPMENT	GO:0050773	36	-0.23	-1.62	3.77E-02	1.10E-01
POSITIVE REGULATION OF EPITHELIAL CELL MIGRATION	GO:0010634	98	-0.14	-1.62	3.55E-02	1.10E-01
MUCOPOLYSACCHARIDE METABOLIC PROCESS	GO:1903510	39	-0.22	-1.62	4.42E-02	1.09E-01
NUCLEOBASE-CONTAINING COMPOUND TRANSPORT	GO:0015931	111	-0.13	-1.63	2.70E-02	1.09E-01
POSITIVE REGULATION OF NERVOUS SYSTEM DEVELOPMENT	GO:0051962	93	-0.15	-1.63	3.67E-02	1.09E-01
REGULATION OF INTERFERON-BETA PRODUCTION	GO:0032648	48	-0.20	-1.63	4.99E-02	1.08E-01
SPINDLE LOCALIZATION	GO:0051653	30	-0.25	-1.63	4.41E-02	1.08E-01
REGULATION OF CHROMATIN ORGANIZATION	GO:1902275	30	-0.25	-1.63	3.86E-02	1.07E-01
POSITIVE REGULATION OF FAT CELL DIFFERENTIATION	GO:0045600	33	-0.24	-1.63	4.41E-02	1.06E-01
POTASSIUM ION TRANSMEMBRANE TRANSPORT	GO:0071805	45	-0.20	-1.63	3.37E-02	1.06E-01
MYELOID LEUKOCYTE MIGRATION	GO:0097529	54	-0.19	-1.63	2.64E-02	1.06E-01
REGULATION OF EPITHELIAL CELL PROLIFERATION	GO:0050678	128	-0.12	-1.64	2.39E-02	1.05E-01
BLOOD VESSEL DIAMETER MAINTENANCE	GO:0097746	32	-0.24	-1.64	4.40E-02	1.04E-01
DEVELOPMENTAL MATURATION	GO:0021700	65	-0.17	-1.64	4.91E-02	1.03E-01
EPIDERMIS DEVELOPMENT	GO:0008544	59	-0.18	-1.64	2.27E-02	1.03E-01
ANTERIOR/POSTERIOR PATTERN SPECIFICATION	GO:0009952	34	-0.23	-1.64	4.73E-02	1.03E-01

ENDOPLASMIC RETICULUM ORGANIZATION	GO:0007029	65	-0.18	-1.64	4.61E-02	1.03E-01
REGULATION OF MICROTUBULE CYTOSKELETON ORGANIZATION	GO:0070507	99	-0.14	-1.64	3.87E-02	1.03E-01
CELLULAR RESPONSE TO HYDROGEN PEROXIDE	GO:0070301	34	-0.24	-1.64	3.07E-02	1.03E-01
POSITIVE REGULATION OF MACROAUTOPHAGY	GO:0016239	45	-0.21	-1.65	3.75E-02	1.02E-01
MULTI-ORGANISM REPRODUCTIVE PROCESS	GO:0044703	197	-0.10	-1.65	3.34E-02	1.02E-01
REGULATION OF INTERLEUKIN-10 PRODUCTION	GO:0032653	38	-0.22	-1.65	3.91E-02	1.02E-01
PROTEIN LOCALIZATION TO CYTOSKELETON	GO:0044380	30	-0.25	-1.65	5.19E-02	1.02E-01
TRANSCRIPTION BY RNA POLYMERASE II	GO:0006366	160	-0.11	-1.65	3.30E-02	1.02E-01
DEMETHYLATION	GO:0070988	44	-0.21	-1.65	3.41E-02	1.01E-01
POSITIVE REGULATION OF PROTEASOMAL PROTEIN CATABOLIC PROCESS	GO:1901800	85	-0.15	-1.65	3.02E-02	1.00E-01
CELL AGING	GO:0007569	48	-0.20	-1.65	3.22E-02	1.00E-01
MICROTUBULE POLYMERIZATION	GO:0046785	31	-0.25	-1.65	4.41E-02	9.97E-02
SECRETION BY CELL	GO:0032940	161	-0.11	-1.65	2.36E-02	9.94E-02
POSITIVE REGULATION OF NEURON DEATH	GO:1901216	39	-0.22	-1.65	2.12E-02	9.95E-02
REGULATION OF GLUCOSE TRANSMEMBRANE TRANSPORT	GO:0010827	32	-0.25	-1.66	3.11E-02	9.95E-02
GRANULOCYTE MIGRATION	GO:0097530	40	-0.22	-1.66	3.33E-02	9.91E-02
POSITIVE REGULATION OF AUTOPHAGY	GO:0010508	95	-0.14	-1.66	3.68E-02	9.91E-02
REGULATION OF HISTONE METHYLATION	GO:0031060	38	-0.22	-1.66	3.36E-02	9.92E-02
SPHINGOLIPID METABOLIC PROCESS	GO:0006665	90	-0.15	-1.66	3.50E-02	9.87E-02
AXONOGENESIS	GO:0007409	130	-0.13	-1.66	2.92E-02	9.78E-02
MAPK CASCADE	GO:0000165	90	-0.15	-1.66	2.40E-02	9.78E-02
REGULATION OF STRESS-ACTIVATED PROTEIN KINASE SIGNALING CASCADE	GO:0070302	126	-0.13	-1.66	2.24E-02	9.77E-02
REGULATION OF UBIQUITIN-DEPENDENT PROTEIN CATABOLIC PROCESS	GO:2000058	129	-0.13	-1.66	3.72E-02	9.75E-02
REGULATION OF AXONOGENESIS	GO:0050770	61	-0.18	-1.66	3.02E-02	9.75E-02
PROTEIN GLYCOSYLATION	GO:0006486	131	-0.13	-1.66	3.08E-02	9.77E-02
CALCIUM ION TRANSPORT INTO CYTOSOL	GO:0060402	32	-0.25	-1.67	2.30E-02	9.71E-02
LEUKOCYTE ACTIVATION INVOLVED IN IMMUNE RESPONSE	GO:0002366	80	-0.16	-1.67	3.54E-02	9.69E-02
POSITIVE REGULATION OF CELLULAR PROTEIN CATABOLIC PROCESS	GO:1903364	113	-0.13	-1.67	4.76E-02	9.62E-02
MACROMOLECULE GLYCOSYLATION	GO:0043413	131	-0.13	-1.67	2.23E-02	9.54E-02
NEGATIVE REGULATION OF ENDOPEPTIDASE ACTIVITY	GO:0010951	72	-0.17	-1.67	2.69E-02	9.53E-02
POSITIVE REGULATION OF LEUKOCYTE MEDIATED IMMUNITY	GO:0002705	82	-0.15	-1.67	2.71E-02	9.48E-02
ACTIVATION OF GTPASE ACTIVITY	GO:0090630	73	-0.17	-1.67	2.56E-02	9.50E-02
POSITIVE REGULATION OF NEUROGENESIS	GO:0050769	80	-0.16	-1.67	2.62E-02	9.52E-02

EPITHELIAL CELL DIFFERENTIATION	GO:0030855	166	-0.11	-1.67	3.10E-02	9.46E-02
CELL SURFACE RECEPTOR SIGNALING PATHWAY INVOLVED IN CELL-CELL SIGNALING	GO:1905114	71	-0.17	-1.67	2.40E-02	9.45E-02
MONONUCLEAR CELL DIFFERENTIATION	GO:1903131	129	-0.12	-1.68	3.70E-02	9.38E-02
TELENCEPHALON DEVELOPMENT	GO:0021537	53	-0.20	-1.68	2.85E-02	9.32E-02
REGULATION OF LEUKOCYTE CHEMOTAXIS	GO:0002688	71	-0.17	-1.68	2.94E-02	9.31E-02
B CELL DIFFERENTIATION	GO:0030183	50	-0.20	-1.68	2.14E-02	9.29E-02
MODULATION BY SYMBIONT OF ENTRY INTO HOST	GO:0052372	32	-0.25	-1.68	3.19E-02	9.24E-02
NEGATIVE REGULATION OF CELLULAR PROTEIN LOCALIZATION	GO:1903828	75	-0.17	-1.68	3.00E-02	9.26E-02
REGULATION OF NATURAL KILLER CELL MEDIATED IMMUNITY	GO:0002715	30	-0.26	-1.68	3.07E-02	9.25E-02
REGULATION OF VASCULATURE DEVELOPMENT	GO:1901342	145	-0.12	-1.68	2.29E-02	9.19E-02
GRANULOCYTE CHEMOTAXIS	GO:0071621	38	-0.23	-1.68	2.73E-02	9.20E-02
CILIUM OR FLAGELLUM-DEPENDENT CELL MOTILITY	GO:0001539	31	-0.25	-1.68	3.78E-02	9.19E-02
NEGATIVE REGULATION OF CELL-SUBSTRATE ADHESION	GO:0010812	35	-0.23	-1.69	2.69E-02	9.17E-02
NEGATIVE REGULATION OF COLD-INDUCED THERMOGENESIS	GO:0120163	34	-0.24	-1.69	2.22E-02	9.00E-02
PROTEIN COMPLEX OLIGOMERIZATION	GO:0051259	122	-0.13	-1.69	2.62E-02	9.00E-02
POSITIVE REGULATION OF PHOSPHOLIPASE ACTIVITY	GO:0010518	31	-0.25	-1.69	3.22E-02	8.96E-02
PROTEIN HOMOOLIGOMERIZATION	GO:0051260	88	-0.15	-1.69	2.48E-02	8.97E-02
MITOTIC SPINDLE ORGANIZATION	GO:0007052	71	-0.17	-1.69	3.25E-02	8.98E-02
RESPONSE TO METAL ION	GO:0010038	129	-0.13	-1.69	2.88E-02	9.00E-02
POSITIVE REGULATION OF LIPASE ACTIVITY	GO:0060193	36	-0.24	-1.69	2.70E-02	9.00E-02
REGULATION OF MITOTIC CELL CYCLE PHASE TRANSITION	GO:1901990	191	-0.11	-1.69	2.55E-02	8.93E-02
NEGATIVE REGULATION OF SECRETION	GO:0051048	58	-0.19	-1.70	2.85E-02	8.85E-02
NEGATIVE REGULATION OF MRNA CATABOLIC PROCESS	GO:1902373	36	-0.24	-1.70	1.80E-02	8.85E-02
POSITIVE REGULATION OF RESPONSE TO DNA DAMAGE STIMULUS	GO:2001022	83	-0.16	-1.70	3.97E-02	8.76E-02
REGULATION OF ENDOCYTOSIS	GO:0030100	120	-0.13	-1.70	2.53E-02	8.76E-02
REGULATION OF CHROMOSOME SEGREGATION	GO:0051983	69	-0.17	-1.70	3.04E-02	8.66E-02
CELLULAR SENESCENCE	GO:0090398	36	-0.24	-1.70	2.79E-02	8.64E-02
CILIUM-DEPENDENT CELL MOTILITY	GO:0060285	31	-0.25	-1.71	3.00E-02	8.53E-02
REGULATION OF MONONUCLEAR CELL MIGRATION	GO:0071675	71	-0.18	-1.71	2.70E-02	8.47E-02
ENDOTHELIAL CELL DIFFERENTIATION	GO:0045446	40	-0.23	-1.71	3.07E-02	8.48E-02
POSITIVE REGULATION OF NEURON PROJECTION DEVELOPMENT	GO:0010976	51	-0.20	-1.71	2.67E-02	8.38E-02
ENDOSOME TO LYSOSOME TRANSPORT	GO:0008333	46	-0.22	-1.71	2.01E-02	8.37E-02
PEPTIDYL-TYROSINE PHOSPHORYLATION	GO:0018108	46	-0.22	-1.71	2.78E-02	8.37E-02

REGULATION OF ANGIOGENESIS	GO:0045765	145	-0.12	-1.71	2.74E-02	8.36E-02
HISTONE LYSINE METHYLATION	GO:0034968	46	-0.22	-1.71	1.41E-02	8.35E-02
MICROTUBULE-BASED TRANSPORT	GO:0099111	107	-0.14	-1.72	2.59E-02	8.32E-02
ACTIN CYTOSKELETON REORGANIZATION	GO:0031532	48	-0.21	-1.72	3.43E-02	8.31E-02
UBIQUITIN-DEPENDENT ERAD PATHWAY	GO:0030433	69	-0.17	-1.72	3.28E-02	8.31E-02
IMPORT INTO NUCLEUS	GO:0051170	68	-0.18	-1.72	1.58E-02	8.15E-02
POSITIVE REGULATION OF PROTEIN SERINE/THREONINE KINASE ACTIVITY	GO:0071902	107	-0.14	-1.72	2.26E-02	8.17E-02
POTASSIUM ION TRANSPORT	GO:0006813	50	-0.21	-1.72	1.59E-02	8.16E-02
POSITIVE REGULATION OF PROTEOLYSIS INVOLVED IN CELLULAR PROTEIN CATABOLIC PROCESS	GO:1903052	98	-0.14	-1.72	3.48E-02	8.13E-02
LIPID LOCALIZATION	GO:0010876	144	-0.12	-1.72	1.52E-02	8.13E-02
SENSORY SYSTEM DEVELOPMENT	GO:0048880	82	-0.16	-1.72	1.02E-02	8.12E-02
VISUAL SYSTEM DEVELOPMENT	GO:0150063	82	-0.16	-1.73	2.40E-02	8.08E-02
NEGATIVE REGULATION OF NF-KAPPAB TRANSCRIPTION FACTOR ACTIVITY	GO:0032088	67	-0.18	-1.73	2.24E-02	7.94E-02
CELLULAR RESPONSE TO CHEMICAL STRESS	GO:0062197	149	-0.12	-1.73	2.07E-02	7.93E-02
EXOCYTOSIS	GO:0006887	110	-0.14	-1.73	2.23E-02	7.95E-02
POSITIVE REGULATION OF PROTEASOMAL UBIQUITIN-DEPENDENT PROTEIN CATABOLIC PROCESS	GO:0032436	70	-0.18	-1.73	2.83E-02	7.90E-02
RESPONSE TO MOLECULE OF BACTERIAL ORIGIN	GO:0002237	135	-0.13	-1.73	3.15E-02	7.83E-02
NEGATIVE REGULATION OF RESPONSE TO EXTERNAL STIMULUS	GO:0032102	200	-0.10	-1.74	1.99E-02	7.81E-02
REGULATION OF PROTEIN LOCALIZATION TO CELL PERIPHERY	GO:1904375	80	-0.17	-1.74	1.17E-02	7.82E-02
CELLULAR RESPONSE TO CHEMOKINE	GO:1990869	32	-0.26	-1.74	2.34E-02	7.80E-02
PEPTIDYL-TYROSINE MODIFICATION	GO:0018212	49	-0.21	-1.74	1.62E-02	7.67E-02
POSITIVE REGULATION OF UBIQUITIN-DEPENDENT PROTEIN CATABOLIC PROCESS	GO:2000060	81	-0.16	-1.74	2.71E-02	7.68E-02
SEXUAL REPRODUCTION	GO:0019953	177	-0.11	-1.74	2.39E-02	7.64E-02
MAINTENANCE OF PROTEIN LOCATION IN CELL	GO:0032507	47	-0.22	-1.75	2.45E-02	7.46E-02
CELLULAR RESPONSE TO BIOTIC STIMULUS	GO:0071216	115	-0.14	-1.75	2.93E-02	7.30E-02
HISTONE UBIQUITINATION	GO:0016574	35	-0.25	-1.75	3.47E-02	7.31E-02
AXON DEVELOPMENT	GO:0061564	143	-0.13	-1.75	2.34E-02	7.33E-02
SECRETION	GO:0046903	178	-0.11	-1.75	1.96E-02	7.26E-02
POSITIVE REGULATION OF INTERLEUKIN-8 PRODUCTION	GO:0032757	48	-0.22	-1.76	3.20E-02	7.23E-02
NUCLEAR-TRANSCRIBED MRNA CATABOLIC PROCESS, DEADENYLATION-DEPENDENT DECAY	GO:0000288	34	-0.25	-1.76	1.47E-02	7.18E-02

CENTROSOME DUPLICATION	GO:0051298	31	-0.26	-1.76	2.61E-02	7.18E-02
REGULATION OF CELL DIVISION	GO:0051302	80	-0.16	-1.76	2.27E-02	7.04E-02
POSITIVE REGULATION OF EPITHELIAL TO MESENCHYMAL TRANSITION	GO:0010718	30	-0.27	-1.76	2.24E-02	6.95E-02
NEGATIVE REGULATION OF RESPONSE TO CYTOKINE STIMULUS	GO:0060761	42	-0.23	-1.77	1.62E-02	6.94E-02
ENDOSOME ORGANIZATION	GO:0007032	65	-0.18	-1.77	2.42E-02	6.94E-02
NUCLEAR TRANSPORT	GO:0051169	154	-0.12	-1.77	1.70E-02	6.85E-02
REGULATION OF DEVELOPMENTAL GROWTH	GO:0048638	92	-0.16	-1.77	2.32E-02	6.85E-02
REGULATION OF ERYTHROCYTE DIFFERENTIATION	GO:0045646	35	-0.26	-1.77	1.60E-02	6.78E-02
IMPORT ACROSS PLASMA MEMBRANE	GO:0098739	63	-0.19	-1.77	2.11E-02	6.77E-02
POSITIVE REGULATION OF PHAGOCYTOSIS	GO:0050766	43	-0.22	-1.77	1.37E-02	6.75E-02
CIRCADIAN RHYTHM	GO:0007623	72	-0.18	-1.78	9.67E-03	6.63E-02
REGULATION OF ERK1 AND ERK2 CASCADE	GO:0070372	136	-0.13	-1.78	1.18E-02	6.51E-02
NEGATIVE REGULATION OF ION TRANSPORT	GO:0043271	56	-0.21	-1.78	9.96E-03	6.51E-02
PROTEIN IMPORT INTO NUCLEUS	GO:0006606	65	-0.18	-1.78	1.18E-02	6.52E-02
NEGATIVE REGULATION OF TRANSLATION	GO:0017148	129	-0.13	-1.78	1.84E-02	6.52E-02
VESICLE TARGETING	GO:0006903	31	-0.27	-1.78	1.19E-02	6.52E-02
RESPONSE TO CHEMOKINE	GO:1990868	32	-0.26	-1.78	8.02E-03	6.49E-02
NUCLEOCYTOPLASMIC TRANSPORT	GO:0006913	154	-0.12	-1.79	1.65E-02	6.50E-02
REGULATION OF RNA STABILITY	GO:0043487	129	-0.13	-1.79	1.43E-02	6.51E-02
REGULATION OF CELLULAR RESPONSE TO INSULIN STIMULUS	GO:1900076	32	-0.26	-1.79	1.74E-02	6.48E-02
REGULATION OF PROTEASOMAL PROTEIN CATABOLIC PROCESS	GO:0061136	140	-0.13	-1.79	1.37E-02	6.41E-02
STRESS-ACTIVATED PROTEIN KINASE SIGNALING CASCADE	GO:0031098	40	-0.24	-1.79	1.87E-02	6.42E-02
VESICLE BUDDING FROM MEMBRANE	GO:0006900	48	-0.22	-1.79	1.82E-02	6.44E-02
CALCIUM-MEDIATED SIGNALING	GO:0019722	83	-0.17	-1.79	1.93E-02	6.44E-02
NEGATIVE REGULATION OF RESPONSE TO BIOTIC STIMULUS	GO:0002832	78	-0.17	-1.79	1.15E-02	6.43E-02
POSITIVE REGULATION OF CYTOSOLIC CALCIUM ION CONCENTRATION	GO:0007204	114	-0.15	-1.79	1.39E-02	6.44E-02
POSITIVE REGULATION OF DNA-BINDING TRANSCRIPTION FACTOR ACTIVITY	GO:0051091	176	-0.12	-1.79	1.84E-02	6.46E-02
REGULATION OF VACUOLE ORGANIZATION	GO:0044088	40	-0.24	-1.79	1.39E-02	6.46E-02
REGULATION OF REGULATED SECRETORY PATHWAY	GO:1903305	60	-0.20	-1.79	5.80E-03	6.42E-02
REGULATION OF TYPE I INTERFERON PRODUCTION	GO:0032479	78	-0.17	-1.79	1.41E-02	6.42E-02
AXO-DENDRITIC TRANSPORT	GO:0008088	44	-0.23	-1.79	1.56E-02	6.42E-02
REGULATION OF WNT SIGNALING PATHWAY	GO:0030111	173	-0.12	-1.79	1.06E-02	6.44E-02
POSITIVE REGULATION OF NF-KAPPAB TRANSCRIPTION FACTOR ACTIVITY	GO:0051092	119	-0.14	-1.80	1.22E-02	6.42E-02

REGULATION OF GENE SILENCING	GO:0060968	45	-0.23	-1.80	2.20E-02	6.39E-02
ANATOMICAL STRUCTURE MATURATION	GO:0071695	47	-0.22	-1.80	7.53E-03	6.39E-02
CELL MORPHOGENESIS INVOLVED IN NEURON DIFFERENTIATION	GO:0048667	154	-0.13	-1.80	4.11E-03	6.31E-02
MULTICELLULAR ORGANISMAL HOMEOSTASIS	GO:0048871	119	-0.14	-1.80	1.25E-02	6.19E-02
NEGATIVE REGULATION OF LEUKOCYTE PROLIFERATION	GO:0070664	44	-0.23	-1.80	1.19E-02	6.19E-02
SISTER CHROMATID SEGREGATION	GO:0000819	109	-0.15	-1.81	2.06E-02	6.06E-02
REGULATION OF T CELL MEDIATED IMMUNITY	GO:0002709	62	-0.19	-1.81	1.85E-02	5.99E-02
GASTRULATION	GO:0007369	44	-0.23	-1.81	1.35E-02	5.94E-02
POSITIVE REGULATION OF WNT SIGNALING PATHWAY	GO:0030177	76	-0.18	-1.82	1.21E-02	5.85E-02
CARDIAC MUSCLE TISSUE DEVELOPMENT	GO:0048738	45	-0.23	-1.82	1.77E-02	5.74E-02
POSITIVE REGULATION OF LIPID BIOSYNTHETIC PROCESS	GO:0046889	42	-0.24	-1.82	1.82E-02	5.68E-02
REGULATION OF MRNA CATABOLIC PROCESS	GO:0061013	127	-0.14	-1.83	1.56E-02	5.63E-02
CELLULAR PROCESS INVOLVED IN REPRODUCTION IN MULTICELLULAR ORGANISM	GO:0022412	78	-0.18	-1.83	2.19E-02	5.61E-02
NEGATIVE REGULATION OF PROTEIN-CONTAINING COMPLEX DISASSEMBLY	GO:0043242	44	-0.23	-1.83	1.43E-02	5.58E-02
HISTONE H4 ACETYLATION	GO:0043967	44	-0.23	-1.83	8.62E-03	5.58E-02
CELL-CELL ADHESION	GO:0098609	192	-0.11	-1.83	1.53E-02	5.48E-02
NEGATIVE REGULATION OF SECRETION BY CELL	GO:1903531	53	-0.21	-1.83	9.90E-03	5.49E-02
PATHWAY	GO:0007200	32	-0.28	-1.83	1.56E-02	5.48E-02
NEGATIVE REGULATION OF DNA-BINDING TRANSCRIPTION FACTOR ACTIVITY	GO:0043433	116	-0.15	-1.84	2.02E-02	5.47E-02
REGULATION OF PROTEIN UBIQUITINATION	GO:0031396	153	-0.13	-1.84	1.21E-02	5.45E-02
MACROAUTOPHAGY	GO:0016236	136	-0.14	-1.84	8.00E-03	5.42E-02
ENDOSOMAL TRANSPORT	GO:0016197	186	-0.12	-1.84	1.58E-02	5.42E-02
REGULATION OF TYROSINE PHOSPHORYLATION OF STAT PROTEIN	GO:0042509	35	-0.26	-1.84	1.43E-02	5.37E-02
REGULATION OF LIPID METABOLIC PROCESS	GO:0019216	170	-0.12	-1.84	1.24E-02	5.39E-02
LEUKOCYTE CHEMOTAXIS	GO:0030595	72	-0.18	-1.85	2.01E-03	5.25E-02
REGULATION OF AMYLOID PRECURSOR PROTEIN CATABOLIC PROCESS	GO:1902991	34	-0.27	-1.85	7.55E-03	5.19E-02
MYELOID CELL DIFFERENTIATION	GO:0030099	115	-0.15	-1.85	9.77E-03	5.21E-02
NEGATIVE REGULATION OF DEFENSE RESPONSE	GO:0031348	130	-0.14	-1.85	2.12E-03	5.22E-02
REGULATION OF ION TRANSMEMBRANE TRANSPORT	GO:0034765	159	-0.12	-1.85	8.06E-03	5.18E-02
REGULATION OF ORGANELLE ASSEMBLY	GO:1902115	155	-0.13	-1.85	1.76E-02	5.15E-02
POSITIVE REGULATION OF PROTEIN BINDING	GO:0032092	50	-0.22	-1.85	1.05E-02	5.11E-02
STRIATED MUSCLE TISSUE DEVELOPMENT	GO:0014706	60	-0.21	-1.85	1.21E-02	5.12E-02
INSULIN RECEPTOR SIGNALING PATHWAY	GO:0008286	37	-0.25	-1.86	9.65E-03	5.11E-02

NUCLEAR CHROMOSOME SEGREGATION	GO:0098813	134	-0.14	-1.86	1.17E-02	5.12E-02
REGULATION OF CELL SHAPE	GO:0008360	91	-0.17	-1.86	6.16E-03	5.12E-02
CELL MORPHOGENESIS INVOLVED IN DIFFERENTIATION	GO:0000904	198	-0.11	-1.86	9.71E-03	5.13E-02
MEMBRANE DOCKING	GO:0022406	64	-0.20	-1.86	1.01E-02	5.13E-02
REGULATION OF PHOSPHATASE ACTIVITY	GO:0010921	51	-0.21	-1.86	7.89E-03	5.14E-02
KIDNEY DEVELOPMENT	GO:0001822	64	-0.20	-1.86	1.24E-02	5.15E-02
REGULATION OF CELL-SUBSTRATE ADHESION	GO:0010810	112	-0.15	-1.86	1.81E-02	5.11E-02
NEGATIVE REGULATION OF PROTEIN DEPOLYMERIZATION	GO:1901880	34	-0.26	-1.86	1.36E-02	5.12E-02
NEGATIVE REGULATION OF LEUKOCYTE DIFFERENTIATION	GO:1902106	51	-0.22	-1.86	1.01E-02	5.13E-02
REGULATION OF LIPID BIOSYNTHETIC PROCESS	GO:0046890	83	-0.18	-1.86	8.25E-03	5.13E-02
RNA DESTABILIZATION	GO:0050779	64	-0.20	-1.86	4.04E-03	5.14E-02
SECOND-MESSENGER-MEDIATED SIGNALING	GO:0019932	106	-0.16	-1.86	1.55E-02	5.07E-02
REGIONALIZATION	GO:0003002	55	-0.22	-1.87	4.00E-03	5.04E-02
PROTEIN AUTOUBIQUITINATION	GO:0051865	57	-0.21	-1.87	8.11E-03	5.01E-02
EPITHELIAL CELL DEVELOPMENT	GO:0002064	53	-0.22	-1.87	9.84E-03	4.98E-02
MYELOID LEUKOCYTE DIFFERENTIATION	GO:0002573	58	-0.21	-1.87	4.18E-03	4.99E-02
PHOSPHATIDYLINOSITOL PHOSPHATE BIOSYNTHETIC PROCESS	GO:0046854	38	-0.25	-1.87	1.82E-02	4.97E-02
NEGATIVE REGULATION OF MONONUCLEAR CELL PROLIFERATION	GO:0032945	42	-0.25	-1.87	6.12E-03	4.95E-02
HEART DEVELOPMENT	GO:0007507	142	-0.14	-1.87	3.98E-03	4.93E-02
NEGATIVE REGULATION OF HEMOPOIESIS	GO:1903707	51	-0.22	-1.87	6.19E-03	4.92E-02
MICROTUBULE CYTOSKELETON ORGANIZATION INVOLVED IN MITOSIS	GO:1902850	90	-0.17	-1.88	1.17E-02	4.90E-02
CYTOSKELETON-DEPENDENT INTRACELLULAR TRANSPORT	GO:0030705	130	-0.14	-1.88	1.33E-02	4.75E-02
INTRACELLULAR STEROID HORMONE RECEPTOR SIGNALING PATHWAY	GO:0030518	35	-0.27	-1.88	1.78E-02	4.73E-02
IMPORT INTO CELL	GO:0098657	81	-0.18	-1.88	8.25E-03	4.74E-02
RENAL SYSTEM DEVELOPMENT	GO:0072001	64	-0.20	-1.89	1.55E-02	4.70E-02
REGULATION OF CELLULAR RESPONSE TO TRANSFORMING GROWTH FACTOR BETA STIMULUS	GO:1903844	72	-0.19	-1.89	1.04E-02	4.69E-02
PROTEIN K48-LINKED UBIQUITINATION	GO:0070936	55	-0.21	-1.89	9.88E-03	4.62E-02
PROCESS	GO:0032434	106	-0.16	-1.89	1.75E-02	4.63E-02
CELLULAR RESPONSE TO STEROID HORMONE STIMULUS	GO:0071383	52	-0.22	-1.89	1.21E-02	4.63E-02
MICROTUBULE-BASED MOVEMENT	GO:0007018	159	-0.13	-1.89	6.32E-03	4.64E-02
CENTRAL NERVOUS SYSTEM NEURON DIFFERENTIATION	GO:0021953	31	-0.29	-1.89	5.88E-03	4.56E-02
NEGATIVE REGULATION OF LYMPHOCYTE PROLIFERATION	GO:0050672	42	-0.25	-1.90	1.61E-02	4.54E-02

NEGATIVE REGULATION OF CELLULAR AMIDE METABOLIC PROCESS	GO:0034249	145	-0.13	-1.90	6.11E-03	4.38E-02
MITOTIC SPINDLE ASSEMBLY	GO:0090307	37	-0.26	-1.90	1.97E-03	4.35E-02
NEGATIVE REGULATION OF CELL MOTILITY	GO:2000146	151	-0.14	-1.90	1.26E-02	4.35E-02
REGULATION OF TRANSFORMING GROWTH FACTOR BETA RECEPTOR SIGNALING PATHWAY	GO:0017015	72	-0.19	-1.90	9.88E-03	4.35E-02
UROGENITAL SYSTEM DEVELOPMENT	GO:0001655	72	-0.20	-1.91	9.26E-03	4.29E-02
SULFUR COMPOUND BIOSYNTHETIC PROCESS	GO:0044272	89	-0.18	-1.91	6.34E-03	4.27E-02
NEGATIVE REGULATION OF CYTOKINE-MEDIATED SIGNALING PATHWAY	GO:0001960	38	-0.26	-1.91	1.24E-02	4.25E-02
NEGATIVE REGULATION OF LOCOMOTION	GO:0040013	165	-0.13	-1.91	2.01E-03	4.20E-02
RECOMBINATIONAL REPAIR	GO:0000725	82	-0.18	-1.92	3.69E-03	4.17E-02
LEUKOCYTE MIGRATION	GO:0050900	119	-0.15	-1.92	1.23E-02	4.17E-02
POSITIVE REGULATION OF TRANSMEMBRANE TRANSPORT	GO:0034764	92	-0.17	-1.92	1.76E-02	4.17E-02
PROTEIN LOCALIZATION TO VACUOLE	GO:0072665	51	-0.23	-1.92	6.07E-03	4.18E-02
RESPONSE TO ALCOHOL	GO:0097305	44	-0.25	-1.92	1.02E-02	4.18E-02
NEGATIVE REGULATION OF TRANSFORMING GROWTH FACTOR BETA RECEPTOR SIGNALING PATHWAY	GO:0030512	38	-0.26	-1.92	1.19E-02	4.17E-02
ORGANELLE TRANSPORT ALONG MICROTUBULE	GO:0072384	58	-0.22	-1.92	6.11E-03	4.18E-02
GLYCOPROTEIN BIOSYNTHETIC PROCESS	GO:0009101	170	-0.13	-1.92	3.96E-03	4.17E-02
CARDIAC SEPTUM DEVELOPMENT	GO:0003279	30	-0.29	-1.92	8.32E-03	4.17E-02
PHOSPHATIDYLINOSITOL BIOSYNTHETIC PROCESS	GO:0006661	98	-0.16	-1.92	8.11E-03	4.13E-02
CELL-CELL ADHESION VIA PLASMA-MEMBRANE ADHESION MOLECULES	GO:0098742	59	-0.21	-1.92	8.08E-03	4.14E-02
POSITIVE REGULATION OF MRNA METABOLIC PROCESS	GO:1903313	94	-0.17	-1.92	8.00E-03	4.13E-02
REGULATION OF MRNA STABILITY	GO:0043488	119	-0.15	-1.93	1.05E-02	4.08E-02
REGULATION OF RESPONSE TO WOUNDING	GO:1903034	73	-0.19	-1.93	6.04E-03	4.05E-02
CELL ACTIVATION INVOLVED IN IMMUNE RESPONSE	GO:0002263	84	-0.18	-1.93	3.96E-03	4.03E-02
CELLULAR RESPONSE TO ALCOHOL	GO:0097306	31	-0.29	-1.93	4.06E-03	4.02E-02
TRANSPORT ALONG MICROTUBULE	GO:0010970	100	-0.16	-1.93	3.85E-03	4.03E-02
POSITIVE REGULATION OF PEPTIDYL-TYROSINE PHOSPHORYLATION	GO:0050731	85	-0.18	-1.93	2.05E-03	4.03E-02
REGULATION OF INTERLEUKIN-8 PRODUCTION	GO:0032677	64	-0.20	-1.93	9.78E-03	4.03E-02
POSITIVE REGULATION OF CELL PROJECTION ORGANIZATION	GO:0031346	161	-0.13	-1.93	3.88E-03	4.04E-02
CELL POPULATION PROLIFERATION	GO:0008283	147	-0.14	-1.93	4.13E-03	4.00E-02
VESICLE DOCKING	GO:0048278	42	-0.25	-1.94	1.00E-02	3.96E-02
DOUBLE-STRAND BREAK REPAIR VIA HOMOLOGOUS RECOMBINATION	GO:0000724	81	-0.19	-1.95	8.42E-03	3.80E-02

REGULATION OF EPITHELIAL TO MESENCHYMAL TRANSITION	GO:0010717	53	-0.22	-1.95	4.23E-03	3.80E-02
INORGANIC ANION TRANSPORT	GO:0015698	46	-0.24	-1.95	1.37E-02	3.80E-02
SPINDLE ASSEMBLY	GO:0051225	71	-0.20	-1.95	4.10E-03	3.79E-02
POSITIVE REGULATION OF RESPONSE TO WOUNDING	GO:1903036	31	-0.29	-1.95	1.27E-02	3.77E-02
REGULATION OF RNA SPLICING	GO:0043484	111	-0.16	-1.95	9.73E-03	3.77E-02
POSITIVE REGULATION OF CARBOHYDRATE METABOLIC PROCESS	GO:0045913	33	-0.28	-1.95	1.18E-02	3.78E-02
NEPHRON DEVELOPMENT	GO:0072006	34	-0.28	-1.95	4.12E-03	3.80E-02
REGULATION OF PROTEIN TYROSINE KINASE ACTIVITY	GO:0061097	50	-0.24	-1.95	7.46E-03	3.75E-02
STEROID HORMONE MEDIATED SIGNALING PATHWAY	GO:0043401	36	-0.28	-1.96	7.83E-03	3.72E-02
POSITIVE REGULATION OF INTERFERON-BETA PRODUCTION	GO:0032728	35	-0.28	-1.96	4.14E-03	3.73E-02
REGULATION OF CYTOSOLIC CALCIUM ION CONCENTRATION	GO:0051480	122	-0.15	-1.96	1.95E-03	3.73E-02
REGULATION OF AUTOPHAGOSOME ASSEMBLY	GO:2000785	33	-0.29	-1.96	0.00E+00	3.62E-02
MITOTIC NUCLEAR DIVISION	GO:0140014	130	-0.15	-1.97	1.95E-03	3.56E-02
REGULATION OF TISSUE REMODELING	GO:0034103	34	-0.29	-1.97	1.01E-02	3.50E-02
POSITIVE REGULATION OF CELL CYCLE PROCESS	GO:0090068	136	-0.15	-1.97	2.09E-03	3.50E-02
REGULATION OF LEUKOCYTE MEDIATED CYTOTOXICITY	GO:0001910	54	-0.23	-1.97	6.09E-03	3.49E-02
NEURON PROJECTION MORPHOGENESIS	GO:0048812	182	-0.13	-1.97	1.03E-02	3.46E-02
REGULATION OF MEMBRANE LIPID DISTRIBUTION	GO:0097035	36	-0.28	-1.98	7.65E-03	3.43E-02
VESICLE-MEDIATED TRANSPORT TO THE PLASMA MEMBRANE	GO:0098876	97	-0.17	-1.98	4.00E-03	3.44E-02
REGULATION OF RECEPTOR-MEDIATED ENDOCYTOSIS	GO:0048259	59	-0.22	-1.98	0.00E+00	3.40E-02
REGULATION OF CHEMOKINE PRODUCTION	GO:0032642	48	-0.24	-1.98	6.01E-03	3.41E-02
REGULATION OF TRANSMEMBRANE RECEPTOR PROTEIN SERINE/THREONINE KINASE SIGNALING PATHWAY	GO:0090092	118	-0.15	-1.98	1.27E-02	3.42E-02
AUTOPHAGOSOME ORGANIZATION	GO:1905037	55	-0.23	-1.98	1.95E-03	3.42E-02
REGULATION OF PROTEIN DEPOLYMERIZATION	GO:1901879	46	-0.25	-1.99	4.11E-03	3.35E-02
REGULATION OF CELL-SUBSTRATE JUNCTION ASSEMBLY	GO:0090109	39	-0.27	-1.99	6.19E-03	3.35E-02
CELL PART MORPHOGENESIS	GO:0032990	196	-0.13	-1.99	9.71E-03	3.24E-02
VESICLE FUSION	GO:0006906	62	-0.21	-1.99	6.09E-03	3.25E-02
NEGATIVE REGULATION OF T CELL PROLIFERATION	GO:0042130	32	-0.30	-1.99	6.09E-03	3.24E-02
REGULATION OF WOUND HEALING	GO:0061041	61	-0.22	-1.99	6.47E-03	3.24E-02
NEGATIVE REGULATION OF CELLULAR COMPONENT MOVEMENT	GO:0051271	156	-0.14	-2.00	9.75E-03	3.21E-02
VESICLE LOCALIZATION	GO:0051648	104	-0.17	-2.00	4.12E-03	3.21E-02
REGULATION OF GENE EXPRESSION, EPIGENETIC	GO:0040029	72	-0.20	-2.00	3.88E-03	3.12E-02

REGULATION OF FOCAL ADHESION ASSEMBLY	GO:0051893	39	-0.27	-2.00	3.94E-03	3.12E-02
REGULATION OF TUMOR NECROSIS FACTOR PRODUCTION	GO:0032680	98	-0.18	-2.01	5.92E-03	3.12E-02
BLOOD CIRCULATION	GO:0008015	122	-0.16	-2.01	1.93E-03	3.10E-02
MRNA DESTABILIZATION	GO:0061157	62	-0.22	-2.01	0.00E+00	3.09E-02
ANION TRANSPORT	GO:0006820	163	-0.13	-2.01	2.01E-03	3.06E-02
ORGANELLE MEMBRANE FUSION	GO:0090174	66	-0.21	-2.01	0.00E+00	2.99E-02
CELLULAR CARBOHYDRATE METABOLIC PROCESS	GO:0044262	72	-0.20	-2.02	7.77E-03	2.99E-02
POSITIVE REGULATION OF RAS PROTEIN SIGNAL TRANSDUCTION	GO:0046579	36	-0.28	-2.02	2.01E-03	2.98E-02
REGULATION OF BIOMINERALIZATION	GO:0110149	42	-0.27	-2.02	4.00E-03	2.95E-02
NEGATIVE REGULATION OF HYDROLASE ACTIVITY	GO:0051346	148	-0.14	-2.02	7.86E-03	2.92E-02
POSITIVE REGULATION OF TUMOR NECROSIS FACTOR PRODUCTION	GO:0032760	63	-0.22	-2.02	0.00E+00	2.94E-02
FC RECEPTOR SIGNALING PATHWAY	GO:0038093	37	-0.28	-2.02	2.02E-03	2.95E-02
NEGATIVE REGULATION OF TRANSMEMBRANE RECEPTOR PROTEIN SERINE/THREONINE KINASE SIGNALING PATHWAY	GO:0090101	57	-0.23	-2.03	4.03E-03	2.90E-02
PHOSPHOLIPID TRANSPORT	GO:0015914	47	-0.26	-2.03	2.15E-03	2.85E-02
RHYTHMIC PROCESS	GO:0048511	78	-0.19	-2.03	0.00E+00	2.86E-02
PROTEIN O-LINKED GLYCOSYLATION	GO:0006493	62	-0.22	-2.03	4.06E-03	2.86E-02
ERBB SIGNALING PATHWAY	GO:0038127	35	-0.29	-2.03	2.05E-03	2.87E-02
POSITIVE REGULATION OF SMALL GTPASE MEDIATED SIGNAL TRANSDUCTION	GO:0051057	39	-0.27	-2.03	2.00E-03	2.86E-02
LIPID TRANSPORT	GO:0006869	125	-0.15	-2.03	0.00E+00	2.87E-02
ADENYLATE CYCLASE-ACTIVATING G PROTEIN-COUPLED RECEPTOR SIGNALING PATHWAY	GO:0007189	49	-0.24	-2.03	3.92E-03	2.88E-02
REGULATION OF BIOMINERAL TISSUE DEVELOPMENT	GO:0070167	42	-0.27	-2.04	4.26E-03	2.85E-02
REGULATION OF CELL KILLING	GO:0031341	66	-0.22	-2.04	4.05E-03	2.85E-02
CYTOSOLIC TRANSPORT	GO:0016482	129	-0.15	-2.04	8.25E-03	2.74E-02
REGULATION OF EPITHELIAL CELL DIFFERENTIATION	GO:0030856	61	-0.23	-2.04	1.96E-03	2.76E-02
POSITIVE REGULATION OF MRNA CATABOLIC PROCESS	GO:0061014	74	-0.20	-2.05	3.96E-03	2.72E-02
LEARNING OR MEMORY	GO:0007611	55	-0.23	-2.05	2.00E-03	2.71E-02
REGULATION OF CELL-MATRIX ADHESION	GO:0001952	69	-0.21	-2.05	0.00E+00	2.70E-02
REGULATION OF NEUROGENESIS	GO:0050767	131	-0.15	-2.05	1.95E-03	2.68E-02
CILIUM ASSEMBLY	GO:0060271	173	-0.13	-2.05	3.83E-03	2.66E-02
CELL PROJECTION MORPHOGENESIS	GO:0048858	187	-0.13	-2.06	0.00E+00	2.63E-02
PROTEIN MODIFICATION BY SMALL PROTEIN REMOVAL	GO:0070646	107	-0.17	-2.06	3.98E-03	2.64E-02

REGULATION OF PEPTIDYL-TYROSINE PHOSPHORYLATION	GO:0050730	128	-0.15	-2.06	5.75E-03	2.63E-02
SISTER CHROMATID COHESION	GO:0007062	35	-0.30	-2.06	4.03E-03	2.60E-02
REGULATION OF CYTOKINESIS	GO:0032465	60	-0.22	-2.06	4.02E-03	2.61E-02
REGULATION OF CELL-SUBSTRATE JUNCTION ORGANIZATION	GO:0150116	44	-0.26	-2.06	3.87E-03	2.62E-02
CELL GROWTH	GO:0016049	30	-0.31	-2.06	6.40E-03	2.62E-02
REGULATION OF CELL JUNCTION ASSEMBLY	GO:1901888	84	-0.19	-2.07	7.89E-03	2.55E-02
ORGANELLE LOCALIZATION BY MEMBRANE TETHERING	GO:0140056	56	-0.24	-2.07	0.00E+00	2.45E-02
REGULATION OF PHOSPHOLIPASE ACTIVITY	GO:0010517	38	-0.28	-2.07	7.52E-03	2.44E-02
FORMATION OF PRIMARY GERM LAYER	GO:0001704	35	-0.29	-2.07	1.96E-03	2.44E-02
PLASMA MEMBRANE BOUNDED CELL PROJECTION MORPHOGENESIS	GO:0120039	185	-0.13	-2.08	3.90E-03	2.44E-02
RECEPTOR METABOLIC PROCESS	GO:0043112	60	-0.23	-2.08	1.97E-03	2.39E-02
REGULATION OF CYTOKINE-MEDIATED SIGNALING PATHWAY	GO:0001959	84	-0.19	-2.08	7.87E-03	2.38E-02
MACROPHAGE ACTIVATION	GO:0042116	32	-0.31	-2.08	2.04E-03	2.38E-02
REGULATION OF LIPASE ACTIVITY	GO:0060191	48	-0.26	-2.09	3.96E-03	2.29E-02
AUTOPHAGOSOME ASSEMBLY	GO:0000045	54	-0.24	-2.09	2.01E-03	2.30E-02
REGULATION OF BONE MINERALIZATION	GO:0030500	33	-0.31	-2.09	4.28E-03	2.28E-02
CELLULAR RESPONSE TO MECHANICAL STIMULUS	GO:0071260	44	-0.27	-2.10	1.96E-03	2.24E-02
HORMONE-MEDIATED SIGNALING PATHWAY	GO:0009755	54	-0.24	-2.10	6.05E-03	2.19E-02
ESTABLISHMENT OF VESICLE LOCALIZATION	GO:0051650	102	-0.18	-2.10	0.00E+00	2.17E-02
ORGANELLE FISSION	GO:0048285	194	-0.13	-2.10	2.09E-03	2.18E-02
REGULATION OF TUMOR NECROSIS FACTOR SUPERFAMILY CYTOKINE PRODUCTION	GO:1903555	103	-0.18	-2.11	2.13E-03	2.15E-02
ENDOPLASMIC RETICULUM TO GOLGI VESICLE-MEDIATED TRANSPORT	GO:0006888	105	-0.17	-2.11	0.00E+00	2.16E-02
RESPONSE TO ENDOPLASMIC RETICULUM STRESS	GO:0034976	167	-0.14	-2.11	0.00E+00	2.16E-02
NUCLEAR DIVISION	GO:0000280	175	-0.14	-2.11	3.84E-03	2.10E-02
CILIUM ORGANIZATION	GO:0044782	189	-0.13	-2.12	5.81E-03	2.06E-02
POSITIVE REGULATION OF PROTEIN UBIQUITINATION	GO:0031398	89	-0.19	-2.12	2.03E-03	2.07E-02
POSITIVE REGULATION OF TUMOR NECROSIS FACTOR SUPERFAMILY CYTOKINE PRODUCTION	GO:1903557	66	-0.23	-2.12	2.00E-03	2.07E-02
PROTEIN DEUBIQUITINATION	GO:0016579	89	-0.19	-2.12	0.00E+00	2.08E-02
I-KAPPAB KINASE/NF-KAPPAB SIGNALING	GO:0007249	40	-0.29	-2.12	0.00E+00	2.05E-02
REGULATION OF SMALL GTPASE MEDIATED SIGNAL TRANSDUCTION	GO:0051056	175	-0.14	-2.13	2.04E-03	2.00E-02
REGULATION OF CELL MORPHOGENESIS	GO:0022604	177	-0.14	-2.13	1.92E-03	1.95E-02
REGULATION OF PATTERN RECOGNITION RECEPTOR SIGNALING PATHWAY	GO:0062207	75	-0.21	-2.14	1.92E-03	1.83E-02

NEGATIVE REGULATION OF ERK1 AND ERK2 CASCADE	GO:0070373	34	-0.31	-2.14	0.00E+00	1.83E-02
NEGATIVE REGULATION OF CELLULAR RESPONSE TO GROWTH FACTOR STIMULUS	GO:0090288	41	-0.29	-2.15	0.00E+00	1.82E-02
VACUOLE ORGANIZATION	GO:0007033	104	-0.18	-2.15	2.04E-03	1.82E-02
GOLGI TO PLASMA MEMBRANE TRANSPORT	GO:0006893	45	-0.27	-2.15	0.00E+00	1.83E-02
NEGATIVE REGULATION OF CELL MIGRATION	GO:0030336	145	-0.15	-2.15	3.95E-03	1.84E-02
MUSCLE TISSUE DEVELOPMENT	GO:0060537	67	-0.22	-2.15	2.02E-03	1.82E-02
REGULATION OF NEURON PROJECTION DEVELOPMENT	GO:0010975	171	-0.14	-2.16	1.98E-03	1.74E-02
REGULATION OF LYMPHOCYTE MEDIATED IMMUNITY	GO:0002706	106	-0.18	-2.16	0.00E+00	1.70E-02
NEGATIVE REGULATION OF VIRAL PROCESS	GO:0048525	73	-0.22	-2.16	1.98E-03	1.69E-02
ESTABLISHMENT OF PROTEIN LOCALIZATION TO VACUOLE	GO:0072666	38	-0.30	-2.17	4.06E-03	1.68E-02
NEGATIVE REGULATION OF CELL-CELL ADHESION	GO:0022408	108	-0.18	-2.17	2.07E-03	1.69E-02
CELLULAR RESPONSE TO EXTRACELLULAR STIMULUS	GO:0031668	128	-0.17	-2.17	0.00E+00	1.67E-02
RESPONSE TO EXTRACELLULAR STIMULUS	GO:0009991	168	-0.14	-2.17	0.00E+00	1.69E-02
LYSOSOMAL TRANSPORT	GO:0007041	94	-0.19	-2.17	0.00E+00	1.66E-02
REGULATION OF RESPONSE TO DNA DAMAGE STIMULUS	GO:2001020	172	-0.14	-2.17	2.03E-03	1.66E-02
NEGATIVE REGULATION OF ADAPTIVE IMMUNE RESPONSE	GO:0002820	36	-0.30	-2.18	1.92E-03	1.67E-02
FOREBRAIN DEVELOPMENT	GO:0030900	72	-0.21	-2.18	0.00E+00	1.68E-02
NEGATIVE REGULATION OF VIRAL GENOME REPLICATION	GO:0045071	46	-0.28	-2.18	2.02E-03	1.68E-02
CELL CHEMOTAXIS	GO:0060326	108	-0.18	-2.18	0.00E+00	1.68E-02
POSITIVE REGULATION OF PROTEIN MODIFICATION BY SMALL PROTEIN CONJUGATION OR REMOVAL	GO:1903322	105	-0.18	-2.18	1.99E-03	1.68E-02
REGULATION OF EXOCYTOSIS	GO:0017157	102	-0.18	-2.18	4.02E-03	1.64E-02
NEGATIVE REGULATION OF LEUKOCYTE MEDIATED IMMUNITY	GO:0002704	41	-0.29	-2.18	0.00E+00	1.64E-02
POSITIVE REGULATION OF PROTEIN CATABOLIC PROCESS	GO:0045732	161	-0.15	-2.19	4.05E-03	1.54E-02
POSITIVE REGULATION OF ENDOCYTOSIS	GO:0045807	54	-0.25	-2.20	6.11E-03	1.56E-02
PHOSPHOLIPID BIOSYNTHETIC PROCESS	GO:0008654	177	-0.14	-2.20	0.00E+00	1.54E-02
PROTEIN DESTABILIZATION	GO:0031648	32	-0.32	-2.20	3.98E-03	1.52E-02
POSITIVE REGULATION OF INTERLEUKIN-1 PRODUCTION	GO:0032732	52	-0.26	-2.20	3.80E-03	1.49E-02
REGULATION OF NERVOUS SYSTEM DEVELOPMENT	GO:0051960	155	-0.15	-2.21	0.00E+00	1.45E-02
NEGATIVE REGULATION OF ADAPTIVE IMMUNE RESPONSE BASED ON SOMATIC RECOMBINATION OF IMMUNE RECEPTORS BUILT FROM IMMUNOGLOBULIN SUPERFAMILY DOMAINS	GO:0002823	31	-0.34	-2.21	2.02E-03	1.45E-02
REGULATION OF TOLL-LIKE RECEPTOR SIGNALING PATHWAY	GO:0034121	50	-0.27	-2.21	2.10E-03	1.44E-02

ADENYLATE CYCLASE-MODULATING G PROTEIN-COUPLED RECEPTOR SIGNALING PATHWAY	GO:0007188	78	-0.22	-2.22	0.00E+00	1.38E-02
COGNITION	GO:0050890	81	-0.21	-2.22	1.86E-03	1.37E-02
REGULATION OF COLD-INDUCED THERMOGENESIS	GO:0120161	97	-0.19	-2.22	1.99E-03	1.38E-02
REGULATION OF VIRAL PROCESS	GO:0050792	129	-0.17	-2.22	0.00E+00	1.35E-02
ORGANOPHOSPHATE ESTER TRANSPORT	GO:0015748	72	-0.22	-2.22	4.00E-03	1.36E-02
NEGATIVE REGULATION OF INNATE IMMUNE RESPONSE	GO:0045824	51	-0.27	-2.22	1.83E-03	1.37E-02
REGULATION OF VIRAL LIFE CYCLE	GO:1903900	116	-0.17	-2.23	2.04E-03	1.36E-02
CELLULAR RESPONSE TO ABIOTIC STIMULUS	GO:0071214	175	-0.15	-2.23	2.05E-03	1.33E-02
PROTEIN LOCALIZATION TO NUCLEUS	GO:0034504	115	-0.18	-2.24	0.00E+00	1.29E-02
NEGATIVE REGULATION OF LYMPHOCYTE MEDIATED IMMUNITY	GO:0002707	34	-0.32	-2.24	1.88E-03	1.28E-02
REGULATION OF INTERLEUKIN-1 PRODUCTION	GO:0032652	71	-0.23	-2.24	0.00E+00	1.29E-02
REGULATION OF ADAPTIVE IMMUNE RESPONSE BASED ON SOMATIC RECOMBINATION OF IMMUNE RECEPTORS BUILT FROM IMMUNOGLOBULIN SUPERFAMILY DOMAINS	GO:0002822	105	-0.18	-2.24	0.00E+00	1.30E-02
RECEPTOR INTERNALIZATION	GO:0031623	40	-0.31	-2.24	2.02E-03	1.31E-02
REGULATION OF RESPONSE TO CYTOKINE STIMULUS	GO:0060759	94	-0.20	-2.24	0.00E+00	1.30E-02
CELLULAR RESPONSE TO ENVIRONMENTAL STIMULUS	GO:0104004	175	-0.15	-2.25	0.00E+00	1.29E-02
REGULATION OF LEUKOCYTE MEDIATED IMMUNITY	GO:0002703	144	-0.16	-2.26	0.00E+00	1.22E-02
CELLULAR RESPONSE TO INTERFERON-GAMMA	GO:0071346	37	-0.31	-2.26	2.02E-03	1.18E-02
TUBE MORPHOGENESIS	GO:0035239	194	-0.14	-2.26	2.04E-03	1.19E-02
REGULATION OF EPITHELIAL CELL MIGRATION	GO:0010632	141	-0.16	-2.27	0.00E+00	1.13E-02
VASCULAR PROCESS IN CIRCULATORY SYSTEM	GO:0003018	99	-0.19	-2.27	1.95E-03	1.12E-02
REGULATION OF PROTEIN-CONTAINING COMPLEX DISASSEMBLY	GO:0043244	76	-0.22	-2.28	1.99E-03	1.10E-02
REGULATION OF ADAPTIVE IMMUNE RESPONSE	GO:0002819	119	-0.18	-2.28	0.00E+00	1.10E-02
PROTEIN TARGETING TO VACUOLE	GO:0006623	32	-0.35	-2.29	0.00E+00	1.03E-02
REGULATION OF MACROAUTOPHAGY	GO:0016241	108	-0.19	-2.29	0.00E+00	1.01E-02
POSITIVE REGULATION OF INTERLEUKIN-6 PRODUCTION	GO:0032755	56	-0.27	-2.29	1.92E-03	1.01E-02
REGULATION OF OSSIFICATION	GO:0030278	39	-0.30	-2.30	2.01E-03	1.00E-02
NEGATIVE REGULATION OF TRANSFERASE ACTIVITY	GO:0051348	170	-0.15	-2.30	0.00E+00	1.01E-02
INOSITOL LIPID-MEDIATED SIGNALING	GO:0048017	46	-0.29	-2.30	0.00E+00	1.00E-02
POSITIVE REGULATION OF CELL CYCLE	GO:0045787	175	-0.15	-2.31	2.07E-03	9.90E-03
POSITIVE REGULATION OF PATTERN RECOGNITION RECEPTOR SIGNALING PATHWAY	GO:0062208	34	-0.33	-2.31	2.08E-03	9.87E-03
ANGIOGENESIS	GO:0001525	104	-0.19	-2.32	2.03E-03	9.56E-03

GLYCEROPHOSPHOLIPID BIOSYNTHETIC PROCESS	GO:0046474	157	-0.16	-2.32	1.96E-03	9.57E-03
MEMBRANE FUSION	GO:0061025	93	-0.21	-2.32	0.00E+00	9.39E-03
TRANSMEMBRANE RECEPTOR PROTEIN SERINE/THREONINE KINASE SIGNALING PATHWAY	GO:0007178	82	-0.22	-2.32	0.00E+00	9.42E-03
POSITIVE REGULATION OF CHEMOKINE PRODUCTION	GO:0032722	37	-0.32	-2.33	0.00E+00	9.25E-03
REGULATION OF CYTOKINE PRODUCTION INVOLVED IN INFLAMMATORY RESPONSE	GO:1900015	33	-0.33	-2.33	2.07E-03	9.30E-03
GOLGI ORGANIZATION	GO:0007030	107	-0.20	-2.33	0.00E+00	8.92E-03
REGULATION OF PROTEIN MODIFICATION BY SMALL PROTEIN CONJUGATION OR REMOVAL	GO:1903320	180	-0.15	-2.34	0.00E+00	8.68E-03
VACUOLAR TRANSPORT	GO:0007034	127	-0.18	-2.35	1.92E-03	8.45E-03
REGULATION OF CELLULAR RESPONSE TO GROWTH FACTOR STIMULUS	GO:0090287	130	-0.18	-2.35	0.00E+00	8.51E-03
PEPTIDYL-THREONINE PHOSPHORYLATION	GO:0018107	56	-0.26	-2.35	0.00E+00	8.30E-03
GLYCEROLIPID BIOSYNTHETIC PROCESS	GO:0045017	167	-0.16	-2.36	1.91E-03	7.71E-03
NEGATIVE REGULATION OF IMMUNE EFFECTOR PROCESS	GO:0002698	66	-0.24	-2.37	0.00E+00	7.48E-03
TRANSFORMING GROWTH FACTOR BETA RECEPTOR SIGNALING PATHWAY	GO:0007179	51	-0.28	-2.37	0.00E+00	7.39E-03
REGULATION OF INTERLEUKIN-1 BETA PRODUCTION	GO:0032651	63	-0.25	-2.37	0.00E+00	7.46E-03
PATTERN SPECIFICATION PROCESS	GO:0007389	84	-0.23	-2.38	1.99E-03	7.28E-03
RESPONSE TO TRANSFORMING GROWTH FACTOR BETA	GO:0071559	70	-0.25	-2.38	0.00E+00	6.93E-03
BLOOD VESSEL MORPHOGENESIS	GO:0048514	132	-0.18	-2.39	1.90E-03	6.94E-03
BEHAVIOR	GO:0007610	95	-0.21	-2.39	0.00E+00	6.77E-03
RESPONSE TO MECHANICAL STIMULUS	GO:0009612	66	-0.25	-2.40	0.00E+00	6.49E-03
DEPHOSPHORYLATION	GO:0016311	150	-0.17	-2.41	1.94E-03	6.27E-03
AGING	GO:0007568	64	-0.26	-2.41	1.95E-03	6.22E-03
VASCULATURE DEVELOPMENT	GO:0001944	152	-0.17	-2.41	2.02E-03	6.24E-03
CELLULAR RESPONSE TO NUTRIENT LEVELS	GO:0031669	119	-0.19	-2.41	0.00E+00	6.25E-03
TOLL-LIKE RECEPTOR SIGNALING PATHWAY	GO:0002224	44	-0.30	-2.42	2.03E-03	6.04E-03
PHOSPHATIDYLINOSITOL-MEDIATED SIGNALING	GO:0048015	45	-0.30	-2.42	0.00E+00	6.03E-03
POSITIVE REGULATION OF TYPE I INTERFERON PRODUCTION	GO:0032481	49	-0.29	-2.43	0.00E+00	5.60E-03
DEVELOPMENTAL GROWTH	GO:0048589	61	-0.26	-2.43	0.00E+00	5.66E-03
NEGATIVE REGULATION OF T CELL ACTIVATION	GO:0050868	67	-0.25	-2.43	0.00E+00	5.71E-03
NEGATIVE REGULATION OF CELL ACTIVATION	GO:0050866	109	-0.20	-2.44	0.00E+00	5.18E-03
RESPONSE TO NUTRIENT LEVELS	GO:0031667	156	-0.16	-2.45	1.96E-03	4.75E-03
PROTEIN DEPHOSPHORYLATION	GO:0006470	109	-0.20	-2.45	0.00E+00	4.77E-03

POSITIVE REGULATION OF IMMUNE EFFECTOR PROCESS	GO:0002699	143	-0.17	-2.45	0.00E+00	4.82E-03
BLOOD VESSEL DEVELOPMENT	GO:0001568	146	-0.18	-2.46	0.00E+00	4.79E-03
NEGATIVE REGULATION OF LYMPHOCYTE ACTIVATION	GO:0051250	83	-0.24	-2.46	0.00E+00	4.76E-03
GROWTH	GO:0040007	62	-0.27	-2.46	0.00E+00	4.79E-03
POSITIVE REGULATION OF INTERLEUKIN-1 BETA PRODUCTION	GO:0032731	47	-0.31	-2.47	0.00E+00	4.37E-03
NEGATIVE REGULATION OF IMMUNE RESPONSE	GO:0050777	121	-0.20	-2.49	0.00E+00	3.96E-03
REGULATION OF INFLAMMATORY RESPONSE	GO:0050727	183	-0.16	-2.49	0.00E+00	4.03E-03
NEGATIVE REGULATION OF PROTEIN KINASE ACTIVITY	GO:0006469	131	-0.19	-2.49	0.00E+00	4.07E-03
REGULATION OF PHAGOCYTOSIS	GO:0050764	62	-0.27	-2.49	0.00E+00	4.14E-03
PHOSPHATIDYLINOSITOL METABOLIC PROCESS	GO:0046488	115	-0.20	-2.49	0.00E+00	4.19E-03
RESPONSE TO INSULIN	GO:0032868	91	-0.22	-2.50	0.00E+00	4.11E-03
RESPONSE TO INTERFERON-GAMMA	GO:0034341	54	-0.29	-2.50	0.00E+00	4.06E-03
CELLULAR RESPONSE TO TRANSFORMING GROWTH FACTOR BETA STIMULUS	GO:0071560	66	-0.26	-2.51	0.00E+00	4.04E-03
NEGATIVE REGULATION OF KINASE ACTIVITY	GO:0033673	144	-0.18	-2.51	0.00E+00	4.12E-03
CELLULAR RESPONSE TO STARVATION	GO:0009267	104	-0.21	-2.52	0.00E+00	4.08E-03
POSITIVE REGULATION OF GTPASE ACTIVITY	GO:0043547	149	-0.18	-2.52	0.00E+00	4.06E-03
NEGATIVE REGULATION OF LEUKOCYTE ACTIVATION	GO:0002695	99	-0.22	-2.52	0.00E+00	4.14E-03
NEGATIVE REGULATION OF MAPK CASCADE	GO:0043409	87	-0.23	-2.52	2.02E-03	4.16E-03
NEGATIVE REGULATION OF LEUKOCYTE CELL-CELL ADHESION	GO:1903038	74	-0.25	-2.52	2.03E-03	4.25E-03
POST-GOLGI VESICLE-MEDIATED TRANSPORT	GO:0006892	86	-0.23	-2.52	0.00E+00	4.20E-03
CELLULAR RESPONSE TO PEPTIDE HORMONE STIMULUS	GO:0071375	103	-0.22	-2.52	0.00E+00	4.22E-03
CIRCULATORY SYSTEM PROCESS	GO:0003013	177	-0.16	-2.53	0.00E+00	4.20E-03
ACTIVATION OF INNATE IMMUNE RESPONSE	GO:0002218	45	-0.32	-2.53	0.00E+00	4.22E-03
REGULATION OF INTERLEUKIN-6 PRODUCTION	GO:0032675	82	-0.24	-2.53	0.00E+00	4.20E-03
CELLULAR RESPONSE TO ORGANIC CYCLIC COMPOUND	GO:0071407	187	-0.16	-2.53	0.00E+00	4.23E-03
INFLAMMATORY RESPONSE	GO:0006954	176	-0.17	-2.54	0.00E+00	3.99E-03
CELLULAR RESPONSE TO INSULIN STIMULUS	GO:0032869	81	-0.24	-2.54	0.00E+00	4.10E-03
GENE SILENCING BY RNA	GO:0031047	46	-0.33	-2.58	0.00E+00	3.54E-03
PEPTIDYL-THREONINE MODIFICATION	GO:0018210	61	-0.28	-2.59	0.00E+00	3.46E-03
NEGATIVE REGULATION OF PROTEIN SERINE/THREONINE KINASE ACTIVITY	GO:0071901	68	-0.27	-2.59	0.00E+00	3.41E-03
RESPONSE TO STARVATION	GO:0042594	115	-0.21	-2.62	0.00E+00	2.68E-03
POSITIVE REGULATION OF INFLAMMATORY RESPONSE	GO:0050729	70	-0.26	-2.63	0.00E+00	2.51E-03
MYELOID LEUKOCYTE ACTIVATION	GO:0002274	73	-0.26	-2.64	0.00E+00	2.43E-03

REGULATION OF VIRAL GENOME REPLICATION	GO:0045069	70	-0.27	-2.65	0.00E+00	2.50E-03
RESPONSE TO GROWTH FACTOR	GO:0070848	188	-0.16	-2.65	0.00E+00	2.59E-03
CELLULAR RESPONSE TO EXTERNAL STIMULUS	GO:0071496	169	-0.17	-2.65	0.00E+00	2.62E-03
POST-TRANSCRIPTIONAL GENE SILENCING BY RNA	GO:0035194	44	-0.34	-2.67	0.00E+00	2.54E-03
REGULATION OF MAP KINASE ACTIVITY	GO:0043405	102	-0.23	-2.67	1.90E-03	2.63E-03
CELLULAR RESPONSE TO GROWTH FACTOR STIMULUS	GO:0071363	175	-0.17	-2.70	0.00E+00	2.41E-03
PROTEIN AUTOPHOSPHORYLATION	GO:0046777	123	-0.21	-2.71	0.00E+00	2.05E-03
RESPONSE TO PEPTIDE HORMONE	GO:0043434	130	-0.21	-2.72	0.00E+00	2.06E-03
RECEPTOR-MEDIATED ENDOCYTOSIS	GO:0006898	108	-0.22	-2.74	0.00E+00	1.65E-03
POSITIVE REGULATION OF INNATE IMMUNE RESPONSE	GO:0045089	88	-0.25	-2.75	0.00E+00	1.72E-03
NEGATIVE REGULATION OF PROTEIN PHOSPHORYLATION	GO:0001933	198	-0.17	-2.75	0.00E+00	1.80E-03
GENE SILENCING	GO:0016458	67	-0.29	-2.77	0.00E+00	1.65E-03
GENE SILENCING BY MIRNA	GO:0035195	38	-0.38	-2.79	0.00E+00	1.38E-03
POSTTRANSCRIPTIONAL GENE SILENCING	GO:0016441	46	-0.35	-2.82	0.00E+00	1.37E-03
PEPTIDYL-SERINE MODIFICATION	GO:0018209	145	-0.21	-2.86	0.00E+00	9.66E-04
CYTOKINE-MEDIATED SIGNALING PATHWAY	GO:0019221	177	-0.19	-2.87	0.00E+00	8.23E-04
INTRACELLULAR RECEPTOR SIGNALING PATHWAY	GO:0030522	73	-0.29	-2.88	0.00E+00	6.47E-04
NEGATIVE REGULATION OF MAP KINASE ACTIVITY	GO:0043407	34	-0.42	-2.89	0.00E+00	5.78E-04
REGULATION OF GTPASE ACTIVITY	GO:0043087	198	-0.18	-2.94	0.00E+00	4.99E-04
RESPONSE TO VIRUS	GO:0009615	189	-0.19	-2.94	0.00E+00	5.40E-04
NEGATIVE REGULATION OF CELL ADHESION	GO:0007162	165	-0.19	-2.98	0.00E+00	4.45E-04
CELLULAR RESPONSE TO PEPTIDE	GO:1901653	140	-0.22	-3.05	0.00E+00	1.64E-04
PEPTIDYL-SERINE PHOSPHORYLATION	GO:0018105	135	-0.22	-3.05	0.00E+00	1.82E-04
PATTERN RECOGNITION RECEPTOR SIGNALING PATHWAY	GO:0002221	67	-0.32	-3.13	0.00E+00	0.00E+00
RESPONSE TO PEPTIDE	GO:1901652	172	-0.21	-3.17	0.00E+00	0.00E+00
PROTEIN POLYUBIQUITINATION	GO:0000209	187	-0.20	-3.18	0.00E+00	0.00E+00
POSITIVE REGULATION OF RESPONSE TO BIOTIC STIMULUS	GO:0002833	110	-0.26	-3.21	0.00E+00	0.00E+00
POSITIVE REGULATION OF DEFENSE RESPONSE	GO:0031349	156	-0.23	-3.33	0.00E+00	0.00E+00
REGULATION OF INNATE IMMUNE RESPONSE	GO:0045088	153	-0.25	-3.57	0.00E+00	0.00E+00
DEFENSE RESPONSE TO VIRUS	GO:0051607	111	-0.29	-3.64	0.00E+00	0.00E+00
DEFENSE RESPONSE TO SYMBIONT	GO:0140546	111	-0.29	-3.65	0.00E+00	0.00E+00
