# Supplementary material for: Whole-Blood Transcriptional Profiles Enable Early Prediction of the Presence of Coronary Atherosclerosis and High-Risk Plaque Features at Coronary CT Angiography
Source: Biomedicines. 2022 Jun 2;10(6):1309. doi: 10.3390/biomedicines10061309 (PMC9219643; doi:10.3390/biomedicines10061309)
Supplement: Supplementary file 1 [file biomedicines-10-01309-s001.zip › Supplementary Table S3.pdf]

Supplementary Table S3. *Results of cell enrichment analysis*

CAD.vs.noCAD

NAME	SIZE	ES	NES	NOM p-val	q-value
T CELLS CD4 NAIVE	45	0.52	4.10	0.00E+00	0.00E+00
T CELLS CD4 MEMORY RESTING	60	0.37	3.36	0.00E+00	0.00E+00
T CELLS CD8	62	0.34	3.18	0.00E+00	0.00E+00
T CELLS REGULATORY (TREGS)	49	0.35	2.93	0.00E+00	0.00E+00
T CELLS FOLLICULAR HELPER	47	0.35	2.87	0.00E+00	0.00E+00
B CELLS MEMORY	48	0.32	2.63	0.00E+00	1.22E-04
T CELLS CD4 MEMORY ACTIVATED	29	0.38	2.46	0.00E+00	4.73E-04
PLASMA CELLS	30	0.30	1.99	7.95E-03	8.80E-03
B CELLS NAIVE	49	0.23	1.90	9.44E-03	1.36E-02
T CELLS GAMMA DELTA	49	0.20	1.67	3.13E-02	3.90E-02
NK CELLS RESTING	53	0.13	1.10	3.18E-01	3.55E-01
NK CELLS ACTIVATED	55	0.12	1.04	3.88E-01	3.92E-01
MACROPHAGES M2	23	-0.18	-1.06	3.65E-01	3.63E-01
DENDRITIC CELLS RESTING	29	-0.19	-1.22	2.20E-01	2.42E-01
DENDRITIC CELLS ACTIVATED	31	-0.21	-1.42	9.93E-02	1.23E-01
MACROPHAGES M1	35	-0.21	-1.50	7.69E-02	1.02E-01
MACROPHAGES M0	23	-0.29	-1.64	3.53E-02	6.01E-02
MAST CELLS ACTIVATED	19	-0.41	-2.11	3.58E-03	5.12E-03
MAST CELLS RESTING	26	-0.37	-2.24	1.39E-03	3.07E-03
MONOCYTES	50	-0.34	-2.86	0.00E+00	0.00E+00
EOSINOPHILS	43	-0.39	-3.05	0.00E+00	0.00E+00
NEUTROPHILS	56	-0.39	-3.48	0.00E+00	0.00E+00
