# Supplementary material for: Whole-Blood Transcriptional Profiles Enable Early Prediction of the Presence of Coronary Atherosclerosis and High-Risk Plaque Features at Coronary CT Angiography
Source: Biomedicines. 2022 Jun 2;10(6):1309. doi: 10.3390/biomedicines10061309 (PMC9219643; doi:10.3390/biomedicines10061309)
Supplement: Supplementary file 1 [file biomedicines-10-01309-s001.zip › Supplementary Table S4.pdf]

Supplementary Table S4. *Results of linear regression analysis: genes significantly associated with high-risk plaque features*

Leaman score

Gene Symbol	R	*P* -value
KMT5A	0.75	0.0001
GSEC	0.70	0.0003
SEMA3B	0.70	0.0003
AC092164.1	0.67	0.0006
LINC01513	0.64	0.0009
ALPL	0.64	0.0010
MAK	0.62	0.0015
N4BP1	0.62	0.0014
KCNJ15	0.61	0.0016
AC040970.1	0.61	0.0016
ROPN1L	0.61	0.0017
AP000919.4	-0.61	0.0016
AL391058.1	-0.61	0.0016
AL603766.1	-0.61	0.0016
SCAT1	-0.61	0.0016
FBXO4	-0.61	0.0015
WDR70	-0.62	0.0008
EXD2	-0.63	0.0012
AC004801.1	-0.66	0.0006

Segment involvement score (SIS)

Gene Symbol	R	*P* -value
KIAA0930	0.70	0.0001
MGST2	0.65	0.0005
FAM20C	0.65	0.0005
SPINT1	0.65	0.0005
FUT4	0.65	0.0005
GNA15	0.63	0.0007
SRA1	0.63	0.0007
PGM5	0.63	0.0010
RNPEP	0.63	0.0008
PLP2	0.62	0.0009
HLA-DRB5	0.61	0.0012
SMARCD3	0.61	0.0010
RBMS2	0.61	0.0013
FXYD6	0.61	0.0013
CTSD	0.60	0.0013
GRK3	0.60	0.0008
MS4A6A	0.60	0.0012
ATP11C	-0.61	0.0012
ADNP2	-0.61	0.0013
FBXO21	-0.61	0.0012
ZSWIM5	-0.61	0.0012
BTN3A3	-0.61	0.0010
TRAV8-3	-0.62	0.0011
CCDC183-AS1	-0.62	0.0011
NCAPD3	-0.63	0.0009
TRAV21	-0.64	0.0008
SMN2	-0.64	0.0007
ZNF337	-0.64	0.0007
AL096816.1	-0.65	0.0006

Segment stenosis score (SSS)

Gene Symbol	R	*P* -value
AC092164.1	0.73	0.0002
GSEC	0.72	0.0002
KMT5A	0.69	0.0004
RGL4	0.64	0.0009
PRMT5	0.63	0.0009
P2RX1	0.63	0.0011
PNPLA1	0.61	0.0016
RCCD1	-0.61	0.0016
CAMKMT	-0.61	0.0015
AP000919.4	-0.63	0.0011
HLCS	-0.63	0.0010
ST3GAL5	-0.64	0.0007
ARMCX7P	-0.64	0.0009
WDR70	-0.64	0.0004
MIR762HG	-0.67	0.0005
SOCS7	-0.68	0.0004
BRMS1L	-0.69	0.0004

AHI1	-0.65	0.0006
NFE2L3	-0.67	0.0004
GALNT12	-0.68	0.0003
TRAV20	-0.70	0.0002
AMIGO2	-0.78	0.0000

	Plaques with density < 150 HU					
	Gene Symbol	R	*P* -value	Gene Symbol	R	*P* -value
	AC092746.1	0.73	0.0002	TOB1-AS1	0.61	0.0019
	LINC02207	0.71	0.0004	CLEC4D	0.61	0.0019
	RNASEL	0.71	0.0002	AFDN	0.61	0.0022
	AC093752.1	0.69	0.0004	Z98886.1	0.61	0.0019
	C5orf30	0.68	0.0005	HSDL2	0.61	0.0017
	PEX2	0.68	0.0004	OSTF1	0.61	0.0020
	HERPUD2	0.67	0.0004	APH1B	0.61	0.0021
	ATP11B	0.66	0.0006	MAP1LC3B	0.61	0.0016
	CEBPB-AS1	0.66	0.0008	SUPT20H	0.61	0.0014
	IL18R1	0.66	0.0008	CDC123	0.61	0.0019
	HMGB2	0.65	0.0008	ZNF100	0.61	0.0018
	KIF1B	0.65	0.0009	SPTY2D1	0.60	0.0024
	GPR141	0.64	0.0013	SRPK1	0.60	0.0019
	GPBP1L1	0.64	0.0009	TPPP3	-0.60	0.0026
	IL18RAP	0.64	0.0014	P2RY11	-0.61	0.0021
	LMTK2	0.63	0.0014	ACSF2	-0.61	0.0019
	SELENOTP1	0.63	0.0014	XKR7	-0.61	0.0022
	POLR2C	0.62	0.0018	AGPAT3	-0.62	0.0015
	DCUN1D3	0.62	0.0019	ALDH1B1	-0.62	0.0018
	ATXN1	0.62	0.0015	EIF3B	-0.62	0.0012
	ZNF396	0.62	0.0018	ADCK1	-0.62	0.0014
	RASGEF1A	0.62	0.0015	C21orf58	-0.64	0.0013
	GADD45A	0.62	0.0018	VASH1	-0.64	0.0014
	BMX	0.62	0.0017	AL009179.1	-0.64	0.0012
	GYG1	0.62	0.0014	IDH3A	-0.64	0.0011
	ZNF608	0.62	0.0020	NLN	-0.65	0.0009
	TMX4	0.62	0.0019	PTPDC1	-0.66	0.0009
	HEATR5A	0.62	0.0015	PTGFRN	-0.66	0.0009
	GTDC1	0.62	0.0019	AC124944.2	-0.70	0.0003
